# Targeting Senescence with Apigenin Improves Chemotherapeutic Efficacy and Ameliorates Age‐Related Conditions in Mice

**DOI:** 10.1002/advs.202412950

**Published:** 2025-04-23

**Authors:** Hongwei Zhang, Qixia Xu, Zhirui Jiang, Rong Sun, Qun Wang, Sanhong Liu, Xin Luan, Judith Campisi, James L. Kirkland, Weidong Zhang, Yu Sun

**Affiliations:** ^1^ Shanghai Frontiers Science Center of TCM Chemical Biology Institute of Interdisciplinary Integrative Medicine Research Shanghai University of Traditional Chinese Medicine Shanghai 201203 P. R. China; ^2^ CAS Key Laboratory of Tissue Microenvironment and Tumor Shanghai Institute of Nutrition and Health Chinese Academy of Sciences Shanghai 200031 P. R. China; ^3^ Department of Discovery Biology Bioduro‐Sundia Zhangjiang Hi‐Tech Park Shanghai 201210 P. R. China; ^4^ Buck Institute for Research on Aging Novato CA 94945 USA; ^5^ Center for Advanced Gerotherapeutics Cedars‐Sinai Medical Center Pacific Design Center West Hollywood CA 90069 USA; ^6^ Division of Endocrinology Diabetes and Metabolism Cedars‐Sinai Medical Center Los Angeles CA 90048 USA; ^7^ State Key Laboratory for Quality Ensurance and Sustainable Use of Dao‐di Herbs Institute of Medicinal Plant Development Chinese Academy of Medical Sciences and Peking Union Medical College Beijing 100193 P. R. China; ^8^ Department of Medicine and VAPSHCS University of Washington Seattle WA 98195 USA

**Keywords:** aging, apigenin, cellular senescence, SASP, senomorphics

## Abstract

Cellular senescence is a cell fate triggered by stressful stimuli and displays a hypersecretory feature, the senescence‐associated secretory phenotype (SASP). Senescent cell burden increases with aging and contributes to age‐related organ dysfunction and multiple chronic disorders. In this study, a large scale screening of a natural product library for senotherapeutic candidates is performed. Apigenin, a dietary flavonoid previously reported with antioxidant and anti‐inflammatory activities, exhibits capacity for targeting senescent cells as a senomorphic agent. This compound blocks the interactions between ATM/p38MAPK and HSPA8, preventing the transition of an acute stress‐associated phenotype (ASAP) toward the SASP. Mechanistically, apigenin targets peroxiredoxin 6 (PRDX6), an intracellular redox‐active molecule, suppressing the iPLA2 activity of PRDX6 and disrupting downstream reactions underlying SASP development. Apigenin reduces the severity of cancer cell malignancy promoted by senescent stromal cells in culture, while restraining chemoresistance when combined with chemotherapy in anticancer regimens. In preclinical trials, apigenin improves the physical function of animals with a premature aging‐like state, alleviating physical frailty and cognitive impairment. Together, the study demonstrates the feasibility of exploiting a natural compound with senomorphic capacity to achieve geroprotective effects by modulating the SASP, thus providing a baseline for future exploration of natural agents for alleviating age‐related conditions.

## Graphical abstract

As a plant‐derived flavonoid, apigenin directly targets PRDX6 to inhibit its PLA2 activity, blocking HSPA8 activation and disturbing its interactions with ATM and p38MAPK in senescent cells. By dampening wide spectrum expression of the SASP, apigenin prevents loss of tissue homeostasis, improves organ function, and alleviates multiple age‐related pathologies as an emerging senomorphic agent.

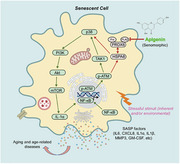

## 1 Introduction

Recent demographic data demonstrate a growing older population with chronic age‐related diseases in the elderly, representing an enormous social and economic burden and an unprecedented challenge to medical healthcare.^[^ ^1^ ^]^ Cellular senescence is one of the central hallmarks of aging and is an emerging therapeutic target for intervening in the aging processes per se and against multiple diseases and disorders.^[^ ^2^, ^3^ ^]^ Although senescence appears to be beneficial in a few physiological settings such as embryonic development, tissue repair and wound healing, increasing lines of evidence suggest that most, if not all, human pathologies can be promoted or caused by the accumulation and persistence of senescent cells in tissues and organs.^[^ ^4^, ^5^ ^]^ Recent preclinical studies have intensively explored the therapeutic potential of targeting senescence‐related pathways in aging and diseases, while more investigations particularly those involving future clinical trials, are essential to understand the efficacy and safety of senotherapy, a novel therapeutic modality that minimizes, or at least, attenuates the detrimental effects of senescent cell accumulation with aging.^[^ ^6^ ^]^

Senotherapeutics, a group of pharmacological agents that specifically target senescence, typically include those attenuating the pro‐inflammatory activity of senescent cells (senomorphics) and those inducing preferential lysis of senescent cells (senolytics).^[^ ^7^, ^8^ ^]^ Elimination of senescent cells markedly increases mobility, improves physical condition, enhances cognitive function and extends overall lifespan in aged or diseased animals.^[^ ^9^, ^10^, ^11^ ^]^ Notably, administration of senolytics also confers additional health benefits in human patients, as evidenced by the case of dasatinib and quercetin (‘D + Q’) combination in clinical trials for diabetic kidney disease (DKD) and idiopathic pulmonary fibrosis (IPF).^[^ ^12^, ^13^, ^14^ ^]^ Initial outcomes from a vanguard clinical trial conducted in early‐stage symptomatic patients with Alzheimer's disease (AD) aimed to assess central nervous system (CNS) penetrance further supported the safety, feasibility and efficacy of these agents and provided mechanistic insights of senolytic effects.^[^ ^15^ ^]^ In contrast to senolysis, attenuating the influence of senescent cells via senomorphic agents represents another effective approach.^[^ ^4^, ^16^ ^]^ An ideal senomorphic candidate should be able to target senescent cells by blocking the development of the senescence‐associated secretory phenotype (SASP), rather than killing senescent cells or eliciting their re‐entry to cell cycle, since rejuvenated cells may still carry a mutation load.^[^ ^17^ ^]^ Phytochemicals, including alkaloids and polyphenols, have demonstrated the ability to target senescent cells, either reducing their burden in vivo and/or modulating their SASP, together enhancing the potential of organ rejuvenation.^[^ ^18^, ^19^ ^]^ Interestingly, some natural agents exhibit both senomorphic and senolytic activities, largely depending on the dose to be used and/or pathological settings intervened against.^[^ ^11^, ^20^ ^]^

The drug development pipeline has been inefficient, as only 15.3% agents in phase 1 clinical trials can advance to FDA approval in the U.S.^[^ ^21^ ^]^ Repurposing drugs formerly approved for clinical use represents an efficient tactic to circumvent drug development pitfalls and to reduce overall costs. Panels of small molecules for drug repurposing studies can be procured and customized, providing an opportunity to adapt drug repurposing screens for alternative indications. As a naturally available flavonoid in fruits and vegetables, apigenin has been reported to have multiple biological activities including antioxidant, anti‐inflammatory, anti‐viral, and anticancer properties.^[^ ^22^ ^]^ By reducing reactive oxygen species (ROS) production while enhancing glutathione and mitochondrial adenosine triphosphate levels, apigenin effectively prevents abnormal mitochondrial distribution and early apoptosis in oocytes, thus minimizing the deterioration of oocyte quality during aging.^[^ ^23^ ^]^ It can significantly reverse glutathione reduction tendencies while decreasing malondialdehyde and catalase activity in the brain, displaying potential anti‐depressant effects in animal models.^[^ ^24^ ^]^ Apigenin can suppress the SASP partially by inhibiting IL‐1α signaling via IRAK1, IRAK4, p38MAPK, and NF‐κB, resulting in declined aggressiveness of human breast cancer cells.^[^ ^25^ ^]^ However, the potential benefits of apigenin across a wider range of age‐related settings, particularly those involving cellular senescence in advanced stages of life, and its potential value in antagonizing pathologies and physical dysfunction, remains underexplored. In this study, we screened a library of natural medicinal agents (NMAs) for potential effects to modulate the survival and pro‐inflammatory activity of human primary cells induced to become senescent in vitro. Our results support the capacity of apigenin for targeting human senescent cells as a novel senomorphic agent. We further unmasked its functional mechanisms of action and pharmacological value in restraining senescence‐associated activities as exemplified by the case of interventions against tumor progression and premature aging. This study demonstrates the benefits of apigenin, a natural compound, in curtailing the pathological influence of senescent cells, thus providing a foundation for its future development and application in clinical conditions including geriatric medicine.

## 2 Results

### 2.1 Drug Screening Discloses the Potential of Apigenin as a Senomorphic Agent

**Figure 1:**
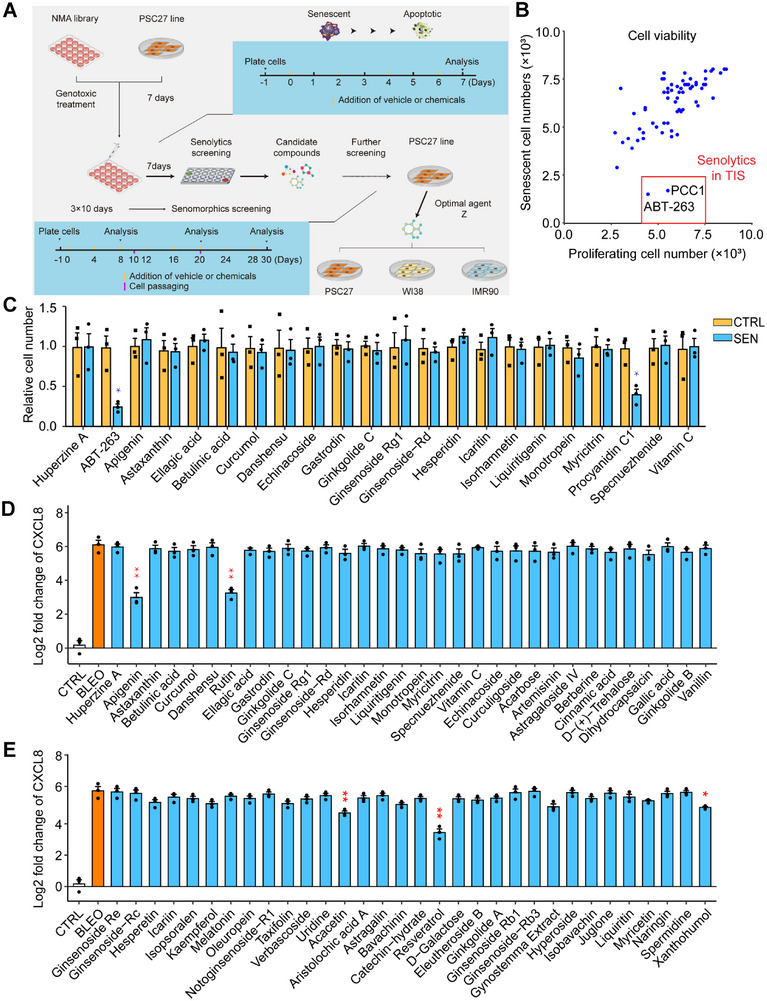
In vitro screening of a NMA library for potential senotherapeutics. A) A schematic representation of the procedure for human cell‐based screening of a NMA library composed of 66 naturally‐derived agents. Screening was first performed for all natural compounds for senomorphics, with the efficacy of potential candidates further validated in multiple human stromal cell lines including PSC27, WI38 and IMR90.^[^ ^85^ ^]^ B) Experimental outputs of senolytics screening after pharmacological treatments in vitro. Natural compounds were assayed by incubation for 3 days at a concentration of 3 µg/mL/agent (ABT‐263 at 1.25 µM) with 5.0x10^3^ cells. Each hit denoted by a blue dot is shown based on the effect to selectively kill SEN but not CTRL cells, and represents the mean of 3 biological replicates. The red‐edged rectangular region shows candidates of senolytics. Note, only ABT‐263 and procyanidin C1 (PCC1), the positive controls, fell in this specific region. C) Evaluation of the efficacy of natural compounds as senolytics in SEN and CTRL counterpart cells. (D‐E) Assessment of the efficacy of natural compounds (D, group A, E, group B) by analyzing the expression of a canonical SASP soluble factor CXCL8. For all datasets, samples were examined after treatment with individual agents in culture for 3 days. NMA, natural medicinal agent. CTRL, control. SEN, senescent. TIS, therapy‐induced senescence. Data in C, D and E are shown as mean ± SD and representative of 3 independent biological replicates. *P* values were calculated by Student's *t‐*tests. *P* > 0.05, *P < 0.05, **P < 0.01, ***P < 0.001, ****P < 0.0001.

In an effort to discover novel natural agents that can effectively regulate senescent cells, we performed comprehensive drug screening with a NMA library comprising 66 natural products (Table S1, Supporting Information), mostly secondary metabolites derived from plants, microbes, and certain animals. To this end, PSC27, a primary normal human prostate stromal cell line, was selected as an experimental cell model for extensive studies. PSC27 develops a canonical SASP upon senescence induced by inherent stress and/or exterior stimuli, including exhaustive replication (replicative senescence, RS), genotoxic chemotherapy, and ionizing radiation (therapy‐induced senescence, TIS).^[^ ^11^, ^26^, ^27^, ^28^ ^]^ We exposed cells to a sub‐lethal dose of bleomycin (BLEO, 50 µg mL^−1^), with a typical cellular senescence phenotype being observed 8–10 d after treatment, as evidenced by elevated senescence‐associated β‐galactosidase (SA‐β‐Gal) staining positivity, decreased BrdU incorporation and increased DNA damage repair (DDR) foci (Figure S1A–C, Supporting Information). To streamline our approach, we designed a screening procedure to evaluate the effects individual natural agents exerted on the in vitro viability of senescent cells and expression of their SASP (**Figure** **1**A).

Induction of apoptosis in senescent cells represents a central feature and the most valuable capacity of senolytics, as exemplified by studies involving senolytic compounds such as ABT‐263 (navitoclax), ABT‐737 and procyanidin C1 (PCC1).^[^ ^11^, ^29^, ^30^ ^]^ Alternatively, a major advantage of senomorphics, for example, rutin and resveratrol,^[^ ^28^, ^31^ ^]^ is the ability to selectively downregulate the SASP expression. To determine the relevant anti‐senescence potentials in these NMA components, we analyzed the effect of these natural agents on senescent PSC27 cells, by first confirming its experimental feasibility as a cell‐based model for geroprotective compound screening purposes. Preliminary results indicated that some agents either killed senescent cells (while preserving their proliferating counterparts), or suppressed the expression of CXCL8, a canonical SASP factor, thus further validating the potential and appropriateness of PSC27 for extended investigations (Figure 1B–E, Figure S1D,E, Supporting Information). Despite a thorough screening of the NMA library, we failed to discover any new senolytic agents, which are supposed to functionally resemble the positive controls ABT‐263 and PCC1 (Figure 1B,C; Figure S1D,E, Supporting Information). The results also implied the rareness of natural senolytic agents and the challenge in expanding the present reservoir of senotherapeutic agents particularly those of senolytic potential. Nevertheless, we noticed that several natural compounds generated a significant senomorphic effect, which warrants in‐depth studies (Figure 1D,E).

Among the medicinal agents, rutin, resveratrol, and apigenin displayed strong senomorphic capacity (Figure 1D,E). Resveratrol is a natural product with SASP‐inhibiting activity and targets PI3K/Akt signaling pathway.^[^ ^31^ ^]^ As another phytochemical molecule recently unraveled with senomorphic efficacy, rutin attenuates the acute stress‐associated phenotype (ASAP) and affects the SASP development afterward, mainly through disrupting the interactions between ATM and HIF‐1α as well as between ATM and TRAF6.^[^ ^28^ ^]^ In this study, we chose to specifically focus on apigenin, a plant‐derived natural flavonoid, which can downregulate the expression of typical SASP factors upon bleomycin‐induced senescence in BJ fibroblasts by inhibiting NF‐κB p65 activity through targeting IRAK1/IκBα signaling and IκBζ expression, with in vivo activity of SASP suppression identified in the kidney of aged rats.^[^ ^32^ ^]^ Apigenin can also restrain the SASP by blockade of IL‐1α signaling through IRAK1, p38MAPK, and NF‐κB, thus alleviating pro‐tumorigenic effects of the SASP in breast tumors.^[^ ^25^ ^]^ However, despite these intracellular activities, which are relatively downstream in the SASP signaling network, a wider influence of apigenin on the transcriptome‐wide expression of senescent cells, especially the more upstream or even direct targets after exposure of these cells to apigenin, remains basically unknown.

### 2.2 Apigenin Prominently Attenuates the SASP but not Cellular Senescence

**Figure 2:**
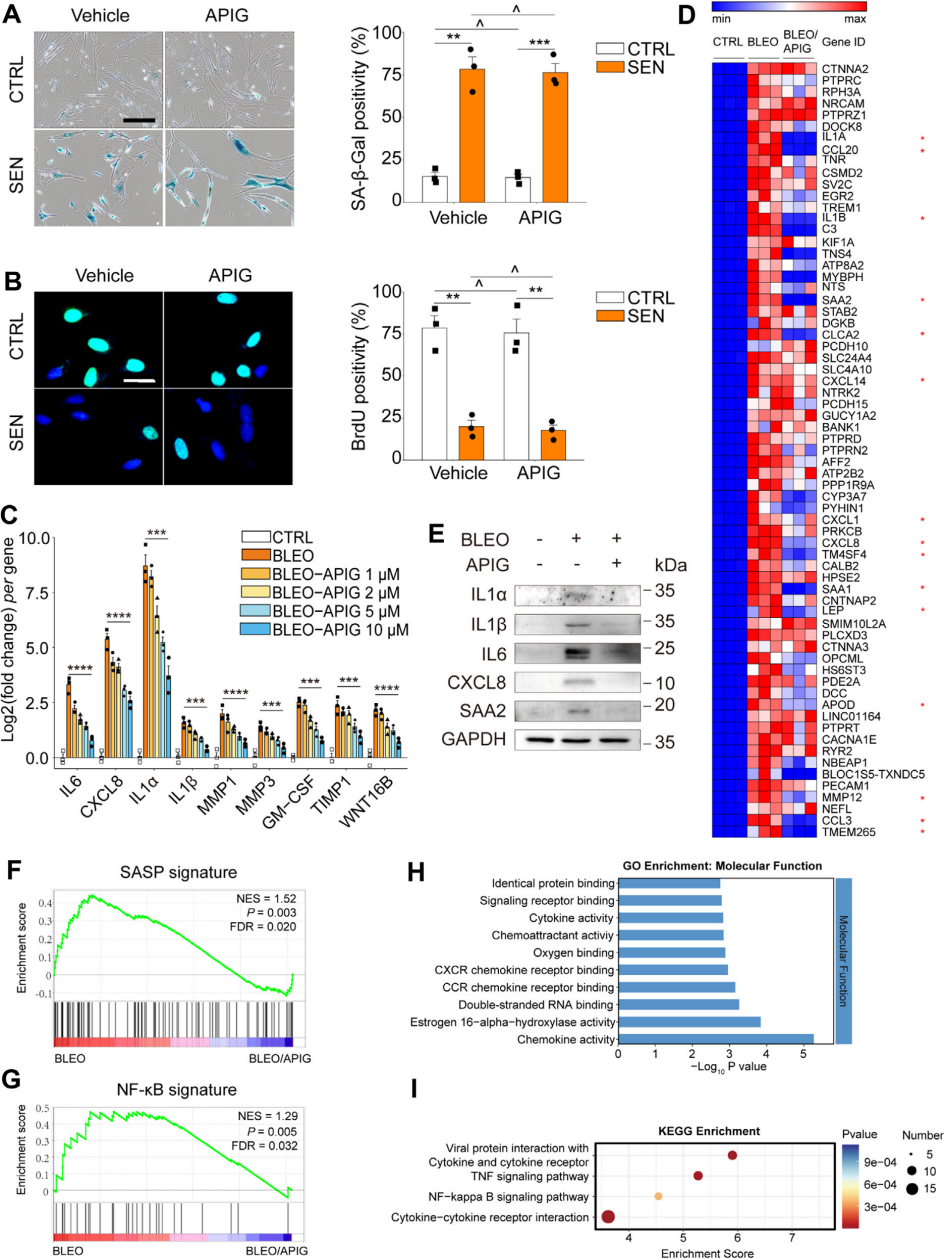
Characterization of the senomorphic potential of apigenin. A) Senescence examination by SA‐β‐Gal staining of PSC27 cells. Left, representative images. Scale bar, 20 µm. Right, statistics. B) DNA synthesis assessment by BrdU incorporation. Left, representative images, Scale bars, 5 µm. Right, statistics. C) Quantitative analysis of the expression of canonical SASP factors at the transcriptional level upon BLEO‐induced senescence and treatment by apigenin at increasing concentrations. D) Hierarchical clustering heatmap depicting top human genes (67) that were significantly upregulated in senescent stromal cells but downregulated by apigenin treatment (10 µM). Red stars, representative SASP factors. E) Immunoblot analysis of SASP factors at protein levels of stromal cells exposed to BLEO and/or apigenin. GAPDH, loading control. F) GSEA profiling of enrichment scores of soluble factors across the SASP spectrum. G) GSEA profiling of enrichment scores of factors related to NF‐κB pathway. H) A bar chart shows the GO molecular function of differentially expressed genes (67) depicted in (d). I) KEGG pathway enrichment analysis of differentially expressed genes (67) depicted in (D). Unless specially noted, data in A, B and C are shown as mean ± SD and representative of 3 independent biological replicates with *P* values calculated by Student's *t*‐tests. *P* > 0.05, *P < 0.05, **P < 0.01, ***P < 0.001, ****P < 0.0001.

Given that apigenin inhibits the expression of CXCL8, a SASP hallmark factor, we further asked whether it downregulates the majority of SASP factors, or even counteracts cellular senescence. Therefore, we performed SA‐β‐Gal staining and BrdU incorporation assays in vitro to determine the anti‐senescence effects of apigenin. The data indicated that SA‐β‐Gal staining profiles and mitotic inactivity remained largely unchanged, in both proliferating cells and their senescent counterparts (**Figure** **2**A,B).

A subset of hallmark SASP factors, including IL6, CXCL8, IL1α/1β, MMP1/3, GM‐CSF, TIMP1, and WNT16B displayed a dose‐dependent decline upon exposure of senescent cells to apigenin, with a concentration of 10 µM appearing most effective (Figure 2C). Furthermore, transcriptomic data from RNA‐seq showed that expression of the canonical SASP factors was markedly upregulated upon cellular senescence but significantly downregulated upon apigenin treatment (Figure 2D). Immunoblot assays indicated that apigenin abrogated SASP expression in senescent cells, as evidenced by the decreased expression of IL1α, IL1β, IL6, CXCL8, and SAA2 (Figure 2E). Given the striking effect of apigenin in restraining the SASP expression, we performed GSEA analysis for signatures of SASP and NF‐κB, the latter plays a significant role in activating the ASAP and sustaining the SASP.^[^ ^27^ ^]^ Our results suggest that both molecular signatures were apparently suppressed upon apigenin treatment (Figure 2F,G).

Gene ontology (GO) enrichment analysis of apigenin‐downregulated genes indicated that chemokine activity, estrogen 16‐alpha‐hydroxylase activity, double‐stranded RNA binding and CCR/CXCR chemokine receptor binding activities were among the most significantly suppressed molecular functions (MF) (Figure 2H). In terms of biological processes (BP), type I interferon signaling pathway, defense response to virus, response to viruses, and inflammatory response were most dramatically affected (Figure S2A, Supporting Information). The representative cellular components (CC) most associated with downregulated genes were those residing in extracellular region and space, although some proteins were transported to plasma membrane, platelet dense granule lumen, as well as calcium‐ and calmodulin‐dependent protein kinase complex (Figure S2B, Supporting Information). KEGG pathway analysis suggested that cytokine‐cytokine receptor interaction, viral protein interaction with cytokine receptor, TNF signaling and NF‐κB signaling pathways were most significantly inhibited upon treatment of senescent cells by apigenin (Figure 2I). Altogether, all these data confirm the capacity of apigenin to suppress the expression of pro‐inflammatory cytokines and chemokines, a subset of central SASP factors, starting from the transcriptomic level.

As the vast majority of SASP components are soluble factors secreted into the extracellular space, we collected the conditioned media (CM) from human stromal cells and analyzed them with mass spectrometry (MS). The resulting data suggest that among the SASP factors upregulated in senescent cells, a large number exhibited a reversal change pattern, namely downregulation, upon apigenin treatment (Figure S2C,D, Supporting Information). Some typical SASP components such as IL6, CXCL1/3/8, CSF1, CCL2, MMP1/2/3, IGFBP7, GDF15, HGF, AREG, and EREG, which are frequently reported by literatures to be secreted by human senescent stromal cells,^[^ ^33^, ^34^ ^]^ appeared in the top list with statistical significance. Therefore, as a senomorphic agent, apigenin can restrain the expression of a large subset of SASP factors, although not the full spectrum SASP, implying the presence of other signal transduction pathway(s) involved in mediating signals that support the SASP development.

To expand, we further assessed the effect of apigenin on senescent cells induced by other means, including inherent stress or environmental stimulation, as illustrated by replicative senescence (RS) and ionizing radiation (RAD), respectively. As expected, apigenin holds the potential to diminish the expression of the majority of canonical SASP factors, regardless of the types of senescence‐inducing modality (Figure S2E,F, Supporting Information). Additionally, we performed further experiments with WI38 and IMR90, two typical fibroblast lines derived from human embryonic lung tissues. The results largely resembled that of PSC27, a human prostate‐derived stromal line, suggesting that apigenin‐exerted suppression of the SASP is essentially organ type‐independent (Figure S2G,H, Supporting Information). Together, our studies demonstrated a salient capacity of apigenin in restraining SASP expression, which is neither senescence type‐ nor organ type‐dependent.

### 2.3 Apigenin Dampens the SASP Expression by Interfering with Interactions of ATM and p38MAPK with HSPA8

**Figure 3:**
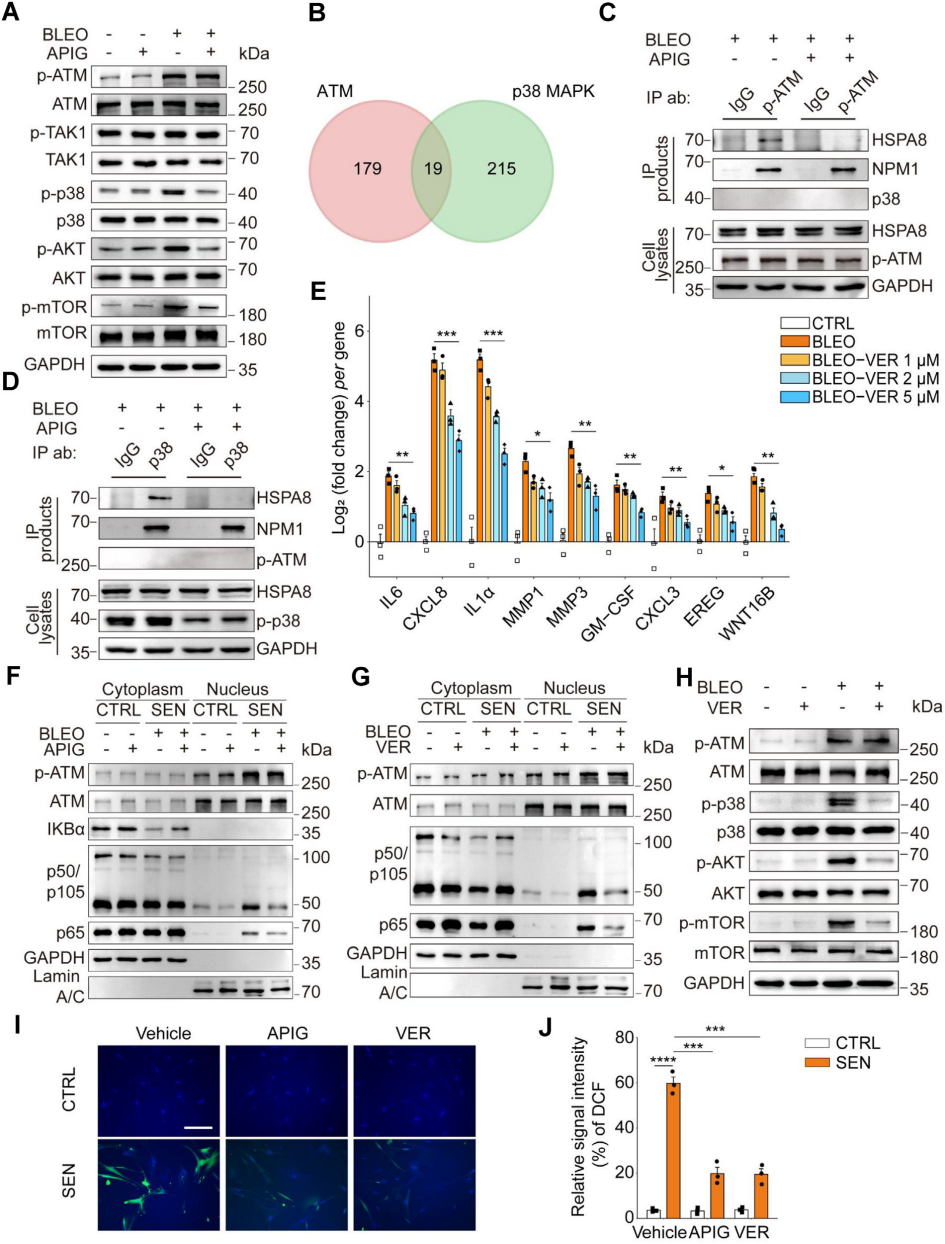
Apigenin restrains SASP expression by interfering with DDR signal transduction in senescent cells. A) Immunoblot analysis of DDR signaling‐related molecules involved in regulation of SASP expression in mammalian cells. After 7 d incubation with BLEO and/or apigenin, PSC27 cells were lysed for examination. GAPDH, loading control. B) Venn plot depicting 19 mutually interacting molecules shared by both ATM and p38MAPK uncovered by bioinformatics mining. C) Immunoprecipitation (IP) assay coupled with immunoblot analysis to detect protein‐protein interactions. PSC27 cells were treated with BLEO (50 µg/mL) for 12 h to induce senescence, in the absence or presence of apigenin in culture for 7 d. Cells were then lysed for IP with IgG or anti‐p‐ATM, with HSPA8, NPM1, p38MAPK and p‐ATM in immunoprecipitates (IPs) and/or inputs examined. GAPDH, intracellular protein loading control. D) IP coupled with immunoblot to probe protein‐protein interactions. Sample preparation was performed as described in (C). Lysates were immunoprecipitated with IgG or anti‐p38MAPK, with HSPA8, NPM1, p‐ATM and p‐p38 in IPs and/or inputs examined. E) Quantitative assessment of canonical SASP factor expression at transcriptional level upon senescence induced by BLEO at increasing concentrations of VER155008 (VER), a HSPA8 inhibitor. F) Immunoblot analysis of ATM, p50/105, p65 and IKBα translocation between nucleus and cytoplasm upon treatment with apigenin. Lamin A/C and GAPDH, loading controls for nucleus and cytoplasm, respectively. G) Immunoblot analysis of ATM, p50/105, p65 and IKBα translocation between nucleus and cytoplasm. Samples preparation was similar to that described in (f), except that VER155008 was applied instead of apigenin. Lamin A/C and GAPDH, loading controls for nuclei and cytoplasm, respectively. H) Immunoblot assessment of DDR signaling‐related molecules involved in regulation of SASP expression. After 7 d incubation with BLEO with or without VER, PSC27 cells were lysed for analysis. GAPDH, loading control. I) Measurement of ROS level via 2′‐7′‐dichlorodihydrofluorescein diacetate (DCFH‐DA), a ROS‐sensitive probe, in proliferating or senescent cells in the presence or absence of apigenin in culture 1 d afterward. Representative images are shown. Scale bar, 20 µm. J) Comparative statistics of ROS signals imaged by the DCFH‐DA fluorescent probe. DDR, DNA damage response. BLEO, bleomycin. ROS, reactive oxygen species. Unless specially noted, data in E and J are shown as mean ± SD and representative of 3 independent biological replicates, with *P* values calculated by Student's *t*‐tests. *P* > 0.05, *P* > 0.05, *P < 0.05, **P < 0.01, ***P < 0.001, ****P < 0.0001.

Given the critical role of apigenin in suppressing the SASP, we next queried its functional mechanism in targeting senescent cells. To examine the potential effect of apigenin on DDR signaling, which is usually the driving force of a persistent SASP, we first investigated the extent of molecular pathway perturbation in senescent cells upon exposure to apigenin. Induction of senescence by BLEO caused notable activation of ATM, a central modulator of the DDR signaling cascade, as evidenced by immunoblot assays (**Figure** **3**A). As a response to external challenge or endogenous stimuli, proliferating cells first develop an acute stress‐associated phenotype (ASAP), characterized by phosphorylation and nucleus‐to‐cytoplasm translocation of ATM, TRAF6 auto‐ubiquitination and TAK1 activation, a series of prompt intracellular reactions to damage before senescence markers become evident^[^ ^27^ ^]^ (Figure 3A). Upon cell genotoxic treatment, TAK1, a key cytoplasmic kinase that modulates the SASP, was rapidly phosphorylated, a change allowing its functional involvement in the transition of the ASAP toward the SASP via dual feedforward mechanisms.^[^ ^27^ ^]^ We further observed phosphorylation/activation of p38MAPK, a molecule downstream of TAK1, followed by engagement of PI3K/Akt/mTOR, a signaling pathway that supports the formation of a chronic secretory phenotype, the SASP. However, in the presence of apigenin, these sequential molecular events were generally abrogated, although phosphorylation of ATM and TAK1 remained largely unaffected. In accordance with this molecular pattern, we would surmise that the potential target(s) of apigenin may be mechanistically located downstream of ATM and/or TAK1, but upstream of p38MAPK, PI3K/Akt/mTOR and other factors that may be implicated in the transition of the ASAP to the SASP during cellular senescence.

To unravel the mechanism regulating the function of apigenin in targeting senescent cells, we conducted a proteome‐scale profiling of interactors with capacity to interact with both ATM and p38MAPK, two molecules that are known to be involved in the transduction of signals that allow development of the SASP but lack direct correlation in senescent cells. Bioinformatics suggested 198 ATM‐ and 234 p38MAPK‐uniquely interacting molecules in human cells, with 19 such interactors shared by both ATM and p38MAPK (Figure 3B, Figure S3A,B, Supporting Information). Among these interactor candidates, we noticed that HSPA8 and NPM1, intracellular molecules that are involved in cellular stress responses, particularly senescence,^[^ ^35^, ^36^ ^]^ and hold the potential to mediate the interaction between ATM and p38MAPK, thus deserving further investigation. p‐ATM‐mediated co‐immunoprecipitation (Co‐IP) revealed that each of HSPA8 and NPM1 can directly interact with p‐ATM and p38MAPK. However, the interaction of HSPA8, but not NPM1, with p‐ATM or p38MAPK was remarkably attenuated by apigenin, indicating that apigenin specifically hinders the interaction of HSPA8, rather than other molecules such as NPM1, with p‐ATM and p38MAPK (Figure 3C,D).

HSPA8, an important member of the heat shock protein 70 family, is functionally involved in chaperone‐mediated autophagy, one of the main pathways of the lysosome‐autophagy proteolytic system, whereas deficient protein degradation compromises cellular proteostasis and activates signaling pathways to culminate in induction of cellular senescence, a major feature of aging.^[^ ^37^ ^]^ HSPA8 activates NF‐κB signaling by destabilizing IκBβ protein in the absence of lipopolysaccharide (LPS) or facilitating its nuclear translocation in the presence of LPS, an activity that can be synergized by thioredoxin domain‐containing protein 5 (TXNDC5) to exacerbate the inflammatory phenotype of synovial fibroblasts in rheumatoid arthritis via NF‐κB signaling.^[^ ^38^ ^]^ HSPA8 is also essential for VEGF‐induced Akt phosphorylation, while downregulation of HSPA8 abolishes Akt phosphorylation.^[^ ^39^ ^]^ Therefore, we speculate that HSPA8 plays a key role in orchestrating the functional involvement of a number of factors responsible for maintaining senescence and SASP development. To test this hypothesis, we evaluated expression of a series of SASP factors or senescence‐associated markers. Our study indicated that in the presence of VER155008 (hereafter VER), a selective HSPA8 inhibitor, expression of canonical SASP factors was dampened in a concentration‐dependent manner (Figure 3E). Further data indicated that cellular senescence was accompanied by IκBα degradation in the cytoplasm as well as cytoplasm‐to‐nucleus translocation of NF‐κB subunits (p65, p50), a tendency that was generally reversed upon apigenin or VER treatment, suggesting a mechanism shared by these agents in curtailing activation of the NF‐κB complex (Figure 3F,G). Interestingly, treatment with VER also suppressed the occurrence of a cascade of molecular events including activation of p38MAPK and engagement of PI3K/Akt/mTOR upon genotoxic stress (Figure 3H). We further observed markedly reduced interactions between ATM/p38MAPK and HSPA8 upon inhibition of HSPA8 activity with the small molecule inhibitor VER, as evidenced by data from IP and immunoblot assys (Figure S3C,D, Supporting Information).

Thus, interfering with crosstalk between ATM and its critical targets such as HSPA8 and p38MAPK, exemplified by the natural agent apigenin, is able to attenuate SASP expression while maintaining cellular senescence or growth arrest, a distinctive feature that is basically in line with effects of senomorphics. As the majority of natural flavonoid polyphenols have antioxidant activities, including reducing ROS overproduction and alleviating oxidative stress, we next asked whether apigenin has a similar effect in senescent cells. When exposed to a genotoxic agent (BLEO) for 8–10 d, stromal cells had markedly elevated ROS levels compared with their proliferating counterparts. However, in the presence of apigenin (or VER as an experimental control), production of ROS was significantly inhibited, largely consistent with the oxidative radical‐neutralizing capacity of apigenin, although the ROS level remained largely unaffected in normal cells (Figure 3I,J).

### 2.4 Apigenin Directly Targets PRDX6 to Attenuate its iPLA2 Activity and SASP Expression in Senescent Cells

**Figure 4:**
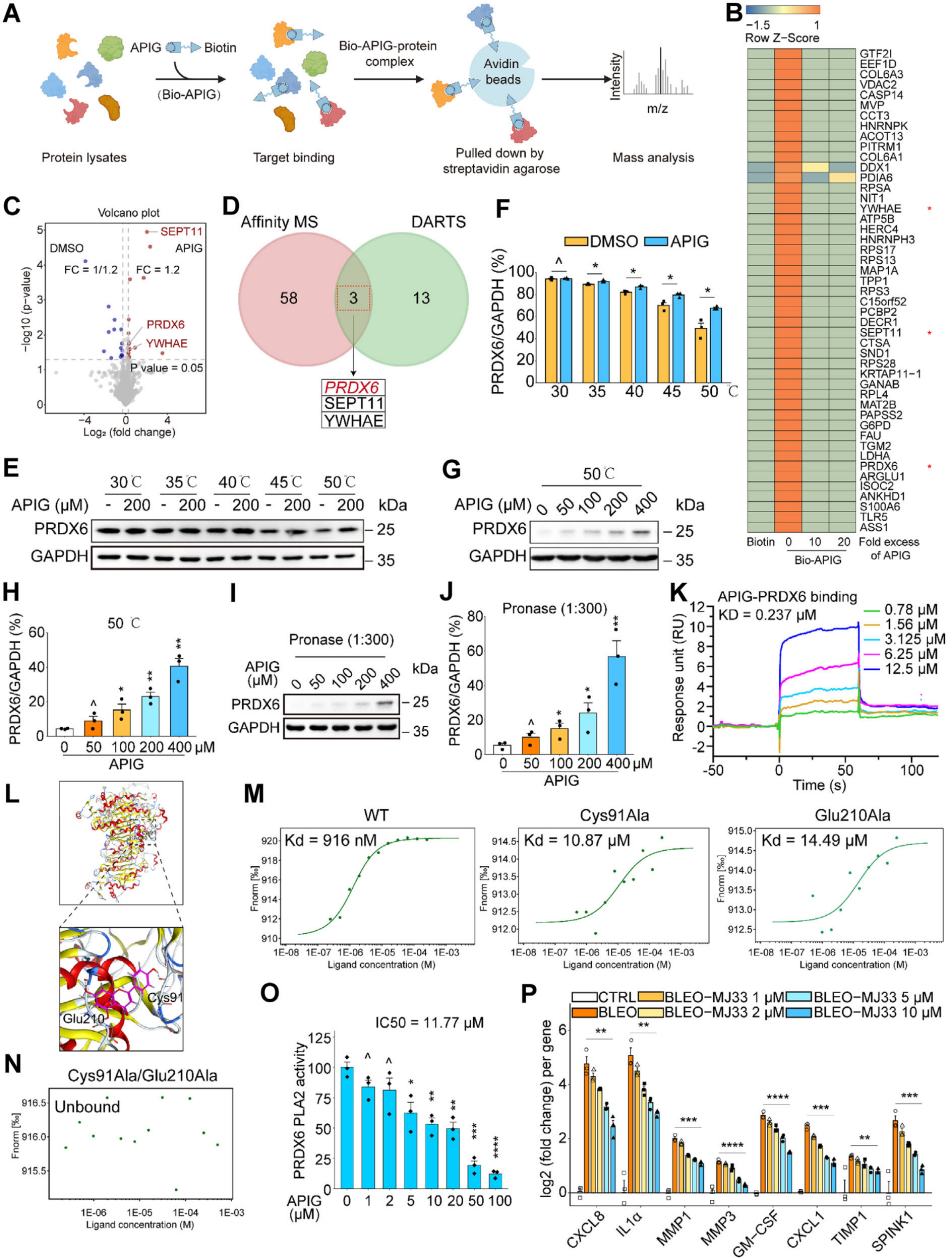
Apigenin binds to PRDX6, inhibits its iPLA2 activity, and dampens the SASP development. A) A schematic illustration of the technical procedure of affinity‐based MS that applied a Bio‐APIG probe for target enrichment, followed by streptavidin‐agarose pulldown. This approach allows to discover target(s) of apigenin with protein lysates of senescent cells. B) Heatmap displaying top human proteins (47) enriched by Bio‐APIG probe but competitively inhibited by a 10‐ or 20‐fold excess of unlabeled apigenin. Red stars denote proteins alternatively identified by the DARTS approach, which was technically combined with MS assay. C) Volcano plot displaying the significantly and differentially regulated proteins (red, upregulated, blue, downregulated) in lysates of senescence cells by DARTS in the presence or absence of apigenin. Denoted proteins were also identified by Bio‐APIG probing. D) Venn diagram depicting the 3 potential target proteins identified by both Bio‐APIG probing and DARTS strategies. E) CETSA assay appraisal of the thermal stabilization of PRDX6 upon incubation with apigenin at a gradient range of temperatures from 30 to 50 °C in protein lysates of PSC27 cells. F) Comparative statistics of the thermal stabilization of PRDX6 as depicted in (E). G) Assessment of thermal stability of PRDX6 over a range of concentrations from 0 to 400 µM of apigenin when examined at a fixed temperature of 50 °C. H) Comparative statistics of the stabilization of PRDX6 over a range of concentrations of apigenin at 50 °C as depicted in (g). I) Evaluation of proteolysis stability (pronase) of PRDX6 over a range of concentrations from 0 to 400 µM of apigenin when the ratio of enzyme to protein was fixed at 1: 300. J) Comparative statistics of the enzymatic stabilization of PRDX6 at a gradient range of concentrations of apigenin as depicted in (I). K) Demonstration that PRDX6 directly binds to apigenin. In SPR assay, PRDX6 was treated by apigenin over a range of concentrations from 0.78 to 12.5 µM. KD value is shown beside the traces. L) In silico molecular modeling of apigenin potential bound to Cys91 and Glu210 of PRDX6. Reported X‐ray diffraction microscopy structure (PDB ID, 5B6M) was applied to perform molecular docking, with Cys91 and Glu210 highlighted in the structure. (M,N) The interaction between apigenin and PRDX6 or its functionally disruptive mutants (m) overexpressed in cells with GFP‐tag was assessed by the cellular microscale thermophoresis analysis (MST). Alternatively, the interaction between apigenin and a double site mutant (Cys91/Ala‐Glu210/Ala) of PRDX6 was analyzed (n). O) Evaluation of iPLA2 activity upon treatment of cell lysates containing overexpressed PRDX6 with apigenin and measurement of fluorescence at a wavelength of 515 nm. P) Quantitative assay of the expression of SASP soluble factors at transcriptional level upon senescent cell treatment with increasing concentrations of MJ33 (1–10µM), a selective PRDX6‐iPLA2 inhibitor. MS, mass spectrometry. Bio‐APIG, biotin‐apigenin. DARTS, drug affinity responsive target stability. SPR, surface plasmon resonance. iPLA2, PRDX6 phospholipase A2. Data in (F, H, J, O, and P) are shown as mean ± SD and representative of 3 independent biological replicates, with *P* values calculated by Student's *t*‐tests. *P* > 0.05, *P* > 0.05, *P < 0.05, **P < 0.01, ***P < 0.001, ****P < 0.0001.

Although our data suggested that apigenin inhibits SASP expression through disrupting the interactions of HSPA8 with other critical factors that mediate SASP signal transduction, resulting in a blocked transition of ASAP to the SASP (Figure 3C–E), the potential direct target(s) of apigenin in senescent cells causing a senomorphic effect remains unclear. Therefore, we applied two affinity‐based approaches, biotin‐tagged apigenin (hereafter Bio‐APIG) and drug affinity responsive target stability (DARTS),^[^ ^40^, ^41^ ^]^ to reveal the potential direct target(s) of apigenin (**Figures** **4**A, S4A–C, Supporting Information). In the probe‐based route, upon incubation with senescent cell lysates, Bio‐APIG bound to proteins before streptavidin‐agarose beads were applied to pull down the Bio‐APIG‐protein complex for assay by MS. We observed 61 potential target proteins, which were identified with distinct enrichment by Bio‐APIG but competitively inhibited by unlabeled apigenin (unique peptides ≥2, LFQ intensity ≥1.5 for enrichment or ≤0.67 for competitive inhibition), also evidenced by protein sliver staining (Figure 4B, Figure S4D, Supporting Information). To further discover bona fide target proteins, we followed another approach, namely DARTS, to substantiate the findings by target identification. The data indicated that 16 proteins were markedly upregulated upon incubation with apigenin, relative to vehicle (unique peptides ≥2, LFQ intensity ≥1.2) (Figure 4C). Among the proteins (PRDX6, SEPT11, and YWHAE) identified by both approaches, we speculated that apigenin may directly bind to PRDX6, a hypothesis that needs to be experimentally proved (Figure 4D).

We next interrogated the biophysical properties underpinning the interaction between PRDX6 and apigenin. As an approach to analyze protein thermal stabilization after ligand binding, cellular thermal shift assay (CETSA) was performed in a temperature range from 30 to 50 °C. We observed an increase of the stability of PRDX6 upon incubation with apigenin compared to the vehicle (Figure 4E,F). We further examined the binding effect by performing CETSA at concentrations ranging from 0 to 400 µM of apigenin either at the fixed temperature of 50 °C, or the ratio of enzyme (pronase) to protein fixed at 1: 300. Unsurprisingly, there appeared a marked stability elevation of PRDX6 as reflected by both approaches in a concentration‐dependent manner (Figure 4G–J). Results from surface plasmon resonance (SPR) assay, a label‐free direct optical biosensor approach, showed a distinct binding of recombinant human (rh) PRDX6 in the presence of apigenin, yielding a predicted dissociation constant (KD) of 0.237 µM (Figure 4K). To identify the residue(s) of PRDX6 responsible for the binding by apigenin, we performed in silico molecular modeling, with the results illustrating that apigenin establishes direct hydrogen bond connections with PRDX6 via the Cys91 and Glu210 of PRDX6 (Figure 4L, Figure S4E, Supporting Information). We then performed microscale thermophoresis (MST) with exogenous PRDX6 protein linked with a GFP tag. Similar to the SPR assay, MST analysis displayed evident interaction between PRDX6 and apigenin with an estimated Kd of 916 nM (Figure 4M,N). Since we speculated Cys91 and Glu210 of PRDX6 may be critical for the binding between target and ligand, we generated constructs that allow to express mutants at these two sites individually or simultaneously. In contrast to wild type PRDX6, mutation of either Cys91 or Glu210 to alanine increased their Kd by approximately tenfold. However, when both sites were mutated, binding between PRDX6 and apigenin was basically abrogated (Figure 4M,N, Figure S4F, Supporting Information). Altogether, our data indicate that the binding of PRDX6 and apigenin may generate a significant effect during cellular senescence.

Previous studies reported that PRDX6 has unique dual‐function enzyme activities within the peroxidase family, with its phospholipase A2 (PLA2) activity causing cells to generate arachidonic acid (AA), a molecule that often induces inflammation.^[^ ^42^ ^]^ As a peroxidase, PRDX6 facilitates the conversion of H_2_O_2_ into water in the presence of NADPH to minimize oxidative damage. Thus, we engineered a construct to overexpress PRDX6 and assessed the capacity of apigenin to remove H_2_O_2_, especially when compared with NAC, a selective inhibitor of peroxidase (Figure S4G,H, Supporting Information). In contrast to NAC, which remarkably reduced the peroxidase activity of purging H_2_O_2_ (IC50 = 15.5 µM), the efficacy of clearing H_2_O_2_ by apigenin was less notably compromised (IC50 = 49.5 µM) (Figure S4I, Supporting Information). The differential consequences generated by PRDX6 and NAC may be attributable to the multiple biological effects caused by NAC, a competent antioxidant that also elevates intracellular Cys level and allows for sulfane sulfur production, stimulating mitochondrial bioenergetics, actions that are beyond its well‐known function of attenuating oxidative stress and protecting cells against oxidative damage, mainly as a ROS scavenger.^[^ ^43^ ^]^

However, upon measurement of PLA2 activity, we noticed a distinct reduction, implying that the capacity of apigenin to dampen the expression of pro‐inflammatory factors may be attributed to its capacity to block the PLA2 activity of PRDX6 (Figure 4O). Moreover, we applied MJ33, a selective PRDX6 PLA2 inhibitor, to examine its efficacy to constrain SASP expression, which is correlated with inflammatory activity of senescent cells. We found that MJ33 treatment lowered expression of the canonical SASP factors in a concentration‐dependent manner (Figure 4P). Proteomics profiling showed that, unlike the vehicle, MJ33 treatment significantly inhibited the PI3K/Akt signaling pathway, further substantiating its senomorphic effect (Figure S4J, Supporting Information). We analyzed the protein levels of human PRDX family, which encompasses six homologs (PRDX1‐6), in senescent cells in the absence or presence of apigenin. We observed neither increased nor decreased expression of these homologs (Figure S4K, Supporting Information). To further dissect the mechanism underlying SASP downregulation by apigenin‐induced suppression of the PLA2 activity of PRDX6, we performed PRDX6‐mediated co‐immunoprecipitation (co‐IP) followed by MS analysis. The results suggested that HSPA8 indeed does interact with PRDX6, suggesting functional interference with either of these molecules may explain the senomorphic effect of apigenin we observed in this study (Figure S4L,M, Supporting Information). Altogether, the data support the possibility that apigenin dampens SASP expression through directly binging to PRDX6 and blocking its PLA2 activity.

Given that PRDX6 is a direct target of apigenin, we queried whether PRDX6 plays an important role in mediating the SASP development. To address this, we chose to deplete PRDX6 from human stromal cells with small hairpin RNAs (shRNAs) (Figure S5A,B, Supporting Information). Upon treatment of cells with BLEO, we observed markedly weakened upregulation of SASP factors (Figure S5C, Supporting Information). Of note, the addition of apigenin to cell culture did not further reduce expression level of the SASP, as evidenced by the case of key SASP factors such as IL6, CXCL8, IL1α, and ILβ, suggesting that apigenin‐caused inhibitory effect of the SASP was mainly through targeting PRDX6.

### 2.5 Apigenin Deprives Cancer Cells of Malignancy Acquired From Senescent Stromal Cells

**Figure 5:**
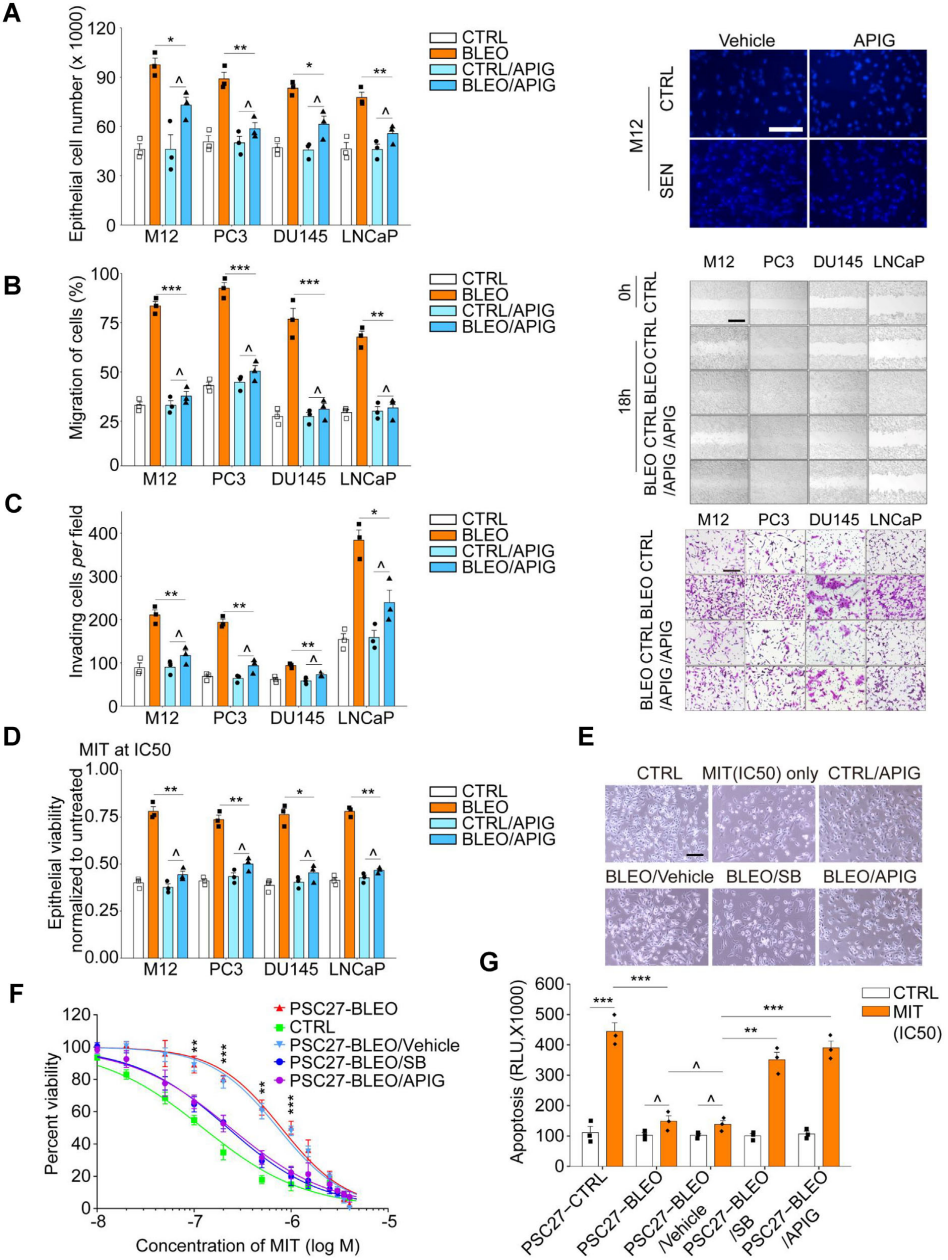
Apigenin reduces PCa cell malignancy conferred by senescent stromal cell‐derived conditioned media. A) Proliferation assay of PCa cells incubated with different types of CM for 3 days. Left, statistics. Right, representative images. Scale bar, 50 µm. B) Migration appraisal of PCa cells incubated with several types of CM for 18 h. Right, representative images. Scale bar, 200 µm. C) Invasiveness measurement of PCa cells across collagen‐based transwell membrance incubated with different types of CM for 3 days. Right, representative images. Scale bar, 200 µm. D) Chemoresistance assay of PCa cells treated with MIT while being cultured with different types of CM. MIT was given at a pre‐determined half‐maximal inhibitory concentration (IC50) for each cell line. E) Examination of cell viability in culture upon exposure to IC50 concentrations of MIT. Representative images are shown. Scale bar, 50 µm. F) Dose‐response curves (nonlinear regression/curve fit) of PC3 cells treated with different types of CM, and concurrently exposed to a range of concentrations of MIT. Data were plotted on an exponential scale, with cell viability assessed relative to the untreated group and calculated as a percentage. G) Signal readings from caspase‐3/7 activity assay of PC3 cells were plotted as relative luminescence units (RLUs). PCa, prostate cancer. CM, conditioned media. MIT, mitoxantrone. SB, SB203580 (a p38MAPK inhibitor that suppresses the SASP expression). Data in A, B, C, D, F and G are shown as mean ± SD and representative of 3 independent biological replicates, with *P* values calculated by Student's *t*‐tests. *P* > 0.05, *P* > 0.05, *P < 0.05, **P < 0.01, ***P < 0.001, ****P < 0.0001. [Correction added on 16 May 2025, after first online publication: Figure 5C has been replaced.]

**Figure 6:**
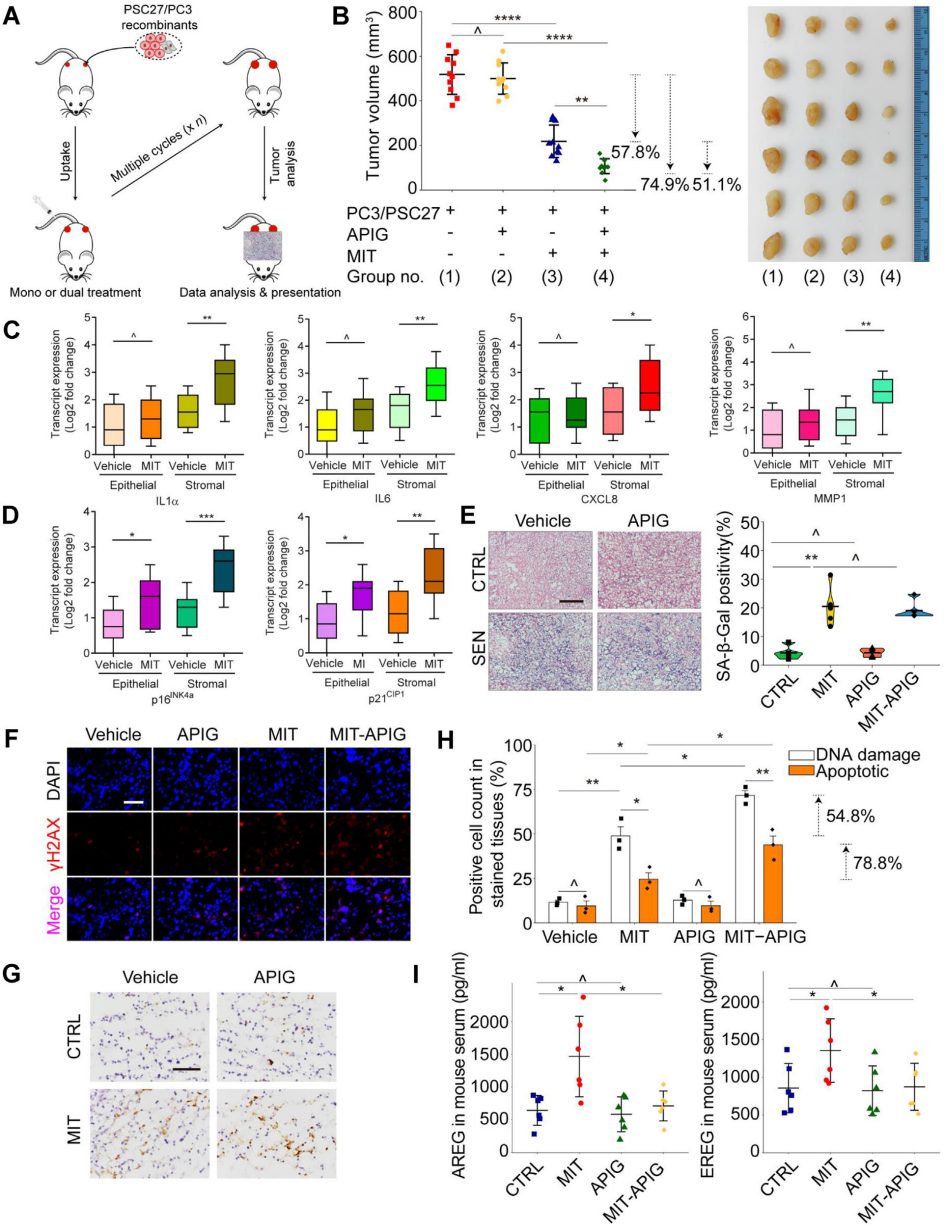
Combination of apigenin and chemotherapy improves preclinical outcomes. A) A schematic illustrating preclinical workflow. Two weeks after subcutaneous inoculation and in vivo uptake of PC3/PSC27 recombinants, severe combined immunodeficient (SCID) mice underwent either single agent or combinatorial treatment in a metronomic schedule composed of several cycles. B) Statistical analysis of tumor end volumes. PC3 cells were inoculated either alone or combined with PSC27 before implanted subcutaneously to the hind flank of NOD/SCID mice, which were administered with MIT and apigenin, alone or sequentially. Left, comparative statistics. Right, representative tumor images. C) Transcriptional analysis of several SASP factors in stromal cells isolated from PC3/PSC27 tumor foci, which were subjected to laser capture microdissection (LCM) for stromal and cancer cell isolation, respectively. Signals were normalized to the lowest sample in placebo group. D) Transcriptional analysis of the two canonical senescence biomarkers p16^INK4a^ and p21^CIP1^. E) Appraisal of cellular senescence in xenograft tissue by SA‐β‐Gal staining. Left, representative images. Right, comparative statistics. Scale bar, 100 µm. F) Quantification of DDR signals by immunofluorescence (IF) staining for γH2AX in xenograft tissues (red, γH2AX, blue, DAPI). Scale bar, 20 µm. G) Assessment of cell apoptosis in xenograft tissues by immunohistochemistry (IHC) staining for caspase cleaved 3 (CCL3) at the completion of treatment regimens. Biopsy samples from animals receiving a placebo served as negative controls for mice receiving treatments by MIT and/or apigenin. Scale bar, 20 µm. H) Statistical evaluation of DNA damage and cell apoptosis in xenograft tissues. Values are shown as the percentage of cells positively stained by IF or IHC specific to γH2AX or CCL3, respectively. I) Evaluation of the circulating levels of two typical SASP factors (AREG, left, EREG, right) in the serum of animals receiving MIT and/or apigenin treatments. DDF, DNA damage foci. Data in B, C, D, E, H and I are shown as mean ± SD and representative of 3 independent biological replicates, with *P* values calculated by Student's *t*‐tests. ^, *P* > 0.05, *P* > 0.05, *P < 0.05, **P < 0.01, ***P < 0.001, ****P < 0.0001.

Many soluble SASP factors secreted by senescent stromal cells favor malignant changes of cancer cells, acting as a significant force that fuels tumor progression as demonstrated by previous studies.^[^ ^26^, ^44^, ^45^, ^46^ ^]^ Herein, we examined the capacity of SASP factor‐containing CM in promoting cancer cell proliferation, a basic feature of activated stroma in the tumor microenvironment (TME). The stromal cell line PSC27 was induced to become senescent by BLEO, a chemotherapeutic agent frequently used in cancer treatment, in the presence or absence of apigenin, with CM collected 8–10 d afterward and used to incubate prostate cancer (PCa) cell lines (PC3, DU145, M12, LNCaP) (**Figure** **5**A). Not surprisingly, proliferative potential was substantially enhanced in all PCa lines after treatment with CM from senescent stromal cells, a tendency consistent with increased migration and invasion of these cells (Figure 5A–C). To exclude potential effects of proliferation on cell migration, we performed wound healing assays during a shorter period, 18 h after cell scratch. Apigenin exhibited a remarkable ability to inhibit cell migration, even though no significant increase in proliferation was observed within such a timeframe. However, the malignant phenotype was markedly attenuated by apigenin treatment (**Figure** **6**A–C).

Our previous studies revealed that genotoxicity‐damaged stromal cells in TME hold remarkable potential to confer resistance on remnant cancer cells, as evidenced by the SASP factors WNT16B, AREG, and EREG.^[^ ^26^, ^45^, ^47^ ^]^ Whether or not the ability of the SASP factors to enhance cancer chemoresistance can be reduced by apigenin remains unknown. We found that the viability of cancer cells was markedly elevated upon co‐culture with CM derived from senescent stromal cells, but diminished almost to the basal level as compared to their normal stromal cell counterparts upon treatment with apigenin (Figure 5D). The capacity of apigenin in counteracting chemoresistance was also in line with the distinct shift of cell survival curves, which was based on the percentage of cell viability across MIT concentrations in the range of 0.1–10 µM, a clinically relevant window of circulating concentrations in cancer patients^[^ ^48^ ^]^ (Figure 5E,F). Such decreased cancer cell viability may be explained by the elevated pro‐apoptotic effect of apigenin on cancer cells via its SASP‐dampening effects, as reflected by the increased activity of caspase 3/7 (Figure 5G). We used docetaxel (DTX), another chemotherapeutic agent, to further confirm the capacity of apigenin in preventing chemoresistance. Replacement of MIT with DTX largely recapitulated the effects of MIT by decreasing apoptosis and increasing chemoresistance, while both were considerably suppressed by apigenin (Figure S6B–D, Supporting Information). These results suggest that senescent stromal cell‐derived CM conferred cancer cells with remarkable therapeutic resistance, a tendency that was attenuated upon apigenin treatment of stromal cells.

Metformin, a widely used antidiabetic drug, is a repurposable agent and can exert multiple beneficial effects including reduction of the SASP without causing substantial cytotoxicity to senescent cells,^[^ ^49^, ^50^ ^]^ thus holding the potential as a senomorphic agent. The results from our metformin‐involving assays generally phenocopied those derived from apigenin assays in lowering cancer cell malignancy by downregulating the activity of stromal cell CM, indicating a common pattern shared by both agents (Figure S6E–H, Supporting Information). Thus, pharmacologically targeting SASP development with a senomorphic agent such as apigenin, can substantially deprive cancer cells of these stromal CM‐conferred gain‐of‐functions, specifically resistance to a chemotherapeutic drug. These findings imply the possibility of developing relevant strategies to improve the efficacy of current anticancer regimens.

### 2.6 Apigenin Combined with Chemotherapy Effectively Reduces Chemoresistance

Given the efficacy of apigenin in attenuating SASP expression in senescent stromal cells and restraining malignant phenotypes of cancer cells in vitro, it is tempting to ask if this agent holds potential for controling senescence‐related pathologies in vivo. To this end, PSC27 sublines were mixed together with PC3, a typically malignant prostate cancer line, at a pre‐optimized ratio (1:, 4) to generate tissue recombinants prior to subcutaneous inoculation to the hind flank of mice with non‐obese diabetes and severe combined immunodeficiency (NOD/SCID). Tumor sizes were determined 8 weeks later for pathological appraisal. In comparison to tumors made up of PC3 and PSC27^Naive^, xenografts comprising PC3 and PSC27^SEN^ were remarkably enlarged. However, treatment of PSC27^SEN^ cells with apigenin prior to tissue recombinant construction substantially reduced tumor volumes (*P* < 0.01). Notably, the efficacy of apigenin was largely reproduced by rapamycin, which exerted a durable in vivo effect even when senescent human cells were treated only once by such a SASP‐inhibiting agent in vitro (Figure S7A, Supporting Information).^[^ ^51^ ^]^

To simulate clinical settings, we designed a preclinical regimen involving genotoxic agents and/or apigenin (Figure 6A, Figure S7B, Supporting Information). Following a 2‐week period after subcutaneous implantation with generally observable tissue recombinant uptake in host animals, a single dose of placebo or MIT was administered at the beginning of the 3rd, 5th, and 7th week, with apigenin given 7 days after each of these administrations, until the end of a 8‐week regimen (Figure S7B, Supporting Information). Although no significant benefits were observed in the apigenin group, MIT administration caused remarkable tumor shrinkage (57.8% reduction in volume), confirming the effectiveness of MIT as a chemotherapeutic agent. When apigenin was administered after MIT, we noticed an additional reduction in tumor size by 51.1%, corresponding to a total decrease of 74.9% compared with the placebo group. We next queried whether cellular senescence occurred in xenografts. As expected, in the TME of PC3/PSC27 recombinant tumors, stromal cells exhibited a markedly elevated expression of canonical SASP factors including IL‐1α/IL‐6, CXCL8, MMP1/MMP3, ANGPTL4 and AREG, a pattern arising in parallel with the upregulation of typical senescence markers p16^INK4a^ and p21^CIP1^ in the MIT group, indicating senescence induction in vivo and SASP expression upon chemotherapeutic treatment (Figure 6C,D, Figure S7C, Supporting Information). Moreover, histologic staining indicated enhanced SA‐β‐Gal positivity in xenografts of mice exposed to MIT, a feature in sharp contrast to apigenin, which neither promoted nor suppressed cellular senescence (Figure 6E), a tendency in line with our results acquired from in vitro assays (Figure 2A). The alterations were predominantly observed in stromal cells instead of adjacent cancer cells, indicating the potential of residual cancer cell repopulation and resistance acquisition in treatment‐damaged TME. However, upon delivery of apigenin, SASP expression was dampened, as evidenced by data from transcript‐based quantitative assays (Figure S7D, Supporting Information).

We next interrogated how the expression of the SASP induces therapeutic resistance of cancer cells. To explore underlying mechanisms, we dissected tissues from animals 7 days after treatment initiation, a time point just prior to the emergence of resistant colonies. In contrast to the placebo group, MIT treatment per se enhanced DDR signaling (as measured by γH2AX) and caused cell death particularly apoptosis (as measured by caspase 3 cleavage (CCL), an apoptosis marker) in cancer cells, when apigenin was combined with MIT, while the administration of apigenin alone triggered neither alteration, implying limited effectiveness of apigenin when used as a single agent for tumor treatment (Figure 6F–H). Upon co‐administration of apigenin and MIT, intensities of both DNA damage and apoptosis were further elevated, indicating increased cytotoxicity upon combinatorial therapy. Upon ELISA analysis of the circulating SASP factors, AREG, and EREG, we found MIT treatment resulted in elevated circulating levels of these factors, a tendency that was essentially reversed upon apigenin administration (Figure 6I).

As an additional point, it remains undetermined whether PRDX6 is functionally involved in regulation of the SASP in cancer cells per se. To this end, we generated PC3 sublines, wherein PRDX6 was depleted by shRNAs (Figure S7E, Supporting Information). The growth of xenografts comprising only PC3 was measured in a way similar to that of tumor composed of both PSC27 and PC3. However, we noticed that apigenin treatment did not confer significant benefits on tumor regression (Figure S7F, Supporting Information), suggesting stromal cell‐related sensitivity to apigenin cannot be simply reproduced by cancer cells. Although there seems to be a trend for PC3 tumors depleted of PRDX6 to be smaller upon apigenin treatment, the changes were not statistically significant (*P* > 0.05). Our datasets suggest that in response to MIT‐mediated chemotherapy, cancer cells also become senescent, but they do not express a typical SASP, as the expression levels of a number of key SASP factors such as IL1α, IL6, CXCL8, and MMP1 remained largely unchanged (Figure 6C,D). Moreover, we found that the DNA damage/apoptosis indices of tumors composed entirely of cancer cells, failed to show significant changes upon apigenin treatment (Figure S7G, Supporting Information). Thus, the tumor‐regression effect conferred by apigenin is not detectable in absence of stromal cells, suggesting a critical role of senescent stromal cells in the development of cancer resistance during chemotherapy.

To establish the safety and effectiveness of our therapeutic strategy, a systematic appraisal of the physiology of experimental animals was performed. Of note, gross assessment data indicated that both single and co‐administration were well tolerated, as evidenced by the absence of body weight loss between different groups during the entire regimen (Figure S8A, Supporting Information). Further, no remarkable fluctuation was observed in the serum level of creatinine, urea, or metabolic status including alkaline phosphatase (ALP) and alanine aminotransferase (ALT) activities, which represent a set of biochemical measurements of the function of major organs such as liver and kidney (Figure S8B, Supporting Information). Hence, co‐administration of a senomorphic agent and canonical chemotherapy holds the potential to achieve maximal anticancer effects while minimizing severe systemic toxicities.

### 2.7 Preclinical Treatment by Apigenin Alleviates Age‐Related Physical Dysfunction and Cognitive Decline

**Figure 7:**
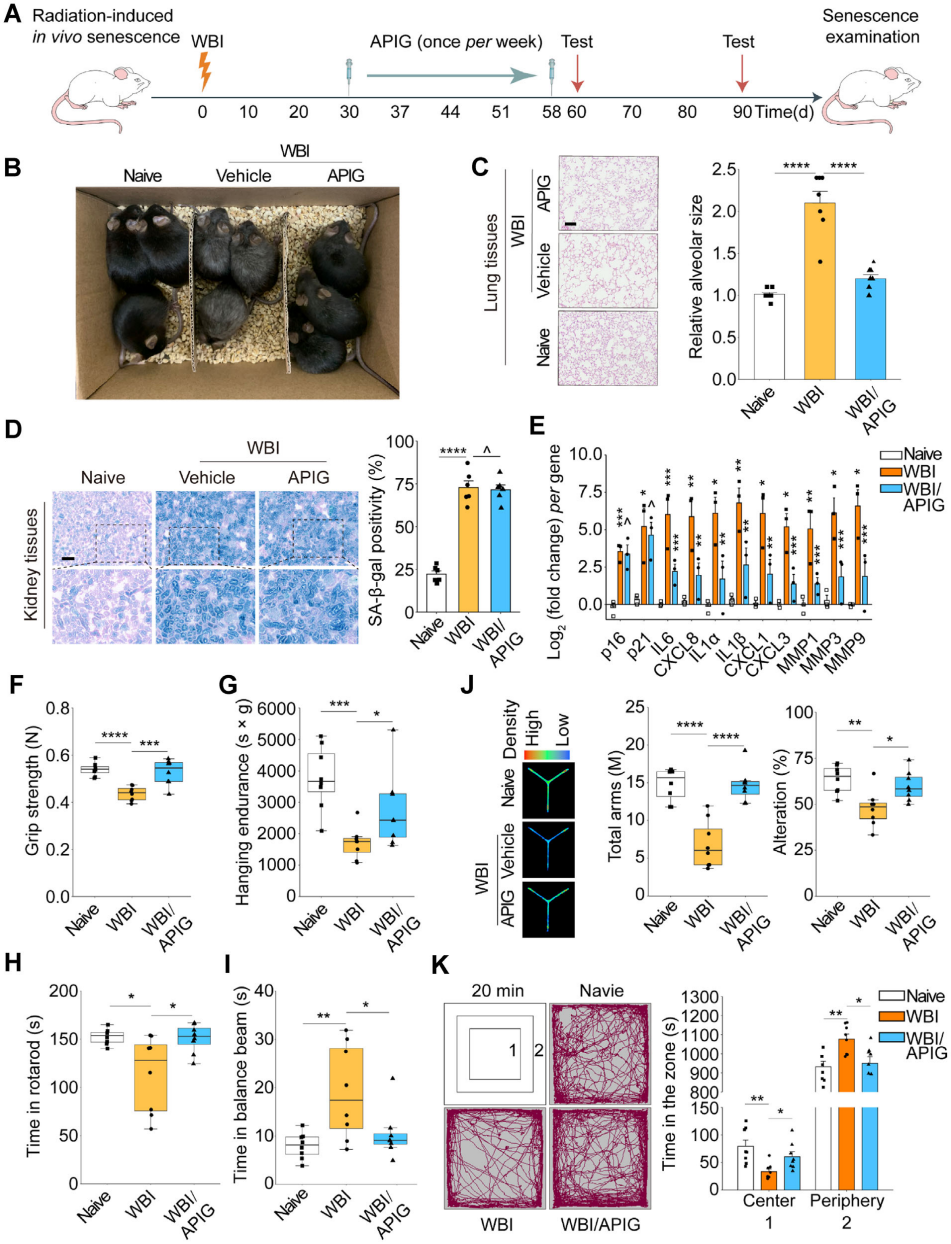
Apigenin administration improves physical function of mice prematurely aged after WBI treatment. A) Schematic illustration of a preclinical procedure for C57BL/6J animals undergoing WBI treatment and subject to physical function tests. B) A representative snapshot of the whole body profile of experimental animals. Naïve mice, WBI mice experiencing vehicle treatment and WBI mice receiving apigenin administration, respectively, were studied. C) H&E staining to dissect the morphology of lung tissues from mice as depicted in (b). Scale bar, 100 µm. D) Representative images of SA‐β‐Gal staining (left) and quantification (right) from kidney tissues from each group. Scale bar, 5 µm. E) Quantitative assessment of the transcript expression of senescence markers (p16^INK4a^ and p21^CIP1^) and several SASP factors in kidney tissues. Signals were normalized to samples of the naïve group per gene. (F–I) Quantitative appraisal of physical functions, including grip strength (F), hanging endurance (G), time in rotarod (H), and time needed for crossing the balance beam (I), all typical physical capacities of animals depicted in (A). n = 8. J) Y maze test of mice. n = 8. K) Evaluation of short‐term memory of animals as depicted in (A) via open field test during a 20 min exploration. “1′ and ‘2′ denote the ‘central” and “peripheral” zones, respectively, of the open field (top left). The representative trajectory (left) and quantification analysis (right) indicate the time animals spent in two zones. n = 8. Each data point represents an individual mouse. n represents the number of animals per group. WBI, whole body irradiation. H&E, hematoxylin and eosin. Data in (C–K) are shown as mean ± SD and representative of 3 independent biological replicates. *P* values were calculated by Student's *t*‐tests. P > 0.05, *P* > 0.05, *P < 0.05, **P < 0.01, ***P < 0.001, ****P < 0.0001.

In the tissue microenvironment, the SASP is a major executor of the paracrine effects of senescent cells and mediates various local and systemic biological effects, including those underlying pathologies and impacting overall healthspan.^[^ ^34^ ^]^ To determine the efficacy of apigenin in regulating senescence‐related in vivo changes including organismal aging, we chose to employ irradiation‐challenged mice for age‐related studies. Briefly, wild type (WT) mice were exposed to a sublethal dose of whole body irradiation (WBI) to induce substantial senescence within tissues, followed by senotherapeutic intervention with apigenin or vehicle, the latter a therapeutic control (**Figure** **7**A). As a result, animals experiencing WBI manifested an aberrant physiological profile, as exemplified by apparently graying fur (Figure 7B). However, this condition was markedly improved upon treatment by apigenin.

Age‐dependent expansion of the pulmonary alveolus, an important tissue structural alteration that contributes to pulmonary dysfunction, was found in WBI‐treated mice, but much attenuated in animals receiving apigenin treatment (Figure 7C). Our data suggest a benefit of apigenin supplementation in preventing the appearance of age‐related pulmonary abnormalities, as changes in the composition of the airways and the alveoli may cause respiratory dysfunction and eventually promote chronic lung disorders during chronological aging.^[^ ^52^ ^]^ Upon histological assessment of spleens of aged mice, we observed atrophy and disarrangement of white pulp, the major immune organ comprising various immune cell subtypes, including DC cells, CD4^+^, CD8^+^ T cells, and CD19^+^ B cells, abnormal changes of which may contribute to age‐associated immune dysfunction (Figure S9A, Supporting Information). However, apigenin exhibited capacity to improve the structural profile of spleen tissues by preventing such abnormalities. Furthermore, the density of spleen‐derived immune cell populations, particularly CD4^+^ T and CD19^+^ B cells, which mainly reside in the periarteriolar lymphocyte sheath (PALS) and the region between PALS and marginal sinuses, respectively,^[^ ^53^ ^]^ markedly decreased in the WBI group, but the change was reversed upon apigenin treatment.

Subsequent analysis indicated the emergence of senescent cells in several major organs, as demonstrated by increased SA‐β‐Gal positivity in kidney and spleen tissues of these animals post WBI‐treatment (Figure 7D, Figure S9B, Supporting Information). As compared with vehicle‐treated mice, the tendency of SA‐β‐Gal staining positivity remained largely unchanged in the apigenin group, suggesting treatment with such a senomorphic agent cannot prevent cellular senescence in these animals. We noted that apigenin failed to downregulate the expression of key senescence markers including p16^INK4a^ and p21^CIP1^ (Figure 7E). The data were further validated by immunofluorescence analysis of p21^CIP1^ expression in the tissue microenvironment (Figure S9C, Supporting Information). However, in contrast, a subset of SASP factors including IL6, CXCL8, IL1α, IL1β, CXCL1/3, and MMP1/3/9, which were significantly upregulated after exposure of mice to WBI, exhibited remarkable decline in animals treated with apigenin, as exemplified by the data from analysis of kidney tissues (Figure 7E). This observation was largely consistent with data acquired from mice involved in chemotherapeutic regimens (Figure S7D, Supporting Information). We examined the biospecimens at protein level. Our data indicated that the upregulation of the canonical SASP marker IL6 in tissues such as the kidney after animals underwent WBI treatment, was partially reversed in animals treated by apigenin (Figure S9D,E, Supporting Information). Upon performance of ELISA assays to measure the circulating levels of AREG and EREG, we observed an apparent elevation of circulating concentration of these SASP factors in irradiation‐challenged animals, a tendency that was counteracted upon apigenin administration (Figure S9F,G, Supporting Information).

Next, we assessed whether preclinical interventions altered the physical function in these animals. Unsurprisingly, mice experiencing WBI manifested reduced exercise performance and muscle strength, as gauged by grip strength, hanging endurance, rotard rod duration, and balance cross tests (Figure 7F–I). However, declines in each of these activities were partially but significantly reversed upon treatment of prematurely aged animals with apigenin, as compared with the vehicle group. We questioned whether apigenin could generally improve health conditions including short term memory and anxiety. Notably, we observed a basic recovery of short‐term memory in aged animals that received apigenin treatment, as evidenced by data from Y‐maze tests (Figure 7J). Data from open field assays indicated that apigenin prolonged duration of exploring the central zone of a wide arena, suggesting anxiety of aged mice was alleviated in aged mice compared with the vehicle group (Figure 7K). The data suggest that apigenin administration represents an effective approach to alleviate physical dysfunction and avoid cognitive impairment during premature aging, promoting overall health.

To establish the safety of our interventional strategy in these immunocompetent animals (C57BL/6J), a systemic and routine evaluation of blood cell counts and serum biochemical indices was performed. As a result, we observed a slight decrease in the counts of both white blood cells (WBCs) and red blood cells (RBCs) in WBI‐treated mice (Figure S9H–J, Supporting Information). Although the difference appeared insignificant, it reflected a partial decline of hematopoietic capacity and immune competency. Upon apigenin administration, these indices remained generally unchanged, validating the safety of apigenin treatment in the hematopoiesis system (Figure S9H‐L, Supporting Information). We found no obvious abnormality in the serum levels of creatinine, urea, and activities of ALP and ALT, liver, and renal biochemical parameters (Figure S9M–P, Supporting Information). Altogether, our preclinical data support that therapeutic regimens involving a senomorphic agent, such as apigenin, in prematurely aged mice, hold potential to alleviate dysfunction without causing systemic cytotoxicity.

## 3 Discussion

Aging affects the functional integrity of various organ types and leads to chronic degenerative pathologies.^[^ ^2^, ^54^ ^]^ The expanding human aging population in most developed countries has generated significant societal and economic costs, making the development of gerotherapeutics an outstanding need. One promising avenue is to explore the potential of natural products, particularly botanical extracts, which are rich in phytochemical constituents with a capacity to serve as health‐promoting agents.^[^ ^55^ ^]^ Increasing lines of evidence suggest that naturally derived compounds targeting cellular senescence hold the potential to improve the healthspan of various mammalian species, specifically rodents, and humans.^[^ ^11^, ^14^, ^15^, ^56^ ^]^ Cellular senescence is a state representing a permanent cell cycle arrest in which cells remain viable, but it contributes to organ degeneration and age‐related diseases when a threshold senescent cell accumulation is exceeded during the course of aging.^[^ ^8^, ^57^ ^]^

The pathogenic roles of senescent cells have been well recognized, with more recent scientific and medical efforts aimed at making them a potentially treatable target.^[^ ^16^, ^58^, ^59^ ^]^ As a critical mediator of senescent cell‐associated pathophysiological effects, the SASP can be effectively targeted through two principal strategies, clearance of senescent cells with senolytics and specific inhibition of the SASP per se using senomorphics, together termed as senotherapeutics.^[^ ^57^, ^60^ ^]^ Promising results have been observed in preclinical studies, and more importantly, in clinical trials, which involve the administration of senolytics for age‐related disorders including diabetic kidney disease (DKD), idiopathic pulmonary fibrosis (IPF), sight‐threatening diabetic macular edema (DME) and early stage Alzheimer's disease (AD).^[^ ^12^, ^13^, ^15^, ^61^ ^]^ In contrast, a major advantage of senomorphics, for example, rutin and resveratrol, is the ability to selectively restrain SASP expression, but without the need to eliminate senescent cell populations in vivo.^[^ ^28^, ^31^ ^]^ This approach, although considered pharmacologically milder than senolytics, can effectively avert the potentially harmful effects of SASP factors, while allowing senescent cells to stay in the tissue microenvironment, which may play essential roles such as impeding tumor development by enhancing immunosurveillance and other physiologically essential activities.^[^ ^62^ ^]^

In this study, we performed in vitro screening of a NMA library to search for candidates of natural senotherapeutics and noticed a small molecule compound apigenin, which exhibits a prominent senomorphic effect. Treatment of senescent cells with apigenin can abrogate the expression of a wide spectrum SASP factors that mediate the occurrence and progression of most, if not all, age‐related pathologies.^[^ ^4^ ^]^ Data from proteome‐wide profiling suggested the possibility that HSPA8 interacts with ATM and p38MAPK, two molecules known to be implicated in the transduction of intracellular signaling to allow SASP development upon cellular senescence.^[^ ^63^, ^64^ ^]^ Further studies indicated that apigenin interferes with crosstalk between ATM and HSPA8, as well as between HSPA8 and p38MAPK, thus blocking more than one signaling mechanism that functionally mediates SASP progression, which usually occurs in a cascading manner in senescent cells. Apigenin abrogates the transition of the ASAP, an acute response of cells after exposure to stress, toward a full scale SASP, a chronic and pro‐inflammatory phenotype. Indeed, such functional efficacy is reminiscent of 5Z‐7‐oxozeaenol, a TAK1 inhibitor, which diminishes SASP expression by restraining the activity of TAK1, a kinase that is involved in the ATM‐TRAF6‐TAK1 axis during the acute DNA damage response and subsequently orchestrates engagement of p38MAPK and PI3K/Akt/mTOR pathways to support persistent SASP signaling.^[^ ^27^ ^]^

**Figure 8:**
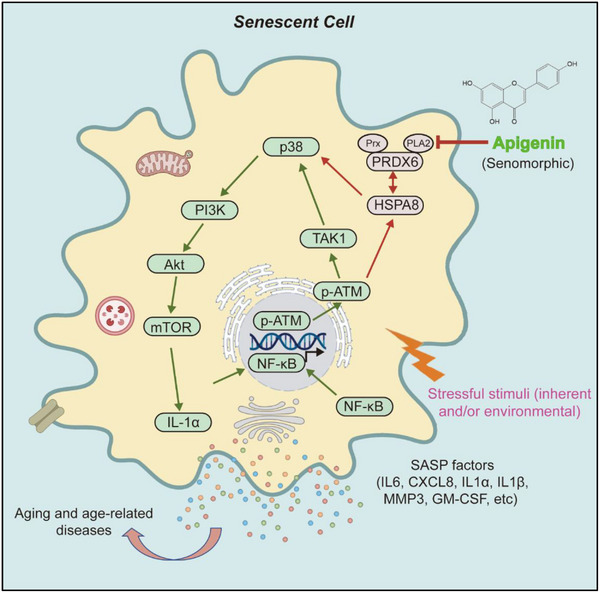
A working model of apigenin as a novel senomorphic agent to target senescent cells and exploration of its potential in future pipelines for treating age‐related conditions. Senescent cells actively synthesize and secrete a number of SASP factors, which encompass soluble proteins including pro‐inflammatory interleukins, chemokines, growth factors, and others. The hypersecretory SASP of senescent cells affects the physiology of neighboring cells and causes chronic inflammation in the tissue microenvironment. As a naturally derived small molecule compound, apigenin directly targets PRDX6 to restrain its PLA2 activity, disrupting HSPA8 activation and interfering with its interactions with ATM and p38MAPK in senescent cells. Through restraining SASP expression, apigenin effectively minimizes the impact of senescent cells on tissue homeostasis, preventing organ dysfunction and alleviating multiple age‐related conditions.

PRDX6 is a unique 1‐Cys member of the peroxiredoxin family, which comprises six highly conserved antioxidant enzymes (PRDX1‐PRDX6) featuring a cysteine (cys) residue involved in peroxide reduction.^[^ ^65^ ^]^ PRDX6 has peroxidase, acidic calcium‐independent phospholipase A2 (aiPLA2), and lysophosphatidylcholine acyltransferase (LPCAT) activities, and is important for the maintenance of lipid peroxidation repair, inflammatory signaling, and antioxidant damage response.^[^ ^66^ ^]^ Although PRDX6 has been extensively investigated in brain disorders such as AD and Parkinson's disease (PD), the functional role of PRDX6 in cellular senescence and its associated phenotypes remains largely unknown. In this study, we first evaluated the effect of apigenin on cellular capacity to scavenge H_2_O_2_. The data suggested that apigenin reduced the ability of cells to clear H_2_O_2_, but in a less effective manner than that of NAC, a selective inhibitor of peroxidase activity, suggesting that the efficacy of apigenin may not rely on its peroxidase activity. Upon alternative dissection, which was conducted on PLA2 activity, we found that the capacity of apigenin to dampen pre‐inflammatory factor expression was indeed correlated with suppressed PLA2 activity of PRDX6. This was further substantiated by assays involving MJ33, a selective PRDX6 PLA2 inhibitor, which remarkably decreased SASP expression in a concentration‐dependent manner. Through a PRDX6‐mediated Co‐IP assay followed by MS profiling, we uncovered an interaction of HSPA8 with PRDX6, two molecules functionally involved in mediating the senomorphic effect of apigenin. Mechanistically, targeting senescent cells by apigenin interferes with PRDX6 PLA2 activity, affecting HSPA8 activation and abrogating HSPA8‐related events including its interactions with both ATM and p38MAPK (**Figure** **8**).

Given the prominent potential of apigenin in dampening SASP expression, we examined the capacity of this compound in restraining the malignancy of cancer cells. Experimental results indicated that almost all the gained functions, namely a series of enhanced malignant phenotypes of PCa cells (a cancer cell model used in this study), were markedly reduced by apigenin. Importantly, augmented chemoresistance of cancer cells conferred by their senescent cell counterparts was largely decreased in the presence of apigenin, implying the possibility of applying apigenin in combinatorial anticancer treatments. We further extended these findings to a mouse model. The data showed that biweekly treatment with apigenin via i.p. significantly inhibited SASP expression in vivo of mice carrying PCa xenografts, resulting in significantly decreased tumor volumes at the end of therapeutic regimens. More importantly, preclinical administration of apigenin to mice prematurely aged by WBI remarkably attenuated age‐related symptoms. Typical geriatric conditions, including fur modification, alveolar volume expansion, muscle strength loss, hanging endurance reduction, and beam balance crossing difficulty, were substantially improved in aged animals receiving administration of apigenin. Interestingly, cognitive impairment, a neurodegenerative deficit observed in these mice, was also prevented by apigenin. Although further studies remain necessary to establish the possible benefits of apigenin in improving other age‐related health conditions, our preclinical evidence suggests a prominent role for apigenin in mitigating physical dysfunction of aged animals, principally by targeting the senescence‐associated inflammatory phenotype, the SASP.

One of the strengths of this study is that we determined the role of apigenin against cellular senescence occurring in cell lines representative of different organ origins as well as upon different forms of senescence induced by alternative stimuli, including the cases of RS, OIS, and TIS. In this study, we mainly focused on TIS, as senescence accumulation within otherwise healthy tissues is an off‐target effect of most chemotherapies.^[^ ^27^, ^67^ ^]^ We used BLEO, a genotoxic agent frequently used for treating cancers, for in vitro senescence induction and phenotypic assessments. We employed MIT, another chemotherapeutic agent that induces senescence by acting as an inhibitor of topoisomerase II to cause DNA damage.^[^ ^68^ ^]^ Administration of MIT to experimental mice triggered significant DDR and cellular senescence in vivo. Apigenin further promoted both DNA damage and cell apoptosis in tumor foci, suggesting that cancer chemoresistance conferred by in vivo senescence can be remarkably weakened by apigenin. To date, most senotherapeutic or anti‐senescence studies chose to use senolytics in design and complementation of preclinical interventions against age‐related pathologies, as removal of senescent cells does result in rejuvenated tissues and organs, a benefit that has been demonstrated by multiple studies including those apply natural agents.^[^ ^11^, ^69^ ^]^ Although it is well established that senescent cell accumulation leads to persistent release of SASP factors and contributes to low‐grade inflammation fueling age‐related degenerative diseases,^[^ ^70^ ^]^ much less effort has been invested to explore the therapeutic potential of senomorphics for long term age‐related conditions.

Apigenin is a flavonoid (4′,5,7‐trihydroxyflavone) and holds substantial promise as a preventative agent against chronic disorders. Holding free radical scavenging capacity, apigenin has neuroprotective, anti‐inflammatory, and antioxidant effects.^[^ ^71^ ^]^ In contrast, its therapeutic potential in targeting senescent cells and minimizing senescence‐related pathological impacts via blockage of SASP expression has remained largely overlooked. In this study, we unmasked a new mechanism by which apigenin can specifically alter the senescence phenotype, curtailing SASP expression. We further demonstrated benefits of apigenin to intervene against age‐related pathologies, opening a new avenue to exploit this a small molecule compound as a novel senomorphic agent. Taken together, our study supports the possibility that apigenin, a plant‐derived flavonoid, can effectively alleviate age‐related disorders, as exemplified by increased anticancer efficacy, improved physical function, and enhanced cognitive behavior of prematurely aged mice. We present a series of proof‐of‐concept data to show that a senomorphic agent can exert significant geroprotective effects at relatively low doses and with a favorable safety profile. This study provides pilot data and a foundation for future research and development of natural compounds as senotherapeutics to prevent, delay or alleviate a number of age‐related pathologies.

## 4 Experimental Section

### Cell Culture

The primary normal human prostate stromal cell line PSC27 was a gift from Dr. Peter Nelson (Fred Hutchinson Cancer Center) and maintained in stromal complete medium as described previously.^[^ ^26^ ^]^ Human fetal lung stromal lines WI38 and IMR90 were from ATCC and cultured with F‐12K medium supplemented with 10% FBS. Prostate cancer epithelial cell lines PC3, DU145, and LNCaP and the human embryonic kidney line 293T were from ATCC and cultured with RPMI 1640 (10% FBS). The prostate cancer epithelial line M12 was kindly provided by Dr. Stephen Plymate (University of Washington), which was originally derived from the benign line BPH1 but phenotypically neoplastic and metastatic.^[^ ^72^ ^]^ All lines were tested for mycoplasma contamination and authenticated with STR assays.

### Cell Treatments

Stromal cells were grown until 80% confluent (CTRL) and treated with 50 µg/mL bleomycin (BLEO). Cells were rinsed briefly with PBS and maintained for 8–10 days prior to performing examinations. For drug screening of potential senolytics, natural candidates (totally 66 in the NMA library) (Selleckchem L8300‐TCM subset) were tested each at 3 µg mL^−1^ on survival of 5.0 × 10^3^ control and senescent cells for 3 d. Subsequent evaluation of effects of these candidate agents (each applied at 1 µg mL^−1^) was conducted to test the extent of SASP inhibition (assay for senomorphics). For those with the potential to act as effective and safe senomorphics, further assessments were done. The phytochemical agent apigenin was examined in the range of 1–10 µM, with 10 µM determined to be the lowest effective concentration for senomorphics activity. The positive control senolytics ABT‐263 (navitoclax) and PCC1 were applied at 1.25 and 50 µM, respectively. The small molecule inhibitor of p38MAPK, SB203580, and the PRDX6‐PLA2 inhibitor MJ33 were employed at 10 µM, to treat senescent cells in culture before collection for lysis and expression analysis.

### Vectors, Viruses, and Infection

Full length human PRDX6 gene sequence was cloned into pHBLV‐U6‐MCS‐CMV‐ZsGreen‐PGK‐Puro (Hanbio Biotechnology). Small hairpin RNAs (shRNAs) targeting PRDX6 (1#, sense strand 5′‐ GATCCGCTCCCAACTTTGAGGCCAATACCATTCAAGAGATGGTATTGGCCTCAAAGTTGGGAGCTTTTTTG‐3′, anti‐sense strand 5′‐ AATTCAAAAAAGCTCCCAACTTTGAGGCCAATACCATCTCTTGAATGGTATTGGCCTCAAAGTTGGGAGCG‐3′, 2#, sense strand 5′‐ GATCCGATTGCCCTTTCAATAGACAGTGTTTTCAAGAGAAACACTGTCTATTGAAAGGGCAATCTTTTTTG‐3′, anti‐sense strand 5′‐ AATTCAAAAAAGATTGCCCTTTCAATAGACAGTGTTTCTCTTGAAAACACTGTCTATTGAAAGGGCAATCG‐3′, scramble, sense strand 5′‐ GATCCGTTCTCCGAACGTGTCACGTAATTCAAGAGATTACGTGACACGTTCGGAGAATTTTTTC‐3′, anti‐sense strand 5′‐ AATTGAAAAAATTCTCCGAACGTGTCACGTAATCTCTTGAATTACGTGACACGTTCGGAGAACG‐3′) were cloned in pLKO.1‐Puro vector (Addgene). Upon production by 293T cells, lentivirual titers were adjusted to infect ≈90% of cells. Stromal cells were infected overnight in the presence of polybrene (8 µg mL^−1^), allowed to recover for 48 h, and selected for 72 h before being used for further analysis.

### Bulk RNA‐seq and Bioinformatics

Total RNA samples were prepared from senescent PSC27 cells cultured with regular DMEM or DMEM containing apigenin (10 µM) for 3 consecutive days. Sample quality was validated by Bioanalyzer 2100 (Agilent), and RNA was subjected to sequencing by Illumina NovaSeq 6000 with gene expression levels quantified by the software package RSEM (https://deweylab.github.io/RSEM/). Briefly, rRNAs in the RNA samples were eliminated using the RiboMinus Eukaryote kit (Qiagen), and strand‐specific RNA‐seq libraries were generated using the TruSeq Stranded Total RNA preparation kit (Illumina, San Diego, CA, USA) according to the manufacturer's instructions before deep sequencing.

Pair‐end transcriptomic reads were mapped to the reference genome (GRCh38.p14) (Genome Reference Consortium Human Build 38, INSDC Assembly GCA_0 00001405.28, 12.2013) (http://asia.ensembl.org/Homo_sapiens/Info/Index) (ensembl_105) with reference annotation from GENCODE v42 using the Bowtie tool. Duplicate reads were identified using Picard tools (1.98) script mark duplicates (https://github.com/broadinstitute/picard), and only non‐duplicate reads were retained. Reference splice junctions are provided by a reference transcriptome (Ensembl build 73).^[^ ^73^ ^]^ FPKM values were calculated using Cufflinks, with differential gene expression called by the Cuffdiff maximum‐likelihood estimate function.^[^ ^74^ ^]^ Genes of significantly changed expression were defined by a false discovery rate (FDR)‐corrected *P* value < 0.05. Only Ensembl genes 73 of status “known” and biotype “coding” were used for downstream analysis.

Reads were trimmed using Trim Galore (v0.6.1) (http://www.bioinformatics.babraham.ac.uk/projects/trim_galore/) and quality assessed using FastQC (v0.10.0) (http://www.bioinformatics.bbsrc.ac.uk/projects/fastqc/). Differentially expressed genes were subsequently analyzed for enrichment of biological themes using the DAVID bioinformatics platform (https://david.ncifcrf.gov/) and the Ingenuity Pathways Analysis program (http://www.ingenuity.com/index.html). Raw data of bulk RNA‐seq were deposited in the NCBI Gene Expression Omnibus (GEO) database under the accession code GSE273159.

Venn diagrams and associated empirical *P*‐values were generated using the USeq (v7.1.2) tool IntersectLists.^[^ ^75^ ^]^ The *t*‐value used was 22008, as the total number of genes of status “known” and biotype “coding” in Ensembl genes 73. The number of iterations used was 1000.

For each gene, the FPKM value was calculated based on aligned reads, using Cufflinks.^[^ ^74^ ^]^ Z‐scores were generated from FPKMs. Hierarchical clustering was performed using the R package heatmap.2 and the distfun = “pearson” and hclustfun = “average”.

### Mapping of Unique Interactors with High‐Throughput Datasets

Profiling of ATM‐ and p38MAPK‐interactive molecules was performed with BioGRID (v4.4.237), a biomedical interaction repository with data compiled through comprehensive curation efforts and used as a public database archiving and disseminating genetic and protein interaction data from all major model organisms and humans (thebiogrid.org).^[^ ^76^, ^77^ ^]^ BioGRID searches 85467 publications for 2800228 protein and genetic interactions, 31144 chemical interactions, and 1128339 post‐translational modifications from major model organism species, with the human selected as the target species throughout this study.

### Cellular thermal shift assay (CETSA)

To assess whether apigenin targets PRDX6 protein, CETSA‐immunoblot experiments were carried out. CETSA is used to examine the binding efficiency of drug and protein. Briefly, PSC27 cells in culture were trypsinized and precipitated by centrifugation, lysed on ice with RIPA buffer containing protease inhibitor cocktail, and centrifuged at 14 000 × g at 4 °C for 20 min. Each lysate was then equally aliquoted into several parts in EP tubes, and incubated with apigenin at room temperature for 1 h. Samples were heated for 3 min under different temperatures (30, 35, 40, 45, and 50 °C). The precipitated proteins were separated from the soluble fraction by centrifugation, and boiled at 95 °C for 10 min. Samples were subsequently used for immunoblot analysis.

### Drug affinity Responsive Target Stabilization (DARTS) Assays

DARTS assays were performed to measure the interaction stability of the small molecule apigenin with its targeting protein(s), and performed according to a previously reported protocol.^[^ ^78^ ^]^ PSC27 cells were lysed with IP lysate buffer on ice with protease and phosphatase inhibitors, before being centrifuged at 4 °C and quantified by the BCA method. Lysates were allowed to rapidly warm up to room temperature and DMSO or apigenin (100 µM) was added to the lysates for 1 h incubation in a rotator. Samples were digested by pronase (1, 300 (w/w), 10 µg mL^−1^) for 30 min at room temperature. Digestion was stopped by 0.5 M EDTA (pH 8.0), with samples boiled in loading buffer for SDS‐PAGE and immunoblot evaluation, or directly subjected to MS analysis for proteomic profiling. The resultant proteomic data of DARTS were deposited to the ProteomeXchange Consortium (http://proteomecentral.proteomexchange.org) through the iProX partner repository with a unique dataset identifier PXD055761.

### Surface Plasmon Resonance (SPR) Assays

The binding affinity of apigenin to recombinant human PRDX6 (rhPRDX6) was determined at 22 °C with a Biacore T200 instrument equipped with CM5 sensor chips (GE Healthcare).^[^ ^79^ ^]^ Briefly, rhPRDX6 protein (SinoBiological) was diluted in sodium acetate solution (pH 4.5) to a final concentration of 20 µg mL^−1^. PRDX6 protein was immobilized on a CM5 sensor chip (GE Healthcare) by amine, allowed to couple with the final immobilization densities of 8000 RU. Immobilized PRDX6 protein was then applied to capture apigenin. The running buffer contained PBS‐P (10 mM sodium phosphate, 150 mM NaCl, 0.05% surfactant P20, pH 7.3) and 5% DMSO. The pH was changed from 7.0 to 5.5 to adjust the binding affinity under several pH environments. Experiments were performed at 25 °C, with a series of concentrations (12.5, 6.25, 3.125, 1.56, and 0.78 µM) of apigenin analyzed at a flow rate of 30 µL min^−1^, with an injection time of 60 s and a dissociation time of 60 s in each binding cycle. A blank immobilization was performed on one channel surface of the chip to adjust the binding response. Association and dissociation constants were acquired using the Biacore T200 Evaluation software (v3.0 GE Healthcare). Data was exported to GraphPad Prism to plot the final curves.

### Pulldown of Apigenin‐Bound Proteins

PSC27 cells were lysed in IP lysate buffer on ice with protease and phosphatase inhibitors, then centrifugated at 4 °C for 10 min, the supernatant was collected and subjected to protein quantification by BCA method. Then, the supernatant was divided into 4 equal parts, wherein 2 of them were pretreated with 10 and 20‐fold concentrations of unlabeled apigenin, respectively, at room temperature for 30 min. Subsequently, upon treatment by biotin or biotin‐apigenin (100 µM) with unlabeled apigenin in the IP buffer overnight at 4 °C. The next day, 30 µL of streptavidin‐agarose beads (Sigma–Aldrich) were added to bind for 2 h at 4 °C. After rinsed thrice with IP buffer, proteins in the beads were eluted, separated by SDS‐PAGE and stained with silver or directly subjected to sample separation for MS analysis.

### Nano‐LC‐MS/MS for Proteomic Analysis

Peptides were resolved in 0.1% formic acid, with the BCA protein quantification kit employed to measure peptide concentration. Samples were separated utilizing an analytic column (75 µm × 15 cm, 3 µm, 100Å) (C‐18, Thermo Scientific) on a nanoflow HPLC Easy‐nLC 1200 system (Thermo Scientific), using a 120 min LC gradient at 300 nL min^−1^. Buffer A comprises 0.1% (v/v) formic acid in H_2_O and Buffer B comprises 0.1% (v/v) formic acid in 80% acetonitrile. The solution gradient was as follows, 1–5% B in 6 min, 5–26% B in 88 min, 26–40% B in 22 min, 40–100% B in 5 min, 90% B in 5 min. MS analyses were conducted on a Q Exactive HF‐X mass spectrometer (Thermo Scientific). MS scan was performed in positive ion mode with spray voltage at 1900 V, with ion transfer tube temperature at 320 °C. Xcalibur software was used to acquire profile spectrum data in a data‐dependent acquisition pattern (DDA). The MS1 full scan was set at a resolution of 60000 @ m/z 200, AGC target 3e6, and maximum IT 50 ms by orbitrap mass analyzer (350‐1800 m/z), followed by ‘top 15′ MS2 scans generated by HCD fragmentation at a resolution of 15000 @ m/z 200, AGC target 1e5 and maximum IT 30 ms. Isolation window was set at 1.6 m/z. The normalized collision energy (NCE) was set at NCE 28%, with the dynamic exclusion time as 45 s. Precursors with charge 1, 7, 8, and > 8 were excluded from MS2 analysis.

### Database Mining of MS Data

All raw mass spectrometric data were analyzed using MaxQuant 2.0.3.0 against the human Swiss‐Prot database comprising 20423 sequences (2023). Carbamidomethyl of cysteine was selected as a fixed modification. Oxidized methionine, protein N‐term acetylation, lysine acetylation, asparagine And glutamine (NQ) deamidation were selected as variable modifications. Enzyme specificity was set as trypsin. The tolerances of first search and main search (Andromeda search engine) for peptides were set at 20 ppm and 6 ppm, respectively. Minimum cutoff for peptide length was set at 7 amino acids, with maximum permissible missed cleavage set at 2. Maximal FDR for peptide spectral match, proteins, and site was set to 0.01. A minimum of 2 sequence‐unique peptides was required for identification. Feature matching between runs was done with a retention time window of 2 min, with the label‐free quantitation (LFQ) function enabled. The MaxQuant peptide and protein quantification results from the “peptides.txt” and“proteinGroups.txt” files were imported into Perseus software (version 1.5.1.6) for further analysis. Statistical significance between the groups was evaluated using Student's *t* tests (p < 0.05), Proteins were defined as differentially expressed if the ratios were ≥ 1.5 or ≤ 0.67 in the experiment group compared with the control.

### In Silico Modeling of Apigenin Binding to PRDX6

The amino acid sequence of PRDX6 (code, P30041) was acquired from UniProt. The structures of apigenin were derived from PubChem (https://pubchem.ncbi.nlm.nih.gov/) and then were energy‐minimized by Molecular Operating Environment (MOE) (v2019.01.02), using MMFF94 force field with the gradient convergence set to 0.1 kcal mol^−1^. The X‐ray diffraction microscopy structure of PRDX6 (PDB ID, 5B6M) was obtained from RCSB Protein Data Bank (PDB, https://www.rcsb.org/). The water molecules farther than 4.5 Å from the receptor were removed. Subsequently, all protein structures were prepared by the built‐in MOE structure preparation and Protonate3D software tools with the default parameters. The binding site of each protein was generated by the SiteFinder module in MOE. The placement method was set to Triangle Matcher with the London dG scoring function, with the refinement score algorithm set to GBVI/WSA dG. Ultimately, the binding mode with the best docking score was selected for the next analysis. The docking score denotes the affinity between the protein receptor and the docking ligand. The lower docking score suggests better affinity.^[^ ^80^ ^]^

### Microscale Thermophoresis (MST)

For confirmation of the binding of apigenin to wild type and mutant PRDX6, PRDX6 constructs with GFP protein or mutant (Cys91 or/and Glu210 to alanine) cloned in pcDNA3.1‐PRDX6‐EGFP‐C2 were emgineered and overexpressed in 293T cells, which were lysed 48 h afterward for MST analysis using Nano Temper Monolith NT Labelfree instrument (Nano Temper Technologies). A series of concentrations of apigenin (200, 100,50, 25, 12.5, 6.25, 3.12, 1.6, 0.8, 0.4 and 0.2 µM) were mixed with PRDX6 protein solution. After incubation at room temperature for 5 min, the mixtures were loaded into MO‐AK002 capillaries. MST assessments were carried out with 20% MST power and 20% LED power with nano‐BLUE pattern. MST datasets were processed using the NT analysis 1.5.41 software (Nano Temper Technologies).

### Peroxiredoxin Activity Evaluation

Peroxiredoxin (Prdx) activity was determined by Hydrogen Peroxide Assay Kit (Beyotime). Briefly, PRDX was overexpressed in PSC27 cells, which were then lysed and quantified by BCA. Cell lysates were incubated with a series of concentrations of apigenin at room temperature for 10 min. H_2_O_2_ was added to a final concentration of 50 µM, with samples incubated for 5 min. H_2_O_2_ assay agents were added, with samples incubated for 30 min at room temperature, the remaining H_2_O_2_ was determined by assessment of the absorbance value at 560 nm. N‐acetylcysteine (NAC) was used as a positive control.

### Phospholipase A2 (PLA2) Activity Assessment

Phospholipase A2 activity was determined according to the manufacturer's instructions (EnzChek Phospholipase A2 kit, Invitrogen). Briefly, PSC27 cells overexpressing PRDX6 were harvested, lysed, and quantified by BCA. For standard curve preparation, PLA2 stock solution (500 U/mL) was diluted with 1 × reaction buffer to make a series of concentrations (0–10 units/mL) of PLA2. For sample measurement, equal amounts of protein were diluted with 1 × PLA2 reaction buffer (1, 1, v/v) up to 50 µL volume, 50 µL of the substrate liposome mix were added to each black microplate for incubation with a total volume of 100 µL for 10 min. The fluorescence of standards, controls, and samples was measured at Ex460 nm/Em515 nm.

### Immunoblot and Immunofluorescence Assays

Whole cell lysates were prepared using RIPA lysis buffer supplemented with protease/phosphatase inhibitor cocktail (Biomake). Nitrocellulose membranes were incubated overnight at 4 °C with primary antibodies, and HRP‐conjugated goat anti‐mouse or ‐rabbit served as secondary antibodies (Vazyme). For immunofluorescence analysis, cells were fixed with 4% formaldehyde and permeabilized before incubation with primary and secondary antibodies, each for 1 h. Upon counterstaining with DAPI (0.5 µg mL^−1^), samples were examined with an Imager A2. Axio (Zeiss) upright microscope to analyze specific gene expression.

### Intracellular Reactive Oxygen Species (ROS) Measurements

Levels of intracellular ROS were determined using a ROS Assay Kit (Beyotime) which employs dichloro‐dihydro‐fluorescein diacetate (DCFH‐DA) as a probe. Briefly, cells were cultured in 6‐well plates for 24 h at 37 °C and were then washed twice with serum‐free medium. Medium containing 10 *µ*M DCFH‐DA was added. Cells were then incubated for 20 min at 37 °C, with light avoided during incubation. After incubation, the cells were washed thrice with serum‐free medium, then observed and photographed using a fluorescence microscope (Nikon). The fluorescence intensity was measured using ImageJ software (v1.51, NIH).

### In Vitro Cell Phenotypic Characterization

For proliferation assays of cancer cells, 2 × 10^4^ cells were dispensed into 6 well‐plates and co‐cultured with stromal cell‐derived conditioned medium (CM). Three days later, cells were digested and counted with hemocytometer. For migration assays, cells were added to the top chambers of transwells (8 µm pore), while stromal CM was added to the bottom. Migrating cells in the bottom chambers were stained by crystal violet 12–24 h later, with samples examined with an Observer A1. Axio (Zeiss) inverted microscope. Invasion assays were performed similarly with migration experiments, except that transwell were coated with basement membrane matrix (phenol red free, Corning). Alternatively, cancer cells were subject to wound healing assays performed in 6‐well plates, with healing patterns graphed with a brightfield microscope. For chemoresistance assays, cancer cells were incubated with stromal CM, with the chemotherapeutic agent MIT (mitoxantrone) provided in wells for 3 days at each cell line's IC50, a value experimentally predetermined. Cell viability was assayed by a CCK‐8 kit, with the absorbance at 450 nm measured using a microplate reader.

### Experimental Animals and Chemotherapeutic Studies

Animals were maintained in a specific pathogen‐free (SPF) facility, with NOD/SCID (Model Animal Research Center of Nanjing University) mice at an age of approximately 6 weeks (≈20 g body weight) used. Ten mice were incorporated in each group, with xenografts subcutaneously generated at the hind flank under isoflurane inhalation. Stromal cells (PSC27) were mixed with cancer cells (PC3) at a ratio of 1: 4 (i.e., 250 000 stromal cells admixed with 1 000 000 cancer cells to make tissue recombinants before implantation in vivo). Animals were sacrificed at 2–8 weeks after tumor implantation, according to tumor burden or experimental requirements. Tumor growth was monitored weekly, with tumor volume (v) measured and calculated according to the tumor length (l), width (w), and height (h) by the formula, v = (π/6) × ((l+w+h)/3)^[^ ^3^, ^81^ ^]^ Freshly dissected tumors were either snap‐frozen or fixed to prepare FFPE samples. Resulting sections were used for IHC staining against specific antigens or subject to hematoxylin/eosin staining.

For chemoresistance studies, animals received subcutaneous implantation of tissue recombinants as described above and were given standard laboratory diets for 2 weeks to allow tumor uptake and growth initiation. Starting from the 3^rd^ week (tumors reaching 4–8 mm in diameter), MIT (0.2 mg kg^−1^ doses) or vehicle controls were administered through intraperitoneal injection (therapeutic agents via i.p. route), on the 1st day of 3rd, 5th and 7th weeks, respectively. Apigenin was delivered 7 days after each MIT or vehicle administration, at doses of 10 mg kg^−1^. Upon completion of the 8‐week therapeutic regimen, animals were sacrificed, with tumor volumes recorded and tissues processed for histological evaluation.

At the end of chemotherapy and/or targeting treatment, animals were anesthetized and peripheral blood was gathered via cardiac puncture. Blood was transferred into a 1.5 ml Eppendorf tube and kept on ice for 45 min, followed by centrifugation at 9000 x g for 10 min at 4 °C. Clear supernatants containing serum were collected and transferred into a sterile 1.5 ml Eppendorf tube. All serum markers were measured using dry‐slide technology on IDEXX VetTest 8008 chemistry analyzer (IDEXX). About 50 µl of the serum sample was loaded on the VetTest pipette tip before securely fit on the pipettor and the manufacturer's instructions were followed for further examination.

All animal experiments were performed in compliance with NIH Guide for the Care and Use of Laboratory Animals (National Academies Press, 2011) and the ARRIVE guidelines, and were approved by the Institutional Animal Care and Use Committee (IACUC) of the Shanghai Institute of Nutrition and Health (no. SINH‐2023‐SY‐1/SINH‐2024‐SY‐1), Chinese Academy of Sciences. Every effort was made to minimize the number of animals used and to reduce potential suffering.

### Tissue SA‐β‐Gal Staining and Histological Examination

For SA‐β‐Gal staining, frozen sections were dried at 37 °C for 20–30 min before being fixed for 15 min at room temperature. The frozen sections were washed thrice with PBS and incubated with SA‐β‐Gal staining reagent (Beyotime) overnight at 37 °C. After completion of SA‐β‐Gal staining, sections were stained with eosin for 1–2 min, rinsed under running water for 1 min, differentiated in 1% acid alcohol for 10–20 s, and washed again under running water for 1 min. Sections were dehydrated in increasing concentrations of alcohol and cleared in xylene. After drying, samples were examined under a bright‐field microscope.

### Histology and immunohistochemistry

Preclinical specimens from mouse tissues were fixed overnight in 10% neutral‐buffered formalin and processed for paraffin embedding. For histological assessment, standard staining with hematoxylin/eosin was performed on sections of 5–8 µm thickness cut from each specimen block. For immunohistochemistry, tumor specimens were first fixed in 4% paraformaldehyde and embedded in paraffin. For appraisal, tissue sections were cut from FFPE chunks, de‐paraffinized, and incubated in 10 mM sodium citrate buffer (pH 6.5) at 95 °C for 40 min for antigen retrieval. Peroxidase activity was quenched with 3% H_2_O_2_ and tissues were blocked in 5% bovine serum albumin for 30 min, before being incubated with primary antibodies (*e.g*., against IL6 or cleaved caspase 3 [CCL3]), for SASP expression appraisal or cell apoptosis evaluation, respectively) overnight at 4 °C. After 3 washes with PBS, tissue sections were incubated with biotinylated secondary antibody (1, 200 dilutions, Vector Laboratories) for 1 h at room temperature then washed thrice, after which streptavidin‐horseradish peroxidase conjugates (Vector Laboratories) were added and the slides incubated for 45 min. DAB solution (Vector Laboratories) was then added and slides were counterstained with hematoxylin. Alternatively, frozen tissue sections were used for immunofluorescence staining to probe target protein expression (e.g., p21 induction) in animals receiving various preclinical treatments, with counterstaining performed with DAPI. Images were then captured with epifluorescence microscope.

### Appraisal of In Vivo Cytotoxicity by Blood Tests

For routine blood examination, 100 µL fresh blood was acquired from each animal and mixed with EDTA immediately. The blood samples were analyzed with Celltac Alpha MEK‐6400 series hematology analyzers (Nihon Kohden). For serum biochemical analyses, blood samples were collected and clotted for 2 h at room temperature or overnight at 4 °C. An aliquot of approximately 50 µl serum was subject to analysis for creatinine, urea, alkaline phosphatase (ALP), and alanine transaminase (ALT) by an VetTest 8008 chemistry analyzer (IDEXX) as reported previously.^[^ ^81^ ^]^ For blood cell tests, 50 µl fresh blood was collected from each mouse and mixed with EDTA immediately. Circulating levels of hemoglobin, white blood cells, lymphocytes, and platelets of the blood samples were analyzed by Sysmex XN‐1000 series automated hematology analyzers (Sysmex, XN‐1000).

All animal experiments were conducted in compliance with the NIH Guide for the Care and Use of Laboratory Animals (National Academies Press, 2011) and the ARRIVE guidelines, and were approved by the IACUC of Shanghai Institute of Nutrition and Health, Chinese Academy of Sciences. For each preclinical regimen, animals were monitored for conditions including hypersensitivity (changes in body temperature, altered breathing, and ruffled fur), body weight, mortality, and changes in behavior (i.e., loss of appetite and distress), and were disposed of appropriately according to the individual pathological severity as defined by relevant guidelines.

### Open Field Testing

The open‐field test was employed to assess the locomotor activity and anxiety of experimental animals when placed in an unfamiliar environment.^[^ ^82^ ^]^ The open‐field chambers (l × w × h, 60 cm × 60 cm × 60 cm) were made of Plexiglas with a non‐reflective square base. Total distance in the whole open‐field chamber, distance traveled in the center zone (30 cm × 30 cm, 50% of the total area) and in the edge zone (20% of the total area, along edges) were recorded. Less activity in the center zone indicated anxiety‐like behaviors.

### Spontaneous Alternation Y‐Maze Test

Spontaneous alternation behavior indicates the tendency for mice to alternate their (conventionally) nonreinforced choices on successive opportunities.^[^ ^83^ ^]^ The Y‐maze spontaneous alternation was employed to assess short‐time spatial recognition and working memory in mice by measurement of spontaneous alternations.^[^ ^84^ ^]^ The maze consisted of three arms, with each arm 31 cm long, 5 cm wide, and 10 cm high. Each arm had markers of different colors as distinct visual cues. After being placed individually at the center of the apparatus, mice were allowed to explore freely through the maze during an 8‐min session. Alternation was defined as successive entries into all three arms on overlapping triplet sets. The number of arm entries and alternations were recorded visually to calculate the percentage of the alternation behavior with the following formula, % Alternation = [Number of Alternations/ (Total number of arm entries ‐2)] x 100. Spontaneous alternation (%), defined as successive entries into the three arms on overlapping triplet sets, is associated with spatial short‐term memory.

### Statistical Analysis

All in vitro experiments were performed in triplicate, while animal studies were conducted with at least eight mice per group except for assays involving PC3‐only xenografts, wherein three mice were used per group. Data are presented as mean ± SD except where otherwise indicated. Sample size (n) for each statistical analysis was indicated wherever possible. Statistical methods used to assess significant differences with sufficient details were provided. GraphPad Prism (9.5.1) was used to collect and analyze data, with statistical significance determined according to individual settings. Cox proportional hazards regression model and multivariate Cox proportional hazards model analyses were performed with SPSS statistical software. Statistical significance was determined by unpaired two‐tailed Student's *t*‐tests, one‐ or two‐way ANOVA, Pearson's correlation coefficients tests, Kruskal‐Wallis, log‐rank tests, Wilcoxon–Mann–Whitney tests, or Fisher's exact tests. When ANOVA indicated significant differences, *post hoc* Tukey's test was applied to compare mean values. For all statistical tests, a *P* value < 0.05 was considered significant. The sample size for each experiment was not predetermined by statistical method, and no data were excluded from analyses.

## Conflict of Interest

The authors declare no conflict of interest.

## Author Contributions

H.Z. and Q.X. contributed equally to this work. Y.S. conceived this study, designed the experiments, and orchestrated the project. H.Z. performed most of the in vitro assays, part of the in vivo experiments, and wrote part of the manuscript. Q.X. and Z.J. helped with cell culture, drug treatment, sample preparation, and proteomic profiling of human senescent cells. R.S. conducted analysis of SASP factor expression in vivo and biochemical parameter tests of animal serum. Q.W., S.L., X.L., J.C., and J.L.K. provided constructive advice and/or supervised a specific subset of experiments. W.Z. provided partial funding support and conceptual input. Y.S. and H.Z. performed data analysis, graphic presentation and finalized the manuscript. All authors critically read and commented on the final manuscript.

## Supporting information



Supporting Information

## Data Availability

The data that support the findings of this study are available from the corresponding author upon reasonable request.

## References

[1] A. Ibanez , M. Maito , F. Botero‐Rodríguez , S. Fittipaldi , C. Coronel , J. Migeot , A. Lacroix , B. Lawlor , C. Duran‐Aniotz , S. Baez , H. Santamaria‐Garcia , Nat. Aging 2024, 4, 1153. 38886210 10.1038/s43587-024-00648-6 PMC11333291

[2] C. Lopez‐Otin , M. A. Blasco , L. Partridge , M. Serrano , G. Kroemer , Cell 2023, 186, 243. 36599349 10.1016/j.cell.2022.11.001

[3] R. L. Cohn , N. S. Gasek , G. A. Kuchel , M. Xu , Trends Cell Biol. 2023, 33, 9. 35599179 10.1016/j.tcb.2022.04.011 PMC9812642

[4] Y. Sun , Q. Li , J. L. Kirkland , Life Med 2022, 1, 103. 36699942 10.1093/lifemedi/lnac030 PMC9869767

[5] V. Lucas , C. Cavadas , C. A. Aveleira , Pharmacol. Rev. 2023, 75, 675. 36732079 10.1124/pharmrev.122.000622

[6] M. DAmbrosio , J. Gil , Dev. Cell 2023, 58, 1007. 37339603 10.1016/j.devcel.2023.05.010

[7] O. Lushchak , M. Schosserer , J. Grillari , Biomolecules 2023, 13, 966. 37371545 10.3390/biom13060966 PMC10296713

[8] S. Chaib , T. Tchkonia , J. L. Kirkland , Nat. Med. 2022, 28, 1556. 35953721 10.1038/s41591-022-01923-y PMC9599677

[9] K. E. Simon , K. Russell , A. Mondino , C. C. Yang , B. C. Case , Z. Anderson , C. Whitley , E. Griffith , M. E. Gruen , N. J. Olby , Sci. Rep. 2024, 14, 12399. 38811634 10.1038/s41598-024-63031-w PMC11137034

[10] M. Xu , T. Pirtskhalava , J. N. Farr , B. M. Weigand , A. K. Palmer , M. M. Weivoda , C. L. Inman , M. B. Ogrodnik , C. M. Hachfeld , D. G. Fraser , J. L. Onken , K. O. Johnson , G. C. Verzosa , L. G. P. Langhi , M. Weigl , N. Giorgadze , N. K. LeBrasseur , J. D. Miller , D. Jurk , R. J. Singh , D. B. Allison , K. Ejima , G. B. Hubbard , Y. Ikeno , H. Cubro , V. D. Garovic , X. Hou , S. J. Weroha , P. D. Robbins , L. J. Niedernhofer , et al., Nat Med. 2018, 24, 1246. 29988130 10.1038/s41591-018-0092-9 PMC6082705

[11] Q. Xu , Q. Fu , Z. Li , H. Liu , Y. Wang , X. Lin , R. He , X. Zhang , Z. Ju , J. Campisi , J. L. Kirkland , Y. Sun , Nat. Metab. 2021, 3, 1706. 34873338 10.1038/s42255-021-00491-8 PMC8688144

[12] J. N. Justice , A. M. Nambiar , T. Tchkonia , N. K. LeBrasseur , R. Pascual , S. K. Hashmi , L. Prata , M. M. Masternak , S. B. Kritchevsky , EBioMedicine 2019, 40, 554. 30616998 10.1016/j.ebiom.2018.12.052 PMC6412088

[13] L. J. Hickson , L. G. P Langhi Prata , S. A. Bobart , T. K. Evans , N. Giorgadze , S. K. Hashmi , S. M. Herrmann , M. D. Jensen , Q. Jia , K. L. Jordan , T. A. Kellogg , S. Khosla , D. M. Koerber , A. B. Lagnado , D. K. Lawson , N. K. LeBrasseur , L. O. Lerman , K. M. McDonald , T. J. McKenzie , J. F. Passos , R. J. Pignolo , T. Pirtskhalava , I. M. Saadiq , K. K. Schaefer , S. C. Textor , S. G. Victorelli , T. L. Volkman , A. Xue , M. A. Wentworth , E. O. Wissler Gerdes , et al., EBioMedicine. 2019, 47, 446. 31542391 10.1016/j.ebiom.2019.08.069 PMC6796530

[14] A. Nambiar , D. Kellogg 3rd, J. Justice , M. Goros , J. Gelfond , R. Pascual , S. Hashmi , M. Masternak , L. Prata , N. LeBrasseur , A. Limper , S. Kritchevsky , N. Musi , T. Tchkonia , J. Kirkland , EBioMedicine. 2023, 90, 104481. 36857968 10.1016/j.ebiom.2023.104481 PMC10006434

[15] M. M. Gonzales , V. R. Garbarino , T. F. Kautz , J. P. Palavicini , M. Lopez‐Cruzan , S. K. Dehkordi , J. J. Mathews , H. Zare , P. Xu , B. Zhang , C. Franklin , M. Habes , S. Craft , R. C. Petersen , T. Tchkonia , J. L. Kirkland , A. Salardini , S. Seshadri , N. Musi , M. E. Orr , Nat. Med. 2023, 29, 2481. 37679434 10.1038/s41591-023-02543-w PMC10875739

[16] S. Song , E. W. Lam , T. Tchkonia , J. L. Kirkland , Y. Sun , Trends Biochem. Sci. 2020, 45, 578. 32531228 10.1016/j.tibs.2020.03.008 PMC7649645

[17] L. R. Bramwell , R. Frankum , L. W. Harries , Cells 2024, 13, 517. 38534362 10.3390/cells13060517 PMC10969307

[18] F. Matteini , S. Montserrat‐Vazquez , F. MC , FEBS Lett. 2024, 598, 2776. 38604982 10.1002/1873-3468.14865 PMC11586596

[19] N. Joma , P. B. Bielawski , A. Saini , A. Kakkar , D. Maysinger , Aging Cell 2024, 23, 14178. 10.1111/acel.14178 PMC11113259 38685568

[20] J. Q. Wang , W. W. Liu , Y. Y. Huang , G. Wang , X. Guo , D. Shi , T. Sun , C. Xiao , C. Zhang , B. Jiang , Y. Guo , J. Li , Adv. Sci. 2024, 11, 2401862. 10.1002/advs.202401862 PMC11423240 39073681

[21] M. Hay , D. W. Thomas , J. L. Craighead , C. Economides , J. Rosenthal , Nat. Biotechnol. 2014, 32, 40. 24406927 10.1038/nbt.2786

[22] N. M. Abdelmaksoud , A. I. Abulsoud , T. M. Abdelghany , S. S. Elshaer , S. M. Rizk , M. A. Senousy , N. W. Maurice , Exp. Cell Res. 2024, 441, 114150. 38971519 10.1016/j.yexcr.2024.114150

[23] X. R. Yao , P. P. Guo , Y. H. Li , H. Guo , Z. Jin , W. Lui , J. Yuan , Q. Gao , L. Wang , Y. Li , J. Shi , X. Zhang , Q. Cao , Y. N. Xu , N. H. Kim/, Theriogenology. 2024, 218, 89. 38308957 10.1016/j.theriogenology.2024.01.007

[24] A. Alghamdi , M. Almuqbil , M. A. Alrofaidi , A. S. Burzangi , A. A. Alshamrani , A. R. Alzahrani , M. Kamal , M. Imran , S. Alshehri , B. A. Mannasaheb , N. F. Alomar , S. M. B. Asdaq , Molecules 2022, 27, 9055. 36558188 10.3390/molecules27249055 PMC9787100

[25] K. M. Perrott , C. D. Wiley , P. Y. Desprez , J. Campisi , Geroscience. 2017, 39, 161. 28378188 10.1007/s11357-017-9970-1 PMC5411372

[26] Y. Sun , J. Campisi , C. Higano , T. M. Beer , P. Porter , I. Coleman , L. True , P. S. Nelson , Nat Med. 2012, 18, 1359. 22863786 10.1038/nm.2890 PMC3677971

[27] B. Zhang , D. Fu , Q. Xu , X. Cong , C. Wu , X. Zhong , Y. Ma , Z. Lv , F. Chen , L. Han , M. Qian , Y. E. Chin , E. W. Lam , P. Chiao , Y. Sun , Nat Commun. 2018, 9, 1723. 29712904 10.1038/s41467-018-04010-4 PMC5928226

[28] H. Liu , Q. Xu , H. Wufuer , Z. Li , R. Sun , Z. Jiang , X. Dou , Q. Fu , J. Campisi , Y. Sun , Aging cell. 2024, 23, 13921. 10.1111/acel.13921 PMC10776113 37475161

[29] J. Chang , Y. Wang , L. Shao , R. M. Laberge , M. Demaria , J. Campisi , K. Janakiraman , N. E. Sharpless , S. Ding , W. Feng , Y. Luo , X. Wang , N. Aykin‐Burns , K. Krager , U. Ponnappan , M. Hauer‐Jensen , A. Meng , D. Zhou , Nat. Med. 2015, 22, 78. 26657143 10.1038/nm.4010 PMC4762215

[30] N. Rachmian , S. Medina , U. Cherqui , H. Akiva , D. Deitch , D. Edilbi , T. Croese , T. M. Salame , J. M. P. Ramos , L. Cahalon , V. Krizhanovsky , M. Schwartz , Nat. Neurosci. 2024, 27, 1116. 38637622 10.1038/s41593-024-01620-8

[31] L. Xia , X. X. Wang , X. S. Hu , X. G. Guo , Y. P. Shang , H. J. Chen , C. L. Zeng , F. R. Zhang , J. Z. Chen , Br. J. Pharmacol. 2009, 155, 387. 10.1038/bjp.2008.272 PMC2567879 18587418

[32] H. Lim , H. Park , H. P. Kim , Biochem. Pharmacol. 2015, 96, 337. 26093063 10.1016/j.bcp.2015.06.013

[33] J. P. Coppé , C. K. Patil , F. Rodier , Y. Sun , D. P. Muñoz , J. Goldstein , P. S. Nelson , P. Y. Desprez , J. Campisi , PLoS Biol. 2008, 6, 2853. 19053174 10.1371/journal.pbio.0060301 PMC2592359

[34] B. Wang , J. Han , J. H. Elisseeff , M. Demaria , Nat. Rev. Mol. Cell Biol. 2024, 25, 958. 38654098 10.1038/s41580-024-00727-x

[35] W. Hui , T. Song , L. Yu , X. Chen , Antioxidants (Basel) 2023, 13, 42. 38247467 10.3390/antiox13010042 PMC10812545

[36] W. Luo , J. Li , Z. Li , T. Lin , L. Zhang , W. Yang , Y. Mai , R. Liu , M. Chen , C. Dai , H. Yang , J. Lu , H. Li , G. Guan , M. Huang , P. Liu , Z. Li , Cell Death Dis. 2021, 12, 738. 34312365 10.1038/s41419-021-04035-6 PMC8313700

[37] D. Moreno‐Blas , E. Gorostieta‐Salas , S. Castro‐Obregon , Ageing Res. Rev. 2018, 41, 34. 29113832 10.1016/j.arr.2017.11.001

[38] L. Wang , H. Dong , G. Song , R. Zhang , J. Pan , J. Han , Cell Mol Immunol. 2018, 15, 685. 28603283 10.1038/cmi.2017.20 PMC6123406

[39] M. Shiota , H. Kusakabe , Y. Izumi , Y. Hikita , T. Nakao , Y. Funae , K. Miura , H. Iwao , Arterioscler Thromb Vasc Biol 2010, 30, 491. 20018937 10.1161/ATVBAHA.109.193631

[40] B. H. R. Lomenick , N. Jonai , Proc. Natl. Acad. Sci. USA 2009, 106, 21984. 19995983 10.1073/pnas.0910040106 PMC2789755

[41] C. X. Liu , Q. Q. Yin , H. C. Zhou , Y. L. Wu , J. X. Pu , L. Xia , W. Liu , X. Huang , T. Jiang , M. X. Wu , L. C. He , Y. X. Zhao , X. L. Wang , W. L. Xiao , H. Z. Chen , Q. Zhao , A. W. Zhou , L. S. Wang , H. D. Sun , G. Q. Chen , Nat. Chem. Biol. 2012, 8, 486. 22484541 10.1038/nchembio.935

[42] J. P. Vázquez‐Medina , J. Q. Tao , P. Patel , R. Bannitz‐Fernandes , C. Dodia , E. M. Sorokina , S. I. Feinstein , S. Chatterjee , A. B. Fisher , Am J. Physiol. Lung Cell Mol. Physiol. 2019, 316, L656. 30702344 10.1152/ajplung.00344.2018 PMC6483013

[43] B. Pedre , U. Barayeu , D. Ezerina , T. P. Dick , Pharmacol. Ther. 2021, 228, 107916. 34171332 10.1016/j.pharmthera.2021.107916

[44] X. Dou , Q. Fu , Q. Long , S. Liu , Y. Zou , D. Fu , Q. Xu , Z. Jiang , X. Ren , G. Zhang , X. Wei , Q. Li , J. Campisi , Y. Zhao , Y. Sun , Nat. Metab. 2023, 5, 1887. 37903887 10.1038/s42255-023-00912-w PMC10663165

[45] Q. Xu , Q. Long , D. Zhu , D. Fu , B. Zhang , L. Han , M. Qian , J. Guo , J. Xu , L. Cao , Y. E. Chin , J. P. Coppé , E. W.‐F. Lam , J. Campisi , Y. Sun , Aging Cell 2019, 18. 10.1111/acel.13027 PMC6826133 31493351

[46] Y. Sun , D. Zhu , F. Chen , M. Qian , H. Wei , W. Chen , J. Xu , Oncogene 2016, 35, 4321. 26751775 10.1038/onc.2015.494 PMC4994019

[47] C. Wang , Q. Long , Q. Fu , Q. Xu , D. Fu , Y. Li , L. Gao , J. Guo , X. Zhang , E. W. Lam , J. Campisi , Y. Sun , Oncogene. 2022, 41, 4941. 36202915 10.1038/s41388-022-02476-7 PMC9630100

[48] V. M. Costa , J. P. Capela , J. R. Sousa , R. P. Eleutério , P. R. S. Rodrigues , J. L. Dores‐Sousa , R. A. Carvalho , M. Lourdes Bastos , J. A. Duarte , F. Remião , M. G. Almeida , K. J. Varner , F. Carvalho , Arch Toxicol. 2020, 94, 4067. 32894303 10.1007/s00204-020-02874-4

[49] Q. Hu , J. Peng , L. Jiang , W. Li , Q. Su , J. Zhang , H. Li , M. Song , B. Cheng , J. Xia , T. Wu , Cell Death Dis. 2020, 11, 925. 33116117 10.1038/s41419-020-03126-0 PMC7595194

[50] J. J. Petrocelli , A. I. McKenzie , N. de Hart , P. T. Reidy , Z. S. Mahmassani , A. R. Keeble , K. L. Kaput , M. P. Wahl , M. T. Rondina , R. L. Marcus , C. K. Welt , W. L. Holland , K. Funai , C. S. Fry , M. J. Drummond , Aging Cell. 2023, 22, 13936. 10.1111/acel.13936 PMC10652302 37486024

[51] R.‐M. L. YS , A. V. Orjalo , Nat. Cell Biol. 2015, 17, 1049. 26147250 10.1038/ncb3195 PMC4691706

[52] J. E. Elliott , C. B. Mantilla , C. M. Pabelick , A. C. Roden , G. C. Sieck , Am J Physiol Lung Cell Mol Physiol 2016, 311, L167. 27288490 10.1152/ajplung.00232.2016 PMC4967189

[53] T. Satoh , H. Oikawa , A. Yashima‐Abo , M. Nishiya , T. Masuda , J. Clin. Exp. Hematop. 2019, 59, 187. 31866620 10.3960/jslrt.19032 PMC6954172

[54] Y. Sun , Q. Li , J. K. Kirkland , Life Med 2022, 1, 103. 36699942 10.1093/lifemedi/lnac030 PMC9869767

[55] B. Pacularu‐Burada , A. I Ciric , M. Begea , Foods 2024, 13, 2441. 39123632 10.3390/foods13152441 PMC11311508

[56] S. Zumerle , M. Sarill , M. Saponaro , M. Colucci , L. Contu , E. Lazzarini , R. Sartori , C. Pezzini , A. Rinaldi , A. Scanu , J. Sgrignani , P. Locatelli , M. Sabbadin , A. Valdata , D. Brina , I. Giacomini , B. Rizzo , A. Pierantoni , S. Sharifi , S. Bressan , C. Altomare , Y. Goshovska , C. Giraudo , R. Luisetto , L. Iaccarino , C. Torcasio , S. Mosole , E. Pasquini , A. Rinaldi , L. Pellegrini , et al., Nat Aging 2024, 4, 1231. 38951692 10.1038/s43587-024-00663-7 PMC11408255

[57] M. Ogrodnik , J. Carlos Acosta , P. D. Adams , F. d'Adda di Fagagna , D. J. Baker , C. L. Bishop , T. Chandra , M. Collado , J. Gil , V. Gorgoulis , F. Gruber , E. Hara , P. Jansen‐Dürr , D. Jurk , S. Khosla , J. L. Kirkland , V. Krizhanovsky , T. Minamino , L. J. Niedernhofer , J. F. Passos , N. A. R. Ring , H. Redl , P. D. Robbins , F. Rodier , K. Scharffetter‐Kochanek , J. M. Sedivy , E. Sikora , K. Witwer , T. von Zglinicki , M. H. Yun , et al., Cell 2024, 187, 4150. 39121846 10.1016/j.cell.2024.05.059 PMC11790242

[58] R. J. Pignolo , J. F. Passos , S. Khosla , T. Tchkonia , J. L. Kirkland , Trends Mol. Med. 2020, 26, 630. 32589933 10.1016/j.molmed.2020.03.005 PMC7857028

[59] S. Song , T. Tchkonia , J. Jiang , J. L. Kirkland , Y. Sun , Adv Sci (Weinh). 2020, 7, 2002611. 33304768 10.1002/advs.202002611 PMC7709980

[60] W. Huang , L. J. Hickson , A. Eirin , J. L. Kirkland , L. LO , Nat. Rev. Nephrol. 2022, 18, 611. 35922662 10.1038/s41581-022-00601-z PMC9362342

[61] S. Crespo‐Garcia , F. Fournier , R. Diaz‐Marin , S. Klier , D. Ragusa , L. Masaki , G. Cagnone , G. Blot , I. Hafiane , A. Dejda , R. Rizk , R. Juneau , M. Buscarlet , S. Chorfi , P. Patel , P. J. Beltran , J. S. Joyal , F. A. Rezende , M. Hata , A. Nguyen , L. Sullivan , J. Damiano , A. M. Wilson , F. A. Mallette , N. E. David , A. Ghosh , P. R. Tsuruda , J. Dananberg , P. Sapieha , Nat Med. 2024, 30, 443. 38321220 10.1038/s41591-024-02802-4

[62] Z. Dong , Y. Luo , Z. Yuan , Y. Tian , T. Jin , F. Xu , Mol. Cancer 2024, 23, 181. 39217404 10.1186/s12943-024-02096-7 PMC11365203

[63] Y. P. Chung , T. I. Weng , D. C. Chan , R. S. Yang , S. H. Liu , Arch. Toxicol. 2023, 97, 547. 36319700 10.1007/s00204-022-03407-x

[64] T. Odawara , S. Yamauchi , H. Ichijo , Commun. Biol. 2024, 7, 691. 38839869 10.1038/s42003-024-06386-0 PMC11153534

[65] H. Fujita , Y. K. Tanaka , S. Ogata , N. Suzuki , S. Kuno , U. Barayeu , T. Akaike , Y. Ogra , K. Iwai , Nat. Struct. Mol. Biol. 2024, 31, 1277. 38867112 10.1038/s41594-024-01329-z PMC11327102

[66] M. Xue , X. Huang , T. Zhu , L. Zhang , H. Yang , Y. Shen , L. Feng , Antioxidants (Basel) 2024, 13, 449. 38671897 10.3390/antiox13040449 PMC11047492

[67] M. Demaria , M. N. O'Leary , J. Chang , L. Shao , S. Liu , F. Alimirah , K. Koenig , C. Le , N. Mitin , A. M. Deal , S. Alston , E. C. Academia , S. Kilmarx , A. Valdovinos , B. Wang , A. de Bruin , B. K. Kennedy , S. Melov , D. Zhou , N. E. Sharpless , H. Muss , J. Campisi , Cancer discovery 2017, 7, 165. 27979832 10.1158/2159-8290.CD-16-0241 PMC5296251

[68] Z. Chen , S. Li , F. Li , C. Qin , X. Li , G. Qing , J. Wang , B. Xia , F. Zhang , L. Meng , X. J. Liang , Y. Xiao , Adv. Sci. (Weinh) 2023, 10, 2206707. 37066748 10.1002/advs.202206707 PMC10238214

[69] Y. Liu , X. Liu , X. Chen , Z. Yang , J. Chen , W. Zhu , Y. Li , Y. Wen , C. Deng , C. Gu , J. Lv , R. Ju , Y. Zhuo , W. Su , Proc. Natl. Acad. Sci. USA 2024, 121, 2311028121. 10.1073/pnas.2311028121 PMC11067450 38657052

[70] S. Khosla , J. N. Farr , T. Tchkonia , J. L. Kirkland , Nat. Rev. Endocrinol. 2020, 16, 263. 32161396 10.1038/s41574-020-0335-y PMC7227781

[71] A. Singh , J. Singh , G. Parween , R. Khator , V. Monga , Crit. Rev. Food Sci. Nutr. 2024, 1. 10.1080/10408398.2024.2390550 39154213

[72] V. L. Bae , J. C. CK , S. J. Maygarden , S. R. Plymate , J. Chen , J. L. Ware , The Prostate. 1998, 34, 275. 9496902 10.1002/(sici)1097-0045(19980301)34:4<275::aid-pros5>3.0.co;2-g

[73] D. R. Zerbino , S. P. Wilder , N. Johnson , T. Juettemann , P. R. Flicek , Genome Biol. 2015, 16, 56. 25887522 10.1186/s13059-015-0621-5 PMC4407537

[74] C. Trapnell , A. Roberts , L. Goff , G. Pertea , D. Kim , D. R. Kelley , H. Pimentel , S. L. Salzberg , J. L. Rinn , L. Pachter , Nat Protoc. 2012, 7, 562. 22383036 10.1038/nprot.2012.016 PMC3334321

[75] D. A. Nix , S. J. Courdy , K. M. Boucher , BMC Bioinformatics. 2008, 9, 523. 19061503 10.1186/1471-2105-9-523 PMC2628906

[76] C. Stark , B. J. Breitkreutz , T. Reguly , L. Boucher , A. Breitkreutz , M. Tyers , Nucleic Acids Res.(Database issue) 2006, 34, D535 10.1093/nar/gkj109 PMC1347471 16381927

[77] R. Oughtred , C. Stark , B. J. Breitkreutz , J. Rust , L. Boucher , C. Chang , N. Kolas , L. O'Donnell , G. Leung , R. McAdam , F. Zhang , S. Dolma , A. Willems , J. Coulombe‐Huntington , A. Chatr‐aryamontri , K. Dolinski , M. Tyers , Nucleic Acids Res. 2019, 47, D529. 30476227 10.1093/nar/gky1079 PMC6324058

[78] M. Y. Pai , B. Lomenick , H. Hwang , R. Schiestl , W. McBride , J. A. Loo , J. Huang , Methods in molecular biology 2015, 1263, 287. 25618353 10.1007/978-1-4939-2269-7_22 PMC4442491

[79] X. Chen , Y. Zhao , W. Luo , S. Chen , F. Lin , X. Zhang , S. Fan , X. Shen , Y. Wang , G. Liang , Theranostics 2020, 10, 10290. 32929349 10.7150/thno.46728 PMC7481428

[80] U. Kalathiya , M. Padariya , M. Baginski , Sci. Rep. 2019, 9, 8707. 31213647 10.1038/s41598-019-45206-y PMC6581908

[81] F. Chen , Q. Long , D. Fu , D. Zhu , Y. Ji , L. Han , B. Zhang , Q. Xu , B. Liu , Y. Li , S. Wu , C. Yang , M. Qian , J. Xu , S. Liu , L. Cao , Y. E. Chin , E. W. Lam , J. P. Coppé , Y. Sun , Nat. Commun. 2018, 9, 4315. 30333494 10.1038/s41467-018-06860-4 PMC6193001

[82] L. A. Reboli , R. M. Maciel , J. C. de Oliveira , M. F. D. Moraes , C. Q. Tilelli , V. R. Cota , Behav. Brain Res. 2022, 426, 113843. 35304185 10.1016/j.bbr.2022.113843

[83] R. N. Hughes , Neurosci. Biobehav. Rev. 2004, 28, 497. 15465137 10.1016/j.neubiorev.2004.06.006

[84] D. Ibi , M. de la Fuente Revenga , N. Kezunovic , C. Muguruza , J. M. Saunders , S. A. Gaitonde , J. L. Moreno , M. K. Ijaz , V. Santosh , A. Kozlenkov , T. Holloway , J. Seto , A. García‐Bea , M. Kurita , G. E. Mosley , Y. Jiang , D. J. Christoffel , L. F. Callado , S. J. Russo , S. Dracheva , J. F. López‐Giménez , Y. Ge , C. R. Escalante , J. J. Meana , S. Akbarian , G. W. Huntley , J. González‐Maeso , Nat. Neurosci. 2017, 20, 1247. 28783139 10.1038/nn.4616 PMC5675106

[85] L. Geng , Z. Liu , W. Zhang , W. Li , Z. Wu , W. Wang , R. Ren , Y. Su , P. Wang , L. Sun , Z. Ju , P. Chan , M. Song , J. Qu , G. H. Liu , Protein Cell. 2019, 10, 417. 30069858 10.1007/s13238-018-0567-y PMC6538594

