# Correction to “Targeting Senescence with Apigenin Improves Chemotherapeutic Efficacy and Ameliorates Age‐Related Conditions in Mice”

**DOI:** 10.1002/advs.202510217

**Published:** 2025-07-14

**[Citation to article being corrected]**

Zhang H, Xu Q, Jiang Z, Sun R, Wang Q, Liu S, Luan X, Campisi J, Kirkland JL, Zhang W, Sun Y. 2025. Targeting Senescence with Apigenin Improves Chemotherapeutic Efficacy and Ameliorates Age‐Related Conditions in Mice. *Adv Sci (Weinh)*. e2412950. https://doi.org/10.1002/advs.202412950.

**[Description of error]**

In the course of data organization and manuscript preparation, there were two pieces of small images mistakenly and inadvertently incorporated into the manuscript and not recognized effectively during the proofing stage. We noticed that the following item needs to be appropriately corrected.

In Figure 5C (right side panel), for representative images of PCa cell invasiveness measurement, the current PC3‐CTRL and M12‐BLEO+APIG images were mistakenly picked up by authors to make the original panel. As a necessary effort, the authors have now corrected the images. Please refer to the updated Figure 5C right side panel.


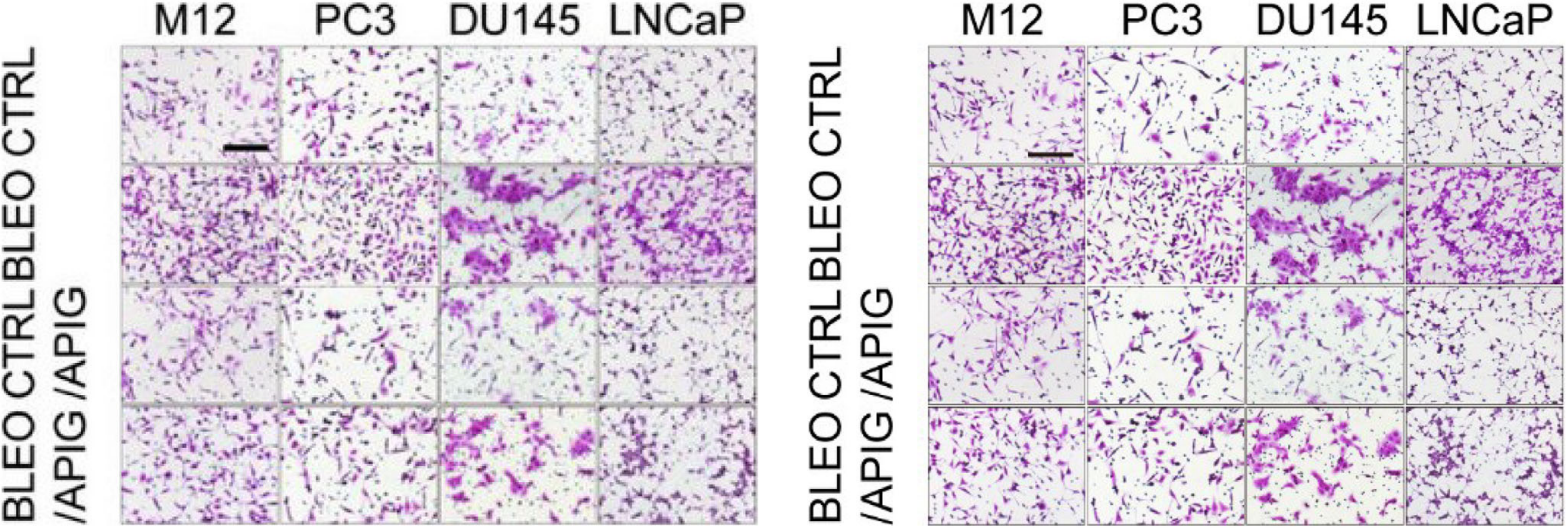


All other parts of this article remain intact, valid and unchanged. We apologize for this error. The corrected figure is provided below.

