# Supplementary material for: Targeting Senescence with Apigenin Improves Chemotherapeutic Efficacy and Ameliorates Age‐Related Conditions in Mice
Source: Adv Sci (Weinh). 2025 Apr 23;12(20):2412950. doi: 10.1002/advs.202412950 (PMC12120719; doi:10.1002/advs.202412950)
Supplement: Supplementary file 1 — Supporting Information [file ADVS-12-2412950-s001.pdf]

ADVANCED SCIENCE Open Access

Supporting Information

for *Adv. Sci.*, DOI 10.1002/advs.202412950

Targeting Senescence with Apigenin Improves Chemotherapeutic Efficacy and Ameliorates Age-Related Conditions in Mice

Hongwei Zhang, Qixia Xu, Zhirui Jiang, Rong Sun, Qun Wang, Sanhong Liu, Xin Luan, Judith Campisi, James L. Kirkland, Weidong Zhang and Yu Sun**

Targeting Senescence with Apigenin Improves Chemotherapeutic Efficacy and Ameliorates Age-Related Conditions in Mice

Hongwei Zhang, Qixia Xu, Zhirui Jiang, Rong Sun, Qun Wang, Sanhong Liu, Xin Luan, Judith Campisi, James L. Kirkland, Weidong Zhang, Yu Sun

Supporting Information

This file includes:

(1) Figures S1-9.

(2) Tables S1-5.

Figure S1

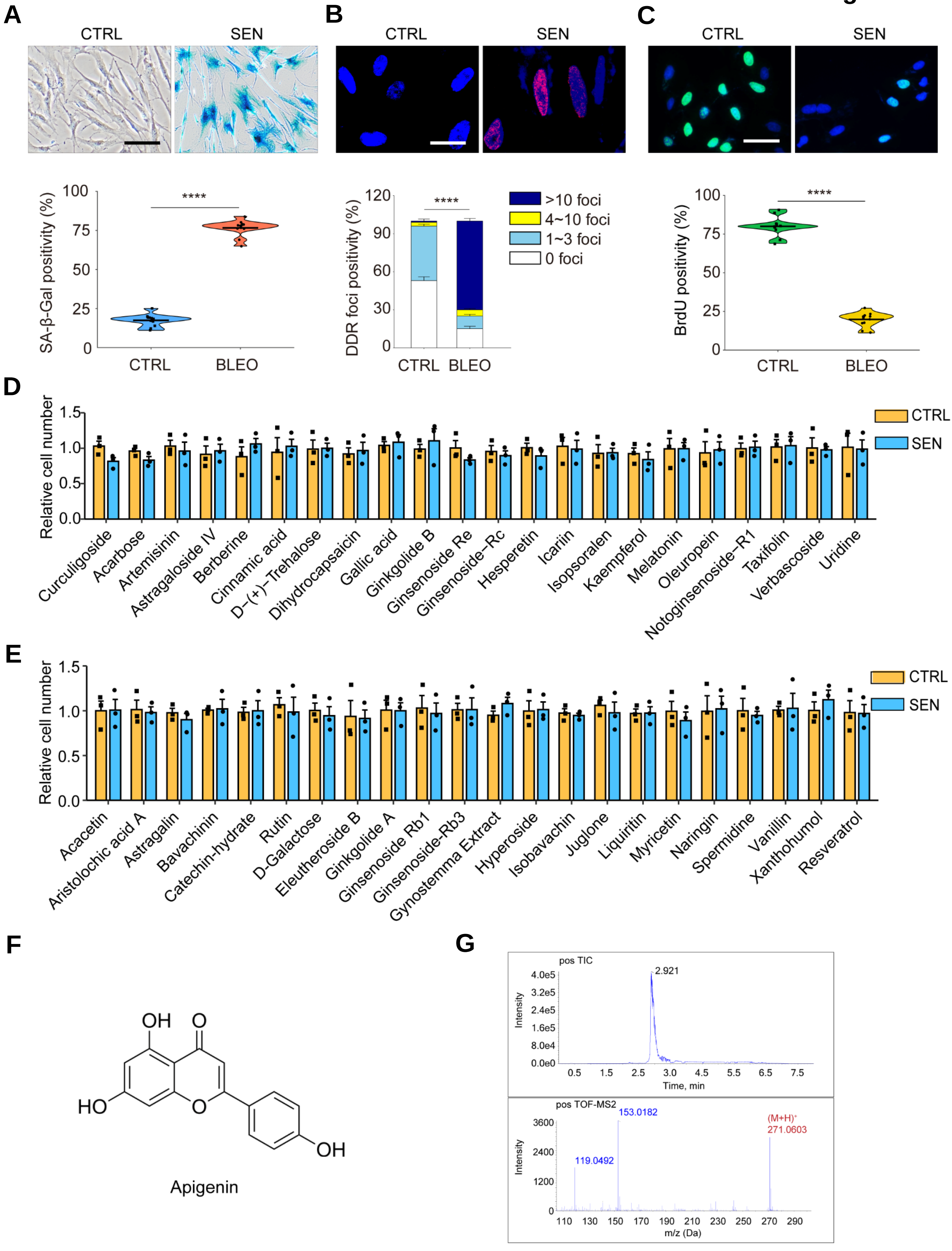
A
CTRL
SEN
SA-β-Gal positivity (%)
****
CTRL
BLEO
B
CTRL
SEN
DDR foci positivity (%)
****
>10 foci
4~10 foci
1~3 foci
0 foci
CTRL
BLEO
C
CTRL
SEN
BrdU positivity (%)
****
CTRL
BLEO
D
Relative cell number
CTRL
SEN
Curculigoside
Acarbose
Artemisinin
Astragaloside IV
Berberine
Cinnamic acid
D-(+)-Trehalose
Dihydrocapsaicin
Gallic acid
Ginkgolide B
Ginsenoside Re
Ginsenoside-Rc
Hesperetin
Icariin
Isopsoralen
Kaempferol
Melatonin
Oleuropein
Notoginsenoside-R1
Taxifolin
Verbascoside
Uridine
E
Relative cell number
CTRL
SEN
Acacetin
Aristolochic acid A
Astragalin
Bavachinin
Catechin-hydrate
Rutin
D-Galactose
Eleutheroside B
Ginkgolide A
Ginsenoside Rb1
Ginsenoside-Rb3
Gynostemma Extract
Hyperoside
Isobavachin
Juglone
Liquiritin
Myricetin
Naringin
Spermidine
Vanillin
Xanthohumol
Resveratrol
F
OH
O
HO
O
OH
Apigenin
G
pos TIC
2.921
Intensity
Time, min
pos TOF-MS2
153.0182
119.0492
(M+H)+
271.0603
Intensity
m/z (Da)

Figure S2

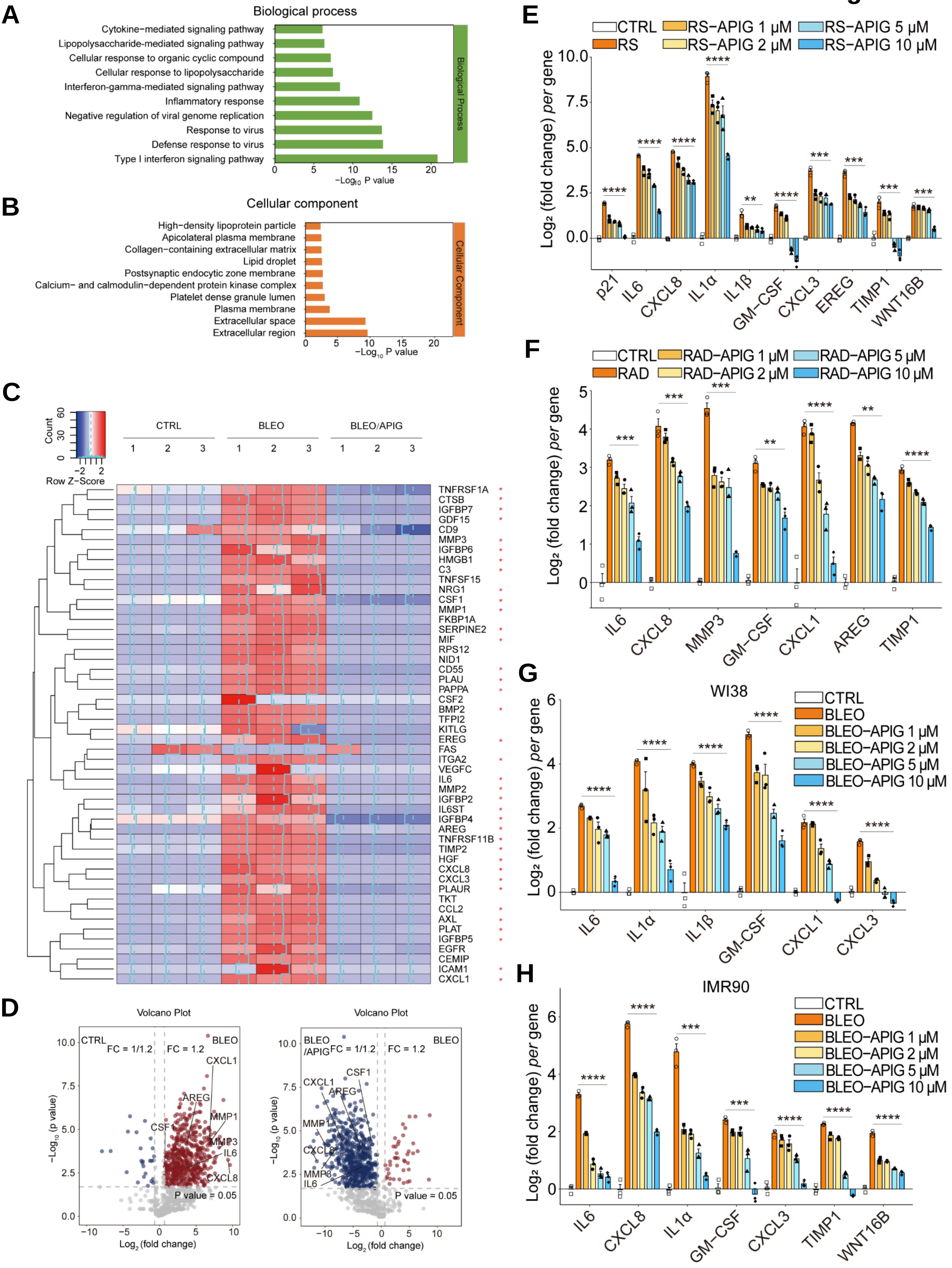

A
Biological process
Cytokine−mediated signaling pathway
Lipopolysaccharide−mediated signaling pathway
Cellular response to organic cyclic compound
Cellular response to lipopolysaccharide
Interferon−gamma−mediated signaling pathway
Inflammatory response
Negative regulation of viral genome replication
Response to virus
Defense response to virus
Type I interferon signaling pathway
Biological Process
−Log10 P value
B
Cellular component
High−density lipoprotein particle
Apicolateral plasma membrane
Collagen−containing extracellular matrix
Lipid droplet
Postsynaptic endocytic zone membrane
Calcium− and calmodulin−dependent protein kinase complex
Platelet dense granule lumen
Plasma membrane
Extracellular space
Extracellular region
Cellular Component
−Log10 P value
C
Count
Row Z-Score
CTRL
BLEO
BLEO/APIG
TNFRSF1A
CTSB
IGFBP7
GDF15
CD9
MMP3
IGFBP6
HMGB1
C3
TNFSF15
NRG1
CSF1
MMP1
FKBP1A
SERPINE2
MIF
RPS12
NID1
CD55
PLAU
PAPPA
CSF2
BMP2
TFPI2
KITLG
EREG
FAS
ITGA2
VEGFC
IL6
MMP2
IGFBP2
IL6ST
IGFBP4
AREG
TNFRSF11B
TIMP2
HGF
CXCL8
CXCL3
PLAUR
TKT
CCL2
AXL
PLAT
IGFBP5
EGFR
CEMIP
ICAM1
CXCL1
D
Volcano Plot
CTRL
BLEO
FC = 1/1.2
FC = 1.2
P value = 0.05
CXCL1
AREG
MMP1
CSF1
MMP3
IL6
CXCL8
Volcano Plot
BLEO /APIG
BLEO
FC = 1/1.2
FC = 1.2
P value = 0.05
CSF1
CXCL1
AREG
MMP1
CXCL8
MMP3
IL6
−Log10 (p value)
Log2 (fold change)
E
CTRL
RS
RS−APIG 1 μM
RS−APIG 2 μM
RS−APIG 5 μM
RS−APIG 10 μM
Log2 (fold change) per gene
p21
IL6
CXCL8
IL1α
IL1β
GM−CSF
CXCL3
EREG
TIMP1
WNT16B
F
CTRL
RAD
RAD−APIG 1 μM
RAD−APIG 2 μM
RAD−APIG 5 μM
RAD−APIG 10 μM
Log2 (fold change) per gene
IL6
CXCL8
MMP3
GM−CSF
CXCL1
AREG
TIMP1
G
WI38
CTRL
BLEO
BLEO−APIG 1 μM
BLEO−APIG 2 μM
BLEO−APIG 5 μM
BLEO−APIG 10 μM
Log2 (fold change) per gene
IL6
IL1α
IL1β
GM−CSF
CXCL1
CXCL3
H
IMR90
CTRL
BLEO
BLEO−APIG 1 μM
BLEO−APIG 2 μM
BLEO−APIG 5 μM
BLEO−APIG 10 μM
Log2 (fold change) per gene
IL6
CXCL8
IL1α
GM−CSF
CXCL3
TIMP1
WNT16B

Figure S3

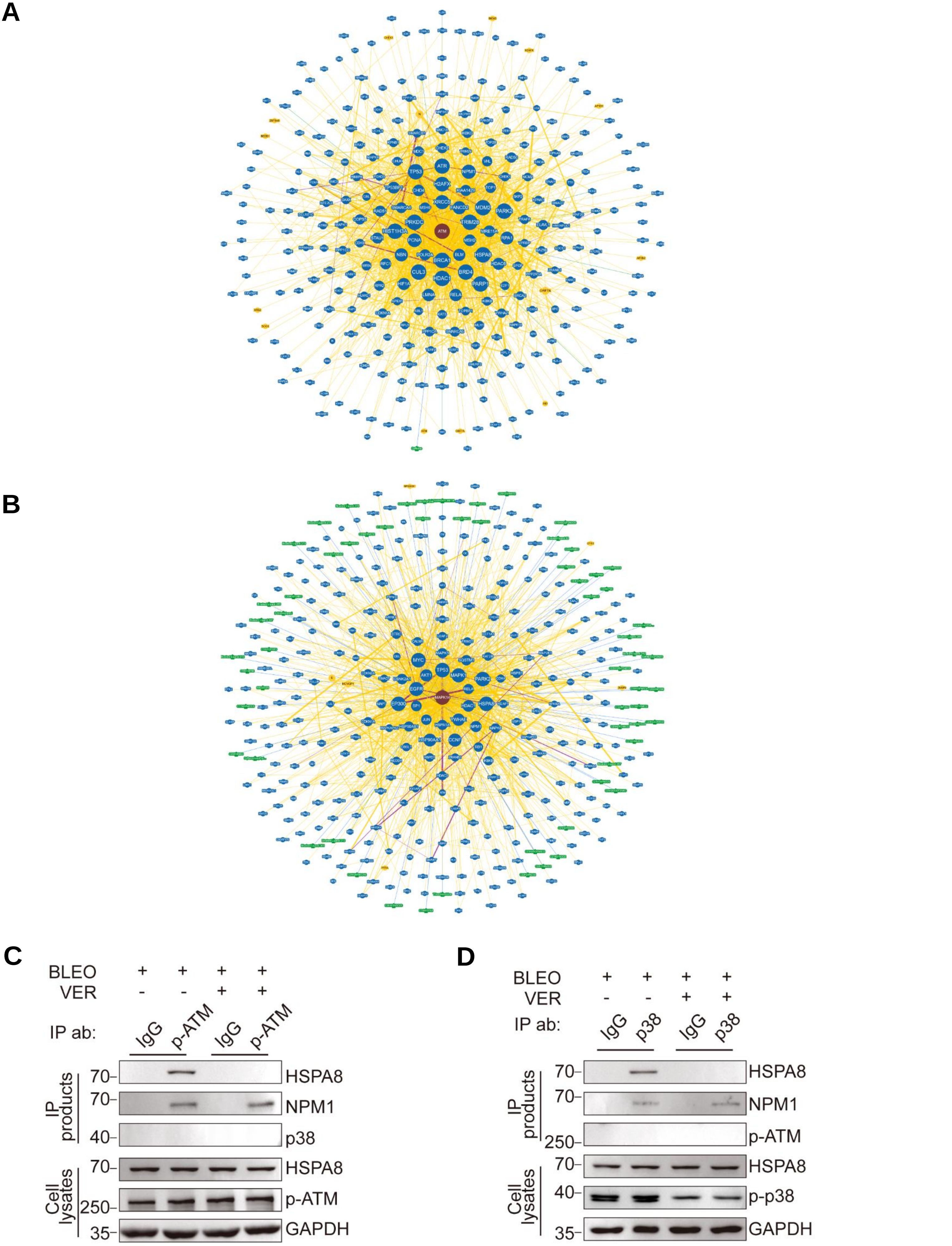
A
B
C
BLEO + + + +
VER - - + +
IP ab: IgG p-ATM IgG p-ATM
IP products
70– HSPA8
70– NPM1
40– p38
Cell lysates
70– HSPA8
250– p-ATM
35– GAPDH
D
BLEO + + + +
VER - - + +
IP ab: IgG p38 IgG p38
IP products
70– HSPA8
70– NPM1
250– p-ATM
Cell lysates
70– HSPA8
40– p-p38
35– GAPDH

Figure S4

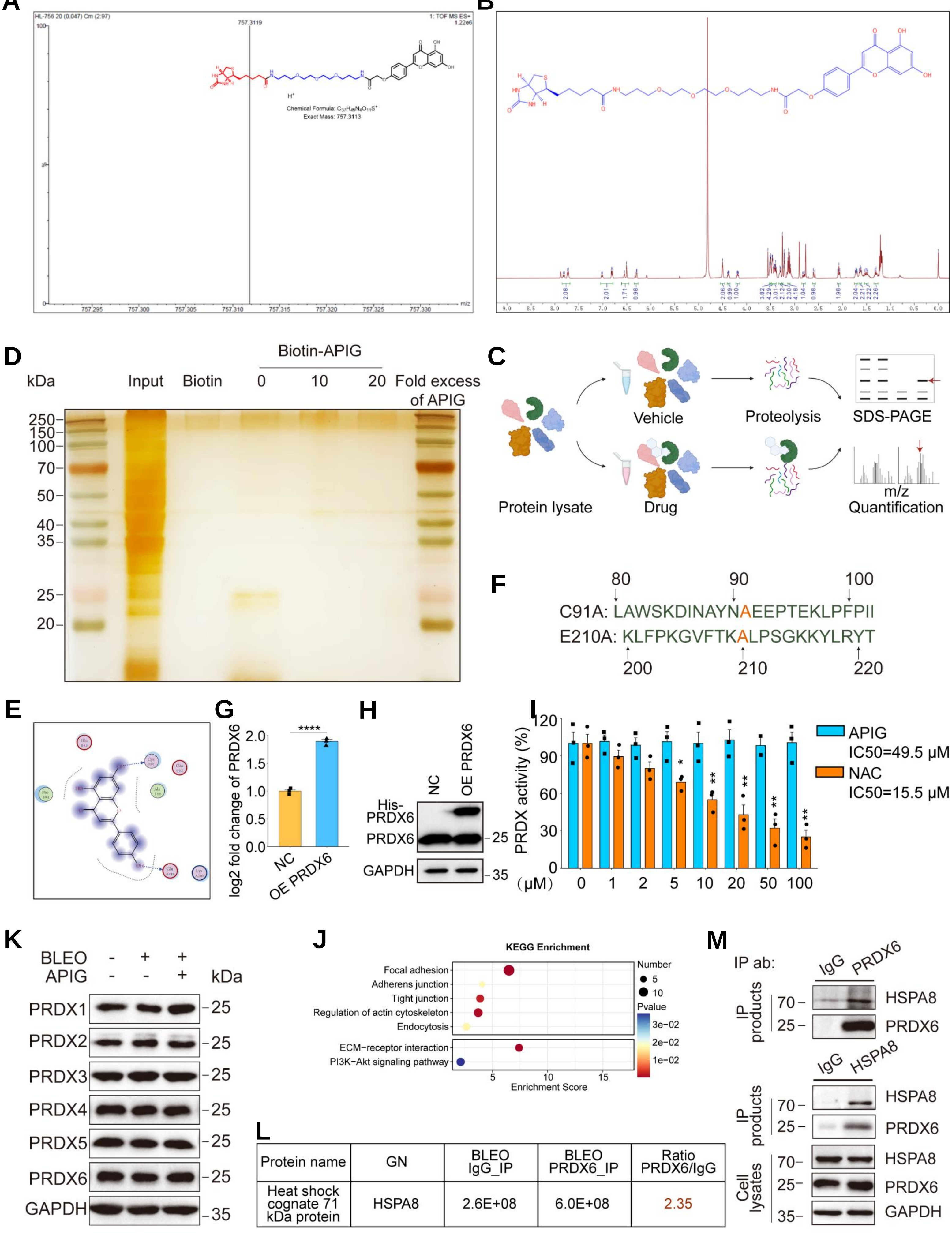

Protein name	GN	BLEO IgG_IP	BLEO PRDX6_IP	Ratio PRDX6/IgG
Heat shock cognate 71 kDa protein	HSPA8	2.6E+08	6.0E+08	2.35

Figure S5

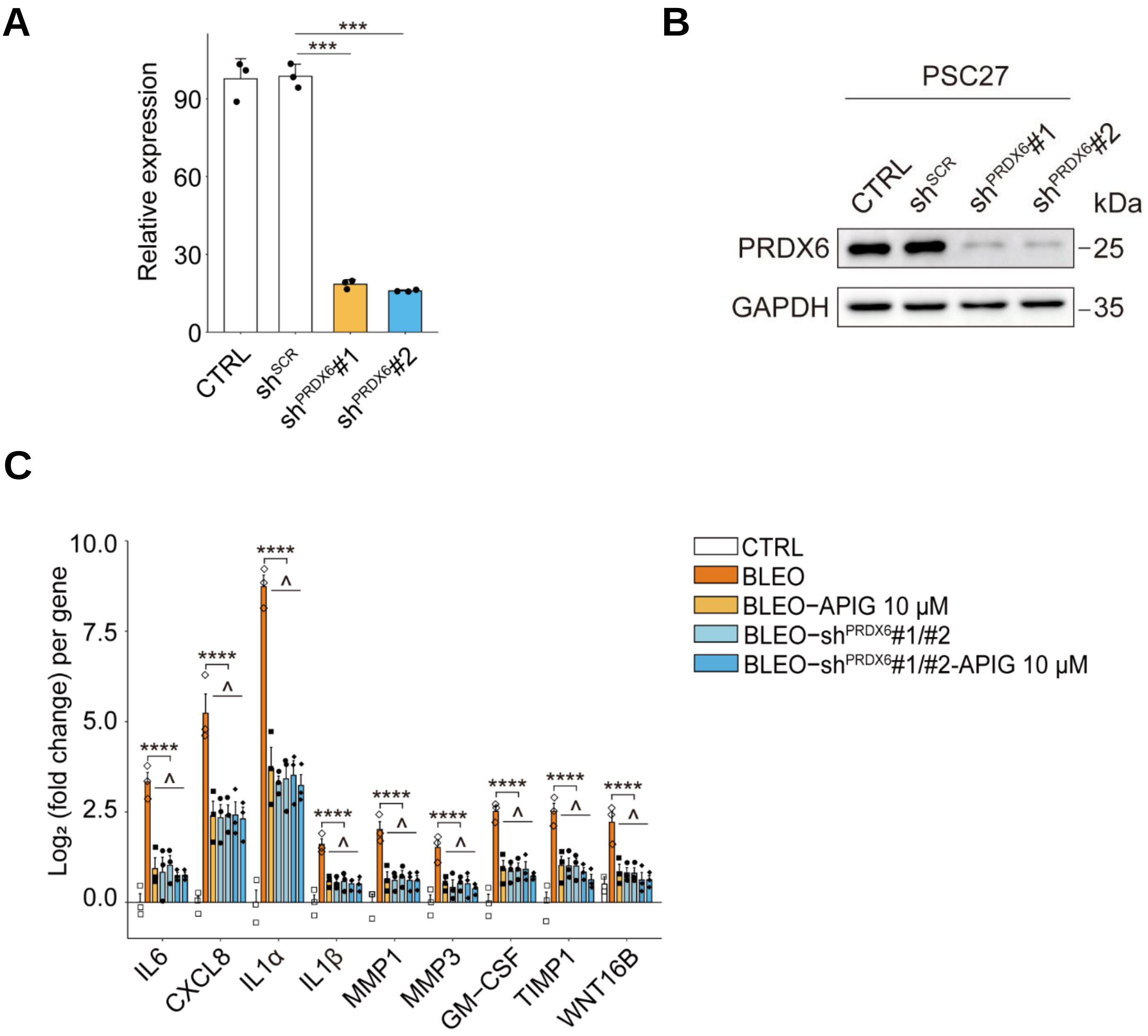

A
Relative expression
0
30
60
90
***
***
CTRL
sh^SCR
sh^PRDX6#1
sh^PRDX6#2
B
PSC27
CTRL
sh^SCR
sh^PRDX6#1
sh^PRDX6#2
kDa
PRDX6
-25
GAPDH
-35
C
Log₂ (fold change) per gene
0.0
2.5
5.0
7.5
10.0
****
^
IL6
CXCL8
IL1α
IL1β
MMP1
MMP3
GM-CSF
TIMP1
WNT16B
CTRL
BLEO
BLEO-APIG 10 μM
BLEO-sh^PRDX6#1/#2
BLEO-sh^PRDX6#1/#2-APIG 10 μM

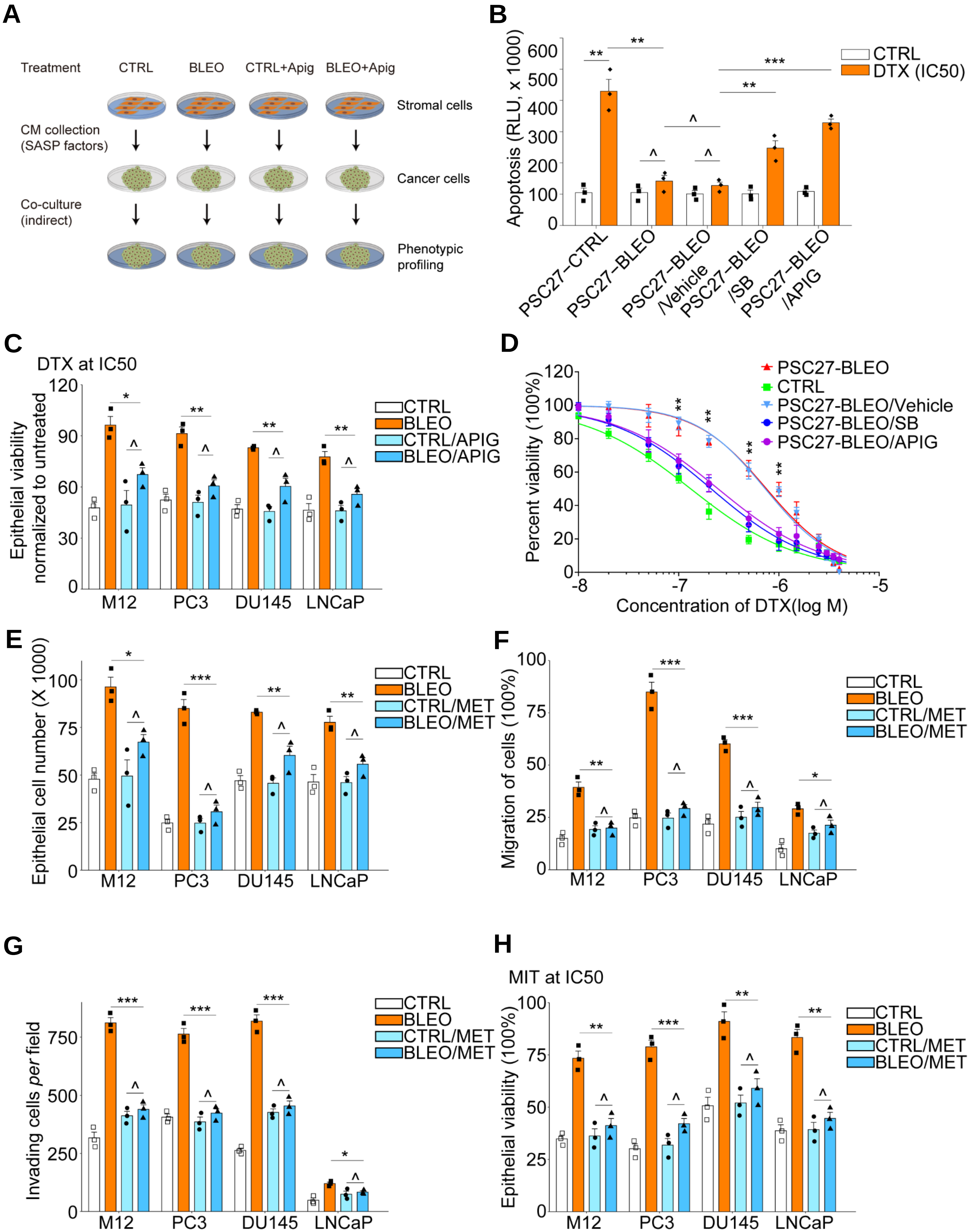
A
Treatment
CTRL
BLEO
CTRL+Apig
BLEO+Apig
Stromal cells
CM collection
(SASP factors)
Cancer cells
Co-culture
(indirect)
Phenotypic
profiling
B
Apoptosis (RLU, x 1000)
CTRL
DTX (IC50)
PSC27-CTRL
PSC27-BLEO
PSC27-BLEO
/Vehicle
PSC27-BLEO
/SB
PSC27-BLEO
/APIG
C
DTX at IC50
Epithelial viability
normalized to untreated
CTRL
BLEO
CTRL/APIG
BLEO/APIG
M12
PC3
DU145
LNCaP
D
Percent viability (100%)
PSC27-BLEO
CTRL
PSC27-BLEO/Vehicle
PSC27-BLEO/SB
PSC27-BLEO/APIG
Concentration of DTX(log M)
E
Epithelial cell number (X 1000)
CTRL
BLEO
CTRL/MET
BLEO/MET
F
Migration of cells (100%)
G
Invading cells per field
H
MIT at IC50
Epithelial viability (100%)

Figure S7

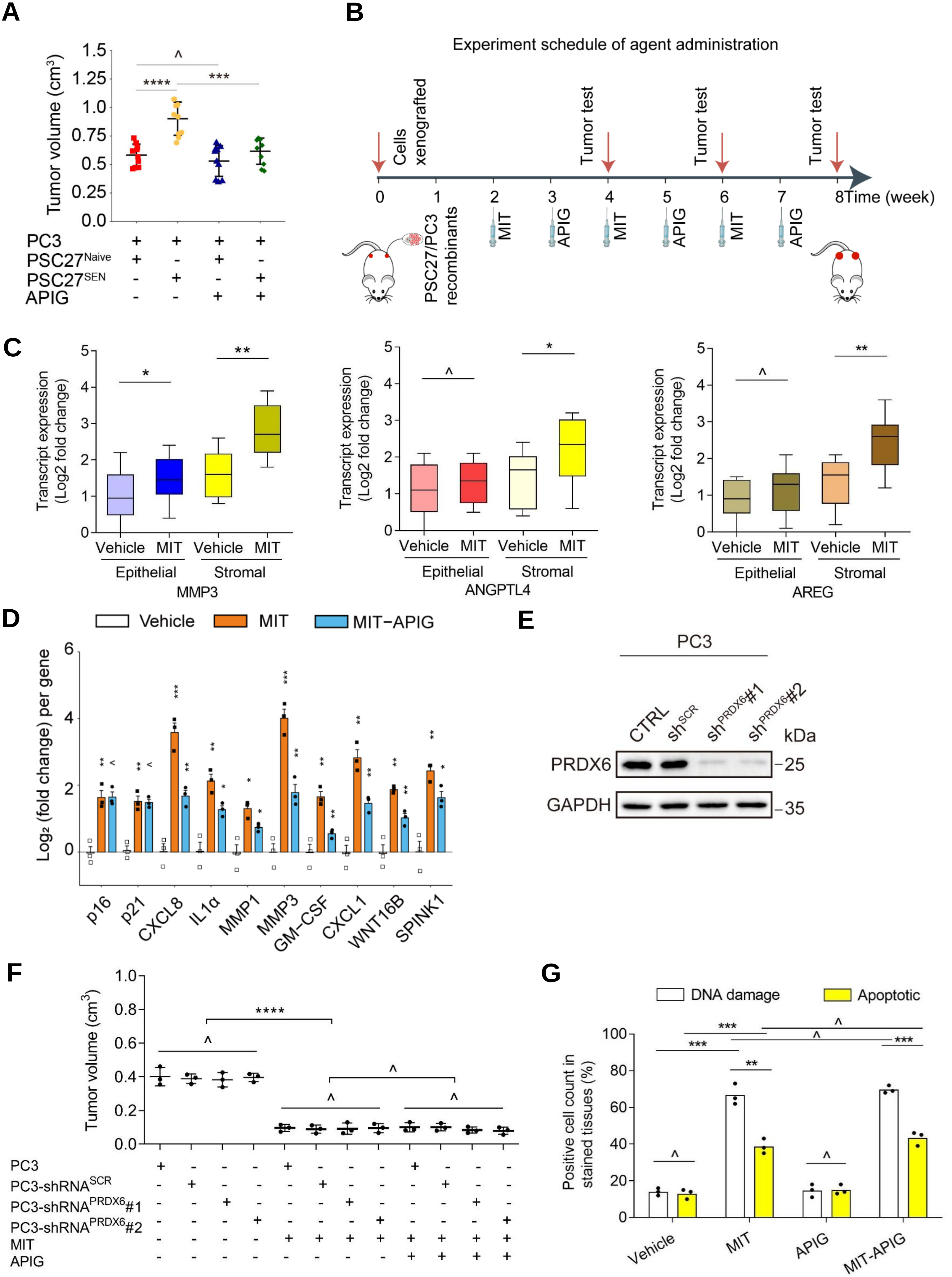

A
Tumor volume (cm³)
PC3
PSC27^Naive
PSC27^SEN
APIG
B
Experiment schedule of agent administration
Cells xenografted
Tumor test
Tumor test
Tumor test
Time (week)
PSC27/PC3 recombinants
MIT
APIG
MIT
APIG
MIT
APIG
C
Transcript expression (Log2 fold change)
Vehicle
MIT
Vehicle
MIT
Epithelial
Stromal
MMP3
ANGPTL4
AREG
D
Vehicle
MIT
MIT−APIG
Log₂ (fold change) per gene
p16
p21
CXCL8
IL1α
MMP1
MMP3
GM-CSF
CXCL1
WNT16B
SPINK1
E
PC3
CTRL
sh^SCR
sh^PRDX6#1
sh^PRDX6#2
kDa
PRDX6
GAPDH
-25
-35
F
Tumor volume (cm³)
PC3
PC3-shRNA^SCR
PC3-shRNA^PRDX6#1
PC3-shRNA^PRDX6#2
MIT
APIG
G
DNA damage
Apoptotic
Positive cell count in stained tissues (%)
Vehicle
MIT
APIG
MIT-APIG

Figure S8

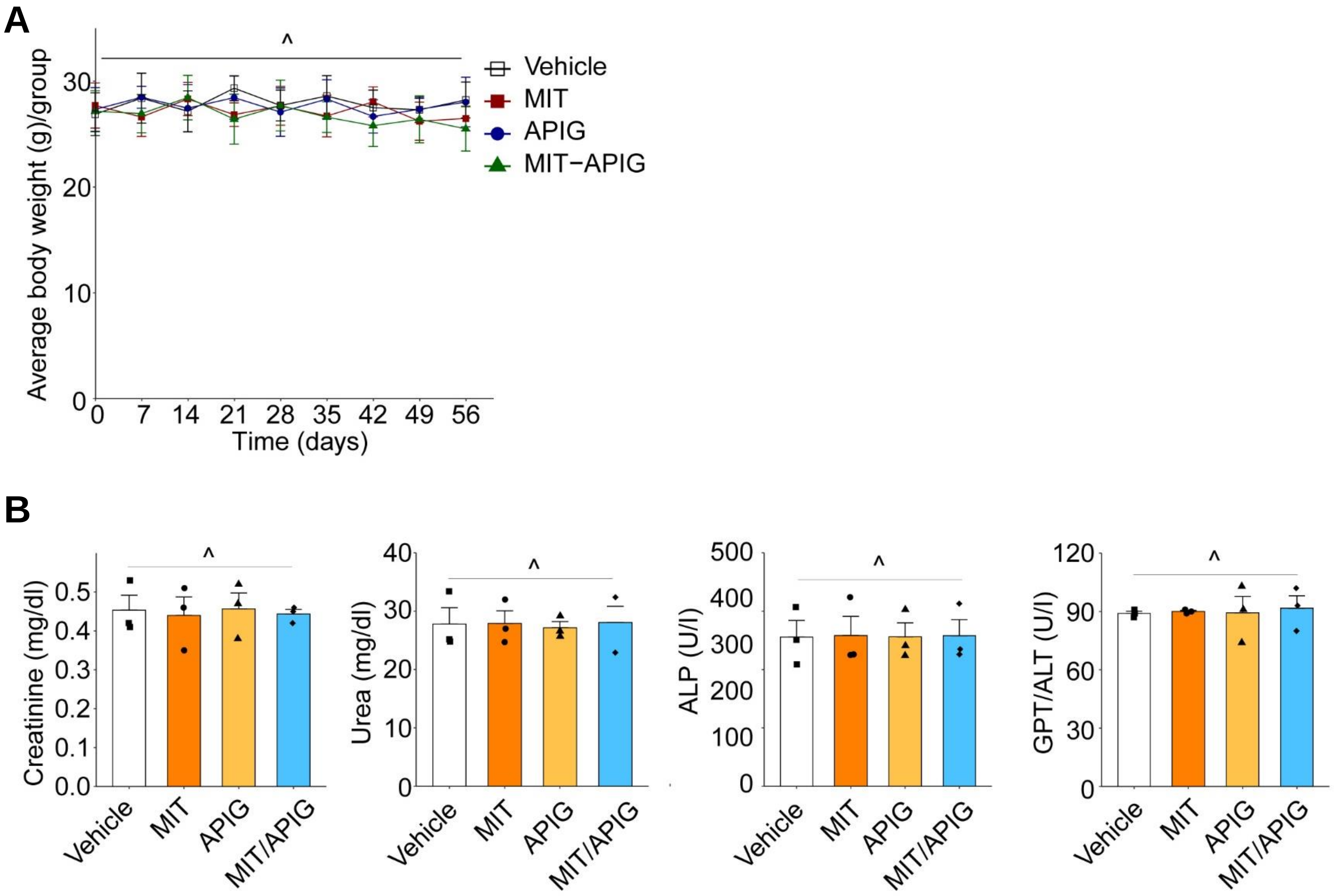
A
Average body weight (g)/group
Time (days)
^
Vehicle
MIT
APIG
MIT−APIG
B
Creatinine (mg/dl)
Urea (mg/dl)
ALP (U/l)
GPT/ALT (U/l)
Vehicle
MIT
APIG
MIT/APIG
^

Figure S9

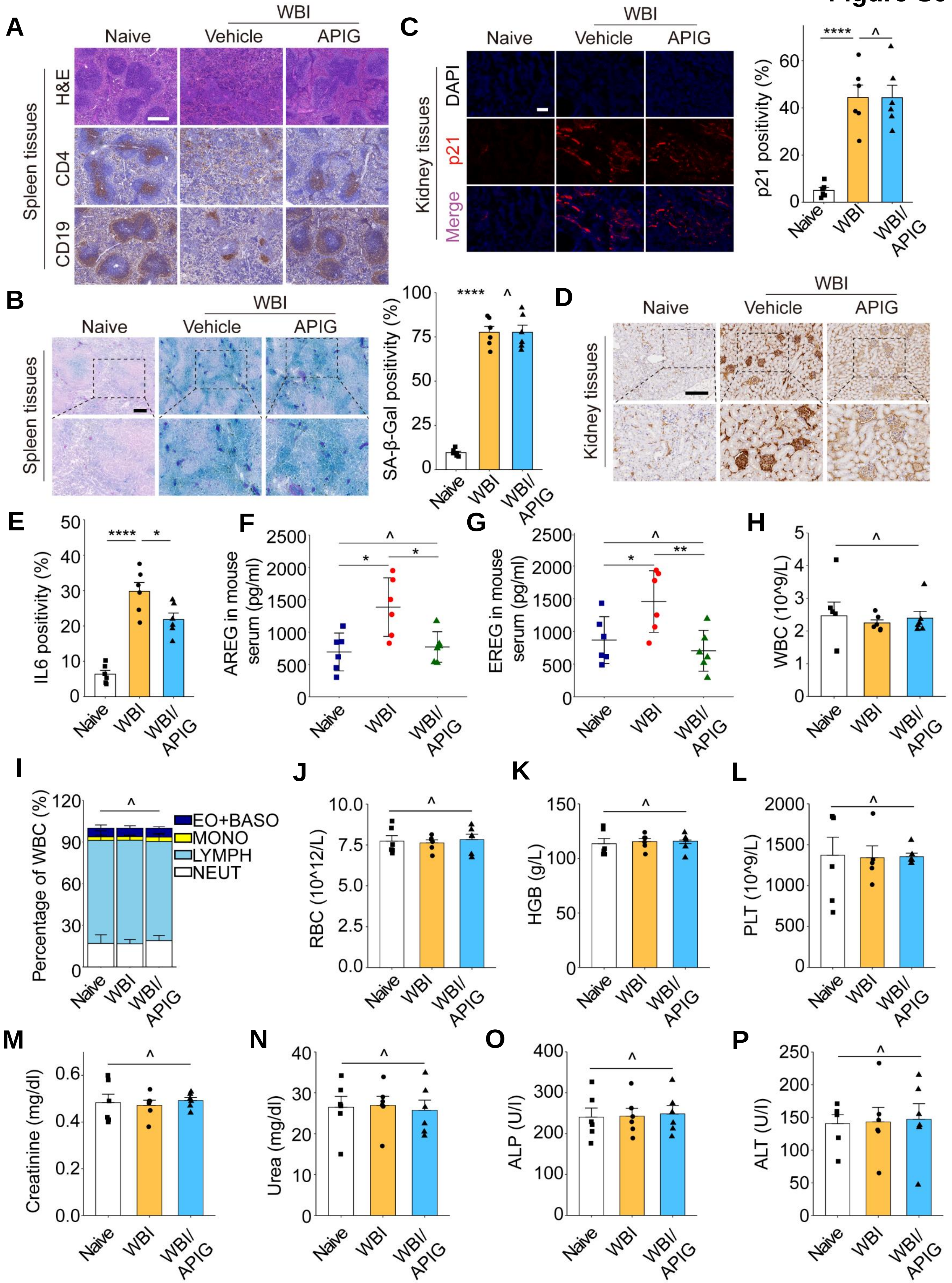
A
Naive
WBI
Vehicle
APIG
Spleen tissues
H&E
CD4
CD19
B
SA-β-Gal positivity (%)
C
Kidney tissues
DAPI
p21
Merge
p21 positivity (%)
D
E
IL6 positivity (%)
F
AREG in mouse serum (pg/ml)
G
EREG in mouse serum (pg/ml)
H
WBC (10^9/L)
I
Percentage of WBC (%)
EO+BASO
MONO
LYMPH
NEUT
J
RBC (10^12/L)
K
HGB (g/L)
L
PLT (10^9/L)
M
Creatinine (mg/dl)
N
Urea (mg/dl)
O
ALP (U/l)
P
ALT (U/l)
WBI/APIG

Figure S1. Phenotypic profiling of TIS, senolytics screening and a potential senomorphic candidate apigenin. (A) Representative images of senescence appraisal by SA-β-Gal staining of PSC27 cells upon occurrence of TIS. Scale bar, 15 μm. Lower, statistics. (B) Representative images of BrdU staining to assess DNA incorporation of PSC27 cells. Scale bar, 5 μm. Lower, statistics. (C) Representative images of DDR by immunofluorescence staining of γh2AX in PSC27 cells. The DDR profile was categorized into 4 sub-groups including 0 foci, 1-3 foci, 4-10 foci and >10 foci *per* cell. Scale bar, 10 μm. Lower, statistics. (D-E) Evaluation of the senolytic potential of remaining natural products (10 μM/agent) in SEN and CTRL cells. (F) Chemical structure of the natural flavonoid apigenin. (G) Mass spectrometry plots displaying base peak chromatogram spectra, precursor ion (MS1) and fragment ion (MS2) of apigenin by performing HPLC-QTOF-MS/MS. Data in a and c are plotted as violin graphs and representative of 10 independent biological replicates with *P* values calculated by Student's *t*-tests. TIS, therapy-induced senescence. DDR, DNA damage response. Data in A, B, C, D and E are shown as mean ± SD and are representative of 3 independent biological replicates with *P* values calculated by two-way ANOVA with Turkey's multiple-comparison tests (B) or Student's *t*-tests (A, C, D, E). ^, $P > 0.05$; *, $P < 0.05$; **, $P < 0.01$; ***, $P < 0.001$; ****, $P < 0.0001$.

Figure S2. Transcriptome- and proteome-wide expression analysis of senescent cells upon apigenin treatment. (A-B) Bar plots showing GO aspects including biological process (BP, A) and cellular component (CC, B) of 67 human genes most significantly restrained by apigenin in senescent PSC27 cells. (C) Heatmap profiling of proteomics in the conditioned media of control cells, senescent cells and senescent cells exposed to apigenin (10 μM) as examined by mass spectrometry. Red stars, SASP factors that were upregulated in senescent cells but significantly downregulated upon apigenin treatment. Note, some proteins are not labelled with red stars, because they are either SASP factors not exhibiting significant changes (between 3 groups) or non-SASP factors *per se*. (D) Volcano plots displaying the differentially expressed SASP factors (red, upregulated; blue,

downregulated) in control cells, senescent cells or senescent cells treated by apigenin. (E) Quantitative appraisal of the expression of canonical SASP factors at transcriptional level upon replicative senescence (RS) at increasing apigenin concentrations. (F) Quantitative evaluation of canonical SASP factor expression at transcriptional level upon ionizing radiation (RAD)-induced senescence with cells exposed to increasing apigenin concentrations. (G) Quantitative analysis of canonical SASP factor expression at transcriptional level in human lung fibroblast cell line WI38 upon BLEO-induced senescence with cells exposed to increasing apigenin concentrations. (H) Quantitative evaluation of canonical SASP factor expression at transcriptional level in human lung fibroblast cell line IMR90 upon BLEO-induced senescence with cells exposed to increasing apigenin concentrations. Unless specially noted, data in E-H are shown as mean ± SD and representative of 3 independent biological replicates with *P* values calculated by Student's *t*-tests. ^, $P > 0.05$; ^, $P > 0.05$; *, $P < 0.05$; **, $P < 0.01$; ***, $P < 0.001$; ****, $P < 0.0001$.

Figure S3. Proteome-wide mapping of potential protein-protein interactions of target molecules. (A) Mapping of the interactive proteins of ATM with BioGRID, a biomedical interaction repository with data compiled through comprehensive curation and serving as an open access database archiving and sharing protein interaction data from model organism species and humans. (B) A BioGRID-based mapping of p38MAPK-interactive molecules, with the biomedical interaction repository as described in (A). (C) Immunoprecipitation (IP) assay coupled with immunoblot analysis to detect protein-protein interactions. PSC27 cells were treated with BLEO (50 μg/mL) for 12 h to induce senescence, in the absence or presence of VER155008 (VER), a HSPA8 inhibitor, in culture for 7 d. Cells were then lysed for IP with IgG or anti-p-ATM, with HSPA8, NPM1, p38 and p-ATM in immunoprecipitates (IPs) and/or inputs examined. (D) IP assay coupled with immunoblot analysis to detect protein-protein interactions. PSC27 cells were treated with BLEO (50 μg/mL) for 12 h to induce senescence, in the absence or presence

of VER in culture for 7 d. Cells were then lysed for IP with IgG or anti-p38, with HSPA8, NPM1, p-ATM, p-p38 in IPs and/or inputs examined. GAPDH, protein loading control.

Figure S4. The interaction of apigenin with PRDX6 results in suppressed iPLA2 activity. (A) High resolution MS of Bio-APIG probe, which connects biotin and hydroxyl of apigenin with bio-inert PEG. (B) ^{1}H NMR spectrum of biotin-apigenin probe (400 MHz, methanol-d4). (C) A schematic illustration introducing the process of DARTS, a label-free drug target discovery approach. PSC27 cell lysates were incubated with apigenin or vehicle for 1 h, followed by proteolysis with pronase for 0.5 h and mass spectrometry (MS) for differential protein quantification. (D) Lysates of senescent PSC27 cells were treated by Bio-APIG or biotin with or without a 10- or 20-fold excess of unlabeled apigenin, before subjected to pulldown by streptavidin-agarose. Proteins captured in beads were determined by SDS-PAGE and subjected to silver staining. (E) Two dimensional *in silico* molecular modelling of apigenin potentially bound to Cys91 and Glu210 of PRDX6 as depicted in Figure 4L. (F) Diagram showing peptide sequences of PRDX6 with two amino acid mutations in the active site. (G-H) HEK293T cells were transfected with a PRDX6 expression construct encoding His-tagged PRDX6 sequence for stable overexpression. (G) Quantitative analysis of PRDX6 expression in cells transfected with the His-tagged PRDX6 construct or vector control. (H) Immunoblot examination of protein expression in cells as described in (G). GAPDH, loading control. (I) Examination of peroxidase activity by measuring the remaining H_2O_2 levels in HEK293T cells stably overexpressing PRDX6 after incubation with gradient concentrations of apigenin or NAC, an strong antioxidant or typical ROS scavenger, for 30 min. (J) KEGG pathway enrichment analysis of significantly downregulated proteins at proteomic level in senescent cells treated with MJ33, a selective PRDX6-iPLA2 inhibitor, as compared with cells treated with vehicle. (K) Immunoblot profiling of the PRDX family (PRDX1-6) at protein level in stromal cells exposed to BLEO and/or apigenin. GAPDH, loading control. (L-M) Immunoprecipitation (IP) coupled

with MS analysis or immunoblot assay to detect protein-protein interactions between PRDX6 and HSPA8. (L) Senescent cells were lysed for IP with IgG or anti-PRDX6, with the enriched beads analyzed by MS thereafter. (M) Senescent cells were lysed for IP with IgG, anti-PRDX6 or anti-HSPA8, with both immunoprecipitates (IPs) and inputs analyzed. MS, mass spectrometry. Bio-APIG, biotin-apigenin. DARTS, drug affinity responsive target stability. Data in G and I are shown as mean ± SD and representative of 3 independent biological replicates, with P values calculated by Student's t-tests. ^, $P > 0.05$; ^, $P > 0.05$; *, $P < 0.05$; **, $P < 0.01$; ***, $P < 0.001$; ****, $P < 0.0001$.

Figure S5. Knockdown of PRDX6 in human stromal cells and the impact of PRDX6 depletion on SASP expression. (A) Quantitative measurement of PRDX6 expression at transcription level in PSC27 cells upon establishment of sublines that underwent PRDX6 knockdown by shRNAs. shRNA, small hairpin RNA. SCR, scramble. (B) Immunoblot assay to examine the expression of PRDX6 in PSC27 cells upon establishment of stable sublines after shRNA-mediated PRDX6 knockdown. GAPDH, loading control. (C) Quantitative expression analysis of a subgroup of canonical SASP factors at transcription level upon occurrence of BLEO-induced senescence, treatment by apigenin and/or PRDX6 depletion. Data in A and C are shown as mean ± SD and representative of 3 independent biological replicates with P values calculated by Student's t tests. ^, $P > 0.05$; *, $P < 0.05$; **, $P < 0.01$; ***, $P < 0.001$; ****, $P < 0.0001$.

Figure S6. Apigenin reduces PCa cell malignancy conferred by senescent stromal cell-derived conditioned media. (A) A schematic workflow showing the procedure of the indirect co-culture involving stromal cells and PCa cells. CM containing a myriad of soluble factors were collected from native or senescent stromal cells 8-10 d post BLEO treatment, with cells cultured in the absence or presence of apigenin. PCa cells including PC3, DU145, M12 and LNCaP were examined for phenotypic assessments. The CM of an equal number of cells was

collected under each condition. (B) Apoptotic activity measurement of PCa cells in culture supplemented with half-maximal inhibitory concentration (IC50) of DTX. Signal readings proportional to the caspase-3/7 activity of PC3 cells were depicted by relative luminescence units (RLUs). (C) Chemoresistance assay of PCa cells exposed to DTX while being cultured with different types of CM. DTX was administered at a pre-determined IC50 for each cell line. (D) Dose-response curves (nonlinear regression/curve fit) of PC3 cells cultured with the different types of CM, and concurrently exposed to a wide range of concentrations of DTX. Data were plotted on an exponential scale, with cell viability examined relative to the untreated group and calculated as a percentage. (E) Proliferation assay of PCa cells, which were incubated for 3 days with different types of CM as indicated in (a). (F) Migration assay of PCa cells incubated with several types of CM for 18 h. (G) Invasiveness assay of PCa cells across collagen-based transwell membrane incubated with different types of CM. (H) Chemoresistance assay of PCa cells treated with MIT while being cultured with different types of CM. MIT was added at a pre-determined IC50 for each cell line. In e-h, metformin (MET) was used instead of apigenin. PCa, prostate cancer. CM, conditioned media. DTX, docetaxel (a chemotherapeutic agent). MIT, mitoxantrone. SB, SB203580 (a p38MAPK inhibitor that can block the SASP expression). Data in B-H are shown as mean ± SD and representative of 3 independent biological replicates, with P values calculated by Student's t-tests. ^, $P > 0.05$; ^, $P > 0.05$; *, $P < 0.05$; **, $P < 0.01$; ***, $P < 0.001$; ****, $P < 0.0001$.

Figure S7. Experimental design of preclinical regimen, evaluation of *in vivo* SASP expression and appraisal of tumor regression in mice harboring PC3-only xenograft. (A) Comparative statistics of tumor volumes at the end of an 8-week period in NOD/SCID mice carrying PC3 cells alone or admixed with PSC27 cells in the hind flank. PSC27 cells were either naive or senescent induced by BLEO prior to inoculation ($PSC27^{Naive}$ and $PSC27^{SEN}$, respectively). (B) Schematic diagram of drug administration and tumor evaluation of the preclinical trial. Two weeks prior to chemotherapy, PC3 cells alone or together with PSC27 cells were

xenografted subcutaneously to animals. MIT was administrated *via* i.v. on the 1st day of each week starting from the 3rd week, then delivered every other week with a total number of 3 doses. Apigenin was delivered *via* i.p. on the 1st day of every other week starting from 4th week. Mice were sacrificed at the end of the 8-week regimen for tumor evaluation and histological assessment. (C) Transcriptional evaluation of several typical SASP factors (MMP3, ANGPTL4 and AREG) in stromal cells isolated from PC3/PSC27 tumor foci *via* laser capture microdissection (LCM). (D) Transcriptional analysis of senescence markers and several typical SASP factors in stromal cells as depicted in (C). (E) Immunoblots showing the expression of PRDX6 in PC3 sublines. PC3 cells were infected with lentivirus encoding scramble or PRDX6-specific shRNAs. GAPDH, loading control. (F) Comparative statistics of tumor volumes at the end of an 8-week period in NOD/SCID mice carrying PC3-only xenografts in the hind flank. PC3 cells were either naïve or depleted of PRDX6. Mice were subjected to MIT and/or apigenin treatment. (G) Statistical assessment of DNA damage and cell apoptosis in xenograft tissues described in (F). Values are shown as the percentage of cells positively stained by IF or IHC specific to γH2AX or cleaved caspase 3 (CCL3), respectively. Data in A, C, D, F and G are shown as mean ± SD and representative of 3 independent biological replicates, with *P* values calculated by Student's *t*-tests (A, C, D and G) or one-way ANOVA (F). ^, P > 0.05; ^, $P > 0.05$; *, $P < 0.05$; **, $P < 0.01$; ***, $P < 0.001$; ****, $P < 0.0001$.

Figure S8. Evaluation of therapeutic safety of MIT and/or apigenin-mediated preclinical regimens. (A) Measurement of mouse body weights once a week throughout the whole period of the therapeutic regimen. (B) Terminal bleeds were taken from retro-orbital at the end of therapeutic regimens, and subject to analysis of circulating concentrations of creatinine, urea, alkaline phosphatase (ALP) and alanine aminotransferase (ALT) for toxicity evaluation. Data in A and B are shown as mean ± SD and representative of 3 independent biological replicates, with *P* values calculated by one-way ANOVA. ^, P > 0.05.

Figure S9. Apigenin treatment alleviates physical frailty and reduces pathological indices of animals prematurely aged after WBI. (A) H&E and IHC staining (against CD4 or CD19) to histologically profile the morphological changes and physiological status of spleen tissues of C57BL/6J mice. Naïve animals, WBI-treated animals experiencing vehicle treatment and WBI-treated animals receiving apigenin administration, respectively, were recruited to the preclinical study. Scale bar, 400 μm. (B) Representative images (left) and comparative quantification (right) of SA-β-Gal staining of spleen tissues dissected from experimental mice. Animals experiencing different treatments as described in (A) were assessed. Scale bar, 100 μm. (C) IF staining of p21 expressed in kidney tissues. Scale bar, 20 μm. Left, representative images. Right, comparative statistics. (D) Representative images of IHC staining to detect IL6 expression in kidney tissues. Scale bar, 200 μm. (E) Statistic comparison of IL6 staining positivity in kidney tissues as examined in (D). (F-G) ELISA measurement of circulating concentrations of typical SASP factors represented by AREG (F) and EREG (G) in animal serum. (H) Measurement of WBC counts in serum to evaluate the potential effect of preclinical regimens on the immune system and tissue homeostasis of C57BL/6 mice. (I) Percentage of WBC constituents. EO (eosinophil), BASO (basophil), MONO (monocyte), LYMPH (lymphocyte) and NEUT (neutrophil) cell subpopulations were determined separately to categorize these subpopulations. (J-L) Measurement of blood cell components and circulating factors, including RBC count (J, /L), HGB amount (K, g/L) and PLT number (L, /L) in the peripheral blood of animals. (M-P) Routine tests of biochemical parameters. Terminal bleeds were taken from retro-orbital of animals at the end of therapeutic regimens, serum concentrations of creatinine (M, mg/dL), urea (N, mg/dL), ALP (O, U/L) and ALT (P, U/L)) were determined for *in vivo* toxicity appraisal. H&E, hematoxylin and eosin. IHC, immunohistochemistry. IF, immunofluorescence. WBC, white blood cell. RBC, red blood cell. HGB, hemoglobin. PLT, platelets. ALP, alkaline phosphatase. ALT, alanine aminotransferase. Data in B, C, E-P are shown as mean ± SD and representative of 3 independent biological replicates. P values were calculated by Student's t-tests. ^, $P > 0.05$; ^, $P > 0.05$; *,

$P < 0.05$; **, $P < 0.01$; ***, $P < 0.001$; ****, $P < 0.0001$.

Table S1. A full list of natural products in the NMA library subject to screening for senotherapeutic candidates

No.	*Agent name*	*Molecular format*	*CAS no.*	*Cat. no.*
1	(-)-Huperzine A	$C_{15}H_{18}N_2O$	102518-79-6	S2251
2	Acacetin	$C_{16}H_{12}O_5$	480-44-4	S5318
3	Acarbose	$C_{25}H_{43}NO_{18}$	56180-94-0	S1271
4	Apigenin	$C_{15}H_{10}O_5$	520-36-5	S2262
5	Aristolochic acid A	$C_{17}H_{11}NO_7$	313-67-7	S9193
6	Artemisinin	$C_{15}H_{22}O_5$	63968-64-9	S1282
7	Astaxanthin	$C_{40}H_{52}O_4$	472-61-7	S3834
8	Astragalin	$C_{21}H_{20}O_{11}$	480-10-4	S9289
9	Astragaloside IV	$C_{41}H_{68}O_{14}$	84687-43-4	S3901
10	Bavachinin	$C_{21}H_{22}O_4$	19879-30-2	S3878
11	Berberine	$C_{20}H_{18}ClNO_4$	633-65-8	S2271
12	Betulinic acid	$C_{30}H_{48}O_3$	472-15-1	S3603
13	Catechin-hydrate	$C_{15}H_{14}O_6$	225937-10-0	S3974
14	Cinnamic acid	$C_9H_8O_2$	140-10-3	S3677
15	Curculigoside	$C_{22}H_{26}O_{11}$	85643-19-2	S5457
16	Curcumol	$C_{15}H_{24}O_2$	4871-97-0	S2407
17	D-(+)-Trehalose	$C_{12}H_{22}O_{11}$	99-20-7	S9348
18	Danshensu	$C_9H_{10}O_5$	76822-21-4	S4741
19	D-Galactose	$C_6H_{12}O_6$	59-23-4	S3849
20	Dihydrocapsaicin	$C_{18}H_{29}NO_3$	19408-84-5	S9086
21	Echinacoside	$C_{35}H_{46}O_{20}$	82854-37-3	S3783
22	Eleutheroside B	$C_{17}H_{24}O_9$	118-34-3	S3841
23	Ellagic acid	$C_{14}H_6O_8$	476-66-4	S1327
24	Gallic acid	$C_7H_6O_5$	149-91-7	S4603
25	Gastrodin	$C_{13}H_{18}O_7$	62499-27-8	S2383
26	Ginkgolide A	$C_{20}H_{24}O_9$	15291-75-5	S2026
27	Ginkgolide B	$C_{20}H_{24}O_{10}$	15291-77-7	S1343
28	Ginkgolide C	$C_{20}H_{24}O_{11}$	15291-76-6	S3781
29	Ginsenoside Rb1	$C_{54}H_{92}O_{23}$	41753-43-9	S3924
30	Ginsenoside Re	$C_{48}H_{82}O_{18}$	52286-59-6	S3811
31	Ginsenoside Rg1	$C_{42}H_{72}O_{14}$	22427-39-0	S3923
32	Ginsenoside-Rb3	$C_{53}H_{90}O_{22}$	68406-26-8	S9208
33	Ginsenoside-Rc	$C_{53}H_{90}O_{22}$	11021-14-0	S9266
34	Ginsenoside-Rd	$C_{48}H_{82}O_{18}$	52705-93-8	S3931
35	Gynostemma Extract	$C_{47}H_{80}O_{17}$	80321-63-7	S2306
36	Hesperetin	$C_{16}H_{14}O_6$	520-33-2	S2308
37	Hesperidin	$C_{28}H_{34}O_{15}$	520-26-3	S2309
38	Hyperoside	$C_{21}H_{20}O_{12}$	482-36-0	S5453

39	Icariin	$C_{33}H_{40}O_{15}$	489-32-7	S2312
40	Icaritin	$C_{21}H_{20}O_6$	118525-40-9	S9080
41	Isobavachin	$C_{20}H_{20}O_4$	31524-62-6	S9256
42	Isopsoralen	$C_{11}H_6O_3$	523-50-2	S3892
43	Isorhamnetin	$C_{16}H_{12}O_7$	480-19-3	S9111
44	Juglone	$C_{10}H_6O_3$	481-39-0	S5512
45	Kaempferol	$C_{15}H_{10}O_6$	520-18-3	S2314
46	Liquiritigenin	$C_{15}H_{12}O_4$	578-86-9	S3929
47	Liquiritin	$C_{21}H_{22}O_9$	551-15-5	S3930
48	Melatonin	$C_{13}H_{16}N_2O_2$	73-31-4	S1204
49	Monotropein	$C_{16}H_{22}O_{11}$	5945-50-6	S5471
50	Myricetin	$C_{15}H_{10}O_8$	529-44-2	S2326
51	Myricitrin	$C_{21}H_{20}O_{12}$	17912-87-7	S2327
52	Naringin	$C_{27}H_{32}O_{14}$	10236-47-2	S2329
53	Notoginsenoside-R1	$C_{47}H_{80}O_{18}$	80418-24-2	S3785
54	Oleuropein	$C_{25}H_{32}O_{13}$	32619-42-4	S7867
55	Resveratrol	$C_{14}H_{12}O_3$	501-36-0	S1396
56	Rutin	$C_{27}H_{30}O_{16}$	153-18-4	S2350
57	Specnuezhenide	$C_{31}H_{42}O_{17}$	449733-84-0	S9066
58	Spermidine	$C_7H_{19}N_3$	124-20-9	S3569
59	Taxifolin	$C_{15}H_{12}O_7$	480-18-2	S2366
60	Uridine	$C_9H_{12}N_2O_6$	58-96-8	S2029
61	Vanillin	$C_8H_8O_3$	121-33-5	S3071
62	Verbascoside	$C_{29}H_{36}O_{15}$	61276-17-3	S5458
63	Vitamin C	$C_6H_8O_6$	50-81-7	S3114
64	Xanthohumol	$C_{21}H_{22}O_5$	6754-58-1	S7889
65	ABT-263	$C_{47}H_{55}ClF_3N_5O_6S_3$	923564-51-6	S1001
66	procyanidin C1	$C_{45}H_{38}O_{18}$	37064-30-5	E0478

Table S2. A full list of potental target human proteins of apigenin as identified by DARTS-MS assays

Majority protein IDs	Protein names	GN	Number of proteins	Peptides	Unique peptides	Sequence coverage [%]	Mol. weight [kDa]	LFQ intensity_ DMSO-1	LFQ intensity_ DMSO-2	LFQ intensity_ DMSO-3	LFQ intensity_ APIG-1	LFQ intensity_ APIG-2	LFQ intensity_ APIG-3	Ratio_APIG/DMSO	P value
Q15019	Septin-2	SEPT2	1	8	8	19.7	41.487	1.1E+08	1.4E+08	1.3E+08	1.1E+08	1.2E+08	1.4E+08	0.96601	0.72936
Q16181	Septin-7	SEPT7	1	3	3	9.2	50.679	1.1E+08	8.3E+07	1.2E+08	1.1E+08	1.2E+08	1.2E+08	1.13009	0.29227
Q9UHD8	Septin-9	SEPT9	1	3	3	6	65.401	NaN	NaN	5.1E+07	4.1E+07	4.7E+07	NaN	0.86721	NaN
Q9NVA2	Septin-11	SEPT11	1	2	2	5.8	49.398	NaN	NaN	NaN	2.4E+07	2.4E+07	2.6E+07	NaN	NaN
Q86V21	Acetoacetyl-CoA synthetase	AACS	1	1	1	2.7	75.143	NaN	NaN	NaN	NaN	NaN	NaN	NaN	NaN
P49588	Alanine--tRNA ligase, cytoplasmic	AARS	1	6	6	7.5	106.81	2E+08	2.1E+08	1.7E+08	2.5E+08	2E+08	2E+08	1.12857	0.3062
Q9NRN7	L-aminoadipate-semialdehyde dehydrogenase-phosphopante	AASDHPPT	1	1	1	5.5	35.776	NaN	NaN	NaN	NaN	NaN	NaN	NaN	NaN
P33527	Multidrug resistance-associated protein 1	ABCC1	1	6	6	5.4	171.59	6.1E+07	7.6E+07	8.5E+07	5.4E+07	8.2E+07	5.9E+07	0.88205	0.46762
O15438	Canalicular multispecific organic anion transporter 2	ABCC3	1	2	2	2	169.34	NaN	NaN	NaN	NaN	NaN	NaN	NaN	NaN
P28288	ATP-binding cassette sub-family D member 3	ABCD3	1	1	1	2.3	75.475	NaN	NaN	NaN	NaN	NaN	NaN	NaN	NaN
Q8NE71	ATP-binding cassette sub-family F member 1	ABCF1	1	3	3	4.1	95.925	2.4E+07	NaN	2.3E+07	NaN	2.4E+07	2.6E+07	1.06497	0.29697
Q9UG63	ATP-binding cassette sub-family F member 2	ABCF2	1	1	1	2.2	71.289	NaN	NaN	NaN	NaN	NaN	NaN	NaN	NaN
Q9NUJ1	Mycophenolic acid acyl-glucuronide esterase, mitochondrial	ABHD10	1	2	2	10.1	33.932	NaN	NaN	NaN	NaN	NaN	NaN	NaN	NaN
Q8N2K0	Monoacylglycerol lipase ABHD12	ABHD12	1	1	1	3	45.096	NaN	NaN	NaN	NaN	NaN	NaN	NaN	NaN
Q12979	Active breakpoint cluster region-related protein	ABR	1	1	1	1	97.597	NaN	NaN	NaN	NaN	NaN	NaN	NaN	NaN
Q13085	Acetyl-CoA carboxylase 1;Biotin carboxylase	ACACA	1	4	4	2.5	265.55	1.4E+07	2E+07	1.5E+07	1.5E+07	1.8E+07	1.6E+07	0.9969	0.98135
Q9H845	Acyl-CoA dehydrogenase family member 9, mitochondrial	ACAD9	1	3	3	5.6	68.76	2.4E+07	NaN	2.4E+07	2E+07	2.3E+07	2.4E+07	0.93713	0.36997
P49748	Very long-chain specific acyl-CoA dehydrogenase, mitochond	ACADVL	1	8	8	14.4	70.389	1.2E+08	1.2E+08	1.1E+08	1.2E+08	1.2E+08	1.3E+08	1.09345	0.02488
P24752	Acetyl-CoA acetyltransferase, mitochondrial	ACAT1	1	2	2	5.4	45.199	NaN	NaN	NaN	NaN	NaN	NaN	NaN	NaN
Q9H3P7	Golgi resident protein GCP60	ACBD3	1	3	3	10	60.593	2.4E+07	2.6E+07	2.8E+07	3.2E+07	3.1E+07	2.9E+07	1.17164	0.03144
Q9UKV3	Apoptotic chromatin condensation inducer in the nucleus	ACIN1	1	1	1	1.4	151.86	NaN	NaN	NaN	NaN	NaN	NaN	NaN	NaN
P53396	ATP-citrate synthase	ACLY	1	22	22	20.8	120.84	5.7E+08	5.7E+08	5.5E+08	6.5E+08	5.9E+08	5.4E+08	1.05507	0.41196
Q99798	Aconitate hydratase, mitochondrial	ACO2	1	9	9	12.1	85.424	3.5E+07	4.7E+07	3.5E+07	2.9E+07	3.6E+07	3.8E+07	0.88889	0.43397
P49753;Q86TX2	Acyl-coenzyme A thioesterase 2, mitochondrial;Acyl-coenzyme	ACOT2	3	4	4	10.4	53.218	1.8E+07	1.8E+07	1.9E+07	2E+07	2.1E+07	2.2E+07	1.11291	0.04381
O00154	Cytosolic acyl coenzyme A thioester hydrolase	ACOT7	1	1	1	3.9	41.796	NaN	NaN	NaN	NaN	NaN	NaN	NaN	NaN
Q9Y305	Acyl-coenzyme A thioesterase 9, mitochondrial	ACOT9	1	2	2	4.3	49.901	NaN	NaN	NaN	NaN	NaN	NaN	NaN	NaN
O15254	Peroxisomal acyl-coenzyme A oxidase 3	ACOX3	1	1	1	2.4	77.628	NaN	NaN	NaN	NaN	NaN	NaN	NaN	NaN
P24666	Low molecular weight phosphotyrosine protein phosphatase	ACP1	1	2	2	13.3	18.042	1.6E+07	NaN	NaN	NaN	1.1E+07	2.3E+07	1.05889	NaN
P11117	Lysosomal acid phosphatase	ACP2	1	1	1	4	48.344	NaN	NaN	NaN	NaN	NaN	NaN	NaN	NaN
Q96CM8	Acyl-CoA synthetase family member 2, mitochondrial	ACSF2	1	1	1	2.4	68.124	NaN	NaN	NaN	NaN	NaN	NaN	NaN	NaN
P33121	Long-chain-fatty-acid--CoA ligase 1	ACSL1	1	2	2	2.9	77.942	NaN	NaN	NaN	NaN	NaN	NaN	NaN	NaN
O95573	Long-chain-fatty-acid--CoA ligase 3	ACSL3	1	3	3	6.1	80.419	NaN	NaN	NaN	NaN	NaN	NaN	NaN	NaN
P68133;P68032;P62736	Actin, alpha skeletal muscle;Actin, alpha cardiac muscle 1;Actin	ACTA1	3	8	1	17.5	42.051	NaN	NaN	NaN	NaN	NaN	NaN	NaN	NaN
P63261;P60709;P63267	Actin, cytoplasmic 2;Actin, cytoplasmic 2, N-terminally process	ACTG1	9	11	4	30.7	41.792	5.2E+09	5.4E+09	5.7E+09	6.2E+09	5.7E+09	5.5E+09	1.06308	0.26426
P12814	Alpha-actinin-1	ACTN1	3	20	14	20.4	103.06	4.8E+08	4E+08	4.4E+08	4.2E+08	5.1E+08	5E+08	1.08645	0.35693
O43707	Alpha-actinin-4	ACTN4	1	19	13	20.5	104.85	6.2E+08	4.7E+08	6.4E+08	4.7E+08	6.3E+08	6E+08	0.98514	0.91184
P61163	Alpha-centractin	ACTR1A	1	2	2	6.9	42.613	NaN	NaN	NaN	NaN	NaN	NaN	NaN	NaN
P42025	Beta-centractin	ACTR1B	1	1	1	4.5	42.293	NaN	NaN	NaN	NaN	NaN	NaN	NaN	NaN
P61160	Actin-related protein 2	ACTR2	1	4	4	11.2	44.76	4.6E+07	4E+07	4.2E+07	4.3E+07	4.7E+07	NaN	1.04004	0.59558
P61158	Actin-related protein 3	ACTR3	3	7	7	15.8	47.371	1.8E+08	1.8E+08	1.8E+08	1.6E+08	1.7E+08	1.5E+08	0.90949	0.04326
P00813	Adenosine deaminase	ADA	1	2	2	6.3	40.764	NaN	NaN	NaN	NaN	NaN	NaN	NaN	NaN
O14672	Disintegrin and metalloproteinase domain-containing protein	ADAM10	1	3	3	4.3	84.141	3.5E+07	3.7E+07	3.8E+07	2.8E+07	3.3E+07	3.5E+07	0.87458	0.13582
Q13443	Disintegrin and metalloproteinase domain-containing protein	ADAM9	1	1	1	1.7	90.555	NaN	NaN	NaN	NaN	NaN	NaN	NaN	NaN
Q8N6G6	ADAMTS-like protein 1	ADAMTSL1	1	1	1	0.7	193.41	NaN	NaN	NaN	NaN	NaN	NaN	NaN	NaN
P55263	Adenosine kinase	ADK	1	3	3	9.4	40.545	5.1E+07	4.2E+07	6.1E+07	2.9E+07	6.2E+07	7.6E+07	1.08874	0.7759
Q96SZ5	2-aminoethanethiol dioxygenase	ADO	1	1	1	5.6	29.751	NaN	NaN	NaN	NaN	NaN	NaN	NaN	NaN
Q8NDY3	[Protein ADP-ribosylarginine] hydrolase-like protein 1	ADPRHL1	1	1	1	2	40.104	NaN	NaN	NaN	NaN	NaN	NaN	NaN	NaN
Q16186	Proteasomal ubiquitin receptor ADRM1	ADRM1	1	2	2	4.9	42.153	7.2E+07	5.5E+07	5.6E+07	5.5E+07	4.8E+07	4E+07	0.77521	0.12494
P30566	Adenylosuccinate lyase	ADSL	1	1	1	2.7	54.889	NaN	NaN	NaN	NaN	NaN	NaN	NaN	NaN
P30520	Adenylosuccinate synthetase isozyme 2	ADSS	1	3	3	8.3	50.097	6.9E+07	8.5E+07	6.8E+07	7.7E+07	8.9E+07	6.4E+07	1.0356	0.78506
Q9Y4W6	AFG3-like protein 2	AFG3L2	1	3	3	3.5	88.583	2.5E+07	2.3E+07	NaN	1.8E+07	NaN	2.1E+07	0.8069	0.17772
P52594	Arf-GAP domain and FG repeat-containing protein 1	AGFG1	1	1	1	2.1	58.259	NaN	NaN	NaN	NaN	NaN	NaN	NaN	NaN

P35573	Glycogen debranching enzyme;4-alpha-glucanotransferase;Ar	AGL	1	3	3	2.3	174.76	NaN	NaN	NaN	NaN	NaN	NaN	NaN	NaN
Q9UL18;Q9UKV8;Q9H9	Protein argonaute-1;Protein argonaute-2;Protein argonaute-3	AGO1	4	1	1	1.6	97.213	NaN	NaN	NaN	NaN	NaN	NaN	NaN	NaN
Q9NRZ7	1-acyl-sn-glycerol-3-phosphate acyltransferase gamma	AGPAT3	1	1	1	3.2	43.38	NaN	NaN	NaN	NaN	NaN	NaN	NaN	NaN
Q53EU6	Glycerol-3-phosphate acyltransferase 3	AGPAT9	1	1	1	3	48.705	NaN	NaN	NaN	NaN	NaN	NaN	NaN	NaN
O95994	Anterior gradient protein 2 homolog	AGR2	1	1	1	6.3	19.979	NaN	NaN	NaN	NaN	NaN	NaN	NaN	NaN
P23526	Adenosylhomocysteinase	AHCY	1	11	11	19.9	47.716	2.1E+08	2.2E+08	2.2E+08	2.5E+08	2E+08	2.4E+08	1.06728	0.35324
O43865;Q96HN2	Putative adenosylhomocysteinase 2;Putative adenosylhomocys	AHCYL1	2	3	3	6	58.951	NaN	7.3E+07	9.2E+07	NaN	6.2E+07	9.1E+07	0.92646	0.75803
Q09666	Neuroblast differentiation-associated protein AHNAK	AHNAK	1	48	48	21	629.09	8.8E+07	7.5E+07	9.7E+07	8.3E+07	8E+07	9.1E+07	0.9787	0.81021
Q8IVF2	Protein AHNAK2	AHNAK2	1	4	4	1.4	616.62	1.9E+07	1.9E+07	1.5E+07	1.4E+07	2E+07	2.3E+07	1.07515	0.65313
O95433	Activator of 90 kDa heat shock protein ATPase homolog 1	AHSA1	1	2	2	9.2	38.274	NaN	NaN	NaN	NaN	NaN	NaN	NaN	NaN
O95831	Apoptosis-inducing factor 1, mitochondrial	AIFM1	1	1	1	2	66.9	NaN	NaN	NaN	NaN	NaN	NaN	NaN	NaN
Q9BRQ8	Apoptosis-inducing factor 2	AIFM2	1	1	1	3.5	40.526	NaN	NaN	NaN	NaN	NaN	NaN	NaN	NaN
Q12904	Aminoacyl tRNA synthase complex-interacting multifunctional	AIMP1	1	2	2	9.9	34.352	NaN	NaN	NaN	NaN	NaN	NaN	NaN	NaN
O00170	AH receptor-interacting protein	AIP	1	1	1	3.9	37.664	NaN	NaN	NaN	NaN	NaN	NaN	NaN	NaN
P54819	Adenylate kinase 2, mitochondrial;Adenylate kinase 2, mitocho	AK2	1	6	6	33.5	26.477	8.9E+07	7.5E+07	5.6E+07	6.5E+07	8.9E+07	7.6E+07	1.04353	0.79934
Q9UIJ7	GTP:AMP phosphotransferase AK3, mitochondrial	AK3	1	4	4	21.6	25.565	1.1E+08	7.5E+07	7.8E+07	1.4E+08	1.2E+08	1.2E+08	1.39003	0.06465
Q02952	A-kinase anchor protein 12	AKAP12	1	1	1	1.2	191.48	NaN	NaN	NaN	NaN	NaN	NaN	NaN	NaN
Q9ULX6	A-kinase anchor protein 8-like	AKAP8L	1	1	1	2.2	71.639	NaN	NaN	NaN	NaN	NaN	NaN	NaN	NaN
Q5T1N1	Protein AKNAD1	AKNAD1	1	1	1	1	92.863	NaN	NaN	NaN	NaN	NaN	NaN	NaN	NaN
P14550	Alcohol dehydrogenase [NADP(+)]	AKR1A1	1	1	1	3.4	36.573	NaN	NaN	NaN	NaN	NaN	NaN	NaN	NaN
P15121	Aldose reductase	AKR1B1	1	9	8	23.4	35.853	4E+09	4.6E+09	5E+09	4.3E+09	4.9E+09	4.3E+09	0.99226	0.92507
O60218	Aldo-keto reductase family 1 member B10	AKR1B10	1	11	7	27.2	36.019	2.2E+09	2.3E+09	2.2E+09	2.2E+09	2E+09	2E+09	0.94613	0.15916
Q04828	Aldo-keto reductase family 1 member C1	AKR1C1	1	8	0	24.8	36.788	NaN	NaN	NaN	NaN	NaN	NaN	NaN	NaN
P52895	Aldo-keto reductase family 1 member C2	AKR1C2	1	6	1	16.4	36.735	NaN	NaN	NaN	NaN	NaN	NaN	NaN	NaN
P42330;P17516	Aldo-keto reductase family 1 member C3;Aldo-keto reductase	AKR1C3	2	8	2	24.8	36.853	1.1E+09	1E+09	8.9E+08	1.2E+09	9.7E+08	1.1E+09	1.09403	0.32883
P13716	Delta-aminolevulinic acid dehydratase	ALAD	1	2	2	10.3	36.294	NaN	NaN	NaN	NaN	NaN	NaN	NaN	NaN
Q13740	CD166 antigen	ALCAM	1	5	5	10.3	65.102	3.7E+07	3.5E+07	4E+07	3.9E+07	4E+07	4.3E+07	1.09361	0.12359
Q8IZ83	Aldehyde dehydrogenase family 16 member A1	ALDH16A1	1	2	2	3.4	85.126	2.3E+07	2E+07	2.1E+07	2.4E+07	2.3E+07	NaN	1.0908	0.20841
P54886	Delta-1-pyrroline-5-carboxylate synthase;Glutamate 5-kinase;	ALDH18A1	1	3	3	3.3	87.301	4E+07	NaN	4.1E+07	5.5E+07	4.1E+07	3.7E+07	1.09131	0.62978
P00352	Retinal dehydrogenase 1	ALDH1A1	3	21	17	34.3	54.861	3.9E+09	3.9E+09	4E+09	4.4E+09	4E+09	3.8E+09	1.03969	0.47357
P30837	Aldehyde dehydrogenase X, mitochondrial	ALDH1B1	1	4	2	6	57.249	2.8E+07	3E+07	3.6E+07	NaN	2.7E+07	NaN	0.86963	NaN
O75891	Cytosolic 10-formyltetrahydrofolate dehydrogenase	ALDH1L1	1	1	1	1.7	98.828	NaN	NaN	NaN	NaN	NaN	NaN	NaN	NaN
P05091	Aldehyde dehydrogenase, mitochondrial	ALDH2	1	7	3	10.4	56.381	7E+07	7.1E+07	7.2E+07	7.9E+07	6.3E+07	6.6E+07	0.97557	0.74546
P30838	Aldehyde dehydrogenase, dimeric NADP-preferring	ALDH3A1	2	8	7	18.5	50.394	6.6E+08	7.6E+08	7E+08	7.5E+08	6.8E+08	7.1E+08	1.00789	0.87696
P51648	Fatty aldehyde dehydrogenase	ALDH3A2	1	4	3	11.8	54.847	8809800	NaN	1.2E+07	NaN	1.5E+07	1E+07	1.22244	0.5276
P43353	Aldehyde dehydrogenase family 3 member B1	ALDH3B1	1	2	1	5.6	51.839	NaN	NaN	NaN	NaN	NaN	NaN	NaN	NaN
P30038	Delta-1-pyrroline-5-carboxylate dehydrogenase, mitochondri	ALDH4A1	1	2	2	4.3	61.719	1.8E+07	1.7E+07	1.9E+07	1.7E+07	2.1E+07	NaN	1.07311	0.47058
P51649	Succinate-semialdehyde dehydrogenase, mitochondrial	ALDH5A1	1	1	1	2.6	57.214	NaN	NaN	NaN	NaN	NaN	NaN	NaN	NaN
P49419	Alpha-aminoadipic semialdehyde dehydrogenase	ALDH7A1	1	2	2	3.3	58.486	NaN	3.5E+07	3.7E+07	3.4E+07	3.5E+07	3.7E+07	0.9864	0.77034
P49189	4-trimethylaminobutyraldehyde dehydrogenase	ALDH9A1	1	3	3	7.3	53.801	8.9E+07	8.7E+07	8.6E+07	7.5E+07	8.8E+07	8.7E+07	0.95033	0.35674
P04075	Fructose-bisphosphate aldolase A	ALDOA	2	9	9	17.3	39.42	4.2E+08	4.6E+08	4.4E+08	5.1E+08	4.5E+08	4.5E+08	1.07433	0.2581
Q86SJ2	Amphoterin-induced protein 2	AMIGO2	1	1	1	3.1	57.933	NaN	NaN	NaN	NaN	NaN	NaN	NaN	NaN
Q01433	AMP deaminase 2	AMPD2	1	2	2	2.8	100.69	NaN	NaN	NaN	NaN	NaN	NaN	NaN	NaN
Q9UJX5	Anaphase-promoting complex subunit 4	ANAPC4	1	1	1	1.4	92.115	NaN	NaN	NaN	NaN	NaN	NaN	NaN	NaN
Q4KMQ2	Anoctamin-6	ANO6	1	1	1	1.1	106.16	NaN	NaN	NaN	NaN	NaN	NaN	NaN	NaN
P39687;O95626	Acidic leucine-rich nuclear phosphoprotein 32 family member	ANP32A	3	4	4	10.4	28.585	2E+08	2.3E+08	2.1E+08	2E+08	1.8E+08	2.1E+08	0.92111	0.16266
Q9H6X2	Anthrax toxin receptor 1	ANTXR1	1	1	1	2.1	62.788	NaN	NaN	NaN	NaN	NaN	NaN	NaN	NaN
P04083	Annexin A1	ANXA1	1	7	7	17.3	38.714	7.7E+08	9.7E+08	8.8E+08	8E+08	7.3E+08	8E+08	0.88724	0.19208
P50995	Annexin A11	ANXA11	1	4	4	8.7	54.389	9.8E+07	9.4E+07	1.1E+08	8.5E+07	1.1E+08	9.4E+07	0.94164	0.48416
P07355;A6NMY6	Annexin A2;Putative annexin A2-like protein	ANXA2	2	15	15	34.5	38.604	1.6E+09	1.7E+09	1.7E+09	1.4E+09	1.6E+09	1.6E+09	0.91517	0.09186
P12429	Annexin A3	ANXA3	1	7	7	20.7	36.375	8.5E+07	7.3E+07	9.5E+07	7.3E+07	8.2E+07	8E+07	0.92635	0.41738
P09525	Annexin A4	ANXA4	1	7	7	21.9	35.882	4.2E+08	4.3E+08	4.3E+08	4.3E+08	4E+08	3.8E+08	0.94673	0.23734
P08758	Annexin A5	ANXA5	1	6	6	21.9	35.936	3.7E+08	3.7E+08	4.4E+08	3.2E+08	3.4E+08	4.1E+08	0.9083	0.39675
P08133	Annexin A6	ANXA6	1	10	10	17.1	75.872	5.2E+07	4.6E+07	5.2E+07	3.6E+07	4.5E+07	4.4E+07	0.83951	0.08195
P20073	Annexin A7	ANXA7	1	4	4	9	52.739	7.8E+07	9.6E+07	8.3E+07	7.2E+07	7.9E+07	7.3E+07	0.87333	0.14208
Q5VT79;P13928	Annexin A8-like protein 2;Annexin A8	ANXA8L2	2	1	1	4.9	36.879	NaN	NaN	NaN	NaN	NaN	NaN	NaN	NaN
O43747	AP-1 complex subunit gamma-1	AP1G1	1	1	1	1.3	91.35	NaN	NaN	NaN	NaN	NaN	NaN	NaN	NaN
Q9BXS5	AP-1 complex subunit mu-1	AP1M1	1	2	2	6.1	48.586	NaN	1.2E+07	NaN	8370500	NaN	NaN	0.71918	NaN
O95782	AP-2 complex subunit alpha-1	AP2A1	1	5	4	6	107.54	3E+07	3.7E+07	2.3E+07	2.5E+07	2.1E+07	3E+07	0.84972	0.38311

O94973	AP-2 complex subunit alpha-2	AP2A2	1	5	4	5	103.96	1.5E+07	NaN	1.2E+07	1.6E+07	1.6E+07	1.9E+07	1.2803	0.09998
P63010;Q10567	AP-2 complex subunit beta;AP-1 complex subunit beta-1	AP2B1	2	5	5	5.3	104.55	1.2E+08	6.9E+07	1.1E+08	1.1E+08	9.7E+07	1.2E+08	1.12449	0.50576
Q96CW1	AP-2 complex subunit mu	AP2M1	1	2	2	4.1	49.654	6.1E+07	NaN	7.1E+07	NaN	NaN	7.3E+07	1.10283	NaN
P53680	AP-2 complex subunit sigma	AP2S1	1	1	1	5.6	17.018	NaN	NaN	NaN	NaN	NaN	NaN	NaN	NaN
O00203	AP-3 complex subunit beta-1	AP3B1	1	5	5	4.7	121.32	1.8E+07	2.6E+07	2.2E+07	NaN	2.1E+07	1.9E+07	0.90457	0.52507
O14617	AP-3 complex subunit delta-1	AP3D1	1	2	2	2.1	130.16	2.1E+07	2.1E+07	NaN	1.6E+07	2.1E+07	NaN	0.8744	0.38644
P27695	DNA-(apurinic or apyrimidinic site) lyase;DNA-(apurinic or apy	APEX1	1	3	3	15.7	35.554	NaN	NaN	NaN	NaN	NaN	NaN	NaN	NaN
Q9BZZ5	Apoptosis inhibitor 5	API5	1	4	4	10.3	59.004	7.3E+07	6.7E+07	7.4E+07	6.5E+07	6.4E+07	6.8E+07	0.92374	0.11112
Q96GX9	Methylthioribulose-1-phosphate dehydratase	APIP	1	1	1	7	27.125	NaN	NaN	NaN	NaN	NaN	NaN	NaN	NaN
Q9HDC9	Adipocyte plasma membrane-associated protein	APMAP	1	5	5	12.7	46.48	NaN	8.9E+07	NaN	6.7E+07	9.2E+07	NaN	0.89358	NaN
Q8NCW5	NAD(P)H-hydrate epimerase	APOA1BP	1	2	2	8.3	31.674	NaN	NaN	NaN	NaN	NaN	NaN	NaN	NaN
P04114	Apolipoprotein B-100;Apolipoprotein B-48	APOB	1	1	1	0.2	515.6	NaN	NaN	NaN	NaN	NaN	NaN	NaN	NaN
Q9NRW3	DNA dC->dU-editing enzyme APOBEC-3C	APOBEC3C	1	1	1	6.8	22.826	NaN	NaN	NaN	NaN	NaN	NaN	NaN	NaN
Q9BQE5	Apolipoprotein L2	APOL2	1	1	1	3.3	37.078	3E+07	NaN	3.1E+07	3.5E+07	3.4E+07	1.8E+07	0.95567	0.85778
P05067	Amyloid beta A4 protein;N-APP;Soluble APP-alpha;Soluble AP	APP	1	1	1	2.1	86.942	NaN	NaN	NaN	NaN	NaN	NaN	NaN	NaN
P07741	Adenine phosphoribosyltransferase	APRT	1	4	4	26.1	19.608	2.7E+07	4.6E+07	3.7E+07	3.1E+07	4.1E+07	3.2E+07	0.9472	0.7696
P48444	Coatomer subunit delta	ARCN1	1	1	1	2.5	57.21	NaN	NaN	NaN	NaN	NaN	NaN	NaN	NaN
P84077;P61204	ADP-ribosylation factor 1;ADP-ribosylation factor 3	ARF1	2	1	1	4.4	20.697	NaN	NaN	NaN	NaN	NaN	NaN	NaN	NaN
P18085	ADP-ribosylation factor 4	ARF4	1	1	1	5.6	20.511	NaN	NaN	NaN	NaN	NaN	NaN	NaN	NaN
P84085	ADP-ribosylation factor 5	ARF5	1	2	2	10	20.529	2.4E+07	NaN	NaN	NaN	1.4E+07	2.9E+07	0.91234	NaN
Q07960	Rho GTPase-activating protein 1	ARHGAP1	1	2	2	5.2	50.435	6.6E+07	4E+07	NaN	1.2E+08	7.1E+07	9.2E+07	1.7831	0.13657
Q8N392	Rho GTPase-activating protein 18	ARHGAP18	1	1	1	1.4	74.976	NaN	NaN	NaN	NaN	NaN	NaN	NaN	NaN
Q9NRY4	Rho GTPase-activating protein 35	ARHGAP35	1	1	1	1.1	170.51	NaN	NaN	NaN	NaN	NaN	NaN	NaN	NaN
P52565	Rho GDP-dissociation inhibitor 1	ARHGDIA	1	2	2	10.8	23.207	NaN	NaN	NaN	NaN	NaN	NaN	NaN	NaN
Q92888	Rho guanine nucleotide exchange factor 1	ARHGEF1	1	5	5	6.1	102.43	6.6E+07	5.3E+07	6.7E+07	6.6E+07	6.4E+07	7.7E+07	1.10734	0.32464
Q6ZSZ5	Rho guanine nucleotide exchange factor 18	ARHGEF18	1	1	1	1.3	151.64	NaN	NaN	NaN	NaN	NaN	NaN	NaN	NaN
Q9Y4X5	E3 ubiquitin-protein ligase ARIH1	ARIH1	1	1	1	2.5	64.117	NaN	NaN	NaN	NaN	NaN	NaN	NaN	NaN
Q9Y2Y0	ADP-ribosylation factor-like protein 2-binding protein	ARL2BP	1	1	1	5.5	18.822	NaN	NaN	NaN	NaN	NaN	NaN	NaN	NaN
P36405	ADP-ribosylation factor-like protein 3	ARL3	1	2	2	9.3	20.455	1.7E+07	2E+07	1.6E+07	2.2E+07	1.6E+07	2.1E+07	1.12778	0.37829
O75915	PRA1 family protein 3	ARL6IP5	1	1	1	5.9	21.614	NaN	NaN	NaN	NaN	NaN	NaN	NaN	NaN
Q96BM9	ADP-ribosylation factor-like protein 8A	ARL8A	1	1	1	4.8	21.416	NaN	NaN	NaN	NaN	NaN	NaN	NaN	NaN
Q9H993	Protein-glutamate O-methyltransferase	ARMT1	1	4	4	7.5	51.172	5.3E+07	6.1E+07	6.7E+07	5.8E+07	5.7E+07	5.2E+07	0.91879	0.35765
O15143	Actin-related protein 2/3 complex subunit 1B	ARPC1B	1	2	2	5.9	40.949	NaN	NaN	NaN	NaN	NaN	NaN	NaN	NaN
O15145	Actin-related protein 2/3 complex subunit 3	ARPC3	1	2	2	13.5	20.546	4.6E+07	4.8E+07	4.6E+07	5E+07	4.5E+07	4.2E+07	0.97967	0.69553
P59998	Actin-related protein 2/3 complex subunit 4	ARPC4	1	4	4	21.4	19.667	1.4E+08	1.2E+08	8.9E+07	1.4E+08	1.4E+08	1.3E+08	1.19142	0.21026
O15511	Actin-related protein 2/3 complex subunit 5	ARPC5	1	2	2	23.8	16.32	NaN	NaN	NaN	NaN	NaN	NaN	NaN	NaN
O43681	ATPase ASNA1	ASNA1	1	1	1	2.6	38.792	NaN	NaN	NaN	NaN	NaN	NaN	NaN	NaN
Q12797	Aspartyl/asparaginyl beta-hydroxylase	ASPH	1	11	11	19.3	85.862	2.6E+08	3.2E+08	2.3E+08	2.6E+08	2.2E+08	2.3E+08	0.88353	0.32101
Q9NVM9	Protein asunder homolog	ASUN	1	1	1	1.6	80.224	NaN	NaN	NaN	NaN	NaN	NaN	NaN	NaN
P31939	Bifunctional purine biosynthesis protein PURH;Phosphoribosyla	ATIC	1	5	5	8.3	64.615	8.7E+07	1.2E+08	1E+08	9.5E+07	1E+08	8.5E+07	0.90633	0.41085
Q8NHH9	Atlastin-2	ATL2	2	3	3	7.2	66.228	1.5E+07	NaN	1.2E+07	1.2E+07	NaN	NaN	0.85961	NaN
Q6DD88	Atlastin-3	ATL3	1	2	2	4.1	60.541	1.2E+08	NaN	5.9E+07	1.2E+08	9.2E+07	1.2E+08	1.25331	0.44306
O00244	Copper transport protein ATOX1	ATOX1	1	1	1	13.2	7.4016	NaN	NaN	NaN	NaN	NaN	NaN	NaN	NaN
P05023	Sodium/potassium-transporting ATPase subunit alpha-1	ATP1A1	3	9	9	10.9	112.89	8.6E+07	8.8E+07	8.3E+07	9.3E+07	8E+07	1E+08	1.06698	0.43069
P05026	Sodium/potassium-transporting ATPase subunit beta-1	ATP1B1	1	4	4	16.5	35.061	6.1E+07	6.6E+07	4.2E+07	8.4E+07	7.5E+07	6.9E+07	1.34938	0.08363
P16615	Sarcoplasmic/endoplasmic reticulum calcium ATPase 2	ATP2A2	3	10	10	10.1	114.76	1.6E+08	1.5E+08	1.5E+08	1.9E+08	1.4E+08	1.6E+08	1.06241	0.50113
P20020	Plasma membrane calcium-transporting ATPase 1	ATP2B1	2	3	2	5	134.68	2.8E+07	4.2E+07	2.9E+07	1.9E+07	NaN	2.9E+07	0.73836	0.30228
P23634	Plasma membrane calcium-transporting ATPase 4	ATP2B4	1	2	1	2.7	137.92	NaN	NaN	NaN	NaN	NaN	NaN	NaN	NaN
P25705	ATP synthase subunit alpha, mitochondrial	ATP5A1	1	7	7	12.7	59.75	2.3E+08	4E+08	2.8E+08	2.4E+08	3.9E+08	2.3E+08	0.9488	0.83553
P06576	ATP synthase subunit beta, mitochondrial	ATP5B	1	5	5	10.8	56.559	1.8E+08	1.9E+08	1.7E+08	1.2E+08	1.6E+08	1.9E+08	0.8941	0.41121
O75947	ATP synthase subunit d, mitochondrial	ATP5H	1	1	1	5.6	18.491	NaN	NaN	NaN	NaN	NaN	NaN	NaN	NaN
P18859	ATP synthase-coupling factor 6, mitochondrial	ATP5J	1	3	3	30.6	12.587	NaN	3.7E+07	2.2E+07	NaN	2.1E+07	2.2E+07	0.73152	0.39237
P56134	ATP synthase subunit f, mitochondrial	ATP5J2	1	1	1	13.8	10.918	NaN	NaN	NaN	NaN	NaN	NaN	NaN	NaN
O75964	ATP synthase subunit g, mitochondrial	ATP5L	1	1	1	12.6	11.428	NaN	NaN	NaN	NaN	NaN	NaN	NaN	NaN
Q93050	V-type proton ATPase 116 kDa subunit a isoform 1	ATP6V0A1	1	1	1	2.3	96.412	NaN	NaN	NaN	NaN	NaN	NaN	NaN	NaN
P38606	V-type proton ATPase catalytic subunit A	ATP6V1A	1	6	6	10.7	68.303	NaN	5.1E+07	3.6E+07	6.9E+07	6.9E+07	5.4E+07	1.46737	0.09181
P36543	V-type proton ATPase subunit E 1	ATP6V1E1	1	1	1	6.2	26.145	NaN	NaN	NaN	NaN	NaN	NaN	NaN	NaN
Q9UBB4	Ataxin-10	ATXN10	1	2	2	4.8	53.488	2E+07	NaN	NaN	1.5E+07	NaN	NaN	0.7645	NaN
Q9H3M9	Putative ataxin-3-like protein	ATXN3L	1	1	1	2.5	40.747	NaN	NaN	NaN	NaN	NaN	NaN	NaN	NaN

P0DMR3	Putative protein ATXN8OS	ATXN8OS	1	1	1	4.5	22.759	NaN	NaN	NaN	NaN	NaN	NaN	NaN	NaN
Q9Y679	Ancient ubiquitous protein 1	AUP1	1	1	1	2.9	45.786	NaN	NaN	NaN	NaN	NaN	NaN	NaN	NaN
O95816	BAG family molecular chaperone regulator 2	BAG2	1	3	3	13.7	23.772	3.4E+07	2.7E+07	3.2E+07	3.2E+07	NaN	3.2E+07	1.03038	0.73774
O95817	BAG family molecular chaperone regulator 3	BAG3	1	2	2	6.4	61.594	NaN	NaN	NaN	NaN	NaN	NaN	NaN	NaN
Q9UHR4	Brain-specific angiogenesis inhibitor 1-associated protein 2-lik	BAIAP2L1	1	2	2	4.3	56.882	NaN	NaN	NaN	NaN	NaN	NaN	NaN	NaN
O75531	Barrier-to-autointegration factor;Barrier-to-autointegration fa	BANF1	1	1	1	27	10.058	NaN	NaN	NaN	NaN	NaN	NaN	NaN	NaN
P80723	Brain acid soluble protein 1	BASP1	1	4	4	32.2	22.693	NaN	2.7E+07	NaN	1.6E+07	1.5E+07	2.8E+07	0.73511	NaN
Q07812	Apoptosis regulator BAX	BAX	1	2	2	17.2	21.184	3.4E+07	3.9E+07	4.8E+07	2.8E+07	3.5E+07	4.2E+07	0.86804	0.39404
P50895	Basal cell adhesion molecule	BCAM	1	2	2	4.5	67.404	NaN	1.4E+07	1.5E+07	1.9E+07	1.5E+07	1.9E+07	1.17461	0.19503
P51572	B-cell receptor-associated protein 31	BCAP31	1	6	6	11.8	27.991	2.6E+08	2.1E+08	2.2E+08	3.1E+08	2.4E+08	2.8E+08	1.21178	0.11059
Q9P287	BRCA2 and CDKN1A-interacting protein	BCCIP	1	1	1	5.1	35.979	NaN	NaN	NaN	NaN	NaN	NaN	NaN	NaN
Q8TD16;Q96G01	Protein bicaudal D homolog 2;Protein bicaudal D homolog 1	BICD2	2	1	1	1.1	93.532	NaN	NaN	NaN	NaN	NaN	NaN	NaN	NaN
P78537	Biogenesis of lysosome-related organelles complex 1 subunit 1	BLOC1S1	1	1	1	6.5	17.262	NaN	NaN	NaN	NaN	NaN	NaN	NaN	NaN
P53004	Biliverdin reductase A	BLVRA	1	2	2	7.4	33.428	NaN	NaN	NaN	NaN	NaN	NaN	NaN	NaN
P30043	Flavin reductase (NADPH)	BLVRB	1	2	2	8.3	22.119	6.6E+07	1.1E+08	NaN	NaN	NaN	NaN	NaN	NaN
Q96IK1;Q8NFC6	Biorientation of chromosomes in cell division protein 1;Biorient	BOD1	2	1	1	8.6	19.196	NaN	NaN	NaN	NaN	NaN	NaN	NaN	NaN
Q14137	Ribosome biogenesis protein BOP1	BOP1	1	1	1	1.5	83.629	NaN	NaN	NaN	NaN	NaN	NaN	NaN	NaN
Q8WY22	BRI3-binding protein	BRI3BP	1	1	1	6.8	27.835	NaN	NaN	NaN	NaN	NaN	NaN	NaN	NaN
Q8WUW1	Protein BRICK1	BRK1	1	1	1	10.7	8.7448	NaN	NaN	NaN	NaN	NaN	NaN	NaN	NaN
Q5VW32	BRO1 domain-containing protein BROX	BROX	1	1	1	2.7	46.476	NaN	NaN	NaN	NaN	NaN	NaN	NaN	NaN
P35613	Basigin	BSG	1	2	2	4.7	42.2	NaN	NaN	NaN	NaN	NaN	NaN	NaN	NaN
O14981	TATA-binding protein-associated factor 172	BTAF1	1	1	1	0.8	206.89	NaN	NaN	NaN	NaN	NaN	NaN	NaN	NaN
P20290	Transcription factor BTF3	BTF3	1	3	3	21.8	22.168	NaN	7926800	6572900	NaN	8987100	NaN	1.23963	NaN
Q96K17	Transcription factor BTF3 homolog 4	BTF3L4	1	2	2	19	17.27	8132400	NaN	NaN	7510300	8659300	6589400	0.93285	NaN
Q7L1Q6	Basic leucine zipper and W2 domain-containing protein 1	BZW1	1	2	2	3.8	48.043	2.5E+07	3.7E+07	NaN	6.2E+07	9.6E+07	4.1E+07	2.12472	0.19773
Q9Y6E2	Basic leucine zipper and W2 domain-containing protein 2	BZW2	1	2	2	4.8	48.162	NaN	NaN	NaN	3.3E+07	3.3E+07	NaN	NaN	NaN
E9PRG8	Uncharacterized protein C11orf98	C11orf98	1	1	1	10.6	14.234	NaN	NaN	NaN	NaN	NaN	NaN	NaN	NaN
Q9HB07	UPF0160 protein MYG1, mitochondrial	C12orf10	1	2	2	6.9	42.477	NaN	NaN	NaN	NaN	NaN	NaN	NaN	NaN
Q8N999	Uncharacterized protein C12orf29	C12orf29	1	1	1	3.1	37.49	NaN	NaN	NaN	NaN	NaN	NaN	NaN	NaN
Q9UKR5	Probable ergosterol biosynthetic protein 28	C14orf1	1	1	1	6.4	15.864	NaN	NaN	NaN	NaN	NaN	NaN	NaN	NaN
Q9Y224	UPF0568 protein C14orf166	C14orf166	1	2	2	7	28.068	6E+07	7.3E+07	NaN	5.3E+07	5.8E+07	6.4E+07	0.87942	0.30593
Q6ZSJ8	Uncharacterized protein C1orf122	C1orf122	1	1	1	11.8	11.471	NaN	NaN	NaN	NaN	NaN	NaN	NaN	NaN
Q8N1A6	UPF0462 protein C4orf33	C4orf33	1	1	1	5.5	23.467	NaN	NaN	NaN	NaN	NaN	NaN	NaN	NaN
Q9BRJ6	Uncharacterized protein C7orf50	C7orf50	1	1	1	10.8	22.083	NaN	NaN	NaN	NaN	NaN	NaN	NaN	NaN
Q9H7E9	UPF0488 protein C8orf33	C8orf33	1	2	2	10.5	24.992	NaN	NaN	NaN	NaN	NaN	NaN	NaN	NaN
Q5T6V5	UPF0553 protein C9orf64	C9orf64	1	2	2	6.7	39.028	5.3E+07	4.2E+07	NaN	3.4E+07	4.2E+07	4.8E+07	0.87515	0.42084
Q9HB71	Calcyclin-binding protein	CACYBP	1	3	3	18	26.21	8.4E+07	8.8E+07	9.1E+07	8.2E+07	8.7E+07	8.9E+07	0.98258	0.64264
P27708	CAD protein;Glutamine-dependent carbamoyl-phosphate synt	CAD	1	14	14	7.2	242.98	6.3E+07	6.5E+07	6.4E+07	7.7E+07	6.3E+07	5.9E+07	1.03032	0.73205
Q05682	Caldesmon	CALD1	1	6	6	9.2	93.23	7.4E+07	7.4E+07	6.9E+07	6.1E+07	6.5E+07	6.9E+07	0.90087	0.05672
P27797	Calreticulin	CALR	1	8	8	28.3	48.141	4.1E+08	7E+08	5.7E+08	4.5E+08	5.3E+08	3.2E+08	0.7734	0.29262
O43852	Calumenin	CALU	1	4	4	11.7	37.106	5.9E+07	5.6E+07	7.8E+07	5.6E+07	5.4E+07	6.3E+07	0.89846	0.42845
Q13557	Calcium/calmodulin-dependent protein kinase type II subunit	CAMK2D	1	1	1	2	56.369	NaN	NaN	NaN	NaN	NaN	NaN	NaN	NaN
Q86VP6	Cullin-associated NEDD8-dissociated protein 1	CAND1	2	9	9	6.9	136.37	7.9E+07	8.5E+07	7.2E+07	7.5E+07	8.4E+07	7.8E+07	1.00266	0.96714
P27824	Calnexin	CANX	1	9	9	16.4	67.567	4.2E+08	3.9E+08	3.5E+08	3.8E+08	4.1E+08	3.9E+08	1.01308	0.8362
Q01518	Adenylyl cyclase-associated protein 1	CAP1	1	3	3	8.6	51.901	1.9E+08	NaN	1E+08	2.1E+08	3.6E+08	1.5E+08	1.64605	0.36319
P07384	Calpain-1 catalytic subunit	CAPN1	1	3	3	4.1	81.889	5.6E+07	5.4E+07	5.7E+07	5.2E+07	5.3E+07	3.3E+07	0.82696	0.22124
P17655	Calpain-2 catalytic subunit	CAPN2	1	7	7	10.9	79.994	2.6E+08	3.3E+08	2.7E+08	2.8E+08	3.1E+08	2.4E+08	0.96171	0.74692
Q96L46;P04632	Calpain small subunit 2;Calpain small subunit 1	CAPNS2	2	1	1	2.8	27.66	NaN	NaN	NaN	NaN	NaN	NaN	NaN	NaN
Q14444	Caprin-1	CAPRIN1	1	4	4	5.9	78.365	1.4E+08	1.9E+08	1.2E+08	1.3E+08	9.4E+07	1.6E+08	0.85247	0.46831
P47755	F-actin-capping protein subunit alpha-2	CAPZA2	2	4	4	17.5	32.949	4.8E+07	5.9E+07	5.2E+07	NaN	5.1E+07	4.7E+07	0.92461	0.42228
P47756	F-actin-capping protein subunit beta	CAPZB	1	7	7	29	30.628	1.4E+08	1.5E+08	1.4E+08	1E+08	1.4E+08	1.4E+08	0.88339	0.27313
P49589	Cysteine--tRNA ligase, cytoplasmic	CARS	1	5	5	7.2	85.472	3.7E+07	3.5E+07	3.9E+07	3.5E+07	4.7E+07	5.1E+07	1.19594	0.2264
Q9HA77	Probable cysteine--tRNA ligase, mitochondrial	CARS2	1	1	1	3.2	62.223	NaN	NaN	NaN	NaN	NaN	NaN	NaN	NaN
Q14790	Caspase-8;Caspase-8 subunit p18;Caspase-8 subunit p10	CASP8	1	2	2	4.4	55.391	NaN	NaN	NaN	NaN	NaN	NaN	NaN	NaN
P20810	Calpastatin	CAST	1	4	4	5.9	76.572	6.6E+07	7.5E+07	7.4E+07	7.6E+07	6.2E+07	5.6E+07	0.89736	0.31779
P04040	Catalase	CAT	1	2	2	4	59.755	NaN	NaN	NaN	NaN	NaN	NaN	NaN	NaN
Q03135	Caveolin-1	CAV1	2	3	3	21.9	20.471	NaN	NaN	NaN	NaN	NaN	NaN	NaN	NaN
P51636	Caveolin-2	CAV2	1	1	1	7.4	18.291	3.1E+07	NaN	3.8E+07	NaN	NaN	NaN	NaN	NaN
P83916	Chromobox protein homolog 1	CBX1	1	1	1	9.2	21.418	NaN	NaN	NaN	NaN	NaN	NaN	NaN	NaN

Q13185	Chromobox protein homolog 3	CBX3	1	1	1	7.7	20.811	NaN	NaN	NaN	NaN	NaN	NaN	NaN	NaN
P45973	Chromobox protein homolog 5	CBX5	1	2	2	13.6	22.225	NaN	NaN	NaN	NaN	NaN	NaN	NaN	NaN
Q5T0F9	Coiled-coil and C2 domain-containing protein 1B	CC2D1B	1	1	1	1.4	94.223	NaN	NaN	NaN	NaN	NaN	NaN	NaN	NaN
Q96CT7	Coiled-coil domain-containing protein 124	CCDC124	1	1	1	4	25.835	NaN	NaN	NaN	NaN	NaN	NaN	NaN	NaN
O60826	Coiled-coil domain-containing protein 22	CCDC22	1	1	1	2.2	70.755	NaN	NaN	NaN	NaN	NaN	NaN	NaN	NaN
Q86WR0	Coiled-coil domain-containing protein 25	CCDC25	1	1	1	6.2	24.479	NaN	NaN	NaN	NaN	NaN	NaN	NaN	NaN
Q9Y3C0	WASH complex subunit CCDC53	CCDC53	1	2	2	20.1	21.172	NaN	NaN	NaN	NaN	NaN	NaN	NaN	NaN
Q16204	Coiled-coil domain-containing protein 6	CCDC6	1	6	6	13.5	53.29	3.1E+07	3.5E+07	2.8E+07	3.4E+07	3E+07	3E+07	1.00349	0.96766
O14618	Copper chaperone for superoxide dismutase	CCS	1	1	1	4.7	29.04	NaN	NaN	NaN	NaN	NaN	NaN	NaN	NaN
P78371	T-complex protein 1 subunit beta	CCT2	1	4	4	8.6	57.488	1.1E+08	1.1E+08	1.1E+08	6.8E+07	9.8E+07	1E+08	0.8128	0.14557
P49368	T-complex protein 1 subunit gamma	CCT3	1	8	8	14.3	60.533	1.2E+08	1.3E+08	1.7E+08	2.1E+08	1.7E+08	1.8E+08	1.35202	0.06182
P50991	T-complex protein 1 subunit delta	CCT4	1	2	2	3.7	57.924	2.3E+08	1.8E+08	1.7E+08	1.8E+08	1.7E+08	1.9E+08	0.9149	0.46851
P48643	T-complex protein 1 subunit epsilon	CCT5	1	4	4	6.8	59.67	5.9E+07	6E+07	NaN	5.2E+07	5.4E+07	6.8E+07	0.97672	0.83937
P40227	T-complex protein 1 subunit zeta	CCT6A	2	4	4	6.8	58.024	1.5E+08	1.4E+08	1.3E+08	1.2E+08	1.3E+08	1.4E+08	0.9117	0.24009
Q99832	T-complex protein 1 subunit eta	CCT7	1	7	7	11.8	59.366	9.4E+07	9.9E+07	8.6E+07	9.5E+07	8.2E+07	8.2E+07	0.92751	0.31368
P50990	T-complex protein 1 subunit theta	CCT8	1	9	9	19	59.62	3.9E+08	3.2E+08	3.9E+08	3.1E+08	3.5E+08	4.1E+08	0.98021	0.85607
P86791;P86790	Vacuolar fusion protein CCZ1 homolog;Vacuolar fusion proteir	CCZ1	2	2	2	5.2	55.866	NaN	NaN	NaN	NaN	NaN	NaN	NaN	NaN
Q9Y5K6	CD2-associated protein	CD2AP	1	1	1	2.2	71.45	NaN	NaN	NaN	NaN	NaN	NaN	NaN	NaN
P28907	ADP-ribosyl cyclase/cyclic ADP-ribose hydrolase 1	CD38	1	2	2	8.3	34.328	2.8E+07	3.3E+07	3.2E+07	3.5E+07	3.3E+07	3E+07	1.04942	0.50576
O15446	DNA-directed RNA polymerase I subunit RPA34	CD3EAP	1	1	1	4.7	54.985	NaN	NaN	NaN	NaN	NaN	NaN	NaN	NaN
P16070	CD44 antigen	CD44	1	1	1	1.3	81.537	NaN	NaN	NaN	NaN	NaN	NaN	NaN	NaN
P08962	CD63 antigen	CD63	1	2	2	7.6	25.636	1.6E+08	1.7E+08	1.9E+08	1.9E+08	1.8E+08	1.9E+08	1.05844	0.34116
P32970	CD70 antigen	CD70	1	2	2	10.9	21.118	NaN	NaN	5.8E+07	5.6E+07	5.6E+07	NaN	0.9591	NaN
P14209	CD99 antigen	CD99	1	2	2	32.4	18.848	NaN	1.2E+07	1.7E+07	NaN	NaN	NaN	NaN	NaN
Q8TCZ2	CD99 antigen-like protein 2	CD99L2	1	1	1	5.7	27.986	NaN	NaN	NaN	NaN	NaN	NaN	NaN	NaN
Q13042	Cell division cycle protein 16 homolog	CDC16	1	1	1	1.6	71.655	NaN	NaN	NaN	NaN	NaN	NaN	NaN	NaN
Q8NHZ8	Anaphase-promoting complex subunit CDC26	CDC26	1	2	2	14.1	9.7769	NaN	NaN	NaN	NaN	NaN	NaN	NaN	NaN
Q16543	Hsp90 co-chaperone Cdc37;Hsp90 co-chaperone Cdc37, N-te	CDC37	1	2	2	8.2	44.468	5.9E+07	6.4E+07	6.4E+07	8.1E+07	4.7E+07	5.4E+07	0.9819	0.91994
Q9Y5S2	Serine/threonine-protein kinase MRCK beta	CDC42BPB	1	2	2	1.4	194.31	NaN	NaN	NaN	NaN	NaN	NaN	NaN	NaN
P12830	Cadherin-1;E-Cad/CTF1;E-Cad/CTF2;E-Cad/CTF3	CDH1	1	1	1	1.8	97.455	NaN	NaN	NaN	NaN	NaN	NaN	NaN	NaN
P06493	Cyclin-dependent kinase 1	CDK1	1	4	3	13.5	34.095	3.4E+07	3.1E+07	2.9E+07	NaN	NaN	3.4E+07	1.09576	NaN
P24941	Cyclin-dependent kinase 2	CDK2	1	2	1	7.4	33.929	NaN	NaN	NaN	NaN	NaN	NaN	NaN	NaN
Q00535	Cyclin-dependent-like kinase 5	CDK5	1	5	4	13	33.304	4.8E+07	6.4E+07	NaN	4.1E+07	NaN	5.4E+07	0.84772	0.48543
Q16878	Cysteine dioxygenase type 1	CDO1	1	1	1	3.5	22.972	NaN	NaN	NaN	NaN	NaN	NaN	NaN	NaN
O95674;Q92903	Phosphatidate cytidylyltransferase 2;Phosphatidate cytidylyltrar	CDS2	2	2	2	7.6	51.417	NaN	NaN	NaN	NaN	NaN	NaN	NaN	NaN
P41208	Centrin-2	CETN2	1	1	1	7	19.738	NaN	NaN	NaN	NaN	NaN	NaN	NaN	NaN
Q9Y6A4	Cilia- and flagella-associated protein 20	CFAP20	1	1	1	6.2	22.774	NaN	NaN	NaN	NaN	NaN	NaN	NaN	NaN
P23528	Cofilin-1	CFL1	1	3	3	20.5	18.502	3.6E+08	2.5E+08	7.7E+08	3E+08	3.5E+08	2.6E+08	0.65648	0.38261
Q9NX63	MICOS complex subunit MIC19	CHCHD3	1	4	4	18.9	26.152	5.5E+07	5.9E+07	NaN	NaN	5.5E+07	NaN	0.95991	NaN
Q9BWS9	Chitinase domain-containing protein 1	CHID1	1	1	1	3.6	44.94	NaN	NaN	NaN	NaN	NaN	NaN	NaN	NaN
Q9NZZ3	Charged multivesicular body protein 5	CHMP5	1	1	1	7.3	24.57	NaN	NaN	NaN	NaN	NaN	NaN	NaN	NaN
Q8WUX9	Charged multivesicular body protein 7	CHMP7	1	1	1	2.2	50.91	NaN	NaN	NaN	NaN	NaN	NaN	NaN	NaN
Q9UHD1	Cysteine and histidine-rich domain-containing protein 1	CHORDC1	1	2	2	8.7	37.489	NaN	NaN	NaN	NaN	NaN	NaN	NaN	NaN
Q99653	Calcineurin B homologous protein 1	CHP1	1	2	2	10.3	22.456	NaN	NaN	NaN	NaN	NaN	NaN	NaN	NaN
O76071	Probable cytosolic iron-sulfur protein assembly protein CIAO1	CIAO1	1	1	1	3.5	37.84	NaN	NaN	NaN	NaN	NaN	NaN	NaN	NaN
Q6FI81	Anamorsin	CIAPIN1	1	2	2	5.1	33.582	NaN	NaN	NaN	NaN	NaN	NaN	NaN	NaN
Q9NZ45	CDGSH iron-sulfur domain-containing protein 1	CISD1	1	1	1	13.9	12.199	NaN	NaN	NaN	NaN	NaN	NaN	NaN	NaN
Q8N5K1	CDGSH iron-sulfur domain-containing protein 2	CISD2	1	1	1	11.1	15.278	NaN	NaN	NaN	NaN	NaN	NaN	NaN	NaN
Q07065	Cytoskeleton-associated protein 4	CKAP4	1	5	5	8.1	66.022	1E+08	9.6E+07	9.5E+07	1.4E+08	1.2E+08	1.2E+08	1.27232	0.0168
Q14008	Cytoskeleton-associated protein 5	CKAP5	1	11	11	6.6	225.49	4.2E+07	5.3E+07	5E+07	3.9E+07	4.2E+07	4.5E+07	0.87251	0.18794
O95832	Claudin-1	CLDN1	1	1	1	7.1	22.744	NaN	NaN	NaN	NaN	NaN	NaN	NaN	NaN
O00299	Chloride intracellular channel protein 1	CLIC1	1	6	6	41.5	26.922	2E+08	2.7E+08	2.3E+08	1.9E+08	2.5E+08	1.4E+08	0.84168	0.40383
O95833	Chloride intracellular channel protein 3	CLIC3	1	1	1	5.9	26.648	NaN	NaN	NaN	NaN	NaN	NaN	NaN	NaN
Q9Y696	Chloride intracellular channel protein 4	CLIC4	1	5	5	29.2	28.772	3.9E+07	5.1E+07	4.9E+07	5.1E+07	4.9E+07	4.1E+07	1.01422	0.89512
Q14677	Clathrin interactor 1	CLINT1	1	1	1	3.2	68.259	NaN	NaN	NaN	NaN	NaN	NaN	NaN	NaN
P30622	CAP-Gly domain-containing linker protein 1	CLIP1	2	7	7	5.4	162.24	2.7E+07	2.6E+07	2.5E+07	2E+07	2.3E+07	2.4E+07	0.84953	0.05164
Q9H078	Caseinolytic peptidase B protein homolog	CLPB	1	3	3	5.2	78.728	NaN	NaN	NaN	NaN	1.3E+07	1.2E+07	NaN	NaN
O96005	Cleft lip and palate transmembrane protein 1	CLPTM1	1	5	5	6	76.096	4.2E+07	5.1E+07	4.3E+07	4.3E+07	4.2E+07	5E+07	0.98984	0.91326
P09496	Clathrin light chain A	CLTA	1	3	3	10.1	27.076	1E+08	1.4E+08	1.2E+08	7.8E+07	8.7E+07	9.4E+07	0.72395	0.04264

Q00610	Clathrin heavy chain 1	CLTC	2	13	13	6.9	191.61	4.7E+08	4.7E+08	4.7E+08	3.8E+08	4.8E+08	3.7E+08	0.87485	0.17534
P10909	Clusterin;Clusterin beta chain;Clusterin alpha chain	CLU	1	2	2	4.7	52.494	NaN	NaN	NaN	NaN	NaN	NaN	NaN	NaN
O75153	Clustered mitochondria protein homolog	CLUH	2	5	5	5.4	146.67	1.6E+07	1.9E+07	1.5E+07	1.3E+07	1.4E+07	1.8E+07	0.91983	0.49045
Q8NFW8	N-acylneuraminate cytidylyltransferase	CMAS	1	1	1	2.8	48.379	NaN	NaN	NaN	NaN	NaN	NaN	NaN	NaN
Q96DG6	Carboxymethylenebutenolidase homolog	CMBL	1	2	2	11.8	28.048	NaN	NaN	NaN	NaN	NaN	NaN	NaN	NaN
P30085	UMP-CMP kinase	CMPK1	1	2	2	6.6	22.222	9E+07	7.9E+07	NaN	6.7E+07	5.4E+07	NaN	0.71545	0.10934
Q96KP4	Cytosolic non-specific dipeptidase	CNDP2	1	5	5	14.7	52.878	7.8E+07	8.7E+07	7.7E+07	4.1E+07	6.7E+07	8.1E+07	0.78167	0.21666
Q99439	Calponin-2	CNN2	1	1	1	3.6	33.697	NaN	NaN	NaN	NaN	NaN	NaN	NaN	NaN
A5YKK6	CCR4-NOT transcription complex subunit 1	CNOT1	1	5	5	2.5	266.94	1.5E+07	2.1E+07	1.6E+07	1.6E+07	1.7E+07	NaN	0.9269	0.63077
O75175	CCR4-NOT transcription complex subunit 3	CNOT3	1	1	1	1.9	81.871	NaN	NaN	NaN	NaN	NaN	NaN	NaN	NaN
P09543	2,3-cyclic-nucleotide 3-phosphodiesterase	CNP	1	3	3	6.7	47.578	1.3E+07	1.2E+07	1.3E+07	NaN	1.4E+07	1.3E+07	1.09172	0.24184
Q9Y2B0	Protein canopy homolog 2	CNPY2	1	4	4	24.7	20.652	5.6E+07	6E+07	6.6E+07	4.5E+07	5.1E+07	4.6E+07	0.77982	0.01753
Q12860	Contactin-1	CNTN1	1	1	1	1	113.32	NaN	NaN	NaN	NaN	NaN	NaN	NaN	NaN
Q9Y2R0	Cytochrome c oxidase assembly factor 3 homolog, mitochondı	COA3	1	2	2	18.9	11.731	NaN	NaN	NaN	NaN	NaN	NaN	NaN	NaN
Q5JTJ3	Cytochrome c oxidase assembly factor 6 homolog	COA6	1	1	1	10.4	14.116	NaN	NaN	NaN	NaN	NaN	NaN	NaN	NaN
P12111	Collagen alpha-3(VI) chain	COL6A3	1	1	1	0.4	343.67	NaN	NaN	NaN	NaN	NaN	NaN	NaN	NaN
Q8NBJ5	Procollagen galactosyltransferase 1	COLGALT1	1	2	2	3.4	71.635	NaN	8494800	2.2E+07	5917000	NaN	NaN	0.38768	NaN
P53621	Coatomer subunit alpha;Xenin;Proxenin	COPA	1	5	5	3.4	138.34	9.6E+07	1.1E+08	9.3E+07	9.5E+07	1.2E+08	9E+07	1.01964	0.84343
P53618	Coatomer subunit beta	COPB1	1	9	9	8.8	107.14	1.7E+08	1.4E+08	1.3E+08	1.7E+08	1.8E+08	1.4E+08	1.14397	0.27596
P35606	Coatomer subunit beta	COPB2	1	1	1	1.4	102.49	NaN	NaN	NaN	NaN	NaN	NaN	NaN	NaN
O14579	Coatomer subunit epsilon	COPE	1	1	1	4.5	34.482	NaN	NaN	NaN	NaN	NaN	NaN	NaN	NaN
Q9Y678	Coatomer subunit gamma-1	COPG1	1	4	4	4.9	97.717	8.8E+07	8E+07	8.4E+07	6E+07	8.2E+07	8.8E+07	0.91181	0.4371
P61201	COP9 signalosome complex subunit 2	COPS2	1	3	3	7.2	51.596	7.7E+07	NaN	4.7E+07	9.1E+07	NaN	7.8E+07	1.36269	0.3005
Q92905	COP9 signalosome complex subunit 5	COPS5	1	1	1	5.7	37.578	NaN	NaN	NaN	NaN	NaN	NaN	NaN	NaN
Q7L5N1	COP9 signalosome complex subunit 6	COPS6	1	1	1	4.6	36.163	NaN	NaN	6.6E+07	1.4E+07	5.1E+07	3.4E+07	0.50057	NaN
Q9BR76	Coronin-1B	CORO1B	1	2	2	3.1	54.234	2.3E+07	2E+07	2.7E+07	2.9E+07	2.9E+07	2.7E+07	1.208	0.06967
Q9ULV4	Coronin-1C	CORO1C	1	1	1	2.5	53.248	NaN	NaN	NaN	NaN	NaN	NaN	NaN	NaN
Q14019	Coactosin-like protein	COTL1	1	3	3	12.7	15.945	3E+08	2.9E+08	3.2E+08	2.2E+08	2.4E+08	3.1E+08	0.8382	0.14511
P13073	Cytochrome c oxidase subunit 4 isoform 1, mitochondrial	COX4I1	1	2	2	13	19.576	2.5E+07	NaN	1.8E+07	NaN	1.8E+07	1.6E+07	0.7821	0.32435
P20674	Cytochrome c oxidase subunit 5A, mitochondrial	COX5A	1	1	1	6	16.762	NaN	NaN	NaN	NaN	NaN	NaN	NaN	NaN
P09669	Cytochrome c oxidase subunit 6C	COX6C	1	2	2	13.3	8.7813	NaN	NaN	NaN	NaN	NaN	NaN	NaN	NaN
P14406	Cytochrome c oxidase subunit 7A2, mitochondrial	COX7A2	1	1	1	15.7	9.3959	NaN	NaN	NaN	NaN	NaN	NaN	NaN	NaN
O75976	Carboxypeptidase D	CPD	1	2	2	2.4	152.93	NaN	NaN	NaN	NaN	NaN	NaN	NaN	NaN
Q6PUV4	Complexin-2	CPLX2	1	3	3	29.1	15.394	5.3E+07	5.3E+07	4.6E+07	5E+07	4.8E+07	5E+07	0.9697	0.53939
Q96FN4	Copine-2	CPNE2	1	3	2	6.8	61.189	NaN	NaN	8245400	6638800	7964600	NaN	0.88555	NaN
O75131	Copine-3	CPNE3	7	4	3	7.3	60.13	8.1E+07	NaN	3.3E+07	5.6E+07	1.2E+08	NaN	1.55171	0.52033
P36551	Oxygen-dependent coproporphyrinogen-III oxidase, mitochor	CPOX	1	2	2	5.3	50.151	NaN	NaN	NaN	NaN	NaN	NaN	NaN	NaN
Q9BRF8	Serine/threonine-protein phosphatase CPPED1	CPPED1	1	2	2	8.3	35.548	NaN	2.7E+07	2.4E+07	2.9E+07	2.9E+07	2.6E+07	1.11038	0.2051
P31327	Carbamoyl-phosphate synthase [ammonia], mitochondrial	CPS1	1	4	4	3.5	164.94	2.5E+07	2.4E+07	2.3E+07	2.3E+07	3.6E+07	3.5E+07	1.31359	0.14607
Q16630	Cleavage and polyadenylation specificity factor subunit 6	CPSF6	1	2	2	5.4	59.209	NaN	NaN	NaN	NaN	NaN	NaN	NaN	NaN
Q8N684	Cleavage and polyadenylation specificity factor subunit 7	CPSF7	1	1	1	4	52.049	NaN	NaN	NaN	NaN	NaN	NaN	NaN	NaN
P50416	Carnitine O-palmitoyltransferase 1, liver isoform	CPT1A	1	5	5	7.1	88.367	2.1E+07	2E+07	1.7E+07	2E+07	1.4E+07	2.4E+07	0.9783	0.89579
P29373	Cellular retinoic acid-binding protein 2	CRABP2	1	1	1	6.5	15.693	NaN	NaN	NaN	NaN	NaN	NaN	NaN	NaN
P43155	Carnitine O-acetyltransferase	CRAT	1	1	1	2.4	70.857	NaN	NaN	NaN	NaN	NaN	NaN	NaN	NaN
P50238	Cysteine-rich protein 1	CRIP1	1	2	2	19.5	8.5328	8.3E+07	7.8E+07	9.2E+07	9.3E+07	7.9E+07	7.4E+07	0.97149	0.752
Q9BZJ0	Crooked neck-like protein 1	CRNKL1	1	1	1	1.4	100.45	NaN	NaN	NaN	NaN	NaN	NaN	NaN	NaN
Q08257	Quinone oxidoreductase	CRYZ	1	3	3	8.5	35.206	6.6E+07	4.1E+07	6.6E+07	5.1E+07	6.3E+07	6.7E+07	1.04688	0.79447
O75390	Citrate synthase, mitochondrial	CS	1	5	5	9.2	51.712	1.5E+08	1.3E+08	1.7E+08	1.7E+08	1.6E+08	1.5E+08	1.07734	0.39973
P55060	Exportin-2	CSE1L	1	11	11	12.7	110.42	1.1E+08	1.5E+08	1.4E+08	1.1E+08	1.2E+08	1.1E+08	0.84787	0.14981
P48729	Casein kinase I isoform alpha	CSNK1A1	1	1	1	3.3	38.914	NaN	NaN	NaN	NaN	NaN	NaN	NaN	NaN
P68400	Casein kinase II subunit alpha	CSNK2A1	1	1	1	3.3	45.143	NaN	NaN	NaN	NaN	NaN	NaN	NaN	NaN
P19784	Casein kinase II subunit alpha	CSNK2A2	1	1	1	3.7	41.213	NaN	NaN	NaN	NaN	NaN	NaN	NaN	NaN
P67870	Casein kinase II subunit beta	CSNK2B	1	3	3	16.7	24.942	NaN	NaN	NaN	NaN	NaN	NaN	NaN	NaN
P21291	Cysteine and glycine-rich protein 1	CSRP1	1	1	1	8.8	20.567	1.8E+08	2E+08	NaN	1.6E+08	1.3E+08	1.6E+08	0.78668	0.05853
P04080	Cystatin-B	CSTB	1	1	1	12.2	11.139	1.6E+08	1.1E+08	1.9E+08	7.2E+07	2E+08	2E+08	1.03525	0.91806
P35221	Catenin alpha-1	CTNNA1	1	2	2	2.4	100.07	3.4E+07	5E+07	3.5E+07	2.8E+07	3E+07	NaN	0.73265	0.21797
P35222	Catenin beta-1	CTNNB1	1	1	1	1.5	85.496	NaN	NaN	NaN	NaN	NaN	NaN	NaN	NaN
Q8WYA6	Beta-catenin-like protein 1	CTNNBL1	1	2	2	2.8	65.173	NaN	NaN	NaN	NaN	NaN	NaN	NaN	NaN
O60716	Catenin delta-1	CTNND1	1	4	4	2.9	108.17	5.6E+07	4E+07	3.9E+07	2.8E+07	4.2E+07	3.8E+07	0.80289	0.28431

P17812	CTP synthase 1	CTPS1	1	8	8	13.9	66.69	1.3E+08	1.4E+08	1.5E+08	1.3E+08	1.6E+08	1.4E+08	1.02094	0.78754
P10619	Lysosomal protective protein;Lysosomal protective protein 32 I	CTSA	1	1	1	2.7	54.465	NaN	NaN	NaN	NaN	NaN	NaN	NaN	NaN
P07858	Cathepsin B;Cathepsin B light chain;Cathepsin B heavy chain	CTSB	1	2	2	8	37.821	NaN	NaN	NaN	NaN	NaN	NaN	NaN	NaN
P07339	Cathepsin D;Cathepsin D light chain;Cathepsin D heavy chain	CTSD	1	3	3	10.2	44.552	1.4E+08	1.1E+08	1.4E+08	1.1E+08	1.3E+08	1.5E+08	1.01346	0.91098
Q14247	Src substrate cortactin	CTTN	1	1	1	2.9	61.585	NaN	NaN	NaN	NaN	NaN	NaN	NaN	NaN
Q13618	Cullin-3	CUL3	1	3	3	4.9	88.929	5.7E+07	5.6E+07	6.1E+07	6.9E+07	6.3E+07	5.8E+07	1.0874	0.2408
Q13619	Cullin-4A	CUL4A	1	2	1	3	87.679	NaN	NaN	NaN	NaN	NaN	NaN	NaN	NaN
Q13620	Cullin-4B	CUL4B	1	4	3	5	103.98	4.3E+07	5.4E+07	4.9E+07	4.1E+07	4.9E+07	3.8E+07	0.88101	0.26109
Q93034	Cullin-5	CUL5	1	2	2	2.9	90.954	NaN	NaN	NaN	NaN	NaN	NaN	NaN	NaN
O60888	Protein CutA	CUTA	1	1	1	7.8	19.116	NaN	NaN	NaN	NaN	NaN	NaN	NaN	NaN
P42830	C-X-C motif chemokine 5;ENA-78(8-78);ENA-78(9-78)	CXCL5	1	1	1	10.5	11.972	NaN	NaN	NaN	NaN	NaN	NaN	NaN	NaN
P00167	Cytochrome b5	CYB5A	1	2	2	25.4	15.33	7.7E+07	8.1E+07	8.8E+07	9.3E+07	7.8E+07	8.1E+07	1.02347	0.75677
Q9UHQ9	NADH-cytochrome b5 reductase 1	CYB5R1	1	1	1	4.6	34.094	NaN	NaN	NaN	NaN	NaN	NaN	NaN	NaN
P00387	NADH-cytochrome b5 reductase 3;NADH-cytochrome b5 redu	CYB5R3	1	1	1	4	34.234	1.1E+08	1.5E+08	NaN	NaN	1.3E+08	1E+08	0.91646	0.70318
P13498	Cytochrome b-245 light chain	CYBA	1	2	2	12.8	21.012	NaN	NaN	NaN	NaN	NaN	NaN	NaN	NaN
P08574	Cytochrome c1, heme protein, mitochondrial	CYC1	1	2	2	3.4	35.422	NaN	NaN	NaN	NaN	NaN	NaN	NaN	NaN
Q7L576	Cytoplasmic FMR1-interacting protein 1	CYFIP1	2	6	6	5.7	145.18	3.7E+07	2.9E+07	3.6E+07	3.4E+07	3.5E+07	3.8E+07	1.04396	0.62829
Q07973	1,25-dihydroxyvitamin D(3) 24-hydroxylase, mitochondrial	CYP24A1	1	2	2	4.9	58.875	NaN	NaN	NaN	NaN	NaN	NaN	NaN	NaN
Q08477;P78329;Q9HCS	Docosahexaenoic acid omega-hydroxylase CYP4F3;Phylloquin	CYP4F3	5	2	2	3.1	59.846	NaN	1.2E+07	NaN	NaN	1.3E+07	1.5E+07	1.20187	NaN
Q16850	Lanosterol 14-alpha demethylase	CYP51A1	1	3	3	7.5	57.278	1.6E+07	2E+07	2.4E+07	1.9E+07	1.8E+07	1.8E+07	0.92203	0.56825
Q15438	Cytohesin-1	CYTH1	1	1	1	3.5	46.412	NaN	NaN	NaN	NaN	NaN	NaN	NaN	NaN
P61803	Dolichyl-diphosphooligosaccharide--protein glycosyltransfera	DAD1	1	2	2	19.5	12.497	4E+07	6.3E+07	6.8E+07	3E+07	8.5E+07	3.1E+07	0.85034	0.69304
Q14118	Dystroglycan;Alpha-dystroglycan;Beta-dystroglycan	DAG1	1	2	2	2.5	97.44	NaN	1.3E+07	NaN	1.3E+07	NaN	NaN	1.02843	NaN
Q3LXA3	Bifunctional ATP-dependent dihydroxyacetone kinase/FAD-AM	DAK	1	1	1	3.7	58.946	NaN	NaN	NaN	NaN	NaN	NaN	NaN	NaN
P14868	Aspartate--tRNA ligase, cytoplasmic	DARS	1	6	6	14.2	57.136	9.9E+07	9.1E+07	8.4E+07	9.6E+07	1E+08	9.4E+07	1.0713	0.26861
Q6PI48	Aspartate--tRNA ligase, mitochondrial	DARS2	1	1	1	1.6	73.562	NaN	NaN	NaN	NaN	NaN	NaN	NaN	NaN
Q96EP5	DAZ-associated protein 1	DAZAP1	1	1	1	3.9	43.383	NaN	NaN	NaN	NaN	NaN	NaN	NaN	NaN
Q96PD2	Discoidin, CUB and LCCL domain-containing protein 2	DCBLD2	1	1	1	1.5	85.034	NaN	NaN	NaN	NaN	NaN	NaN	NaN	NaN
P27707	Deoxycytidine kinase	DCK	1	2	2	10.8	30.518	NaN	NaN	NaN	NaN	NaN	NaN	NaN	NaN
Q96C86	m7GpppX diphosphatase	DCPS	1	1	1	3.3	38.608	NaN	NaN	NaN	NaN	NaN	NaN	NaN	NaN
Q14203	Dynactin subunit 1	DCTN1	1	4	4	4.1	141.69	3.4E+07	2.7E+07	NaN	NaN	2.9E+07	NaN	0.96355	NaN
O75935	Dynactin subunit 3	DCTN3	1	2	2	9.1	21.119	4.4E+07	4.4E+07	6E+07	3.4E+07	4.3E+07	5.9E+07	0.92489	0.70988
Q9H773	dCTP pyrophosphatase 1	DCTPP1	1	4	4	21.2	18.681	8.6E+07	8.9E+07	8.4E+07	8.8E+07	9.4E+07	6.7E+07	0.96758	0.76138
O95865	N(G),N(G)-dimethylarginine dimethylaminohydrolase 2	DDAH2	1	1	1	4.9	29.644	NaN	NaN	NaN	NaN	NaN	NaN	NaN	NaN
Q16531	DNA damage-binding protein 1	DDB1	1	7	7	6.1	126.97	6.8E+07	7.1E+07	6.6E+07	6E+07	6.6E+07	6.6E+07	0.93586	0.17094
P39656	Dolichyl-diphosphooligosaccharide--protein glycosyltransfera	DDOST	1	2	2	5	50.8	2.5E+07	2.2E+07	2.8E+07	2.2E+07	2.3E+07	NaN	0.89362	0.32712
Q96HY6	DDRGK domain-containing protein 1	DDRGK1	1	2	2	10.2	35.61	2.5E+07	1.7E+07	2.4E+07	2.3E+07	2.3E+07	2.4E+07	1.05454	0.65971
P30046;A6NHG4	D-dopachrome decarboxylase;D-dopachrome decarboxylase-	DDT	2	2	2	17.8	12.712	NaN	NaN	NaN	NaN	NaN	NaN	NaN	NaN
Q92499	ATP-dependent RNA helicase DDX1	DDX1	1	2	2	3.6	82.431	6.4E+07	6.7E+07	8.4E+07	4.9E+07	7.2E+07	7.7E+07	0.92268	0.63289
Q92841	Probable ATP-dependent RNA helicase DDX17	DDX17	1	4	2	5.9	80.272	7.3E+07	7.7E+07	7.4E+07	9E+07	6.6E+07	7.8E+07	1.04397	0.66588
Q9NVP1	ATP-dependent RNA helicase DDX18	DDX18	1	1	1	1.6	75.406	NaN	NaN	NaN	NaN	NaN	NaN	NaN	NaN
Q9NR30	Nucleolar RNA helicase 2	DDX21	1	1	1	1.4	87.343	NaN	NaN	NaN	NaN	NaN	NaN	NaN	NaN
Q9BUQ8	Probable ATP-dependent RNA helicase DDX23	DDX23	1	1	1	2.1	95.581	NaN	NaN	NaN	NaN	NaN	NaN	NaN	NaN
Q9GZR7	ATP-dependent RNA helicase DDX24	DDX24	1	1	1	1.5	96.331	NaN	NaN	NaN	NaN	NaN	NaN	NaN	NaN
Q13838;O00148	Spliceosome RNA helicase DDX39B;ATP-dependent RNA helic	DDX39B	2	3	3	7	48.991	3.6E+07	1.9E+07	3.4E+07	5.2E+07	4E+07	3.8E+07	1.46319	0.11791
O15523;O00571	ATP-dependent RNA helicase DDX3Y;ATP-dependent RNA he	DDX3Y	2	6	5	10	73.153	6.1E+07	6.8E+07	6E+07	7.7E+07	7.1E+07	6.9E+07	1.14072	0.06559
Q86XP3	ATP-dependent RNA helicase DDX42	DDX42	1	2	2	2.7	102.97	1.6E+07	2.5E+07	NaN	1.9E+07	1.9E+07	NaN	0.93046	0.78301
Q7L014	Probable ATP-dependent RNA helicase DDX46	DDX46	1	1	1	1.5	117.36	NaN	NaN	NaN	NaN	NaN	NaN	NaN	NaN
P17844	Probable ATP-dependent RNA helicase DDX5	DDX5	1	8	6	11.1	69.147	7.4E+07	6.4E+07	7.2E+07	8.5E+07	6.9E+07	7.6E+07	1.09382	0.30848
P26196	Probable ATP-dependent RNA helicase DDX6	DDX6	1	2	2	6	54.416	NaN	NaN	NaN	NaN	NaN	NaN	NaN	NaN
Q16698	2,4-dienoyl-CoA reductase, mitochondrial	DECR1	1	3	3	9.9	36.067	NaN	9E+07	9.1E+07	NaN	7.8E+07	8.7E+07	0.90976	0.23604
Q9NUI1	Peroxisomal 2,4-dienoyl-CoA reductase	DECR2	1	1	1	5.8	30.777	NaN	NaN	NaN	NaN	NaN	NaN	NaN	NaN
O15121	Sphingolipid delta(4)-desaturase DES1	DEGS1	1	1	1	4.6	37.866	NaN	NaN	NaN	NaN	NaN	NaN	NaN	NaN
P35659	Protein DEK	DEK	1	2	2	6.1	42.674	NaN	2.6E+07	2.1E+07	2.4E+07	1.9E+07	1.5E+07	0.82011	0.31986
O43583	Density-regulated protein	DENR	1	3	3	12.6	22.092	NaN	NaN	NaN	NaN	NaN	NaN	NaN	NaN
Q9Y315	Deoxyribose-phosphate aldolase	DERA	1	2	2	9.4	35.23	NaN	NaN	NaN	NaN	NaN	NaN	NaN	NaN
Q9BUN8	Derlin-1	DERL1	1	1	1	3.2	28.8	NaN	NaN	NaN	NaN	NaN	NaN	NaN	NaN
O00273	DNA fragmentation factor subunit alpha	DFFA	1	1	1	4.8	36.521	NaN	NaN	NaN	NaN	NaN	NaN	NaN	NaN
O60443	Non-syndromic hearing impairment protein 5	DFNA5	1	1	1	4.6	54.554	2.1E+07	NaN	NaN	2.6E+07	1.8E+07	NaN	1.03099	NaN

Q15392	Delta(24)-sterol reductase	DHCR24	1	3	3	5.8	60.101	3.4E+07	3.1E+07	3.3E+07	3.2E+07	3.1E+07	3.2E+07	0.97925	0.54203
Q9UBM7	7-dehydrocholesterol reductase	DHCR7	1	1	1	1.9	54.489	NaN	NaN	NaN	NaN	NaN	NaN	NaN	NaN
O43143	Pre-mRNA-splicing factor ATP-dependent RNA helicase DHX1	DHX15	1	7	7	8.8	90.932	4.7E+07	5E+07	4.9E+07	4.2E+07	4.6E+07	4.4E+07	0.90002	0.03051
Q7L2E3	Putative ATP-dependent RNA helicase DHX30	DHX30	1	2	2	1.7	133.94	NaN	NaN	NaN	NaN	NaN	NaN	NaN	NaN
Q9H2U1	ATP-dependent RNA helicase DHX36	DHX36	1	2	2	2.4	114.76	NaN	NaN	NaN	NaN	NaN	NaN	NaN	NaN
Q6P158	Putative ATP-dependent RNA helicase DHX57	DHX57	1	2	2	1.4	155.6	NaN	NaN	NaN	NaN	NaN	NaN	NaN	NaN
Q08211	ATP-dependent RNA helicase A	DHX9	1	6	6	5	140.96	5.8E+07	5.1E+07	5E+07	5.3E+07	5.3E+07	5.2E+07	0.98494	0.75075
Q9NR28	Diablo homolog, mitochondrial	DIABLO	1	1	1	7.1	27.131	NaN	NaN	NaN	NaN	NaN	NaN	NaN	NaN
O60610	Protein diaphanous homolog 1	DIAPH1	1	3	3	3.1	141.35	1.8E+07	2.3E+07	1.9E+07	2.5E+07	2E+07	1.8E+07	1.04504	0.73567
Q9UPY3	Endoribonuclease Dicer	DICER1	1	2	2	1.2	218.68	NaN	NaN	NaN	NaN	NaN	NaN	NaN	NaN
Q68CQ4	Digestive organ expansion factor homolog	DIEXF	1	1	1	2.2	87.054	NaN	NaN	NaN	NaN	NaN	NaN	NaN	NaN
Q9Y2L1	Exosome complex exonuclease RRP44	DIS3	1	1	1	1.8	109	9462100	1.2E+07	1E+07	9515500	1.2E+07	8810500	0.96972	0.79932
P10515	Dihydrolipoyllysine-residue acetyltransferase component of py	DLAT	1	2	2	3.4	68.996	1.6E+07	NaN	1.6E+07	NaN	NaN	1.8E+07	1.14151	NaN
P09622	Dihydrolipoyl dehydrogenase, mitochondrial	DLD	1	2	2	5.3	54.177	NaN	NaN	2.7E+07	NaN	3.7E+07	NaN	1.35459	NaN
P36957	Dihydrolipoyllysine-residue succinyltransferase component of :	DLST	1	3	3	8.2	48.755	2.1E+07	1.6E+07	1.3E+07	1.3E+07	1.6E+07	1.7E+07	0.91206	0.58333
Q86Y56	Dynein assembly factor 5, axonemal	DNAAF5	1	2	2	2.2	93.52	1.8E+07	2.2E+07	1.7E+07	1.9E+07	2E+07	NaN	1.03708	0.75577
P31689	DnaJ homolog subfamily A member 1	DNAJA1	1	6	6	17.4	44.868	8E+07	9.6E+07	1.1E+08	8.3E+07	1E+08	6.8E+07	0.87966	0.45144
O60884	DnaJ homolog subfamily A member 2	DNAJA2	1	5	5	11.2	45.745	1E+08	1.2E+08	9.1E+07	9.1E+07	1E+08	1E+08	0.92168	0.46928
Q96EY1	DnaJ homolog subfamily A member 3, mitochondrial	DNAJA3	1	2	2	5.8	52.488	NaN	1.9E+07	NaN	NaN	2.8E+07	NaN	1.4417	NaN
P25685	DnaJ homolog subfamily B member 1	DNAJB1	2	7	7	23.5	38.044	4.3E+07	6.4E+07	4.9E+07	6.9E+07	6.2E+07	6.2E+07	1.23278	0.13628
Q9UBS4	DnaJ homolog subfamily B member 11	DNAJB11	1	2	2	9.5	40.513	3.1E+07	NaN	4.4E+07	3.4E+07	3.5E+07	3.3E+07	0.91066	0.51976
Q8WWF6;Q7Z6W7;O75	DnaJ homolog subfamily B member 3;DnaJ homolog subfamily	DNAJB3	3	1	1	10.3	16.558	NaN	NaN	NaN	NaN	NaN	NaN	NaN	NaN
Q96KC8	DnaJ homolog subfamily C member 1	DNAJC1	1	1	1	2.7	63.882	NaN	NaN	NaN	NaN	NaN	NaN	NaN	NaN
Q8IXB1	DnaJ homolog subfamily C member 10	DNAJC10	1	1	1	1.3	91.079	NaN	NaN	NaN	NaN	NaN	NaN	NaN	NaN
Q9UKB3	DnaJ homolog subfamily C member 12	DNAJC12	1	1	1	7.1	23.415	NaN	NaN	NaN	NaN	NaN	NaN	NaN	NaN
O75165	DnaJ homolog subfamily C member 13	DNAJC13	1	2	2	1.4	254.41	NaN	NaN	NaN	NaN	NaN	NaN	NaN	NaN
Q99543	DnaJ homolog subfamily C member 2;DnaJ homolog subfamily	DNAJC2	1	2	2	3.9	71.996	NaN	NaN	NaN	NaN	NaN	NaN	NaN	NaN
Q9H3Z4	DnaJ homolog subfamily C member 5	DNAJC5	1	1	1	8.6	22.149	NaN	NaN	NaN	NaN	NaN	NaN	NaN	NaN
Q99615	DnaJ homolog subfamily C member 7	DNAJC7	1	2	2	4.7	56.44	NaN	NaN	NaN	1.1E+07	8431600	1.3E+07	NaN	NaN
O75937	DnaJ homolog subfamily C member 8	DNAJC8	1	1	1	6.3	29.841	4E+07	4.9E+07	6.2E+07	3.8E+07	5.1E+07	4.7E+07	0.90114	0.54225
Q8WXX5	DnaJ homolog subfamily C member 9	DNAJC9	1	2	2	8.8	29.909	NaN	NaN	NaN	NaN	NaN	NaN	NaN	NaN
Q05193	Dynamin-1	DNM1	1	5	3	6.5	97.407	2.4E+07	NaN	NaN	3E+07	NaN	NaN	1.24603	NaN
O00429	Dynamin-1-like protein	DNM1L	1	2	2	2.9	81.876	1.8E+07	NaN	1.9E+07	2.2E+07	2.3E+07	2.4E+07	1.22396	0.00433
P50570	Dynamin-2	DNM2	2	6	4	8.6	98.063	4E+07	3.4E+07	4.1E+07	5E+07	3.8E+07	4.5E+07	1.15461	0.21965
Q6XZF7	Dynamin-binding protein	DNMBP	1	2	2	1.3	177.35	NaN	NaN	NaN	NaN	NaN	NaN	NaN	NaN
P26358	DNA (cytosine-5)-methyltransferase 1	DNMT1	1	1	1	0.9	183.16	NaN	NaN	NaN	NaN	NaN	NaN	NaN	NaN
Q9H7D0	Dedicator of cytokinesis protein 5	DOCK5	1	1	1	0.7	215.31	NaN	NaN	NaN	NaN	NaN	NaN	NaN	NaN
Q9NY33	Dipeptidyl peptidase 3	DPP3	1	3	3	6	82.588	4.5E+07	4E+07	4.6E+07	4.6E+07	4.3E+07	5.1E+07	1.06961	0.37426
Q86TI2	Dipeptidyl peptidase 9	DPP9	1	1	1	2.1	98.262	NaN	NaN	NaN	NaN	NaN	NaN	NaN	NaN
Q16555	Dihydropyrimidinase-related protein 2	DPYSL2	2	7	7	15.9	62.293	5.8E+07	7.1E+07	6.9E+07	5E+07	6.2E+07	5.3E+07	0.84043	0.11782
Q9Y295	Developmentally-regulated GTP-binding protein 1	DRG1	1	3	3	10.6	40.542	3.4E+07	2.9E+07	3.6E+07	2.3E+07	2.8E+07	2.5E+07	0.76254	0.03763
P15924	Desmoplakin	DSP	1	2	2	1	331.77	NaN	NaN	NaN	NaN	NaN	NaN	NaN	NaN
Q03001	Dystonin	DST	1	2	2	0.4	860.65	NaN	NaN	NaN	NaN	NaN	NaN	NaN	NaN
P60981	Destrin	DSTN	1	7	7	33.9	18.506	4.1E+08	4.3E+08	4.4E+08	4.9E+08	3.8E+08	4.4E+08	1.02003	0.81164
Q9BVJ7	Dual specificity protein phosphatase 23	DUSP23	1	2	2	17.3	16.588	NaN	NaN	NaN	NaN	NaN	NaN	NaN	NaN
P33316	Deoxyuridine 5-triphosphate nucleotidohydrolase, mitochondr	DUT	1	2	2	9.9	26.563	2.2E+07	2.8E+07	2.6E+07	2.7E+07	3.1E+07	2.2E+07	1.04611	0.72739
Q14204	Cytoplasmic dynein 1 heavy chain 1	DYNC1H1	1	49	49	10.1	532.4	1.6E+08	1.6E+08	1.6E+08	1.5E+08	1.6E+08	1.7E+08	0.98059	0.60406
Q13409	Cytoplasmic dynein 1 intermediate chain 2	DYNC1I2	1	5	5	9.6	71.456	3.2E+07	3.3E+07	2.8E+07	3.2E+07	3.1E+07	3.2E+07	1.02606	0.64776
Q9Y6G9	Cytoplasmic dynein 1 light intermediate chain 1	DYNC1LI1	1	3	3	8.8	56.578	1.8E+07	1.3E+07	1.6E+07	1.6E+07	1.4E+07	1.7E+07	1.01122	0.92699
Q9NP97;Q8TF09	Dynein light chain roadblock-type 1;Dynein light chain roadblo	DYNLRB1	2	3	3	21.9	10.921	1.6E+08	NaN	1.5E+08	1.5E+08	NaN	1.5E+08	0.96211	0.34201
P42892	Endothelin-converting enzyme 1	ECE1	1	1	1	2.3	87.163	NaN	NaN	NaN	NaN	NaN	NaN	NaN	NaN
Q13011	Delta(3,5)-Delta(2,4)-dienoyl-CoA isomerase, mitochondrial	ECH1	1	1	1	4.3	35.816	NaN	NaN	NaN	NaN	NaN	NaN	NaN	NaN
Q9NTX5	Ethylmalonyl-CoA decarboxylase	ECHDC1	1	3	3	9.4	33.698	NaN	NaN	NaN	NaN	NaN	NaN	NaN	NaN
P30084	Enoyl-CoA hydratase, mitochondrial	ECHS1	1	2	2	9	31.387	NaN	NaN	NaN	NaN	NaN	NaN	NaN	NaN
P42126	Enoyl-CoA delta isomerase 1, mitochondrial	ECI1	1	1	1	3.3	32.816	NaN	NaN	NaN	NaN	NaN	NaN	NaN	NaN
O75521	Enoyl-CoA delta isomerase 2, mitochondrial	ECI2	1	1	1	2.3	43.585	NaN	NaN	NaN	NaN	NaN	NaN	NaN	NaN
Q5VYK3	Proteasome-associated protein ECM29 homolog	ECM29	1	3	3	2.3	204.29	2.9E+07	2.7E+07	2.5E+07	2.7E+07	2.7E+07	3E+07	1.02702	0.61731
Q6P2E9	Enhancer of mRNA-decapping protein 4	EDC4	1	2	2	1.5	151.66	NaN	NaN	NaN	NaN	NaN	NaN	NaN	NaN
Q5VTE0;P68104;Q0563	Putative elongation factor 1-alpha-like 3;Elongation factor 1-a	EEF1A1P5	3	5	5	10	50.184	8.9E+08	1.2E+09	1E+09	9.6E+08	1.1E+09	1.1E+09	1.00123	0.98858

P24534	Elongation factor 1-beta	EEF1B2	1	1	1	8	24.763	NaN	NaN	NaN	NaN	NaN	NaN	NaN	NaN
P29692	Elongation factor 1-delta	EEF1D	1	3	3	16.7	31.121	7.8E+07	5.9E+07	6E+07	8.4E+07	3E+07	6.3E+07	0.89889	0.7181
P26641	Elongation factor 1-gamma	EEF1G	1	8	8	15.6	50.118	3.7E+08	3.9E+08	4.1E+08	3.7E+08	4E+08	4E+08	0.99159	0.84174
P13639	Elongation factor 2	EEF2	2	17	17	16.7	95.337	8.4E+08	8.8E+08	9.3E+08	7.6E+08	9.2E+08	8.6E+08	0.96432	0.5929
Q96C19;Q9BUP0	EF-hand domain-containing protein D2;EF-hand domain-cont	EFHD2	2	2	2	10.8	26.697	NaN	NaN	NaN	NaN	NaN	NaN	NaN	NaN
P00533	Epidermal growth factor receptor	EGFR	1	3	3	3.1	134.28	2.5E+07	2E+07	2.1E+07	2.3E+07	NaN	2.4E+07	1.0861	0.43441
Q9H4M9	EH domain-containing protein 1	EHD1	1	8	6	17.2	60.626	1.7E+08	1.5E+08	1.5E+08	1.8E+08	1.7E+08	1.4E+08	1.03253	0.7073
Q9NZN4	EH domain-containing protein 2	EHD2	1	8	6	15.7	61.161	1.4E+08	1.6E+08	1.3E+08	1.1E+08	1.4E+08	1.3E+08	0.90887	0.35018
Q9NZN3	EH domain-containing protein 3	EHD3	1	5	2	10.1	60.886	NaN	1.7E+07	NaN	1.2E+07	1.1E+07	NaN	0.65304	NaN
Q9H223	EH domain-containing protein 4	EHD4	1	3	1	5.5	61.174	NaN	NaN	NaN	NaN	NaN	NaN	NaN	NaN
Q08426	Peroxisomal bifunctional enzyme;Enoyl-CoA hydratase/3,2-tra	EHHADH	1	1	1	2.1	79.494	NaN	NaN	NaN	NaN	NaN	NaN	NaN	NaN
Q8N9N8	Probable RNA-binding protein EIF1AD	EIF1AD	1	2	2	18.8	19.053	7376200	8019200	1E+07	7754300	6915100	NaN	0.85525	0.37716
P47813;O14602	Eukaryotic translation initiation factor 1A, X-chromosomal;Euka	EIF1AX	2	2	2	11.1	16.46	NaN	NaN	NaN	NaN	1E+08	1.2E+08	NaN	NaN
P19525	Interferon-induced, double-stranded RNA-activated protein k	EIF2AK2	1	4	4	9.1	62.094	6.2E+07	5.3E+07	6.1E+07	6.1E+07	3.9E+07	6.3E+07	0.92228	0.59792
Q14232	Translation initiation factor eIF-2B subunit alpha	EIF2B1	1	1	1	3.3	33.712	NaN	NaN	NaN	NaN	NaN	NaN	NaN	NaN
P05198	Eukaryotic translation initiation factor 2 subunit 1	EIF2S1	1	5	5	13.7	36.112	1.5E+08	1.7E+08	1.3E+08	1.1E+08	1.3E+08	1.5E+08	0.88239	0.32246
P20042	Eukaryotic translation initiation factor 2 subunit 2	EIF2S2	1	4	4	16.5	38.388	6E+07	8.8E+07	8.3E+07	7.8E+07	6.8E+07	7E+07	0.94159	0.6436
P41091;Q2VIR3	Eukaryotic translation initiation factor 2 subunit 3;Putative euka	EIF2S3	2	7	7	17.8	51.109	1.3E+08	1.2E+08	1.3E+08	1.1E+08	1.3E+08	1.4E+08	0.99504	0.95059
Q14152	Eukaryotic translation initiation factor 3 subunit A	EIF3A	1	21	21	12.9	166.57	1.8E+08	1.8E+08	1.6E+08	2.3E+08	1.9E+08	2.1E+08	1.18092	0.06947
P55884	Eukaryotic translation initiation factor 3 subunit B	EIF3B	1	2	2	2.2	92.48	6.3E+07	6.3E+07	6.4E+07	6.1E+07	7.1E+07	7E+07	1.0686	0.24505
Q99613;B5ME19	Eukaryotic translation initiation factor 3 subunit C;Eukaryotic tra	EIF3C	2	3	3	3.3	105.34	8.4E+07	8.6E+07	8.3E+07	9.5E+07	8.4E+07	9.2E+07	1.06969	0.13449
O15371	Eukaryotic translation initiation factor 3 subunit D	EIF3D	1	2	2	3.3	63.972	4.1E+07	NaN	NaN	4.8E+07	3.7E+07	4E+07	1.00794	NaN
P60228	Eukaryotic translation initiation factor 3 subunit E	EIF3E	1	6	6	13.3	52.22	7.2E+07	6.1E+07	9E+07	6.7E+07	7.2E+07	7.1E+07	0.93748	0.61552
O75821	Eukaryotic translation initiation factor 3 subunit G	EIF3G	1	2	2	6.9	35.611	NaN	NaN	NaN	NaN	NaN	NaN	NaN	NaN
O15372	Eukaryotic translation initiation factor 3 subunit H	EIF3H	1	3	3	9.1	39.93	3.2E+07	2.8E+07	3E+07	2.8E+07	3.5E+07	2.8E+07	1.01143	0.89967
Q13347	Eukaryotic translation initiation factor 3 subunit I	EIF3I	1	1	1	4	36.501	NaN	NaN	NaN	NaN	NaN	NaN	NaN	NaN
Q9Y262	Eukaryotic translation initiation factor 3 subunit L	EIF3L	1	5	5	6.9	66.726	6.7E+07	7.1E+07	6.4E+07	7E+07	7.4E+07	7.3E+07	1.07141	0.12166
P60842	Eukaryotic initiation factor 4A-I	EIF4A1	1	7	3	13.5	46.153	3.4E+08	3.6E+08	3.6E+08	3.4E+08	3.9E+08	3.6E+08	1.01737	0.72327
Q14240	Eukaryotic initiation factor 4A-II;Eukaryotic initiation factor 4A-	EIF4A2	1	5	1	11.1	46.402	NaN	NaN	NaN	NaN	NaN	NaN	NaN	NaN
P38919	Eukaryotic initiation factor 4A-III;Eukaryotic initiation factor 4A-	EIF4A3	1	4	2	4.4	46.871	NaN	NaN	NaN	NaN	NaN	NaN	NaN	NaN
P23588	Eukaryotic translation initiation factor 4B	EIF4B	1	1	1	2.1	69.15	NaN	NaN	NaN	NaN	NaN	NaN	NaN	NaN
P06730	Eukaryotic translation initiation factor 4E	EIF4E	1	3	3	19.8	25.097	NaN	NaN	NaN	NaN	NaN	NaN	NaN	NaN
Q04637	Eukaryotic translation initiation factor 4 gamma 1	EIF4G1	2	5	5	3.6	175.49	7.6E+07	5.8E+07	6.8E+07	7.3E+07	6.5E+07	6.4E+07	1.00479	0.9578
P78344	Eukaryotic translation initiation factor 4 gamma 2	EIF4G2	1	7	7	8.5	102.36	3.5E+07	4.7E+07	4.4E+07	4E+07	3.1E+07	3.6E+07	0.84134	0.18746
P55010	Eukaryotic translation initiation factor 5	EIF5	1	5	5	10.9	49.222	5.4E+07	5E+07	5.7E+07	5.8E+07	5.7E+07	5.5E+07	1.0501	0.3433
P63241;Q6IS14	Eukaryotic translation initiation factor 5A-1;Eukaryotic translatio	EIF5A	3	5	5	22.7	16.832	2.2E+08	3.1E+08	2.2E+08	1.7E+08	2.4E+08	2.1E+08	0.81584	0.27845
O60841	Eukaryotic translation initiation factor 5B	EIF5B	1	3	3	3.8	138.83	NaN	NaN	NaN	NaN	NaN	NaN	NaN	NaN
P56537	Eukaryotic translation initiation factor 6	EIF6	1	1	1	4.1	26.599	NaN	NaN	NaN	NaN	NaN	NaN	NaN	NaN
Q9BQ52	Zinc phosphodiesterase ELAC protein 2	ELAC2	1	3	3	3.9	92.218	1.4E+07	9975900	1.4E+07	NaN	NaN	NaN	NaN	NaN
Q15717	ELAV-like protein 1	ELAVL1	1	2	2	5.8	36.091	NaN	NaN	NaN	NaN	NaN	NaN	NaN	NaN
Q96JJ3;Q92556	Engulfment and cell motility protein 2;Engulfment and cell mot	ELMO2	2	2	2	3.5	82.614	NaN	NaN	NaN	NaN	NaN	NaN	NaN	NaN
Q8IZ81	ELMO domain-containing protein 2	ELMOD2	1	2	2	9.9	34.96	NaN	NaN	NaN	NaN	NaN	NaN	NaN	NaN
P15502	Elastin	ELN	1	1	1	1.3	68.397	NaN	NaN	NaN	NaN	NaN	NaN	NaN	NaN
Q9BW60	Elongation of very long chain fatty acids protein 1	ELOVL1	1	1	1	5	32.662	NaN	NaN	NaN	NaN	NaN	NaN	NaN	NaN
Q8N766	ER membrane protein complex subunit 1	EMC1	1	2	2	2.6	111.76	2.8E+07	NaN	2.2E+07	2.2E+07	2.4E+07	NaN	0.9161	0.58343
Q15006	ER membrane protein complex subunit 2	EMC2	1	3	3	11.8	34.833	2.5E+07	2E+07	2.3E+07	1.8E+07	2.4E+07	2.5E+07	0.99225	0.94675
Q9P0I2	ER membrane protein complex subunit 3	EMC3	1	1	1	5.4	29.952	NaN	NaN	NaN	NaN	NaN	NaN	NaN	NaN
P50402	Emerin	EMD	1	3	3	12.2	28.994	1.4E+08	1.3E+08	1.3E+08	1.5E+08	1.1E+08	NaN	0.98958	0.93452
O95834	Echinoderm microtubule-associated protein-like 2	EML2	1	1	1	2.2	70.678	NaN	NaN	NaN	NaN	NaN	NaN	NaN	NaN
Q9HC35	Echinoderm microtubule-associated protein-like 4	EML4	1	5	5	6	108.91	1.5E+08	2.1E+08	1.6E+08	1.7E+08	NaN	1.7E+08	0.98106	0.90868
Q8N8S7	Protein enabled homolog	ENAH	1	4	4	8.8	66.509	4.9E+07	6.1E+07	4.8E+07	4.7E+07	5.1E+07	4E+07	0.86927	0.25912
P06733	Alpha-enolase	ENO1	1	5	5	10.1	47.168	3.2E+08	3.1E+08	3.5E+08	3.8E+08	4.1E+08	3.4E+08	1.13802	0.13445
Q9NPA8	Transcription and mRNA export factor ENY2	ENY2	1	1	1	16.8	11.528	NaN	NaN	NaN	NaN	NaN	NaN	NaN	NaN
O43491	Band 4.1-like protein 2	EPB41L2	1	1	1	1.2	112.59	NaN	NaN	NaN	NaN	NaN	NaN	NaN	NaN
P29317	Ephrin type-A receptor 2	EPHA2	1	2	2	2	108.27	3E+07	NaN	2.8E+07	2.9E+07	3E+07	3E+07	1.02537	0.54328
P07099	Epoxide hydrolase 1	EPHX1	1	7	7	14.9	52.948	3.5E+08	4.2E+08	4.1E+08	3.5E+08	3.3E+08	3.5E+08	0.87514	0.11369
P07814	Bifunctional glutamate/proline--tRNA ligase;Glutamate--tRNA	EPRS	1	12	12	9.4	170.59	9.4E+07	8.1E+07	8.5E+07	9E+07	8.1E+07	1E+08	1.04553	0.60216
P42566	Epidermal growth factor receptor substrate 15	EPS15	1	1	1	1.5	98.655	NaN	NaN	NaN	NaN	NaN	NaN	NaN	NaN
Q9H6S3	Epidermal growth factor receptor kinase substrate 8-like prote	EPS8L2	1	3	3	6.3	80.62	2.3E+07	NaN	NaN	1.2E+07	1E+07	NaN	0.49379	NaN

Q8IUD2	ELKS/Rab6-interacting/CAST family member 1	ERC1	1	2	2	1.8	128.08	NaN	NaN	NaN	NaN	NaN	NaN	NaN	NaN
Q2NKX8	DNA excision repair protein ERCC-6-like	ERCC6L	1	1	1	1.2	141.1	NaN	NaN	NaN	NaN	NaN	NaN	NaN	NaN
Q969X5	Endoplasmic reticulum-Golgi intermediate compartment prote	ERGIC1	1	2	2	6.9	32.592	NaN	NaN	NaN	NaN	5.4E+07	5.1E+07	NaN	NaN
P84090	Enhancer of rudimentary homolog	ERH	1	1	1	16.3	12.259	NaN	NaN	NaN	NaN	NaN	NaN	NaN	NaN
O75477	Erlin-1	ERLIN1	1	2	1	6	39.171	NaN	NaN	NaN	NaN	NaN	NaN	NaN	NaN
Q7Z2K6	Endoplasmic reticulum metallopeptidase 1	ERMP1	1	1	1	1.5	100.23	NaN	NaN	NaN	NaN	NaN	NaN	NaN	NaN
Q96HE7	ERO1-like protein alpha	ERO1L	1	3	3	7.5	54.392	NaN	5.7E+07	7.6E+07	NaN	6.7E+07	6.6E+07	0.99806	0.99043
P30040	Endoplasmic reticulum resident protein 29	ERP29	1	5	5	23.8	28.993	7.1E+07	7E+07	7.3E+07	5.7E+07	6.9E+07	7.1E+07	0.92035	0.28139
Q9BSJ8	Extended synaptotagmin-1	ESYT1	1	4	4	4.3	122.85	9.8E+07	9.8E+07	1.1E+08	9.7E+07	1.2E+08	9.2E+07	1.03176	0.75454
A0FGR8	Extended synaptotagmin-2	ESYT2	1	3	3	3.6	102.36	NaN	NaN	NaN	NaN	NaN	NaN	NaN	NaN
P62495	Eukaryotic peptide chain release factor subunit 1	ETF1	1	2	2	5.3	49.03	6.5E+07	5.4E+07	5E+07	4.3E+07	5.7E+07	5.7E+07	0.92886	0.57237
P13804	Electron transfer flavoprotein subunit alpha, mitochondrial	ETFA	1	3	3	9	35.079	7.6E+07	8E+07	9.4E+07	1.1E+08	9.9E+07	7.7E+07	1.13924	0.34403
P38117	Electron transfer flavoprotein subunit beta	ETFB	1	2	2	9	27.843	1.1E+08	9E+07	1.2E+08	9.9E+07	9.4E+07	1.2E+08	0.993	0.95631
O95571	Persulfide dioxygenase ETHE1, mitochondrial	ETHE1	1	3	3	14.2	27.873	1.9E+08	2.5E+08	2.6E+08	NaN	2.2E+08	NaN	0.94747	NaN
Q96A65	Exocyst complex component 4	EXOC4	1	1	1	2	110.5	NaN	NaN	NaN	NaN	NaN	NaN	NaN	NaN
O00471	Exocyst complex component 5	EXOC5	1	3	3	4.9	81.852	2.3E+07	2.2E+07	2.1E+07	2.2E+07	2.2E+07	2.1E+07	0.98649	0.70897
Q8TAG9	Exocyst complex component 6	EXOC6	1	1	1	2.5	93.721	NaN	NaN	NaN	NaN	NaN	NaN	NaN	NaN
Q9UPT5	Exocyst complex component 7	EXOC7	1	1	1	1.5	83.381	NaN	NaN	NaN	NaN	NaN	NaN	NaN	NaN
Q5RKV6	Exosome complex component MTR3	EXOSC6	1	1	1	5.9	28.235	NaN	NaN	NaN	NaN	NaN	NaN	NaN	NaN
Q15024	Exosome complex component RRP42	EXOSC7	1	2	2	12.7	31.821	NaN	NaN	NaN	NaN	NaN	NaN	NaN	NaN
P15311	Ezrin	EZR	1	15	6	20.1	69.412	2E+08	2.1E+08	1.9E+08	2.1E+08	1.9E+08	2.1E+08	1.00871	0.82864
O95864	Fatty acid desaturase 2	FADS2	1	2	2	4.7	52.259	NaN	NaN	NaN	NaN	NaN	NaN	NaN	NaN
Q9UNN5	FAS-associated factor 1	FAF1	1	2	2	3.4	73.953	NaN	NaN	NaN	NaN	NaN	NaN	NaN	NaN
P16930	Fumarylacetoacetase	FAH	1	2	2	4.3	46.374	NaN	NaN	NaN	NaN	NaN	NaN	NaN	NaN
Q9H098	Protein FAM107B	FAM107B	1	2	2	16	15.558	NaN	NaN	NaN	NaN	NaN	NaN	NaN	NaN
Q9NZB2	Constitutive coactivator of PPAR-gamma-like protein 1	FAM120A	1	3	3	3.7	121.89	1.9E+07	2.2E+07	1.9E+07	1.9E+07	2E+07	2.1E+07	1.00219	0.97026
Q9NX05	Constitutive coactivator of PPAR-gamma-like protein 2	FAM120C	1	1	1	1.5	120.57	NaN	NaN	NaN	NaN	NaN	NaN	NaN	NaN
Q96TA1	Niban-like protein 1	FAM129B	1	2	2	2.9	84.137	3.9E+07	3.7E+07	4E+07	2.8E+07	5.3E+07	3.8E+07	1.02758	0.88939
Q9BRX8	Redox-regulatory protein FAM213A	FAM213A	1	1	1	5.2	25.764	NaN	NaN	NaN	NaN	NaN	NaN	NaN	NaN
Q9NUQ9	Protein FAM49B	FAM49B	2	4	4	15.7	36.748	5E+07	5.2E+07	4.5E+07	6.4E+07	4E+07	4.3E+07	0.99684	0.98544
Q8TBF8	Protein FAM81A	FAM81A	1	1	1	2.7	42.391	NaN	NaN	NaN	NaN	NaN	NaN	NaN	NaN
Q9Y3D0	Mitotic spindle-associated MMXD complex subunit MIP18	FAM96B	1	1	1	13.5	17.663	NaN	NaN	NaN	NaN	NaN	NaN	NaN	NaN
Q8NCA5;Q52LJ0	Protein FAM98A;Protein FAM98B	FAM98A	2	2	2	7.9	55.272	NaN	NaN	NaN	NaN	NaN	NaN	NaN	NaN
Q9NVI1	Fanconi anemia group I protein	FANCI	1	3	3	3.8	149.32	1.5E+07	NaN	1.3E+07	1.1E+07	NaN	9674100	0.75877	0.12356
Q9Y285	Phenylalanine--tRNA ligase alpha subunit	FARSA	1	4	4	9.8	57.563	9.1E+07	6.7E+07	8.4E+07	9.4E+07	6.7E+07	7.6E+07	0.97313	0.85071
Q9NSD9	Phenylalanine--tRNA ligase beta subunit	FARSB	1	5	5	10.2	66.115	3.5E+07	3.7E+07	3.6E+07	3.2E+07	4.4E+07	2.4E+07	0.93407	0.69864
P49327	Fatty acid synthase;[Acyl-carrier-protein] S-acetyltransferase;[A	FASN	1	20	20	8.9	273.42	1.5E+08	1.5E+08	1.3E+08	1.4E+08	1.5E+08	1.5E+08	0.9975	0.96414
Q7L8L6	FAST kinase domain-containing protein 5	FASTKD5	1	1	1	1.2	86.573	NaN	NaN	NaN	NaN	NaN	NaN	NaN	NaN
P62861	40S ribosomal protein S30	FAU	1	1	1	7.5	14.39	NaN	NaN	NaN	NaN	NaN	NaN	NaN	NaN
Q9UK22	F-box only protein 2	FBXO2	1	3	3	16.6	33.327	1.3E+07	1.4E+07	2.4E+07	1.5E+07	1.9E+07	1.8E+07	1.0343	0.87841
O94952	F-box only protein 21	FBXO21	1	1	1	1.8	72.269	NaN	NaN	NaN	NaN	NaN	NaN	NaN	NaN
P37268	Squalene synthase	FDFT1	1	1	1	2.4	48.115	NaN	NaN	NaN	NaN	NaN	NaN	NaN	NaN
P14324	Farnesyl pyrophosphate synthase	FDPS	1	3	3	8.8	48.275	7.2E+07	9.9E+07	7.5E+07	5.2E+07	7.9E+07	8E+07	0.85682	0.40032
P22570	NADPH:adrenodoxin oxidoreductase, mitochondrial	FDXR	1	5	5	11.4	53.836	6.3E+07	6.4E+07	5.7E+07	5.9E+07	6.3E+07	6.4E+07	1.01389	0.7785
P22830	Ferrochelatase, mitochondrial	FECH	1	2	2	4	47.862	NaN	NaN	NaN	NaN	NaN	NaN	NaN	NaN
Q96AC1	Fermitin family homolog 2	FERMT2	1	4	4	6.2	77.86	6.7E+07	NaN	NaN	7.4E+07	1.2E+07	NaN	0.63892	NaN
P09038	Fibroblast growth factor 2	FGF2	1	1	1	3.8	30.77	NaN	NaN	NaN	NaN	NaN	NaN	NaN	NaN
Q7Z7B0	Filamin-A-interacting protein 1	FILIP1	1	1	1	1.6	138.11	NaN	NaN	NaN	NaN	NaN	NaN	NaN	NaN
Q9Y3D6	Mitochondrial fission 1 protein	FIS1	1	1	1	8.6	16.937	NaN	NaN	NaN	NaN	NaN	NaN	NaN	NaN
Q96AY3	Peptidyl-prolyl cis-trans isomerase FKBP10	FKBP10	1	3	3	4.5	64.244	2.7E+07	2.3E+07	2.5E+07	2.5E+07	2.7E+07	NaN	1.04354	0.59825
Q9NYL4	Peptidyl-prolyl cis-trans isomerase FKBP11	FKBP11	1	1	1	5	22.18	NaN	NaN	NaN	NaN	NaN	NaN	NaN	NaN
P26885	Peptidyl-prolyl cis-trans isomerase FKBP2	FKBP2	1	1	1	8.5	15.649	NaN	NaN	NaN	NaN	NaN	NaN	NaN	NaN
Q00688	Peptidyl-prolyl cis-trans isomerase FKBP3	FKBP3	1	2	2	11.6	25.177	2.2E+07	7.2E+07	NaN	NaN	2.7E+07	NaN	0.57801	NaN
Q02790	Peptidyl-prolyl cis-trans isomerase FKBP4;Peptidyl-prolyl cis-tı	FKBP4	1	6	6	12.6	51.804	7E+07	1.1E+08	8.1E+07	7.1E+07	7.4E+07	8.5E+07	0.87286	0.46682
Q13045	Protein flightless-1 homolog	FLII	1	3	3	2.8	144.75	NaN	NaN	2E+07	NaN	1.7E+07	NaN	0.86456	NaN
P21333	Filamin-A	FLNA	2	45	37	19	280.74	6.6E+08	6.7E+08	7E+08	6.9E+08	6.5E+08	6.6E+08	0.98927	0.71528
O75369	Filamin-B	FLNB	1	26	18	10	278.16	6.3E+08	5.6E+08	7.3E+08	6.1E+08	6.2E+08	6.8E+08	0.99221	0.9279
Q53EP0	Fibronectin type III domain-containing protein 3B	FNDC3B	1	1	1	1.2	132.89	NaN	NaN	NaN	NaN	NaN	NaN	NaN	NaN
Q16658	Fascin	FSCN1	1	7	7	15.4	54.529	1.9E+08	2.4E+08	1.5E+08	2.3E+08	2.4E+08	2.9E+08	1.31638	0.11845

P02792	Ferritin light chain	FTL	1	1	1	8.6	20.019	NaN	NaN	NaN	NaN	NaN	NaN	NaN	NaN
Q9C0B1	Alpha-ketoglutarate-dependent dioxygenase FTO	FTO	1	1	1	3	58.281	NaN	NaN	NaN	NaN	NaN	NaN	NaN	NaN
Q96AE4	Far upstream element-binding protein 1	FUBP1	1	6	5	9.9	67.56	2.9E+08	2.8E+08	2.9E+08	3E+08	2.9E+08	2.1E+08	0.93809	0.56654
P35637	RNA-binding protein FUS	FUS	1	2	2	7.2	53.425	7E+07	1.2E+08	1.2E+08	1.1E+08	8.9E+07	1E+08	0.99207	0.96284
Q9BYC5	Alpha-(1,6)-fucosyltransferase	FUT8	1	1	1	2.4	66.515	NaN	NaN	NaN	NaN	NaN	NaN	NaN	NaN
P51114	Fragile X mental retardation syndrome-related protein 1	FXR1	2	3	1	4.3	69.72	NaN	NaN	NaN	NaN	NaN	NaN	NaN	NaN
P51116	Fragile X mental retardation syndrome-related protein 2	FXR2	1	3	1	4.5	74.222	3.7E+07	3.2E+07	NaN	3.7E+07	3.6E+07	NaN	1.04804	0.55451
Q13283	Ras GTPase-activating protein-binding protein 1	G3BP1	1	3	2	9	52.164	1.3E+07	5.4E+07	2.6E+07	2.6E+07	1.9E+07	NaN	0.72999	0.63172
P11413	Glucose-6-phosphate 1-dehydrogenase	G6PD	1	14	14	25.8	59.256	1.6E+09	1.7E+09	1.6E+09	1.7E+09	1.5E+09	1.6E+09	0.99422	0.85081
Q14376	UDP-glucose 4-epimerase	GALE	1	2	2	7.8	38.281	2.8E+07	4E+07	3.5E+07	2.2E+07	3.3E+07	3.1E+07	0.82529	0.27508
P51570	Galactokinase	GALK1	1	5	5	11.5	42.272	1.1E+08	1.2E+08	1.2E+08	7.6E+07	8.9E+07	9.4E+07	0.7436	0.00697
Q96C23	Aldose 1-epimerase	GALM	1	2	2	8.5	37.765	NaN	NaN	NaN	NaN	NaN	NaN	NaN	NaN
Q10472	Polypeptide N-acetylgalactosaminyltransferase 1;Polypeptide N	GALNT1	1	2	2	3.8	64.218	NaN	NaN	2.8E+07	2.2E+07	3E+07	3.9E+07	1.09803	NaN
Q10471	Polypeptide N-acetylgalactosaminyltransferase 2;Polypeptide N	GALNT2	1	2	2	3.5	64.732	3.6E+07	4.3E+07	NaN	2.8E+07	NaN	4.4E+07	0.91446	0.73354
Q86SF2	N-acetylgalactosaminyltransferase 7	GALNT7	1	2	2	2.6	75.388	NaN	NaN	NaN	NaN	NaN	NaN	NaN	NaN
Q14697	Neutral alpha-glucosidase AB	GANAB	1	9	9	11.4	106.87	2.1E+08	2.3E+08	2.3E+08	1.9E+08	2.3E+08	2.5E+08	0.98312	0.8467
P04406	Glyceraldehyde-3-phosphate dehydrogenase	GAPDH	1	5	5	17.6	36.053	1E+09	7.5E+08	1.1E+09	1.5E+09	1.1E+09	1.2E+09	1.36614	0.07637
P41250	Glycine--tRNA ligase	GARS	1	4	4	5.7	83.165	6.8E+07	6.2E+07	5.8E+07	6.3E+07	5.6E+07	7.4E+07	1.02345	0.81433
P22102	Trifunctional purine biosynthetic protein adenosine-3;Phospho	GART	1	2	2	2.5	107.77	NaN	NaN	NaN	NaN	NaN	NaN	NaN	NaN
O75879	Glutamyl-tRNA(Gln) amidotransferase subunit B, mitochondria	GATB	1	1	1	2	61.863	NaN	NaN	NaN	NaN	NaN	NaN	NaN	NaN
Q04446	1,4-alpha-glucan-branching enzyme	GBE1	1	3	3	4.3	80.473	2.1E+07	1.7E+07	2.6E+07	2.3E+07	2E+07	2.3E+07	1.04048	0.75598
Q92538	Golgi-specific brefeldin A-resistance guanine nucleotide excha	GBF1	1	4	4	2.4	206.44	3.1E+07	2.7E+07	2.9E+07	2.5E+07	2.7E+07	3.2E+07	0.97317	0.78142
P32455	Interferon-induced guanylate-binding protein 1	GBP1	1	1	1	1.4	67.93	NaN	NaN	NaN	NaN	NaN	NaN	NaN	NaN
P48506	Glutamate--cysteine ligase catalytic subunit	GCLC	1	2	2	5	72.765	NaN	NaN	NaN	NaN	NaN	NaN	NaN	NaN
P48507	Glutamate--cysteine ligase regulatory subunit	GCLM	1	1	1	5.5	30.727	NaN	NaN	NaN	NaN	NaN	NaN	NaN	NaN
Q92616	Translational activator GCN1	GCN1L1	1	23	23	8.9	292.71	8.5E+07	9.1E+07	9.1E+07	8.4E+07	8.1E+07	8.6E+07	0.93916	0.11501
P31150	Rab GDP dissociation inhibitor alpha	GDI1	1	3	2	10.3	50.582	1.2E+08	9.7E+07	1.1E+08	9.7E+07	1.4E+08	1.3E+08	1.10121	0.46307
P50395	Rab GDP dissociation inhibitor beta	GDI2	1	9	8	21.8	50.663	2.1E+08	1.9E+08	2E+08	2E+08	1.9E+08	2E+08	0.98227	0.60148
P57678	Gem-associated protein 4	GEMIN4	1	2	2	2.1	120.04	1.5E+07	1.5E+07	1.5E+07	1.9E+07	1.5E+07	1.3E+07	1.06133	0.65203
Q8TEQ6	Gem-associated protein 5	GEMIN5	1	2	2	1.7	168.59	NaN	NaN	NaN	NaN	NaN	NaN	NaN	NaN
Q7L5D6	Golgi to ER traffic protein 4 homolog	GET4	1	1	1	4.6	36.504	NaN	NaN	NaN	NaN	NaN	NaN	NaN	NaN
Q96RP9	Elongation factor G, mitochondrial	GFM1	1	4	4	6.1	83.471	NaN	NaN	NaN	NaN	NaN	NaN	NaN	NaN
Q06210	Glutamine--fructose-6-phosphate aminotransferase [isomeriz	GFPT1	1	10	8	15.6	78.806	8.2E+07	1E+08	8.9E+07	7.8E+07	9.6E+07	8.7E+07	0.95617	0.63459
O94808	Glutamine--fructose-6-phosphate aminotransferase [isomeriz	GFPT2	1	3	1	4.7	76.93	NaN	NaN	NaN	NaN	NaN	NaN	NaN	NaN
Q6Y7W6	PERQ amino acid-rich with GYF domain-containing protein 2	GIGYF2	1	2	2	2.8	150.07	NaN	NaN	NaN	NaN	NaN	NaN	NaN	NaN
Q14691	DNA replication complex GINS protein PSF1	GINS1	1	1	1	7.7	22.988	NaN	NaN	NaN	NaN	NaN	NaN	NaN	NaN
Q9BRX5	DNA replication complex GINS protein PSF3	GINS3	1	1	1	6.9	24.534	NaN	NaN	NaN	NaN	NaN	NaN	NaN	NaN
Q9BRT9	DNA replication complex GINS protein SLD5;DNA replication c	GINS4	1	1	1	8.5	26.047	NaN	NaN	NaN	NaN	NaN	NaN	NaN	NaN
O14908	PDZ domain-containing protein GIPC1	GIPC1	1	4	4	15.6	36.049	NaN	1.4E+07	1.4E+07	1.4E+07	1.5E+07	NaN	1.0295	0.39682
Q9Y2X7	ARF GTPase-activating protein GIT1	GIT1	1	2	2	3.3	84.34	NaN	NaN	NaN	NaN	NaN	NaN	NaN	NaN
Q92896	Golgi apparatus protein 1	GLG1	1	3	3	3.1	134.55	NaN	NaN	NaN	NaN	NaN	NaN	NaN	NaN
Q9HC38	Glyoxalase domain-containing protein 4	GLOD4	1	2	2	6.4	34.793	NaN	NaN	NaN	NaN	NaN	NaN	NaN	NaN
O76003	Glutaredoxin-3	GLRX3	1	4	4	13.7	37.432	9.2E+07	7.9E+07	8.7E+07	9.1E+07	8.9E+07	9.4E+07	1.06477	0.23763
O94925	Glutaminase kidney isoform, mitochondrial	GLS	1	4	4	5.7	73.46	1.6E+08	9.3E+07	1.2E+08	1.3E+08	1.5E+08	1.4E+08	1.11786	0.50238
P00367;P49448	Glutamate dehydrogenase 1, mitochondrial;Glutamate dehydro	GLUD1	2	8	8	16.7	61.397	1.1E+08	8.8E+07	8.8E+07	1.1E+08	1E+08	9.8E+07	1.10121	0.23897
Q96IJ6	Mannose-1-phosphate guanyltransferase alpha	GMPPA	1	1	1	5	46.291	NaN	NaN	NaN	NaN	NaN	NaN	NaN	NaN
Q9P2T1	GMP reductase 2	GMPR2	1	1	1	2.6	37.874	NaN	NaN	NaN	NaN	NaN	NaN	NaN	NaN
P49915	GMP synthase [glutamine-hydrolyzing]	GMPS	1	4	4	5.9	76.715	4E+07	4.2E+07	3.7E+07	4.3E+07	3.8E+07	4E+07	1.01841	0.75217
Q14344;P19086;O9583	Guanine nucleotide-binding protein subunit alpha-13;Guanine	GNA13	6	2	2	5.8	44.049	NaN	NaN	NaN	NaN	NaN	NaN	NaN	NaN
P63096;P08754;P04899	Guanine nucleotide-binding protein G(i) subunit alpha-1;Guan	GNAI1	6	2	2	4.8	40.361	NaN	NaN	NaN	NaN	NaN	NaN	NaN	NaN
P63092;Q5JWF2	Guanine nucleotide-binding protein G(s) subunit alpha isoform	GNAS	2	3	3	9.9	45.664	2E+07	2.2E+07	2.6E+07	2.4E+07	2.4E+07	2.1E+07	1.01103	0.91727
P62873	Guanine nucleotide-binding protein G(I)/G(S)/G(T) subunit bet	GNB1	1	1	1	4.1	37.377	NaN	NaN	NaN	NaN	NaN	NaN	NaN	NaN
P63244	Guanine nucleotide-binding protein subunit beta-2-like 1;Gua	GNB2L1	1	2	2	6.6	35.076	3.5E+08	4.2E+08	4.1E+08	4.2E+08	3.9E+08	3.2E+08	0.95418	0.62508
Q9UBI6	Guanine nucleotide-binding protein G(I)/G(S)/G(O) subunit ga	GNG12	1	1	1	22.2	8.0061	NaN	NaN	NaN	NaN	NaN	NaN	NaN	NaN
P59768	Guanine nucleotide-binding protein G(I)/G(S)/G(O) subunit ga	GNG2	1	1	1	22.5	7.8501	NaN	NaN	NaN	NaN	NaN	NaN	NaN	NaN
P46926	Glucosamine-6-phosphate isomerase 1	GNPDA1	1	1	1	3.5	32.668	NaN	NaN	NaN	NaN	NaN	NaN	NaN	NaN
Q96EK6	Glucosamine 6-phosphate N-acetyltransferase	GNPNAT1	1	2	2	7.1	20.749	NaN	NaN	NaN	NaN	NaN	NaN	NaN	NaN
P15586	N-acetylglucosamine-6-sulfatase	GNS	1	1	1	2.2	62.081	NaN	NaN	NaN	NaN	NaN	NaN	NaN	NaN
Q08378	Golgin subfamily A member 3	GOLGA3	1	1	1	0.8	167.35	NaN	NaN	NaN	NaN	NaN	NaN	NaN	NaN

Q8TBA6	Golgin subfamily A member 5	GOLGA5	1	1	1	1.9	83.023	NaN	NaN	NaN	NaN	NaN	NaN	NaN	NaN
Q14789	Golgin subfamily B member 1	GOLGB1	1	2	2	0.9	376.01	NaN	NaN	NaN	NaN	NaN	NaN	NaN	NaN
Q9H4A5	Golgi phosphoprotein 3-like	GOLPH3L	1	1	1	5.3	32.767	NaN	NaN	NaN	NaN	NaN	NaN	NaN	NaN
P17174	Aspartate aminotransferase, cytoplasmic	GOT1	1	2	2	5.8	46.247	9.3E+07	NaN	7E+07	6.9E+07	NaN	NaN	0.84942	NaN
P00505	Aspartate aminotransferase, mitochondrial	GOT2	1	3	3	9.1	47.517	NaN	NaN	NaN	NaN	NaN	NaN	NaN	NaN
Q8N335	Glycerol-3-phosphate dehydrogenase 1-like protein	GPD1L	1	2	2	5.7	38.418	NaN	3.6E+07	2.3E+07	NaN	NaN	NaN	NaN	NaN
P43304	Glycerol-3-phosphate dehydrogenase, mitochondrial	GPD2	1	1	1	1.8	80.852	NaN	NaN	NaN	NaN	NaN	NaN	NaN	NaN
P06744	Glucose-6-phosphate isomerase	GPI	2	7	7	11.8	63.146	6.4E+08	7.9E+08	6.5E+08	6.8E+08	6.2E+08	6.7E+08	0.95195	0.568
Q8NFJ5	Retinoic acid-induced protein 3	GPRC5A	1	1	1	5	40.251	NaN	NaN	NaN	NaN	NaN	NaN	NaN	NaN
Q13098	COP9 signalosome complex subunit 1	GPS1	1	3	3	6.3	55.536	NaN	3E+07	NaN	9752000	NaN	NaN	0.33054	NaN
P07203	Glutathione peroxidase 1	GPX1	1	1	1	5.4	22.088	NaN	NaN	NaN	NaN	NaN	NaN	NaN	NaN
P18283	Glutathione peroxidase 2	GPX2	1	1	1	5.8	21.954	NaN	NaN	NaN	NaN	NaN	NaN	NaN	NaN
Q8TED1	Probable glutathione peroxidase 8	GPX8	1	2	2	10.5	23.881	NaN	5.4E+07	7E+07	NaN	5.2E+07	5.6E+07	0.87056	0.42141
Q9UBQ7	Glyoxylate reductase/hydroxypyruvate reductase	GRHPR	1	2	2	3	35.668	1.1E+08	8.8E+07	1.3E+08	NaN	1.2E+08	1.5E+08	1.21058	0.3113
P42262;P42263;P42261	Glutamate receptor 2;Glutamate receptor 3;Glutamate recepto	GRIA2	3	1	1	1	98.82	2E+08	2E+08	2.1E+08	2.4E+08	NaN	2.5E+08	1.19228	0.00624
Q9HAV7	GrpE protein homolog 1, mitochondrial	GRPEL1	1	1	1	5.1	24.279	2.7E+07	2.6E+07	NaN	2.7E+07	2.7E+07	2.9E+07	1.01917	0.59509
P15170;Q8IYD1	Eukaryotic peptide chain release factor GTP-binding subunit EI	GSPT1	2	4	4	12.6	55.755	1.4E+08	1.4E+08	1.3E+08	1.6E+08	1.2E+08	1.4E+08	1.02408	0.76176
P00390	Glutathione reductase, mitochondrial	GSR	1	1	1	1.3	56.256	NaN	NaN	NaN	NaN	NaN	NaN	NaN	NaN
P48637	Glutathione synthetase	GSS	1	4	4	7.4	52.384	2.7E+07	3.1E+07	NaN	2.6E+07	3E+07	2.9E+07	0.97556	0.77242
P21266	Glutathione S-transferase Mu 3	GSTM3	1	2	2	8.9	26.559	3.9E+07	3.3E+07	3.5E+07	3.7E+07	3.5E+07	3.5E+07	0.98917	0.84674
P78417	Glutathione S-transferase omega-1	GSTO1	1	7	7	17.8	27.566	2.5E+08	2.7E+08	2.3E+08	3.2E+08	2.8E+08	3.2E+08	1.22903	0.02535
P09211	Glutathione S-transferase P	GSTP1	1	1	1	5.2	23.356	NaN	NaN	NaN	NaN	NaN	NaN	NaN	NaN
P52655	Transcription initiation factor IIA subunit 1;Transcription initiati	GTF2A1	1	1	1	2.9	41.513	NaN	NaN	NaN	NaN	NaN	NaN	NaN	NaN
P78347	General transcription factor II-I	GTF2I	1	5	5	5.6	112.42	5.3E+07	NaN	4E+07	4.3E+07	6.3E+07	NaN	1.14026	0.64363
Q9UBP9	PTB domain-containing engulfment adapter protein 1	GULP1	1	1	1	3.6	34.49	NaN	NaN	NaN	NaN	NaN	NaN	NaN	NaN
P13807	Glycogen [starch] synthase, muscle	GYS1	1	2	2	3.8	83.785	NaN	NaN	NaN	NaN	NaN	NaN	NaN	NaN
Q9P035	Very-long-chain (3R)-3-hydroxyacyl-CoA dehydratase 3	HACD3	1	5	5	14.9	43.159	1.9E+08	1.8E+08	1.8E+08	1.3E+08	1.6E+08	2E+08	0.8891	0.32392
Q16836	Hydroxyacyl-coenzyme A dehydrogenase, mitochondrial	HADH	1	1	1	2.9	34.293	NaN	NaN	NaN	NaN	NaN	NaN	NaN	NaN
P40939	Trifunctional enzyme subunit alpha, mitochondrial;Long-chain	HADHA	1	6	6	11.5	82.999	1.3E+08	1.2E+08	9.9E+07	1.1E+08	1.1E+08	8.2E+07	0.88403	0.38327
P55084	Trifunctional enzyme subunit beta, mitochondrial;3-ketoacyl-C	HADHB	1	6	6	12.2	51.294	9E+07	1.1E+08	9.1E+07	6.9E+07	9.5E+07	8.6E+07	0.85817	0.23856
Q16775	Hydroxyacylglutathione hydrolase, mitochondrial	HAGH	1	2	2	7.1	33.805	NaN	NaN	NaN	4E+07	NaN	4.9E+07	NaN	NaN
P12081	Histidine--tRNA ligase, cytoplasmic	HARS	2	4	4	8.4	57.41	7.8E+07	8.2E+07	6.5E+07	7.6E+07	7.4E+07	6.9E+07	0.97234	0.73469
P53701	Cytochrome c-type heme lyase	HCCS	1	1	1	4.1	30.601	NaN	NaN	NaN	NaN	NaN	NaN	NaN	NaN
P51858	Hepatoma-derived growth factor	HDGF	1	5	5	27.1	26.788	1.1E+08	NaN	8.8E+07	NaN	9.6E+07	NaN	0.96204	NaN
Q7Z4V5	Hepatoma-derived growth factor-related protein 2	HDGFRP2	1	3	3	5.4	74.316	2E+07	NaN	1.7E+07	2.3E+07	NaN	1.8E+07	1.12704	0.53543
Q9Y3E1	Hepatoma-derived growth factor-related protein 3	HDGFRP3	1	1	1	7.4	22.619	NaN	NaN	NaN	NaN	NaN	NaN	NaN	NaN
Q00341	Vigilin	HDLBP	1	6	6	6.3	141.45	3.1E+07	3E+07	3.1E+07	3E+07	3.6E+07	2.5E+07	0.99697	0.97746
Q9NRV9	Heme-binding protein 1	HEBP1	1	1	1	7.9	21.097	NaN	NaN	NaN	NaN	NaN	NaN	NaN	NaN
Q9ULT8	E3 ubiquitin-protein ligase HECTD1	HECTD1	1	3	3	1.4	289.37	2.4E+07	NaN	2.8E+07	2.7E+07	2.4E+07	NaN	0.97049	0.80723
Q9BYK8	Helicase with zinc finger domain 2	HELZ2	1	1	1	0.6	294.65	NaN	NaN	NaN	NaN	NaN	NaN	NaN	NaN
Q5GLZ8	Probable E3 ubiquitin-protein ligase HERC4	HERC4	1	1	1	1.4	118.56	NaN	NaN	NaN	NaN	NaN	NaN	NaN	NaN
O94992	Protein HEXIM1	HEXIM1	1	1	1	4.7	40.623	NaN	NaN	NaN	NaN	NaN	NaN	NaN	NaN
Q9BTY7	Protein HGH1 homolog	HGH1	1	1	1	3.1	42.129	NaN	NaN	NaN	NaN	NaN	NaN	NaN	NaN
O14964	Hepatocyte growth factor-regulated tyrosine kinase substrate	HGS	1	2	2	2.8	86.191	NaN	NaN	NaN	1.9E+07	NaN	1.8E+07	NaN	NaN
Q6NVY1	3-hydroxyisobutyryl-CoA hydrolase, mitochondrial	HIBCH	1	3	3	8.5	43.482	NaN	NaN	NaN	NaN	NaN	NaN	NaN	NaN
Q9NWT6	Hypoxia-inducible factor 1-alpha inhibitor	HIF1AN	1	1	1	3.7	40.285	NaN	NaN	NaN	NaN	NaN	NaN	NaN	NaN
Q9Y241	HIG1 domain family member 1A, mitochondrial	HIGD1A	1	1	1	19.4	10.143	NaN	NaN	NaN	NaN	NaN	NaN	NaN	NaN
P49773	Histidine triad nucleotide-binding protein 1	HINT1	1	2	2	12.7	13.802	1.9E+08	2.3E+08	1.6E+08	1.6E+08	1.4E+08	1.6E+08	0.80808	0.15759
P16401	Histone H1.5	HIST1H1B	1	2	2	12.4	22.58	NaN	1.4E+08	1.3E+08	9.9E+07	1.3E+08	1.4E+08	0.91496	0.53721
P16403	Histone H1.2	HIST1H1C	1	3	1	13.6	21.364	NaN	NaN	NaN	NaN	NaN	NaN	NaN	NaN
P16402	Histone H1.3	HIST1H1D	1	3	1	13.1	22.35	5.8E+08	4.4E+08	6.5E+08	6.1E+08	6.1E+08	6.3E+08	1.09848	0.42107
P10412	Histone H1.4	HIST1H1E	1	3	1	13.2	21.865	NaN	NaN	NaN	NaN	NaN	NaN	NaN	NaN
Q99878;Q96KK5;Q9BTM	Histone H2A type 1-J;Histone H2A type 1-H;Histone H2A.J;His	HIST1H2AJ	13	2	1	29.7	13.936	NaN	NaN	NaN	NaN	NaN	NaN	NaN	NaN
Q99880;Q99879;Q9987	Histone H2B type 1-L;Histone H2B type 1-M;Histone H2B type	HIST1H2BL	16	2	2	15.9	13.952	9.8E+07	8E+07	8.7E+07	1E+08	9.3E+07	NaN	1.10659	0.30478
Q16777;Q6FI13	Histone H2A type 2-C;Histone H2A type 2-A	HIST2H2AC	2	2	1	29.5	13.988	NaN	NaN	NaN	NaN	NaN	NaN	NaN	NaN
Q5TEC6;Q6NXT2;Q71D	Histone H3;Histone H3.3C;Histone H3.2;Histone H3.1t;Histone I	HIST2H3PS	6	1	1	5.1	15.43	NaN	NaN	NaN	NaN	NaN	NaN	NaN	NaN
P19367	Hexokinase-1	HK1	1	4	4	4.5	102.48	5.5E+07	5.2E+07	5.1E+07	5.2E+07	5E+07	5.5E+07	0.99429	0.88363
Q2TB90	Putative hexokinase HKDC1	HKDC1	1	3	3	4.3	102.54	2.7E+07	3E+07	3.6E+07	2.4E+07	NaN	NaN	0.78121	NaN
P01889;P17693;P13747	HLA class I histocompatibility antigen, B-7 alpha chain;HLA cla:	HLA-B	3	2	1	7.5	40.46	NaN	NaN	NaN	NaN	NaN	NaN	NaN	NaN

P10321	HLA class I histocompatibility antigen, Cw-7 alpha chain	HLA-C	1	2	1	10.4	40.648	2.5E+07	2.5E+07	2.7E+07	2.4E+07	2.3E+07	2.6E+07	0.94084	0.25673
Q8TCT9	Minor histocompatibility antigen H13	HM13	1	3	3	7.7	41.488	8.8E+07	9.1E+07	1.1E+08	1.2E+08	1.2E+08	8.9E+07	1.12428	0.35673
P17096	High mobility group protein HMG-I/HMG-Y	HMGA1	1	2	2	22.4	11.676	NaN	9.3E+07	4.8E+07	NaN	6.3E+07	NaN	0.89258	NaN
P52926	High mobility group protein HMGI-C	HMGA2	1	1	1	11.9	11.832	NaN	NaN	NaN	NaN	NaN	NaN	NaN	NaN
B2RPK0;P09429	Putative high mobility group protein B1-like 1;High mobility gr	HMGB1P1	3	4	4	14.7	24.238	5.1E+08	5.3E+08	4.3E+08	6.4E+08	4.7E+08	4.3E+08	1.04784	0.75916
P26583	High mobility group protein B2	HMGB2	1	2	2	7.2	24.033	NaN	NaN	NaN	NaN	NaN	NaN	NaN	NaN
O15347	High mobility group protein B3	HMGB3	1	1	1	6.5	22.98	NaN	NaN	NaN	NaN	NaN	NaN	NaN	NaN
Q01581	Hydroxymethylglutaryl-CoA synthase, cytoplasmic	HMGCS1	1	1	1	2.5	57.293	NaN	NaN	NaN	NaN	NaN	NaN	NaN	NaN
P09601	Heme oxygenase 1	HMOX1	1	4	4	19.1	32.818	6.9E+07	1E+08	6.9E+07	7.9E+07	7.7E+07	6.6E+07	0.91732	0.62178
P30519	Heme oxygenase 2	HMOX2	1	3	3	10.8	36.032	NaN	3.2E+07	2.5E+07	2.7E+07	2.7E+07	2.6E+07	0.93752	0.52532
Q13151	Heterogeneous nuclear ribonucleoprotein A0	HNRNPA0	1	1	1	4.3	30.84	NaN	NaN	NaN	NaN	NaN	NaN	NaN	NaN
P09651;Q32P51	Heterogeneous nuclear ribonucleoprotein A1;Heterogeneous r	HNRNPA1	2	2	2	6.5	38.746	NaN	NaN	NaN	NaN	NaN	NaN	NaN	NaN
P22626	Heterogeneous nuclear ribonucleoproteins A2/B1	HNRNPA2I	1	6	6	18.1	37.429	2E+08	2.3E+08	1.8E+08	1.7E+08	2E+08	2.1E+08	0.94646	0.5512
P51991	Heterogeneous nuclear ribonucleoprotein A3	HNRNPA3	1	3	3	8.2	39.594	5.8E+07	7.5E+07	5.8E+07	5.4E+07	5.3E+07	5.8E+07	0.86494	0.21569
P0DMR1;O60812;B7ZW	Heterogeneous nuclear ribonucleoprotein C-like 4;Heterogene	HNRNPCL4	5	2	2	6.8	32.029	NaN	NaN	NaN	NaN	NaN	NaN	NaN	NaN
Q14103	Heterogeneous nuclear ribonucleoprotein D0	HNRNPD	1	3	3	8.7	38.434	2.2E+08	2E+08	2.5E+08	2.1E+08	2.3E+08	2.3E+08	1.01273	0.88014
P52597	Heterogeneous nuclear ribonucleoprotein F;Heterogeneous nu	HNRNPF	1	5	4	15.2	45.671	4.2E+07	5.4E+07	5.2E+07	3.9E+07	5.1E+07	4.2E+07	0.88747	0.32719
P31943;P55795	Heterogeneous nuclear ribonucleoprotein H;Heterogeneous nu	HNRNPH1	2	4	3	10	49.229	9.7E+07	9E+07	1.2E+08	1E+08	1.1E+08	1.3E+08	1.08411	0.5234
P31942	Heterogeneous nuclear ribonucleoprotein H3	HNRNPH3	1	1	1	3.8	36.926	NaN	NaN	NaN	NaN	NaN	NaN	NaN	NaN
P61978	Heterogeneous nuclear ribonucleoprotein K	HNRNPK	1	12	12	26.6	50.976	1.6E+08	1.7E+08	1.5E+08	2E+08	1.6E+08	1.7E+08	1.0812	0.38756
Q8WVV9	Heterogeneous nuclear ribonucleoprotein L-like	HNRNPLL	1	2	2	5.7	60.082	NaN	NaN	NaN	NaN	NaN	NaN	NaN	NaN
P52272	Heterogeneous nuclear ribonucleoprotein M	HNRNPM	1	3	3	4.8	77.515	2.8E+07	NaN	NaN	2.7E+07	2.2E+07	2.5E+07	0.90026	NaN
Q00839	Heterogeneous nuclear ribonucleoprotein U	HNRNPU	1	8	8	9.5	90.583	1.9E+08	1.6E+08	1.3E+08	1.6E+08	1.8E+08	1.7E+08	1.06004	0.60393
Q9BUJ2	Heterogeneous nuclear ribonucleoprotein U-like protein 1	HNRNPUL1	1	5	5	4.6	95.737	1.2E+08	8.3E+07	1.2E+08	1E+08	1.2E+08	1.3E+08	1.08346	0.58777
P37235;P84074;P61601	Hippocalcin-like protein 1;Neuron-specific calcium-binding pr	HPCAL1	3	2	2	11.4	22.313	NaN	NaN	NaN	NaN	NaN	NaN	NaN	NaN
P00492	Hypoxanthine-guanine phosphoribosyltransferase	HPRT1	1	3	3	17.4	24.579	1.5E+08	1.1E+08	1.5E+08	1.3E+08	1.4E+08	1.5E+08	1.05528	0.64328
Q86YV9	Hermansky-Pudlak syndrome 6 protein	HPS6	1	1	1	2.6	82.974	NaN	NaN	NaN	NaN	NaN	NaN	NaN	NaN
P52758	Ribonuclease UK114	HRSP12	1	1	1	8	14.494	NaN	NaN	NaN	NaN	NaN	NaN	NaN	NaN
Q99714	3-hydroxyacyl-CoA dehydrogenase type-2	HSD17B10	1	2	2	7.3	26.923	NaN	NaN	NaN	NaN	NaN	NaN	NaN	NaN
Q8NBQ5	Estradiol 17-beta-dehydrogenase 11	HSD17B11	1	3	3	11.7	32.963	4.2E+07	NaN	4.8E+07	3.8E+07	4.4E+07	4.1E+07	0.90822	0.26177
Q53GQ0	Very-long-chain 3-oxoacyl-CoA reductase	HSD17B12	1	5	5	11.2	34.324	NaN	4.3E+07	6.2E+07	9E+07	NaN	1.1E+08	1.86103	0.06426
P51659	Peroxisomal multifunctional enzyme type 2;(3R)-hydroxyacyl-C	HSD17B4	1	2	2	2.9	79.685	5.3E+07	5.7E+07	7.3E+07	5.7E+07	6.2E+07	7.1E+07	1.03638	0.77624
P07900	Heat shock protein HSP 90-alpha	HSP90AA1	1	16	7	25.5	84.659	3.9E+08	4.2E+08	3.5E+08	3.5E+08	3.8E+08	2.3E+08	0.83072	0.2749
P08238	Heat shock protein HSP 90-beta	HSP90AB1	6	19	10	31.2	83.263	1.9E+09	2E+09	2E+09	2.1E+09	2E+09	2.1E+09	1.04531	0.18369
P14625	Endoplasmin	HSP90B1	2	13	13	15.3	92.468	5.1E+08	5.8E+08	5.4E+08	5.4E+08	5.2E+08	5.3E+08	0.96787	0.45463
Q0VDF9	Heat shock 70 kDa protein 14	HSPA14	1	1	1	1.8	54.794	NaN	NaN	NaN	NaN	NaN	NaN	NaN	NaN
P0DMV8;P0DMV9	Heat shock 70 kDa protein 1A;Heat shock 70 kDa protein 1B	HSPA1A	3	9	4	13.9	70.051	2.5E+08	2.2E+08	1.9E+08	2.8E+08	2.9E+08	2.4E+08	1.23596	0.08325
P54652	Heat shock-related 70 kDa protein 2	HSPA2	1	9	6	14.9	70.02	1.3E+08	1.5E+08	1.2E+08	1.9E+08	1.3E+08	1.5E+08	1.20129	0.22125
P34932	Heat shock 70 kDa protein 4	HSPA4	1	12	12	20	94.33	1.7E+08	1.7E+08	1.8E+08	1.5E+08	1.7E+08	1.8E+08	0.99299	0.8948
O95757	Heat shock 70 kDa protein 4L	HSPA4L	1	3	3	3.5	94.511	5.3E+07	6.6E+07	NaN	4E+07	4.2E+07	NaN	0.6898	0.10256
P11021	78 kDa glucose-regulated protein	HSPA5	1	12	11	18.2	72.332	6.3E+08	6.4E+08	6.5E+08	7.3E+08	6.8E+08	6.7E+08	1.08536	0.05437
P11142	Heat shock cognate 71 kDa protein	HSPA8	1	12	9	20.6	70.897	9.1E+08	9.2E+08	8.4E+08	1E+09	8.7E+08	9E+08	1.03872	0.49625
P38646	Stress-70 protein, mitochondrial	HSPA9	1	10	10	22.7	73.68	2.6E+08	2.7E+08	2.7E+08	2.7E+08	2.4E+08	2.7E+08	0.98674	0.75213
P04792	Heat shock protein beta-1	HSPB1	1	5	5	27.3	22.782	2.4E+08	1.6E+08	2.1E+08	2.7E+08	1.9E+08	2.7E+08	1.19759	0.33561
Q9Y547	Intraflagellar transport protein 25 homolog	HSPB11	1	1	1	7.6	16.297	NaN	NaN	NaN	NaN	NaN	NaN	NaN	NaN
Q9NZL4	Hsp70-binding protein 1	HSPBP1	1	2	2	3.9	39.302	3.6E+07	2.7E+07	3.5E+07	4.2E+07	2.6E+07	3.6E+07	1.05932	0.7394
P10809	60 kDa heat shock protein, mitochondrial	HSPD1	1	15	15	27.4	61.054	8.8E+08	8.5E+08	9.2E+08	1E+09	8.2E+08	8.9E+08	1.0248	0.72158
P61604	10 kDa heat shock protein, mitochondrial	HSPE1	1	4	4	39.2	10.932	3.2E+08	4.2E+08	5.2E+08	4.6E+08	6.4E+08	5.1E+08	1.26878	0.2241
Q92598	Heat shock protein 105 kDa	HSPH1	1	15	15	19.8	96.864	9.1E+07	1.1E+08	1.3E+08	9.5E+07	1.2E+08	1.2E+08	1.0174	0.89089
Q9BUP3	Oxidoreductase HTATIP2	HTATIP2	1	3	3	12.4	27.049	NaN	3.6E+07	2.6E+07	2.9E+07	3.2E+07	NaN	0.98183	0.92662
Q7Z6Z7	E3 ubiquitin-protein ligase HUWE1	HUWE1	1	7	7	2	481.89	5.9E+07	4.8E+07	6.1E+07	5.4E+07	5.4E+07	6.3E+07	1.01814	0.84799
Q9Y4L1	Hypoxia up-regulated protein 1	HYOU1	1	3	3	4	111.33	NaN	NaN	NaN	NaN	NaN	NaN	NaN	NaN
Q9NX55	Huntingtin-interacting protein K	HYPK	1	1	1	20.7	13.651	NaN	NaN	NaN	NaN	NaN	NaN	NaN	NaN
P41252	Isoleucine--tRNA ligase, cytoplasmic	IARS	1	5	5	4.2	144.5	5.1E+07	5.1E+07	5.8E+07	5E+07	6.1E+07	4.9E+07	1.00452	0.96147
Q9NSE4	Isoleucine--tRNA ligase, mitochondrial	IARS2	1	2	2	2.9	113.79	NaN	NaN	NaN	NaN	NaN	NaN	NaN	NaN
P14735	Insulin-degrading enzyme	IDE	1	4	4	4	117.97	3.8E+07	2.9E+07	3.6E+07	3.1E+07	3.6E+07	3.6E+07	1.00301	0.97688
O75874	Isocitrate dehydrogenase [NADP] cytoplasmic	IDH1	1	6	6	12.1	46.659	1.9E+08	2.4E+08	1.8E+08	2.2E+08	2.1E+08	1.7E+08	0.97929	0.86696
P48735	Isocitrate dehydrogenase [NADP], mitochondrial	IDH2	1	5	5	12.2	50.909	NaN	4.9E+07	3.4E+07	NaN	1.9E+07	5.2E+07	0.8524	0.76595
P50213	Isocitrate dehydrogenase [NAD] subunit alpha, mitochondrial	IDH3A	1	4	4	10.1	39.591	4.7E+07	NaN	3.5E+07	4.5E+07	3.6E+07	3.7E+07	0.9629	0.80245

Q13907	Isopentenyl-diphosphate Delta-isomerase 1	IDI1	1	2	2	5.7	26.319	NaN	NaN	NaN	NaN	NaN	NaN	NaN	NaN
Q9Y5U9	Immediate early response 3-interacting protein 1	IER3IP1	1	1	1	24.4	8.9687	NaN	NaN	NaN	NaN	NaN	NaN	NaN	NaN
P80217	Interferon-induced 35 kDa protein	IFI35	1	1	1	3.8	31.546	NaN	NaN	NaN	NaN	NaN	NaN	NaN	NaN
P09914	Interferon-induced protein with tetratricopeptide repeats 1	IFIT1	1	1	1	2.3	55.36	NaN	NaN	NaN	NaN	NaN	NaN	NaN	NaN
Q8WYA0	Intraflagellar transport protein 81 homolog	IFT81	1	1	1	2.7	79.745	NaN	NaN	NaN	NaN	NaN	NaN	NaN	NaN
P78318	Immunoglobulin-binding protein 1	IGBP1	1	2	2	7.4	39.221	NaN	3.4E+07	1.9E+07	NaN	1.9E+07	NaN	0.7275	NaN
P01344	Insulin-like growth factor II;Insulin-like growth factor II;Insulin-	IGF2	1	1	1	5	20.14	NaN	NaN	NaN	NaN	NaN	NaN	NaN	NaN
Q9NZI8	Insulin-like growth factor 2 mRNA-binding protein 1	IGF2BP1	1	6	6	10.1	63.48	5E+07	3.8E+07	5.4E+07	5.2E+07	5E+07	5.4E+07	1.09939	0.39228
O00425	Insulin-like growth factor 2 mRNA-binding protein 3	IGF2BP3	1	2	2	4.5	63.704	2E+07	1.5E+07	2.3E+07	NaN	2.1E+07	NaN	1.09981	NaN
P11717	Cation-independent mannose-6-phosphate receptor	IGF2R	1	1	1	0.6	274.37	NaN	NaN	NaN	NaN	NaN	NaN	NaN	NaN
P22692	Insulin-like growth factor-binding protein 4	IGFBP4	1	3	3	16.3	27.934	2.3E+07	2.7E+07	2.7E+07	2.7E+07	3E+07	2.3E+07	1.04928	0.63998
Q9Y6K9	NF-kappa-B essential modulator	IKBKG	1	1	1	2.9	48.197	NaN	NaN	NaN	NaN	NaN	NaN	NaN	NaN
Q13478	Interleukin-18 receptor 1	IL18R1	1	1	1	1.3	62.303	NaN	NaN	NaN	NaN	NaN	NaN	NaN	NaN
Q12905	Interleukin enhancer-binding factor 2	ILF2	1	3	3	5.9	43.062	5.8E+07	6E+07	2.6E+07	4.7E+07	5.5E+07	4.7E+07	1.03839	0.87844
Q12906	Interleukin enhancer-binding factor 3	ILF3	2	7	7	8.1	95.337	4.7E+07	4.6E+07	4.8E+07	5.8E+07	5.2E+07	4.7E+07	1.11822	0.1748
Q13418	Integrin-linked protein kinase	ILK	1	3	3	6.2	51.419	5.2E+07	5E+07	4.7E+07	6.5E+07	4.9E+07	5E+07	1.10036	0.42101
Q16891	MICOS complex subunit MIC60	IMMT	1	7	7	9.6	83.677	1.3E+08	1.1E+08	1.3E+08	1.3E+08	1.1E+08	1.4E+08	1.00976	0.90795
Q9P2X3	Protein IMPACT	IMPACT	1	1	1	6.2	36.476	NaN	NaN	NaN	NaN	NaN	NaN	NaN	NaN
P12268	Inosine-5-monophosphate dehydrogenase 2	IMPDH2	1	8	8	19.8	55.804	5E+07	7.8E+07	5.7E+07	5.7E+07	8.9E+07	3.8E+07	0.99717	0.99239
Q27J81	Inverted formin-2	INF2	1	2	2	2.8	135.62	NaN	NaN	NaN	NaN	NaN	NaN	NaN	NaN
O15327	Type II inositol 3,4-bisphosphate 4-phosphatase	INPP4B	1	1	1	1.7	104.74	NaN	NaN	NaN	NaN	NaN	NaN	NaN	NaN
Q92835	Phosphatidylinositol 3,4,5-trisphosphate 5-phosphatase 1	INPP5D	1	1	1	0.6	133.29	NaN	NaN	NaN	NaN	NaN	NaN	NaN	NaN
Q8TEX9	Importin-4	IPO4	1	2	2	1.8	118.71	NaN	NaN	NaN	NaN	NaN	NaN	NaN	NaN
O00410	Importin-5	IPO5	2	8	8	7.2	123.63	9.7E+07	8.4E+07	9.4E+07	9.8E+07	1E+08	1.1E+08	1.11672	0.10537
O95373	Importin-7	IPO7	1	6	6	6.5	119.52	2.1E+08	1.7E+08	2E+08	2.2E+08	1.9E+08	2.3E+08	1.11447	0.20963
Q96P70	Importin-9	IPO9	1	1	1	1.1	115.96	NaN	NaN	NaN	NaN	NaN	NaN	NaN	NaN
P46940	Ras GTPase-activating-like protein IQGAP1	IQGAP1	2	23	22	13.8	189.25	2.3E+08	2.2E+08	2.1E+08	1.9E+08	2.3E+08	2.2E+08	0.96297	0.55971
Q9H0X4	Protein ITFG3	ITFG3	1	1	1	2.4	59.659	NaN	NaN	NaN	NaN	NaN	NaN	NaN	NaN
P23229	Integrin alpha-6;Integrin alpha-6 heavy chain;Integrin alpha-6	ITGA6	1	1	1	1.2	126.6	NaN	NaN	NaN	NaN	NaN	NaN	NaN	NaN
P06756	Integrin alpha-V;Integrin alpha-V heavy chain;Integrin alpha-V	ITGAV	1	2	2	1.9	116.04	8.4E+07	1.2E+08	NaN	1E+08	NaN	NaN	1.00502	NaN
P05556	Integrin beta-1	ITGB1	1	12	12	14.8	88.414	1.7E+08	1.8E+08	1.8E+08	2E+08	1.8E+08	1.9E+08	1.07766	0.19319
P18564	Integrin beta-6	ITGB6	1	1	1	1.1	85.935	NaN	NaN	NaN	NaN	NaN	NaN	NaN	NaN
Q13572	Inositol-tetrakisphosphate 1-kinase	ITPK1	1	1	1	3.4	45.621	NaN	NaN	NaN	NaN	NaN	NaN	NaN	NaN
Q96ST2	Protein IWS1 homolog	IWS1	1	1	1	1.1	91.954	NaN	NaN	NaN	NaN	NaN	NaN	NaN	NaN
Q8N5M9	Protein jagunal homolog 1	JAGN1	1	1	1	6.6	21.125	NaN	NaN	NaN	NaN	NaN	NaN	NaN	NaN
P14923	Junction plakoglobin	JUP	1	3	3	4.2	81.744	NaN	2.8E+07	NaN	NaN	3E+07	NaN	1.08417	NaN
P33947;P24390	ER lumen protein-retaining receptor 2;ER lumen protein-retair	KDELR2	2	1	1	5.2	24.422	NaN	NaN	4.6E+07	5.3E+07	NaN	NaN	1.14446	NaN
Q07666	KH domain-containing, RNA-binding, signal transduction-asso	KHDRBS1	1	2	2	5.4	48.227	2E+08	2.3E+07	3.7E+07	3.9E+07	NaN	NaN	0.43657	NaN
Q92945	Far upstream element-binding protein 2	KHSRP	2	3	2	5.2	73.114	NaN	NaN	2.1E+07	2.4E+07	1.7E+07	7689800	0.76694	NaN
Q12768	WASH complex subunit strumpellin	KIAA0196	1	1	1	1.1	134.28	NaN	NaN	NaN	NaN	NaN	NaN	NaN	NaN
Q2M389	WASH complex subunit 7	KIAA1033	1	1	1	0.9	136.4	NaN	NaN	NaN	NaN	NaN	NaN	NaN	NaN
A0MZ66	Shootin-1	KIAA1598	1	4	4	11.3	71.639	2.6E+07	2.7E+07	2.8E+07	2.1E+07	1.9E+07	2.4E+07	0.80634	0.03855
Q68DE3	Basic helix-loop-helix domain-containing protein KIAA2018	KIAA2018	1	2	2	0.8	241.65	NaN	NaN	1.3E+08	1.6E+08	1.3E+08	NaN	1.17521	NaN
Q9Y496	Kinesin-like protein KIF3A	KIF3A	1	1	1	1.7	80.04	NaN	NaN	NaN	NaN	NaN	NaN	NaN	NaN
O15066	Kinesin-like protein KIF3B;Kinesin-like protein KIF3B, N-termina	KIF3B	1	2	2	3.1	85.124	NaN	NaN	NaN	NaN	NaN	NaN	NaN	NaN
P33176	Kinesin-1 heavy chain	KIF5B	3	7	7	10	109.68	2E+07	2.4E+07	2.5E+07	1.8E+07	2.2E+07	2.1E+07	0.88804	0.22484
Q07866	Kinesin light chain 1	KLC1	2	5	3	9.4	65.309	NaN	NaN	NaN	NaN	NaN	NaN	NaN	NaN
Q03164	Histone-lysine N-methyltransferase 2A;MLL cleavage product	KMT2A	1	1	1	0.4	431.76	NaN	NaN	NaN	NaN	NaN	NaN	NaN	NaN
P52294	Importin subunit alpha-5;Importin subunit alpha-5, N-termina	KPNA1	1	1	1	3.3	60.221	NaN	NaN	NaN	NaN	NaN	NaN	NaN	NaN
O00629	Importin subunit alpha-3	KPNA4	2	3	3	9.2	57.886	4.4E+07	6.1E+07	5.9E+07	4E+07	4.4E+07	4.9E+07	0.80819	0.14973
O60684;O15131	Importin subunit alpha-7;Importin subunit alpha-6	KPNA6	2	2	2	4.7	60.029	NaN	NaN	NaN	NaN	NaN	NaN	NaN	NaN
Q14974	Importin subunit beta-1	KPNB1	1	5	5	8.1	97.169	2.6E+08	2.9E+08	2.5E+08	2.9E+08	2.7E+08	2.1E+08	0.95721	0.6642
P05783	Keratin, type I cytoskeletal 18	KRT18	3	15	14	24.7	48.057	1.8E+09	2E+09	1.9E+09	1.9E+09	1.9E+09	1.9E+09	1.00384	0.92559
Q86UP2	Kinectin	KTN1	1	9	9	7.1	156.27	3.2E+07	3.8E+07	3.4E+07	4.1E+07	2.8E+07	3.4E+07	0.99242	0.95316
Q16719	Kynureninase	KYNU	1	4	4	6.5	52.351	5.9E+08	5.7E+08	6.3E+08	4.9E+08	5.3E+08	5.1E+08	0.8594	0.01902
P83111	Serine beta-lactamase-like protein LACTB, mitochondrial	LACTB	1	2	2	2.2	60.693	NaN	2.1E+07	2E+07	NaN	NaN	2.2E+07	1.0725	NaN
Q53H82	Beta-lactamase-like protein 2	LACTB2	1	3	3	11.1	32.805	NaN	8.7E+07	3.4E+07	NaN	NaN	NaN	NaN	NaN
O15230	Laminin subunit alpha-5	LAMA5	1	3	3	1.1	399.73	NaN	NaN	NaN	NaN	NaN	NaN	NaN	NaN
P07942	Laminin subunit beta-1	LAMB1	1	4	4	2.6	198.04	2.4E+07	2.6E+07	NaN	2.6E+07	2.7E+07	2.2E+07	0.99663	0.97193

P11047	Laminin subunit gamma-1	LAMC1	1	1	1	0.9	177.6	NaN	NaN	NaN	NaN	NaN	NaN	NaN	NaN
P11279	Lysosome-associated membrane glycoprotein 1	LAMP1	1	1	1	2.2	44.882	NaN	NaN	NaN	NaN	NaN	NaN	NaN	NaN
P13473	Lysosome-associated membrane glycoprotein 2	LAMP2	1	1	1	2	44.96	NaN	NaN	NaN	NaN	NaN	NaN	NaN	NaN
O43813	LanC-like protein 1	LANCL1	1	5	5	15	45.283	4.4E+07	4.2E+07	4.7E+07	3.4E+07	4.8E+07	4.8E+07	0.97223	0.8103
P28838	Cytosol aminopeptidase	LAP3	1	6	6	12.9	56.166	2.6E+07	2.6E+07	3E+07	3E+07	2.1E+07	3E+07	0.99401	0.96095
Q659C4;Q6PKG0	La-related protein 1B;La-related protein 1	LARP1B	2	1	1	1.1	105.32	NaN	NaN	NaN	NaN	NaN	NaN	NaN	NaN
Q4G0J3	La-related protein 7	LARP7	1	2	2	3.8	66.898	NaN	NaN	NaN	NaN	NaN	NaN	NaN	NaN
Q9P2J5	Leucine--tRNA ligase, cytoplasmic	LARS	1	7	7	6.3	134.46	5.4E+07	5.8E+07	5.2E+07	6E+07	5.6E+07	5.5E+07	1.04397	0.34517
Q53QV2	Protein LBH	LBH	1	1	1	18.1	12.217	NaN	NaN	NaN	NaN	NaN	NaN	NaN	NaN
Q14739	Lamin-B receptor	LBR	1	2	2	3.1	70.702	4E+07	NaN	3E+07	2.5E+07	3.2E+07	3.1E+07	0.82996	0.3027
P00338	L-lactate dehydrogenase A chain	LDHA	3	7	6	19.3	36.688	1.3E+09	1.6E+09	1.4E+09	1.4E+09	1.3E+09	1.5E+09	0.97794	0.80806
P07195	L-lactate dehydrogenase B chain	LDHB	1	9	8	27.5	36.638	2.2E+09	2.4E+09	2.5E+09	2.4E+09	2.2E+09	2.4E+09	0.98934	0.84273
Q92791	Synaptonemal complex protein SC65	LEPREL4	1	1	1	2.5	50.381	NaN	NaN	NaN	NaN	NaN	NaN	NaN	NaN
O95202	LETM1 and EF-hand domain-containing protein 1, mitochondı	LETM1	1	5	5	7.6	83.353	NaN	1.1E+07	1.3E+07	5964200	1.3E+07	NaN	0.78646	0.55226
P09382	Galectin-1	LGALS1	1	3	3	20.7	14.716	5.8E+08	4E+08	6.7E+08	7.4E+08	5.8E+08	4.7E+08	1.08701	0.69327
Q08380	Galectin-3-binding protein	LGALS3BP	1	1	1	1.9	65.33	NaN	NaN	NaN	NaN	NaN	NaN	NaN	NaN
P48059	LIM and senescent cell antigen-like-containing domain proteir	LIMS1	1	1	1	4	37.251	NaN	NaN	NaN	NaN	NaN	NaN	NaN	NaN
P49257	Protein ERGIC-53	LMAN1	1	5	5	11.8	57.548	5.2E+07	5.7E+07	5.9E+07	4.9E+07	5.1E+07	5.5E+07	0.92152	0.17772
Q12907	Vesicular integral-membrane protein VIP36	LMAN2	1	2	2	5.6	40.228	NaN	3.8E+07	3.8E+07	6.6E+07	NaN	9.7E+07	2.12786	0.10833
Q9NZU5	LIM and cysteine-rich domains protein 1	LMCD1	1	5	5	15.3	40.832	4.4E+07	3.3E+07	4.6E+07	4.6E+07	3.9E+07	4.7E+07	1.07648	0.53923
P02545	Prelamin-A/C;Lamin-A/C	LMNA	1	13	13	19.6	74.139	1.3E+08	1.2E+08	1.3E+08	1.4E+08	1.3E+08	1.4E+08	1.05163	0.16195
Q9UIQ6	Leucyl-cystinyl aminopeptidase;Leucyl-cystinyl aminopeptidase	LNPEP	1	4	4	4.1	117.35	NaN	1.4E+07	NaN	NaN	1.9E+07	NaN	1.37853	NaN
P36776	Lon protease homolog, mitochondrial	LONP1	1	2	2	2.3	106.49	2.4E+07	NaN	1.8E+07	NaN	2.4E+07	3.5E+07	1.41948	0.27845
Q6P1A2	Lysophospholipid acyltransferase 5	LPCAT3	1	2	2	4.5	56.034	2.5E+07	2.5E+07	2.4E+07	2.7E+07	3.1E+07	2.6E+07	1.13565	0.0809
Q93052	Lipoma-preferred partner	LPP	1	1	1	2.3	65.746	NaN	NaN	NaN	NaN	NaN	NaN	NaN	NaN
P50851	Lipopolysaccharide-responsive and beige-like anchor protein	LRBA	2	4	4	1.5	319.1	NaN	NaN	NaN	NaN	NaN	NaN	NaN	NaN
P42704	Leucine-rich PPR motif-containing protein, mitochondrial	LRPPRC	1	22	22	14.2	157.9	1.9E+08	2.1E+08	1.7E+08	2E+08	1.8E+08	1.8E+08	0.99585	0.94973
Q5VZK9	Leucine-rich repeat-containing protein 16A	LRRC16A	1	1	1	0.8	151.56	NaN	NaN	NaN	NaN	NaN	NaN	NaN	NaN
Q8N1G4	Leucine-rich repeat-containing protein 47	LRRC47	1	3	3	7	63.472	3.8E+07	4.5E+07	NaN	3.9E+07	NaN	NaN	0.94525	NaN
Q96AG4	Leucine-rich repeat-containing protein 59	LRRC59	1	2	2	6.5	34.93	NaN	NaN	NaN	NaN	NaN	NaN	NaN	NaN
Q32MZ4	Leucine-rich repeat flightless-interacting protein 1	LRRFIP1	1	2	2	4.1	89.252	3.1E+07	5.5E+07	3E+07	1.5E+07	NaN	NaN	0.40055	NaN
Q9Y608	Leucine-rich repeat flightless-interacting protein 2	LRRFIP2	1	3	3	6	82.17	NaN	NaN	NaN	NaN	NaN	NaN	NaN	NaN
Q8ND56	Protein LSM14 homolog A	LSM14A	1	2	2	4.8	50.529	NaN	NaN	NaN	NaN	NaN	NaN	NaN	NaN
Q9Y4Z0	U6 snRNA-associated Sm-like protein LSm4	LSM4	1	1	1	7.2	15.35	NaN	NaN	NaN	NaN	NaN	NaN	NaN	NaN
P48449	Lanosterol synthase	LSS	1	3	3	4.2	83.308	2.7E+07	2.9E+07	2.5E+07	2.8E+07	2.3E+07	NaN	0.93639	0.54136
P09960	Leukotriene A-4 hydrolase	LTA4H	1	3	3	4.7	69.284	7.6E+07	7.8E+07	6.8E+07	7.9E+07	7.6E+07	NaN	1.04907	0.41419
Q9Y383	Putative RNA-binding protein Luc7-like 2	LUC7L2	2	3	3	7.7	46.513	NaN	1.9E+07	2E+07	2.2E+07	NaN	1.9E+07	1.07036	0.5412
P20645	Cation-dependent mannose-6-phosphate receptor	M6PR	1	2	2	12.6	30.993	NaN	NaN	NaN	NaN	NaN	NaN	NaN	NaN
Q9UPN3	Microtubule-actin cross-linking factor 1, isoforms 1/2/3/5	MACF1	2	6	5	0.9	838.3	1.4E+07	1.2E+07	NaN	1.8E+07	NaN	1.6E+07	1.29406	0.08538
Q9UNF1;Q9Y5V3	Melanoma-associated antigen D2;Melanoma-associated antig	MAGED2	2	1	1	2	64.953	NaN	NaN	NaN	NaN	NaN	NaN	NaN	NaN
P61326;Q96A72	Protein mago nashi homolog;Protein mago nashi homolog 2	MAGOH	2	2	2	19.2	17.163	9.7E+07	8.8E+07	7.4E+07	1.2E+08	7.9E+07	1.1E+08	1.16877	0.33424
Q5VYJ5	MAM and LDL-receptor class A domain-containing protein 1	MALRD1	1	1	1	0.4	241.01	NaN	NaN	NaN	NaN	NaN	NaN	NaN	NaN
O00754	Lysosomal alpha-mannosidase;Lysosomal alpha-mannosidase	MAN2B1	1	2	2	1.9	113.74	NaN	NaN	NaN	NaN	NaN	NaN	NaN	NaN
P55145	Mesencephalic astrocyte-derived neurotrophic factor	MANF	1	2	2	6	20.7	5.7E+07	4.5E+07	2.9E+07	5.3E+07	5.2E+07	5.9E+07	1.25333	0.26243
P46821	Microtubule-associated protein 1B;MAP1B heavy chain;MAP1 l	MAP1B	1	5	5	2.8	270.63	7.9E+07	8.6E+07	8.7E+07	7.5E+07	8.1E+07	7.4E+07	0.91389	0.111
Q9H492;Q9GZQ8;A6N(	Microtubule-associated proteins 1A/1B light chain 3A;Microtul	MAP1LC3A	3	1	1	5.8	14.272	NaN	NaN	NaN	NaN	NaN	NaN	NaN	NaN
Q66K74	Microtubule-associated protein 1S;MAP1S heavy chain;MAP1S	MAP1S	1	1	1	1.3	112.21	NaN	NaN	NaN	NaN	NaN	NaN	NaN	NaN
Q02750	Dual specificity mitogen-activated protein kinase kinase 1	MAP2K1	1	3	3	5.9	43.439	NaN	4.2E+07	4.4E+07	5E+07	5.1E+07	NaN	1.16602	0.01521
P36507	Dual specificity mitogen-activated protein kinase kinase 2	MAP2K2	1	6	6	13.8	44.424	3.4E+07	3.4E+07	3.5E+07	3.1E+07	3.4E+07	3.6E+07	0.97539	0.62219
P46734	Dual specificity mitogen-activated protein kinase kinase 3	MAP2K3	1	1	1	4	39.318	NaN	NaN	NaN	NaN	NaN	NaN	NaN	NaN
P52564	Dual specificity mitogen-activated protein kinase kinase 6	MAP2K6	1	2	2	6.9	37.492	NaN	NaN	NaN	NaN	NaN	NaN	NaN	NaN
P27816	Microtubule-associated protein 4	MAP4	1	2	2	2.7	121	NaN	NaN	NaN	NaN	NaN	NaN	NaN	NaN
O95819	Mitogen-activated protein kinase kinase kinase kinase 4	MAP4K4	1	1	1	0.8	142.1	NaN	NaN	NaN	NaN	NaN	NaN	NaN	NaN
P28482	Mitogen-activated protein kinase 1	MAPK1	1	2	2	4.2	41.389	1.1E+07	2.3E+07	NaN	8319600	7367000	NaN	0.45597	0.27233
P27361	Mitogen-activated protein kinase 3	MAPK3	1	2	2	4	43.135	1.9E+07	1.9E+07	NaN	NaN	NaN	1.8E+07	0.97523	NaN
Q15691	Microtubule-associated protein RP/EB family member 1	MAPRE1	1	3	3	13.8	29.999	1.1E+08	9E+07	9.1E+07	1.1E+08	7.9E+07	1E+08	0.97358	0.81833
Q9UPY8	Microtubule-associated protein RP/EB family member 3	MAPRE3	1	1	1	6.4	31.982	NaN	NaN	NaN	NaN	NaN	NaN	NaN	NaN
P29966	Myristoylated alanine-rich C-kinase substrate	MARCKS	1	1	1	4.5	31.554	NaN	NaN	NaN	NaN	NaN	NaN	NaN	NaN
P49006	MARCKS-related protein	MARCKSL1	1	1	1	7.7	19.529	NaN	NaN	NaN	NaN	NaN	NaN	NaN	NaN

P56192	Methionine--tRNA ligase, cytoplasmic	MARS	1	6	6	7.2	101.11	7.2E+07	7.4E+07	6.4E+07	7.1E+07	5.7E+07	7.2E+07	0.95256	0.58428
P31153	S-adenosylmethionine synthase isoform type-2	MAT2A	2	6	6	16.7	43.66	5.6E+07	6.1E+07	5.9E+07	4.4E+07	5.7E+07	5.3E+07	0.86951	0.12515
Q9NZL9	Methionine adenosyltransferase 2 subunit beta	MAT2B	1	2	2	3.6	37.551	NaN	1.1E+07	NaN	NaN	9614100	NaN	0.84586	NaN
P43243	Matrin-3	MATR3	1	3	3	5.2	94.622	NaN	1E+07	NaN	NaN	6556000	NaN	0.64117	NaN
Q96N66	Lysophospholipid acyltransferase 7	MBOAT7	1	2	2	6.1	52.764	NaN	NaN	NaN	NaN	NaN	NaN	NaN	NaN
Q96RQ3	Methylcrotonoyl-CoA carboxylase subunit alpha, mitochondria	MCCC1	1	1	1	1.8	80.472	NaN	NaN	NaN	NaN	NaN	NaN	NaN	NaN
P49736	DNA replication licensing factor MCM2	MCM2	1	2	2	2.9	101.89	3.1E+07	2.2E+07	NaN	2.1E+07	NaN	2.4E+07	0.835	0.46313
P25205	DNA replication licensing factor MCM3	MCM3	1	2	2	3.1	90.98	2.5E+07	2.5E+07	2.9E+07	3.3E+07	2.7E+07	3E+07	1.13709	0.1818
Q14566	DNA replication licensing factor MCM6	MCM6	1	3	3	4.6	92.888	NaN	1.7E+07	9434000	1.2E+07	1.4E+07	NaN	0.96942	0.92581
P33993	DNA replication licensing factor MCM7	MCM7	1	2	2	3.8	81.307	2.8E+07	NaN	NaN	NaN	NaN	2.6E+07	0.91344	NaN
Q8NE86	Calcium uniporter protein, mitochondrial	MCU	1	1	1	3.7	39.866	1.5E+07	1.3E+07	1.6E+07	1.3E+07	1.7E+07	1.7E+07	1.098	0.40041
P40925	Malate dehydrogenase, cytoplasmic	MDH1	1	3	3	9.6	36.426	6.1E+08	6.1E+08	6.1E+08	6E+08	6.4E+08	7E+08	1.05944	0.26342
P40926	Malate dehydrogenase, mitochondrial	MDH2	1	6	6	17.5	35.503	2.7E+08	3.9E+08	3.3E+08	4.7E+08	1.7E+08	NaN	0.95862	0.91664
P48163	NADP-dependent malic enzyme	ME1	1	3	3	4.2	64.149	NaN	NaN	3E+07	1.9E+07	NaN	2.2E+07	0.69779	NaN
P23368	NAD-dependent malic enzyme, mitochondrial	ME2	1	3	3	4.6	65.443	5.3E+07	4.3E+07	4.2E+07	NaN	5.5E+07	5.4E+07	1.18083	0.1517
Q9Y316	Protein MEMO1	MEMO1	1	1	1	4.4	33.733	NaN	NaN	NaN	NaN	NaN	NaN	NaN	NaN
Q14696	LDLR chaperone MESD	MESDC2	1	2	2	12.8	26.076	1.1E+07	8713600	1.2E+07	NaN	NaN	1.3E+07	1.20047	NaN
P50579	Methionine aminopeptidase 2	METAP2	1	2	2	5.6	52.891	NaN	NaN	NaN	NaN	NaN	NaN	NaN	NaN
Q6UX53	Methyltransferase-like protein 7B	METTL7B	1	1	1	4.1	27.775	NaN	NaN	NaN	NaN	NaN	NaN	NaN	NaN
Q08431	Lactadherin;Lactadherin short form;Medin	MFGE8	1	1	1	2.6	43.104	NaN	NaN	NaN	NaN	NaN	NaN	NaN	NaN
O95140	Mitofusin-2	MFN2	1	1	1	2.2	86.401	NaN	NaN	NaN	NaN	NaN	NaN	NaN	NaN
Q6N075	Molybdate-anion transporter	MFSD5	1	1	1	2.9	49.764	NaN	NaN	NaN	NaN	NaN	NaN	NaN	NaN
Q9BQP7	Mitochondrial genome maintenance exonuclease 1	MGME1	1	1	1	4.1	39.42	NaN	NaN	NaN	NaN	NaN	NaN	NaN	NaN
P10620	Microsomal glutathione S-transferase 1	MGST1	1	2	2	9	17.598	9.6E+07	1E+08	9.6E+07	8.9E+07	1.7E+08	8E+07	1.16573	0.61195
O94851	Protein-methionine sulfoxide oxidase MICAL2	MICAL2	1	1	1	0.5	219.06	NaN	NaN	NaN	NaN	NaN	NaN	NaN	NaN
O15344	E3 ubiquitin-protein ligase Midline-1	MID1	1	3	3	5.8	75.25	1.1E+07	NaN	1.7E+07	9661900	1.2E+07	1.5E+07	0.88241	0.61399
Q96T76	MMS19 nucleotide excision repair protein homolog	MMS19	1	1	1	1.1	113.29	NaN	NaN	NaN	NaN	NaN	NaN	NaN	NaN
Q9H8S9;Q7L9L4	MOB kinase activator 1A;MOB kinase activator 1B	MOB1A	2	2	2	11.1	25.079	1.2E+07	NaN	1.7E+07	1.4E+07	1.4E+07	NaN	0.95057	0.82767
Q70IA6	MOB kinase activator 2	MOB2	1	1	1	4.6	26.926	NaN	NaN	NaN	NaN	NaN	NaN	NaN	NaN
Q9Y3A3	MOB-like protein phocein	MOB4	1	1	1	6.7	26.032	NaN	NaN	NaN	NaN	NaN	NaN	NaN	NaN
Q13724	Mannosyl-oligosaccharide glucosidase	MOGS	1	4	4	4.7	91.916	6.2E+07	NaN	NaN	NaN	6E+07	NaN	0.97712	NaN
Q7Z3U7	Protein MON2 homolog	MON2	1	1	1	0.6	190.36	NaN	NaN	NaN	NaN	NaN	NaN	NaN	NaN
O75352	Mannose-P-dolichol utilization defect 1 protein	MPDU1	1	2	2	8.1	26.638	6.6E+07	6.3E+07	6.1E+07	6.7E+07	5.3E+07	5.3E+07	0.91497	0.33184
P49959	Double-strand break repair protein MRE11A	MRE11A	1	2	2	3.5	80.592	NaN	NaN	NaN	NaN	NaN	NaN	NaN	NaN
Q7Z745	Maestro heat-like repeat-containing protein family member 2I	MROH2B	1	1	1	0.5	180.78	NaN	NaN	NaN	NaN	NaN	NaN	NaN	NaN
P52815	39S ribosomal protein L12, mitochondrial	MRPL12	1	3	3	10.1	21.348	2.9E+08	2.5E+08	2.1E+08	2E+08	2.8E+08	NaN	0.96861	0.86808
Q9BYD1	39S ribosomal protein L13, mitochondrial	MRPL13	1	3	3	14	20.692	5.2E+07	4.8E+07	NaN	4.2E+07	3.9E+07	4.1E+07	0.80825	0.01725
Q9NX20	39S ribosomal protein L16, mitochondrial	MRPL16	1	2	2	8.4	28.449	NaN	NaN	NaN	NaN	NaN	NaN	NaN	NaN
Q9NRX2	39S ribosomal protein L17, mitochondrial	MRPL17	1	1	1	4	20.05	NaN	NaN	NaN	NaN	NaN	NaN	NaN	NaN
P49406	39S ribosomal protein L19, mitochondrial	MRPL19	1	1	1	3.1	33.535	NaN	NaN	NaN	NaN	NaN	NaN	NaN	NaN
Q9NWU5	39S ribosomal protein L22, mitochondrial	MRPL22	1	1	1	6.3	23.64	NaN	NaN	NaN	NaN	NaN	NaN	NaN	NaN
Q13084	39S ribosomal protein L28, mitochondrial	MRPL28	1	2	2	9.8	30.156	9506800	1.1E+07	NaN	1.2E+07	NaN	NaN	1.1589	NaN
Q8TCC3	39S ribosomal protein L30, mitochondrial	MRPL30	1	2	2	13	18.546	NaN	NaN	NaN	NaN	NaN	NaN	NaN	NaN
Q9NZE8	39S ribosomal protein L35, mitochondrial	MRPL35	1	1	1	5.3	21.514	NaN	NaN	NaN	NaN	NaN	NaN	NaN	NaN
Q96DV4	39S ribosomal protein L38, mitochondrial	MRPL38	1	3	3	10.8	44.596	1.3E+07	1.2E+07	1.4E+07	1E+07	1.3E+07	NaN	0.88328	0.31454
Q9BYD3	39S ribosomal protein L4, mitochondrial	MRPL4	1	2	2	9.3	34.919	9295600	7084200	NaN	8982800	7965100	8777200	1.04703	0.70263
Q9Y6G3	39S ribosomal protein L42, mitochondrial	MRPL42	1	1	1	11.3	16.661	1.6E+07	NaN	NaN	1.8E+07	1.7E+07	NaN	1.06021	NaN
Q8N983	39S ribosomal protein L43, mitochondrial	MRPL43	1	1	1	5.1	23.431	NaN	NaN	NaN	NaN	NaN	NaN	NaN	NaN
Q13405	39S ribosomal protein L49, mitochondrial	MRPL49	1	1	1	7.8	19.198	NaN	NaN	NaN	NaN	NaN	NaN	NaN	NaN
Q8N5N7	39S ribosomal protein L50, mitochondrial	MRPL50	1	1	1	7	18.325	NaN	NaN	NaN	NaN	NaN	NaN	NaN	NaN
Q96EL3	39S ribosomal protein L53, mitochondrial	MRPL53	1	1	1	13.4	12.107	NaN	NaN	NaN	NaN	NaN	NaN	NaN	NaN
Q9BYD2	39S ribosomal protein L9, mitochondrial	MRPL9	1	1	1	3.7	30.243	NaN	NaN	NaN	NaN	NaN	NaN	NaN	NaN
O60783	28S ribosomal protein S14, mitochondrial	MRPS14	1	1	1	11.7	15.139	NaN	NaN	NaN	NaN	NaN	NaN	NaN	NaN
Q9Y2R5	28S ribosomal protein S17, mitochondrial	MRPS17	1	1	1	6.9	14.502	NaN	NaN	NaN	NaN	NaN	NaN	NaN	NaN
Q9NVS2	28S ribosomal protein S18a, mitochondrial	MRPS18A	1	1	1	8.2	22.184	NaN	NaN	NaN	NaN	NaN	NaN	NaN	NaN
P82673	28S ribosomal protein S35, mitochondrial	MRPS35	1	1	1	4.6	36.844	NaN	NaN	NaN	NaN	NaN	NaN	NaN	NaN
Q9Y2R9	28S ribosomal protein S7, mitochondrial	MRPS7	1	1	1	4.5	28.134	NaN	NaN	NaN	NaN	NaN	NaN	NaN	NaN
P82933	28S ribosomal protein S9, mitochondrial	MRPS9	1	2	2	4.5	45.834	NaN	NaN	NaN	NaN	NaN	NaN	NaN	NaN
Q96E11	Ribosome-recycling factor, mitochondrial	MRRF	1	1	1	6.1	29.277	NaN	NaN	NaN	NaN	NaN	NaN	NaN	NaN

P43246	DNA mismatch repair protein Msh2	MSH2	1	5	5	6.7	104.74	2.8E+07	NaN	3.3E+07	NaN	3.3E+07	2.9E+07	1.01531	0.90522
P52701	DNA mismatch repair protein Msh6	MSH6	1	8	8	7.1	152.78	4E+07	4.1E+07	4.7E+07	3.6E+07	3.8E+07	3.6E+07	0.8572	0.05978
P26038	Moesin	MSN	1	20	13	23.4	67.819	7.3E+08	6.6E+08	6.3E+08	8E+08	7.5E+08	7.6E+08	1.14737	0.03684
P00403	Cytochrome c oxidase subunit 2	MT-CO2	1	1	1	4.4	25.565	NaN	NaN	NaN	NaN	NaN	NaN	NaN	NaN
Q86UE4	Protein LYRIC	MTDH	1	3	3	6.7	63.836	NaN	NaN	NaN	NaN	NaN	NaN	NaN	NaN
P11586	C-1-tetrahydrofolate synthase, cytoplasmic;Methylenetetrahyd	MTHFD1	1	9	9	10.6	101.53	1.7E+08	2.1E+08	1.8E+08	1.6E+08	1.6E+08	1.6E+08	0.8767	0.11824
Q6UB35	Monofunctional C1-tetrahydrofolate synthase, mitochondrial	MTHFD1L	1	2	2	2.6	105.79	NaN	NaN	NaN	NaN	NaN	NaN	NaN	NaN
P49914	5-formyltetrahydrofolate cyclo-ligase	MTHFS	1	2	2	12.8	23.255	1.9E+07	2.9E+07	3.2E+07	NaN	3E+07	2.9E+07	1.10855	0.61537
P03897	NADH-ubiquinone oxidoreductase chain 3	MT-ND3	1	1	1	13	13.186	NaN	NaN	NaN	NaN	NaN	NaN	NaN	NaN
P03905	NADH-ubiquinone oxidoreductase chain 4	MT-ND4	1	1	1	2	51.58	NaN	NaN	NaN	NaN	NaN	NaN	NaN	NaN
P58546	Myotrophin	MTPN	1	1	1	11	12.895	NaN	NaN	NaN	NaN	NaN	NaN	NaN	NaN
Q13505	Metaxin-1	MTX1	1	1	1	3	51.462	NaN	NaN	NaN	NaN	NaN	NaN	NaN	NaN
O75431	Metaxin-2	MTX2	1	1	1	4.9	29.763	NaN	NaN	NaN	NaN	NaN	NaN	NaN	NaN
Q14764	Major vault protein	MVP	1	7	7	10.1	99.326	1.2E+08	1.3E+08	1.2E+08	1.1E+08	1.1E+08	1.3E+08	0.9314	0.19448
Q9BQG0	Myb-binding protein 1A	MYBBP1A	1	2	2	1.4	148.85	NaN	NaN	NaN	NaN	NaN	NaN	NaN	NaN
Q99836	Myeloid differentiation primary response protein MyD88	MYD88	1	2	2	6.8	33.233	NaN	NaN	NaN	NaN	NaN	NaN	NaN	NaN
P35580	Myosin-10	MYH10	1	9	5	5.6	229	3.4E+07	3.3E+07	NaN	2.6E+07	2.7E+07	4.1E+07	0.93345	0.74616
P35579	Myosin-9	MYH9	11	38	34	19.4	226.53	6.7E+08	8E+08	7.5E+08	6.8E+08	6.7E+08	7.3E+08	0.93749	0.31893
P19105;O14950	Myosin regulatory light chain 12A;Myosin regulatory light chai	MYL12A	2	2	1	12.9	19.794	3.1E+08	NaN	3.7E+08	2.5E+08	2.8E+08	3.9E+08	0.90106	0.59715
P60660	Myosin light polypeptide 6	MYL6	1	5	4	31.8	16.93	1.9E+08	1.5E+08	2.1E+08	1.3E+08	1.5E+08	2E+08	0.85994	0.41338
Q9UKN7	Unconventional myosin-XV	MYO15A	1	1	1	0.3	395.29	NaN	NaN	NaN	NaN	NaN	NaN	NaN	NaN
Q92614	Unconventional myosin-XVIIIa	MYO18A	1	2	2	1.2	233.11	NaN	NaN	NaN	NaN	NaN	NaN	NaN	NaN
O00159	Unconventional myosin-Ic	MYO1C	1	5	5	4.4	121.68	7.6E+07	6.3E+07	7.7E+07	7.1E+07	6.1E+07	6.6E+07	0.91096	0.29806
Q12965;O00160	Unconventional myosin-Ie;Unconventional myosin-If	MYO1E	2	2	2	2.1	127.06	NaN	NaN	NaN	NaN	NaN	NaN	NaN	NaN
Q9UM54	Unconventional myosin-VI	MYO6	1	3	3	2.6	149.69	1.6E+07	1.5E+07	1.5E+07	1.6E+07	1.4E+07	1.4E+07	0.96375	0.50285
Q9NZM1	Myoferlin	MYOF	2	33	33	18.4	234.71	1.4E+08	1.4E+08	1.4E+08	1.3E+08	1.4E+08	1.4E+08	0.96972	0.38093
Q9BSU3;P41227	N-alpha-acetyltransferase 11;N-alpha-acetyltransferase 10	NAA11	2	1	1	5.2	25.979	NaN	NaN	NaN	NaN	NaN	NaN	NaN	NaN
Q9BXJ9	N-alpha-acetyltransferase 15, NatA auxiliary subunit	NAA15	2	5	5	7.5	101.27	5.8E+07	4.5E+07	NaN	NaN	4E+07	4.8E+07	0.85861	0.43316
Q14CX7	N-alpha-acetyltransferase 25, NatB auxiliary subunit	NAA25	1	4	4	5.1	112.29	NaN	9112400	NaN	1.2E+07	9341100	NaN	1.14647	NaN
Q9GZZ1	N-alpha-acetyltransferase 50	NAA50	1	4	4	24.9	19.398	NaN	2.6E+07	1.7E+07	2E+07	1.9E+07	1.6E+07	0.8571	0.49403
Q9BZK3;Q13765;E9PAV	Putative nascent polypeptide-associated complex subunit alph	NACAP1	3	1	1	6.1	23.306	NaN	NaN	NaN	NaN	NaN	NaN	NaN	NaN
Q13564	NEDD8-activating enzyme E1 regulatory subunit	NAE1	1	2	2	3.7	60.246	3.9E+07	3.9E+07	3.6E+07	4E+07	3.7E+07	4.2E+07	1.0395	0.42303
Q9UJ70	N-acetyl-D-glucosamine kinase	NAGK	1	2	2	6.7	37.375	2.9E+07	4.4E+07	3.3E+07	NaN	3E+07	1.2E+07	0.60294	0.21122
P43490	Nicotinamide phosphoribosyltransferase	NAMPT	1	8	8	16.1	55.52	2.1E+08	2.5E+08	2.3E+08	2.2E+08	2.5E+08	1.7E+08	0.93324	0.56002
Q9NR45	Sialic acid synthase	NANS	1	4	4	14.2	40.307	2.8E+07	2.5E+07	2.7E+07	1.8E+07	2.7E+07	2.9E+07	0.92579	0.59708
P55209;Q99733	Nucleosome assembly protein 1-like 1;Nucleosome assembly	NAP1L1	2	2	2	6.9	45.374	1E+08	1.1E+08	1.6E+08	9.8E+07	9.8E+07	5E+07	0.6564	0.14962
Q6XQN6	Nicotinate phosphoribosyltransferase	NAPRT	1	1	1	1.9	57.578	NaN	NaN	NaN	NaN	NaN	NaN	NaN	NaN
O43776	Asparagine--tRNA ligase, cytoplasmic	NARS	1	5	5	11.9	62.942	1.8E+08	1.6E+08	1.8E+08	1.6E+08	2E+08	1.3E+08	0.9458	0.65591
P49321	Nuclear autoantigenic sperm protein	NASP	1	2	2	1.6	85.237	NaN	NaN	NaN	NaN	NaN	NaN	NaN	NaN
Q6ZNJ1	Neurobeachin-like protein 2	NBEAL2	1	2	2	1.1	302.51	NaN	NaN	NaN	NaN	NaN	NaN	NaN	NaN
Q15021	Condensin complex subunit 1	NCAPD2	1	5	5	4.9	157.18	NaN	NaN	NaN	NaN	NaN	NaN	NaN	NaN
Q9BPX3	Condensin complex subunit 3	NCAPG	1	1	1	1.8	114.33	NaN	NaN	NaN	NaN	NaN	NaN	NaN	NaN
Q6PIU2	Neutral cholesterol ester hydrolase 1	NCEH1	1	1	1	2.9	45.807	NaN	NaN	NaN	NaN	NaN	NaN	NaN	NaN
P16333	Cytoplasmic protein NCK1	NCK1	1	1	1	5.8	42.864	NaN	NaN	NaN	NaN	NaN	NaN	NaN	NaN
Q9Y2A7	Nck-associated protein 1	NCKAP1	1	2	2	1.7	128.79	NaN	NaN	NaN	NaN	NaN	NaN	NaN	NaN
P19338	Nucleolin	NCL	1	11	11	12.1	76.613	2.1E+08	1.9E+08	2.3E+08	1.9E+08	2.2E+08	2.1E+08	0.98873	0.88852
Q969V3	Nicalin	NCLN	1	3	3	6	62.974	5.3E+07	NaN	NaN	NaN	6E+07	5.7E+07	1.09663	NaN
Q92597	Protein NDRG1	NDRG1	1	1	1	3.8	42.835	NaN	NaN	NaN	NaN	NaN	NaN	NaN	NaN
Q9UGV2	Protein NDRG3	NDRG3	1	2	2	8	41.408	NaN	NaN	NaN	NaN	NaN	NaN	NaN	NaN
Q9P0J0	NADH dehydrogenase [ubiquinone] 1 alpha subcomplex subu	NDUFA13	1	3	3	24.3	16.698	2.8E+07	NaN	NaN	NaN	3.1E+07	3.7E+07	1.22376	NaN
O43678	NADH dehydrogenase [ubiquinone] 1 alpha subcomplex subu	NDUFA2	1	1	1	10.1	10.921	NaN	NaN	NaN	NaN	NaN	NaN	NaN	NaN
Q16718	NADH dehydrogenase [ubiquinone] 1 alpha subcomplex subu	NDUFA5	1	1	1	8.6	13.459	NaN	NaN	NaN	NaN	NaN	NaN	NaN	NaN
P51970	NADH dehydrogenase [ubiquinone] 1 alpha subcomplex subu	NDUFA8	1	1	1	7.6	20.105	NaN	NaN	NaN	NaN	NaN	NaN	NaN	NaN
Q16795	NADH dehydrogenase [ubiquinone] 1 alpha subcomplex subu	NDUFA9	1	1	1	2.7	42.509	NaN	NaN	NaN	NaN	NaN	NaN	NaN	NaN
O75438	NADH dehydrogenase [ubiquinone] 1 beta subcomplex subun	NDUFB1	1	1	1	19	6.9611	NaN	NaN	NaN	NaN	NaN	NaN	NaN	NaN
O96000	NADH dehydrogenase [ubiquinone] 1 beta subcomplex subun	NDUFB10	1	2	2	16.3	20.776	NaN	1.5E+07	NaN	NaN	NaN	1.4E+07	0.94481	NaN
Q9NX14	NADH dehydrogenase [ubiquinone] 1 beta subcomplex subun	NDUFB11	1	1	1	15	17.316	NaN	NaN	NaN	NaN	NaN	NaN	NaN	NaN
O43676	NADH dehydrogenase [ubiquinone] 1 beta subcomplex subun	NDUFB3	1	2	2	18.4	11.402	NaN	NaN	NaN	NaN	NaN	NaN	NaN	NaN
O95168	NADH dehydrogenase [ubiquinone] 1 beta subcomplex subun	NDUFB4	1	2	2	21.7	15.208	1.4E+07	2.3E+07	1.5E+07	1.8E+07	1.5E+07	1.5E+07	0.94414	0.76679

O95139	NADH dehydrogenase [ubiquinone] 1 beta subcomplex subun	NDUFB6	1	1	1	14.1	15.489	NaN	NaN	NaN	NaN	NaN	NaN	NaN	NaN
Q9Y6M9	NADH dehydrogenase [ubiquinone] 1 beta subcomplex subun	NDUFB9	1	3	3	24	21.831	1.2E+07	1.6E+07	1.2E+07	9057400	1E+07	1.3E+07	0.80428	0.19076
P28331	NADH-ubiquinone oxidoreductase 75 kDa subunit, mitochond	NDUFS1	1	4	4	7.4	79.467	1.9E+07	1.7E+07	1.8E+07	2.3E+07	2E+07	2.3E+07	1.21861	0.03319
O75306	NADH dehydrogenase [ubiquinone] iron-sulfur protein 2, mitc	NDUFS2	1	2	2	6.7	52.545	NaN	NaN	NaN	NaN	NaN	NaN	NaN	NaN
O00217	NADH dehydrogenase [ubiquinone] iron-sulfur protein 8, mitc	NDUFS8	1	1	1	5.2	23.705	NaN	NaN	NaN	NaN	NaN	NaN	NaN	NaN
P49821	NADH dehydrogenase [ubiquinone] flavoprotein 1, mitochond	NDUFV1	1	2	2	4.3	50.817	5E+07	6.8E+07	NaN	5.2E+07	4.7E+07	4.4E+07	0.80843	0.22249
P19404	NADH dehydrogenase [ubiquinone] flavoprotein 2, mitochond	NDUFV2	1	2	2	12.9	27.391	NaN	NaN	NaN	NaN	NaN	NaN	NaN	NaN
Q15843	NEDD8	NEDD8	1	3	3	28.4	9.0714	NaN	7E+07	1.2E+08	3.4E+07	4.9E+07	4.9E+07	0.46086	0.0799
Q8TD19	Serine/threonine-protein kinase Nek9	NEK9	1	1	1	1.7	107.17	NaN	2.9E+07	NaN	NaN	2.6E+07	NaN	0.91395	NaN
Q9UMX5	Neudesin	NENF	1	2	2	11.6	18.856	NaN	NaN	NaN	NaN	NaN	NaN	NaN	NaN
P08651	Nuclear factor 1 C-type	NFIC	1	1	1	2.8	55.674	NaN	NaN	NaN	NaN	NaN	NaN	NaN	NaN
Q00653	Nuclear factor NF-kappa-B p100 subunit;Nuclear factor NF-ka	NFKB2	1	4	4	6.8	96.748	1.3E+07	NaN	1.2E+07	1.2E+07	1.1E+07	NaN	0.89647	0.29879
Q13952	Nuclear transcription factor Y subunit gamma	NFYC	1	1	1	1.7	50.302	NaN	NaN	NaN	NaN	NaN	NaN	NaN	NaN
Q9BPW8	Protein NipSnap homolog 1	NIPSNAP1	1	1	1	4.9	33.31	NaN	NaN	NaN	NaN	NaN	NaN	NaN	NaN
Q9NQR4	Omega-amidase NIT2	NIT2	1	1	1	5.4	30.608	NaN	NaN	NaN	NaN	NaN	NaN	NaN	NaN
Q9BYT8	Neurolysin, mitochondrial	NLN	1	3	3	5.5	80.651	NaN	NaN	NaN	NaN	NaN	NaN	NaN	NaN
Q9NX02	NACHT, LRR and PYD domains-containing protein 2	NLRP2	2	4	4	4.1	120.51	NaN	NaN	NaN	NaN	NaN	NaN	NaN	NaN
Q96D46	60S ribosomal export protein NMD3	NMD3	1	2	2	3.4	57.603	NaN	NaN	NaN	NaN	NaN	NaN	NaN	NaN
P15531	Nucleoside diphosphate kinase A	NME1	1	6	1	29.6	17.149	NaN	NaN	3.1E+08	NaN	NaN	5.1E+08	1.63755	NaN
P22392	Nucleoside diphosphate kinase B	NME2	1	7	0	38.2	17.298	9.8E+08	9.7E+08	8E+08	1E+09	1E+09	1E+09	1.12825	0.11234
Q13232	Nucleoside diphosphate kinase 3	NME3	1	1	1	10.1	19.015	NaN	NaN	NaN	NaN	NaN	NaN	NaN	NaN
P30419	Glycylpeptide N-tetradecanoyltransferase 1	NMT1	1	1	1	3.8	56.806	NaN	NaN	NaN	NaN	NaN	NaN	NaN	NaN
P40261	Nicotinamide N-methyltransferase	NNMT	1	2	2	6.4	29.574	5.6E+08	1.7E+08	3.6E+08	5.5E+08	2.5E+08	3.9E+08	1.10298	0.80495
Q14978	Nucleolar and coiled-body phosphoprotein 1	NOLC1	1	1	1	2	73.602	NaN	NaN	NaN	NaN	NaN	NaN	NaN	NaN
P69849;Q15155;Q5JPE7	Nodal modulator 3;Nodal modulator 1;Nodal modulator 2	NOMO3	3	1	1	1.2	134.13	NaN	NaN	NaN	NaN	NaN	NaN	NaN	NaN
Q15233	Non-POU domain-containing octamer-binding protein	NONO	1	9	7	19.7	54.231	1.5E+08	1.5E+08	1.4E+08	1.5E+08	1.5E+08	1.4E+08	1.0259	0.4029
O15118	Niemann-Pick C1 protein	NPC1	1	3	3	2.7	142.17	4.3E+07	3.5E+07	3.5E+07	4.3E+07	4.4E+07	4.7E+07	1.19058	0.08106
P55786	Puromycin-sensitive aminopeptidase	NPEPPS	2	8	8	9.1	103.28	8.1E+07	6.8E+07	7.4E+07	6.9E+07	9E+07	8.3E+07	1.08795	0.42325
Q8TAT6	Nuclear protein localization protein 4 homolog	NPLOC4	1	1	1	1.8	68.119	NaN	NaN	NaN	NaN	NaN	NaN	NaN	NaN
P06748	Nucleophosmin	NPM1	1	5	5	17.7	32.575	4.9E+08	5.6E+08	4.9E+08	5.5E+08	5.4E+08	4.6E+08	1.00689	0.92861
P15559	NAD(P)H dehydrogenase [quinone] 1	NQO1	1	4	4	14.6	30.867	1.2E+09	1.3E+09	1.2E+09	1.5E+09	1.5E+09	1.2E+09	1.14107	0.20247
Q9Y5X4	Photoreceptor-specific nuclear receptor	NR2E3	1	1	1	4.1	44.692	NaN	NaN	NaN	NaN	NaN	NaN	NaN	NaN
Q92823	Neuronal cell adhesion molecule	NRCAM	1	2	2	1.8	143.89	NaN	NaN	NaN	NaN	NaN	NaN	NaN	NaN
O43847	Nardilysin	NRD1	1	4	4	3.9	131.7	2.5E+07	2.2E+07	2.8E+07	2.8E+07	2.6E+07	2.2E+07	1.00574	0.95472
O14786	Neuropilin-1	NRP1	1	1	1	1.7	103.13	NaN	NaN	NaN	NaN	NaN	NaN	NaN	NaN
O60462	Neuropilin-2	NRP2	1	2	2	1.9	104.83	1.2E+07	NaN	1.5E+07	NaN	NaN	NaN	NaN	NaN
P46459	Vesicle-fusing ATPase	NSF	1	4	4	5.2	82.593	3E+07	1.6E+07	2.5E+07	2.2E+07	2.3E+07	2.5E+07	0.99723	0.98847
Q9UNZ2	NSFL1 cofactor p47	NSFL1C	1	4	4	12.4	40.572	1.4E+08	9.7E+07	1.5E+08	9.7E+07	1.1E+08	1.1E+08	0.83377	0.24847
Q08J23	tRNA (cytosine(34)-C(5))-methyltransferase	NSUN2	1	6	6	10.3	86.47	2.9E+07	2.8E+07	2.6E+07	2E+07	2.6E+07	2.4E+07	0.85114	0.10914
Q8TEA1	Putative methyltransferase NSUN6	NSUN6	1	1	1	2.3	51.77	NaN	NaN	NaN	NaN	NaN	NaN	NaN	NaN
Q9H0P0	Cytosolic 5-nucleotidase 3A	NT5C3A	1	1	1	3	37.948	NaN	NaN	NaN	NaN	NaN	NaN	NaN	NaN
Q5TFE4	5-nucleotidase domain-containing protein 1	NT5DC1	1	1	1	2.2	51.844	NaN	NaN	NaN	NaN	NaN	NaN	NaN	NaN
Q86UY8	5-nucleotidase domain-containing protein 3	NT5DC3	1	1	1	1.6	63.419	NaN	NaN	NaN	NaN	NaN	NaN	NaN	NaN
Q9Y5A7	NEDD8 ultimate buster 1	NUB1	1	1	1	2	70.537	NaN	NaN	NaN	NaN	NaN	NaN	NaN	NaN
Q02818	Nucleobindin-1	NUCB1	1	1	1	2.8	53.879	NaN	NaN	NaN	NaN	NaN	NaN	NaN	NaN
P80303	Nucleobindin-2;Nesfatin-1	NUCB2	1	2	2	7.6	50.222	NaN	7733400	NaN	NaN	8827000	NaN	1.14141	NaN
Q9Y266	Nuclear migration protein nudC	NUDC	1	4	4	13.3	38.242	3.3E+07	4E+07	2.8E+07	2.9E+07	2.8E+07	3.6E+07	0.91588	0.53138
P50583	Bis(5-nucleosyl)-tetraphosphatase [asymmetrical]	NUDT2	1	2	2	23.8	16.829	NaN	NaN	1.5E+07	NaN	1.6E+07	NaN	1.01207	NaN
O43809	Cleavage and polyadenylation specificity factor subunit 5	NUDT21	1	2	2	7.9	26.227	9.5E+07	2.3E+08	1.1E+08	9.7E+07	9.4E+07	1.3E+08	0.73063	0.41937
Q9UKK9	ADP-sugar pyrophosphatase	NUDT5	1	3	3	17.4	24.327	1.1E+08	1E+08	1.2E+08	1.1E+08	1.2E+08	1E+08	1.01118	0.87157
Q9BW91	ADP-ribose pyrophosphatase, mitochondrial	NUDT9	1	1	1	4.9	39.125	NaN	NaN	NaN	NaN	NaN	NaN	NaN	NaN
O75694	Nuclear pore complex protein Nup155	NUP155	1	2	2	1.8	155.2	NaN	NaN	NaN	NaN	NaN	NaN	NaN	NaN
Q92621	Nuclear pore complex protein Nup205	NUP205	1	2	2	1	227.92	NaN	NaN	NaN	NaN	NaN	NaN	NaN	NaN
Q7Z3B4	Nucleoporin p54	NUP54	1	1	1	2.6	55.435	NaN	NaN	NaN	NaN	NaN	NaN	NaN	NaN
Q8N1F7	Nuclear pore complex protein Nup93	NUP93	1	2	2	4.5	93.487	8177600	8580000	9336600	8730800	7262700	8922600	0.95485	0.56372
Q6DKJ4	Nucleoredoxin	NXN	1	2	2	5.1	48.392	NaN	NaN	NaN	NaN	NaN	NaN	NaN	NaN
P00973	2-5-oligoadenylate synthase 1	OAS1	1	1	1	4.8	46.028	NaN	NaN	NaN	NaN	NaN	NaN	NaN	NaN
Q9Y6K5	2-5-oligoadenylate synthase 3	OAS3	1	2	2	2.5	121.17	NaN	NaN	NaN	NaN	NaN	NaN	NaN	NaN
P04181	Ornithine aminotransferase, mitochondrial;Ornithine aminotrar	OAT	1	4	4	10.9	48.534	NaN	NaN	NaN	4.1E+07	5.1E+07	NaN	NaN	NaN

Q9NX40	OCIA domain-containing protein 1	OCIAD1	1	2	2	4.9	27.626	1E+08	1E+08	NaN	6.6E+07	5.7E+07	6.2E+07	0.59399	0.00127
Q56VL3	OCIA domain-containing protein 2	OCIAD2	1	1	1	5.8	16.953	NaN	NaN	NaN	NaN	NaN	NaN	NaN	NaN
Q01968	Inositol polyphosphate 5-phosphatase OCRL-1	OCRL	1	1	1	1.3	104.2	NaN	NaN	NaN	NaN	NaN	NaN	NaN	NaN
Q5SWX8	Protein odr-4 homolog	ODR4	1	1	1	4	51.103	NaN	NaN	NaN	NaN	NaN	NaN	NaN	NaN
Q02218	2-oxoglutarate dehydrogenase, mitochondrial	OGDH	1	7	7	6.4	115.93	2.8E+07	3.2E+07	2.9E+07	3.5E+07	3E+07	3E+07	1.06434	0.42889
Q9NZT2	Opioid growth factor receptor	OGFR	1	1	1	7.7	73.324	NaN	NaN	NaN	NaN	NaN	NaN	NaN	NaN
O15294	UDP-N-acetylglucosamine--peptide N-acetylglucosaminyltrar	OGT	1	3	3	3.2	116.92	NaN	NaN	NaN	1.1E+07	NaN	1.5E+07	NaN	NaN
Q9NTK5	Obg-like ATPase 1	OLA1	1	6	6	19.7	44.743	4.9E+07	4.7E+07	4.1E+07	4.1E+07	4.1E+07	3.8E+07	0.87333	0.08457
O60313	Dynamin-like 120 kDa protein, mitochondrial;Dynamin-like 12	OPA1	1	3	3	2.9	111.63	1.7E+07	1.9E+07	NaN	NaN	2E+07	NaN	1.14147	NaN
P22059	Oxysterol-binding protein 1	OSBP	1	4	4	6.4	89.42	3.1E+07	3E+07	4.3E+07	3E+07	2.9E+07	3.2E+07	0.87151	0.35088
Q9H4L5	Oxysterol-binding protein-related protein 3	OSBPL3	1	1	1	1.5	101.22	NaN	NaN	NaN	NaN	NaN	NaN	NaN	NaN
Q9NPF4	Probable tRNA N6-adenosine threonylcarbamoyltransferase	OSGEP	1	1	1	5.1	36.426	NaN	NaN	NaN	NaN	NaN	NaN	NaN	NaN
Q96FW1	Ubiquitin thioesterase OTUB1	OTUB1	1	2	2	10.3	31.284	NaN	NaN	NaN	NaN	NaN	NaN	NaN	NaN
P13674	Prolyl 4-hydroxylase subunit alpha-1	P4HA1	1	4	4	9.4	61.049	2.3E+07	2.7E+07	2.4E+07	1.5E+07	2.3E+07	2E+07	0.79046	0.11741
O15460	Prolyl 4-hydroxylase subunit alpha-2	P4HA2	1	2	2	4.5	60.901	NaN	NaN	NaN	NaN	NaN	NaN	NaN	NaN
Q7Z4N8	Prolyl 4-hydroxylase subunit alpha-3	P4HA3	1	1	1	2.9	61.126	NaN	NaN	NaN	NaN	NaN	NaN	NaN	NaN
P07237	Protein disulfide-isomerase	P4HB	1	11	11	19.3	57.116	4.5E+08	4E+08	4.9E+08	4.8E+08	4.2E+08	4.4E+08	1.0042	0.9554
Q9UQ80	Proliferation-associated protein 2G4	PA2G4	1	3	3	6.9	43.786	8E+07	3.7E+08	1.2E+08	1.6E+08	7.7E+07	1.3E+08	0.664	0.5366
P11940	Polyadenylate-binding protein 1	PABPC1	4	3	2	4.6	70.67	5.8E+07	3.7E+07	6.3E+07	6.4E+07	5.2E+07	2.8E+07	0.90373	0.72043
Q9UNF0	Protein kinase C and casein kinase substrate in neurons protein	PACSIN2	1	4	4	8.2	55.738	5E+07	5E+07	NaN	5.9E+07	5.5E+07	NaN	1.15176	0.05489
P43034	Platelet-activating factor acetylhydrolase IB subunit alpha	PAFAH1B1	1	4	4	9.5	46.637	8.2E+07	NaN	9.5E+07	NaN	7.2E+07	6.8E+07	0.78519	0.10582
P68402	Platelet-activating factor acetylhydrolase IB subunit beta	PAFAH1B2	1	1	1	4.4	25.569	NaN	NaN	NaN	NaN	NaN	NaN	NaN	NaN
Q15102	Platelet-activating factor acetylhydrolase IB subunit gamma	PAFAH1B3	1	6	6	32	25.734	8.8E+07	9.3E+07	7.7E+07	1.2E+08	9.5E+07	9.3E+07	1.20003	0.18854
Q99487	Platelet-activating factor acetylhydrolase 2, cytoplasmic	PAFAH2	1	1	1	3.3	44.035	NaN	NaN	NaN	NaN	NaN	NaN	NaN	NaN
P22234	Multifunctional protein ADE2;Phosphoribosylaminoimidazole-s	PAICS	1	4	4	7.5	47.079	1.3E+08	9.1E+07	1.1E+08	1.1E+08	9.9E+07	1.1E+08	0.96936	0.78016
Q9H074	Polyadenylate-binding protein-interacting protein 1	PAIP1	1	2	2	4.2	53.524	5.4E+07	6E+07	5.1E+07	4E+07	4.7E+07	5E+07	0.83044	0.07229
Q13177	Serine/threonine-protein kinase PAK 2;PAK-2p27;PAK-2p34	PAK2	1	1	1	3.8	58.042	NaN	NaN	NaN	NaN	NaN	NaN	NaN	NaN
Q8WX93	Palladin	PALLD	1	1	1	0.6	150.56	NaN	NaN	NaN	NaN	NaN	NaN	NaN	NaN
P51003;Q9NRJ5	Poly(A) polymerase alpha;Poly(A) polymerase beta	PAPOLA	2	2	2	3.5	82.842	NaN	NaN	NaN	NaN	NaN	NaN	NaN	NaN
O95340	Bifunctional 3-phosphoadenosine 5-phosphosulfate synthase	PAPSS2	1	3	3	5.5	69.5	NaN	NaN	NaN	NaN	NaN	NaN	NaN	NaN
Q99497	Protein deglycase DJ-1	PARK7	1	2	2	15.9	19.891	2.5E+08	2.1E+08	2E+08	2.3E+08	2.5E+08	2.2E+08	1.04815	0.57221
P09874	Poly [ADP-ribose] polymerase 1	PARP1	1	5	5	5.9	113.08	2.7E+07	2.7E+07	3.1E+07	3.4E+07	2.6E+07	2.9E+07	1.05074	0.62885
Q9UKK3	Poly [ADP-ribose] polymerase 4	PARP4	1	2	2	1.4	192.59	NaN	NaN	NaN	NaN	NaN	NaN	NaN	NaN
Q9NWS1	PCNA-interacting partner	PARPBP	1	1	1	1.2	65.054	NaN	NaN	NaN	NaN	NaN	NaN	NaN	NaN
Q9NVD7	Alpha-parvin	PARVA	1	2	2	7	42.243	NaN	NaN	NaN	NaN	NaN	NaN	NaN	NaN
Q9HBI1	Beta-parvin	PARVB	1	1	1	3.8	41.714	NaN	NaN	NaN	NaN	NaN	NaN	NaN	NaN
Q9BVG4	Protein PBDC1	PBDC1	1	2	2	11.6	26.056	NaN	NaN	8737800	9443400	NaN	NaN	1.08075	NaN
Q96AQ6	Pre-B-cell leukemia transcription factor-interacting protein 1	PBXIP1	1	1	1	2.1	80.642	NaN	NaN	NaN	NaN	NaN	NaN	NaN	NaN
P11498	Pyruvate carboxylase, mitochondrial	PC	1	3	3	4.1	129.63	1.1E+07	NaN	NaN	NaN	7924300	NaN	0.74998	NaN
P61457	Pterin-4-alpha-carbinolamine dehydratase	PCBD1	1	2	2	17.3	11.999	1.2E+08	1E+08	1.3E+08	NaN	1E+08	1.2E+08	0.94729	0.66846
Q15365	Poly(rC)-binding protein 1	PCBP1	1	4	3	13.8	37.497	7.2E+07	8.2E+07	7.8E+07	1E+08	5.4E+07	9.8E+07	1.1003	0.6499
Q15366;P57721	Poly(rC)-binding protein 2;Poly(rC)-binding protein 3	PCBP2	2	2	1	6	38.58	NaN	NaN	NaN	NaN	NaN	NaN	NaN	NaN
Q08174	Protocadherin-1	PCDH1	1	1	1	1.9	114.74	NaN	NaN	NaN	NaN	NaN	NaN	NaN	NaN
Q9H4Z3	Phosphorylated CTD-interacting factor 1	PCIF1	1	2	2	2.8	80.669	NaN	NaN	NaN	NaN	NaN	NaN	NaN	NaN
Q16822	Phosphoenolpyruvate carboxykinase [GTP], mitochondrial	PCK2	1	1	1	1.7	70.698	NaN	NaN	NaN	NaN	NaN	NaN	NaN	NaN
P22061	Protein-L-isoaspartate(D-aspartate) O-methyltransferase	PCMT1	1	2	2	8.8	24.636	5.5E+07	NaN	4.6E+07	5.5E+07	NaN	NaN	1.08028	NaN
P12004	Proliferating cell nuclear antigen	PCNA	1	1	1	3.1	28.768	NaN	NaN	NaN	NaN	NaN	NaN	NaN	NaN
Q9UHG3	Prenylcysteine oxidase 1	PCYOX1	1	3	3	7.1	56.639	NaN	NaN	NaN	1.8E+07	2.1E+07	2E+07	NaN	NaN
Q13442	28 kDa heat- and acid-stable phosphoprotein	PDAP1	1	3	3	16.6	20.63	3.4E+07	2.9E+07	4.3E+07	2.7E+07	3.1E+07	2.9E+07	0.82398	0.23226
Q9BUL8	Programmed cell death protein 10	PDCD10	1	2	2	9.4	24.701	NaN	NaN	NaN	NaN	NaN	NaN	NaN	NaN
O14737	Programmed cell death protein 5	PDCD5	1	2	2	16.8	14.285	5.7E+07	5.1E+07	5.8E+07	5.9E+07	4.4E+07	6.8E+07	1.02523	0.8584
O75340	Programmed cell death protein 6	PDCD6	1	1	1	5.8	21.868	NaN	NaN	NaN	NaN	NaN	NaN	NaN	NaN
Q8WUM4	Programmed cell death 6-interacting protein	PDCD6IP	1	13	13	16.9	96.022	1.1E+08	1.2E+08	1.3E+08	1.2E+08	1.1E+08	1.1E+08	0.92676	0.21744
P08559	Pyruvate dehydrogenase E1 component subunit alpha, somati	PDHA1	1	2	2	5.6	43.295	NaN	NaN	NaN	NaN	NaN	NaN	NaN	NaN
O00330	Pyruvate dehydrogenase protein X component, mitochondrial	PDHX	1	1	1	2.8	54.122	NaN	NaN	NaN	NaN	NaN	NaN	NaN	NaN
P30101	Protein disulfide-isomerase A3	PDIA3	1	6	6	14.1	56.782	2.5E+08	2.6E+08	2.6E+08	3.7E+08	2.9E+08	3.1E+08	1.26261	0.0422
P13667	Protein disulfide-isomerase A4	PDIA4	1	9	9	14.7	72.932	1.3E+08	1.4E+08	1.5E+08	1.4E+08	1.2E+08	1.5E+08	1.00652	0.92547
Q14554	Protein disulfide-isomerase A5	PDIA5	1	1	1	2.3	59.594	NaN	NaN	NaN	NaN	NaN	NaN	NaN	NaN
Q15084	Protein disulfide-isomerase A6	PDIA6	1	6	6	13	48.121	6.4E+08	6.4E+08	6.6E+08	7.2E+08	4.5E+08	5.6E+08	0.89199	0.42157

O00151	PDZ and LIM domain protein 1	PDLIM1	1	2	2	6.1	36.071	6.6E+07	5.4E+07	NaN	NaN	6E+07	7.1E+07	1.08551	0.58533
Q96HC4	PDZ and LIM domain protein 5	PDLIM5	1	2	2	4	63.944	NaN	NaN	1.6E+07	NaN	1.6E+07	NaN	0.98884	NaN
Q9NR12	PDZ and LIM domain protein 7	PDLIM7	1	3	3	7.2	49.844	2.6E+07	1.6E+07	2.5E+07	2.6E+07	3.3E+07	2.7E+07	1.27242	0.16624
Q9NUG6	p53 and DNA damage-regulated protein 1	PDRG1	1	1	1	12	15.511	NaN	NaN	NaN	NaN	NaN	NaN	NaN	NaN
Q29RF7	Sister chromatid cohesion protein PDS5 homolog A	PDS5A	1	6	4	5.5	150.83	1E+07	NaN	1.2E+07	1.9E+07	NaN	NaN	1.69813	NaN
Q9NTI5	Sister chromatid cohesion protein PDS5 homolog B	PDS5B	1	4	2	2.6	164.67	NaN	NaN	NaN	NaN	NaN	NaN	NaN	NaN
O00764	Pyridoxal kinase	PDXK	1	3	3	12.5	35.102	1.1E+08	9.4E+07	1.2E+08	1.1E+08	9E+07	9.5E+07	0.92288	0.3917
P30086	Phosphatidylethanolamine-binding protein 1;Hippocampal ch	PEBP1	1	2	2	15.5	21.057	NaN	NaN	NaN	NaN	NaN	NaN	NaN	NaN
P12955	Xaa-Pro dipeptidase	PEPD	1	3	3	5.7	54.548	2.2E+07	2.6E+07	2.5E+07	2.5E+07	2.6E+07	2.7E+07	1.06642	0.26567
O96011	Peroxisomal membrane protein 11B	PEX11B	1	1	1	4.2	28.431	NaN	NaN	NaN	NaN	NaN	NaN	NaN	NaN
O15067	Phosphoribosylformylglycinamidine synthase	PFAS	1	2	2	1.6	144.73	5.1E+07	NaN	5E+07	3.4E+07	3.9E+07	4.1E+07	0.75469	0.02428
Q9UHV9	Prefoldin subunit 2	PFDN2	1	2	2	15.6	16.648	NaN	NaN	NaN	NaN	NaN	NaN	NaN	NaN
Q9NQP4	Prefoldin subunit 4	PFDN4	1	1	1	9	15.314	NaN	NaN	NaN	NaN	NaN	NaN	NaN	NaN
O15212	Prefoldin subunit 6	PFDN6	1	1	1	10.9	14.582	NaN	NaN	NaN	NaN	NaN	NaN	NaN	NaN
O60825	6-phosphofructo-2-kinase/fructose-2,6-bisphosphatase 2;6-p	PFKFB2	1	2	2	4.8	58.476	NaN	1.3E+07	1.1E+07	9972500	9620900	NaN	0.82683	0.19991
Q16875	6-phosphofructo-2-kinase/fructose-2,6-bisphosphatase 3;6-p	PFKFB3	1	1	1	2.5	59.608	NaN	NaN	NaN	NaN	NaN	NaN	NaN	NaN
P17858	ATP-dependent 6-phosphofructokinase, liver type	PFKL	1	3	2	3.7	85.018	1.5E+07	1.1E+07	NaN	NaN	1.4E+07	NaN	1.10528	NaN
P08237	ATP-dependent 6-phosphofructokinase, muscle type	PFKM	1	4	3	5.4	85.182	NaN	NaN	9.7E+07	9.1E+07	1.8E+08	NaN	1.39987	NaN
Q01813	ATP-dependent 6-phosphofructokinase, platelet type	PFKP	1	8	7	9.8	85.595	2.3E+08	2.2E+08	1.8E+08	2.5E+08	2.4E+08	2.6E+08	1.19639	0.04551
P07737	Profilin-1	PFN1	1	4	3	26.4	15.054	6.4E+08	8E+08	7.9E+08	7.8E+08	8.6E+08	6.7E+08	1.03404	0.75261
P18669	Phosphoglycerate mutase 1	PGAM1	3	6	6	22.4	28.804	4.6E+08	4.3E+08	4.9E+08	3.7E+08	4.9E+08	5.1E+08	0.99146	0.93931
Q96HS1	Serine/threonine-protein phosphatase PGAM5, mitochondrial	PGAM5	1	3	3	12.5	32.004	NaN	4.6E+07	NaN	2.7E+07	NaN	NaN	0.58017	NaN
P52209	6-phosphogluconate dehydrogenase, decarboxylating	PGD	1	9	9	18.2	53.139	1.2E+09	1.3E+09	1.2E+09	1.2E+09	1.3E+09	1.3E+09	0.99695	0.9512
P00558	Phosphoglycerate kinase 1	PGK1	2	6	6	14.1	44.614	8.4E+08	7.3E+08	7.2E+08	6.7E+08	7.2E+08	7.3E+08	0.92873	0.26423
O95336	6-phosphogluconolactonase	PGLS	1	4	4	23.3	27.547	8.9E+07	NaN	7.6E+07	7.2E+07	4.7E+07	9.1E+07	0.85082	0.52476
P36871	Phosphoglucomutase-1	PGM1	1	11	11	20.8	61.448	6.4E+07	6.4E+07	6.5E+07	6.3E+07	6.3E+07	6.4E+07	0.98747	0.21906
Q96G03	Phosphoglucomutase-2	PGM2	1	3	3	5.7	68.283	1.2E+07	NaN	NaN	1.1E+07	1.9E+07	NaN	1.25292	NaN
Q6PCE3	Glucose 1,6-bisphosphate synthase	PGM2L1	1	6	6	11.4	70.441	3.9E+07	5.1E+07	4E+07	3.8E+07	4.5E+07	2.7E+07	0.8511	0.37308
A6NDG6	Phosphoglycolate phosphatase	PGP	1	2	2	7.2	34.006	NaN	NaN	NaN	NaN	NaN	NaN	NaN	NaN
O00264	Membrane-associated progesterone receptor component 1	PGRMC1	1	1	1	10.3	21.671	NaN	NaN	NaN	NaN	NaN	NaN	NaN	NaN
O15173	Membrane-associated progesterone receptor component 2	PGRMC2	1	2	2	9	23.818	NaN	1.1E+08	1.1E+08	5.6E+07	1.2E+08	1.1E+08	0.85682	0.56707
P35232	Prohibitin	PHB	1	4	4	14.7	29.804	NaN	2.8E+08	2.7E+08	2.2E+08	3E+08	2.9E+08	0.97515	0.84188
Q99623	Prohibitin-2	PHB2	1	3	3	10.4	33.296	NaN	2.4E+08	NaN	NaN	3.2E+08	2.4E+08	1.14908	NaN
O43175	D-3-phosphoglycerate dehydrogenase	PHGDH	1	4	4	10.1	56.65	3.1E+07	NaN	3.8E+07	NaN	4.6E+07	2.6E+07	1.0456	0.89847
Q9BTU6	Phosphatidylinositol 4-kinase type 2-alpha	PI4K2A	1	2	2	5.6	54.022	NaN	NaN	NaN	NaN	NaN	NaN	NaN	NaN
Q13492	Phosphatidylinositol-binding clathrin assembly protein	PICALM	1	1	1	1.4	70.754	NaN	NaN	NaN	NaN	NaN	NaN	NaN	NaN
Q8NEB9	Phosphatidylinositol 3-kinase catalytic subunit type 3	PIK3C3	1	1	1	1.4	101.55	NaN	NaN	NaN	NaN	NaN	NaN	NaN	NaN
P48426	Phosphatidylinositol 5-phosphate 4-kinase type-2 alpha	PIP4K2A	1	1	1	3.4	46.224	NaN	NaN	NaN	NaN	NaN	NaN	NaN	NaN
P78356	Phosphatidylinositol 5-phosphate 4-kinase type-2 beta	PIP4K2B	1	1	1	3.4	47.377	NaN	NaN	NaN	NaN	NaN	NaN	NaN	NaN
Q5JRX3	Presequence protease, mitochondrial	PITRM1	1	8	8	8.1	117.41	4.1E+07	3.3E+07	3E+07	3.2E+07	3.8E+07	3.1E+07	0.97266	0.81981
P30613	Pyruvate kinase PKLR	PKLR	1	2	1	3.1	61.829	NaN	NaN	NaN	NaN	NaN	NaN	NaN	NaN
P14618	Pyruvate kinase PKM	PKM	1	18	17	30.3	57.936	1.8E+09	2.1E+09	1.9E+09	2E+09	1.9E+09	1.7E+09	0.95379	0.56467
Q16512;Q16513	Serine/threonine-protein kinase N1;Serine/threonine-protein k	PKN1	2	2	2	2.2	103.93	7.1E+07	1.1E+08	NaN	6.5E+07	7.1E+07	6.6E+07	0.74922	0.20942
Q9Y263	Phospholipase A-2-activating protein	PLAA	1	3	3	4.2	87.156	NaN	NaN	NaN	2.4E+07	1.7E+07	NaN	NaN	NaN
Q8N3E9	1-phosphatidylinositol 4,5-bisphosphate phosphodiesterase d	PLCD3	1	1	1	1.4	89.257	NaN	NaN	NaN	NaN	NaN	NaN	NaN	NaN
P19174	1-phosphatidylinositol 4,5-bisphosphate phosphodiesterase g	PLCG1	1	1	1	1.1	148.53	NaN	NaN	NaN	NaN	NaN	NaN	NaN	NaN
Q8IV08	Phospholipase D3	PLD3	1	3	3	8.4	54.705	1.1E+07	1.4E+07	1.1E+07	NaN	NaN	NaN	NaN	NaN
Q15149	Plectin	PLEC	1	72	69	15.2	531.78	3E+08	2.8E+08	3E+08	3E+08	2.8E+08	3.1E+08	1.00457	0.90482
Q9Y2H5	Pleckstrin homology domain-containing family A member 6	PLEKHA6	1	1	1	1	117.13	NaN	NaN	NaN	NaN	NaN	NaN	NaN	NaN
Q96PX9	Pleckstrin homology domain-containing family G member 4B	PLEKHG4B	1	1	1	0.7	139.67	NaN	NaN	NaN	NaN	NaN	NaN	NaN	NaN
Q9HBL7	Plasminogen receptor (KT)	PLGRKT	1	1	1	8.8	17.201	NaN	NaN	NaN	NaN	NaN	NaN	NaN	NaN
Q99541	Perilipin-2	PLIN2	1	1	1	4.3	48.075	NaN	NaN	NaN	NaN	NaN	NaN	NaN	NaN
O60664	Perilipin-3	PLIN3	1	4	4	10.1	47.074	1.4E+08	1.3E+08	1.1E+08	1.4E+08	1.6E+08	8.6E+07	1.03131	0.87867
Q02809	Procollagen-lysine,2-oxoglutarate 5-dioxygenase 1	PLOD1	1	1	1	1.5	83.549	NaN	NaN	NaN	NaN	NaN	NaN	NaN	NaN
O00469	Procollagen-lysine,2-oxoglutarate 5-dioxygenase 2	PLOD2	1	10	10	15.7	84.685	1.1E+08	9.1E+07	1.2E+08	1E+08	9.7E+07	1.1E+08	0.95488	0.61251
O60568	Procollagen-lysine,2-oxoglutarate 5-dioxygenase 3	PLOD3	1	2	2	2.6	84.784	NaN	NaN	NaN	NaN	NaN	NaN	NaN	NaN
Q04941	Proteolipid protein 2	PLP2	1	1	1	8.6	16.691	NaN	NaN	NaN	NaN	NaN	NaN	NaN	NaN
Q14651	Plastin-1	PLS1	2	3	1	5.9	70.253	NaN	NaN	NaN	NaN	NaN	NaN	NaN	NaN
P13797	Plastin-3	PLS3	1	9	7	13.8	70.81	3.9E+08	4.4E+08	3.9E+08	3.4E+08	4.4E+08	3.6E+08	0.93134	0.46251

O15305	Phosphomannomutase 2	PMM2	2	4	4	14.6	28.082	NaN	5.2E+07	NaN	NaN	1.9E+07	NaN	0.37563	NaN
Q15126	Phosphomevalonate kinase	PMVK	1	1	1	4.7	21.995	NaN	NaN	NaN	NaN	NaN	NaN	NaN	NaN
Q8N490	Probable hydrolase PNKD	PNKD	1	1	1	3.9	42.875	NaN	NaN	NaN	NaN	NaN	NaN	NaN	NaN
Q96T60	Bifunctional polynucleotide phosphatase/kinase;Polynucleotide	PNKP	1	1	1	2.3	57.076	NaN	NaN	NaN	NaN	NaN	NaN	NaN	NaN
P00491	Purine nucleoside phosphorylase	PNP	1	6	6	23.2	32.118	5.5E+07	6.6E+07	4.7E+07	5.6E+07	5.7E+07	5.6E+07	0.9951	0.96273
Q96AD5	Patatin-like phospholipase domain-containing protein 2	PNPLA2	1	1	1	2.6	55.315	NaN	NaN	NaN	NaN	NaN	NaN	NaN	NaN
Q9NVS9	Pyridoxine-5-phosphate oxidase	PNPO	1	2	2	6.9	29.988	NaN	NaN	NaN	NaN	NaN	NaN	NaN	NaN
P28340	DNA polymerase delta catalytic subunit	POLD1	1	2	2	1.7	123.63	1.4E+07	NaN	NaN	9179000	NaN	NaN	0.6615	NaN
P49005	DNA polymerase delta subunit 2	POLD2	1	1	1	3.4	51.289	NaN	NaN	NaN	NaN	NaN	NaN	NaN	NaN
Q9Y2S7	Polymerase delta-interacting protein 2	POLDIP2	1	1	1	3.3	42.033	NaN	NaN	NaN	NaN	NaN	NaN	NaN	NaN
Q9NRF9	DNA polymerase epsilon subunit 3	POLE3	1	2	2	18.4	16.859	2.9E+07	3.1E+07	2.6E+07	2.9E+07	3.4E+07	2.9E+07	1.05902	0.48541
P30876	DNA-directed RNA polymerase II subunit RPB2	POLR2B	1	1	1	1.2	133.9	NaN	NaN	NaN	NaN	NaN	NaN	NaN	NaN
P19387	DNA-directed RNA polymerase II subunit RPB3	POLR2C	1	1	1	6.2	31.441	NaN	NaN	NaN	NaN	NaN	NaN	NaN	NaN
O00411	DNA-directed RNA polymerase, mitochondrial	POLRMT	1	1	1	1.1	138.62	NaN	NaN	NaN	NaN	NaN	NaN	NaN	NaN
Q15165	Serum paraoxonase/arylesterase 2	PON2	1	1	1	4	39.38	8E+07	9.4E+07	1.2E+08	5.7E+07	1.4E+08	1.6E+08	1.20991	0.58485
P16435	NADPH--cytochrome P450 reductase	POR	1	4	4	5.9	76.689	2.1E+07	NaN	2.8E+07	2.6E+07	2.3E+07	3.3E+07	1.13369	0.53318
Q15181	Inorganic pyrophosphatase	PPA1	1	2	2	5.9	32.66	3.2E+07	NaN	3.4E+07	2.7E+07	3E+07	2.9E+07	0.86471	0.03439
Q9H2U2	Inorganic pyrophosphatase 2, mitochondrial	PPA2	1	1	1	2.7	37.92	NaN	NaN	NaN	NaN	NaN	NaN	NaN	NaN
Q06203	Amidophosphoribosyltransferase	PPAT	1	3	3	6.2	57.398	2.7E+07	2.4E+07	NaN	2.6E+07	3.6E+07	3.2E+07	1.22497	0.22123
Q9HAB8	Phosphopantothenate--cysteine ligase	PPCS	1	2	2	9	34.005	NaN	NaN	NaN	4.2E+07	NaN	3.8E+07	NaN	NaN
Q86W92	Liprin-beta-1	PPFIBP1	1	2	2	2.8	114.02	NaN	NaN	NaN	NaN	NaN	NaN	NaN	NaN
P62937	Peptidyl-prolyl cis-trans isomerase A;Peptidyl-prolyl cis-trans i	PPIA	8	3	3	13.3	18.012	6.4E+08	7E+08	6.1E+08	6.2E+08	6.5E+08	6.3E+08	0.97345	0.56564
P23284	Peptidyl-prolyl cis-trans isomerase B	PPIB	1	2	2	7.4	23.742	1.2E+08	1.1E+08	1.2E+08	1.1E+08	1.2E+08	1.2E+08	0.9778	0.65605
Q08752	Peptidyl-prolyl cis-trans isomerase D	PPID	1	3	3	10.5	40.763	3.4E+07	NaN	NaN	3.9E+07	NaN	NaN	1.13707	NaN
P35813	Protein phosphatase 1A	PPM1A	1	1	1	4.7	42.447	NaN	NaN	NaN	NaN	NaN	NaN	NaN	NaN
O15355	Protein phosphatase 1G	PPM1G	1	2	2	4.4	59.271	1.8E+07	1.9E+07	NaN	2.2E+07	NaN	NaN	1.24045	NaN
Q9Y570	Protein phosphatase methylesterase 1	PPME1	1	3	3	8.8	42.315	1.9E+07	1.8E+07	2.8E+07	3.1E+07	2.5E+07	1.9E+07	1.16268	0.51334
P36873;P62140;P62136	Serine/threonine-protein phosphatase PP1-gamma catalytic su	PPP1CC	3	2	2	6.8	36.983	1.5E+08	NaN	NaN	2E+08	2.6E+08	NaN	1.49955	NaN
Q96QC0	Serine/threonine-protein phosphatase 1 regulatory subunit 10	PPP1R10	1	1	1	2	99.057	2.2E+07	NaN	NaN	1.8E+07	1.5E+07	NaN	0.76188	NaN
Q96A00	Protein phosphatase 1 regulatory subunit 14A	PPP1R14A	1	1	1	7.5	16.693	NaN	NaN	NaN	NaN	NaN	NaN	NaN	NaN
Q15435	Protein phosphatase 1 regulatory subunit 7	PPP1R7	1	3	3	8.9	41.564	2E+07	9193500	1.8E+07	1.4E+07	1.6E+07	1.7E+07	1.01739	0.94017
P67775	Serine/threonine-protein phosphatase 2A catalytic subunit alp	PPP2CA	1	1	1	4.2	35.594	NaN	NaN	NaN	NaN	NaN	NaN	NaN	NaN
P62714	Serine/threonine-protein phosphatase 2A catalytic subunit bet	PPP2CB	1	1	1	4.2	35.575	NaN	NaN	NaN	NaN	NaN	NaN	NaN	NaN
P30153	Serine/threonine-protein phosphatase 2A 65 kDa regulatory su	PPP2R1A	1	5	5	10.5	65.308	1.6E+08	1.7E+08	1.5E+08	1.9E+08	1.4E+08	1.9E+08	1.08634	0.46305
Q9Y2T4;P63151	Serine/threonine-protein phosphatase 2A 55 kDa regulatory su	PPP2R2C	2	1	1	2.5	51.514	NaN	NaN	NaN	NaN	NaN	NaN	NaN	NaN
Q15257	Serine/threonine-protein phosphatase 2A activator	PPP2R4	1	3	3	10.1	40.667	7.1E+07	5.6E+07	7.4E+07	7.5E+07	6.5E+07	7.4E+07	1.06521	0.52186
Q13362	Serine/threonine-protein phosphatase 2A 56 kDa regulatory su	PPP2R5C	1	1	1	1.9	61.06	NaN	NaN	NaN	NaN	NaN	NaN	NaN	NaN
Q14738	Serine/threonine-protein phosphatase 2A 56 kDa regulatory su	PPP2R5D	1	1	1	1.7	69.991	NaN	NaN	NaN	NaN	NaN	NaN	NaN	NaN
Q16537	Serine/threonine-protein phosphatase 2A 56 kDa regulatory su	PPP2R5E	1	1	1	2.8	54.699	NaN	NaN	NaN	NaN	NaN	NaN	NaN	NaN
Q08209;P16298	Serine/threonine-protein phosphatase 2B catalytic subunit alp	PPP3CA	2	4	4	7.5	58.687	NaN	NaN	2.5E+07	NaN	3E+07	NaN	1.19236	NaN
P53041	Serine/threonine-protein phosphatase 5	PPP5C	1	2	2	3.8	56.878	NaN	NaN	NaN	NaN	NaN	NaN	NaN	NaN
Q5H9R7	Serine/threonine-protein phosphatase 6 regulatory subunit 3	PPP6R3	1	3	3	3.9	97.668	NaN	NaN	NaN	NaN	NaN	NaN	NaN	NaN
O60831	PRA1 family protein 2	PRAF2	1	1	1	6.2	19.258	NaN	NaN	NaN	NaN	NaN	NaN	NaN	NaN
O43663	Protein regulator of cytokinesis 1	PRC1	1	1	1	2.1	71.606	NaN	NaN	NaN	NaN	NaN	NaN	NaN	NaN
Q06830	Peroxiredoxin-1	PRDX1	1	7	5	32.7	22.11	6.4E+08	4.8E+08	6.2E+08	6E+08	5.1E+08	6E+08	0.98689	0.90246
P30048	Thioredoxin-dependent peroxide reductase, mitochondrial	PRDX3	1	3	3	10.5	27.692	1.4E+08	1.2E+08	1.3E+08	1.2E+08	1.2E+08	1.1E+08	0.8826	0.10566
Q13162	Peroxiredoxin-4	PRDX4	1	3	1	10.3	30.54	NaN	NaN	NaN	NaN	NaN	NaN	NaN	NaN
P30044	Peroxiredoxin-5, mitochondrial	PRDX5	1	4	4	27.6	22.086	3.5E+07	3.8E+07	3.5E+07	4.5E+07	4.2E+07	4E+07	1.17112	0.02238
P30041	**Peroxiredoxin-6**	**PRDX6**	1	11	11	42.4	25.035	5.9E+08	5.3E+08	6.1E+08	7.6E+08	6.9E+08	6.7E+08	1.23205	0.01849
Q13131	5-AMP-activated protein kinase catalytic subunit alpha-1	PRKAA1	1	1	1	1.8	64.009	NaN	NaN	NaN	NaN	NaN	NaN	NaN	NaN
P17612	cAMP-dependent protein kinase catalytic subunit alpha	PRKACA	1	2	1	8	40.589	NaN	NaN	NaN	NaN	NaN	NaN	NaN	NaN
P22694	cAMP-dependent protein kinase catalytic subunit beta	PRKACB	2	3	2	7.4	40.622	NaN	NaN	NaN	NaN	NaN	NaN	NaN	NaN
P13861	cAMP-dependent protein kinase type II-alpha regulatory subu	PRKAR2A	1	1	1	5	45.518	NaN	NaN	NaN	NaN	NaN	NaN	NaN	NaN
P17252	Protein kinase C alpha type	PRKCA	1	2	2	4.2	76.749	NaN	NaN	NaN	NaN	NaN	NaN	NaN	NaN
P14314	Glucosidase 2 subunit beta	PRKCSH	1	4	4	7.6	59.425	3.1E+07	2.5E+07	2.9E+07	2.2E+07	2.7E+07	3.2E+07	0.94818	0.69239
P78527	DNA-dependent protein kinase catalytic subunit	PRKDC	1	24	24	5.9	469.08	1.2E+08	1.5E+08	1.2E+08	1.2E+08	1E+08	1.2E+08	0.89286	0.26053
Q99873	Protein arginine N-methyltransferase 1	PRMT1	2	5	5	14.6	42.461	2.1E+08	2.2E+08	1.8E+08	1.9E+08	2.3E+08	2.6E+08	1.11084	0.36834
O60678	Protein arginine N-methyltransferase 3	PRMT3	1	1	1	1.7	59.902	NaN	NaN	NaN	NaN	NaN	NaN	NaN	NaN
O14744	Protein arginine N-methyltransferase 5;Protein arginine N-met	PRMT5	1	4	4	8.2	72.683	8.2E+07	9.5E+07	8.8E+07	7.8E+07	8.8E+07	9.1E+07	0.97221	0.66967

Q9UMS4	Pre-mRNA-processing factor 19	PRPF19	1	2	2	5	55.18	5E+07	5.1E+07	NaN	NaN	4.8E+07	NaN	0.95179	NaN
O75400	Pre-mRNA-processing factor 40 homolog A	PRPF40A	1	1	1	1.4	108.8	NaN	NaN	NaN	NaN	NaN	NaN	NaN	NaN
Q6P2Q9	Pre-mRNA-processing-splicing factor 8	PRPF8	1	5	5	2.2	273.6	7496400	1.7E+07	1.7E+07	3E+07	1.4E+07	1.7E+07	1.47993	0.32684
P60891	Ribose-phosphate pyrophosphokinase 1	PRPS1	1	2	2	6.3	34.834	8.6E+07	NaN	8E+07	NaN	8.2E+07	8.7E+07	1.01991	0.73409
P11908;P21108	Ribose-phosphate pyrophosphokinase 2;Ribose-phosphate py	PRPS2	2	2	2	8.8	34.769	9E+07	9.1E+07	7.6E+07	NaN	NaN	NaN	NaN	NaN
Q14558;O60256	Phosphoribosyl pyrophosphate synthase-associated protein 1;	PRPSAP1	2	1	1	3.9	39.393	NaN	NaN	NaN	NaN	NaN	NaN	NaN	NaN
P48634	Protein PRRC2A	PRRC2A	1	4	4	2.8	228.86	NaN	NaN	NaN	NaN	NaN	NaN	NaN	NaN
Q6PEW0	Inactive serine protease 54	PRSS54	1	1	1	3.3	43.832	NaN	NaN	NaN	NaN	NaN	NaN	NaN	NaN
P07602	Prosaposin;Saposin-A;Saposin-B-Val;Saposin-B;Saposin-C;Sap	PSAP	1	5	5	10.1	58.112	1.9E+08	2.7E+08	2.8E+08	1.6E+08	2.2E+08	2.3E+08	0.83593	0.33325
Q9Y617	Phosphoserine aminotransferase	PSAT1	1	2	2	5.7	40.422	8.9E+07	9.7E+07	2.7E+07	5.6E+07	8.6E+07	6.5E+07	0.97186	0.93727
O75475	PC4 and SFRS1-interacting protein	PSIP1	1	1	1	2.8	60.103	NaN	NaN	NaN	NaN	NaN	NaN	NaN	NaN
P25786	Proteasome subunit alpha type-1	PSMA1	1	3	3	15.2	29.555	1.3E+08	1E+08	1.2E+08	1E+08	1.2E+08	1.3E+08	0.9774	0.82997
P25788	Proteasome subunit alpha type-3	PSMA3	1	1	1	2.7	28.433	NaN	NaN	NaN	NaN	NaN	NaN	NaN	NaN
P25789	Proteasome subunit alpha type-4	PSMA4	1	1	1	3.8	29.483	NaN	NaN	NaN	NaN	NaN	NaN	NaN	NaN
P60900	Proteasome subunit alpha type-6	PSMA6	1	1	1	4.9	27.399	NaN	NaN	NaN	NaN	NaN	NaN	NaN	NaN
O14818	Proteasome subunit alpha type-7	PSMA7	2	4	4	21	27.887	2E+08	2.4E+08	2E+08	1.9E+08	2.4E+08	1.9E+08	0.96102	0.7259
P49721	Proteasome subunit beta type-2	PSMB2	1	2	2	10.4	22.836	2.8E+07	NaN	2.9E+07	2.4E+07	3.1E+07	2.3E+07	0.90442	0.49012
P62191	26S protease regulatory subunit 4	PSMC1	1	5	5	7.7	49.184	2.8E+07	5.5E+07	3.9E+07	NaN	4E+07	NaN	0.96651	NaN
P35998	26S protease regulatory subunit 7	PSMC2	1	6	6	13.2	48.633	1.9E+08	2E+08	1.7E+08	1.7E+08	1.9E+08	1.9E+08	0.98155	0.7668
P17980	26S protease regulatory subunit 6A	PSMC3	1	4	4	13.9	49.203	2.2E+07	1.8E+07	2.7E+07	1.9E+07	2.5E+07	2.7E+07	1.04601	0.7926
P43686	26S protease regulatory subunit 6B	PSMC4	1	4	4	8.6	47.366	4.1E+07	5.3E+07	4.3E+07	5.4E+07	5.4E+07	4.3E+07	1.10739	0.39214
P62195	26S protease regulatory subunit 8	PSMC5	1	6	6	15.8	45.626	5.6E+07	6.1E+07	4.2E+07	5E+07	4.9E+07	5.3E+07	0.95209	0.67594
P62333	26S protease regulatory subunit 10B	PSMC6	1	5	5	14.4	44.172	7.5E+07	8.4E+07	6.1E+07	5.8E+07	8.1E+07	7.9E+07	0.99403	0.96685
Q99460	26S proteasome non-ATPase regulatory subunit 1	PSMD1	1	8	8	10.6	105.84	3.5E+07	4.2E+07	3.8E+07	4.2E+07	4E+07	3.4E+07	1.01419	0.87332
O75832	26S proteasome non-ATPase regulatory subunit 10	PSMD10	1	1	1	5.3	24.428	NaN	NaN	NaN	NaN	NaN	NaN	NaN	NaN
O00231	26S proteasome non-ATPase regulatory subunit 11	PSMD11	1	1	1	2.8	47.463	NaN	NaN	NaN	NaN	NaN	NaN	NaN	NaN
O00232	26S proteasome non-ATPase regulatory subunit 12	PSMD12	1	5	5	10.7	52.904	3E+07	2.8E+07	3.5E+07	3.2E+07	3.4E+07	3.4E+07	1.06848	0.35594
Q9UNM6	26S proteasome non-ATPase regulatory subunit 13	PSMD13	1	4	4	9.6	42.945	NaN	1.3E+08	1.3E+08	1.1E+08	1.1E+08	1.2E+08	0.85068	0.0155
O00487	26S proteasome non-ATPase regulatory subunit 14	PSMD14	1	1	1	5.2	34.577	NaN	2.5E+07	2.5E+07	NaN	2.2E+07	1.8E+07	0.79601	0.14928
Q13200	26S proteasome non-ATPase regulatory subunit 2	PSMD2	1	5	5	5.5	100.2	4.6E+07	6.2E+07	3.1E+07	4.6E+07	3.8E+07	1.1E+07	0.6785	0.33823
O43242	26S proteasome non-ATPase regulatory subunit 3	PSMD3	1	7	7	15.7	60.977	1.1E+08	9.9E+07	1.2E+08	9.3E+07	9.9E+07	1.2E+08	0.94861	0.6057
P55036;A2A3N6	26S proteasome non-ATPase regulatory subunit 4;Putative PIP	PSMD4	2	1	1	4.5	40.736	NaN	NaN	NaN	NaN	NaN	NaN	NaN	NaN
Q16401	26S proteasome non-ATPase regulatory subunit 5	PSMD5	1	2	2	3.8	56.195	5E+07	4.4E+07	4.7E+07	5.3E+07	6.2E+07	5.4E+07	1.20259	0.05219
Q15008	26S proteasome non-ATPase regulatory subunit 6	PSMD6	1	6	6	11.3	45.531	7.8E+07	7E+07	8.5E+07	7.4E+07	6.9E+07	6.8E+07	0.89995	0.16655
O00233	26S proteasome non-ATPase regulatory subunit 9	PSMD9	1	2	2	12.1	24.682	NaN	NaN	NaN	NaN	NaN	NaN	NaN	NaN
Q06323	Proteasome activator complex subunit 1	PSME1	1	6	6	18.1	28.723	7.7E+07	8.8E+07	6.9E+07	8.4E+07	7.9E+07	6.6E+07	0.97644	0.82173
Q9UL46	Proteasome activator complex subunit 2	PSME2	1	3	3	17.6	27.401	9.9E+07	1.1E+08	8.1E+07	8.5E+07	7.6E+07	8.6E+07	0.84792	0.18747
P61289	Proteasome activator complex subunit 3	PSME3	1	4	4	20.9	29.506	1.2E+08	1.1E+08	1.2E+08	1.2E+08	1.1E+08	1E+08	0.92592	0.19338
Q14997	Proteasome activator complex subunit 4	PSME4	1	4	4	3.3	211.33	NaN	1.1E+07	1.2E+07	NaN	1.1E+07	1.2E+07	1.0066	0.936
Q92530	Proteasome inhibitor PI31 subunit	PSMF1	1	1	1	5.5	29.816	NaN	NaN	NaN	NaN	NaN	NaN	NaN	NaN
Q969U7	Proteasome assembly chaperone 2	PSMG2	1	2	2	8.3	29.396	1.7E+07	NaN	1.4E+07	NaN	1.7E+07	1.6E+07	1.07099	0.54671
Q8WXF1	Paraspeckle component 1	PSPC1	1	3	2	7.1	58.743	1.7E+07	1.4E+07	1.5E+07	1.8E+07	1.3E+07	1.2E+07	0.9153	0.52904
P78330	Phosphoserine phosphatase	PSPH	1	2	2	10.2	25.007	8E+07	8.9E+07	5.1E+07	7.8E+07	7.7E+07	8.4E+07	1.0828	0.62894
P26599	Polypyrimidine tract-binding protein 1	PTBP1	1	2	2	3.1	59.632	1.6E+08	1.5E+08	2.4E+08	2.6E+08	2.3E+08	2.5E+08	1.32624	0.12325
O95758	Polypyrimidine tract-binding protein 3	PTBP3	1	1	1	1.6	59.689	NaN	NaN	NaN	NaN	NaN	NaN	NaN	NaN
Q96EY7	Pentatricopeptide repeat domain-containing protein 3, mitoch	PTCD3	1	2	2	3	78.549	NaN	NaN	NaN	NaN	NaN	NaN	NaN	NaN
Q9BVG9	Phosphatidylserine synthase 2	PTDSS2	1	2	2	7.6	56.252	1.5E+07	1.4E+07	6897000	NaN	NaN	NaN	NaN	NaN
O14684	Prostaglandin E synthase	PTGES	1	1	1	6.6	17.102	NaN	NaN	NaN	NaN	NaN	NaN	NaN	NaN
Q15185	Prostaglandin E synthase 3	PTGES3	1	2	2	6.2	18.697	4.8E+08	5.1E+08	3.8E+08	5.2E+08	4.1E+08	4.6E+08	1.01512	0.89322
Q14914	Prostaglandin reductase 1	PTGR1	1	5	5	13.1	35.869	2.7E+08	3.6E+08	2.7E+08	3.6E+08	3.1E+08	3.3E+08	1.10707	0.39861
P06454	Prothymosin alpha;Prothymosin alpha, N-terminally processed	PTMA	1	3	3	22.5	12.203	1.5E+07	1.1E+07	1.6E+07	1.8E+07	1.4E+07	1.8E+07	1.198	0.23656
Q12974;Q93096	Protein tyrosine phosphatase type IVA 2;Protein tyrosine phosp	PTP4A2	2	1	1	5.4	19.127	NaN	NaN	NaN	NaN	NaN	NaN	NaN	NaN
Q06124	Tyrosine-protein phosphatase non-receptor type 11	PTPN11	1	3	3	5.4	68.01	2.5E+07	2.4E+07	2.3E+07	2.8E+07	2.6E+07	2.5E+07	1.10121	0.10919
P10586	Receptor-type tyrosine-protein phosphatase F	PTPRF	1	3	3	2.5	212.88	NaN	2.1E+07	2.8E+07	1.6E+07	1.8E+07	NaN	0.68811	0.17715
Q12913	Receptor-type tyrosine-protein phosphatase eta	PTPRJ	1	2	2	1.9	145.94	NaN	NaN	NaN	NaN	NaN	NaN	NaN	NaN
Q6GMV3	Putative peptidyl-tRNA hydrolase PTRHD1	PTRHD1	1	2	2	15	15.805	2.2E+07	NaN	NaN	2.2E+07	2.1E+07	NaN	1.00126	NaN
P11216	Glycogen phosphorylase, brain form	PYGB	2	14	12	16.6	96.695	2.3E+08	2.2E+08	2.2E+08	2.3E+08	2.3E+08	2.2E+08	0.98891	0.58449
P06737	Glycogen phosphorylase, liver form	PYGL	1	5	3	6.3	97.147	2.3E+07	2.5E+07	NaN	1.8E+07	2.8E+07	NaN	0.96294	0.86823
P47897	Glutamine--tRNA ligase	QARS	1	5	5	7.7	87.798	8.3E+07	8.3E+07	7.7E+07	7.3E+07	7.8E+07	8.5E+07	0.97544	0.6443

Q9NXS2	Glutaminyl-peptide cyclotransferase-like protein	QPCTL	1	2	2	5.8	42.924	NaN	NaN	NaN	NaN	NaN	NaN	NaN	NaN
Q15907	Ras-related protein Rab-11B	RAB11B	1	4	1	17	24.488	1.4E+08	1.5E+08	1.4E+08	1.4E+08	1.5E+08	1.3E+08	0.9831	0.74598
P61106	Ras-related protein Rab-14	RAB14	1	2	2	10.7	23.897	NaN	NaN	NaN	NaN	NaN	NaN	NaN	NaN
Q9NP72	Ras-related protein Rab-18	RAB18	1	1	1	7.3	22.977	NaN	NaN	NaN	NaN	NaN	NaN	NaN	NaN
P62820	Ras-related protein Rab-1A	RAB1A	4	6	4	25.4	22.677	1.2E+08	8.8E+07	1.1E+08	1.6E+08	1.2E+08	1.4E+08	1.29168	0.11922
Q9H0U4	Ras-related protein Rab-1B	RAB1B	1	4	2	21.9	22.171	5.1E+07	NaN	4.8E+07	4.8E+07	NaN	4.7E+07	0.9645	0.44048
Q9UL25	Ras-related protein Rab-21	RAB21	1	1	1	6.7	24.347	NaN	NaN	NaN	NaN	NaN	NaN	NaN	NaN
O00194	Ras-related protein Rab-27B	RAB27B	1	2	2	11	24.608	NaN	NaN	1.7E+07	1.5E+07	NaN	NaN	0.92577	NaN
Q15286	Ras-related protein Rab-35	RAB35	1	2	2	9	23.025	1.7E+07	2.4E+07	3E+07	NaN	3.1E+07	NaN	1.29673	NaN
P20337	Ras-related protein Rab-3B	RAB3B	1	3	3	16.4	24.758	1.7E+08	NaN	NaN	1.5E+08	1.2E+08	1.3E+08	0.80648	NaN
O95716;P20336	Ras-related protein Rab-3D;Ras-related protein Rab-3A	RAB3D	2	1	1	3.7	24.267	NaN	NaN	NaN	NaN	NaN	NaN	NaN	NaN
Q15042	Rab3 GTPase-activating protein catalytic subunit	RAB3GAP1	1	3	3	3.3	110.52	1.3E+07	1.4E+07	1.8E+07	1.5E+07	1.5E+07	1.8E+07	1.0585	0.6335
Q9H2M9	Rab3 GTPase-activating protein non-catalytic subunit	RAB3GAP2	1	3	3	2.5	155.98	NaN	NaN	NaN	NaN	NaN	NaN	NaN	NaN
P20339	Ras-related protein Rab-5A	RAB5A	1	1	1	5.6	23.658	NaN	NaN	NaN	NaN	NaN	NaN	NaN	NaN
P61020	Ras-related protein Rab-5B	RAB5B	1	1	1	5.6	23.707	NaN	NaN	NaN	NaN	NaN	NaN	NaN	NaN
P51148	Ras-related protein Rab-5C	RAB5C	1	2	2	11.1	23.482	NaN	NaN	NaN	NaN	NaN	NaN	NaN	NaN
P20340	Ras-related protein Rab-6A	RAB6A	1	3	3	12.5	23.593	NaN	3.7E+07	2.9E+07	NaN	NaN	NaN	NaN	NaN
P61006	Ras-related protein Rab-8A	RAB8A	1	3	2	12.6	23.668	NaN	NaN	NaN	NaN	NaN	NaN	NaN	NaN
Q92930	Ras-related protein Rab-8B	RAB8B	1	3	2	17.9	23.584	NaN	NaN	NaN	NaN	NaN	NaN	NaN	NaN
P63000;P60763	Ras-related C3 botulinum toxin substrate 1;Ras-related C3 bot	RAC1	3	3	3	16.1	21.45	2.2E+08	2.7E+08	2.7E+08	1.6E+08	2.7E+08	1.3E+08	0.74711	0.24732
O60216	Double-strand-break repair protein rad21 homolog	RAD21	1	2	2	3.8	71.689	NaN	NaN	NaN	NaN	NaN	NaN	NaN	NaN
P54725	UV excision repair protein RAD23 homolog A	RAD23A	1	3	3	13.2	39.609	3.9E+07	3.8E+07	4.2E+07	4.6E+07	3.9E+07	NaN	1.06191	0.48027
Q92878	DNA repair protein RAD50	RAD50	1	2	2	1.5	153.89	NaN	NaN	NaN	NaN	NaN	NaN	NaN	NaN
P78406	mRNA export factor	RAE1	1	2	2	4.9	40.968	NaN	NaN	7.1E+07	1.1E+08	8.8E+07	7.4E+07	1.28847	NaN
Q86X10	Ral GTPase-activating protein subunit beta	RALGAPB	1	1	1	0.5	166.8	NaN	NaN	NaN	NaN	NaN	NaN	NaN	NaN
P62826	GTP-binding nuclear protein Ran	RAN	1	5	5	17.6	24.423	3.5E+08	3.1E+08	3.2E+08	3.8E+08	3E+08	4.2E+08	1.13376	0.31435
P43487	Ran-specific GTPase-activating protein	RANBP1	1	2	2	22.4	23.31	1.2E+08	1.1E+08	1E+08	1.1E+08	1.1E+08	8.9E+07	0.91409	0.29826
P46060	Ran GTPase-activating protein 1	RANGAP1	1	2	2	4.3	63.541	NaN	NaN	NaN	NaN	NaN	NaN	NaN	NaN
P62834;P61224;A6NIZ1	Ras-related protein Rap-1A;Ras-related protein Rap-1b;Ras-re	RAP1A	3	1	1	5.4	20.987	NaN	NaN	NaN	NaN	NaN	NaN	NaN	NaN
P52306	Rap1 GTPase-GDP dissociation stimulator 1	RAP1GDS1	1	2	2	2.6	66.316	NaN	NaN	NaN	NaN	NaN	NaN	NaN	NaN
Q9Y3L5;P61225;P10114	Ras-related protein Rap-2c;Ras-related protein Rap-2b;Ras-re	RAP2C	3	2	2	10.4	20.745	NaN	NaN	NaN	NaN	NaN	NaN	NaN	NaN
P54136	Arginine--tRNA ligase, cytoplasmic	RARS	1	6	6	9.5	75.378	8.9E+07	8.9E+07	1E+08	1.1E+08	9E+07	9.3E+07	1.02868	0.72133
P20936	Ras GTPase-activating protein 1	RASA1	1	2	2	3.7	116.4	NaN	NaN	NaN	NaN	NaN	NaN	NaN	NaN
Q16576	Histone-binding protein RBBP7	RBBP7	1	1	1	3.8	47.82	NaN	NaN	NaN	NaN	NaN	NaN	NaN	NaN
O75884	Putative hydrolase RBBP9	RBBP9	1	1	1	6.5	21	NaN	NaN	NaN	NaN	NaN	NaN	NaN	NaN
P49756	RNA-binding protein 25	RBM25	1	1	1	2.1	100.18	NaN	NaN	NaN	NaN	NaN	NaN	NaN	NaN
Q9P2N5	RNA-binding protein 27	RBM27	1	2	2	1.1	118.72	NaN	NaN	NaN	NaN	NaN	NaN	NaN	NaN
Q9BWF3;Q9BQ04	RNA-binding protein 4;RNA-binding protein 4B	RBM4	2	3	3	11.8	40.313	NaN	NaN	NaN	NaN	NaN	NaN	NaN	NaN
A0AV96	RNA-binding protein 47	RBM47	1	1	1	1.9	64.098	NaN	NaN	NaN	NaN	NaN	NaN	NaN	NaN
Q9Y5S9	RNA-binding protein 8A	RBM8A	1	1	1	10.9	19.889	NaN	NaN	NaN	NaN	NaN	NaN	NaN	NaN
P29558	RNA-binding motif, single-stranded-interacting protein 1	RBMS1	1	1	1	3.7	44.505	NaN	NaN	NaN	NaN	NaN	NaN	NaN	NaN
P09455	Retinol-binding protein 1	RBP1	1	1	1	8.9	15.85	NaN	NaN	NaN	NaN	NaN	NaN	NaN	NaN
Q9P258	Protein RCC2	RCC2	1	2	2	4.8	56.084	NaN	NaN	NaN	NaN	NaN	NaN	NaN	NaN
Q15293	Reticulocalbin-1	RCN1	1	2	2	3.9	38.89	3.2E+07	3.6E+07	3.9E+07	4.4E+07	3.8E+07	4.4E+07	1.17254	0.09644
Q14257	Reticulocalbin-2	RCN2	1	2	2	6.3	36.876	NaN	NaN	NaN	NaN	NaN	NaN	NaN	NaN
Q9HBH5	Retinol dehydrogenase 14	RDH14	1	1	1	5.1	36.864	NaN	NaN	NaN	NaN	NaN	NaN	NaN	NaN
P35241	Radixin	RDX	1	12	3	13.6	68.563	NaN	NaN	NaN	NaN	NaN	NaN	NaN	NaN
P46063	ATP-dependent DNA helicase Q1	RECQL	1	6	6	9.9	73.457	7.1E+07	4.5E+07	4.5E+07	9.2E+07	5.9E+07	6.3E+07	1.32856	0.25865
Q04206	Transcription factor p65	RELA	1	2	2	4.2	60.218	1.9E+07	NaN	1.6E+07	1.7E+07	2.5E+07	2.1E+07	1.17489	0.41233
O15258	Protein RER1	RER1	1	1	1	9.7	22.958	NaN	NaN	NaN	NaN	NaN	NaN	NaN	NaN
Q9Y3B8	Oligoribonuclease, mitochondrial	REXO2	1	2	2	8.9	26.832	7.5E+07	6.4E+07	NaN	6.4E+07	NaN	6.7E+07	0.93686	0.50139
P35251	Replication factor C subunit 1	RFC1	1	1	1	1.4	128.25	NaN	NaN	NaN	NaN	NaN	NaN	NaN	NaN
P61586;P08134	Transforming protein RhoA;Rho-related GTP-binding protein R	RHOA	2	4	4	19.2	21.768	1.5E+08	1.7E+08	1.4E+08	2.1E+08	1.8E+08	1.6E+08	1.18842	0.15156
P84095	Rho-related GTP-binding protein RhoG	RHOG	1	2	2	13.6	21.308	NaN	NaN	NaN	NaN	NaN	NaN	NaN	NaN
Q9NPQ8	Synembryn-A	RIC8A	1	1	1	1.9	59.709	NaN	NaN	NaN	NaN	NaN	NaN	NaN	NaN
Q96TC7	Regulator of microtubule dynamics protein 3	RMDN3	1	1	1	2.3	52.118	NaN	NaN	NaN	NaN	NaN	NaN	NaN	NaN
Q9H920	RING finger protein 121	RNF121	1	1	1	5.2	37.882	NaN	NaN	NaN	NaN	NaN	NaN	NaN	NaN
Q9BV68	E3 ubiquitin-protein ligase RNF126	RNF126	1	1	1	4.5	33.861	NaN	NaN	NaN	NaN	NaN	NaN	NaN	NaN
Q9UBS8	E3 ubiquitin-protein ligase RNF14	RNF14	1	1	1	2.5	53.837	NaN	NaN	NaN	NaN	NaN	NaN	NaN	NaN

Q96K19	E3 ubiquitin-protein ligase RNF170	RNF170	1	1	1	5.4	29.814	NaN	NaN	NaN	NaN	NaN	NaN	NaN	NaN
Q9P0P0	E3 ubiquitin-protein ligase RNF181	RNF181	1	2	2	22.2	17.909	NaN	NaN	NaN	NaN	NaN	NaN	NaN	NaN
Q5VTR2;O75150	E3 ubiquitin-protein ligase BRE1A;E3 ubiquitin-protein ligase E	RNF20	2	1	1	1.4	113.66	NaN	NaN	NaN	NaN	NaN	NaN	NaN	NaN
Q63HN8	E3 ubiquitin-protein ligase RNF213	RNF213	1	7	7	1.8	591.4	NaN	1.8E+07	1.9E+07	1.7E+07	2.1E+07	2.2E+07	1.05959	0.61729
O60942	mRNA-capping enzyme;Polynucleotide 5-triphosphatase;mRN	RNGTT	1	1	1	2.3	68.556	NaN	NaN	NaN	NaN	NaN	NaN	NaN	NaN
P13489	Ribonuclease inhibitor	RNH1	1	2	2	5.2	49.973	NaN	NaN	NaN	NaN	NaN	NaN	NaN	NaN
Q9HC36	rRNA methyltransferase 3, mitochondrial	RNMTL1	1	2	2	5.2	47.019	NaN	NaN	NaN	NaN	NaN	NaN	NaN	NaN
Q9H4A4	Aminopeptidase B	RNPEP	1	4	4	7.8	72.595	7.7E+07	7.8E+07	6.7E+07	7.1E+07	8E+07	6E+07	0.95707	0.67037
O75116	Rho-associated protein kinase 2	ROCK2	1	5	5	4.4	160.9	3.3E+07	2.8E+07	3.9E+07	2.1E+07	2.8E+07	2.6E+07	0.75227	0.10464
P27694	Replication protein A 70 kDa DNA-binding subunit;Replication	RPA1	1	1	1	1.9	68.137	NaN	NaN	NaN	NaN	NaN	NaN	NaN	NaN
Q9H6T3	RNA polymerase II-associated protein 3	RPAP3	1	3	3	5.7	75.718	1.9E+07	2.4E+07	NaN	NaN	NaN	1.7E+07	0.76482	NaN
Q96KN7	X-linked retinitis pigmentosa GTPase regulator-interacting pro	RPGRIP1	1	1	1	0.7	146.68	NaN	NaN	NaN	NaN	NaN	NaN	NaN	NaN
P62906	60S ribosomal protein L10a	RPL10A	1	3	3	13.4	24.831	2.6E+08	3.6E+08	2.7E+08	2.7E+08	4.2E+08	2.4E+08	1.03745	0.86786
P62913	60S ribosomal protein L11	RPL11	1	4	4	24.2	20.252	3E+08	3.6E+08	2.9E+08	3.5E+08	2.6E+08	3.3E+08	0.98283	0.8825
P30050	60S ribosomal protein L12	RPL12	1	2	2	20.6	17.818	2.7E+08	2.5E+08	2.4E+08	1.7E+08	2.6E+08	2.2E+08	0.83284	0.19712
P26373	60S ribosomal protein L13	RPL13	1	3	3	13.7	24.261	1.6E+08	1.1E+08	1.4E+08	1.9E+08	1.2E+08	1.3E+08	1.0653	0.75677
P40429	60S ribosomal protein L13a	RPL13A	2	4	4	17.7	23.577	3.1E+08	2.7E+08	2.9E+08	2.7E+08	3.1E+08	3.1E+08	1.03117	0.6468
P50914	60S ribosomal protein L14	RPL14	1	3	3	14.4	23.432	6E+08	4.3E+08	5.4E+08	7.1E+08	5.8E+08	5.7E+08	1.18322	0.2283
P61313	60S ribosomal protein L15	RPL15	1	5	5	23.5	24.146	1.6E+08	1.1E+08	1.8E+08	2E+08	1.7E+08	1.8E+08	1.25647	0.14852
P18621	60S ribosomal protein L17	RPL17	1	1	1	7.6	21.397	1.9E+08	1.4E+08	2E+08	1.9E+08	1.6E+08	2.1E+08	1.05576	0.70235
Q07020	60S ribosomal protein L18	RPL18	1	4	4	21.8	21.634	1.5E+08	1.6E+08	1.4E+08	1.6E+08	3.1E+08	1E+08	1.27087	0.5527
Q02543	60S ribosomal protein L18a	RPL18A	1	1	1	4.5	20.762	NaN	NaN	NaN	NaN	NaN	NaN	NaN	NaN
P84098	60S ribosomal protein L19	RPL19	1	2	2	12.8	23.466	NaN	2.5E+08	2.4E+08	3.1E+08	3.1E+08	2.4E+08	1.15659	0.25245
P35268	60S ribosomal protein L22	RPL22	1	1	1	10.2	14.787	NaN	NaN	NaN	NaN	NaN	NaN	NaN	NaN
P62750	60S ribosomal protein L23a	RPL23A	1	4	4	22.4	17.695	7.6E+07	1.2E+08	4E+07	8.8E+07	8.9E+07	7.4E+07	1.05341	0.86759
P83731	60S ribosomal protein L24	RPL24	1	1	1	5.1	17.779	NaN	NaN	NaN	NaN	NaN	NaN	NaN	NaN
P61254	60S ribosomal protein L26	RPL26	1	4	2	23.4	17.258	1.7E+08	1.8E+08	1.8E+08	2.4E+08	2.4E+08	1.9E+08	1.24636	0.07708
Q9UNX3	60S ribosomal protein L26-like 1	RPL26L1	1	4	2	23.4	17.256	NaN	NaN	NaN	NaN	NaN	NaN	NaN	NaN
P61353	60S ribosomal protein L27	RPL27	1	2	2	13.2	15.798	5E+08	4.5E+08	NaN	5.1E+08	4.8E+08	5.5E+08	1.09156	0.29013
P46776	60S ribosomal protein L27a	RPL27A	1	2	2	13.5	16.561	4.7E+08	4.3E+08	4.6E+08	6.1E+08	6E+08	5.9E+08	1.33381	0.00026
P46779	60S ribosomal protein L28	RPL28	1	3	3	16.8	15.747	NaN	1.3E+08	2.1E+08	2.1E+08	1.5E+08	1.6E+08	1.00128	0.99577
P47914	60S ribosomal protein L29	RPL29	1	2	2	14.5	17.752	8.6E+07	1.7E+07	9.6E+07	1.1E+08	7.8E+07	8.8E+07	1.38362	0.3926
P39023	60S ribosomal protein L3	RPL3	2	5	5	10.9	46.108	3.6E+07	7710600	4.1E+07	5.1E+07	3.1E+07	5E+07	1.54591	0.27596
P62888	60S ribosomal protein L30	RPL30	1	1	1	7	12.784	NaN	NaN	NaN	NaN	NaN	NaN	NaN	NaN
P62899	60S ribosomal protein L31	RPL31	1	2	2	13.6	14.463	NaN	NaN	NaN	NaN	NaN	NaN	NaN	NaN
P62910	60S ribosomal protein L32	RPL32	1	1	1	10.4	15.86	NaN	NaN	NaN	NaN	NaN	NaN	NaN	NaN
P49207	60S ribosomal protein L34	RPL34	1	1	1	7.7	13.293	NaN	NaN	NaN	NaN	NaN	NaN	NaN	NaN
P18077	60S ribosomal protein L35a	RPL35A	1	3	3	20.9	12.538	6.2E+07	4.5E+08	1.2E+08	7.4E+07	3.3E+08	NaN	0.9628	0.96839
Q969Q0;P83881	60S ribosomal protein L36a-like;60S ribosomal protein L36a	RPL36AL	2	3	3	17	12.469	NaN	1.8E+08	2E+08	2.2E+08	NaN	NaN	1.15127	NaN
P36578	60S ribosomal protein L4	RPL4	1	8	8	15	47.697	5.9E+08	6.5E+08	6.2E+08	6.6E+08	6.2E+08	6.3E+08	1.02293	0.567
P46777	60S ribosomal protein L5	RPL5	1	3	3	12.1	34.362	1.9E+08	1.8E+08	1.9E+08	1.9E+08	1.7E+08	1.7E+08	0.95221	0.4229
Q02878	60S ribosomal protein L6	RPL6	1	7	7	20.8	32.728	3.3E+08	3.7E+08	3.4E+08	4.1E+08	3.4E+08	3.6E+08	1.06238	0.41882
P18124	60S ribosomal protein L7	RPL7	1	10	10	31.9	29.225	3.7E+08	3.2E+08	4E+08	2.9E+08	3.1E+08	3.2E+08	0.8389	0.07701
P62424	60S ribosomal protein L7a	RPL7A	1	5	5	17.7	29.995	2.6E+08	2.9E+08	2.3E+08	2.4E+08	2.6E+08	2.7E+08	0.99662	0.96721
P62917	60S ribosomal protein L8	RPL8	1	5	5	15.6	28.024	3.1E+08	6.5E+08	3.7E+08	4.2E+08	6.7E+08	1.9E+08	0.96493	0.93316
P05388;Q8NHW5	60S acidic ribosomal protein P0;60S acidic ribosomal protein P	RPLP0	2	4	4	11.7	34.273	3E+08	8.7E+08	3.4E+08	3.4E+08	1E+08	3.5E+08	0.52178	0.29398
P05386	60S acidic ribosomal protein P1	RPLP1	1	1	1	14	11.514	NaN	NaN	NaN	NaN	NaN	NaN	NaN	NaN
P05387	60S acidic ribosomal protein P2	RPLP2	1	5	5	32.2	11.665	2.1E+08	3.3E+08	3E+08	1.7E+08	2.6E+08	1.6E+08	0.6976	0.16678
P04843	Dolichyl-diphosphooligosaccharide--protein glycosyltransfera	RPN1	1	6	6	9.7	68.569	2.4E+08	2.4E+08	2.4E+08	2.4E+08	2.2E+08	2.6E+08	0.98467	0.76398
P04844	Dolichyl-diphosphooligosaccharide--protein glycosyltransfera	RPN2	1	3	3	7.4	69.283	4.6E+07	5.6E+07	4.3E+07	NaN	4.6E+07	5.1E+07	1.00597	0.959
P62280	40S ribosomal protein S11	RPS11	1	5	5	26.6	18.431	2.1E+08	2.5E+08	2.3E+08	2.3E+08	2.8E+08	2.4E+08	1.08422	0.37458
P62277	40S ribosomal protein S13	RPS13	1	1	1	9.9	17.222	1.3E+08	1.6E+08	1.5E+08	NaN	1.5E+08	NaN	1.01714	NaN
P62263	40S ribosomal protein S14	RPS14	1	3	3	16.6	16.273	4.2E+08	4.6E+08	4.4E+08	4.3E+08	5.3E+08	4.6E+08	1.08371	0.27579
P62841	40S ribosomal protein S15	RPS15	1	2	2	9	17.04	2.2E+08	2.6E+08	1.9E+08	3.1E+08	2.2E+08	2.6E+08	1.1754	0.3101
P62244	40S ribosomal protein S15a	RPS15A	1	3	3	23.1	14.839	6.5E+08	7.6E+08	8.4E+08	4.5E+08	4.7E+08	8E+08	0.76994	0.24381
P62249	40S ribosomal protein S16	RPS16	1	5	5	25.3	16.445	4.3E+08	4E+08	4.7E+08	4.3E+08	4E+08	4.6E+08	0.99677	0.96006
P08708	40S ribosomal protein S17	RPS17	1	3	3	15.6	15.55	3.3E+08	3.1E+08	3.1E+08	2.4E+08	4.1E+08	3.8E+08	1.0652	0.71272
P62269	40S ribosomal protein S18	RPS18	1	5	5	25	17.718	5.8E+08	7.3E+08	5.2E+08	6E+08	5.4E+08	6.5E+08	0.97381	0.82738
P39019	40S ribosomal protein S19	RPS19	1	3	3	16.6	16.06	4E+08	4.3E+08	4.5E+08	4E+08	4.6E+08	3.4E+08	0.93696	0.48586

P15880	40S ribosomal protein S2	RPS2	1	7	7	22.5	31.324	3.8E+08	4.2E+08	4.1E+08	4.1E+08	4E+08	3.4E+08	0.95378	0.51705
P60866	40S ribosomal protein S20	RPS20	1	2	2	19.3	13.373	8.9E+07	1.4E+08	8.8E+07	1.1E+08	1E+08	9.1E+07	0.95682	0.8183
P63220	40S ribosomal protein S21	RPS21	1	1	1	12	9.1113	NaN	NaN	NaN	NaN	NaN	NaN	NaN	NaN
P62266	40S ribosomal protein S23	RPS23	1	2	2	13.3	15.807	1.2E+08	8.1E+07	2E+08	2E+08	7.9E+07	1.6E+08	1.07389	0.85031
P42677	40S ribosomal protein S27	RPS27	1	4	3	25	9.461	8E+07	6.7E+07	6.5E+07	1.1E+08	9.4E+07	8.3E+07	1.3674	0.05433
Q71UM5	40S ribosomal protein S27-like	RPS27L	1	2	1	22.6	9.4771	NaN	NaN	NaN	NaN	NaN	NaN	NaN	NaN
P23396	40S ribosomal protein S3	RPS3	1	9	9	39.9	26.688	4.8E+08	5.1E+08	5.4E+08	4.2E+08	5.3E+08	5E+08	0.95187	0.54514
P61247	40S ribosomal protein S3a	RPS3A	1	5	5	18.2	29.945	3.8E+08	4E+08	4.1E+08	4.7E+08	3.9E+08	4.4E+08	1.0956	0.2363
P62701;Q8TD47;P2209(	40S ribosomal protein S4, X isoform;40S ribosomal protein S4,	RPS4X	3	5	5	13.3	29.597	1.2E+08	1.8E+08	1.3E+08	1.4E+08	1.4E+08	1.1E+08	0.92036	0.62869
P46782	40S ribosomal protein S5;40S ribosomal protein S5, N-termina	RPS5	1	5	5	20.1	22.876	3.2E+08	3.4E+08	2.9E+08	3.8E+08	4.4E+08	3.6E+08	1.24687	0.04517
P62753	40S ribosomal protein S6	RPS6	1	4	4	16.1	28.68	9E+08	5.3E+08	8.1E+08	6.6E+08	8E+08	9.6E+08	1.08415	0.68346
P51812;Q15349	Ribosomal protein S6 kinase alpha-3;Ribosomal protein S6 kin	RPS6KA3	3	5	5	6.8	83.735	4.9E+07	4E+07	4.4E+07	5.8E+07	4.7E+07	5.8E+07	1.22268	0.08906
P62241	40S ribosomal protein S8	RPS8	1	3	3	15.9	24.205	6.1E+08	2.4E+08	NaN	NaN	6.5E+08	3.6E+08	1.18806	0.76577
P46781	40S ribosomal protein S9	RPS9	1	4	4	17	22.591	4.4E+08	3.3E+08	3.8E+08	4E+08	3.5E+08	4.5E+08	1.0437	0.71042
A0A8I5KQE6;P08865	40S ribosomal protein SA	RPSA	2	2	2	6.4	32.909	2.4E+08	2.8E+08	2.1E+08	2.6E+08	3E+08	2.5E+08	1.10931	0.34511
Q92600	Cell differentiation protein RCD1 homolog	RQCD1	1	2	2	7	33.631	NaN	1.7E+07	1.7E+07	NaN	NaN	NaN	NaN	NaN
P62070	Ras-related protein R-Ras2	RRAS2	1	2	2	12.7	23.399	NaN	NaN	NaN	NaN	NaN	NaN	NaN	NaN
Q9P2E9	Ribosome-binding protein 1	RRBP1	2	11	11	10.6	152.45	5E+07	5.1E+07	5.6E+07	4.6E+07	6E+07	5.5E+07	1.02136	0.81756
Q7LG56;P31350	Ribonucleoside-diphosphate reductase subunit M2 B;Ribonucl	RRM2B	2	2	2	5.7	40.736	NaN	NaN	NaN	NaN	NaN	NaN	NaN	NaN
Q15404	Ras suppressor protein 1	RSU1	1	5	5	14.8	31.54	1.3E+08	8.5E+07	8.9E+07	1.2E+08	9.7E+07	1.4E+08	1.18443	0.36329
O00442	RNA 3-terminal phosphate cyclase	RTCA	1	1	1	4.4	39.336	NaN	NaN	NaN	NaN	NaN	NaN	NaN	NaN
Q9Y3I0	tRNA-splicing ligase RtcB homolog	RTCB	1	8	8	15.2	55.21	1.2E+08	7.7E+07	1.1E+08	8.7E+07	1.2E+08	1.2E+08	1.06039	0.73291
Q92541	RNA polymerase-associated protein RTF1 homolog	RTF1	1	2	2	3.4	80.313	NaN	NaN	NaN	NaN	NaN	NaN	NaN	NaN
Q9Y265	RuvB-like 1	RUVBL1	1	6	6	16.9	50.227	9.5E+07	1.1E+08	1.1E+08	9.3E+07	1.4E+08	9.8E+07	1.05681	0.69409
Q9Y230	RuvB-like 2	RUVBL2	1	6	6	13.2	51.156	1E+08	9E+07	9.7E+07	1.2E+08	1.1E+08	1.2E+08	1.21633	0.0089
P60903	Protein S100-A10	S100A10	1	2	2	17.5	11.203	7.7E+08	9.6E+08	7.5E+08	8.2E+08	8E+08	5.8E+08	0.89109	0.42127
P31949	Protein S100-A11;Protein S100-A11, N-terminally processed	S100A11	1	2	2	15.2	11.74	5E+08	5.1E+08	5.5E+08	6E+08	4.2E+08	5.1E+08	0.98331	0.87799
Q99584	Protein S100-A13	S100A13	1	1	1	7.1	11.471	NaN	NaN	NaN	NaN	NaN	NaN	NaN	NaN
Q96FQ6	Protein S100-A16	S100A16	1	1	1	10.7	11.801	NaN	NaN	NaN	NaN	NaN	NaN	NaN	NaN
P26447	Protein S100-A4	S100A4	1	4	4	25.7	11.728	1.1E+09	1.4E+09	1.1E+09	1.4E+09	1.1E+09	1.2E+09	1.01504	0.91438
P06703	Protein S100-A6	S100A6	1	2	2	16.7	10.18	4E+09	3.9E+09	4.4E+09	4.9E+09	4E+09	4.1E+09	1.06299	0.45423
P25815	Protein S100-P	S100P	1	1	1	13.7	10.4	NaN	NaN	NaN	NaN	NaN	NaN	NaN	NaN
Q9NTJ5	Phosphatidylinositide phosphatase SAC1	SACM1L	1	1	1	1.7	66.966	NaN	NaN	NaN	NaN	NaN	NaN	NaN	NaN
Q9UBE0	SUMO-activating enzyme subunit 1;SUMO-activating enzyme	SAE1	1	4	4	11.8	38.449	2.6E+07	2.9E+07	2.5E+07	3.1E+07	2.7E+07	2.4E+07	1.02493	0.79558
Q9Y3Z3	Deoxynucleoside triphosphate triphosphohydrolase SAMHD1	SAMHD1	1	2	2	2.6	72.2	NaN	1.2E+07	3.2E+07	NaN	NaN	NaN	NaN	NaN
Q9Y512	Sorting and assembly machinery component 50 homolog	SAMM50	1	1	1	1.5	51.976	NaN	NaN	NaN	NaN	NaN	NaN	NaN	NaN
Q9Y6B6;Q9NR31	GTP-binding protein SAR1b;GTP-binding protein SAR1a	SAR1B	2	2	2	9.6	22.41	NaN	NaN	NaN	NaN	NaN	NaN	NaN	NaN
Q9NP81	Serine--tRNA ligase, mitochondrial	SARS2	1	1	1	2.5	58.282	NaN	NaN	NaN	NaN	NaN	NaN	NaN	NaN
Q15020	Squamous cell carcinoma antigen recognized by T-cells 3	SART3	1	1	1	1.1	109.93	NaN	NaN	NaN	NaN	NaN	NaN	NaN	NaN
Q9Y3A5	Ribosome maturation protein SBDS	SBDS	1	3	3	10	28.763	5E+07	NaN	4.8E+07	NaN	4.5E+07	NaN	0.92592	NaN
Q969E2	Secretory carrier-associated membrane protein 4	SCAMP4	1	1	1	4.8	25.728	NaN	NaN	NaN	NaN	NaN	NaN	NaN	NaN
Q14108	Lysosome membrane protein 2	SCARB2	1	2	2	2.5	54.29	8.2E+07	5.1E+07	7.7E+07	4.7E+07	6.6E+07	6.3E+07	0.8414	0.37259
O00767	Acyl-CoA desaturase	SCD	1	2	2	7	41.522	1E+08	1.2E+08	7.7E+07	1.2E+08	1.1E+08	9.4E+07	1.07031	0.64668
Q8WVM8	Sec1 family domain-containing protein 1	SCFD1	1	3	3	6.7	72.379	NaN	NaN	NaN	NaN	NaN	NaN	NaN	NaN
P22307	Non-specific lipid-transfer protein	SCP2	1	2	2	2.2	58.993	NaN	NaN	NaN	NaN	NaN	NaN	NaN	NaN
Q14160;Q9BTT6	Protein scribble homolog;Leucine-rich repeat-containing prote	SCRIB	2	2	2	1.6	174.91	NaN	NaN	NaN	NaN	NaN	NaN	NaN	NaN
Q12765	Secernin-1	SCRN1	1	2	2	5.3	46.382	NaN	1.4E+07	NaN	1.4E+07	NaN	1.2E+07	0.91612	NaN
Q96KG9	N-terminal kinase-like protein	SCYL1	1	3	3	3.8	89.63	NaN	1.8E+07	1.7E+07	1.6E+07	1.4E+07	NaN	0.83292	0.12773
P18827	Syndecan-1	SDC1	1	4	4	15.8	32.461	7.3E+07	8.8E+07	7.8E+07	6.6E+07	8E+07	7.1E+07	0.90483	0.27493
Q9BRK5	45 kDa calcium-binding protein	SDF4	1	2	2	8	41.806	NaN	NaN	NaN	NaN	NaN	NaN	NaN	NaN
P21912	Succinate dehydrogenase [ubiquinone] iron-sulfur subunit, mit	SDHB	1	1	1	3.2	31.629	NaN	NaN	NaN	NaN	NaN	NaN	NaN	NaN
Q99643	Succinate dehydrogenase cytochrome b560 subunit, mitochon	SDHC	1	1	1	4.7	18.61	NaN	NaN	NaN	NaN	NaN	NaN	NaN	NaN
Q96GA7	Serine dehydratase-like	SDSL	1	2	2	7.3	34.674	NaN	NaN	NaN	NaN	NaN	NaN	NaN	NaN
P67812	Signal peptidase complex catalytic subunit SEC11A	SEC11A	1	1	1	4.5	20.625	NaN	NaN	NaN	NaN	NaN	NaN	NaN	NaN
P55735	Protein SEC13 homolog	SEC13	1	1	1	3.4	35.54	NaN	NaN	NaN	NaN	NaN	NaN	NaN	NaN
O75396	Vesicle-trafficking protein SEC22b	SEC22B	1	1	1	6.5	24.74	NaN	NaN	NaN	NaN	NaN	NaN	NaN	NaN
Q15436	Protein transport protein Sec23A	SEC23A	1	7	7	10.6	86.16	5E+07	6.7E+07	4.9E+07	6.9E+07	5E+07	7.1E+07	1.13393	0.44384
Q15437	Protein transport protein Sec23B	SEC23B	1	3	3	5	86.478	NaN	NaN	NaN	NaN	NaN	NaN	NaN	NaN
Q9Y6Y8	SEC23-interacting protein	SEC23IP	1	3	3	3.7	111.08	2.9E+07	2.9E+07	3.4E+07	2.2E+07	2.8E+07	2.8E+07	0.84498	0.12479

P53992	Protein transport protein Sec24C	SEC24C	1	2	2	2.1	118.32	NaN	NaN	NaN	NaN	NaN	NaN	NaN	NaN
O94979	Protein transport protein Sec31A	SEC31A	1	3	3	2.6	133.01	4.5E+07	5.2E+07	4.8E+07	3.8E+07	5E+07	4.3E+07	0.90539	0.32462
P61619	Protein transport protein Sec61 subunit alpha isoform 1	SEC61A1	2	3	3	5.9	52.264	1.3E+08	1.4E+08	1.6E+08	1.4E+08	1.4E+08	1.5E+08	1.02262	0.78171
P60468	Protein transport protein Sec61 subunit beta	SEC61B	1	3	3	37.5	9.9743	1.1E+08	8.9E+07	1.1E+08	1.1E+08	9.7E+07	1E+08	1.04002	0.62341
Q99442	Translocation protein SEC62	SEC62	1	2	2	5.5	45.861	3.1E+07	3.9E+07	NaN	4.7E+07	NaN	3.1E+07	1.09761	0.73806
Q9UGP8	Translocation protein SEC63 homolog	SEC63	1	3	3	4.2	87.996	1.6E+07	1.5E+07	1.3E+07	1.5E+07	1.2E+07	1.3E+07	0.90563	0.38207
Q9UBV2	Protein sel-1 homolog 1	SEL1L	1	1	1	2	88.754	NaN	NaN	NaN	NaN	NaN	NaN	NaN	NaN
P62341	Selenoprotein T	SELT	1	1	1	6.7	22.324	NaN	NaN	NaN	NaN	NaN	NaN	NaN	NaN
Q8NC51	Plasminogen activator inhibitor 1 RNA-binding protein	SERBP1	1	6	6	18.6	44.965	1.3E+08	1.1E+08	2.1E+08	2E+08	1E+08	1.6E+08	1.00819	0.97772
P30740	Leukocyte elastase inhibitor	SERPINB1	1	4	4	7.7	42.741	1.1E+08	8E+07	1.3E+08	1.2E+08	1.2E+08	1.2E+08	1.1296	0.36996
P50453	Serpin B9	SERPINB9	1	3	3	10.1	42.403	2.6E+07	2.7E+07	2.8E+07	2.3E+07	2.7E+07	2.6E+07	0.93888	0.29707
P05121	Plasminogen activator inhibitor 1	SERPINE1	1	1	1	2.5	45.059	NaN	NaN	NaN	NaN	NaN	NaN	NaN	NaN
P50454	Serpin H1	SERPINH1	1	3	3	8.6	46.44	2.4E+07	3.1E+07	3.1E+07	NaN	2.8E+07	2.7E+07	0.95117	0.65492
Q01105;P0DME0	Protein SET;Protein SETSIP	SET	2	2	2	13.4	33.488	3.6E+07	1E+08	NaN	4.3E+07	5.7E+07	4.4E+07	0.69949	0.47296
Q86TU7	Histone-lysine N-methyltransferase setd3	SETD3	1	1	1	1.5	67.256	NaN	NaN	NaN	NaN	NaN	NaN	NaN	NaN
Q15637	Splicing factor 1	SF1	1	1	1	2	68.329	NaN	NaN	NaN	NaN	NaN	NaN	NaN	NaN
Q15459	Splicing factor 3A subunit 1	SF3A1	1	4	4	9	88.885	2.3E+07	NaN	2.4E+07	2.3E+07	2.1E+07	2.4E+07	0.97242	0.68505
Q12874	Splicing factor 3A subunit 3	SF3A3	1	4	4	12	58.848	1.2E+07	9767700	1E+07	1.1E+07	1.3E+07	1.2E+07	1.123	0.24832
O75533	Splicing factor 3B subunit 1	SF3B1	1	9	9	8.7	145.83	4.4E+07	4.9E+07	4.3E+07	5.1E+07	4.5E+07	3.9E+07	0.99976	0.99794
Q13435	Splicing factor 3B subunit 2	SF3B2	1	1	1	1.3	100.23	NaN	NaN	NaN	NaN	NaN	NaN	NaN	NaN
Q15393	Splicing factor 3B subunit 3	SF3B3	1	5	5	6	135.58	7.4E+07	5.9E+07	NaN	9E+07	6.8E+07	7.4E+07	1.16192	0.35993
P31947	14-3-3 protein sigma	SFN	1	5	4	20.2	27.774	9.9E+08	NaN	8E+08	NaN	NaN	NaN	NaN	NaN
P23246	Splicing factor, proline- and glutamine-rich	SFPQ	1	6	5	8.3	76.149	1E+08	1E+08	1.2E+08	1.2E+08	9.4E+07	9.9E+07	0.95369	0.6714
Q9H9B4	Sideroflexin-1	SFXN1	1	2	2	7.1	35.619	2.7E+07	2.7E+07	2.6E+07	3.1E+07	2.6E+07	2.5E+07	1.02852	0.70351
Q9BWM7	Sideroflexin-3	SFXN3	1	3	3	10.3	35.503	3.3E+07	3.4E+07	3.5E+07	2.9E+07	2.1E+07	NaN	0.73827	0.0735
Q9BX95	Sphingosine-1-phosphate phosphatase 1	SGPP1	1	2	2	5.7	49.107	NaN	NaN	NaN	NaN	NaN	NaN	NaN	NaN
O43765	Small glutamine-rich tetratricopeptide repeat-containing prote	SGTA	1	1	1	3.8	34.063	NaN	NaN	NaN	NaN	NaN	NaN	NaN	NaN
Q7L8J4	SH3 domain-binding protein 5-like	SH3BP5L	1	1	1	3.1	43.499	NaN	NaN	NaN	NaN	NaN	NaN	NaN	NaN
Q99961	Endophilin-A2	SH3GL1	2	3	3	12.2	41.489	1.6E+07	2E+07	1.8E+07	1.5E+07	1.5E+07	1.7E+07	0.8669	0.13836
Q96B97	SH3 domain-containing kinase-binding protein 1	SH3KBP1	1	1	1	1.7	73.125	1.6E+07	NaN	2.8E+07	4.4E+07	2.7E+07	2.7E+07	1.48195	0.29808
P29353	SHC-transforming protein 1	SHC1	1	1	1	2.2	62.821	NaN	NaN	NaN	NaN	NaN	NaN	NaN	NaN
P34897	Serine hydroxymethyltransferase, mitochondrial	SHMT2	1	5	5	10.5	55.992	9.9E+07	1.2E+08	1E+08	8.6E+07	9.6E+07	1E+08	0.88989	0.20064
Q8IXJ6	NAD-dependent protein deacetylase sirtuin-2	SIRT2	1	1	1	3.1	43.182	NaN	NaN	NaN	NaN	NaN	NaN	NaN	NaN
P42285	Superkiller viralicidic activity 2-like 2	SKIV2L2	1	4	4	4.5	117.8	1.2E+07	NaN	1.2E+07	7734700	9330100	1E+07	0.75774	0.06589
P63208	S-phase kinase-associated protein 1	SKP1	1	2	2	8.6	18.658	2.5E+08	3.4E+08	2.2E+08	2.9E+08	2.5E+08	2E+08	0.92292	0.66587
P36021	Monocarboxylate transporter 8	SLC16A2	1	1	1	2.4	59.511	NaN	NaN	NaN	NaN	NaN	NaN	NaN	NaN
P43007	Neutral amino acid transporter A	SLC1A4	1	1	1	2.1	55.722	NaN	NaN	NaN	NaN	NaN	NaN	NaN	NaN
Q15758	Neutral amino acid transporter B(0)	SLC1A5	1	2	2	3.9	56.598	NaN	NaN	NaN	NaN	NaN	NaN	NaN	NaN
O75751	Solute carrier family 22 member 3	SLC22A3	1	1	1	2.3	61.279	NaN	NaN	NaN	NaN	NaN	NaN	NaN	NaN
P53007	Tricarboxylate transport protein, mitochondrial	SLC25A1	1	1	1	3.5	34.012	NaN	NaN	NaN	NaN	NaN	NaN	NaN	NaN
Q02978	Mitochondrial 2-oxoglutarate/malate carrier protein	SLC25A11	1	2	2	7.3	34.061	NaN	NaN	NaN	3.3E+07	3E+07	2.8E+07	NaN	NaN
Q9UJS0	Calcium-binding mitochondrial carrier protein Aralar2	SLC25A13	1	2	2	3.3	74.175	NaN	NaN	NaN	NaN	NaN	NaN	NaN	NaN
Q9H936	Mitochondrial glutamate carrier 1	SLC25A22	1	1	1	4.6	34.47	NaN	NaN	NaN	NaN	NaN	NaN	NaN	NaN
Q6NUK1	Calcium-binding mitochondrial carrier protein SCaMC-1	SLC25A24	1	7	7	16.8	53.354	1.1E+08	1.3E+08	1E+08	8.6E+07	1E+08	9.5E+07	0.83437	0.15831
Q00325	Phosphate carrier protein, mitochondrial	SLC25A3	1	3	3	9.1	40.094	1.6E+08	2.6E+08	1.8E+08	1.4E+08	2.8E+08	1.7E+08	0.98912	0.96815
Q6ZT89	Solute carrier family 25 member 48	SLC25A48	1	1	1	2.9	33.439	NaN	NaN	NaN	NaN	NaN	NaN	NaN	NaN
P05141;P12236	ADP/ATP translocase 2;ADP/ATP translocase 2, N-terminally p	SLC25A5	3	5	3	14.1	32.852	6.2E+08	4.4E+08	6.1E+08	6.3E+08	3.6E+08	6.4E+08	0.97848	0.91796
O14975	Very long-chain acyl-CoA synthetase	SLC27A2	1	2	2	4.8	70.311	2.4E+07	2.7E+07	2.5E+07	2.7E+07	NaN	2.9E+07	1.10563	0.13804
P11166	Solute carrier family 2, facilitated glucose transporter member	SLC2A1	1	1	1	2	54.083	NaN	NaN	NaN	NaN	NaN	NaN	NaN	NaN
Q9Y6M5	Zinc transporter 1	SLC30A1	1	1	1	3.6	55.299	NaN	NaN	NaN	NaN	NaN	NaN	NaN	NaN
Q8NEW0	Zinc transporter 7	SLC30A7	1	3	3	9.6	41.625	NaN	NaN	NaN	NaN	NaN	NaN	NaN	NaN
O00400	Acetyl-coenzyme A transporter 1	SLC33A1	1	1	1	2.6	60.908	NaN	NaN	NaN	NaN	NaN	NaN	NaN	NaN
Q8N357	Solute carrier family 35 member F6	SLC35F6	1	2	2	5.1	40.214	NaN	3.9E+07	3.2E+07	3.9E+07	NaN	3.9E+07	1.10607	0.40167
P08195	4F2 cell-surface antigen heavy chain	SLC3A2	1	12	12	18.6	67.993	1.8E+08	1.9E+08	2E+08	1.9E+08	2.2E+08	1.7E+08	1.01697	0.85128
P04920	Anion exchange protein 2	SLC4A2	1	2	2	3.4	137.01	NaN	NaN	NaN	NaN	NaN	NaN	NaN	NaN
P31641	Sodium- and chloride-dependent taurine transporter	SLC6A6	1	2	2	4.5	69.829	2.1E+07	2.2E+07	2E+07	NaN	2.1E+07	NaN	1.00465	NaN
Q01650	Large neutral amino acids transporter small subunit 1	SLC7A5	1	3	3	8.5	55.01	1.3E+08	1.8E+08	1.3E+08	1.4E+08	1.3E+08	1.4E+08	0.8995	0.45724
O14745	Na(+)/H(+) exchange regulatory cofactor NHE-RF1	SLC9A3R1	1	4	4	14	38.868	NaN	NaN	NaN	NaN	NaN	NaN	NaN	NaN
Q9GZT3	SRA stem-loop-interacting RNA-binding protein, mitochondri	SLIRP	1	2	2	23.9	12.349	NaN	2.9E+07	1.7E+07	2.5E+07	NaN	NaN	1.0844	NaN

Q14BN4	Sarcolemmal membrane-associated protein	SLMAP	1	1	1	1.7	95.197	NaN	NaN	NaN	NaN	NaN	NaN	NaN	NaN
P84022;Q15796;O1519	Mothers against decapentaplegic homolog 3;Mothers against	SMAD3	3	1	1	3.1	48.08	NaN	NaN	NaN	NaN	NaN	NaN	NaN	NaN
P28370	Probable global transcription activator SNF2L1	SMARCA1	1	1	1	1.4	122.6	NaN	NaN	NaN	NaN	NaN	NaN	NaN	NaN
P51531;P51532	Probable global transcription activator SNF2L2;Transcription ac	SMARCA2	2	1	1	0.8	181.28	NaN	NaN	NaN	NaN	NaN	NaN	NaN	NaN
O60264	SWI/SNF-related matrix-associated actin-dependent regulator	SMARCA5	1	1	1	1.4	121.9	NaN	NaN	NaN	NaN	NaN	NaN	NaN	NaN
Q9NZC9	SWI/SNF-related matrix-associated actin-dependent regulator	SMARCAL1	1	1	1	1.2	105.94	NaN	NaN	NaN	NaN	NaN	NaN	NaN	NaN
Q92922	SWI/SNF complex subunit SMARCC1	SMARCC1	1	3	1	3.1	122.87	2.9E+07	1.6E+07	NaN	2.4E+07	NaN	NaN	1.0687	NaN
Q8TAQ2	SWI/SNF complex subunit SMARCC2	SMARCC2	1	3	1	3	132.88	NaN	NaN	NaN	NaN	NaN	NaN	NaN	NaN
Q14683	Structural maintenance of chromosomes protein 1A	SMC1A	1	4	4	4	143.23	NaN	NaN	NaN	NaN	NaN	NaN	NaN	NaN
O95347	Structural maintenance of chromosomes protein 2	SMC2	1	3	3	2.8	135.65	NaN	NaN	NaN	NaN	NaN	NaN	NaN	NaN
Q9UQE7	Structural maintenance of chromosomes protein 3	SMC3	1	3	3	3.3	141.54	NaN	NaN	NaN	NaN	NaN	NaN	NaN	NaN
Q9NTJ3	Structural maintenance of chromosomes protein 4	SMC4	1	5	5	4.2	147.18	3E+07	2.6E+07	2.8E+07	3.5E+07	2.5E+07	2.5E+07	1.00581	0.96635
Q6IN85	Serine/threonine-protein phosphatase 4 regulatory subunit 3A	SMEK1	1	1	1	1.4	95.367	NaN	NaN	NaN	NaN	NaN	NaN	NaN	NaN
Q16637	Survival motor neuron protein	SMN1	1	2	2	10.2	31.848	NaN	NaN	NaN	NaN	NaN	NaN	NaN	NaN
P53814	Smoothelin	SMTN	1	1	1	1.5	99.058	NaN	NaN	NaN	NaN	NaN	NaN	NaN	NaN
Q2TAY7	WD40 repeat-containing protein SMU1;WD40 repeat-containi	SMU1	1	1	1	2.5	57.543	NaN	NaN	NaN	NaN	NaN	NaN	NaN	NaN
Q7KZF4	Staphylococcal nuclease domain-containing protein 1	SND1	1	6	6	7.1	102	2E+08	2.5E+08	2.1E+08	1.7E+08	2E+08	1.9E+08	0.86358	0.2021
Q96H20	Vacuolar-sorting protein SNF8	SNF8	1	1	1	4.7	28.864	NaN	NaN	NaN	NaN	NaN	NaN	NaN	NaN
O75643	U5 small nuclear ribonucleoprotein 200 kDa helicase	SNRNP200	1	4	4	2.3	244.5	3.4E+07	3.6E+07	2.9E+07	3.5E+07	3.1E+07	3E+07	0.95901	0.62241
P08621	U1 small nuclear ribonucleoprotein 70 kDa	SNRNP70	1	7	7	24.3	51.556	8.1E+07	7.6E+07	8E+07	8.3E+07	7.2E+07	7.5E+07	0.96776	0.52288
P09012	U1 small nuclear ribonucleoprotein A	SNRPA	1	1	1	10.3	31.279	NaN	NaN	NaN	NaN	NaN	NaN	NaN	NaN
P09661	U2 small nuclear ribonucleoprotein A	SNRPA1	1	1	1	5.1	28.415	NaN	NaN	NaN	NaN	NaN	NaN	NaN	NaN
P09234	U1 small nuclear ribonucleoprotein C	SNRPC	1	1	1	7.5	17.394	NaN	NaN	NaN	NaN	NaN	NaN	NaN	NaN
P62314	Small nuclear ribonucleoprotein Sm D1	SNRPD1	1	1	1	9.2	13.281	NaN	NaN	NaN	NaN	NaN	NaN	NaN	NaN
P62316	Small nuclear ribonucleoprotein Sm D2	SNRPD2	1	4	4	27.1	13.527	1.1E+08	NaN	1.6E+08	4.1E+07	9.5E+07	1E+08	0.59517	0.17865
P62318	Small nuclear ribonucleoprotein Sm D3	SNRPD3	1	1	1	7.9	13.916	NaN	NaN	NaN	NaN	NaN	NaN	NaN	NaN
P62308;A8MWD9	Small nuclear ribonucleoprotein G;Putative small nuclear ribon	SNRPG	2	1	1	9.2	8.496	NaN	NaN	NaN	NaN	NaN	NaN	NaN	NaN
P63162;P14678	Small nuclear ribonucleoprotein-associated protein N;Small nu	SNRPN	2	1	1	3.3	24.614	NaN	NaN	NaN	NaN	NaN	NaN	NaN	NaN
Q13573	SNW domain-containing protein 1	SNW1	1	1	1	2.8	61.494	NaN	NaN	NaN	NaN	NaN	NaN	NaN	NaN
Q13596	Sorting nexin-1	SNX1	2	4	4	7.7	59.069	3E+07	3E+07	2.8E+07	3E+07	2.7E+07	3E+07	0.99998	0.99961
Q9H3E2	Sorting nexin-25	SNX25	1	1	1	1.5	97.944	NaN	NaN	NaN	NaN	NaN	NaN	NaN	NaN
O60493	Sorting nexin-3	SNX3	1	2	2	15.4	18.762	1.6E+07	2.2E+07	1.4E+07	1.8E+07	2.1E+07	1.4E+07	1.03127	0.87314
Q9Y5X3	Sorting nexin-5	SNX5	1	1	1	2.5	46.816	NaN	NaN	NaN	NaN	NaN	NaN	NaN	NaN
Q9UNH7	Sorting nexin-6;Sorting nexin-6, N-terminally processed	SNX6	1	2	2	4.4	46.648	2.2E+07	NaN	2.4E+07	2.1E+07	2.3E+07	2.2E+07	0.97942	0.63916
Q9Y5X2	Sorting nexin-8	SNX8	1	1	1	2.4	52.569	NaN	NaN	NaN	NaN	NaN	NaN	NaN	NaN
P00441	Superoxide dismutase [Cu-Zn]	SOD1	1	3	3	23.4	15.936	NaN	2.8E+07	4.9E+07	NaN	NaN	NaN	NaN	NaN
Q00796	Sorbitol dehydrogenase	SORD	1	1	1	2.5	38.324	NaN	NaN	NaN	NaN	NaN	NaN	NaN	NaN
O75391	Sperm-associated antigen 7	SPAG7	1	2	2	11.5	26.034	2E+07	1.7E+07	NaN	1.2E+07	1.6E+07	NaN	0.7665	0.21699
O60271	C-Jun-amino-terminal kinase-interacting protein 4	SPAG9	1	1	1	1.4	146.2	NaN	NaN	NaN	NaN	NaN	NaN	NaN	NaN
Q9NUQ6	SPATS2-like protein	SPATS2L	1	1	1	2.2	61.728	NaN	NaN	NaN	NaN	NaN	NaN	NaN	NaN
Q9Y6A9	Signal peptidase complex subunit 1	SPCS1	1	1	1	10.1	18.298	4.7E+07	NaN	4.8E+07	2.6E+07	4.6E+07	4.7E+07	0.82956	0.42133
Q15005	Signal peptidase complex subunit 2	SPCS2	1	1	1	9.7	25.003	NaN	NaN	NaN	NaN	NaN	NaN	NaN	NaN
P61009	Signal peptidase complex subunit 3	SPCS3	1	2	2	11.1	20.313	3.2E+07	NaN	NaN	3.9E+07	4.1E+07	NaN	1.2496	NaN
Q5M775	Cytospin-B	SPECC1	1	1	1	1.3	118.58	NaN	NaN	NaN	NaN	NaN	NaN	NaN	NaN
O43291	Kunitz-type protease inhibitor 2	SPINT2	1	1	1	4.8	28.228	NaN	NaN	NaN	NaN	NaN	NaN	NaN	NaN
P35270	Sepiapterin reductase	SPR	1	2	2	7.7	28.048	3.6E+07	3.3E+07	4.3E+07	5.5E+07	3.9E+07	4.6E+07	1.23114	0.18663
Q13813	Spectrin alpha chain, non-erythrocytic 1	SPTAN1	1	13	13	5.9	284.54	1.4E+08	1.4E+08	1.6E+08	1.6E+08	1.3E+08	1.5E+08	1.01084	0.89563
Q01082	Spectrin beta chain, non-erythrocytic 1	SPTBN1	3	25	25	11.7	274.61	1.4E+08	1.3E+08	1.4E+08	1.4E+08	1.3E+08	1.4E+08	1.02607	0.61538
O15269	Serine palmitoyltransferase 1	SPTLC1	1	2	2	3.8	52.743	2.5E+07	2.2E+07	1.8E+07	2.7E+07	2.2E+07	2.4E+07	1.12395	0.31724
Q9Y6N5	Sulfide:quinone oxidoreductase, mitochondrial	SQRDL	1	4	4	10.9	49.96	6.8E+07	7.6E+07	7.9E+07	6.6E+07	7.3E+07	8.7E+07	1.00927	0.92556
P12931;Q9H3Y6;P4268	Proto-oncogene tyrosine-protein kinase Src;Tyrosine-protein	SRC	8	2	2	3.4	59.834	2.8E+07	2.9E+07	NaN	3E+07	NaN	2.7E+07	0.99212	0.90629
P19623	Spermidine synthase	SRM	1	2	2	9.9	33.824	1.8E+07	NaN	NaN	NaN	1.5E+07	1.6E+07	0.87206	NaN
P37108	Signal recognition particle 14 kDa protein	SRP14	1	2	2	19.9	14.57	NaN	NaN	NaN	NaN	NaN	NaN	NaN	NaN
P09132	Signal recognition particle 19 kDa protein	SRP19	1	1	1	10.4	16.156	NaN	NaN	NaN	NaN	NaN	NaN	NaN	NaN
P61011	Signal recognition particle 54 kDa protein	SRP54	1	1	1	2.8	55.704	NaN	NaN	NaN	NaN	NaN	NaN	NaN	NaN
Q9UHB9	Signal recognition particle subunit SRP68	SRP68	1	3	3	5.9	70.729	2.4E+07	3.3E+07	9879800	3.2E+07	2.5E+07	2.3E+07	1.20418	0.56762
O76094	Signal recognition particle subunit SRP72	SRP72	1	2	2	3.9	74.605	NaN	NaN	NaN	NaN	NaN	NaN	NaN	NaN
P49458	Signal recognition particle 9 kDa protein	SRP9	1	1	1	10.5	10.112	NaN	NaN	NaN	NaN	NaN	NaN	NaN	NaN
P78362;Q9UPE1;Q96SB	SRSF protein kinase 2;SRSF protein kinase 2 N-terminal;SRSF p	SRPK2	3	2	2	3.1	77.526	1.7E+07	9903000	2.3E+07	2.2E+07	1.8E+07	1.6E+07	1.13163	0.61445

Q9BXP5	Serrate RNA effector molecule homolog	SRRT	1	5	5	4.9	100.67	3.6E+07	NaN	4.5E+07	5.3E+07	3.1E+07	4.2E+07	1.03653	0.8811
Q07955	Serine/arginine-rich splicing factor 1	SRSF1	1	6	6	25.8	27.744	5.3E+07	4.4E+07	5.4E+07	5.4E+07	5.1E+07	6E+07	1.09429	0.30185
Q01130;Q9BRL6	Serine/arginine-rich splicing factor 2;Serine/arginine-rich splici	SRSF2	2	4	4	18.1	25.476	2.6E+08	3.1E+08	2.7E+08	3.7E+08	2.8E+08	2.8E+08	1.1091	0.40093
P84103	Serine/arginine-rich splicing factor 3	SRSF3	1	2	2	10.4	19.329	2.1E+07	NaN	NaN	NaN	NaN	2.6E+07	1.1958	NaN
Q08170	Serine/arginine-rich splicing factor 4	SRSF4	1	3	2	6.7	56.678	NaN	NaN	NaN	NaN	NaN	NaN	NaN	NaN
Q13247	Serine/arginine-rich splicing factor 6	SRSF6	1	3	2	9.6	39.586	NaN	NaN	NaN	NaN	NaN	NaN	NaN	NaN
Q16629	Serine/arginine-rich splicing factor 7	SRSF7	1	3	3	12.2	27.366	NaN	NaN	NaN	5.1E+07	NaN	4.5E+07	NaN	NaN
Q9BYN0	Sulfiredoxin-1	SRXN1	1	1	1	15.3	14.259	NaN	NaN	NaN	NaN	NaN	NaN	NaN	NaN
P05455	Lupus La protein	SSB	1	8	8	16.9	46.836	1.9E+08	2E+08	1.9E+08	2.2E+08	1.9E+08	2E+08	1.04297	0.49982
Q04837	Single-stranded DNA-binding protein, mitochondrial	SSBP1	1	2	2	11.5	17.259	1.2E+08	9E+07	3.8E+07	8.2E+07	1.1E+08	9.9E+07	1.16073	0.6208
Q9UNL2	Translocon-associated protein subunit gamma	SSR3	1	1	1	7.6	21.08	NaN	NaN	NaN	NaN	NaN	NaN	NaN	NaN
P51571	Translocon-associated protein subunit delta	SSR4	1	1	1	6.4	18.998	NaN	NaN	NaN	NaN	NaN	NaN	NaN	NaN
Q08945	FACT complex subunit SSRP1	SSRP1	1	3	3	4.5	81.074	NaN	6.9E+07	4.6E+07	4.8E+07	5.5E+07	5.7E+07	0.92742	0.6828
Q9NP77	RNA polymerase II subunit A C-terminal domain phosphatase	SSU72	1	1	1	6.2	22.574	NaN	NaN	NaN	NaN	NaN	NaN	NaN	NaN
P50502;Q8IZP2;Q8NFI4	Hsc70-interacting protein;Putative protein FAM10A4;Putative p	ST13	3	2	2	12.5	41.331	NaN	NaN	NaN	NaN	NaN	NaN	NaN	NaN
P42224	Signal transducer and activator of transcription 1-alpha/beta	STAT1	1	4	4	5.1	87.334	9.6E+07	1.1E+08	9.2E+07	1E+08	1.1E+08	1E+08	1.09062	0.14776
P40763	Signal transducer and activator of transcription 3	STAT3	1	5	5	5.7	88.067	NaN	NaN	NaN	NaN	NaN	NaN	NaN	NaN
O95793	Double-stranded RNA-binding protein Staufen homolog 1	STAU1	1	3	3	6.1	63.182	NaN	1E+07	1E+07	NaN	NaN	8993700	0.87739	NaN
Q9NUL3	Double-stranded RNA-binding protein Staufen homolog 2	STAU2	1	2	2	4.9	62.608	9917200	NaN	1.5E+07	NaN	NaN	NaN	NaN	NaN
Q658P3	Metalloreductase STEAP3	STEAP3	1	1	1	2.5	54.6	NaN	NaN	NaN	NaN	NaN	NaN	NaN	NaN
Q13586	Stromal interaction molecule 1	STIM1	1	2	2	3.1	77.422	2.5E+07	NaN	2.4E+07	3.1E+07	2.3E+07	2.1E+07	1.02503	0.88327
P31948	Stress-induced-phosphoprotein 1	STIP1	1	10	10	16.4	62.639	2.7E+08	2.5E+08	2.4E+08	2E+08	3E+08	3.1E+08	1.06684	0.67482
Q9Y6E0	Serine/threonine-protein kinase 24;Serine/threonine-protein k	STK24	1	1	1	3.2	49.307	NaN	NaN	NaN	NaN	NaN	NaN	NaN	NaN
Q15208	Serine/threonine-protein kinase 38	STK38	1	1	1	2.2	54.19	NaN	NaN	NaN	NaN	NaN	NaN	NaN	NaN
Q9UEW8;O95747	STE20/SPS1-related proline-alanine-rich protein kinase;Serine/	STK39	2	2	2	3.3	59.473	3.3E+07	NaN	3.3E+07	2.2E+07	3.5E+07	NaN	0.88817	0.63136
P16949	Stathmin	STMN1	2	4	4	20.8	17.302	6E+07	5.1E+07	7.1E+07	5.7E+07	5.6E+07	6E+07	0.94979	0.63585
P27105	Erythrocyte band 7 integral membrane protein	STOM	1	1	1	4.9	31.73	NaN	NaN	NaN	NaN	NaN	NaN	NaN	NaN
Q9UJZ1	Stomatin-like protein 2, mitochondrial	STOML2	1	2	2	8.7	38.534	3.4E+07	1.4E+07	3.6E+07	3.4E+07	2.8E+07	3E+07	1.10184	0.71823
Q9Y3F4	Serine-threonine kinase receptor-associated protein	STRAP	1	1	1	4.3	38.438	6.9E+07	NaN	8E+07	8.4E+07	6.8E+07	7.3E+07	1.00796	0.93839
P46977	Dolichyl-diphosphooligosaccharide--protein glycosyltransfera	STT3A	1	2	2	2.4	80.529	NaN	NaN	NaN	NaN	NaN	NaN	NaN	NaN
Q8TCJ2	Dolichyl-diphosphooligosaccharide--protein glycosyltransfera	STT3B	1	2	2	2.3	93.673	NaN	5.1E+07	4.8E+07	NaN	NaN	NaN	NaN	NaN
Q9UNE7	E3 ubiquitin-protein ligase CHIP	STUB1	1	2	2	7.9	34.856	NaN	NaN	NaN	NaN	NaN	NaN	NaN	NaN
O14662	Syntaxin-16	STX16	1	1	1	4.3	37.031	NaN	NaN	NaN	NaN	NaN	NaN	NaN	NaN
P56962	Syntaxin-17	STX17	1	2	2	6.3	33.403	1.7E+07	1.7E+07	8238200	NaN	1.6E+07	1.7E+07	1.18015	0.54666
Q12846	Syntaxin-4	STX4	1	1	1	3.7	34.18	NaN	NaN	NaN	NaN	NaN	NaN	NaN	NaN
O15400	Syntaxin-7	STX7	1	1	1	5	29.815	NaN	NaN	NaN	NaN	NaN	NaN	NaN	NaN
O00186	Syntaxin-binding protein 3	STXBP3	1	3	3	6.4	67.764	NaN	NaN	NaN	NaN	NaN	NaN	NaN	NaN
P53999	Activated RNA polymerase II transcriptional coactivator p15	SUB1	1	5	5	44.1	14.395	1.9E+08	1.5E+08	1.7E+08	1.6E+08	1.7E+08	1.8E+08	0.99032	0.90222
P53597	Succinyl-CoA ligase [ADP/GDP-forming] subunit alpha, mitoch	SUCLG1	1	2	2	7.5	36.249	3.5E+07	3.2E+07	3.2E+07	3.3E+07	3.5E+07	2.8E+07	0.97538	0.74041
Q96I99	Succinyl-CoA ligase [GDP-forming] subunit beta, mitochondria	SUCLG2	1	3	3	7.9	46.51	NaN	NaN	NaN	NaN	NaN	NaN	NaN	NaN
Q9Y2Z0	Suppressor of G2 allele of SKP1 homolog	SUGT1	1	1	1	2.7	41.024	NaN	NaN	NaN	NaN	NaN	NaN	NaN	NaN
Q8NBJ7	Sulfatase-modifying factor 2	SUMF2	1	3	3	12.3	33.843	3.1E+07	2.5E+07	NaN	2.8E+07	3.1E+07	3E+07	1.0635	0.52468
G2XKQ0;P63165	Small ubiquitin-related modifier;Small ubiquitin-related modifi	SUMO1	2	2	2	18.8	11.526	3.2E+07	NaN	NaN	3.7E+07	NaN	3.4E+07	1.11906	NaN
P61956;Q6EEV6;P55854	Small ubiquitin-related modifier 2;Small ubiquitin-related mod	SUMO2	3	2	2	23.2	10.871	NaN	NaN	NaN	NaN	NaN	NaN	NaN	NaN
Q9Y5B9	FACT complex subunit SPT16	SUPT16H	1	3	3	2.8	119.91	5.1E+07	5E+07	4.8E+07	3.7E+07	3.8E+07	4.6E+07	0.80727	0.028
O00267	Transcription elongation factor SPT5	SUPT5H	1	5	5	6.8	121	3.4E+07	2.8E+07	2.5E+07	2.6E+07	3.5E+07	2.7E+07	1.01968	0.89478
Q8IYB8	ATP-dependent RNA helicase SUPV3L1, mitochondrial	SUPV3L1	1	2	2	3.2	87.99	9880100	1.5E+07	NaN	NaN	1.1E+07	6473400	0.7203	0.42568
Q9UH65	Switch-associated protein 70	SWAP70	1	2	2	3.6	68.997	2.4E+07	NaN	2.3E+07	1.5E+07	2.5E+07	NaN	0.84896	0.5194
O60506	Heterogeneous nuclear ribonucleoprotein Q	SYNCRIP	1	6	4	13.5	69.602	4.1E+07	3.9E+07	5E+07	4.7E+07	4.7E+07	3.1E+07	0.96605	0.83044
Q8WXH0	Nesprin-2	SYNE2	1	1	1	0.2	796.43	NaN	NaN	NaN	NaN	NaN	NaN	NaN	NaN
O43760	Synaptogyrin-2	SYNGR2	1	2	2	8	24.81	9.2E+07	NaN	NaN	9.6E+07	NaN	8.5E+07	0.98635	NaN
Q01995	Transgelin	TAGLN	2	6	6	24.4	22.611	5.6E+08	5.3E+08	5.8E+08	5.6E+08	5.4E+08	5.2E+08	0.96567	0.38151
P37802	Transgelin-2	TAGLN2	1	5	5	21.6	22.391	1.8E+09	3.4E+09	1.5E+09	1.5E+09	2.1E+09	1.1E+09	0.69202	0.36564
P37837	Transaldolase	TALDO1	1	8	8	24.9	37.54	4.3E+08	4.4E+08	4.1E+08	4.4E+08	4.1E+08	4.3E+08	0.99729	0.93221
Q13148	TAR DNA-binding protein 43	TARDBP	1	1	1	2.9	44.739	NaN	NaN	NaN	NaN	NaN	NaN	NaN	NaN
P26639	Threonine--tRNA ligase, cytoplasmic	TARS	1	10	10	16.7	83.434	1.1E+08	1.2E+08	1.3E+08	1.3E+08	1.2E+08	1.1E+08	0.98728	0.81095
Q9BW92	Threonine--tRNA ligase, mitochondrial	TARS2	1	1	1	1.7	81.035	NaN	NaN	NaN	NaN	NaN	NaN	NaN	NaN
Q6P1N9	Putative deoxyribonuclease TATDN1	TATDN1	1	2	2	8.1	33.601	3E+07	3.2E+07	NaN	3.9E+07	2.6E+07	3.2E+07	1.03513	0.84424
Q4KMP7	TBC1 domain family member 10B	TBC1D10B	1	1	1	1.2	87.198	NaN	NaN	NaN	NaN	NaN	NaN	NaN	NaN

Q8TC07	TBC1 domain family member 15	TBC1D15	1	1	1	1.3	79.49	NaN	NaN	NaN	NaN	NaN	NaN	NaN	NaN
Q9NUY8	TBC1 domain family member 23	TBC1D23	1	1	1	2.4	78.321	NaN	NaN	NaN	NaN	NaN	NaN	NaN	NaN
Q9UPU7	TBC1 domain family member 2B	TBC1D2B	1	1	1	1.2	109.88	NaN	NaN	NaN	NaN	NaN	NaN	NaN	NaN
O60343	TBC1 domain family member 4	TBC1D4	1	1	1	1.2	146.56	2.1E+07	1.6E+07	NaN	NaN	2.1E+07	NaN	1.14079	NaN
Q66K14	TBC1 domain family member 9B	TBC1D9B	1	1	1	1	140.52	NaN	NaN	NaN	NaN	NaN	NaN	NaN	NaN
O75347	Tubulin-specific chaperone A	TBCA	1	2	2	16.7	12.855	5.8E+07	4.9E+07	6E+07	4.4E+07	4.1E+07	6.2E+07	0.88124	0.41184
Q15813	Tubulin-specific chaperone E	TBCE	1	2	2	4	59.345	1.3E+07	2.1E+07	2.6E+07	NaN	3.4E+07	NaN	1.69163	NaN
P23193	Transcription elongation factor A protein 1	TCEA1	1	2	2	8	33.969	4E+07	5.6E+07	4.2E+07	4.6E+07	3.8E+07	4.2E+07	0.91572	0.52164
Q15369	Transcription elongation factor B polypeptide 1	TCEB1	1	2	2	26.8	12.473	NaN	NaN	NaN	NaN	NaN	NaN	NaN	NaN
Q15370	Transcription elongation factor B polypeptide 2	TCEB2	1	2	2	16.1	13.133	NaN	NaN	NaN	NaN	NaN	NaN	NaN	NaN
P17987	T-complex protein 1 subunit alpha	TCP1	1	5	5	9.9	60.343	4.9E+07	4.8E+07	5E+07	4.4E+07	4.8E+07	6.3E+07	1.04826	0.69272
Q9Y2W6	Tudor and KH domain-containing protein	TDRKH	1	1	1	3.2	62.045	NaN	NaN	NaN	NaN	NaN	NaN	NaN	NaN
Q9NZ01	Very-long-chain enoyl-CoA reductase	TECR	1	4	4	11.4	36.034	8.8E+07	9.3E+07	9.7E+07	1.1E+08	9.1E+07	1E+08	1.09811	0.2448
Q9UGI8	Testin	TES	1	2	2	3.3	47.996	NaN	NaN	NaN	NaN	NaN	NaN	NaN	NaN
Q96BS2	Calcineurin B homologous protein 3	TESC	1	2	2	11.7	24.75	NaN	NaN	NaN	NaN	NaN	NaN	NaN	NaN
Q7Z6R9	Transcription factor AP-2-delta	TFAP2D	1	1	1	1.5	49.577	1.5E+08	5.3E+07	6.6E+07	1.3E+08	2.5E+08	1.6E+08	1.99239	0.13278
Q92734	Protein TFG	TFG	1	5	5	12	43.447	1.4E+08	1.5E+08	1.4E+08	1.3E+08	1.6E+08	1.3E+08	0.98512	0.84183
P02786	Transferrin receptor protein 1;Transferrin receptor protein 1, se	TFRC	1	3	3	4.7	84.87	6.5E+07	NaN	NaN	9E+07	NaN	NaN	1.3857	NaN
O43294	Transforming growth factor beta-1-induced transcript 1 protei	TGFB1I1	1	1	1	2	49.814	NaN	NaN	NaN	NaN	NaN	NaN	NaN	NaN
Q15582	Transforming growth factor-beta-induced protein ig-h3	TGFBI	1	3	3	5.9	74.68	1.9E+07	1.5E+07	NaN	1.9E+07	1.7E+07	1.7E+07	1.05654	0.61186
P21980	Protein-glutamine gamma-glutamyltransferase 2	TGM2	2	13	13	16.7	77.328	7.1E+08	7.2E+08	8E+08	6.5E+08	6.8E+08	7.3E+08	0.92715	0.20353
O43493	Trans-Golgi network integral membrane protein 2	TGOLN2	1	2	2	6.2	45.879	NaN	NaN	NaN	NaN	NaN	NaN	NaN	NaN
Q9BV44	THUMP domain-containing protein 3	THUMPD3	1	1	1	2.6	57.002	NaN	NaN	NaN	NaN	NaN	NaN	NaN	NaN
Q9P016	Thymocyte nuclear protein 1	THYN1	1	1	1	4.9	25.697	NaN	NaN	NaN	NaN	NaN	NaN	NaN	NaN
Q01085;P31483	Nucleolysin TIAR;Nucleolysin TIA-1 isoform p40	TIAL1	2	1	1	2.9	41.59	NaN	NaN	NaN	NaN	NaN	NaN	NaN	NaN
Q9NQ88	Fructose-2,6-bisphosphatase TIGAR	TIGAR	1	1	1	6.7	30.062	4.5E+07	NaN	5.1E+07	NaN	4.2E+07	NaN	0.87267	NaN
Q9Y5L4	Mitochondrial import inner membrane translocase subunit Tim	TIMM13	1	2	2	32.6	10.5	NaN	NaN	NaN	NaN	NaN	NaN	NaN	NaN
O60830	Mitochondrial import inner membrane translocase subunit Tim	TIMM17B	1	1	1	8.7	18.273	NaN	NaN	NaN	NaN	NaN	NaN	NaN	NaN
O43615	Mitochondrial import inner membrane translocase subunit TIM	TIMM44	1	1	1	3.1	51.355	NaN	NaN	NaN	NaN	NaN	NaN	NaN	NaN
Q3ZCQ8	Mitochondrial import inner membrane translocase subunit TIM	TIMM50	1	2	2	8.2	39.646	4.1E+07	6.4E+07	4.8E+07	5.6E+07	4.9E+07	4.5E+07	0.97838	0.88941
Q9Y5J7	Mitochondrial import inner membrane translocase subunit Tim	TIMM9	1	1	1	18	10.378	NaN	NaN	NaN	NaN	NaN	NaN	NaN	NaN
Q07157	Tight junction protein ZO-1	TJP1	1	2	2	1.5	195.46	NaN	NaN	NaN	NaN	NaN	NaN	NaN	NaN
P29401	Transketolase	TKT	1	15	15	19.3	67.877	1.7E+09	1.8E+09	1.6E+09	1.9E+09	1.6E+09	1.7E+09	1.00443	0.95129
Q9Y490	Talin-1	TLN1	1	23	21	9.8	269.76	1.7E+08	1.9E+08	1.7E+08	1.9E+08	1.7E+08	1.9E+08	1.03414	0.55332
Q9Y4G6	Talin-2	TLN2	1	4	2	1.5	271.61	NaN	NaN	NaN	NaN	NaN	NaN	NaN	NaN
P30408	Transmembrane 4 L6 family member 1	TM4SF1	1	1	1	4.5	21.632	NaN	NaN	NaN	NaN	NaN	NaN	NaN	NaN
O15321	Transmembrane 9 superfamily member 1	TM9SF1	1	2	2	3.5	68.86	NaN	7884800	NaN	NaN	NaN	9620800	1.22017	NaN
Q9HD45	Transmembrane 9 superfamily member 3	TM9SF3	1	2	2	5.4	67.887	7.6E+07	9.7E+07	8.4E+07	7.8E+07	7.6E+07	NaN	0.89572	0.34973
Q9UM00	Transmembrane and coiled-coil domain-containing protein 1	TMCO1	1	4	4	13.4	27.079	NaN	2.7E+07	NaN	1.7E+07	NaN	NaN	0.63861	NaN
Q15363	Transmembrane emp24 domain-containing protein 2	TMED2	1	1	1	4.5	22.761	NaN	NaN	NaN	NaN	NaN	NaN	NaN	NaN
Q7Z7H5	Transmembrane emp24 domain-containing protein 4	TMED4	1	1	1	4.8	25.943	NaN	NaN	NaN	NaN	NaN	NaN	NaN	NaN
Q9Y3A6	Transmembrane emp24 domain-containing protein 5	TMED5	1	2	2	10	26.005	5.3E+07	NaN	4.8E+07	5.7E+07	6.8E+07	4.8E+07	1.14537	0.40402
Q9BVK6	Transmembrane emp24 domain-containing protein 9	TMED9	1	3	3	8.5	27.277	1.8E+08	2.2E+08	NaN	1.4E+08	1.8E+08	2E+08	0.87701	0.40643
Q9NUM4	Transmembrane protein 106B	TMEM106E	1	2	2	9.5	31.127	NaN	NaN	NaN	NaN	NaN	NaN	NaN	NaN
Q9BVC6	Transmembrane protein 109	TMEM109	1	1	1	4.9	26.21	NaN	NaN	NaN	NaN	NaN	NaN	NaN	NaN
Q24JP5	Transmembrane protein 132A	TMEM132/	1	1	1	1.2	110.11	NaN	NaN	NaN	NaN	NaN	NaN	NaN	NaN
Q9NX00	Transmembrane protein 160	TMEM160	1	1	1	6.9	19.657	NaN	NaN	NaN	NaN	NaN	NaN	NaN	NaN
Q9NX61	Transmembrane protein 161A	TMEM161/	1	1	1	2.5	53.601	NaN	NaN	NaN	NaN	NaN	NaN	NaN	NaN
Q5U3C3	Transmembrane protein 164	TMEM164	1	1	1	6.1	33.507	NaN	NaN	NaN	NaN	NaN	NaN	NaN	NaN
Q9HC07	Transmembrane protein 165	TMEM165	1	2	2	11.4	34.905	1.1E+07	NaN	9860100	NaN	NaN	NaN	NaN	NaN
Q7Z7N9	Transmembrane protein 179B	TMEM179E	1	1	1	7.3	23.55	NaN	NaN	NaN	NaN	NaN	NaN	NaN	NaN
Q9UHN6	Transmembrane protein 2	TMEM2	1	2	2	2	154.37	9058200	NaN	NaN	NaN	1E+07	1.1E+07	1.15917	NaN
Q6UW68	Transmembrane protein 205	TMEM205	1	2	2	15.3	21.198	1.2E+08	1.4E+08	1.2E+08	1.7E+08	1.2E+08	1.2E+08	1.07054	0.58614
Q9NV96	Cell cycle control protein 50A	TMEM30A	1	2	2	8	40.683	NaN	NaN	NaN	NaN	NaN	NaN	NaN	NaN
Q9BTV4	Transmembrane protein 43	TMEM43	1	3	3	8.8	44.875	5.7E+07	2.5E+07	5.4E+07	4.4E+07	5.7E+07	5E+07	1.1078	0.6785
Q5BJF2	Transmembrane protein 97	TMEM97	1	2	2	13.1	20.848	2.2E+07	2.6E+07	1.7E+07	2E+07	NaN	2.3E+07	0.99626	0.98403
P42166;P42167	Lamina-associated polypeptide 2, isoform alpha;Thymopoietin	TMPO	2	4	4	6.8	75.491	NaN	NaN	NaN	NaN	NaN	NaN	NaN	NaN
P62328	Thymosin beta-4;Hematopoietic system regulatory peptide	TMSB4X	1	2	2	31.8	5.0526	4.8E+08	2.6E+08	4.5E+08	3.9E+08	3.3E+08	4E+08	0.94511	0.78147
Q6ZXV5	Transmembrane and TPR repeat-containing protein 3	TMTC3	1	4	4	4.5	104.01	1.7E+07	1.7E+07	1.9E+07	2.3E+07	NaN	1.8E+07	1.15463	0.27573

Q9H3N1	Thioredoxin-related transmembrane protein 1	TMX1	1	2	2	7.1	31.791	4.2E+07	4E+07	NaN	NaN	4E+07	NaN	0.97694	NaN
Q96JJ7	Protein disulfide-isomerase TMX3	TMX3	1	1	1	2.4	51.871	NaN	NaN	NaN	NaN	NaN	NaN	NaN	NaN
Q03169	Tumor necrosis factor alpha-induced protein 2	TNFAIP2	1	2	2	3.4	72.66	NaN	NaN	NaN	NaN	NaN	NaN	NaN	NaN
O14763	Tumor necrosis factor receptor superfamily member 10B	TNFRSF10I	1	1	1	3.4	47.878	NaN	NaN	NaN	NaN	NaN	NaN	NaN	NaN
P41273	Tumor necrosis factor ligand superfamily member 9	TNFSF9	1	1	1	6.7	26.624	NaN	NaN	NaN	NaN	NaN	NaN	NaN	NaN
Q92973	Transportin-1	TNPO1	1	8	8	10.9	102.35	7.5E+07	6.9E+07	8E+07	8.3E+07	7.3E+07	7.3E+07	1.01923	0.76696
Q9Y5L0	Transportin-3	TNPO3	1	3	3	5.5	104.2	2.3E+07	2.6E+07	1.7E+07	2.7E+07	3E+07	1.1E+07	1.01425	0.96324
Q68CZ2	Tensin-3	TNS3	1	3	3	2.9	155.26	1.9E+07	1.9E+07	NaN	NaN	NaN	NaN	NaN	NaN
Q8N4H5	Mitochondrial import receptor subunit TOM5 homolog	TOMM5	1	2	2	19.6	6.0352	NaN	2.8E+07	4E+07	NaN	NaN	3.5E+07	1.03541	NaN
Q96B49	Mitochondrial import receptor subunit TOM6 homolog	TOMM6	1	1	1	18.9	8.0019	NaN	NaN	NaN	NaN	NaN	NaN	NaN	NaN
P11387	DNA topoisomerase 1	TOP1	1	2	2	3.7	90.725	NaN	NaN	NaN	NaN	NaN	NaN	NaN	NaN
Q8NFQ8	Torsin-1A-interacting protein 2	TOR1AIP2	1	2	1	4.9	51.263	NaN	NaN	NaN	NaN	NaN	NaN	NaN	NaN
Q9NXH8	Torsin-4A	TOR4A	1	1	1	3.8	46.914	NaN	NaN	NaN	NaN	NaN	NaN	NaN	NaN
Q53FA7	Quinone oxidoreductase PIG3	TP53I3	1	5	5	17.5	35.536	5.4E+07	8.3E+07	6.2E+07	7.1E+07	7.7E+07	6.3E+07	1.06238	0.68262
Q13641	Trophoblast glycoprotein	TPBG	1	1	1	2.4	46.031	NaN	NaN	NaN	NaN	NaN	NaN	NaN	NaN
O43399	Tumor protein D54	TPD52L2	1	1	1	4.4	22.237	NaN	NaN	NaN	NaN	NaN	NaN	NaN	NaN
P60174	Triosephosphate isomerase	TPI1	1	9	9	39.4	26.669	4.9E+08	4.8E+08	4.6E+08	5.9E+08	5.3E+08	5.6E+08	1.17718	0.01464
P09493	Tropomyosin alpha-1 chain	TPM1	1	6	1	19	32.708	3E+08	2.9E+08	2.4E+08	3E+08	3.3E+08	3.4E+08	1.17255	0.08666
P06753	Tropomyosin alpha-3 chain	TPM3	1	5	1	15.1	32.95	NaN	NaN	NaN	NaN	NaN	NaN	NaN	NaN
P67936	Tropomyosin alpha-4 chain	TPM4	1	1	1	5.2	28.521	NaN	NaN	NaN	NaN	NaN	NaN	NaN	NaN
P29144	Tripeptidyl-peptidase 2	TPP2	1	3	3	2.6	138.35	2.4E+07	NaN	2.4E+07	1.6E+07	2.2E+07	2.3E+07	0.84704	0.26368
P12270	Nucleoprotein TPR	TPR	1	3	3	1.3	267.29	NaN	NaN	NaN	NaN	NaN	NaN	NaN	NaN
P13693	Translationally-controlled tumor protein	TPT1	2	3	3	13.4	19.595	4.6E+08	4E+08	4.7E+08	5E+08	4.1E+08	3.9E+08	0.97136	0.76642
Q15628	Tumor necrosis factor receptor type 1-associated DEATH dom	TRADD	1	1	1	4.5	34.247	8191000	9740300	8345300	1.2E+07	NaN	9138300	1.19635	0.24241
Q12931	Heat shock protein 75 kDa, mitochondrial	TRAP1	1	3	3	4	80.109	3.5E+07	1.6E+07	3.6E+07	3.3E+07	3.5E+07	3.2E+07	1.13497	0.58204
Q9Y5R8	Trafficking protein particle complex subunit 1	TRAPPC1	1	1	1	8.3	16.831	NaN	NaN	NaN	NaN	NaN	NaN	NaN	NaN
Q96F44	E3 ubiquitin-protein ligase TRIM11	TRIM11	1	1	1	2.4	52.774	NaN	NaN	NaN	NaN	NaN	NaN	NaN	NaN
Q309B1;O95361	Tripartite motif-containing protein 16-like protein;Tripartite mo	TRIM16L	2	1	1	4.9	40.305	NaN	NaN	NaN	NaN	NaN	NaN	NaN	NaN
Q13263	Transcription intermediary factor 1-beta	TRIM28	1	2	2	3.6	88.549	8.9E+07	8.1E+07	NaN	5.5E+07	6E+07	4.8E+07	0.64319	0.0103
Q96LD4	Tripartite motif-containing protein 47	TRIM47	1	1	1	2.4	69.531	NaN	NaN	NaN	NaN	NaN	NaN	NaN	NaN
Q9BRZ2	E3 ubiquitin-protein ligase TRIM56	TRIM56	1	1	1	1.9	81.487	NaN	NaN	NaN	NaN	NaN	NaN	NaN	NaN
Q15642	Cdc42-interacting protein 4	TRIP10	1	1	1	2.5	68.351	NaN	NaN	NaN	NaN	NaN	NaN	NaN	NaN
Q7L0Y3	Mitochondrial ribonuclease P protein 1	TRMT10C	1	1	1	3	47.346	NaN	NaN	NaN	NaN	NaN	NaN	NaN	NaN
Q9UI30	Multifunctional methyltransferase subunit TRM112-like protein	TRMT112	1	1	1	11.2	14.199	NaN	NaN	NaN	NaN	NaN	NaN	NaN	NaN
Q32P41	tRNA (guanine(37)-N1)-methyltransferase	TRMT5	1	1	1	2.9	58.256	NaN	NaN	NaN	NaN	NaN	NaN	NaN	NaN
Q96Q11	CCA tRNA nucleotidyltransferase 1, mitochondrial	TRNT1	1	1	1	4.8	50.127	NaN	NaN	NaN	NaN	NaN	NaN	NaN	NaN
P10155	60 kDa SS-A/Ro ribonucleoprotein	TROVE2	1	3	3	5.4	60.67	NaN	NaN	NaN	1.1E+08	3.9E+07	6.1E+07	NaN	NaN
Q8WWH5	Probable tRNA pseudouridine synthase 1	TRUB1	1	2	2	6.3	37.252	NaN	NaN	NaN	NaN	NaN	NaN	NaN	NaN
Q15631	Translin	TSN	1	1	1	3.5	26.183	NaN	NaN	NaN	NaN	NaN	NaN	NaN	NaN
Q99598	Translin-associated protein X	TSNAX	1	2	2	9	33.112	3E+07	3.2E+07	3.2E+07	NaN	NaN	1.9E+07	0.60055	NaN
O95858	Tetraspanin-15	TSPAN15	1	2	2	4.4	33.165	NaN	NaN	NaN	NaN	NaN	NaN	NaN	NaN
Q2NL82	Pre-rRNA-processing protein TSR1 homolog	TSR1	1	3	3	4.5	91.809	2.7E+07	2.6E+07	2.6E+07	2.4E+07	2.5E+07	2.5E+07	0.92759	0.01402
Q16762	Thiosulfate sulfurtransferase	TST	1	2	2	12.5	33.429	3.4E+07	NaN	3.4E+07	2.4E+07	3.4E+07	3E+07	0.85073	0.24153
Q99614	Tetratricopeptide repeat protein 1	TTC1	1	4	4	17.1	33.526	2.1E+07	2.8E+07	NaN	2.1E+07	2.4E+07	NaN	0.92442	0.64792
Q6P3X3	Tetratricopeptide repeat protein 27	TTC27	1	1	1	1.3	96.631	NaN	NaN	NaN	NaN	NaN	NaN	NaN	NaN
Q6PGP7	Tetratricopeptide repeat protein 37	TTC37	1	1	1	1	175.48	NaN	NaN	NaN	NaN	NaN	NaN	NaN	NaN
Q14166	Tubulin--tyrosine ligase-like protein 12	TTLL12	1	3	3	5.6	74.403	NaN	NaN	NaN	NaN	NaN	NaN	NaN	NaN
Q9BQE3;Q71U36;P6836	Tubulin alpha-1C chain;Tubulin alpha-1A chain;Tubulin alpha-	TUBA1C	8	5	1	12.7	49.895	3.2E+09	3.6E+09	3.6E+09	5.3E+09	3.7E+09	2.7E+09	1.1287	0.59407
P68366	Tubulin alpha-4A chain	TUBA4A	1	5	1	12.7	49.924	NaN	NaN	NaN	NaN	NaN	NaN	NaN	NaN
P07437;Q9BVA1;Q1388	Tubulin beta chain;Tubulin beta-2B chain;Tubulin beta-2A chai	TUBB	4	4	1	11.7	49.67	NaN	NaN	NaN	NaN	NaN	NaN	NaN	NaN
P04350;Q3ZCM7;A6NN	Tubulin beta-4A chain;Tubulin beta-8 chain;Tubulin beta-8 ch	TUBB4A	5	4	0	11.7	49.585	NaN	NaN	NaN	NaN	NaN	NaN	NaN	NaN
P68371	Tubulin beta-4B chain	TUBB4B	1	4	0	11.7	49.83	2.6E+08	2.8E+08	2.8E+08	2.4E+08	2.8E+08	3.2E+08	1.01811	0.82398
Q9BUF5	Tubulin beta-6 chain	TUBB6	1	3	1	9.6	49.857	NaN	NaN	NaN	NaN	NaN	NaN	NaN	NaN
Q9NRH3;P23258	Tubulin gamma-2 chain;Tubulin gamma-1 chain	TUBG2	2	1	1	2.4	51.091	NaN	1.8E+07	1.5E+07	NaN	NaN	1.9E+07	1.14481	NaN
Q9BSJ2	Gamma-tubulin complex component 2	TUBGCP2	1	1	1	1.3	102.53	NaN	NaN	NaN	NaN	NaN	NaN	NaN	NaN
P49411	Elongation factor Tu, mitochondrial	TUFM	1	2	2	5.3	49.874	NaN	8.3E+07	2.2E+07	NaN	NaN	3.1E+07	0.58026	NaN
Q6IBS0	Twinfilin-2	TWF2	1	1	1	5.4	39.548	7.4E+07	8.2E+07	6.9E+07	6.1E+07	8E+07	9.3E+07	1.04197	0.76428
P40222	Alpha-taxilin	TXLNA	1	1	1	1.6	61.89	NaN	NaN	NaN	NaN	NaN	NaN	NaN	NaN
P10599	Thioredoxin	TXN	1	1	1	12.4	11.737	NaN	NaN	NaN	NaN	NaN	NaN	NaN	NaN

O95881	Thioredoxin domain-containing protein 12	TXNDC12	1	2	2	14	19.206	NaN	1.2E+07	9452900	1.3E+07	1.2E+07	1.1E+07	1.1169	0.36357
Q9BRA2	Thioredoxin domain-containing protein 17	TXNDC17	1	1	1	7.3	13.941	NaN	NaN	NaN	NaN	NaN	NaN	NaN	NaN
Q8NBS9	Thioredoxin domain-containing protein 5	TXNDC5	1	1	1	1.6	47.628	NaN	NaN	NaN	NaN	NaN	NaN	NaN	NaN
O43396	Thioredoxin-like protein 1	TXNL1	1	2	2	13.5	32.251	NaN	2E+07	1.3E+07	3.3E+07	NaN	NaN	1.95513	NaN
Q16881	Thioredoxin reductase 1, cytoplasmic	TXNRD1	1	7	7	13.3	70.905	4.1E+08	4.1E+08	5.8E+08	5.3E+08	5.8E+08	5.4E+08	1.1866	0.21627
P19971	Thymidine phosphorylase	TYMP	1	4	4	8.3	49.955	2.3E+07	2.1E+07	2.1E+07	2.6E+07	2.4E+07	2.6E+07	1.17386	0.01264
Q6NUM6;Q9NV66	S-adenosyl-L-methionine-dependent tRNA 4-demethylwyosi	TYW1B	2	1	1	1.6	76.946	NaN	NaN	NaN	NaN	NaN	NaN	NaN	NaN
P26368	Splicing factor U2AF 65 kDa subunit	U2AF2	1	2	2	5.7	53.5	2.3E+07	1.6E+07	NaN	2.6E+07	1.8E+07	2.9E+07	1.26262	0.39229
P22314	Ubiquitin-like modifier-activating enzyme 1	UBA1	1	8	8	7.3	117.85	3.3E+08	3.2E+08	2.8E+08	2.7E+08	2.8E+08	2.7E+08	0.89313	0.07733
Q9UBT2	SUMO-activating enzyme subunit 2	UBA2	1	6	6	12	71.223	1.4E+08	1.1E+08	1.4E+08	1.3E+08	1.2E+08	1.4E+08	0.9999	0.99923
Q8TBC4	NEDD8-activating enzyme E1 catalytic subunit	UBA3	1	1	1	3.5	51.852	NaN	NaN	NaN	NaN	NaN	NaN	NaN	NaN
P62987;P62979;P0CG47	Ubiquitin-60S ribosomal protein L40;Ubiquitin;60S ribosomal p	UBA52	4	6	6	44.5	14.728	1.8E+09	1.9E+09	1.8E+09	2.2E+09	1.7E+09	1.6E+09	1.00681	0.94262
A0AVT1	Ubiquitin-like modifier-activating enzyme 6	UBA6	1	2	2	1.9	117.97	NaN	NaN	NaN	NaN	NaN	NaN	NaN	NaN
P51965	Ubiquitin-conjugating enzyme E2 E1	UBE2E1	1	1	1	9.3	21.404	NaN	NaN	NaN	NaN	NaN	NaN	NaN	NaN
Q96LR5;Q969T4	Ubiquitin-conjugating enzyme E2 E2;Ubiquitin-conjugating en	UBE2E2	2	1	1	9	22.255	NaN	NaN	NaN	NaN	NaN	NaN	NaN	NaN
P63279	SUMO-conjugating enzyme UBC9	UBE2I	1	1	1	6.3	18.007	NaN	NaN	NaN	NaN	NaN	NaN	NaN	NaN
P61086	Ubiquitin-conjugating enzyme E2 K	UBE2K	1	3	3	19	22.406	4.4E+07	5.3E+07	5.8E+07	4.8E+07	5.3E+07	4.6E+07	0.94741	0.59028
P61081	NEDD8-conjugating enzyme Ubc12	UBE2M	1	3	3	16.9	20.9	9E+07	6.2E+07	1.3E+08	8.2E+07	1.7E+08	1.5E+08	1.4137	0.30373
P61088;Q5JXB2	Ubiquitin-conjugating enzyme E2 N;Putative ubiquitin-conjuga	UBE2N	2	2	2	11.2	17.138	4.6E+07	7.8E+07	7.5E+07	6.4E+07	7.5E+07	5.6E+07	0.98296	0.92786
Q9C0C9	E2/E3 hybrid ubiquitin-protein ligase UBE2O	UBE2O	1	4	4	4.8	141.29	1.1E+07	1E+07	9845300	1E+07	NaN	NaN	1.02654	NaN
Q16763	Ubiquitin-conjugating enzyme E2 S	UBE2S	1	2	2	12.2	23.845	NaN	NaN	NaN	NaN	NaN	NaN	NaN	NaN
Q13404	Ubiquitin-conjugating enzyme E2 variant 1	UBE2V1	1	2	1	12.9	16.495	NaN	NaN	NaN	NaN	NaN	NaN	NaN	NaN
Q15819	Ubiquitin-conjugating enzyme E2 variant 2	UBE2V2	1	3	2	21.4	16.363	NaN	2.8E+07	NaN	2.9E+07	NaN	2.7E+07	1.00088	NaN
Q15386	Ubiquitin-protein ligase E3C	UBE3C	1	2	2	2.1	123.92	NaN	NaN	NaN	NaN	NaN	NaN	NaN	NaN
Q7Z6J8	E3 ubiquitin-protein ligase E3D	UBE3D	1	1	1	3.9	43.656	NaN	NaN	NaN	NaN	NaN	NaN	NaN	NaN
O95155	Ubiquitin conjugation factor E4 B	UBE4B	1	2	2	1.8	146.18	1.1E+07	NaN	1.2E+07	NaN	NaN	NaN	NaN	NaN
O14562	Ubiquitin domain-containing protein UBFD1	UBFD1	1	2	2	7.1	33.382	NaN	NaN	NaN	NaN	NaN	NaN	NaN	NaN
P11441	Ubiquitin-like protein 4A	UBL4A	1	2	2	16.6	17.776	2.2E+07	1.8E+07	NaN	1.4E+07	NaN	9121400	0.59465	0.1328
Q9BZL1	Ubiquitin-like protein 5	UBL5	1	1	1	16.4	8.5468	NaN	NaN	NaN	NaN	NaN	NaN	NaN	NaN
Q8WVY7	Ubiquitin-like domain-containing CTD phosphatase 1	UBLCP1	1	2	2	5.7	36.804	3.6E+07	NaN	3.3E+07	3.4E+07	3E+07	3.5E+07	0.94354	0.41638
Q9UHD9	Ubiquilin-2	UBQLN2	1	2	2	4.8	65.695	NaN	NaN	NaN	NaN	NaN	NaN	NaN	NaN
Q5T4S7	E3 ubiquitin-protein ligase UBR4	UBR4	1	13	13	3.1	573.83	2.2E+07	3.5E+07	2.6E+07	2.3E+07	2.8E+07	1.7E+07	0.8152	0.35879
P17480	Nucleolar transcription factor 1	UBTF	1	2	2	3.8	89.405	NaN	NaN	NaN	NaN	NaN	NaN	NaN	NaN
Q04323	UBX domain-containing protein 1	UBXN1	1	1	1	9.8	33.325	NaN	NaN	NaN	NaN	NaN	NaN	NaN	NaN
P09936	Ubiquitin carboxyl-terminal hydrolase isozyme L1	UCHL1	1	4	4	21.5	24.824	5.8E+08	7.7E+08	7.4E+08	6.6E+08	7.2E+08	7.8E+08	1.03651	0.73409
P15374	Ubiquitin carboxyl-terminal hydrolase isozyme L3	UCHL3	1	2	2	9.6	26.182	1.9E+07	2E+07	1.9E+07	NaN	1.9E+07	1.9E+07	0.99579	0.81422
Q9Y5K5	Ubiquitin carboxyl-terminal hydrolase isozyme L5	UCHL5	1	1	1	5.2	37.606	NaN	NaN	NaN	NaN	NaN	NaN	NaN	NaN
Q9Y3C8	Ubiquitin-fold modifier-conjugating enzyme 1	UFC1	1	1	1	4.8	19.458	NaN	NaN	NaN	NaN	NaN	NaN	NaN	NaN
Q92890	Ubiquitin fusion degradation protein 1 homolog	UFD1L	1	4	4	16.6	34.5	NaN	6.5E+07	NaN	4.8E+07	5.5E+07	3.3E+07	0.69341	NaN
Q9NUQ7	Ufm1-specific protease 2	UFSP2	1	1	1	2.3	53.261	NaN	NaN	NaN	NaN	NaN	NaN	NaN	NaN
O60701	UDP-glucose 6-dehydrogenase	UGDH	1	6	6	14	55.023	2.8E+08	2.7E+08	3E+08	3.3E+08	3.3E+08	3.1E+08	1.13986	0.02271
Q9NYU2	UDP-glucose:glycoprotein glucosyltransferase 1	UGGT1	1	4	4	2.9	177.19	3.7E+07	NaN	4E+07	3E+07	3.3E+07	5.5E+07	1.03107	0.91427
Q16851	UTP--glucose-1-phosphate uridylyltransferase	UGP2	1	6	6	14.4	56.94	5.5E+07	5.3E+07	5.2E+07	6.5E+07	5.6E+07	5.4E+07	1.0935	0.2367
A6NIH7	Protein unc-119 homolog B	UNC119B	1	1	1	7.2	28.136	NaN	NaN	NaN	NaN	NaN	NaN	NaN	NaN
Q9H3U1	Protein unc-45 homolog A	UNC45A	1	1	1	1.1	103.08	NaN	NaN	NaN	NaN	NaN	NaN	NaN	NaN
Q8N2C7	Protein unc-80 homolog	UNC80	1	1	1	0.3	363.39	NaN	NaN	NaN	NaN	NaN	NaN	NaN	NaN
Q9H1C4	Protein unc-93 homolog B1	UNC93B1	1	1	1	2.8	66.63	NaN	NaN	NaN	NaN	NaN	NaN	NaN	NaN
Q92900	Regulator of nonsense transcripts 1	UPF1	1	3	3	3.3	124.34	2E+07	1.9E+07	2E+07	NaN	2.1E+07	1.9E+07	1.02457	0.60269
Q9BRT2	Ubiquinol-cytochrome-c reductase complex assembly factor 2	UQCC2	1	1	1	9.5	14.875	NaN	NaN	NaN	NaN	NaN	NaN	NaN	NaN
Q9UDW1	Cytochrome b-c1 complex subunit 9	UQCR10	1	1	1	27	7.3084	1.6E+07	NaN	1.5E+07	NaN	2E+07	1.9E+07	1.24808	0.00271
P14927	Cytochrome b-c1 complex subunit 7	UQCRB	1	4	4	34.2	13.53	6E+07	6E+07	5E+07	6.3E+07	6E+07	5.7E+07	1.06227	0.39874
P31930	Cytochrome b-c1 complex subunit 1, mitochondrial	UQCRC1	1	3	3	4.2	52.645	NaN	5E+07	4.6E+07	5.3E+07	4.7E+07	4.8E+07	1.03207	0.64287
P22695	Cytochrome b-c1 complex subunit 2, mitochondrial	UQCRC2	1	1	1	3.5	48.442	NaN	NaN	NaN	NaN	NaN	NaN	NaN	NaN
P07919	Cytochrome b-c1 complex subunit 6, mitochondrial	UQCRH	1	2	2	29.7	10.739	2E+07	2.7E+07	NaN	3.6E+07	3.5E+07	2.3E+07	1.32502	0.28378
Q9BTM9	Ubiquitin-related modifier 1	URM1	1	1	1	12.9	11.38	NaN	NaN	NaN	NaN	NaN	NaN	NaN	NaN
P06132	Uroporphyrinogen decarboxylase	UROD	1	2	2	8.2	40.786	NaN	4.5E+07	5.2E+07	NaN	NaN	NaN	NaN	NaN
O60763	General vesicular transport factor p115	USO1	1	2	2	2.2	107.89	NaN	3.3E+07	1.7E+07	NaN	1.4E+07	1.7E+07	0.6094	0.3367
Q14694	Ubiquitin carboxyl-terminal hydrolase 10	USP10	1	1	1	1.5	87.133	NaN	NaN	NaN	NaN	NaN	NaN	NaN	NaN
P51784	Ubiquitin carboxyl-terminal hydrolase 11	USP11	1	1	1	1	109.82	NaN	NaN	NaN	NaN	NaN	NaN	NaN	NaN

P54578	Ubiquitin carboxyl-terminal hydrolase 14	USP14	1	5	5	12.1	56.068	6.3E+07	1E+08	7.3E+07	7.9E+07	9.3E+07	6.4E+07	1.00234	0.99006
Q9Y4E8	Ubiquitin carboxyl-terminal hydrolase 15	USP15	1	1	1	1.4	112.42	NaN	NaN	NaN	NaN	NaN	NaN	NaN	NaN
Q70CQ2	Ubiquitin carboxyl-terminal hydrolase 34	USP34	1	1	1	0.6	404.23	NaN	NaN	NaN	NaN	NaN	NaN	NaN	NaN
Q53GS9	U4/U6.U5 tri-snRNP-associated protein 2	USP39	1	3	3	5.5	65.38	1.8E+07	NaN	1.8E+07	1.9E+07	1.6E+07	NaN	0.98339	0.87787
P45974	Ubiquitin carboxyl-terminal hydrolase 5	USP5	1	4	4	5.4	95.785	9E+07	7.3E+07	7E+07	8.5E+07	7.2E+07	NaN	1.0115	0.92896
Q93009	Ubiquitin carboxyl-terminal hydrolase 7	USP7	1	3	3	3	128.3	2.6E+07	2.9E+07	1.8E+07	2.3E+07	NaN	2.3E+07	0.95249	0.81112
P40818	Ubiquitin carboxyl-terminal hydrolase 8	USP8	1	1	1	1.1	127.52	NaN	NaN	NaN	NaN	NaN	NaN	NaN	NaN
Q93008	Probable ubiquitin carboxyl-terminal hydrolase FAF-X	USP9X	2	11	11	5	290.46	3.5E+07	3E+07	4.4E+07	4.3E+07	4.5E+07	2.7E+07	1.06071	0.76753
P46939	Utrophin	UTRN	1	3	3	1.2	394.46	1.1E+07	NaN	1.1E+07	NaN	NaN	1.1E+07	1.05769	NaN
O95399	Urotensin-2	UTS2	1	1	1	6.5	14.295	NaN	NaN	NaN	NaN	NaN	NaN	NaN	NaN
Q9P0L0	Vesicle-associated membrane protein-associated protein A	VAPA	1	1	1	5.6	27.893	1.3E+08	3.5E+07	1.2E+08	1.1E+08	1.2E+08	8.9E+07	1.08385	0.81211
P26640	Valine--tRNA ligase	VARS	1	8	8	7.8	140.47	7.3E+07	6.2E+07	6.3E+07	7.7E+07	7.1E+07	9E+07	1.21128	0.09956
P50552	Vasodilator-stimulated phosphoprotein	VASP	1	4	4	8.2	39.829	1.2E+08	1.1E+08	1.1E+08	1.5E+08	1.4E+08	1.4E+08	1.23511	0.00366
Q99536	Synaptic vesicle membrane protein VAT-1 homolog	VAT1	1	4	4	10.7	41.92	8.1E+07	1E+08	7E+07	7.9E+07	7.6E+07	6.9E+07	0.88657	0.3866
P61758	Prefoldin subunit 3	VBP1	1	2	2	10.2	22.626	NaN	NaN	NaN	NaN	NaN	NaN	NaN	NaN
P18206	Vinculin	VCL	1	16	16	16.1	123.8	2.2E+08	2.1E+08	2.3E+08	2.4E+08	2E+08	2.2E+08	0.99598	0.94973
P55072	Transitional endoplasmic reticulum ATPase	VCP	1	22	22	24.7	89.321	5E+08	4.8E+08	4.9E+08	4.9E+08	5.1E+08	4.9E+08	1.01055	0.57195
P21796	Voltage-dependent anion-selective channel protein 1	VDAC1	1	5	5	20.8	30.772	1.1E+08	1E+08	9.9E+07	9.6E+07	1.1E+08	1E+08	1.01172	0.80728
P45880	Voltage-dependent anion-selective channel protein 2	VDAC2	1	2	2	7.1	31.566	NaN	3.3E+07	4E+07	NaN	3.3E+07	NaN	0.89706	NaN
Q9HBM0	Vezatin	VEZT	1	2	2	2.8	88.664	NaN	NaN	NaN	NaN	NaN	NaN	NaN	NaN
P08670	Vimentin	VIM	2	8	6	12.9	53.651	7.9E+07	9.3E+07	7.8E+07	5.5E+07	6.1E+07	6.8E+07	0.73494	0.02359
Q96GC9	Vacuole membrane protein 1	VMP1	1	1	1	2.5	46.237	NaN	NaN	NaN	NaN	NaN	NaN	NaN	NaN
Q9P253	Vacuolar protein sorting-associated protein 18 homolog	VPS18	1	1	1	1.1	110.18	NaN	NaN	NaN	NaN	NaN	NaN	NaN	NaN
O75436	Vacuolar protein sorting-associated protein 26A	VPS26A	1	1	1	3.7	38.169	NaN	NaN	NaN	NaN	NaN	NaN	NaN	NaN
Q9UK41	Vacuolar protein sorting-associated protein 28 homolog	VPS28	1	1	1	4.5	25.425	NaN	NaN	NaN	NaN	NaN	NaN	NaN	NaN
Q9UBQ0	Vacuolar protein sorting-associated protein 29	VPS29	1	2	2	12.6	20.505	8E+07	NaN	7.9E+07	NaN	NaN	NaN	NaN	NaN
Q96QK1	Vacuolar protein sorting-associated protein 35	VPS35	1	9	9	11.8	91.706	8.7E+07	1.2E+08	1E+08	7.2E+07	8.4E+07	7.6E+07	0.7557	0.06317
Q96JC1	Vam6/Vps39-like protein	VPS39	1	1	1	1.8	101.81	NaN	NaN	NaN	NaN	NaN	NaN	NaN	NaN
Q9UID3	Vacuolar protein sorting-associated protein 51 homolog	VPS51	1	1	1	1.9	86.041	NaN	NaN	NaN	NaN	NaN	NaN	NaN	NaN
Q5VIR6	Vacuolar protein sorting-associated protein 53 homolog	VPS53	1	1	1	1.4	94.404	NaN	NaN	NaN	NaN	NaN	NaN	NaN	NaN
P23381	Tryptophan--tRNA ligase, cytoplasmic;T1-TrpRS;T2-TrpRS	WARS	1	1	1	3	53.165	NaN	NaN	NaN	NaN	NaN	NaN	NaN	NaN
Q96I51	Williams-Beuren syndrome chromosomal region 16 protein	WBSCR16	1	1	1	1.9	49.897	NaN	NaN	NaN	NaN	NaN	NaN	NaN	NaN
O75083	WD repeat-containing protein 1	WDR1	1	5	5	7.8	66.193	8.1E+07	6.1E+07	7.8E+07	9.5E+07	9.4E+07	1E+08	1.33376	0.02527
Q9BZH6	WD repeat-containing protein 11	WDR11	1	1	1	0.9	136.68	NaN	NaN	NaN	NaN	NaN	NaN	NaN	NaN
Q9BRP8	Partner of Y14 and mago	WIBG	1	4	4	24	22.655	NaN	2.1E+07	NaN	2E+07	1.9E+07	1.9E+07	0.89963	NaN
Q9H4A3	Serine/threonine-protein kinase WNK1	WNK1	1	1	1	0.7	250.79	NaN	NaN	NaN	NaN	NaN	NaN	NaN	NaN
A4UGR9	Xin actin-binding repeat-containing protein 2	XIRP2	1	1	1	0.2	382.3	NaN	NaN	NaN	NaN	NaN	NaN	NaN	NaN
Q9NQW7	Xaa-Pro aminopeptidase 1	XPNPEP1	1	1	1	1.6	69.917	NaN	NaN	NaN	NaN	NaN	NaN	NaN	NaN
O14980	Exportin-1	XPO1	1	6	6	6.6	123.38	8.3E+07	1.1E+08	8.3E+07	1.3E+08	8.7E+07	9.2E+07	1.10792	0.54115
Q9HAV4	Exportin-5	XPO5	1	2	2	2.1	136.31	1.2E+07	1.6E+07	NaN	NaN	NaN	NaN	NaN	NaN
Q9UIA9	Exportin-7	XPO7	1	1	1	0.8	123.91	NaN	NaN	NaN	NaN	NaN	NaN	NaN	NaN
P13010	X-ray repair cross-complementing protein 5	XRCC5	1	5	5	7.8	82.704	2.6E+08	3E+08	2.9E+08	3.1E+08	2.6E+08	2.9E+08	1.02258	0.75516
P12956	X-ray repair cross-complementing protein 6	XRCC6	1	5	5	8.9	69.842	2.2E+08	2E+08	2E+08	2.2E+08	2.1E+08	2.4E+08	1.0592	0.31493
Q8IZH2	5-3 exoribonuclease 1	XRN1	1	1	1	0.8	194.1	NaN	NaN	NaN	NaN	NaN	NaN	NaN	NaN
Q9H0D6	5-3 exoribonuclease 2	XRN2	1	3	3	2.9	108.58	1.9E+07	1.7E+07	1.9E+07	NaN	2.1E+07	2E+07	1.10892	0.11297
P54577	Tyrosine--tRNA ligase, cytoplasmic;Tyrosine--tRNA ligase, cyto	YARS	1	5	5	10.6	59.143	4.6E+07	3.2E+07	3.1E+07	2E+07	3.1E+07	3.1E+07	0.76756	0.23811
P67809;Q9Y2T7	Nuclease-sensitive element-binding protein 1;Y-box-binding p	YBX1	2	4	2	17	35.924	8E+07	9E+07	8.7E+07	5.9E+07	8E+07	7.1E+07	0.82025	0.08774
P16989	Y-box-binding protein 3	YBX3	1	4	2	17.7	40.089	2.3E+07	2E+07	3.8E+07	2.4E+07	3E+07	2.5E+07	0.96672	0.88286
O95070	Protein YIF1A	YIF1A	1	1	1	5.1	32.011	NaN	NaN	NaN	NaN	NaN	NaN	NaN	NaN
P49750	YLP motif-containing protein 1	YLPM1	1	1	1	0.7	241.64	NaN	NaN	NaN	NaN	NaN	NaN	NaN	NaN
Q96TA2	ATP-dependent zinc metalloprotease YME1L1	YME1L1	1	1	1	1.4	86.454	NaN	NaN	NaN	NaN	NaN	NaN	NaN	NaN
Q9BYJ9;Q7Z739	YTH domain-containing family protein 1;YTH domain-containi	YTHDF1	2	1	1	2.3	60.873	NaN	NaN	NaN	NaN	NaN	NaN	NaN	NaN
Q9Y5A9	YTH domain-containing family protein 2	YTHDF2	1	1	1	2.2	62.333	1.9E+07	2E+07	1.5E+07	NaN	NaN	NaN	NaN	NaN
P31946	14-3-3 protein beta/alpha;14-3-3 protein beta/alpha, N-term	YWHAB	1	7	5	32.9	28.082	3.8E+07	4.7E+07	3.7E+07	4.4E+07	4.5E+07	2.9E+07	0.97269	0.86036
P62258	14-3-3 protein epsilon	YWHAE	1	12	11	40	29.174	3.9E+08	3.9E+08	4.3E+08	5.6E+08	4.7E+08	5E+08	1.25918	0.02622
P61981	14-3-3 protein gamma;14-3-3 protein gamma, N-terminally p	YWHAG	1	5	4	21.1	28.302	3.1E+08	2.7E+08	3.7E+08	5.3E+08	3.1E+08	2.2E+08	1.11824	0.71899
Q04917	14-3-3 protein eta	YWHAH	1	4	3	20.7	28.218	2.3E+07	2.6E+07	2.4E+07	2.4E+07	2.2E+07	NaN	0.94088	0.35178
P27348	14-3-3 protein theta	YWHAQ	1	5	3	23.7	27.764	2.7E+08	NaN	3E+08	3.2E+08	3.9E+08	3E+08	1.18355	0.26222
P63104	14-3-3 protein zeta/delta	YWHAZ	1	8	6	36.7	27.745	6.6E+08	6.7E+08	6.6E+08	6.7E+08	6.9E+08	6.7E+08	1.01938	0.14721

Q8WU90	Zinc finger CCCH domain-containing protein 15	ZC3H15	1	4	4	8.2	48.602	NaN	NaN	NaN	NaN	NaN	NaN	NaN	NaN
P61129	Zinc finger CCCH domain-containing protein 6	ZC3H6	1	1	1	0.6	131.67	NaN	NaN	NaN	NaN	NaN	NaN	NaN	NaN
Q8IWR0	Zinc finger CCCH domain-containing protein 7A	ZC3H7A	1	1	1	1.1	110.54	NaN	NaN	NaN	NaN	NaN	NaN	NaN	NaN
O75844	CAAX prenyl protease 1 homolog	ZMPSTE24	1	2	2	5.9	54.812	NaN	NaN	NaN	NaN	NaN	NaN	NaN	NaN
O43670	BUB3-interacting and GLEBS motif-containing protein ZNF207	ZNF207	1	2	2	5.4	50.75	NaN	NaN	1.6E+07	NaN	9003100	1.5E+07	0.74951	NaN
Q8IWY8	Zinc finger and SCAN domain-containing protein 29	ZSCAN29	1	1	1	1.2	96.718	NaN	NaN	NaN	NaN	NaN	NaN	NaN	NaN
O43264	Centromere/kinetochore protein zw10 homolog	ZW10	1	2	2	3.1	88.828	NaN	1.6E+08	1.8E+08	1.6E+08	1.5E+08	1.6E+08	0.92787	0.20618
Q15942	Zyxin	ZYX	1	1	1	3.3	61.277	NaN	NaN	NaN	NaN	NaN	NaN	NaN	NaN
A0A0U1RRL7	Protein MMP24OS	MMP24OS	1	1	1	36.6	7.6794	NaN	NaN	1.5E+07	1.5E+07	NaN	1.5E+07	0.96265	NaN
P0DP25;P0DP24;P0DP2	Calmodulin-3	CALM3	3	3	3	18.8	16.837	5.1E+08	3.4E+08	NaN	8.3E+08	6.3E+08	6.1E+08	1.62192	0.0904

Table S3. A full list of human proteins identified by biotin-tagged apigenin (Bio-APIG)

Majority protein IDs	Protein names	Gene names	Number of proteins	Peptides	Unique peptides	Sequence coverage [%]	Mol. weight [kDa]	LFQ intensity_Biotin	LFQ intensity_Bio-APIG	LFQ intensity_APIG 10 fold	LFQ intensity_APIG 20 fold	Ratio_Bio-APIG/biotin	Ratio_APIG 10 flod/Bio-APIG	Ratio_APIG 20 flod/Bio-APIG
REV__Q86\	Toll-like receptor 5	TLR5	2	2	2	0.8	92.032	2921200	820300000	2921200	2921200	280.80926	0.0035611	0.0035611
P00966	Argininosuccinate synthase	ASS1	1	2	2	2.9	46.53	2921200	585740000	2921200	2921200	200.51349	0.0049872	0.0049872
Q96AB3	Isochorismatase domain-containing protein 2, mitochon	ISOC2	1	2	2	9.8	22.337	2921200	442720000	2921200	2921200	151.55416	0.0065983	0.0065983
Q8IWZ3	Ankyrin repeat and KH domain-containing protein 1	ANKHD1	1	2	2	0.6	269.45	2921200	388640000	2921200	2921200	133.04122	0.0075165	0.0075165
P06703	Protein S100-A6	S100A6	1	2	2	16.7	10.18	2921200	313080000	2921200	2921200	107.17513	0.0093305	0.0093305
Q9NWB6	Arginine and glutamate-rich protein 1	ARGLU1	1	2	2	5.9	33.216	2921200	82956000	2921200	2921200	28.397919	0.0352138	0.0352138
P00338	L-lactate dehydrogenase A chain	LDHA	3	3	2	9.3	36.688	2921200	69698000	2921200	2921200	23.859373	0.0419122	0.0419122
P30041	**Peroxiredoxin-6**	**PRDX6**	1	3	3	13.4	25.035	2921200	65942000	2921200	2921200	22.5736	0.0442995	0.0442995
P21980	Protein-glutamine gamma-glutamyltransferase 2	TGM2	1	3	3	4.8	77.328	2921200	59641000	2921200	2921200	20.41661	0.0489797	0.0489797
P62861	40S ribosomal protein S30	FAU	1	2	2	8.3	14.39	2921200	52905000	2921200	2921200	18.110708	0.055216	0.055216
O95340	Bifunctional 3-phosphoadenosine 5-phosphosulfate syn	PAPSS2	1	2	2	4.6	69.5	2921200	50721000	2921200	2921200	17.36307	0.0575935	0.0575935
P11413	Glucose-6-phosphate 1-dehydrogenase	G6PD	1	3	3	6.6	59.256	2921200	50514000	2921200	2921200	17.292209	0.0578295	0.0578295
A0A8I5KQI	40S ribosomal protein SA	RPSA	2	3	3	13.9	32.909	2921200	44237000	2921200	2921200	15.143434	0.0660352	0.0660352
Q86X76	Nitrilase homolog 1	NIT1	1	2	2	5.2	35.896	2921200	44195000	2921200	2921200	15.129057	0.066098	0.066098
P62258	**14-3-3 protein epsilon**	**YWHAE**	1	2	2	9	29.174	2921200	42726000	2921200	2921200	14.626181	0.0683705	0.0683705
P06576	ATP synthase subunit beta, mitochondrial	ATP5B	1	2	2	6.2	56.559	2921200	41048000	2921200	2921200	14.05176	0.0711655	0.0711655
Q5GLZ8	Probable E3 ubiquitin-protein ligase HERC4	HERC4	1	2	2	2.9	118.56	2921200	40931000	2921200	2921200	14.011708	0.0713689	0.0713689
P31942	Heterogeneous nuclear ribonucleoprotein H3	HNRNPH3	1	2	2	7.2	36.926	2921200	38572000	2921200	2921200	13.204163	0.0757337	0.0757337
P08708	40S ribosomal protein S17	RPS17	1	2	2	14.1	15.55	2921200	37271000	2921200	2921200	12.758798	0.0783773	0.0783773
P62277	40S ribosomal protein S13	RPS13	1	2	2	15.9	17.222	2921200	35417000	2921200	2921200	12.124127	0.0824802	0.0824802
P61978	Heterogeneous nuclear ribonucleoprotein K	HNRNPK	1	5	5	15.6	50.976	2921200	33240000	2921200	2921200	11.378885	0.0878821	0.0878821
Q9NPJ3	Acyl-coenzyme A thioesterase 13;Acyl-coenzyme A thio	ACOT13	1	2	2	11.4	14.96	2921200	32111000	2921200	2921200	10.9924	0.0909719	0.0909719
Q5JRX3	Presequence protease, mitochondrial	PITRM1	1	2	2	2.6	117.41	2921200	29606000	2921200	2921200	10.134876	0.0986692	0.0986692
P12109	Collagen alpha-1(VI) chain	COL6A1	1	2	2	2.9	108.53	2921200	29590000	2921200	2921200	10.129399	0.0987225	0.0987225
Q92499	ATP-dependent RNA helicase DDX1	DDX1	1	2	2	2.8	82.431	2921200	28263000	14647000	2921200	9.6751335	0.5182394	0.1033577
P49368	T-complex protein 1 subunit gamma	CCT3	1	3	3	6.4	60.533	2921200	27457000	2921200	2921200	9.3992195	0.1063918	0.1063918
P31944	Caspase-14;Caspase-14 subunit p17, mature form;Caspa	CASP14	1	2	2	9.1	27.679	2921200	26241000	2921200	2921200	8.9829522	0.111322	0.111322
Q14764	Major vault protein	MVP	1	3	3	6.4	99.326	2921200	25807000	2921200	2921200	8.8343831	0.1131941	0.1131941
P12111	Collagen alpha-3(VI) chain	COL6A3	1	5	5	2	343.67	2921200	24397000	2921200	2921200	8.3517048	0.119736	0.119736
Q15084	Protein disulfide-isomerase A6	PDIA6	1	2	2	6.8	48.121	2921200	23969000	2921200	14254000	8.2051896	0.1218741	0.5946848
P78347	General transcription factor II-I	GTF2I	1	3	3	3.6	112.42	2921200	23877000	2921200	2921200	8.1736957	0.1223437	0.1223437
P29692	Elongation factor 1-delta	EEF1D	1	3	3	16	31.121	2921200	23838000	2921200	2921200	8.1603451	0.1225438	0.1225438
P45880	Voltage-dependent anion-selective channel protein 2	VDAC2	1	2	2	10.9	31.566	2921200	23269000	2921200	2921200	7.9655621	0.1255404	0.1255404
Q14697	Neutral alpha-glucosidase AB	GANAB	1	3	3	4.1	106.87	2921200	18241000	2921200	2921200	6.2443516	0.1601447	0.1601447
Q7KZF4	Staphylococcal nuclease domain-containing protein 1	SND1	1	2	2	2.6	102	2921200	17074000	2921200	2921200	5.8448583	0.1710905	0.1710905
P62857	40S ribosomal protein S28	RPS28	1	2	2	27.5	7.8409	2921200	16451000	2921200	2921200	5.6315898	0.1775698	0.1775698
Q8IUC1	Keratin-associated protein 11-1	KRTAP11-1	1	2	2	7.4	17.085	2921200	15539000	2921200	2921200	5.3193893	0.1879915	0.1879915
P78559	Microtubule-associated protein 1A;MAP1A heavy chain;M	MAP1A	1	2	2	0.8	305.48	2921200	14588000	2921200	2921200	4.9938381	0.2002468	0.2002468
O14773	Tripeptidyl-peptidase 1	TPP1	1	2	2	4.3	61.247	2921200	14282000	2921200	2921200	4.8890867	0.2045372	0.2045372
P23396	40S ribosomal protein S3	RPS3	1	2	2	10.3	26.688	2921200	13752000	2921200	2921200	4.7076544	0.21242	0.21242
Q6ZUT6	Uncharacterized protein C15orf52	C15orf52	1	3	3	6.9	57.324	2921200	12895000	2921200	2921200	4.4142818	0.2265374	0.2265374
Q15366;P5	Poly(rC)-binding protein 2;Poly(rC)-binding protein 3	PCBP2;PCBP3	2	4	3	13.4	38.58	2921200	11555000	2921200	2921200	3.9555662	0.2528083	0.2528083
Q16698	2,4-dienoyl-CoA reductase, mitochondrial	DECR1	1	2	2	6.9	36.067	2921200	11453000	2921200	2921200	3.920649	0.2550598	0.2550598
Q9NVA2	**Septin-11**	**SEPT11**	1	2	2	5.4	49.398	2921200	10469000	2921200	2921200	3.5838012	0.2790333	0.2790333
P10619	Lysosomal protective protein;Lysosomal protective prote	CTSA	1	2	2	5.4	54.465	2921200	10463000	2921200	2921200	3.5817472	0.2791933	0.2791933
P36578	60S ribosomal protein L4	RPL4	1	2	2	4.4	47.697	2921200	9256100	2921200	2921200	3.1685951	0.3155973	0.3155973
Q9NZL9	Methionine adenosyltransferase 2 subunit beta	MAT2B	1	2	2	7.2	37.551	2921200	9047000	2921200	2921200	3.0970149	0.3228916	0.3228916
P19623	Spermidine synthase	SRM	1	2	2	7.6	33.824	2921200	8651800	2921200	2921200	2.9617281	0.3376407	0.3376407
Q8NC51	Plasminogen activator inhibitor 1 RNA-binding protein	SERBP1	1	2	2	6.6	44.965	2921200	8622700	2921200	2921200	2.9517664	0.3387802	0.3387802
Q99439;Q	Calponin-2;Calponin-3	CNN2;CNN3	2	2	2	5.8	33.697	2921200	8521000	2921200	2921200	2.9169519	0.3428236	0.3428236
P00387	NADH-cytochrome b5 reductase 3;NADH-cytochrome b	CYB5R3	1	2	2	9.3	34.234	2921200	8063000	2921200	2921200	2.7601671	0.3622969	0.3622969

Q07020	60S ribosomal protein L18	RPL18	1	2	2	13.8	21.634	2921200	7640000	2921200	2921200	2.6153635	0.382356	0.382356
P28838	Cytosol aminopeptidase	LAP3	1	2	2	4.6	56.166	2921200	7278600	2921200	2921200	2.4916473	0.4013409	0.4013409
P02751	Fibronectin;Anastellin;Ugl-Y1;Ugl-Y2;Ugl-Y3	FN1	1	10	10	5.6	272.32	80857000	177790000	78716000	81360000	2.1988201	0.4427471	0.4576185
P32119	Peroxiredoxin-2	PRDX2	1	6	5	27.3	21.892	54100000	118750000	77133000	56085000	2.1950092	0.6495411	0.4722947
Q92841	Probable ATP-dependent RNA helicase DDX17	DDX17	1	2	2	3.6	80.272	2921200	5960400	2921200	2921200	2.0403944	0.4901013	0.4901013
O00571;O1	ATP-dependent RNA helicase DDX3X;ATP-dependent RI	DDX3X;DDX3Y	2	4	4	8.2	73.243	61769000	125630000	45864000	46424000	2.0338681	0.365072	0.3695296
P30044	Peroxiredoxin-5, mitochondrial	PRDX5	1	3	3	15	22.086	30238000	52776000	2921200	2921200	1.7453535	0.0553509	0.0553509
Q5VTE0;P6	Putative elongation factor 1-alpha-like 3;Elongation facto	EEF1A1P5;EEF1	3	6	6	15.8	50.184	905340000	1.482E+09	965900000	882470000	1.637175	0.6516664	0.5953785
P50995	Annexin A11	ANXA11	1	4	4	10.5	54.389	57926000	91462000	2921200	58381000	1.5789456	0.0319389	0.6383088
P62979;P6	Ubiquitin-40S ribosomal protein S27a;Ubiquitin;40S ribos	RPS27A;UBA52	4	4	4	23.7	17.965	97708000	147590000	64038000	69699000	1.5105211	0.4338912	0.4722474

Table S4. A representative list of human proteins whose expression was affected by MJ133 as determined by MS assay

Protein IDs	Protein names	Gene names	Number of proteins	Peptides	Unique peptides	Sequence coverage [%]	Mol. weight [kDa]	LFQ intensity_DMSO-1	LFQ intensity_DMSO-2	LFQ intensity_DMSO-3	LFQ intensity_MJ33-1	LFQ intensity_MJ33-2	LFQ intensity_MJ33-3	Ratio_MJ33/BLEO	P value
Q92552	28S ribosomal protein S27, mitochondrial	MRPS27	1	5	5	13.5	47.611	9740500	9803600	9120600	16094000	15894000	16007000	1.674359	8.92E-06
P63010	AP-2 complex subunit beta	AP2B1	1	28	14	39.5	104.55	1.21E+08	1.22E+08	1.22E+08	1.11E+08	1.11E+08	1.1E+08	0.912637	2.03E-05
P04040	Catalase	CAT	1	16	16	35.9	59.755	43170000	45454000	44026000	59865000	59569000	59571000	1.349453	2.14E-05
Q15746	Myosin light chain kinase, smooth muscle;M	MYLK	1	51	51	37.9	210.71	91750000	94468000	91668000	69353000	69928000	71168000	0.757321	2.97E-05
P14625;Q58FF3	Endoplasmin	HSP90B1	2	38	36	50.6	92.468	6.1E+08	6.17E+08	5.84E+08	3.97E+08	4.08E+08	3.95E+08	0.662478	4.77E-05
P51858	Hepatoma-derived growth factor	HDGF	1	10	9	42.5	26.788	1.6E+08	1.67E+08	1.64E+08	1.15E+08	1.05E+08	1.1E+08	0.670901	8.79E-05
Q05682;REV__O75334;REV__Q	Caldesmon	CALD1	3	26	26	31.5	93.23	4.99E+08	4.82E+08	4.81E+08	3.85E+08	3.92E+08	3.88E+08	0.797515	8.89E-05
P20936	Ras GTPase-activating protein 1	RASA1	1	10	10	14.8	116.4	1.1E+08	1.1E+08	1.1E+08	64636000	72409000	63969000	0.609873	9.47E-05
P55072	Transitional endoplasmic reticulum ATPase	VCP	1	39	39	60.9	89.321	5.51E+08	5.44E+08	5.39E+08	4.61E+08	4.71E+08	4.71E+08	0.859415	9.84E-05
P48681	Nestin	NES	1	66	66	52.1	177.44	7.36E+08	7.56E+08	7.2E+08	4.54E+08	4.73E+08	5E+08	0.644835	0.000105
P23284	Peptidyl-prolyl cis-trans isomerase B	PPIB	1	11	11	44.4	23.742	5.76E+08	5.58E+08	5.78E+08	4.6E+08	4.47E+08	4.48E+08	0.791722	0.000106
P35579;REV__Q9UKV3;P12883	Myosin-9	MYH9	7	124	104	56.5	226.53	1.76E+09	1.79E+09	1.85E+09	1.32E+09	1.36E+09	1.37E+09	0.75115	0.000113
Q9NYL2	Mitogen-activated protein kinase kinase kir	ZAK	1	3	3	6.4	91.154	7684700	8064200	7314700	11001000	10830000	11013000	1.424062	0.00013
Q08379	Golgin subfamily A member 2	GOLGA2	1	6	6	7	113.08	13157000	13008000	13850000	8602200	9116100	9037800	0.668652	0.000131
P15559	NAD(P)H dehydrogenase [quinone] 1	NQO1	1	8	8	24.5	30.867	2.33E+08	2.59E+08	2.37E+08	1.23E+08	1.23E+08	1.27E+08	0.511868	0.00014
P61158;Q9P1U1;Q9C0K3	Actin-related protein 3	ACTR3	3	20	20	63.9	47.371	3.01E+08	3.05E+08	3.05E+08	2.23E+08	2.38E+08	2.38E+08	0.766625	0.000142
Q07065	Cytoskeleton-associated protein 4	CKAP4	1	28	28	54.2	66.022	5.03E+08	4.92E+08	5.22E+08	3.78E+08	3.75E+08	3.81E+08	0.74805	0.000147
P11047	Laminin subunit gamma-1	LAMC1	1	21	21	18.1	177.6	69513000	75118000	68163000	39934000	40818000	41992000	0.576821	0.00017
P05120	Plasminogen activator inhibitor 2	SERPINB2	1	15	15	46.3	46.596	1.16E+08	1.22E+08	1.26E+08	71667000	77293000	73093000	0.608932	0.000179
Q8WWI1	LIM domain only protein 7	LMO7	1	39	39	30.4	192.69	59530000	54886000	60005000	35989000	36639000	36834000	0.627574	0.000196
Q9Y490	Talin-1	TLN1	1	101	93	55.9	269.76	3.71E+08	3.75E+08	3.64E+08	3.14E+08	3.02E+08	3.08E+08	0.83209	0.000196
P33176;Q12840;O60282	Kinesin-1 heavy chain	KIF5B	3	34	34	46.7	109.68	1.41E+08	1.46E+08	1.45E+08	1.1E+08	1.14E+08	1.17E+08	0.789645	0.000203
Q14315	Filamin-C	FLNC	1	151	137	70.5	291.02	1.11E+09	1.12E+09	1.16E+09	1.64E+09	1.69E+09	1.79E+09	1.512012	0.000214
Q8WX93;Q86TC9	Palladin	PALLD	2	22	22	20.8	150.56	2.12E+08	2.33E+08	2.19E+08	1.27E+08	1.39E+08	1.31E+08	0.597913	0.000247
Q96B97	SH3 domain-containing kinase-binding pro	SH3KBP1	1	9	9	18.2	73.125	29872000	30385000	31069000	22168000	23441000	23751000	0.759477	0.000252
P00505	Aspartate aminotransferase, mitochondrial	GOT2	1	11	11	32.3	47.517	1.11E+08	1.12E+08	1.07E+08	1.54E+08	1.62E+08	1.67E+08	1.466241	0.000279
P13667	Protein disulfide-isomerase A4	PDIA4	1	28	28	48.1	72.932	2.19E+08	2.32E+08	2.19E+08	1.43E+08	1.49E+08	1.58E+08	0.672998	0.000292
P07355;A6NMY6	Annexin A2;Putative annexin A2-like protei	ANXA2;ANXA2P2	2	27	27	64.6	38.604	2.45E+09	2.46E+09	2.47E+09	2.96E+09	3.09E+09	3.12E+09	1.240511	0.000303
P21980	Protein-glutamine gamma-glutamyltransfe	TGM2	1	30	30	53.4	77.328	5.21E+08	5.53E+08	5.1E+08	3.32E+08	3.61E+08	3.34E+08	0.648484	0.000307
O76074	cGMP-specific 3,5-cyclic phosphodiesteras	PDE5A	1	22	22	28.8	99.984	64257000	67171000	56062000	24177000	21847000	21828000	0.361897	0.000308
P50454	Serpin H1	SERPINH1	1	17	17	47.1	46.44	3.39E+08	3.66E+08	3.48E+08	1.89E+08	2E+08	2.23E+08	0.580921	0.000314
P02545	Prelamin-A/C;Lamin-A/C	LMNA	1	47	46	67.9	74.139	2.09E+09	2.1E+09	2.18E+09	1.8E+09	1.77E+09	1.76E+09	0.83763	0.000333
Q92841	Probable ATP-dependent RNA helicase DD	DDX17	1	21	15	37.4	80.272	1.61E+08	1.62E+08	1.67E+08	1.39E+08	1.41E+08	1.41E+08	0.860222	0.000346
Q9NSD9	Phenylalanine--tRNA ligase beta subunit	FARSB	1	13	13	25.6	66.115	47179000	51206000	50866000	70145000	67236000	66884000	1.368601	0.000375
Q8IVF2	Protein AHNAK2	AHNAK2	1	33	33	18.5	616.62	67222000	63969000	72593000	1.13E+08	1.27E+08	1.19E+08	1.762847	0.000384
Q9ULV4;Q6QEF8	Coronin-1C	CORO1C	2	16	16	34	53.248	1.84E+08	1.71E+08	1.74E+08	2.67E+08	2.47E+08	2.67E+08	1.477495	0.000388
P11413	Glucose-6-phosphate 1-dehydrogenase	G6PD	1	19	19	54.4	59.256	2.13E+08	2.15E+08	2.14E+08	1.77E+08	1.71E+08	1.82E+08	0.824322	0.000396
Q9NUQ6	SPATS2-like protein	SPATS2L	1	13	13	32.6	61.728	1.73E+08	1.79E+08	1.74E+08	1.34E+08	1.21E+08	1.2E+08	0.711763	0.000455
P21333	Filamin-A	FLNA	1	125	113	66.3	280.74	1.04E+09	1.01E+09	1.01E+09	8.24E+08	8.31E+08	8.69E+08	0.824834	0.00046
P78559	Microtubule-associated protein 1A;MAP1A	MAP1A	1	67	65	36.9	305.48	1.63E+08	1.67E+08	1.72E+08	1.17E+08	1.27E+08	1.28E+08	0.741609	0.000541
P29401	Transketolase	TKT	1	23	23	52	67.877	6.6E+08	7.09E+08	6.67E+08	5.33E+08	5.34E+08	5.3E+08	0.784576	0.000684
Q99536	Synaptic vesicle membrane protein VAT-1	VAT1	1	15	15	54.5	41.92	1.4E+08	1.18E+08	1.46E+08	2.4E+08	2.44E+08	2.23E+08	1.752445	0.000714
P18206	Vinculin	VCL	1	52	52	52.6	123.8	3.95E+08	4.02E+08	3.89E+08	3.32E+08	3.38E+08	3.48E+08	0.858526	0.000721
P54619;Q9UGJ0	5-AMP-activated protein kinase subunit ga	PRKAG1	2	7	7	27.2	37.579	26698000	30272000	27225000	40124000	42526000	40043000	1.457248	0.000744
Q15758	Neutral amino acid transporter B(0)	SLC1A5	1	4	4	8.1	56.598	46051000	52658000	53977000	25184000	26108000	28377000	0.521783	0.000757
P15374	Ubiquitin carboxyl-terminal hydrolase isozy	UCHL3	1	8	8	48.3	26.182	32454000	32021000	34440000	52380000	53926000	59990000	1.681201	0.000772
P16615;O14983;Q93084	Sarcoplasmic/endoplasmic reticulum calciu	ATP2A2	3	29	29	34.5	114.76	1.29E+08	1.31E+08	1.24E+08	89271000	96645000	99255000	0.74271	0.000773
P21589	5-nucleotidase	NT5E	1	16	16	44.4	63.367	1.74E+08	1.83E+08	1.66E+08	2.56E+08	2.39E+08	2.38E+08	1.404616	0.000776
Q9UH99	SUN domain-containing protein 2	SUN2	1	6	6	13.8	80.31	27534000	29528000	30485000	18266000	15474000	18391000	0.595463	0.00079
P30041	Peroxiredoxin-6	PRDX6	1	18	18	69.6	25.035	1.07E+09	1.2E+09	1.08E+09	5.85E+08	6.39E+08	6.95E+08	0.572374	0.000823
P07237	Protein disulfide-isomerase	P4HB	1	25	25	55.5	57.116	5.57E+08	5.52E+08	5.41E+08	3.94E+08	4.22E+08	4.44E+08	0.763778	0.000979
P13797;P13796;Q14651	Plastin-3	PLS3	3	24	24	46.2	70.81	2.35E+08	2.48E+08	2.46E+08	2.02E+08	1.93E+08	2.04E+08	0.822233	0.00105

O60711	Leupaxin	LPXN	1	6	6	27.7	43.332	36029000	39490000	32967000	15384000	19314000	17654000	0.482569	0.001051
P68032;P68133	Actin, alpha cardiac muscle 1;Actin, alpha s	ACTC1;ACTA1	2	22	1	68.4	42.019	3.78E+09	4.03E+09	3.47E+09	2.1E+09	2.3E+09	2.34E+09	0.597723	0.001056
Q13813	Spectrin alpha chain, non-erythrocytic 1	SPTAN1	1	91	91	47.2	284.54	75679000	78823000	76151000	90155000	90127000	94656000	1.191998	0.001193
P24534	Elongation factor 1-beta	EEF1B2	1	6	6	41.3	24.763	4.12E+08	4.28E+08	4.02E+08	2.94E+08	3.16E+08	3.28E+08	0.755316	0.001236
P05556	Integrin beta-1	ITGB1	1	23	23	32.3	88.414	5.37E+08	5.38E+08	5.15E+08	3.99E+08	4.27E+08	4.37E+08	0.794591	0.001314
Q0ZGT2	Nexilin	NEXN	1	16	16	25	80.657	1.2E+08	1.32E+08	1.23E+08	71767000	77853000	86617000	0.630638	0.001332
Q14247	Src substrate cortactin	CTTN	1	23	23	49.3	61.585	2.07E+08	2.19E+08	2.12E+08	2.65E+08	2.53E+08	2.71E+08	1.238272	0.001437
P24752	Acetyl-CoA acetyltransferase, mitochondria	ACAT1	1	11	11	34.4	45.199	69748000	60254000	71295000	1.05E+08	98284000	96586000	1.487255	0.001481
Q86UE4	Protein LYRIC	MTDH	1	15	15	32.5	63.836	55387000	58989000	59905000	46716000	47664000	46128000	0.806215	0.001482
P61421	V-type proton ATPase subunit d 1	ATP6V0D1	1	4	4	20.8	40.329	28548000	31507000	28296000	40931000	42399000	38861000	1.383018	0.00149
P27105	Erythrocyte band 7 integral membrane pro	STOM	1	9	9	45.5	31.73	2.13E+08	2.33E+08	2.22E+08	1.57E+08	1.69E+08	1.72E+08	0.746353	0.001551
Q16881	Thioredoxin reductase 1, cytoplasmic	TXNRD1	1	20	20	44.8	70.905	4.18E+08	4.29E+08	4.31E+08	3.12E+08	3.09E+08	3.49E+08	0.759676	0.0016
P62277	40S ribosomal protein S13	RPS13	1	6	6	43	17.222	3.62E+08	3.09E+08	3.36E+08	4.84E+08	4.53E+08	4.67E+08	1.394873	0.001628
Q9BTT0	Acidic leucine-rich nuclear phosphoprotein	ANP32E	1	5	5	23.1	30.692	54861000	65197000	61333000	37806000	36562000	38389000	0.621624	0.001717
Q15436	Protein transport protein Sec23A	SEC23A	1	17	17	30.6	86.16	1.24E+08	1.26E+08	1.31E+08	93476000	1.04E+08	94673000	0.765764	0.001785
P16070	CD44 antigen	CD44	1	10	10	11.9	81.537	7.04E+08	6.77E+08	7.09E+08	5.7E+08	5.03E+08	5.12E+08	0.757912	0.001847
Q02790	Peptidyl-prolyl cis-trans isomerase FKBP4;F	FKBP4	1	11	11	32	51.804	52824000	48513000	50060000	34389000	38551000	38186000	0.734004	0.001851
P41091;Q2VIR3	Eukaryotic translation initiation factor 2 sub	EIF2S3;EIF2S3L	2	13	13	38.8	51.109	83710000	83015000	84387000	73433000	77214000	75306000	0.89981	0.001949
P07686	Beta-hexosaminidase subunit beta;Beta-he	HEXB	1	15	15	30	63.137	2.74E+08	2.87E+08	2.43E+08	3.77E+08	3.84E+08	3.61E+08	1.395229	0.00195
Q00610;P53675	Clathrin heavy chain 1	CLTC	2	72	72	53	191.61	7.49E+08	7.72E+08	7.56E+08	6.84E+08	7.01E+08	7E+08	0.91536	0.001957
Q9UHG3	Prenylcysteine oxidase 1	PCYOX1	1	10	10	24	56.639	66183000	61540000	62182000	73393000	73951000	73929000	1.165177	0.002033
Q9BZE1	39S ribosomal protein L37, mitochondrial	MRPL37	1	5	5	21.5	48.117	22173000	27372000	26676000	39366000	39787000	44008000	1.615841	0.002075
P48556	26S proteasome non-ATPase regulatory su	PSMD8	1	3	3	10	39.611	48298000	47896000	52011000	38444000	38156000	40330000	0.788975	0.002123
P07602	Prosaposin;Saposin-A;Saposin-B-Val;Sapos	PSAP	1	14	14	35.3	58.112	7.33E+08	7.07E+08	7.37E+08	9.16E+08	9.82E+08	8.9E+08	1.280823	0.00216
P19105;O14950	Myosin regulatory light chain 12A;Myosin r	MYL12A;MYL12B	2	9	3	62.6	19.794	1.19E+09	1.12E+09	1.14E+09	9.15E+08	9.71E+08	9.67E+08	0.825483	0.002277
P07942	Laminin subunit beta-1	LAMB1	1	16	16	12.7	198.04	66729000	71806000	56057000	31349000	29021000	33737000	0.483612	0.002282
Q9H4A4	Aminopeptidase B	RNPEP	1	19	19	33.7	72.595	1.76E+08	1.72E+08	1.75E+08	1.88E+08	1.85E+08	1.91E+08	1.078843	0.002318
P12814;P35609;Q08043	Alpha-actinin-1	ACTN1	3	56	39	71.6	103.06	3.91E+09	4.16E+09	3.71E+09	2.76E+09	2.87E+09	3.03E+09	0.735978	0.002329
Q9UBS4	DnaJ homolog subfamily B member 11	DNAJB11	1	8	8	30.7	40.513	54896000	51700000	53296000	29841000	38101000	36702000	0.654467	0.002463
Q92820	Gamma-glutamyl hydrolase	GGH	1	4	4	16.4	35.964	11140000	11602000	14836000	27369000	32127000	36559000	2.55615	0.002542
Q06210;O94808	Glutamine--fructose-6-phosphate aminot	GFPT1	2	29	29	52.1	78.806	1.69E+08	1.55E+08	1.73E+08	1.29E+08	1.21E+08	1.2E+08	0.744595	0.002581
P31943	Heterogeneous nuclear ribonucleoprotein I	HNRNPH1	1	15	10	44.3	49.229	2.8E+08	2.94E+08	2.74E+08	2.02E+08	2.26E+08	2.25E+08	0.77079	0.002699
Q9UHD8	Septin-9	9-Sep	1	23	23	46.6	65.401	1.19E+08	1.21E+08	1.22E+08	88038000	94742000	1.01E+08	0.784227	0.002759
Q9UBE0	SUMO-activating enzyme subunit 1;SUMO	SAE1	1	8	8	34.1	38.449	46993000	47990000	51783000	37228000	39632000	38184000	0.78386	0.002832
P26639	Threonine--tRNA ligase, cytoplasmic	TARS	1	18	18	31.1	83.434	88747000	91436000	82790000	65700000	67878000	71037000	0.778084	0.002872
Q9NZN4	EH domain-containing protein 2	EHD2	1	19	17	48.6	61.161	1.14E+08	1.15E+08	1.26E+08	75968000	88833000	83963000	0.701178	0.002885
Q02809	Procollagen-lysine,2-oxoglutarate 5-dioxyg	PLOD1	1	17	17	35.6	83.549	46209000	45013000	45954000	33550000	35051000	38626000	0.781675	0.002982
Q07812	Apoptosis regulator BAX	BAX	1	6	6	43.8	21.184	1.08E+08	1.19E+08	99763000	1.45E+08	1.52E+08	1.49E+08	1.364077	0.002985
P09960	Leukotriene A-4 hydrolase	LTA4H	1	16	16	37.2	69.284	45451000	42795000	43233000	54003000	58662000	53690000	1.265259	0.002994
P48960	CD97 antigen;CD97 antigen subunit alpha;(	CD97	1	4	4	9.2	91.868	26358000	27028000	25864000	13721000	18874000	16922000	0.62482	0.002994
Q9H425	Uncharacterized protein C1orf198	C1orf198	1	5	5	26.6	36.346	16572000	18411000	17499000	13638000	13276000	12145000	0.744236	0.003006
Q08170	Serine/arginine-rich splicing factor 4	SRSF4	1	5	3	11.1	56.678	87261000	88313000	82979000	62782000	56621000	68106000	0.725225	0.003053
O75718	Cartilage-associated protein	CRTAP	1	8	8	24.7	46.561	37712000	46409000	39944000	23258000	21673000	25246000	0.565647	0.003055
Q04917	14-3-3 protein eta	YWHAH	1	12	9	50.8	28.218	1.83E+08	1.95E+08	1.78E+08	2.21E+08	2.2E+08	2.29E+08	1.20351	0.003105
P07203	Glutathione peroxidase 1	GPX1	1	10	10	65	22.088	74705000	84656000	86149000	1.05E+08	1.09E+08	1.12E+08	1.324793	0.003111
Q9P2E9;Q8N4C6	Ribosome-binding protein 1	RRBP1	2	52	52	42.9	152.45	1.93E+08	1.95E+08	1.96E+08	1.31E+08	1.46E+08	1.58E+08	0.746865	0.003181
P53597	Succinyl-CoA ligase [ADP/GDP-forming] su	SUCLG1	1	3	3	12.4	36.249	19402000	26725000	26600000	55364000	43983000	51291000	2.07128	0.003227
P26006	Integrin alpha-3;Integrin alpha-3 heavy cha	ITGA3	1	13	13	15.7	116.61	96808000	1.04E+08	1.05E+08	1.37E+08	1.38E+08	1.56E+08	1.40774	0.00353
Q96AQ6	Pre-B-cell leukemia transcription factor-int	PBXIP1	1	15	15	25.3	80.642	68926000	73137000	62653000	44616000	45126000	50157000	0.683381	0.003579
Q9H2D6	TRIO and F-actin-binding protein	TRIOBP	1	13	13	6.6	261.37	38987000	37671000	39156000	30910000	33734000	30413000	0.820773	0.003671
Q16181	Septin-7	7-Sep	1	13	13	33.4	50.679	1.87E+08	2.04E+08	1.98E+08	1.47E+08	1.38E+08	1.6E+08	0.756064	0.003773
P46926	Glucosamine-6-phosphate isomerase 1	GNPDA1	1	10	7	53.6	32.668	77579000	90659000	80269000	1.28E+08	1.46E+08	1.61E+08	1.753834	0.003851
O00154	Cytosolic acyl coenzyme A thioester hydrol	ACOT7	1	10	10	30.3	41.796	50914000	56164000	54079000	65338000	71728000	67568000	1.26978	0.003884
P31948	Stress-induced-phosphoprotein 1	STIP1	1	21	21	41.3	62.639	2.39E+08	2.42E+08	2.34E+08	2.54E+08	2.57E+08	2.53E+08	1.068046	0.003907
Q96QD8	Sodium-coupled neutral amino acid transp	SLC38A2	1	3	3	10.5	56.025	73249000	90581000	72655000	42040000	44778000	43122000	0.549464	0.003915
P51452	Dual specificity protein phosphatase 3	DUSP3	1	5	5	34.6	20.478	34359000	33387000	35177000	47867000	45894000	41913000	1.318209	0.003933
Q7KZF4	Staphylococcal nuclease domain-containin	SND1	1	27	27	38.5	102	1.18E+08	1.26E+08	1.2E+08	94528000	93915000	1.04E+08	0.804448	0.004029
Q9UPN3;O94854	Microtubule-actin cross-linking factor 1, iso	MACF1	2	90	88	17.2	838.3	2.73E+08	2.62E+08	2.62E+08	2.15E+08	2.2E+08	2.36E+08	0.841197	0.004143
P30101	Protein disulfide-isomerase A3	PDIA3	1	28	28	62.2	56.782	6.37E+08	7.06E+08	6.57E+08	4.68E+08	4.86E+08	5.37E+08	0.74461	0.004298

P56199	Integrin alpha-1	ITGA1	1	9	9	9.2	130.85	34495000	28814000	36574000	18492000	19314000	20388000	0.582622	0.004311
P23526	Adenosylhomocysteinase	AHCY	1	15	15	38.7	47.716	95680000	99997000	1.04E+08	1.2E+08	1.19E+08	1.29E+08	1.226614	0.004385
P07099	Epoxide hydrolase 1	EPHX1	1	12	12	33.6	52.948	1.59E+08	1.58E+08	1.59E+08	1.28E+08	1.39E+08	1.41E+08	0.855892	0.004424
Q8N474	Secreted frizzled-related protein 1	SFRP1	1	3	3	15.3	35.385	13904000	13031000	13121000	31723000	24091000	33095000	2.219618	0.004434
Q96DG6	Carboxymethylenebutenolidase homolog	CMBL	1	5	5	22	28.048	27257000	28681000	31330000	35886000	38337000	38636000	1.293246	0.004469
Q9UJ70	N-acetyl-D-glucosamine kinase	NAGK	1	10	10	40.4	37.375	86813000	86930000	82799000	97435000	93522000	96938000	1.122214	0.004669
P36551	Oxygen-dependent coproporphyrinogen-I	CPOX	1	8	8	26.2	50.151	42576000	47877000	45407000	63696000	63974000	74611000	1.488893	0.004776
P06737	Glycogen phosphorylase, liver form	PYGL	1	19	16	26.6	97.147	1.92E+08	1.61E+08	1.96E+08	2.52E+08	2.77E+08	2.52E+08	1.42249	0.004851
P48163	NADP-dependent malic enzyme	ME1	1	12	12	26.6	64.149	71003000	78357000	76077000	58847000	63292000	60565000	0.810444	0.004892
P07900;Q14568;Q58FG0	Heat shock protein HSP 90-alpha	HSP90AA1	3	40	27	58.2	84.659	1.35E+09	1.31E+09	1.41E+09	1.62E+09	1.53E+09	1.6E+09	1.166135	0.005067
P09874	Poly [ADP-ribose] polymerase 1	PARP1	1	22	22	28.4	113.08	61965000	53990000	62741000	79186000	80083000	74438000	1.307847	0.005133
P09972	Fructose-bisphosphate aldolase C	ALDOC	1	4	2	14.8	39.455	1.24E+08	1.25E+08	1.32E+08	73230000	82213000	97960000	0.665554	0.005205
Q9NR12	PDZ and LIM domain protein 7	PDLIM7	1	15	15	52.7	49.844	1.61E+08	1.43E+08	1.62E+08	1.15E+08	1.22E+08	1.19E+08	0.76422	0.005218
O94855	Protein transport protein Sec24D	SEC24D	1	8	8	11.1	113.01	31264000	37414000	33348000	22632000	22758000	18389000	0.625125	0.005238
Q07020	60S ribosomal protein L18	RPL18	1	4	4	25.5	21.634	2.52E+08	2.44E+08	2.21E+08	3.88E+08	3.69E+08	3.2E+08	1.503595	0.005548
P27797	Calreticulin	CALR	1	18	18	57.6	48.141	1.11E+09	1.2E+09	1.06E+09	6.97E+08	7.18E+08	8.66E+08	0.675085	0.005642
P46821	Microtubule-associated protein 1B;MAP1B	MAP1B	1	53	51	33.4	270.63	91433000	89604000	88794000	83883000	85155000	85613000	0.943743	0.00568
O00303	Eukaryotic translation initiation factor 3 sub	EIF3F	1	6	6	25.5	37.563	81000000	80422000	76676000	68461000	71330000	71401000	0.886996	0.005752
Q5T9L3	Protein wntless homolog	WLS	1	2	2	5.9	62.253	30200000	24999000	26016000	11821000	17529000	15091000	0.547202	0.005921
Q92522	Histone H1x	H1FX	1	2	2	11.7	22.487	35308000	39739000	37203000	27191000	24514000	29538000	0.723768	0.005947
Q70UQ0	Inhibitor of nuclear factor kappa-B kinase-	IKBIP	1	9	9	27.4	39.309	79258000	68377000	65829000	47448000	49563000	49953000	0.688472	0.006131
Q9Y2A7	Nck-associated protein 1	NCKAP1	1	11	11	12	128.79	52301000	47080000	51462000	58918000	58799000	58648000	1.169196	0.006299
O60664	Perilipin-3	PLIN3	1	19	19	68.7	47.074	3.87E+08	3.9E+08	3.77E+08	3.18E+08	3.28E+08	3.5E+08	0.861927	0.006328
P23381	Tryptophan--tRNA ligase, cytoplasmic;T1-T	WARS	1	15	15	46.1	53.165	2.18E+08	2.15E+08	1.9E+08	1.47E+08	1.62E+08	1.58E+08	0.747755	0.006345
P02751	Fibronectin;Anastellin;Ugl-Y1;Ugl-Y2;Ugl-Y	FN1	1	25	25	15.4	272.32	43427000	49396000	51885000	35543000	34993000	34424000	0.725323	0.006356
P15311	Ezrin	EZR	1	22	12	36.9	69.412	1.21E+08	1.36E+08	1.09E+08	2.04E+08	1.72E+08	2.1E+08	1.595443	0.00656
P51636	Caveolin-2	CAV2	1	4	4	43.8	18.291	2.15E+08	2.42E+08	2.57E+08	1.12E+08	1.42E+08	1.63E+08	0.58422	0.006644
Q9Y639	Neuroplastin	NPTN	1	5	5	15.1	44.387	68739000	62279000	62421000	26818000	41223000	42108000	0.569425	0.006759
P12268;P20839	Inosine-5-monophosphate dehydrogenase	IMPDH2	2	19	19	43.6	55.804	84077000	89763000	87439000	1.3E+08	1.16E+08	1.44E+08	1.495872	0.006786
Q8NC56	LEM domain-containing protein 2	LEMD2	1	4	4	9.3	56.974	35879000	36193000	37571000	27491000	28420000	31838000	0.800316	0.006796
Q9UBY9	Heat shock protein beta-7	HSPB7	1	5	5	44.1	18.61	99319000	1.08E+08	1.21E+08	75098000	75875000	78671000	0.69856	0.006849
P41240	Tyrosine-protein kinase CSK	CSK	1	4	4	12.7	50.704	NaN	9312500	9580800	NaN	12429000	12896000	1.340422	0.006939
Q15149;P58107	Plectin	PLEC	2	215	214	50.5	531.78	4.98E+08	5.14E+08	5.08E+08	4.6E+08	4.55E+08	4.78E+08	0.915952	0.006956
P60891;P21108	Ribose-phosphate pyrophosphokinase 1	PRPS1	2	6	5	28	34.834	17122000	15279000	18508000	24236000	22135000	25632000	1.414347	0.007027
Q10471	Polypeptide N-acetylgalactosaminyltransfe	GALNT2	1	15	15	34.3	64.732	50213000	61855000	57024000	80893000	73093000	82178000	1.39666	0.00714
P31937	3-hydroxyisobutyrate dehydrogenase, mito	HIBADH	1	5	5	24.4	35.329	19046000	23172000	20716000	32323000	31263000	27603000	1.448962	0.007238
Q16539	Mitogen-activated protein kinase 14	MAPK14	1	7	7	23.9	41.293	18398000	14650000	15940000	24184000	24054000	21624000	1.426104	0.007253
Q99519	Sialidase-1	NEU1	1	4	4	11.8	45.467	18356000	16858000	19041000	14973000	13810000	13336000	0.776316	0.007438
Q9HD45	Transmembrane 9 superfamily member 3	TM9SF3	1	4	4	8.7	67.887	47762000	45059000	43783000	38845000	39301000	36445000	0.838855	0.007533
Q8WUM4	Programmed cell death 6-interacting prote	PDCD6IP	1	33	33	48.2	96.022	1.08E+08	1.07E+08	1.04E+08	1.21E+08	1.24E+08	1.15E+08	1.13265	0.007666
P06865	Beta-hexosaminidase subunit alpha	HEXA	1	7	7	14.6	60.702	1.22E+08	78976000	1.01E+08	1.58E+08	1.81E+08	1.79E+08	1.713176	0.007751
P53004	Biliverdin reductase A	BLVRA	1	8	8	25.7	33.428	41355000	35176000	49369000	65398000	74309000	63849000	1.616807	0.00784
P27824	Calnexin	CANX	1	16	16	25.5	67.567	3.22E+08	2.96E+08	3.37E+08	2.47E+08	2.44E+08	2.65E+08	0.791236	0.007903
P16949	Stathmin	STMN1	1	6	5	35.6	17.302	1.08E+08	1.38E+08	1.14E+08	2E+08	2.04E+08	1.69E+08	1.591298	0.008097
P11216	Glycogen phosphorylase, brain form	PYGB	1	29	23	41.3	96.695	75661000	80673000	86341000	96030000	1.04E+08	1.06E+08	1.260863	0.008143
O75477	Erlin-1	ERLIN1	1	6	4	20.7	39.171	17323000	17890000	21601000	26604000	27397000	24994000	1.390414	0.008185
P35610	Sterol O-acyltransferase 1	SOAT1	1	2	2	5.5	64.734	22785000	21521000	15992000	32617000	38926000	32414000	1.724054	0.008204
Q9Y333	U6 snRNA-associated Sm-like protein LSm	LSM2	1	4	4	60	10.834	20204000	20478000	19425000	15880000	16935000	17840000	0.842747	0.008278
Q86VP6;O75155	Cullin-associated NEDD8-dissociated prote	CAND1	2	23	23	24	136.37	1.68E+08	1.66E+08	1.59E+08	1.29E+08	1.46E+08	1.35E+08	0.833496	0.008401
P17980	26S protease regulatory subunit 6A	PSMC3	1	11	11	33.7	49.203	1.05E+08	1.01E+08	1.04E+08	72772000	83241000	87692000	0.785461	0.008667
P78417	Glutathione S-transferase omega-1	GSTO1	1	14	14	47.3	27.566	4.2E+08	3.91E+08	4.27E+08	5.32E+08	5.51E+08	4.84E+08	1.264984	0.008701
O95340	Bifunctional 3-phosphoadenosine 5-phosp	PAPSS2	1	14	14	25.2	69.5	82763000	68916000	82592000	56988000	55460000	50886000	0.697201	0.008734
P38606	V-type proton ATPase catalytic subunit A	ATP6V1A	1	19	19	39.1	68.303	57673000	69577000	65996000	86171000	80070000	83392000	1.291789	0.008857
P22570	NADPH:adrenodoxin oxidoreductase, mito	FDXR	1	18	18	43.2	53.836	72296000	70925000	77645000	88358000	88562000	83003000	1.176836	0.008977
Q86UU1	Pleckstrin homology-like domain family B r	PHLDB1	1	11	11	10.2	151.16	22519000	24001000	21790000	19684000	17559000	18293000	0.813	0.009124
Q9NYU2	UDP-glucose:glycoprotein glucosyltransfer	UGGT1	1	23	23	21.5	177.19	41509000	43971000	46418000	34704000	36613000	37452000	0.824645	0.009179
P24844	Myosin regulatory light polypeptide 9	MYL9	1	9	3	62.2	19.827	1.77E+08	1.81E+08	1.32E+08	90580000	91145000	87279000	0.54901	0.009197
P31150	Rab GDP dissociation inhibitor alpha	GDI1	1	20	14	55.9	50.582	1.24E+08	1.28E+08	1.24E+08	1.12E+08	1.05E+08	97567000	0.836403	0.009198
P28161	Glutathione S-transferase Mu 2	GSTM2	1	13	7	56.4	25.744	93297000	94277000	82555000	1.13E+08	1.07E+08	1.08E+08	1.218122	0.009233

Q969G5	Protein kinase C delta-binding protein	PRKCDBP	1	7	7	29.9	27.701	51014000	56947000	50582000	39289000	38246000	29814000	0.677097	0.009348
P31942	Heterogeneous nuclear ribonucleoprotein	HNRNPH3	1	6	6	25.1	36.926	88193000	95349000	97433000	65476000	64787000	78125000	0.74166	0.009386
O00159	Unconventional myosin-Ic	MYO1C	1	27	27	32.9	121.68	1.23E+08	1.33E+08	1.4E+08	93454000	95491000	1.09E+08	0.753853	0.009421
O75379	Vesicle-associated membrane protein 4	VAMP4	1	3	3	31.9	16.397	24917000	29020000	26356000	18530000	20202000	15252000	0.672338	0.009668
Q7L5N7	Lysophosphatidylcholine acyltransferase 2	LPCAT2	1	7	7	20.8	60.207	40930000	41097000	46992000	33949000	33596000	33709000	0.784799	0.00976
P08195	4F2 cell-surface antigen heavy chain	SLC3A2	1	8	8	19.8	67.993	67430000	74919000	62590000	42984000	35805000	50118000	0.629002	0.009791
Q14257	Reticulocalbin-2	RCN2	1	7	7	26.2	36.876	68078000	59104000	65170000	48568000	47324000	52942000	0.773759	0.009937
P06576	ATP synthase subunit beta, mitochondrial	ATP5B	1	24	24	65.4	56.559	5.13E+08	5.54E+08	5.25E+08	6.05E+08	5.82E+08	5.97E+08	1.120597	0.009991
Q14254	Flotillin-2	FLOT2	1	14	14	37.9	47.064	32198000	30311000	29894000	38629000	43781000	37693000	1.299774	0.010272
P31040	Succinate dehydrogenase [ubiquinone] flav	SDHA	1	15	15	37.2	72.691	1.66E+08	1.73E+08	1.49E+08	1.94E+08	2.12E+08	2.04E+08	1.251138	0.010345
P48507	Glutamate--cysteine ligase regulatory subu	GCLM	1	3	3	16.1	30.727	36905000	34232000	43673000	21501000	22745000	26683000	0.617795	0.010415
O15027	Protein transport protein Sec16A	SEC16A	1	11	11	7.8	251.89	25695000	25180000	27218000	19078000	19134000	22466000	0.776997	0.010432
Q1KMD3	Heterogeneous nuclear ribonucleoprotein	HNRNPUL2	1	14	14	29.5	85.104	65025000	65993000	61818000	58530000	54331000	54385000	0.867297	0.010476
P55036;A2A3N6	26S proteasome non-ATPase regulatory su	PSMD4	2	8	8	30.5	40.736	1.21E+08	1.28E+08	1.36E+08	1.01E+08	1.02E+08	1.11E+08	0.813522	0.010557
P22626	Heterogeneous nuclear ribonucleoproteins	HNRNPA2B1	1	19	19	52.7	37.429	2.02E+09	2.05E+09	2.21E+09	1.59E+09	1.65E+09	1.82E+09	0.804956	0.010575
P11233	Ras-related protein Ral-A	RALA	1	3	2	19.4	23.567	32517000	36186000	35000000	40461000	42341000	39478000	1.179137	0.01063
P50479	PDZ and LIM domain protein 4	PDLIM4	1	14	14	64.8	35.398	89656000	97825000	90961000	76571000	76608000	82293000	0.845677	0.010644
P11021	78 kDa glucose-regulated protein	HSPA5	1	32	31	50.2	72.332	6.74E+08	6.7E+08	7.59E+08	5.66E+08	5.28E+08	5.72E+08	0.792711	0.010645
P10253	Lysosomal alpha-glucosidase;76 kDa lysos	GAA	1	15	15	22	105.32	84943000	91940000	90651000	98316000	1.03E+08	1.04E+08	1.143242	0.01067
O14967	Calmegin	CLGN	1	6	6	18.9	70.038	41568000	38998000	38466000	34496000	31262000	34195000	0.839715	0.010673
P30048	Thioredoxin-dependent peroxide reductase	PRDX3	1	8	8	52	27.692	2.39E+08	2.32E+08	2.02E+08	2.78E+08	2.8E+08	3.04E+08	1.281855	0.010728
Q8NEZ5	F-box only protein 22	FBXO22	1	6	6	18.4	44.508	22083000	19481000	18746000	24554000	26834000	27803000	1.313066	0.010786
P13473	Lysosome-associated membrane glycoprot	LAMP2	1	3	3	7.1	44.96	62975000	89490000	68081000	1.58E+08	1.33E+08	1.19E+08	1.861199	0.010838
P48147	Prolyl endopeptidase	PREP	1	8	8	16.1	80.699	56267000	59033000	54579000	46391000	48862000	41810000	0.806827	0.010963
P50552	Vasodilator-stimulated phosphoprotein	VASP	1	9	9	32.4	39.829	1E+08	1.04E+08	1.13E+08	77213000	89042000	80141000	0.776344	0.011026
Q13526;O15428	Peptidyl-prolyl cis-trans isomerase NIMA-i	PIN1	2	6	6	50.9	18.243	58070000	52871000	54740000	41169000	47714000	40929000	0.783506	0.011336
P56134	ATP synthase subunit f, mitochondrial	ATP5J2	1	3	3	39.4	10.918	NaN	84697000	80569000	1.02E+08	98392000	1.07E+08	1.237851	0.011394
P67936	Tropomyosin alpha-4 chain	TPM4	1	20	11	58.5	28.521	4.86E+08	4.96E+08	4.63E+08	3.26E+08	3.54E+08	4.1E+08	0.75467	0.011488
P51572	B-cell receptor-associated protein 31	BCAP31	1	10	10	30.1	27.991	2.81E+08	2.78E+08	2.91E+08	2.18E+08	2.53E+08	2.21E+08	0.815806	0.011521
P21291	Cysteine and glycine-rich protein 1	CSRP1	1	11	11	64.8	20.567	4.2E+09	3.68E+09	4.08E+09	4.71E+09	4.98E+09	4.69E+09	1.201116	0.011823
O60518	Ran-binding protein 6	RANBP6	1	4	3	5.4	124.71	NaN	31382000	31976000	26503000	24259000	26808000	0.816208	0.011901
Q9Y4L1;Q9BXT5	Hypoxia up-regulated protein 1	HYOU1	2	18	18	26.6	111.33	52246000	50245000	49266000	36363000	34612000	43456000	0.754041	0.011901
O14579	Coatomer subunit epsilon	COPE	1	7	7	37.7	34.482	1.1E+08	1.15E+08	1.08E+08	86805000	89708000	99589000	0.828414	0.011966
P37802	Transgelin-2	TAGLN2	1	14	13	80.9	22.391	7.93E+08	8.52E+08	8.02E+08	6.49E+08	6.73E+08	7.3E+08	0.838722	0.011972
O75964	ATP synthase subunit g, mitochondrial	ATP5L	1	3	3	35.9	11.428	1.17E+08	1.18E+08	1.17E+08	1.36E+08	1.42E+08	1.56E+08	1.229006	0.012075
P55209	Nucleosome assembly protein 1-like 1	NAP1L1	1	5	3	19.9	45.374	2.79E+08	2.76E+08	2.7E+08	2.05E+08	2.04E+08	2.43E+08	0.791229	0.012233
O14966;P57729	Ras-related protein Rab-7L1	RAB29	2	8	7	50.2	23.155	29331000	29067000	22201000	46430000	41066000	37795000	1.554498	0.012253
Q6NZI2	Polymerase I and transcript release factor	PTRF	1	11	11	41.5	43.476	2.98E+08	2.63E+08	2.88E+08	1.94E+08	2.17E+08	2.34E+08	0.76059	0.012265
Q96HE7;Q86YB8	ERO1-like protein alpha	ERO1L	2	11	11	31.4	54.392	47186000	40692000	51253000	61025000	60569000	58653000	1.29552	0.012282
P36543;Q96A05	V-type proton ATPase subunit E 1	ATP6V1E1	2	6	6	29.2	26.145	85893000	98017000	94403000	1.11E+08	1.12E+08	1.07E+08	1.186793	0.012329
P49419	Alpha-aminoadipic semialdehyde dehydro	ALDH7A1	1	8	8	24.3	58.486	22040000	18276000	22130000	26284000	26008000	26844000	1.267271	0.012637
Q15642	Cdc42-interacting protein 4	TRIP10	1	8	8	16.1	68.351	17542000	18756000	17414000	14338000	15987000	14254000	0.829964	0.012642
Q12906;Q96SI9	Interleukin enhancer-binding factor 3	ILF3	2	21	21	29.1	95.337	1.6E+08	1.69E+08	1.46E+08	1.25E+08	1.32E+08	1.24E+08	0.801671	0.012645
P61163	Alpha-centractin	ACTR1A	1	7	6	40.4	42.613	2.36E+08	2.14E+08	2.45E+08	1.86E+08	1.95E+08	1.87E+08	0.816844	0.012752
Q9BY44	Eukaryotic translation initiation factor 2A;Eu	EIF2A	1	7	7	19.7	64.989	25346000	23328000	25293000	19182000	19431000	21765000	0.816283	0.012801
Q6IAA8	Ragulator complex protein LAMTOR1	LAMTOR1	1	3	3	29.8	17.745	38059000	39875000	34303000	48825000	48772000	44014000	1.261714	0.012856
P30040	Endoplasmic reticulum resident protein 29	ERP29	1	6	6	27.6	28.993	78412000	84062000	83957000	90145000	92052000	95550000	1.127078	0.01299
Q99584	Protein S100-A13	S100A13	1	6	6	64.3	11.471	5.45E+08	6.62E+08	5.34E+08	3.78E+08	3.95E+08	4.22E+08	0.686238	0.013025
P62330	ADP-ribosylation factor 6	ARF6	1	4	4	31.4	20.082	44198000	56358000	45154000	68551000	63852000	72499000	1.406232	0.013089
Q9Y5S1	Transient receptor potential cation channel	TRPV2	1	10	10	16.9	85.98	52548000	41043000	47343000	32230000	33397000	32692000	0.697624	0.013173
P22695	Cytochrome b-c1 complex subunit 2, mito	UQCRC2	1	14	14	41.9	48.442	1.08E+08	1.03E+08	1.18E+08	1.39E+08	1.28E+08	1.41E+08	1.235984	0.013219
Q13308	Inactive tyrosine-protein kinase 7	PTK7	1	14	14	23.3	118.39	22901000	25265000	23461000	31184000	28830000	27646000	1.22384	0.013279
P12111	Collagen alpha-3(VI) chain	COL6A3	1	30	30	13.5	343.67	62916000	59030000	72072000	37096000	48743000	43293000	0.665567	0.013475
Q9UMS4	Pre-mRNA-processing factor 19	PRPF19	1	6	6	22.2	55.18	81582000	81305000	83786000	55726000	68417000	68308000	0.780187	0.013497
Q13740	CD166 antigen	ALCAM	1	9	9	22.8	65.102	38028000	31802000	37488000	25357000	28070000	26854000	0.748066	0.01358
P18031	Tyrosine-protein phosphatase non-recept	PTPN1	1	12	12	38.2	49.966	38941000	36810000	41790000	47039000	48959000	44885000	1.198586	0.013924
Q8TCT9	Minor histocompatibility antigen H13	HM13	1	8	8	21.2	41.488	1.21E+08	1.43E+08	1.35E+08	1.03E+08	1.01E+08	1.08E+08	0.781647	0.01393
P83876	Thioredoxin-like protein 4A	TXNL4A	1	2	2	15.5	16.786	1.07E+08	NaN	1.14E+08	78889000	74747000	NaN	0.694212	0.013936
P10809	60 kDa heat shock protein, mitochondrial	HSPD1	1	36	36	75.4	61.054	4.52E+08	4.77E+08	4.5E+08	5.22E+08	5.33E+08	5.84E+08	1.189866	0.014197

P17301	Integrin alpha-2	ITGA2	1	23	23	27.6	129.29	4E+08	3.8E+08	4.28E+08	3.41E+08	3.33E+08	3.51E+08	0.848495	0.014293
P46781	40S ribosomal protein S9	RPS9	1	7	7	31.4	22.591	2E+08	1.64E+08	2.01E+08	2.48E+08	2.49E+08	2.32E+08	1.291361	0.014309
P14314	Glucosidase 2 subunit beta	PRKCSH	1	15	15	35.6	59.425	3.87E+08	3.37E+08	3.49E+08	2.95E+08	2.93E+08	2.97E+08	0.824699	0.014419
Q9NZI8	Insulin-like growth factor 2 mRNA-binding	IGF2BP1	1	12	10	28.1	63.48	1.28E+08	1.45E+08	1.21E+08	97407000	84260000	1.02E+08	0.719552	0.014451
P14618;P30613	Pyruvate kinase PKM	PKM	2	38	38	72.1	57.936	2.8E+09	3.09E+09	2.81E+09	1.97E+09	2.19E+09	2.46E+09	0.761155	0.014867
O75367	Core histone macro-H2A.1	H2AFY	1	10	9	36	39.183	91842000	90189000	87866000	76924000	67487000	79213000	0.828553	0.014949
Q9Y450	HBS1-like protein	HBS1L	1	5	5	11	75.472	1.88E+08	1.77E+08	1.58E+08	3.3E+08	3.45E+08	2.44E+08	1.757771	0.015445
Q08378	Golgin subfamily A member 3	GOLGA3	1	14	14	12.9	167.35	23275000	24195000	21518000	17575000	19643000	19381000	0.820418	0.015464
P26640	Valine--tRNA ligase	VARS	1	27	27	28.3	140.47	1.11E+08	1.24E+08	1.27E+08	96602000	99429000	1.01E+08	0.821274	0.015515
O75521	Enoyl-CoA delta isomerase 2, mitochondria	ECI2	1	10	10	34.5	43.585	25514000	23365000	24483000	44793000	41746000	32456000	1.622025	0.015558
Q8WXF1	Paraspeckle component 1	PSPC1	1	5	5	11.7	58.743	50780000	50875000	62705000	35304000	33979000	40802000	0.66978	0.015585
P63151;Q66LE6;Q00005;Q9Y2	Serine/threonine-protein phosphatase 2A !	PPP2R2A	4	10	10	29.5	51.691	1.31E+08	1.5E+08	1.23E+08	1.72E+08	1.85E+08	1.67E+08	1.291184	0.015631
P11387	DNA topoisomerase 1	TOP1	1	12	10	21.2	90.725	53820000	48899000	54433000	45785000	45144000	43562000	0.855802	0.015665
P29144	Tripeptidyl-peptidase 2	TPP2	1	21	21	24.2	138.35	71822000	82115000	71118000	57191000	56977000	62545000	0.785199	0.01567
O43583	Density-regulated protein	DENR	1	3	3	21.2	22.092	68763000	67471000	NaN	62605000	62999000	NaN	0.921972	0.015766
O00299	Chloride intracellular channel protein 1	CLIC1	1	14	14	79.3	26.922	4.77E+08	5.25E+08	4.8E+08	4.14E+08	3.98E+08	4.36E+08	0.842164	0.015791
Q01433	AMP deaminase 2	AMPD2	1	11	11	14.9	100.69	37261000	38291000	44748000	29561000	31644000	27818000	0.740008	0.015811
P62269	40S ribosomal protein S18	RPS18	1	6	6	34.9	17.718	1.55E+08	2.11E+08	1.4E+08	3.45E+08	2.65E+08	2.85E+08	1.771083	0.016021
P28331	NADH-ubiquinone oxidoreductase 75 kDa	NDUFS1	1	14	14	30.3	79.467	43058000	45272000	41498000	35865000	39119000	35165000	0.848423	0.016065
Q9H3K6	BolA-like protein 2	BOLA2	1	3	3	39.5	10.116	65037000	66912000	71204000	48163000	57010000	56768000	0.797138	0.016135
O60701	UDP-glucose 6-dehydrogenase	UGDH	1	12	12	32.4	55.023	57117000	57382000	67396000	47358000	47422000	46401000	0.776168	0.016229
Q14103	Heterogeneous nuclear ribonucleoprotein I	HNRNPD	1	10	10	30.1	38.434	2.36E+08	2.68E+08	2.4E+08	1.82E+08	1.84E+08	2.12E+08	0.775983	0.016365
P35754	Glutaredoxin-1	GLRX	1	3	3	37.7	11.776	88702000	85928000	90171000	1.2E+08	1.04E+08	1.3E+08	1.335342	0.016372
P12277;P06732	Creatine kinase B-type	CKB	2	8	8	29.7	42.644	27878000	21113000	23652000	40972000	39206000	32180000	1.546715	0.016498
O14773	Tripeptidyl-peptidase 1	TPP1	1	4	4	9.9	61.247	1.06E+08	96144000	65261000	2.07E+08	1.89E+08	1.43E+08	2.013749	0.016571
Q99439	Calponin-2	CNN2	1	12	11	47.9	33.697	6.46E+08	7.36E+08	5.94E+08	5.01E+08	4.98E+08	4.62E+08	0.73899	0.016597
Q99733	Nucleosome assembly protein 1-like 4	NAP1L4	1	7	5	24.3	42.823	2.1E+08	1.88E+08	2.2E+08	1.69E+08	1.7E+08	1.66E+08	0.816848	0.01664
P32455;Q9H0R5	Interferon-induced guanylate-binding prot	GBP1	2	16	14	28	67.93	72924000	75878000	76111000	61170000	69127000	61591000	0.853165	0.016702
P41567;O60739	Eukaryotic translation initiation factor 1;Euk	EIF1;EIF1B	2	7	7	65.5	12.732	1.65E+08	1.51E+08	1.5E+08	1.2E+08	1.21E+08	1.37E+08	0.810746	0.016721
Q53EP0	Fibronectin type III domain-containing prot	FNDC3B	1	4	4	5.7	132.89	14511000	17002000	16772000	NaN	11180000	11055000	0.690742	0.016766
P23528	Cofilin-1	CFL1	1	15	13	72.3	18.502	1.63E+09	1.68E+09	1.7E+09	1.99E+09	1.84E+09	1.84E+09	1.132348	0.016771
P40926	Malate dehydrogenase, mitochondrial	MDH2	1	13	13	48.8	35.503	8.27E+08	9.37E+08	7.68E+08	1.09E+09	1.04E+09	1.03E+09	1.249166	0.016829
Q9C0C2	182 kDa tankyrase-1-binding protein	TNKS1BP1	1	26	26	26.1	181.79	45348000	45181000	43601000	34773000	39546000	39694000	0.850019	0.017206
P48735	Isocitrate dehydrogenase [NADP], mitocho	IDH2	1	11	10	30.1	50.909	39440000	40076000	37385000	44003000	49749000	45514000	1.191316	0.017248
P42224	Signal transducer and activator of transcrip	STAT1	1	29	29	46.9	87.334	2.47E+08	2.58E+08	2.77E+08	2.04E+08	2.1E+08	2.3E+08	0.82348	0.017467
Q04837	Single-stranded DNA-binding protein, mit	SSBP1	1	5	5	41.9	17.259	92250000	87012000	86132000	1.16E+08	1.26E+08	1.03E+08	1.301687	0.017562
Q01082;O15020	Spectrin beta chain, non-erythrocytic 1	SPTBN1	2	74	71	41.9	274.61	83990000	79544000	87385000	91974000	95859000	99273000	1.144218	0.017644
P62333	26S protease regulatory subunit 10B	PSMC6	1	15	15	47.8	44.172	1.01E+08	96640000	96689000	91485000	89790000	85251000	0.904923	0.017646
P09914;Q5T764	Interferon-induced protein with tetratricop	IFIT1	2	10	10	25.9	55.36	39299000	41215000	44559000	31606000	36273000	33191000	0.808088	0.017754
P47755	F-actin-capping protein subunit alpha-2	CAPZA2	1	10	8	58	32.949	50738000	51705000	55735000	46856000	45864000	47096000	0.883916	0.017784
P60709;Q6S8J3;A5A3E0;P0CG	Actin, cytoplasmic 1;Actin, cytoplasmic 1, N	ACTB	5	24	1	75.5	41.736	5.36E+09	5.26E+09	4.07E+09	2.82E+09	3.27E+09	3.39E+09	0.645231	0.01787
Q5GLZ8	Probable E3 ubiquitin-protein ligase HERC	HERC4	1	12	12	17.3	118.56	40575000	41912000	37429000	34393000	33757000	35389000	0.863429	0.018015
P19367;Q2TB90	Hexokinase-1	HK1	2	28	26	37.1	102.48	1.52E+08	1.35E+08	1.43E+08	1.63E+08	1.83E+08	1.73E+08	1.208955	0.018049
Q9BVK6	Transmembrane emp24 domain-containing	TMED9	1	7	6	26	27.277	1.35E+08	1.53E+08	1.38E+08	1.18E+08	1.13E+08	1.23E+08	0.830367	0.018204
Q16629	Serine/arginine-rich splicing factor 7	SRSF7	1	4	3	17.6	27.366	1.91E+08	2.03E+08	1.75E+08	1.32E+08	1.28E+08	1.6E+08	0.73737	0.018439
Q9NTJ5	Phosphatidylinositide phosphatase SAC1	SACM1L	1	8	8	16.7	66.966	29327000	26037000	24979000	22162000	21047000	19325000	0.778338	0.018522
P31946	14-3-3 protein beta/alpha;14-3-3 protein	YWHAB	1	15	9	65.4	28.082	4.53E+08	4.83E+08	5.12E+08	4.16E+08	4.14E+08	3.79E+08	0.835044	0.018529
Q63ZY3	KN motif and ankyrin repeat domain-conta	KANK2	1	12	12	19.7	91.173	29734000	32678000	31893000	19947000	25576000	25342000	0.751445	0.018569
Q7LG56;P31350	Ribonucleoside-diphosphate reductase sub	RRM2B	2	5	5	16.5	40.736	78796000	76092000	65734000	89263000	90970000	99962000	1.270023	0.018592
Q04760	Lactoylglutathione lyase	GLO1	1	8	8	51.1	20.777	46911000	58297000	60266000	87366000	79678000	1.09E+08	1.665422	0.018605
Q5TFE4	5-nucleotidase domain-containing protein	NT5DC1	1	6	6	20.4	51.844	14432000	18644000	20296000	23694000	28387000	28920000	1.517668	0.018735
P50416	Carnitine O-palmitoyltransferase 1, liver isc	CPT1A	1	11	11	16.8	88.367	37630000	39918000	42119000	34926000	35108000	33150000	0.862259	0.01879
P09382	Galectin-1	LGALS1	1	9	9	85.2	14.716	4.01E+09	4.22E+09	3.62E+09	3.1E+09	3.38E+09	3.12E+09	0.809698	0.018845
P41250	Glycine--tRNA ligase	GARS	1	16	16	25.4	83.165	1.21E+08	1.25E+08	1.29E+08	98823000	1.02E+08	1.14E+08	0.840352	0.018924
Q96RP9	Elongation factor G, mitochondrial	GFM1	1	4	4	6.8	83.471	30737000	29306000	25490000	41461000	37926000	NaN	1.392217	0.018929
Q9H910	Hematological and neurological expressed	HN1L	1	5	5	37.4	20.063	32656000	27426000	32560000	24128000	23085000	24842000	0.777779	0.018946
Q9NUQ8	ATP-binding cassette sub-family F membe	ABCF3	1	7	7	14.4	79.744	15695000	18138000	17450000	22196000	22665000	27569000	1.412359	0.019468
P83731	60S ribosomal protein L24	RPL24	1	4	4	24.8	17.779	1.17E+08	1.23E+08	1.03E+08	1.95E+08	1.96E+08	1.47E+08	1.56832	0.019468
Q15042	Rab3 GTPase-activating protein catalytic su	RAB3GAP1	1	9	9	12.9	110.52	50592000	47875000	43564000	35526000	39998000	32315000	0.759264	0.019641

P50991	T-complex protein 1 subunit delta	CCT4	1	23	23	52.1	57.924	2E+08	2.05E+08	2.04E+08	2.21E+08	2.28E+08	2.13E+08	1.085898	0.019661
P49756	RNA-binding protein 25	RBM25	1	5	5	9.6	100.18	63692000	62287000	77761000	43877000	50634000	48992000	0.704344	0.01981
Q9H7D7	WD repeat-containing protein 26	WDR26	1	9	9	19.5	72.123	45571000	47092000	48562000	40862000	42496000	36906000	0.851577	0.020208
P60981	Destrin	DSTN	1	10	10	55.8	18.506	4.36E+08	4.07E+08	4.12E+08	3.71E+08	3.83E+08	3.48E+08	0.877641	0.02026
P18859	ATP synthase-coupling factor 6, mitochonc	ATP5J	1	3	3	45.4	12.587	64125000	75733000	50153000	87266000	98713000	95034000	1.47893	0.020262
P62266	40S ribosomal protein S23	RPS23	1	5	5	42.7	15.807	31124000	39833000	NaN	61623000	52131000	59564000	1.628385	0.020311
Q00839	Heterogeneous nuclear ribonucleoprotein I	HNRNPU	1	18	18	23.9	90.583	2.99E+08	3.46E+08	2.97E+08	2.28E+08	2.43E+08	2.63E+08	0.779109	0.020612
Q5JWF2;P63092;P38405	Guanine nucleotide-binding protein G(s) su	GNAS	3	8	7	8.5	111.02	16243000	16365000	17203000	31847000	24122000	23625000	1.59792	0.020725
Q9Y2V2	Calcium-regulated heat stable protein 1	CARHSP1	1	4	4	59.2	15.892	54999000	63309000	51532000	70389000	68653000	70093000	1.231365	0.020741
P17858	ATP-dependent 6-phosphofructokinase, liv	PFKL	1	13	11	22.3	85.018	48992000	57168000	44357000	66236000	66863000	62149000	1.297182	0.020773
Q9NVA2;Q14141	Septin-11	11-Sep	2	16	14	47.3	49.398	2.18E+08	2.33E+08	2.52E+08	2.02E+08	1.92E+08	1.96E+08	0.837327	0.020893
Q8N5M4	Tetratricopeptide repeat protein 9C	TTC9C	1	3	3	16.4	20.012	13272000	13231000	NaN	16261000	16762000	15117000	1.210932	0.021162
P12270	Nucleoprotein TPR	TPR	1	23	23	14.3	267.29	2.57E+08	2.59E+08	2.8E+08	2.41E+08	2.17E+08	2.22E+08	0.85521	0.021318
P51659	Peroxisomal multifunctional enzyme type 2	HSD17B4	1	16	16	34	79.685	72182000	69778000	75800000	84384000	78313000	84208000	1.13384	0.021567
Q9Y263	Phospholipase A-2-activating protein	PLAA	1	11	11	15.6	87.156	40240000	36617000	37869000	33090000	33978000	34881000	0.88863	0.022662
P51911	Calponin-1	CNN1	1	6	6	25.3	33.17	40527000	43130000	41030000	46985000	44911000	49504000	1.13404	0.022822
P28799	Granulins;Acrogranin;Paragranulin;Granulin	GRN	1	7	7	19.6	63.544	47244000	58268000	46646000	62488000	72765000	68916000	1.341822	0.022867
O15144	Actin-related protein 2/3 complex subunit	ARPC2	1	14	14	48	34.333	2.25E+08	2.31E+08	2.18E+08	1.62E+08	1.99E+08	1.89E+08	0.815201	0.023053
P43034	Platelet-activating factor acetylhydrolase IB	PAFAH1B1	1	14	14	48.8	46.637	1.3E+08	1.14E+08	1.3E+08	1.02E+08	1.07E+08	1.07E+08	0.844698	0.02314
Q16186	Proteasomal ubiquitin receptor ADRM1	ADRM1	1	4	4	12.5	42.153	35241000	39411000	36087000	29192000	32368000	31885000	0.843831	0.023211
Q96KP4	Cytosolic non-specific dipeptidase	CNDP2	1	13	13	37.1	52.878	55431000	45523000	47033000	59014000	62978000	61278000	1.23842	0.023251
Q9NUJ1	Mycophenolic acid acyl-glucuronide estera	ABHD10	1	6	6	27.8	33.932	24485000	20157000	26144000	37838000	50171000	35381000	1.743141	0.023373
P54687	Branched-chain-amino-acid aminotransfer	BCAT1	1	8	8	30.1	42.966	27221000	28089000	29536000	33921000	44994000	42180000	1.427233	0.023511
P04792	Heat shock protein beta-1	HSPB1	1	15	15	74.1	22.782	1.36E+09	1.62E+09	1.31E+09	9.65E+08	1.07E+09	1.12E+09	0.734203	0.023573
P61981	14-3-3 protein gamma;14-3-3 protein gar	YWHAG	1	15	11	60.3	28.302	3.16E+08	4E+08	3.78E+08	2.66E+08	2.68E+08	2.85E+08	0.748791	0.023594
Q9NR30;Q9BQ39	Nucleolar RNA helicase 2	DDX21	2	5	5	8.7	87.343	33474000	NaN	34252000	21530000	27473000	23396000	0.712666	0.023717
Q03135;P56539	Caveolin-1	CAV1	2	8	8	42.1	20.471	8E+08	8.39E+08	9.56E+08	6.02E+08	5.94E+08	7.26E+08	0.740646	0.024049
O94919	Endonuclease domain-containing 1 proteir	ENDOD1	1	4	4	12	55.016	11378000	11847000	13053000	15070000	15406000	13630000	1.215778	0.024174
Q15155	Nodal modulator 1	NOMO1	1	17	1	19.8	134.32	4.72E+08	5.45E+08	4.67E+08	3.85E+08	4.07E+08	4.11E+08	0.810937	0.024371
O43143;Q14562	Pre-mRNA-splicing factor ATP-dependent	DHX15	2	14	13	22.3	90.932	57034000	52576000	62016000	47618000	45910000	39711000	0.776333	0.02442
P15121;C9JRZ8	Aldose reductase	AKR1B1	2	17	17	61.4	35.853	1.01E+09	8.79E+08	1.22E+09	6.38E+08	7.38E+08	5.74E+08	0.628456	0.024474
Q8NI22	Multiple coagulation factor deficiency prot	MCFD2	1	3	3	52.1	16.39	1.23E+08	1.27E+08	1.11E+08	68510000	93542000	94726000	0.712876	0.024525
O43237	Cytoplasmic dynein 1 light intermediate ch	DYNC1LI2	1	17	16	45.1	54.099	59292000	52466000	55518000	62481000	69788000	72327000	1.223104	0.024817
Q6NUK1	Calcium-binding mitochondrial carrier prot	SLC25A24	1	11	11	25.4	53.354	42354000	59542000	43707000	98554000	70754000	79859000	1.711277	0.024945
P18124	60S ribosomal protein L7	RPL7	1	9	9	28.6	29.225	2.36E+08	2.68E+08	2.34E+08	3E+08	2.81E+08	2.86E+08	1.175378	0.024967
Q14204	Cytoplasmic dynein 1 heavy chain 1	DYNC1H1	1	143	143	37.3	532.4	3.1E+08	3.28E+08	3.15E+08	3.46E+08	3.41E+08	3.68E+08	1.107283	0.025044
P39687;O95626;O43423	Acidic leucine-rich nuclear phosphoprotein	ANP32A	3	10	8	46.6	28.585	96718000	98546000	1.01E+08	80155000	88134000	91223000	0.876511	0.02538
O75874	Isocitrate dehydrogenase [NADP] cytoplasr	IDH1	1	17	16	50	46.659	1.7E+08	1.99E+08	2E+08	2.18E+08	2.37E+08	2.46E+08	1.233677	0.025416
Q14019	Coactosin-like protein	COTL1	1	7	7	50.7	15.945	2.39E+08	2.38E+08	2.66E+08	1.77E+08	1.95E+08	2.17E+08	0.792206	0.025962
Q14240	Eukaryotic initiation factor 4A-II;Eukaryotic	EIF4A2	1	19	9	50.1	46.402	96872000	74186000	1.05E+08	1.26E+08	1.35E+08	1.2E+08	1.381859	0.026071
O95372	Acyl-protein thioesterase 2	LYPLA2	1	2	2	11.3	24.737	69750000	80573000	79810000	56545000	61452000	66189000	0.800346	0.026485
P50897	Palmitoyl-protein thioesterase 1	PPT1	1	3	3	14.7	34.193	38826000	31099000	35663000	28224000	26091000	27325000	0.773194	0.026505
Q96FQ6	Protein S100-A16	S100A16	1	6	6	54.4	11.801	2.2E+08	2.48E+08	2.29E+08	2.09E+08	1.92E+08	1.98E+08	0.859269	0.026512
P17812	CTP synthase 1	CTPS1	1	14	13	36.9	66.69	96747000	1.08E+08	94527000	71570000	83220000	84777000	0.801807	0.026941
Q8N3C0	Activating signal cointegrator 1 complex su	ASCC3	1	11	11	7.3	251.46	14920000	15333000	15366000	14528000	14182000	14723000	0.952081	0.026981
Q99961;Q99962	Endophilin-A2	SH3GL1	2	5	5	20.1	41.489	30478000	38273000	40552000	24465000	26176000	26713000	0.707702	0.027063
Q96CW1	AP-2 complex subunit mu	AP2M1	1	9	9	29.7	49.654	97986000	82481000	80059000	69952000	64132000	66572000	0.770196	0.027192
P35613	Basigin	BSG	1	8	8	31.4	42.2	2.67E+08	2.56E+08	2.91E+08	2.2E+08	2.41E+08	2.1E+08	0.825705	0.027339
O00571;O15523;Q9NQI0	ATP-dependent RNA helicase DDX3X;ATP-	DDX3X;DDX3Y	3	22	21	40.3	73.243	1.57E+08	1.85E+08	1.68E+08	1.28E+08	1.35E+08	1.47E+08	0.802937	0.027877
P62910	60S ribosomal protein L32	RPL32	1	4	4	33.3	15.86	1.04E+08	1.46E+08	1.03E+08	1.91E+08	1.6E+08	1.71E+08	1.479333	0.028056
Q8IWB7	WD repeat and FYVE domain-containing p	WDFY1	1	8	8	24.6	46.323	22925000	22992000	20975000	27509000	25087000	24978000	1.15969	0.028185
O95394	Phosphoacetylglucosamine mutase	PGM3	1	9	9	25.8	59.851	49128000	54245000	46221000	40464000	42664000	36384000	0.798909	0.028204
P46976	Glycogenin-1	GYG1	1	5	5	16.9	39.383	1.82E+08	1.8E+08	1.88E+08	2.06E+08	2.26E+08	2E+08	1.148717	0.028248
P10768	S-formylglutathione hydrolase	ESD	1	6	6	31.2	31.462	76029000	92832000	87244000	1E+08	1.07E+08	1.03E+08	1.208879	0.028329
Q92614	Unconventional myosin-XVIIIa	MYO18A	1	18	18	12.4	233.11	32309000	28607000	33762000	35874000	39150000	37841000	1.192093	0.028386
Q5JSH3	WD repeat-containing protein 44	WDR44	1	9	9	12.7	101.37	15509000	13929000	18312000	11518000	10998000	12012000	0.723099	0.028499
Q9NVD7	Alpha-parvin	PARVA	1	9	8	27.7	42.243	58366000	54339000	68536000	43050000	48763000	41493000	0.735518	0.028528
Q9Y2B0	Protein canopy homolog 2	CNPY2	1	11	11	75.8	20.652	1.96E+08	1.8E+08	2.07E+08	1.41E+08	1.47E+08	1.72E+08	0.78891	0.028593
Q92597	Protein NDRG1	NDRG1	1	5	5	21.3	42.835	38617000	44306000	46888000	55599000	50892000	51800000	1.219396	0.028662

Q09666	Neuroblast differentiation-associated prote	AHNAK	1	211	211	59.9	629.09	5.9E+08	5.96E+08	5.87E+08	5.37E+08	5.71E+08	5.6E+08	0.941168	0.029027
Q9Y3E5	Peptidyl-tRNA hydrolase 2, mitochondrial	PTRH2	1	4	4	39.7	19.193	22817000	22119000	23938000	17944000	18297000	21171000	0.83358	0.029402
Q00341	Vigilin	HDLBP	1	29	29	31.2	141.45	1.01E+08	1.22E+08	1.06E+08	83644000	83584000	92615000	0.791288	0.029608
Q96K17	Transcription factor BTF3 homolog 4	BTF3L4	1	4	4	41.1	17.27	52086000	54330000	51419000	57667000	56888000	54996000	1.074229	0.030054
P54819	Adenylate kinase 2, mitochondrial;Adenylat	AK2	1	6	6	34.7	26.477	44885000	45009000	49792000	NaN	54716000	59442000	1.225871	0.030882
P40123	Adenylyl cyclase-associated protein 2	CAP2	1	13	12	31.2	52.823	40934000	40835000	41209000	53563000	49415000	44793000	1.201605	0.031093
Q9UHL4	Dipeptidyl peptidase 2	DPP7	1	3	3	8.9	54.341	15945000	16566000	15328000	19902000	25575000	20408000	1.377224	0.031198
Q9UBG0	C-type mannose receptor 2	MRC2	1	15	15	14.7	166.67	43429000	49372000	42193000	34211000	35414000	39206000	0.806191	0.031209
P07195	L-lactate dehydrogenase B chain	LDHB	1	13	12	38.3	36.638	4.2E+08	4.23E+08	4.78E+08	5.03E+08	4.96E+08	5.17E+08	1.146596	0.031218
Q03518	Antigen peptide transporter 1	TAP1	1	8	8	19.3	80.964	30161000	31699000	26253000	22888000	24961000	23329000	0.807804	0.031507
Q99436	Proteasome subunit beta type-7	PSMB7	1	4	4	18.1	29.965	88886000	77924000	1.1E+08	51521000	61060000	64772000	0.640079	0.031911
P46778	60S ribosomal protein L21	RPL21	1	3	3	21.2	18.565	NaN	1.12E+08	1.04E+08	1.97E+08	1.55E+08	1.63E+08	1.585421	0.031996
Q08211	ATP-dependent RNA helicase A	DHX9	1	27	27	29.1	140.96	1.16E+08	1.09E+08	1.15E+08	1.04E+08	92284000	1.02E+08	0.879769	0.032012
Q9Y6N5	Sulfide:quinone oxidoreductase, mitochonc	SQRDL	1	12	12	39.8	49.96	86283000	93364000	1.01E+08	1.04E+08	1.09E+08	1.12E+08	1.160962	0.032477
P15586	N-acetylglucosamine-6-sulfatase	GNS	1	8	8	19	62.081	75298000	83289000	75019000	92686000	93213000	1.11E+08	1.270169	0.03248
Q9Y383;Q9NQ29	Putative RNA-binding protein Luc7-like 2	LUC7L2	2	6	6	21.4	46.513	72975000	55718000	68606000	49698000	48584000	38407000	0.692801	0.032747
Q00325	Phosphate carrier protein, mitochondrial	SLC25A3	1	5	5	16	40.094	3.04E+08	2.46E+08	2.4E+08	3.64E+08	3.71E+08	3.15E+08	1.3273	0.032832
Q13501	Sequestosome-1	SQSTM1	1	12	12	46.6	47.687	1E+08	1.11E+08	1.09E+08	97710000	94640000	89382000	0.880027	0.032885
O00232	26S proteasome non-ATPase regulatory su	PSMD12	1	13	13	31.1	52.904	1.13E+08	1.09E+08	1.04E+08	96167000	1.01E+08	98027000	0.90806	0.032948
Q99613;B5ME19	Eukaryotic translation initiation factor 3 sub	EIF3C;EIF3CL	2	16	16	21.7	105.34	68756000	76717000	75554000	63186000	67315000	61385000	0.868156	0.033065
Q96AY3	Peptidyl-prolyl cis-trans isomerase FKBP10	FKBP10	1	12	12	25.6	64.244	1.32E+08	1.16E+08	1.14E+08	95732000	95695000	68742000	0.718937	0.033376
Q8NBS9	Thioredoxin domain-containing protein 5	TXNDC5	1	10	10	31	47.628	1.29E+08	1.26E+08	1.18E+08	1.09E+08	1.16E+08	1.08E+08	0.891971	0.03358
Q9H223	EH domain-containing protein 4	EHD4	1	12	9	29.6	61.174	41748000	42456000	41758000	39869000	34439000	34796000	0.866166	0.033653
P49589	Cysteine--tRNA ligase, cytoplasmic	CARS	1	18	18	29.1	85.472	50423000	49961000	56460000	42307000	43816000	46949000	0.848435	0.033888
Q9BUJ2	Heterogeneous nuclear ribonucleoprotein I	HNRNPUL1	1	9	9	12.3	95.737	30411000	30786000	27254000	21367000	24301000	26031000	0.810607	0.033935
P19474	E3 ubiquitin-protein ligase TRIM21	TRIM21	1	9	9	27.2	54.169	94570000	76462000	77613000	58392000	65365000	65266000	0.760212	0.034233
Q9UDT6	CAP-Gly domain-containing linker protein	CLIP2	1	13	11	17.9	115.84	18704000	19794000	21189000	21899000	25587000	25218000	1.218088	0.034376
O60506	Heterogeneous nuclear ribonucleoprotein (	SYNCRIP	1	18	15	36	69.602	1.54E+08	1.45E+08	1.55E+08	1.17E+08	1.31E+08	1.38E+08	0.849867	0.034427
Q13217	DnaJ homolog subfamily C member 3	DNAJC3	1	6	6	18.3	57.579	24826000	22818000	20382000	15455000	18952000	17996000	0.770338	0.034639
O43772	Mitochondrial carnitine/acylcarnitine carrie	SLC25A20	1	4	4	14.3	32.943	NaN	21822000	28025000	39261000	38930000	33486000	1.493597	0.034801
P12956	X-ray repair cross-complementing protein	XRCC6	1	19	19	39.2	69.842	1.38E+08	1.46E+08	1.4E+08	1.32E+08	1.33E+08	1.35E+08	0.944107	0.034803
P07305	Histone H1.0;Histone H1.0, N-terminally pr	H1F0	1	6	6	27.8	20.863	6.15E+08	6.52E+08	6.59E+08	4.57E+08	3.92E+08	5.7E+08	0.736985	0.034984
Q86UP2	Kinectin	KTN1	1	37	37	37.1	156.27	73366000	78430000	72778000	68189000	68591000	70081000	0.921126	0.035057
P61009	Signal peptidase complex subunit 3	SPCS3	1	3	3	17.8	20.313	27873000	24541000	NaN	34520000	34559000	31030000	1.273311	0.035164
Q8NBJ7	Sulfatase-modifying factor 2	SUMF2	1	8	8	32.2	33.843	28059000	26883000	29697000	22199000	21125000	25855000	0.817342	0.035277
O00487	26S proteasome non-ATPase regulatory su	PSMD14	1	4	4	19	34.577	29711000	29063000	28508000	37764000	32143000	33548000	1.185296	0.035306
P68371	Tubulin beta-4B chain	TUBB4B	1	21	1	63.4	49.83	2.24E+09	2.31E+09	2.11E+09	2.51E+09	2.42E+09	2.72E+09	1.150645	0.035317
Q96I99	Succinyl-CoA ligase [GDP-forming] subuni	SUCLG2	1	10	10	31	46.51	41595000	41798000	33112000	75150000	64096000	50157000	1.625707	0.035368
P61020	Ras-related protein Rab-5B	RAB5B	1	7	4	41.4	23.707	57805000	61180000	51686000	75626000	64025000	76481000	1.266366	0.036135
P04843	Dolichyl-diphosphooligosaccharide--prote	RPN1	1	17	17	34.6	68.569	2.99E+08	3.59E+08	3.28E+08	2.4E+08	2.74E+08	2.77E+08	0.803601	0.036278
Q9NQ88	Fructose-2,6-bisphosphatase TIGAR	TIGAR	1	4	4	25.9	30.062	39632000	49619000	47197000	35810000	36377000	36267000	0.794838	0.036316
P49189	4-trimethylaminobutyraldehyde dehydroge	ALDH9A1	1	17	17	40.7	53.801	1.04E+08	1.09E+08	1.07E+08	86633000	99237000	96333000	0.882022	0.036439
P49757	Protein numb homolog	NUMB	1	7	7	20	70.803	48970000	56174000	47535000	40715000	41204000	44111000	0.825457	0.036595
Q96KG9	N-terminal kinase-like protein	SCYL1	1	7	7	15.8	89.63	35169000	36493000	28932000	22550000	24188000	27842000	0.741396	0.036617
Q01130;Q9BRL6	Serine/arginine-rich splicing factor 2	SRSF2	2	6	6	28.5	25.476	83816000	76836000	78326000	72927000	73885000	71730000	0.914486	0.036949
Q96RF0	Sorting nexin-18	SNX18	1	5	5	10.4	68.894	56478000	58716000	56807000	39995000	49840000	50409000	0.815367	0.037411
P61978	Heterogeneous nuclear ribonucleoprotein I	HNRNPK	1	17	17	41	50.976	4.36E+08	4.46E+08	4.19E+08	3.45E+08	4.04E+08	3.78E+08	0.866756	0.037744
Q13838	Spliceosome RNA helicase DDX39B	DDX39B	1	12	5	33.9	48.991	1.25E+08	1.28E+08	1.31E+08	97480000	1.2E+08	1.03E+08	0.835393	0.037941
P67809	Nuclease-sensitive element-binding protei	YBX1	1	9	5	36.4	35.924	3.16E+08	2.54E+08	2.75E+08	2.23E+08	2.35E+08	2.11E+08	0.79116	0.037982
P12236	ADP/ATP translocase 3;ADP/ATP translocas	SLC25A6	1	8	3	25.2	32.866	1.89E+08	2.04E+08	1.84E+08	1.72E+08	1.75E+08	1.74E+08	0.903097	0.038183
P38919	Eukaryotic initiation factor 4A-III;Eukaryotic	EIF4A3	1	12	9	28.5	46.871	59143000	56371000	59903000	47109000	55092000	47342000	0.8525	0.038284
Q969H8	Myeloid-derived growth factor	MYDGF	1	3	3	25.4	18.795	2.62E+08	2.07E+08	2.28E+08	1.85E+08	1.8E+08	1.86E+08	0.790174	0.038361
P49327	Fatty acid synthase;[Acyl-carrier-protein] S	FASN	1	54	54	30.5	273.42	95531000	1.05E+08	94909000	77434000	82865000	90358000	0.849858	0.038551
Q9HB71	Calcyclin-binding protein	CACYBP	1	11	11	43.9	26.21	1.23E+08	99413000	1.17E+08	1.39E+08	1.34E+08	1.34E+08	1.197715	0.038718
Q92598	Heat shock protein 105 kDa	HSPH1	1	31	29	44.8	96.864	83915000	95843000	86985000	73224000	74156000	80640000	0.85483	0.039032
P00568;Q9Y6K8	Adenylate kinase isoenzyme 1	AK1	2	8	8	52.6	21.635	98740000	96973000	77505000	1.27E+08	1.1E+08	1.14E+08	1.285347	0.039592
Q99623	Prohibitin-2	PHB2	1	10	10	39.1	33.296	1.45E+08	2.08E+08	1.85E+08	2.6E+08	2.23E+08	2.47E+08	1.356989	0.039671
Q10713	Mitochondrial-processing peptidase subun	PMPCA	1	7	7	20.6	58.252	54926000	37403000	42994000	62731000	57800000	66159000	1.379588	0.03997
Q13263	Transcription intermediary factor 1-beta	TRIM28	1	18	18	40.6	88.549	64893000	59964000	70979000	50623000	51195000	58109000	0.816637	0.040002

Q9HDC9	Adipocyte plasma membrane-associated p	APMAP	1	11	11	38.5	46.48	98830000	1.1E+08	1.05E+08	83701000	79259000	96288000	0.825973	0.040021
Q9HA77	Probable cysteine--tRNA ligase, mitochonc	CARS2	1	5	5	14.9	62.223	16644000	13883000	23824000	31087000	34362000	25044000	1.664974	0.040307
P19623	Spermidine synthase	SRM	1	9	9	44	33.824	74000000	76004000	67833000	61008000	49500000	63240000	0.797606	0.04036
P39019	40S ribosomal protein S19	RPS19	1	8	8	44.1	16.06	2.76E+08	3.09E+08	2.64E+08	4.11E+08	3.7E+08	3.24E+08	1.301733	0.040531
P67870	Casein kinase II subunit beta	CSNK2B	1	6	6	40.9	24.942	65855000	67741000	64206000	52090000	48944000	61577000	0.82209	0.040601
P78371	T-complex protein 1 subunit beta	CCT2	1	18	18	44.7	57.488	1.79E+08	1.92E+08	1.74E+08	2.03E+08	2.19E+08	1.98E+08	1.137346	0.040612
O14558	Heat shock protein beta-6	HSPB6	1	7	7	76.9	17.135	4.64E+08	4.87E+08	3.7E+08	5.48E+08	6.68E+08	7.75E+08	1.507162	0.040629
Q9Y2Q3	Glutathione S-transferase kappa 1	GSTK1	1	5	5	30.1	25.497	80785000	71554000	69904000	81775000	93318000	94789000	1.214355	0.040736
P48059	LIM and senescent cell antigen-like-contair	LIMS1	1	6	4	23.7	37.251	63156000	47255000	43777000	31458000	34429000	34296000	0.649746	0.040788
P35080	Profilin-2	PFN2	1	4	4	30.7	15.046	1.79E+08	2.03E+08	2.29E+08	2.48E+08	2.52E+08	2.95E+08	1.3009	0.041015
P61221	ATP-binding cassette sub-family E membe	ABCE1	1	9	9	21.5	67.314	28354000	22983000	31897000	36063000	37249000	34280000	1.292645	0.041026
Q7Z3E5	LisH domain-containing protein ARMC9	ARMC9	1	8	8	13.9	91.818	41976000	73663000	67629000	33075000	31542000	31927000	0.526791	0.041041
Q9UHD9	Ubiquilin-2	UBQLN2	1	7	4	17	65.695	52100000	41168000	45494000	56510000	55186000	55605000	1.205669	0.041125
Q9BZF9	Uveal autoantigen with coiled-coil domains	UACA	1	11	11	10.8	162.5	31826000	25582000	32497000	24772000	21909000	21826000	0.761993	0.041332
Q9UEY8	Gamma-adducin	ADD3	1	5	5	7.6	79.154	14402000	13916000	13989000	14847000	14678000	15577000	1.066065	0.041504
O43809	Cleavage and polyadenylation specificity fa	NUDT21	1	3	3	13.2	26.227	36157000	46764000	41913000	26913000	27444000	34897000	0.714981	0.041506
Q13418	Integrin-linked protein kinase	ILK	1	9	9	26.8	51.419	1.01E+08	1.02E+08	81118000	56908000	75696000	72278000	0.721319	0.041656
Q15005	Signal peptidase complex subunit 2	SPCS2	1	6	6	39.4	25.003	1.09E+08	88923000	84737000	70808000	70837000	73642000	0.761	0.041734
P21912	Succinate dehydrogenase [ubiquinone] iror	SDHB	1	6	6	25	31.629	25626000	29895000	29668000	31563000	37499000	37291000	1.248436	0.041828
Q32P28	Prolyl 3-hydroxylase 1	LEPRE1	1	11	10	21.2	83.393	40090000	45934000	35215000	30243000	32222000	30836000	0.769563	0.04184
P10619	Lysosomal protective protein;Lysosomal pr	CTSA	1	5	5	11.5	54.465	1.2E+08	1.24E+08	1.22E+08	1.41E+08	1.53E+08	1.3E+08	1.163478	0.042119
O00267	Transcription elongation factor SPT5	SUPT5H	1	6	6	9.4	121	18590000	21817000	17063000	9774500	13296000	NaN	0.602153	0.042149
Q9UK76	Hematological and neurological expressed	HN1	1	5	5	64.3	16.014	52463000	51005000	42185000	56932000	66446000	73732000	1.353285	0.042261
P07910;P0DMR1;O60812;B7Z\	Heterogeneous nuclear ribonucleoproteins	HNRNPC;HNRNPC	6	14	14	41.5	33.67	1.93E+08	2.02E+08	2.09E+08	1.73E+08	1.73E+08	1.91E+08	0.887979	0.042319
Q13185	Chromobox protein homolog 3	CBX3	1	5	3	36.6	20.811	54564000	44709000	57523000	34032000	43264000	38081000	0.735841	0.042642
Q9Y678	Coatomer subunit gamma-1	COPG1	1	25	23	39.4	97.717	1.34E+08	1.66E+08	1.38E+08	1.16E+08	1.05E+08	1.2E+08	0.778593	0.042824
O75131;Q96FN4;Q8IYJ1;Q96A	Copine-3	CPNE3	7	10	9	24	60.13	62050000	68906000	58103000	96881000	85877000	72994000	1.352763	0.042857
P01033	Metalloproteinase inhibitor 1	TIMP1	1	4	4	25.1	23.171	32859000	30522000	28357000	54972000	46588000	37057000	1.51101	0.042877
Q9H2U2	Inorganic pyrophosphatase 2, mitochondri	PPA2	1	8	8	33.2	37.92	28477000	33825000	36708000	44035000	42514000	56380000	1.443581	0.043131
P62249	40S ribosomal protein S16	RPS16	1	8	8	43.8	16.445	1.9E+08	1.67E+08	1.67E+08	2.4E+08	2.46E+08	1.95E+08	1.300732	0.043435
P15170	Eukaryotic peptide chain release factor GTF	GSPT1	1	14	7	35.7	55.755	84105000	1.01E+08	95781000	70439000	74412000	82474000	0.809256	0.04344
Q16204	Coiled-coil domain-containing protein 6	CCDC6	1	5	5	14.8	53.29	21156000	NaN	18528000	26280000	23619000	27476000	1.299852	0.043547
P02511	Alpha-crystallin B chain	CRYAB	1	6	6	36	20.159	86583000	1.04E+08	87401000	71681000	69054000	80092000	0.794988	0.043588
Q14195	Dihydropyrimidinase-related protein 3	DPYSL3	1	16	14	50.4	61.963	53468000	45241000	54716000	38379000	43716000	42008000	0.808884	0.043842
P46060	Ran GTPase-activating protein 1	RANGAP1	1	13	13	30.2	63.541	54547000	56298000	64072000	45840000	46826000	51924000	0.826621	0.043966
Q9UIA9	Exportin-7	XPO7	1	5	5	6.2	123.91	NaN	20778000	17364000	15249000	14136000	13651000	0.752207	0.044109
P07339	Cathepsin D;Cathepsin D light chain;Cathep	CTSD	1	11	11	32	44.552	3.15E+08	3.04E+08	3.56E+08	2.51E+08	2.84E+08	2.78E+08	0.83413	0.044141
Q9BRF8	Serine/threonine-protein phosphatase CPP	CPPED1	1	4	4	20.4	35.548	32798000	33799000	36837000	21118000	29312000	28256000	0.760736	0.044163
Q8IV08	Phospholipase D3	PLD3	1	5	5	15.3	54.705	50079000	40200000	34322000	27364000	29395000	18558000	0.604465	0.044331
P24539	ATP synthase F(0) complex subunit B1, mitc	ATP5F1	1	11	11	41.8	28.908	1.27E+08	1.39E+08	1.59E+08	1.72E+08	1.63E+08	1.74E+08	1.198719	0.044643
Q13310	Polyadenylate-binding protein 4	PABPC4	1	10	5	21	70.782	28876000	21352000	29541000	41215000	32146000	39693000	1.417267	0.044645
P17655	Calpain-2 catalytic subunit	CAPN2	1	26	26	50	79.994	3.08E+08	3.29E+08	3.57E+08	2.61E+08	2.76E+08	3E+08	0.841853	0.045006
P50395	Rab GDP dissociation inhibitor beta	GDI2	1	29	23	68.5	50.663	2.08E+08	2.46E+08	2.24E+08	1.9E+08	1.98E+08	1.94E+08	0.858373	0.046413
P16989;Q9Y2T7	Y-box-binding protein 3	YBX3	2	9	5	34.7	40.089	40026000	29792000	45131000	24582000	27487000	19610000	0.623572	0.046435
P57764	Gasdermin-D	GSDMD	1	5	5	17.1	52.8	18392000	13559000	13312000	19019000	20334000	22539000	1.367386	0.046512
O15460	Prolyl 4-hydroxylase subunit alpha-2	P4HA2	1	5	5	16.6	60.901	29051000	26201000	31308000	25384000	24218000	23659000	0.846361	0.047005
P61604	10 kDa heat shock protein, mitochondrial	HSPE1	1	6	6	54.9	10.932	2.9E+08	2.71E+08	2.34E+08	6.83E+08	5.08E+08	3.75E+08	1.967656	0.047073
P35606	Coatomer subunit beta	COPB2	1	28	28	42.8	102.49	2.14E+08	2.37E+08	2.17E+08	1.87E+08	1.9E+08	2.08E+08	0.875093	0.047297
P62701;P22090;Q8TD47	40S ribosomal protein S4, X isoform;40S rib	RPS4X;RPS4Y1	3	12	12	47.5	29.597	1.92E+08	2.45E+08	1.86E+08	3.07E+08	2.57E+08	2.66E+08	1.330526	0.047379
Q92734	Protein TFG	TFG	1	10	10	33.2	43.447	3.24E+08	3.41E+08	3.2E+08	2.09E+08	2.58E+08	2.97E+08	0.775811	0.048283
P62857	40S ribosomal protein S28	RPS28	1	2	2	30.4	7.8409	2.95E+08	3.34E+08	3.29E+08	2.63E+08	2.58E+08	2.95E+08	0.851689	0.048374
Q04323	UBX domain-containing protein 1	UBXN1	1	4	4	18.9	33.325	67932000	60009000	50650000	42689000	47403000	44909000	0.755923	0.048479
P08670;P41219;CON__Q9DCV	Vimentin	VIM	16	48	43	78.1	53.651	6.98E+09	7.07E+09	7.67E+09	6.23E+09	6.29E+09	6.81E+09	0.889448	0.048732
P27635	60S ribosomal protein L10	RPL10	1	7	7	28.5	24.577	2.52E+08	2.6E+08	2.23E+08	1.81E+08	1.75E+08	2.23E+08	0.787246	0.048904
Q99615	DnaJ homolog subfamily C member 7	DNAJC7	1	10	10	28.5	56.44	43757000	43546000	38879000	38673000	34345000	36152000	0.865179	0.048947
P46063	ATP-dependent DNA helicase Q1	RECQL	1	8	8	22.7	73.457	39840000	34806000	33659000	28169000	28580000	32257000	0.821809	0.048994
P38159;Q96E39;O75526;Q8N7	RNA-binding motif protein, X chromosome	RBMX;RBMXL1	4	8	8	19.9	42.331	1.49E+08	1.14E+08	1.2E+08	86238000	90014000	1.05E+08	0.733243	0.049153
Q16658	Fascin	FSCN1	1	24	24	54.8	54.529	5.02E+08	5.09E+08	4.5E+08	3.81E+08	4.32E+08	4.35E+08	0.854341	0.049195
Q9NUY8	TBC1 domain family member 23	TBC1D23	1	6	6	14.3	78.321	23418000	27693000	23958000	16711000	16971000	22341000	0.746287	0.049249

P23588	Eukaryotic translation initiation factor 4B	EIF4B	1	10	10	25	69.15	54494000	63394000	60741000	47168000	49320000	54138000	0.843234	0.049388
P08237	ATP-dependent 6-phosphofructokinase, m	PFKM	1	11	10	21.7	85.182	33044000	46595000	52077000	69808000	63054000	56254000	1.435786	0.049724
Q8NCW5	NAD(P)H-hydrate epimerase	APOA1BP	1	6	6	34.4	31.674	35817000	36456000	30263000	38844000	44227000	49087000	1.288894	0.049834
Q9NX55	Huntingtin-interacting protein K	HYPK	1	4	4	54.5	13.651	17115000	NaN	18601000	13804000	12069000	NaN	0.724409	0.049868
Q6DD88	Atlastin-3	ATL3	1	10	10	34.8	60.541	2.02E+08	2.26E+08	1.97E+08	1.78E+08	1.79E+08	1.88E+08	0.872333	0.050625
Q9H8S9;Q7L9L4	MOB kinase activator 1A;MOB kinase activa	MOB1A;MOB1B	2	4	4	22.7	25.079	46808000	57164000	55755000	69508000	64544000	60315000	1.21687	0.051141
P41252	Isoleucine--tRNA ligase, cytoplasmic	IARS	1	23	23	24.7	144.5	1.27E+08	1.16E+08	1.28E+08	1.07E+08	1.15E+08	1.13E+08	0.902947	0.051544
O43396	Thioredoxin-like protein 1	TXNL1	1	7	7	44.6	32.251	68418000	75030000	78644000	60920000	66475000	51917000	0.807377	0.051554
Q9P016	Thymocyte nuclear protein 1	THYN1	1	3	3	17.3	25.697	NaN	20813000	20407000	16112000	14834000	18339000	0.797105	0.051568
O60568	Procollagen-lysine,2-oxoglutarate 5-dioxy	PLOD3	1	12	12	20.6	84.784	42392000	47936000	45979000	36066000	37812000	42251000	0.851967	0.051889
P34931	Heat shock 70 kDa protein 1-like	HSPA1L	1	13	2	28.1	70.374	79422000	54599000	1.13E+08	17917000	23541000	47437000	0.359388	0.051912
P53992	Protein transport protein Sec24C	SEC24C	1	11	11	16.3	118.32	38321000	36749000	35843000	31693000	35432000	33071000	0.903375	0.052597
Q15366	Poly(rC)-binding protein 2	PCBP2	1	8	4	31.2	38.58	89185000	69960000	68405000	1.52E+08	1.16E+08	98655000	1.613162	0.052602
P35221;P26232;Q9UI47	Catenin alpha-1	CTNNA1	3	22	22	36.8	100.07	45158000	49942000	40411000	38720000	37056000	36640000	0.829571	0.052647
Q99878;Q96KK5;Q9BTM1;P20(	Histone H2A type 1-J;Histone H2A type 1-	HIST1H2AJ;HIST1H	11	5	2	58.6	13.936	3.9E+08	3.92E+08	2.77E+08	6.1E+08	4.54E+08	4.9E+08	1.46767	0.052672
P78330	Phosphoserine phosphatase	PSPH	1	4	4	19.1	25.007	29899000	25442000	26378000	43118000	31415000	36945000	1.364163	0.052795
Q02878	60S ribosomal protein L6	RPL6	1	12	12	26	32.728	1.9E+08	1.75E+08	2.2E+08	2.41E+08	2.26E+08	2.73E+08	1.265268	0.052882
Q15334	Lethal(2) giant larvae protein homolog 1	LLGL1	1	9	9	13.1	115.42	12939000	9971100	13729000	23551000	19043000	15410000	1.583117	0.052961
Q14847	LIM and SH3 domain protein 1	LASP1	1	12	12	42.9	29.717	5.36E+08	5.42E+08	4.85E+08	4.21E+08	4.28E+08	4.88E+08	0.855391	0.052971
P22694	cAMP-dependent protein kinase catalytic s	PRKACB	1	7	2	22.5	40.622	71325000	59430000	70729000	84412000	82222000	74628000	1.197425	0.052981
Q14498	RNA-binding protein 39	RBM39	1	6	6	15.5	59.379	23331000	22782000	24736000	20086000	18808000	22323000	0.864049	0.053006
P13674	Prolyl 4-hydroxylase subunit alpha-1	P4HA1	1	10	10	24.2	61.049	38072000	27568000	37779000	26074000	24009000	24793000	0.724006	0.053314
Q92729	Receptor-type tyrosine-protein phosphata	PTPRU	1	2	2	1.5	162.42	49814000	33274000	50547000	NaN	20435000	23162000	0.489359	0.053534
Q12888	Tumor suppressor p53-binding protein 1	TP53BP1	1	11	11	9.5	213.57	21941000	20593000	22391000	15941000	20103000	14290000	0.775264	0.05498
P08133	Annexin A6	ANXA6	1	40	40	61.4	75.872	5.75E+08	5.95E+08	5.14E+08	4.84E+08	4.84E+08	5.09E+08	0.876849	0.055035
P19404	NADH dehydrogenase [ubiquinone] flavop	NDUFV2	1	4	4	21.7	27.391	10784000	13109000	16969000	23360000	17618000	19669000	1.484191	0.055482
P30837	Aldehyde dehydrogenase X, mitochondrial	ALDH1B1	1	6	5	15.3	57.249	NaN	12165000	9768800	13795000	14502000	13495000	1.270247	0.05555
Q00535	Cyclin-dependent-like kinase 5	CDK5	1	3	2	13	33.304	NaN	11182000	11919000	NaN	9799800	9059500	0.816385	0.055637
Q8NE71	ATP-binding cassette sub-family F membe	ABCF1	1	13	13	17.8	95.925	23794000	32117000	29233000	37797000	34733000	34139000	1.252807	0.05599
P78344	Eukaryotic translation initiation factor 4 gar	EIF4G2	1	12	12	17.1	102.36	34027000	34772000	30621000	25306000	30391000	28155000	0.843412	0.056224
Q16270	Insulin-like growth factor-binding protein 7	IGFBP7	1	7	7	39.4	29.13	68909000	56514000	59828000	41413000	51334000	52123000	0.78202	0.056377
Q16850	Lanosterol 14-alpha demethylase	CYP51A1	1	5	5	12.4	57.278	14538000	10422000	15719000	16456000	21110000	20235000	1.420905	0.056612
P25789	Proteasome subunit alpha type-4	PSMA4	1	5	5	25.7	29.483	1.88E+08	1.97E+08	2.55E+08	1.17E+08	1.39E+08	1.74E+08	0.670314	0.057484
P16298	Serine/threonine-protein phosphatase 2B c	PPP3CB	1	8	4	16	59.024	43906000	36456000	35058000	47332000	58736000	46942000	1.32568	0.057496
O75844	CAAX prenyl protease 1 homolog	ZMPSTE24	1	3	3	10.5	54.812	30466000	34645000	36923000	20459000	23219000	30448000	0.726483	0.057759
Q86XZ4	Spermatogenesis-associated serine-rich pr	SPATS2	1	4	4	10.1	59.544	10699000	9647400	9262500	8987300	7663800	8419200	0.846715	0.058273
Q9BS26	Endoplasmic reticulum resident protein 44	ERP44	1	11	11	40.9	46.971	1.75E+08	1.44E+08	1.64E+08	1.31E+08	1.43E+08	1.26E+08	0.827959	0.058417
Q9H488	GDP-fucose protein O-fucosyltransferase 1	POFUT1	1	6	6	22.9	43.955	44695000	51161000	48168000	44408000	41864000	40639000	0.88118	0.058497
Q16610	Extracellular matrix protein 1	ECM1	1	8	8	21.9	60.673	31503000	31654000	31122000	30055000	27931000	25226000	0.882614	0.058604
P04075	Fructose-bisphosphate aldolase A	ALDOA	1	21	19	70.6	39.42	1.3E+09	1.47E+09	1.5E+09	1.01E+09	1.03E+09	1.31E+09	0.786158	0.058659
Q9NS69	Mitochondrial import receptor subunit TOM	TOMM22	1	2	2	16.2	15.521	22908000	27837000	17980000	28371000	32661000	32782000	1.365064	0.058896
P21281;P15313	V-type proton ATPase subunit B, brain isof	ATP6V1B2	2	12	12	30.7	56.5	57382000	57031000	66591000	67986000	69130000	76961000	1.18272	0.059005
P26373	60S ribosomal protein L13	RPL13	1	6	6	29.4	24.261	2.12E+08	2.38E+08	2.04E+08	6.68E+08	4.6E+08	3.21E+08	2.215494	0.059244
P10321	HLA class I histocompatibility antigen, Cw-7	HLA-C	1	10	5	40.2	40.648	1.54E+08	2.06E+08	2.12E+08	1.13E+08	1.3E+08	1.55E+08	0.696551	0.059349
Q9H8Y8	Golgi reassembly-stacking protein 2	GORASP2	1	3	3	10	47.145	14876000	17209000	14599000	19497000	17584000	17682000	1.173057	0.059982
P45973	Chromobox protein homolog 5	CBX5	1	3	2	20.9	22.225	18346000	22869000	NaN	15908000	15028000	15684000	0.754094	0.060186
Q9Y2D5	A-kinase anchor protein 2	AKAP2	1	22	22	35.4	94.659	1.12E+08	1.16E+08	1.24E+08	1.24E+08	1.46E+08	1.5E+08	1.196214	0.060798
P26885	Peptidyl-prolyl cis-trans isomerase FKBP2	FKBP2	1	3	3	26.1	15.649	88777000	99444000	1.07E+08	65373000	82706000	84591000	0.788843	0.061114
P36507	Dual specificity mitogen-activated protein I	MAP2K2	1	13	10	34.8	44.424	56427000	63674000	61455000	46439000	49405000	56759000	0.840529	0.061405
O14561	Acyl carrier protein, mitochondrial	NDUFAB1	1	3	3	15.4	17.417	68808000	73426000	70530000	81134000	81033000	99705000	1.23081	0.061514
Q9NVJ2	ADP-ribosylation factor-like protein 8B	ARL8B	1	7	3	45.2	21.539	45998000	33632000	35058000	48829000	47403000	57891000	1.343846	0.061601
O94979	Protein transport protein Sec31A	SEC31A	1	24	24	28.1	133.01	1.02E+08	1.13E+08	1.15E+08	92129000	1.02E+08	98859000	0.886764	0.061875
P13010	X-ray repair cross-complementing protein	XRCC5	1	17	17	39.3	82.704	2.21E+08	1.99E+08	2.16E+08	1.91E+08	1.88E+08	1.99E+08	0.909363	0.06191
P11279	Lysosome-associated membrane glycoprot	LAMP1	1	3	3	8.2	44.882	61024000	1.35E+08	NaN	2.68E+08	1.95E+08	1.95E+08	2.238788	0.062078
Q9Y2Z0	Suppressor of G2 allele of SKP1 homolog	SUGT1	1	12	12	41.4	41.024	59295000	61013000	58874000	47778000	57346000	52206000	0.878046	0.062378
P49441	Inositol polyphosphate 1-phosphatase	INPP1	1	5	5	20.3	43.998	20525000	19120000	24069000	18456000	16430000	16317000	0.803638	0.062552
P36542	ATP synthase subunit gamma, mitochondri	ATP5C1	1	6	6	22.1	32.996	1.1E+08	1.11E+08	87822000	1.62E+08	1.29E+08	1.24E+08	1.34668	0.062669
Q9UBQ5	Eukaryotic translation initiation factor 3 sub	EIF3K	1	5	5	27.5	25.059	73846000	75224000	62875000	62152000	59693000	59402000	0.855161	0.062894
Q9Y3I0	tRNA-splicing ligase RtcB homolog	RTCB	1	17	17	41.6	55.21	1.29E+08	1.27E+08	1.31E+08	1.4E+08	1.36E+08	1.54E+08	1.11031	0.06311

Q9H832	Ubiquitin-conjugating enzyme E2 Z	UBE2Z	1	7	7	24	38.21	25473000	29190000	NaN	36878000	38037000	31761000	1.301014	0.06311
P62888	60S ribosomal protein L30	RPL30	1	5	5	54.8	12.784	3.03E+08	3.23E+08	2.91E+08	2.84E+08	2.78E+08	2.83E+08	0.921475	0.063179
P53701	Cytochrome c-type heme lyase	HCCS	1	4	4	18.3	30.601	NaN	9795900	11486000	13601000	16651000	13858000	1.381769	0.06341
Q9UPY8	Microtubule-associated protein RP/EB fami	MAPRE3	1	5	3	22.1	31.982	15001000	17685000	17270000	12503000	11983000	15315000	0.796721	0.063538
P05198	Eukaryotic translation initiation factor 2 sub	EIF2S1	1	13	13	47	36.112	89927000	1.01E+08	1.03E+08	79549000	77706000	91498000	0.845331	0.063843
Q8TAT6	Nuclear protein localization protein 4 hom	NPLOC4	1	9	9	20.1	68.119	1.21E+08	1.15E+08	1.11E+08	88995000	1.09E+08	99450000	0.859143	0.064326
Q96AG4	Leucine-rich repeat-containing protein 59	LRRC59	1	11	11	40.4	34.93	1.06E+08	1.11E+08	1.03E+08	87673000	89378000	1.03E+08	0.875794	0.06504
Q01081;Q8WU68	Splicing factor U2AF 35 kDa subunit;Splicin	U2AF1;U2AF1L4	2	4	4	21.7	27.872	62939000	82220000	70997000	46783000	55564000	61103000	0.756167	0.065418
P09525	Annexin A4	ANXA4	1	14	14	50.2	35.882	1.38E+08	1.28E+08	1.38E+08	2.06E+08	1.81E+08	1.47E+08	1.324336	0.065807
P31939	Bifunctional purine biosynthesis protein PU	ATIC	1	20	20	51.9	64.615	73002000	77691000	87762000	88880000	91493000	1.01E+08	1.179397	0.065818
O15118	Niemann-Pick C1 protein	NPC1	1	6	6	6.6	142.17	21047000	29186000	28013000	30582000	34336000	36669000	1.298303	0.065945
Q9BZQ8	Protein Niban	FAM129A	1	15	15	21.8	103.13	49943000	53184000	52834000	57582000	61059000	72759000	1.22723	0.06597
O15355	Protein phosphatase 1G	PPM1G	1	8	8	20.5	59.271	36305000	36531000	36778000	25181000	28570000	34754000	0.807424	0.066214
Q9UJU6	Drebrin-like protein	DBNL	1	9	9	29.3	48.207	45302000	43659000	40349000	34326000	34156000	40888000	0.845797	0.06632
Q96CT7	Coiled-coil domain-containing protein 124	CCDC124	1	5	5	26.5	25.835	34142000	32645000	30341000	41038000	35707000	47663000	1.280866	0.066433
Q13151	Heterogeneous nuclear ribonucleoprotein ,	HNRNPA0	1	5	5	25.9	30.84	37344000	44885000	31988000	24090000	31455000	26210000	0.715787	0.067011
P12235	ADP/ATP translocase 1	SLC25A4	1	6	3	22.8	33.064	24522000	24353000	28054000	32825000	31847000	27494000	1.198066	0.067046
P30044	Peroxiredoxin-5, mitochondrial	PRDX5	1	5	5	33.2	22.086	1.09E+08	91765000	89771000	1.89E+08	1.46E+08	1.18E+08	1.559712	0.067467
P61006;P59190;O95716;Q96E	Ras-related protein Rab-8A	RAB8A	4	7	4	36.2	23.668	51800000	55576000	60335000	49436000	50466000	49162000	0.888815	0.067826
Q9UN37	Vacuolar protein sorting-associated protei	VPS4A	1	4	3	13.3	48.897	13488000	12867000	NaN	18646000	22722000	NaN	1.569645	0.067837
Q13630	GDP-L-fucose synthase	TSTA3	1	3	3	15.9	35.892	22698000	16878000	21590000	17664000	12775000	11988000	0.693637	0.068095
P78527	DNA-dependent protein kinase catalytic su	PRKDC	1	50	50	16.4	469.08	1.65E+08	1.6E+08	1.87E+08	1.47E+08	1.55E+08	1.42E+08	0.866383	0.068185
Q16864	V-type proton ATPase subunit F	ATP6V1F	1	3	3	33.6	13.37	25562000	19051000	22627000	25752000	28774000	28402000	1.233314	0.068276
O95183	Vesicle-associated membrane protein 5	VAMP5	1	2	2	17.2	12.805	15524000	14975000	NaN	20040000	25386000	19896000	1.42785	0.068594
O76094	Signal recognition particle subunit SRP72	SRP72	1	14	14	27.3	74.605	72105000	92771000	91984000	57214000	64965000	73518000	0.761882	0.068617
P06753	Tropomyosin alpha-3 chain	TPM3	1	23	11	58.2	32.95	1.78E+09	1.56E+09	1.85E+09	1.32E+09	1.59E+09	1.17E+09	0.785973	0.068654
P27449	V-type proton ATPase 16 kDa proteolipid s	ATP6V0C	1	1	1	11.6	15.736	27258000	33281000	25077000	89682000	39162000	66604000	2.282844	0.068756
P04062	Glucosylceramidase	GBA	1	11	11	24.6	59.716	75011000	79723000	76337000	80004000	91857000	86704000	1.118985	0.068783
P46776	60S ribosomal protein L27a	RPL27A	1	3	3	22.3	16.561	NaN	1.39E+08	1.57E+08	3.03E+08	2.64E+08	2.01E+08	1.727905	0.069005
P56945	Breast cancer anti-estrogen resistance prot	BCAR1	1	4	4	7.6	93.371	14610000	14036000	10699000	9143600	10949000	7246900	0.694866	0.06909
P43686	26S protease regulatory subunit 6B	PSMC4	1	16	16	47.4	47.366	1.33E+08	1.42E+08	1.33E+08	1.26E+08	1.23E+08	1.31E+08	0.931125	0.069615
Q9UNL2	Translocon-associated protein subunit gam	SSR3	1	1	1	7.6	21.08	1.1E+08	81708000	76736000	64715000	67705000	53724000	0.693884	0.069941
Q12797	Aspartyl/asparaginyl beta-hydroxylase	ASPH	1	15	15	28.2	85.862	1.73E+08	1.77E+08	1.69E+08	1.5E+08	1.59E+08	1.68E+08	0.917309	0.070089
Q9NQR4	Omega-amidase NIT2	NIT2	1	7	7	37.7	30.608	24191000	23439000	22319000	24722000	27780000	30633000	1.188509	0.070161
O60716	Catenin delta-1	CTNND1	1	19	19	31.2	108.17	45848000	46439000	53240000	40926000	44038000	41826000	0.871247	0.070172
Q96L92	Sorting nexin-27	SNX27	1	9	9	20.1	61.264	31700000	34214000	29632000	35233000	35617000	40501000	1.165418	0.070571
Q8TBC4	NEDD8-activating enzyme E1 catalytic subu	UBA3	1	9	9	29.2	51.852	27496000	26272000	22967000	30180000	30399000	27809000	1.15186	0.07058
Q16555;Q14194	Dihydropyrimidinase-related protein 2	DPYSL2	2	17	15	50.7	62.293	1.77E+08	1.91E+08	1.79E+08	1.97E+08	1.96E+08	2.19E+08	1.11763	0.070933
Q27J81	Inverted formin-2	INF2	1	18	18	23.4	135.62	38014000	42442000	41567000	35276000	32347000	38336000	0.868353	0.071338
Q9BSD7	Cancer-related nucleoside-triphosphatase	NTPCR	1	4	4	31.6	20.713	27679000	22347000	19133000	31038000	31861000	27243000	1.303402	0.071394
Q13564	NEDD8-activating enzyme E1 regulatory su	NAE1	1	12	12	33.5	60.246	49525000	56067000	53971000	49849000	47282000	46589000	0.90071	0.071582
Q93052	Lipoma-preferred partner	LPP	1	13	13	36.4	65.746	62319000	60054000	61296000	50053000	51539000	59689000	0.878107	0.071668
O15143	Actin-related protein 2/3 complex subunit	ARPC1B	1	11	10	44.1	40.949	2.08E+08	1.86E+08	1.95E+08	1.73E+08	1.76E+08	1.36E+08	0.821665	0.0717
Q96C23	Aldose 1-epimerase	GALM	1	4	4	17	37.765	20691000	15815000	19817000	26831000	24801000	21099000	1.29132	0.072033
Q16851	UTP--glucose-1-phosphate uridylyltransfe	UGP2	1	12	12	31.5	56.94	68252000	76201000	68905000	61738000	66615000	63775000	0.900496	0.072097
Q15019	Septin-2	2-Sep	1	16	16	57.3	41.487	2.48E+08	2.37E+08	2.06E+08	1.92E+08	2.03E+08	2E+08	0.862046	0.072143
P18669;P15259;Q8N0Y7	Phosphoglycerate mutase 1	PGAM1	3	17	17	70.9	28.804	6.03E+08	5.6E+08	6.5E+08	5.1E+08	5.41E+08	5.52E+08	0.884392	0.072196
Q9UKM9	RNA-binding protein Raly	RALY	1	3	3	13.1	32.463	34996000	30618000	38045000	26599000	31179000	26817000	0.816089	0.072425
Q68CZ2;Q63HR2	Tensin-3	TNS3	2	23	22	26	155.26	56058000	52843000	62483000	51223000	50373000	48130000	0.873629	0.072571
O60443	Non-syndromic hearing impairment protei	DFNA5	1	10	10	25.4	54.554	46416000	44826000	47483000	49871000	55389000	64133000	1.22107	0.072716
P84103	Serine/arginine-rich splicing factor 3	SRSF3	1	3	2	17.7	19.329	1.13E+08	1.68E+08	1.5E+08	72799000	1.04E+08	1.09E+08	0.665424	0.072799
O00469	Procollagen-lysine,2-oxoglutarate 5-dioxy	PLOD2	1	8	8	13.8	84.685	37917000	37058000	35334000	30036000	35612000	31106000	0.877118	0.073028
Q6NVY1	3-hydroxyisobutyryl-CoA hydrolase, mitoc	HIBCH	1	6	6	17.4	43.482	17153000	19964000	21393000	21263000	24964000	25035000	1.217946	0.073309
Q8N766	ER membrane protein complex subunit 1	EMC1	1	6	6	10.4	111.76	27267000	32791000	26473000	21853000	22736000	25510000	0.810103	0.073486
O14617	AP-3 complex subunit delta-1	AP3D1	1	17	17	19.9	130.16	36023000	41485000	38309000	32824000	34930000	35341000	0.890154	0.074163
Q00688	Peptidyl-prolyl cis-trans isomerase FKBP3	FKBP3	1	6	6	26.3	25.177	53582000	54276000	58940000	68690000	64118000	87918000	1.323313	0.074184
P50995;P27216	Annexin A11	ANXA11	2	12	12	26.1	54.389	1.33E+08	1.52E+08	1.41E+08	1.2E+08	1.32E+08	1.27E+08	0.888664	0.074257
P48444	Coatomer subunit delta	ARCN1	1	17	17	36.8	57.21	93199000	94598000	86704000	87542000	82316000	77649000	0.901662	0.074513
Q08AM6	Protein VAC14 homolog	VAC14	1	4	4	7.4	87.972	12876000	18949000	14009000	22991000	20753000	18462000	1.357202	0.074634

Q9Y5K6	CD2-associated protein	CD2AP	1	4	4	11.6	71.45	16229000	15402000	14831000	14669000	10305000	11859000	0.792755	0.074736
Q14444	Caprin-1	CAPRIN1	1	7	7	12.4	78.365	87837000	84431000	83099000	74540000	81055000	81115000	0.92694	0.074869
Q6UXV4	MICOS complex subunit MIC27	APOOL	1	5	5	29.9	29.159	13461000	13334000	12272000	15586000	14219000	13878000	1.118156	0.075161
P30536	Translocator protein	TSPO	1	2	2	9.5	18.828	1.77E+08	2.11E+08	1.64E+08	2.22E+08	2.15E+08	2.16E+08	1.182317	0.075267
Q9NZ08	Endoplasmic reticulum aminopeptidase 1	ERAP1	1	4	4	5.7	107.23	10621000	9033100	5711300	16672000	12822000	NaN	1.744148	0.075577
P49411	Elongation factor Tu, mitochondrial	TUFM	1	24	24	65.9	49.874	2.31E+08	2.6E+08	2.17E+08	2.61E+08	2.7E+08	3E+08	1.174634	0.075595
P20810	Calpastatin	CAST	1	18	18	42.5	76.572	82074000	79433000	75867000	83411000	90369000	99690000	1.152064	0.075664
Q06124	Tyrosine-protein phosphatase non-recepto	PTPN11	1	14	14	34.4	68.01	32184000	40431000	40143000	42085000	45519000	48051000	1.203063	0.076057
Q9Y305	Acyl-coenzyme A thioesterase 9, mitochon	ACOT9	1	11	11	32.6	49.901	50112000	40502000	49459000	64257000	58522000	51555000	1.244594	0.076303
P04080	Cystatin-B	CSTB	1	4	4	70.4	11.139	4.24E+08	4.56E+08	4.38E+08	5.09E+08	6.26E+08	4.91E+08	1.233775	0.076322
Q99798	Aconitate hydratase, mitochondrial	ACO2	1	25	25	42.8	85.424	1.07E+08	1.08E+08	1.01E+08	1.09E+08	1.19E+08	1.14E+08	1.08415	0.076403
Q15654	Thyroid receptor-interacting protein 6	TRIP6	1	11	11	43.3	50.287	62358000	57483000	54642000	62738000	66149000	73695000	1.161041	0.076423
P55957	BH3-interacting domain death agonist;BH3	BID	1	4	4	34.4	21.994	55592000	76955000	65069000	55460000	48016000	44322000	0.747905	0.076471
P49257	Protein ERGIC-53	LMAN1	1	14	14	35.5	57.548	82564000	70626000	87743000	63735000	72437000	58182000	0.806672	0.076766
Q96GK7;Q6P2I3	Fumarylacetoacetate hydrolase domain-co	FAHD2A;FAHD2B	2	5	5	24.8	34.596	14130000	12091000	13350000	20369000	19152000	14166000	1.356726	0.077127
P06744	Glucose-6-phosphate isomerase	GPI	1	13	10	26.2	63.146	1.57E+08	1.57E+08	1.57E+08	1.59E+08	1.66E+08	1.61E+08	1.03029	0.077155
Q9Y520	Protein PRRC2C	PRRC2C	1	11	11	5.4	316.91	19633000	17408000	17535000	17015000	15270000	13536000	0.839582	0.07766
Q96A26	Protein FAM162A	FAM162A	1	3	3	20.1	17.342	1.05E+08	84036000	89368000	1.04E+08	1.12E+08	1.13E+08	1.178776	0.07773
Q9UBR2	Cathepsin Z	CTSZ	1	4	4	18.2	33.868	77121000	76463000	65251000	58806000	57603000	67442000	0.840135	0.077751
P61254	60S ribosomal protein L26	RPL26	1	4	2	24.1	17.258	1.56E+08	1.32E+08	1.61E+08	1.32E+08	1.29E+08	1.13E+08	0.831855	0.077852
Q01813	ATP-dependent 6-phosphofructokinase, pl	PFKP	1	21	19	36.9	85.595	68263000	68695000	72389000	72533000	72797000	75661000	1.055621	0.078127
P52815	39S ribosomal protein L12, mitochondrial	MRPL12	1	5	5	28.3	21.348	1.27E+08	1.1E+08	1.22E+08	1.53E+08	1.69E+08	1.28E+08	1.255587	0.078328
Q9BW60	Elongation of very long chain fatty acids pr	ELOVL1	1	2	2	9.7	32.662	1.84E+08	1.96E+08	1.64E+08	1.61E+08	1.47E+08	1.61E+08	0.862866	0.078338
P68402	Platelet-activating factor acetylhydrolase IB	PAFAH1B2	1	2	2	17.9	25.569	82687000	71711000	66720000	50850000	61500000	64543000	0.799994	0.078827
P46779	60S ribosomal protein L28	RPL28	1	4	4	24.1	15.747	1.86E+08	2.52E+08	1.62E+08	2.83E+08	3.03E+08	2.42E+08	1.379603	0.078841
Q15785	Mitochondrial import receptor subunit TOM	TOMM34	1	7	7	29.8	34.559	21464000	17002000	13077000	25722000	27812000	20628000	1.438837	0.079765
Q92974	Rho guanine nucleotide exchange factor 2	ARHGEF2	1	9	9	11.8	111.54	34269000	32124000	34078000	36546000	34451000	37767000	1.082541	0.080232
Q06830	Peroxiredoxin-1	PRDX1	1	12	9	60.8	22.11	1.28E+09	1.14E+09	1.33E+09	1.66E+09	1.55E+09	1.33E+09	1.207246	0.080284
Q9BRA2	Thioredoxin domain-containing protein 17	TXNDC17	1	4	4	45.5	13.941	1.24E+08	1.26E+08	1.75E+08	1.04E+08	1.08E+08	75033000	0.675875	0.080509
P42126	Enoyl-CoA delta isomerase 1, mitochondria	ECI1	1	5	5	23.2	32.816	67404000	65746000	55199000	49608000	50750000	56944000	0.835162	0.080636
Q96T51;Q7L099;Q8WXA3	RUN and FYVE domain-containing protein	RUFY1	3	13	13	21.8	79.817	41788000	32670000	39091000	41876000	51752000	47345000	1.241517	0.080786
P58546	Myotrophin	MTPN	1	5	5	57.6	12.895	1.68E+08	1.6E+08	1.96E+08	1.43E+08	1.36E+08	1.57E+08	0.831736	0.080917
Q14152	Eukaryotic translation initiation factor 3 sub	EIF3A	1	30	30	25.2	166.57	1.59E+08	1.62E+08	1.45E+08	1.29E+08	1.29E+08	1.49E+08	0.873423	0.08101
Q9NVE7	Pantothenate kinase 4	PANK4	1	5	5	11.5	85.99	13202000	13274000	15232000	11916000	8786200	12163000	0.787983	0.081717
P23634;Q16720	Plasma membrane calcium-transporting AT	ATP2B4	2	22	13	22	137.92	1.13E+08	1.08E+08	95071000	85567000	93432000	94961000	0.867398	0.081792
P48047	ATP synthase subunit O, mitochondrial	ATP5O	1	7	7	35.2	23.277	1.04E+08	1.28E+08	98369000	1.31E+08	1.29E+08	1.34E+08	1.191653	0.082039
Q96N66	Lysophospholipid acyltransferase 7	MBOAT7	1	4	4	12.1	52.764	83163000	1.04E+08	81220000	69093000	67927000	76959000	0.796785	0.082059
Q13573	SNW domain-containing protein 1	SNW1	1	3	3	7.3	61.494	13534000	13661000	NaN	11487000	NaN	12470000	0.880934	0.082299
P33527	Multidrug resistance-associated protein 1	ABCC1	1	11	11	10.7	171.59	44241000	55153000	52000000	42788000	39357000	44514000	0.836618	0.082598
P29590	Protein PML	PML	1	14	14	20.6	97.55	45372000	44270000	55994000	39379000	39718000	40628000	0.822084	0.083008
P55795	Heterogeneous nuclear ribonucleoprotein I	HNRNPH2	1	8	4	21.6	49.263	45168000	59710000	55501000	31686000	48278000	27278000	0.668679	0.08335
Q9NRV9	Heme-binding protein 1	HEBP1	1	3	3	21.7	21.097	43233000	26394000	40068000	53916000	56363000	43760000	1.404248	0.083656
Q12931	Heat shock protein 75 kDa, mitochondrial	TRAP1	1	15	15	27.7	80.109	42707000	39798000	36213000	44186000	47440000	42943000	1.133518	0.083879
P32969	60S ribosomal protein L9	RPL9	1	8	8	47.4	21.863	1.19E+08	1.38E+08	1.19E+08	1.48E+08	1.45E+08	1.35E+08	1.140475	0.083986
Q96JY6	PDZ and LIM domain protein 2	PDLIM2	1	4	4	15.9	37.458	44553000	34478000	49389000	31319000	35522000	28342000	0.741185	0.084861
P61960	Ubiquitin-fold modifier 1	UFM1	1	3	3	60	9.1175	1.13E+08	62679000	90667000	1.27E+08	1.17E+08	1.22E+08	1.378023	0.085146
P82979	SAP domain-containing ribonucleoprotein	SARNP	1	4	4	24.8	23.671	45826000	31065000	37345000	31024000	26613000	24770000	0.721375	0.085183
Q6UXN9	WD repeat-containing protein 82	WDR82	1	2	2	10.5	35.079	8979800	NaN	9895300	7540300	8023800	NaN	0.824584	0.085434
P84098	60S ribosomal protein L19	RPL19	1	3	3	17.3	23.466	2.44E+08	2.78E+08	2.74E+08	2.95E+08	NaN	3.17E+08	1.15258	0.085886
P09651;Q32P51;A0A2R8Y4L2	Heterogeneous nuclear ribonucleoprotein /	HNRNPA1;HNRNP	3	17	17	46.8	38.746	9.16E+08	9.77E+08	8.99E+08	7.56E+08	7.26E+08	9.04E+08	0.854443	0.086002
P60468	Protein transport protein Sec61 subunit be	SEC61B	1	3	3	37.5	9.9743	1.3E+08	1.72E+08	1.57E+08	1.81E+08	1.77E+08	1.88E+08	1.186356	0.087264
P55265	Double-stranded RNA-specific adenosine	ADAR	1	17	17	20.9	136.06	48187000	54755000	53634000	39045000	40899000	49984000	0.829808	0.087319
P46940;Q13576;Q86VI3	Ras GTPase-activating-like protein IQGAP1	IQGAP1	3	67	67	48.7	189.25	2.8E+08	2.62E+08	2.83E+08	2.54E+08	2.56E+08	2.65E+08	0.939408	0.08738
P28838	Cytosol aminopeptidase	LAP3	1	19	19	45.5	56.166	1.08E+08	94513000	1.01E+08	90407000	91689000	94327000	0.911646	0.08753
P08962	CD63 antigen	CD63	1	3	3	8.4	25.636	1.64E+08	1.62E+08	1.43E+08	4.46E+08	3.63E+08	1.84E+08	2.116294	0.087596
Q9HD20	Manganese-transporting ATPase 13A1	ATP13A1	1	11	11	12.7	132.95	25910000	20403000	22500000	15991000	19122000	20066000	0.801869	0.087966
O43169	Cytochrome b5 type B	CYB5B	1	5	5	59.3	16.694	1.18E+08	1.41E+08	1.34E+08	84918000	1.15E+08	1.13E+08	0.797065	0.088392
Q9UNZ2	NSFL1 cofactor p47	NSFL1C	1	11	11	41.6	40.572	64217000	53618000	69474000	51568000	49186000	53898000	0.825652	0.088518
O60763	General vesicular transport factor p115	USO1	1	26	26	43.5	107.89	1.07E+08	1.22E+08	1.13E+08	95506000	94944000	1.09E+08	0.875084	0.088869

P80303	Nucleobindin-2;Nesfatin-1	NUCB2	1	7	7	22.1	50.222	33369000	38039000	28087000	27609000	25036000	26897000	0.799457	0.089073
Q13404	Ubiquitin-conjugating enzyme E2 variant 1	UBE2V1	1	5	2	42.2	16.495	89587000	1.1E+08	1.07E+08	84919000	85065000	91614000	0.853788	0.089192
Q16762	Thiosulfate sulfurtransferase	TST	1	4	4	21.2	33.429	33006000	18174000	29405000	35459000	36243000	40920000	1.397555	0.089239
Q9P0I2	ER membrane protein complex subunit 3	EMC3	1	2	2	10	29.952	11567000	NaN	9932800	8824000	9520500	8955900	0.846532	0.090251
P0DP25;P0DP24;P0DP23			3	11	11	87.9	16.837	1.76E+09	1.46E+09	1.83E+09	1.38E+09	1.44E+09	1.46E+09	0.846597	0.090281
P00492	Hypoxanthine-guanine phosphoribosyltran	HPRT1	1	6	6	32.6	24.579	29788000	25856000	39162000	41157000	39211000	40958000	1.279729	0.091337
O14562	Ubiquitin domain-containing protein UBFD	UBFD1	1	3	3	11	33.382	NaN	51416000	60375000	43904000	40441000	49101000	0.795806	0.091683
P07814	Bifunctional glutamate/proline--tRNA ligas	EPRS	1	42	42	41.1	170.59	1.23E+08	1.22E+08	1.24E+08	1.02E+08	1.11E+08	1.2E+08	0.902232	0.092112
Q13510	Acid ceramidase;Acid ceramidase subunit a	ASAH1	1	7	7	21.5	44.659	98087000	63892000	61244000	1.1E+08	1.02E+08	94727000	1.375293	0.092175
Q8WUH6	Transmembrane protein 263	TMEM263	1	3	3	40.5	11.748	23889000	25566000	NaN	45018000	36251000	31240000	1.516652	0.092483
P05976;P08590	Myosin light chain 1/3, skeletal muscle isof	MYL1;MYL3	2	2	2	8.2	21.145	2.53E+08	2.34E+08	1.95E+08	1.91E+08	1.84E+08	1.19E+08	0.723529	0.093182
O75351	Vacuolar protein sorting-associated protei	VPS4B	1	4	3	13.5	49.301	34605000	29435000	27370000	33076000	38725000	42513000	1.250563	0.093638
O95299	NADH dehydrogenase [ubiquinone] 1 alph	NDUFA10	1	4	4	11.3	40.75	17768000	16090000	15975000	20552000	24543000	17923000	1.264584	0.09403
Q15124	Phosphoglucomutase-like protein 5	PGM5	1	6	6	14.6	62.224	10798000	10837000	11729000	17322000	16561000	11542000	1.361497	0.094034
P55060	Exportin-2	CSE1L	1	12	12	16.4	110.42	28692000	32065000	32266000	35623000	33446000	41598000	1.189674	0.09481
P63261	Actin, cytoplasmic 2;Actin, cytoplasmic 2, N	ACTG1	1	25	2	75.7	41.792	7.74E+09	9.28E+09	8.32E+09	6.89E+09	6.96E+09	7.88E+09	0.857914	0.095166
Q6ZSR9	Uncharacterized protein FLJ45252		1	4	4	20	37.976	24370000	18798000	21787000	13049000	19960000	14853000	0.736849	0.095489
P13807	Glycogen [starch] synthase, muscle	GYS1	1	6	6	10.4	83.785	15799000	18159000	15452000	15351000	13407000	13953000	0.86442	0.095744
Q3ZCQ8	Mitochondrial import inner membrane tran	TIMM50	1	3	3	11	39.646	17969000	20851000	18602000	20470000	21312000	22096000	1.112431	0.096004
Q99685	Monoglyceride lipase	MGLL	1	7	7	33.3	33.261	24428000	20287000	27085000	26606000	29541000	30978000	1.21344	0.096351
P04844	Dolichyl-diphosphooligosaccharide--prote	RPN2	1	13	13	33.3	69.283	1.58E+08	1.87E+08	1.63E+08	1.26E+08	1.31E+08	1.6E+08	0.822552	0.096589
P61970	Nuclear transport factor 2	NUTF2	1	4	4	51.2	14.478	1.12E+08	1.11E+08	99296000	1.16E+08	1.46E+08	1.25E+08	1.198226	0.096661
Q9UHB6	LIM domain and actin-binding protein 1	LIMA1	1	23	23	37	85.225	1.05E+08	1.02E+08	1.1E+08	1.16E+08	1.09E+08	1.27E+08	1.111066	0.0977
P51398	28S ribosomal protein S29, mitochondrial	DAP3	1	5	5	15.3	45.566	21377000	26232000	19153000	25274000	28163000	28163000	1.222252	0.09806
P84077;P61204	ADP-ribosylation factor 1;ADP-ribosylation	ARF1;ARF3	2	7	4	42	20.697	3.32E+08	3.39E+08	3.17E+08	2.93E+08	3.07E+08	3.21E+08	0.932312	0.098298
Q4J6C6	Prolyl endopeptidase-like	PREPL	1	5	5	8	83.926	8955100	12716000	10419000	15340000	12436000	13287000	1.279616	0.098323
Q8NCA5	Protein FAM98A	FAM98A	1	8	6	24.1	55.272	46141000	48084000	46102000	33516000	40778000	44639000	0.847542	0.0986
P62318	Small nuclear ribonucleoprotein Sm D3	SNRPD3	1	5	5	53.2	13.916	84858000	87482000	87247000	72703000	75208000	41229000	0.728619	0.09891
Q6NUM9	All-trans-retinol 13,14-reductase	RETSAT	1	5	5	10.2	66.819	18747000	17091000	NaN	30284000	21156000	27288000	1.464516	0.099134
Q6P2Q9	Pre-mRNA-processing-splicing factor 8	PRPF8	1	20	20	12.2	273.6	52357000	53642000	47144000	40845000	34673000	48678000	0.810981	0.099316
P62851	40S ribosomal protein S25	RPS25	1	4	4	24	13.742	1.81E+08	1.63E+08	1.41E+08	3.64E+08	2.36E+08	2.05E+08	1.659492	0.099708
P13073	Cytochrome c oxidase subunit 4 isoform 1,	COX4I1	1	6	6	32	19.576	1.99E+08	1.8E+08	1.69E+08	2.15E+08	2.14E+08	1.93E+08	1.134439	0.099839
P51149	Ras-related protein Rab-7a	RAB7A	1	12	12	62.8	23.489	1.53E+08	1.63E+08	1.44E+08	1.68E+08	1.67E+08	1.96E+08	1.152671	0.100619
P28074	Proteasome subunit beta type-5	PSMB5	1	6	6	24.7	28.48	76420000	83205000	63281000	60558000	59381000	64425000	0.827093	0.100679
P21266	Glutathione S-transferase Mu 3	GSTM3	1	11	10	52.9	26.559	1.21E+08	1.47E+08	1.2E+08	1.09E+08	1.1E+08	1.14E+08	0.856595	0.100724
Q3SY69	Mitochondrial 10-formyltetrahydrofolate d	ALDH1L2	1	9	9	13.1	101.74	16380000	19307000	14257000	19912000	18964000	21560000	1.210075	0.101205
P12109	Collagen alpha-1(VI) chain	COL6A1	1	7	7	13.1	108.53	24434000	30123000	28959000	14297000	25329000	20742000	0.722832	0.10136
O43847	Nardilysin	NRD1	1	11	11	11.7	131.7	43201000	29892000	44551000	30375000	28085000	29151000	0.744713	0.101444
Q9UL25	Ras-related protein Rab-21	RAB21	1	5	5	32.4	24.347	89827000	78426000	76831000	60605000	67602000	77068000	0.83757	0.102337
P32189;Q14409	Glycerol kinase;Putative glycerol kinase 3	GK;GK3P	2	6	6	15	61.244	22442000	21966000	22268000	17903000	22058000	18080000	0.870493	0.102389
Q6RW13	Type-1 angiotensin II receptor-associated	AGTRAP	1	2	2	24.5	17.419	17539000	17797000	20685000	NaN	15552000	15733000	0.837677	0.102595
O75934	Pre-mRNA-splicing factor SPF27	BCAS2	1	4	4	27.1	26.131	14469000	17302000	14668000	12273000	8776700	14039000	0.755587	0.102837
P23368	NAD-dependent malic enzyme, mitochonc	ME2	1	6	6	13.4	65.443	23261000	16583000	23457000	29055000	31870000	23490000	1.333549	0.10294
P50914	60S ribosomal protein L14	RPL14	1	3	3	15.3	23.432	2.23E+08	2.97E+08	1.92E+08	3.2E+08	2.83E+08	3.18E+08	1.295057	0.1031
P29317;P54764;P54760;Q1537	Ephrin type-A receptor 2	EPHA2	6	7	6	8.8	108.27	16187000	16876000	15866000	19082000	16791000	17668000	1.094259	0.103253
P07384	Calpain-1 catalytic subunit	CAPN1	1	11	11	19.5	81.889	68364000	62710000	72036000	54176000	63423000	61097000	0.879799	0.104075
O95295	SNARE-associated protein Snapin	SNAPIN	1	4	4	41.9	14.874	9538000	11224000	NaN	17463000	13161000	13552000	1.418489	0.104093
O43598	2-deoxynucleoside 5-phosphate N-hydrol	DNPH1	1	2	2	20.1	19.108	32243000	31112000	27351000	37723000	36850000	31483000	1.169228	0.104804
P53634	Dipeptidyl peptidase 1;Dipeptidyl peptidas	CTSC	1	4	4	11.9	51.853	35295000	30782000	31442000	27275000	30212000	21196000	0.806848	0.104883
Q8IVM0	Coiled-coil domain-containing protein 50	CCDC50	1	8	8	44.4	35.822	1E+08	69334000	92466000	58747000	75085000	62633000	0.749609	0.106073
Q99490	Arf-GAP with GTPase, ANK repeat and PH	AGAP2	1	2	2	3.4	124.67	4.27E+08	4.33E+08	5.8E+08	3.15E+08	4.07E+08	3.63E+08	0.754086	0.106587
O75533	Splicing factor 3B subunit 1	SF3B1	1	24	24	27.9	145.83	1.24E+08	1.41E+08	1.35E+08	1.07E+08	1.16E+08	1.27E+08	0.878747	0.107057
Q9UNE7	E3 ubiquitin-protein ligase CHIP	STUB1	1	9	9	33.7	34.856	44988000	39704000	37604000	43966000	46140000	51098000	1.154608	0.107348
Q92508	Piezo-type mechanosensitive ion channel c	PIEZO1	1	7	7	3.7	286.79	16510000	18760000	18105000	16111000	16897000	15005000	0.899541	0.107572
P02786	Transferrin receptor protein 1;Transferrin re	TFRC	1	9	9	14.3	84.87	9724200	13561000	13015000	23001000	17496000	13858000	1.497375	0.107917
P23246	Splicing factor, proline- and glutamine-rich	SFPQ	1	22	21	36.4	76.149	2.07E+08	2.25E+08	2.06E+08	1.94E+08	1.7E+08	2.03E+08	0.887907	0.10799
P61224;A6NIZ1	Ras-related protein Rap-1b;Ras-related pr	RAP1B	2	7	2	38	20.825	97884000	62713000	96564000	1.3E+08	1.22E+08	98060000	1.358526	0.108169
Q9UBC2	Epidermal growth factor receptor substrate	EPS15L1	1	9	9	16.1	94.254	13249000	15263000	NaN	17395000	15635000	16517000	1.158506	0.108229
P00390	Glutathione reductase, mitochondrial	GSR	1	9	9	29.9	56.256	1.24E+08	1.44E+08	1.13E+08	85029000	1.03E+08	1.14E+08	0.797006	0.108237

Q9H074	Polyadenylate-binding protein-interacting	PAIP1	1	3	3	7.3	53.524	20156000	16741000	NaN	22109000	21708000	20802000	1.167557	0.108805
P55735	Protein SEC13 homolog	SEC13	1	11	11	44.7	35.54	1.1E+08	1.26E+08	1.09E+08	1.04E+08	93875000	1.06E+08	0.881692	0.108854
P63167	Dynein light chain 1, cytoplasmic	DYNLL1	1	3	1	49.4	10.366	1.59E+08	1.37E+08	1.43E+08	2.03E+08	1.93E+08	1.5E+08	1.244452	0.109091
P61964	WD repeat-containing protein 5	WDR5	1	2	2	9.3	36.588	14610000	15082000	17843000	13483000	NaN	10939000	0.770653	0.10926
Q16644	MAP kinase-activated protein kinase 3	MAPKAPK3	1	2	2	8.9	42.987	NaN	6E+08	4.59E+08	7.18E+08	NaN	7.36E+08	1.373257	0.109723
Q16222	UDP-N-acetylhexosamine pyrophosphoryl	UAP1	1	10	9	25.3	58.768	53268000	64559000	44274000	62147000	73752000	67751000	1.256316	0.10975
Q15942	Zyxin	ZYX	1	15	15	47.2	61.277	2.67E+08	2.51E+08	2.91E+08	2.37E+08	2.56E+08	2.23E+08	0.885026	0.109834
P52789;P52790	Hexokinase-2	HK2	2	10	8	14.7	102.38	21861000	22129000	24045000	12484000	13054000	22370000	0.704167	0.110068
P51178	1-phosphatidylinositol 4,5-bisphosphate p	PLCD1	1	7	7	13.2	85.664	31111000	21365000	NaN	35746000	38382000	32797000	1.358399	0.110342
O94929	Actin-binding LIM protein 3	ABLIM3	1	6	6	11.7	77.801	19885000	18681000	16716000	15262000	16075000	NaN	0.850286	0.110697
O60826	Coiled-coil domain-containing protein 22	CCDC22	1	4	4	8.6	70.755	15968000	19841000	12629000	21233000	19368000	21129000	1.274413	0.110707
P13693;Q56UQ5	Translationally-controlled tumor protein	TPT1	2	7	7	41.3	19.595	3.26E+08	3.18E+08	4.49E+08	2.91E+08	2.75E+08	2.64E+08	0.759079	0.110715
O00401	Neural Wiskott-Aldrich syndrome protein	WASL	1	4	4	16.4	54.826	11090000	NaN	10805000	10175000	9455600	10480000	0.916818	0.110761
P56537	Eukaryotic translation initiation factor 6	EIF6	1	5	5	33.1	26.599	1.14E+08	1.01E+08	97974000	1.47E+08	1.43E+08	1.07E+08	1.270058	0.11102
P05388;Q8NHW5	60S acidic ribosomal protein P0;60S acidic	RPLP0;RPLP0P6	2	11	11	44.2	34.273	4.27E+08	4.15E+08	3.87E+08	3.64E+08	3.97E+08	3.74E+08	0.923858	0.111067
Q92945	Far upstream element-binding protein 2	KHSRP	1	13	12	28.6	73.114	43234000	39918000	42418000	49943000	52786000	42518000	1.156701	0.111277
O15126	Secretory carrier-associated membrane pro	SCAMP1	1	3	3	17.8	37.92	15543000	NaN	13268000	NaN	17570000	19620000	1.290826	0.111648
Q13492	Phosphatidylinositol-binding clathrin assem	PICALM	1	7	7	18.3	70.754	40647000	46412000	26417000	54212000	49966000	47458000	1.336283	0.11198
Q9NZL4	Hsp70-binding protein 1	HSPBP1	1	7	7	23.7	39.302	17278000	20301000	16553000	22646000	23071000	18973000	1.195042	0.112396
Q9UFN0;Q9BS92	Protein NipSnap homolog 3A	NIPSNAP3A	2	3	3	13.4	28.466	13702000	NaN	12908000	18453000	15772000	NaN	1.286171	0.11252
Q9H845	Acyl-CoA dehydrogenase family member 9	ACAD9	1	7	7	15.6	68.76	30755000	36188000	33652000	36402000	38819000	36197000	1.10759	0.112782
P04181	Ornithine aminotransferase, mitochondrial;	OAT	1	10	10	32.1	48.534	46716000	34107000	39034000	47732000	47476000	46965000	1.186189	0.113013
P35268	60S ribosomal protein L22	RPL22	1	2	2	18.8	14.787	NaN	60613000	48778000	1.87E+08	1.29E+08	90707000	2.482819	0.113073
P61764	Syntaxin-binding protein 1	STXBP1	1	13	13	28.3	67.568	26222000	29592000	26310000	28589000	30885000	33306000	1.129755	0.113175
P42677	40S ribosomal protein S27	RPS27	1	5	3	40.5	9.461	2.56E+08	2.92E+08	2.15E+08	3.23E+08	2.79E+08	3.18E+08	1.208215	0.113278
P04439;P30511	HLA class I histocompatibility antigen, A-3	HLA-A	2	7	3	26	40.84	91090000	89650000	74597000	71595000	74042000	76563000	0.870222	0.113376
Q15293	Reticulocalbin-1	RCN1	1	13	13	48.9	38.89	5.02E+08	4.73E+08	5.21E+08	4.04E+08	4.79E+08	4.55E+08	0.894828	0.113913
P23396	40S ribosomal protein S3	RPS3	1	17	17	71.2	26.688	2.99E+08	2.88E+08	2.92E+08	2.86E+08	2.76E+08	2.51E+08	0.924149	0.113934
Q9BSH4	Translational activator of cytochrome c oxi	TACO1	1	5	5	22.2	32.477	18784000	15033000	15066000	19039000	20035000	18071000	1.169016	0.114368
P01889;P13747	HLA class I histocompatibility antigen, B-7	HLA-B	2	7	2	27.9	40.46	77083000	68926000	1.04E+08	53705000	65761000	63393000	0.73264	0.115395
O15061	Synemin	SYNM	1	14	14	12.8	172.87	1.07E+08	1E+08	1.3E+08	93133000	99984000	83550000	0.818275	0.115479
P42765	3-ketoacyl-CoA thiolase, mitochondrial	ACAA2	1	9	9	36.5	41.924	66888000	64391000	54937000	50875000	55145000	56224000	0.871268	0.11571
P36871	Phosphoglucomutase-1	PGM1	1	8	8	19.9	61.448	23600000	20773000	21418000	23749000	23781000	23440000	1.078719	0.115828
Q9Y262	Eukaryotic translation initiation factor 3 sub	EIF3L	1	15	15	29.6	66.726	47242000	40388000	47437000	37886000	41191000	40759000	0.887234	0.116203
P04179	Superoxide dismutase [Mn], mitochondrial	SOD2	1	8	8	42.8	24.75	2.63E+08	2.13E+08	2.68E+08	2.11E+08	2.21E+08	2.02E+08	0.853639	0.116819
P61081	NEDD8-conjugating enzyme Ubc12	UBE2M	1	6	6	41.5	20.9	34157000	38464000	45746000	30799000	32264000	34220000	0.821876	0.116886
O75368	SH3 domain-binding glutamic acid-rich-lik	SH3BGRL	1	6	6	61.4	12.774	75185000	88102000	86155000	67376000	73777000	77979000	0.878489	0.116891
O75190;Q8WWF6;Q7Z6W7	DnaJ homolog subfamily B member 6;DnaJ	DNAJB6;DNAJB3	3	3	3	12	36.087	24788000	22770000	19096000	24193000	30410000	26743000	1.220422	0.116905
O96011	Peroxisomal membrane protein 11B	PEX11B	1	5	5	20.5	28.431	26054000	27547000	29886000	30834000	33496000	43958000	1.297064	0.117674
Q9BY32	Inosine triphosphate pyrophosphatase	ITPA	1	4	4	30.9	21.445	59315000	51695000	49278000	37104000	46193000	48997000	0.825352	0.117715
O15371	Eukaryotic translation initiation factor 3 sub	EIF3D	1	13	13	30.7	63.972	77111000	80239000	71937000	64568000	65430000	74881000	0.893548	0.117905
Q99417	C-Myc-binding protein	MYCBP	1	3	3	36.9	11.967	24339000	26331000	21259000	15163000	16871000	23085000	0.766297	0.118159
Q9GZZ1	N-alpha-acetyltransferase 50	NAA50	1	6	6	42.6	19.398	31689000	23177000	37065000	22567000	23943000	20741000	0.731538	0.118359
Q8TCJ2	Dolichyl-diphosphooligosaccharide--prote	STT3B	1	4	4	5.4	93.673	49377000	40118000	39834000	39302000	33749000	35162000	0.836726	0.118435
P35475	Alpha-L-iduronidase	IDUA	1	4	4	8.6	72.669	25772000	31234000	21137000	31453000	38973000	30938000	1.29716	0.118587
P43155	Carnitine O-acetyltransferase	CRAT	1	10	10	20.9	70.857	21609000	33623000	36988000	37769000	40420000	43389000	1.318347	0.118772
Q9H2P9	Diphthine synthase	DPH5	1	3	3	16.1	31.651	8834800	6663300	11320000	11396000	13030000	11114000	1.325224	0.119456
Q04637;REV__Q8NEG2	Eukaryotic translation initiation factor 4 gar	EIF4G1	2	30	27	23.1	175.49	79525000	88476000	80166000	78089000	76851000	75671000	0.929257	0.119762
P13639	Elongation factor 2	EEF2	1	45	44	63.2	95.337	6.26E+08	6.44E+08	6.17E+08	6.87E+08	6.52E+08	6.41E+08	1.049904	0.119988
Q9NPJ3	Acyl-coenzyme A thioesterase 13;Acyl-coe	ACOT13	1	4	4	36.4	14.96	35175000	22228000	19308000	43440000	31349000	37351000	1.46185	0.12024
O95292	Vesicle-associated membrane protein-asso	VAPB	1	7	6	38.3	27.228	1.41E+08	1.17E+08	1.25E+08	1.09E+08	1.15E+08	1.16E+08	0.888938	0.121922
Q9NZB2;Q5T035	Constitutive coactivator of PPAR-gamma-li	FAM120A	2	22	22	28.1	121.89	1.44E+08	1.61E+08	1.64E+08	1.22E+08	1.24E+08	1.53E+08	0.853184	0.122311
Q9Y4X5	E3 ubiquitin-protein ligase ARIH1	ARIH1	1	3	3	6.5	64.117	19743000	19336000	20575000	14480000	19461000	17018000	0.854243	0.122431
P35222;P14923	Catenin beta-1	CTNNB1	2	10	10	17	85.496	34010000	32557000	39103000	25045000	27570000	33831000	0.818075	0.122455
P48449	Lanosterol synthase	LSS	1	7	7	12.7	83.308	10697000	11612000	14869000	21804000	19445000	13014000	1.459546	0.122516
P20042	Eukaryotic translation initiation factor 2 sub	EIF2S2	1	9	9	31.5	38.388	82714000	74355000	79842000	72428000	64068000	75660000	0.895509	0.123221
Q9UQ35	Serine/arginine repetitive matrix protein 2	SRRM2	1	12	12	6.6	299.61	73927000	64926000	76516000	60363000	66057000	65761000	0.892334	0.123391
P11940;Q9H361;Q4VXU2;Q5J(	Polyadenylate-binding protein 1;Polyadeny	PABPC1;PABPC3	4	15	10	26.4	70.67	1.11E+08	99079000	1.01E+08	93284000	97991000	96470000	0.926599	0.123469
P0DPI2;A0A0B4J2D5			2	5	5	30.6	28.17	68184000	78234000	58094000	76240000	80260000	93566000	1.222745	0.124261

Q13423	NAD(P) transhydrogenase, mitochondrial	NNT	1	8	8	11.1	113.89	31742000	25089000	31252000	34068000	32697000	34055000	1.144602	0.124375
Q9Y6A9	Signal peptidase complex subunit 1	SPCS1	1	1	1	10.1	18.298	61696000	64770000	60055000	52827000	59306000	59394000	0.919612	0.124393
P48651	Phosphatidylserine synthase 1	PTDSS1	1	4	4	12.5	55.527	10572000	10381000	11257000	11759000	11322000	NaN	1.074868	0.124733
Q13618	Cullin-3	CUL3	1	8	8	12.5	88.929	19363000	20932000	22116000	13316000	15779000	20397000	0.793001	0.12482
P09669	Cytochrome c oxidase subunit 6C	COX6C	1	2	2	17.3	8.7813	29356000	35195000	36153000	23439000	30982000	28462000	0.823036	0.125133
P10644	cAMP-dependent protein kinase type I-alp	PRKAR1A	1	10	10	32.5	42.981	78319000	66096000	74562000	67824000	65916000	62229000	0.89493	0.125568
P14866	Heterogeneous nuclear ribonucleoprotein I	HNRNPL	1	19	19	52.3	64.132	1.19E+08	1.22E+08	1.14E+08	1.09E+08	1.17E+08	1.09E+08	0.943884	0.127157
Q7L2H7	Eukaryotic translation initiation factor 3 sub	EIF3M	1	10	10	46.3	42.502	71588000	79264000	73901000	58003000	72238000	67192000	0.878444	0.12749
O75347	Tubulin-specific chaperone A	TBCA	1	6	6	51.9	12.855	1.29E+08	1.08E+08	1.26E+08	1.88E+08	1.5E+08	1.3E+08	1.286311	0.128056
O75947	ATP synthase subunit d, mitochondrial	ATP5H	1	8	8	59	18.491	73433000	72496000	86686000	82793000	89605000	89453000	1.125684	0.128765
Q8NHP8	Putative phospholipase B-like 2;Putative ph	PLBD2	1	4	4	9.3	65.471	19947000	23853000	19949000	22863000	24922000	29620000	1.214215	0.129062
Q96QK1	Vacuolar protein sorting-associated protein	VPS35	1	16	16	24.6	91.706	94547000	85412000	87857000	98594000	1.03E+08	91240000	1.092369	0.129412
P62241	40S ribosomal protein S8	RPS8	1	10	10	51.4	24.205	2.75E+08	2.94E+08	2.86E+08	2.97E+08	3.08E+08	2.91E+08	1.048523	0.129638
Q96LJ7	Dehydrogenase/reductase SDR family mem	DHRS1	1	3	3	17.3	33.909	10645000	14051000	NaN	16305000	17180000	NaN	1.355888	0.129672
Q9BZG1	Ras-related protein Rab-34	RAB34	1	4	4	20.1	29.044	24493000	23792000	30986000	24760000	18181000	18324000	0.772855	0.129848
Q9GZT3	SRA stem-loop-interacting RNA-binding p	SLIRP	1	5	5	53.2	12.349	20062000	19396000	19222000	20946000	36781000	27194000	1.447188	0.130756
P62913	60S ribosomal protein L11	RPL11	1	7	7	48.3	20.252	2.56E+08	2.77E+08	2.9E+08	2.17E+08	2.38E+08	2.68E+08	0.877352	0.130952
Q14696	LDLR chaperone MESD	MESDC2	1	5	5	30.8	26.076	60631000	51748000	61411000	42432000	53569000	51595000	0.849278	0.131983
Q8WTT2	Nucleolar complex protein 3 homolog	NOC3L	1	2	2	4.2	92.547	10385000	9917400	8996400	10752000	13474000	NaN	1.24029	0.132359
O75531	Barrier-to-autointegration factor;Barrier-to	BANF1	1	1	1	27	10.058	2.08E+08	2.4E+08	1.89E+08	1.63E+08	1.5E+08	2.02E+08	0.807013	0.132685
Q01844	RNA-binding protein EWS	EWSR1	1	5	5	9.9	68.477	62153000	54857000	57659000	56641000	50699000	51225000	0.907803	0.132733
Q14839;Q8TDI0;Q12873	Chromodomain-helicase-DNA-binding pro	CHD4;CHD5	3	6	6	4.8	218	17420000	17858000	17781000	16217000	17317000	17318000	0.958405	0.132968
P62834	Ras-related protein Rap-1A	RAP1A	1	7	2	38	20.987	15720000	12185000	14678000	26694000	29222000	13964000	1.64103	0.133077
Q13200	26S proteasome non-ATPase regulatory su	PSMD2	1	26	26	40.3	100.2	1.69E+08	1.71E+08	1.57E+08	1.39E+08	1.46E+08	1.64E+08	0.901395	0.133203
P62899	60S ribosomal protein L31	RPL31	1	3	3	32.8	14.463	1.77E+08	1.8E+08	1.65E+08	1.98E+08	1.83E+08	NaN	1.095678	0.133402
P50502;Q8NFI4;Q8IZP2	Hsc70-interacting protein;Putative protein	ST13;ST13P5;ST13	3	7	7	23.6	41.331	2.17E+08	2.11E+08	2.45E+08	2.44E+08	2.39E+08	2.54E+08	1.095662	0.133901
Q14108	Lysosome membrane protein 2	SCARB2	1	7	7	12.6	54.29	1.2E+08	82307000	1.19E+08	1.25E+08	1.3E+08	1.39E+08	1.227457	0.133933
Q5JTV8	Torsin-1A-interacting protein 1	TOR1AIP1	1	15	14	40.1	66.248	1.21E+08	1.32E+08	1.1E+08	98187000	1.04E+08	1.15E+08	0.872355	0.134119
P12955	Xaa-Pro dipeptidase	PEPD	1	6	6	17.2	54.548	42104000	33207000	48972000	63944000	65538000	42887000	1.386907	0.136087
P20592	Interferon-induced GTP-binding protein M	MX2	1	5	2	10.5	82.088	12743000	NaN	13688000	7121900	NaN	10591000	0.670156	0.136189
Q9HC07	Transmembrane protein 165	TMEM165	1	3	3	16.7	34.905	11934000	12082000	14235000	11364000	9342900	11704000	0.847322	0.136475
Q9NRW3	DNA dC->dU-editing enzyme APOBEC-3C	APOBEC3C	1	2	2	16.3	22.826	2.32E+08	2.47E+08	2.64E+08	1.54E+08	2.39E+08	2.04E+08	0.803264	0.13666
Q99829	Copine-1	CPNE1	1	8	8	16.9	59.058	30831000	30416000	27494000	35684000	29892000	34728000	1.130301	0.13716
Q99460	26S proteasome non-ATPase regulatory su	PSMD1	1	24	24	30.5	105.84	1.38E+08	1.51E+08	1.47E+08	1.09E+08	1.31E+08	1.4E+08	0.870775	0.137483
Q9UKY7	Protein CDV3 homolog	CDV3	1	9	9	61.6	27.335	1.03E+08	1.05E+08	1.08E+08	1.07E+08	1.21E+08	1.38E+08	1.159243	0.137582
P60953	Cell division control protein 42 homolog	CDC42	1	6	5	36.6	21.258	2.27E+08	2.23E+08	2.08E+08	2.18E+08	1.88E+08	1.81E+08	0.892646	0.137881
P17813	Endoglin	ENG	1	5	5	10.5	70.577	27416000	32184000	17587000	15711000	NaN	13160000	0.56106	0.138466
O43852	Calumenin	CALU	1	23	23	63.5	37.106	1.07E+09	1.13E+09	9.24E+08	8.28E+08	8.93E+08	9.84E+08	0.865957	0.138541
Q9Y3A6	Transmembrane emp24 domain-containing	TMED5	1	3	3	14.4	26.005	37520000	38742000	38765000	47811000	39235000	42117000	1.122893	0.138682
Q14232	Translation initiation factor eIF-2B subunit a	EIF2B1	1	5	5	21.3	33.712	26563000	32064000	36120000	26315000	25695000	27211000	0.836132	0.13869
Q9UMX5	Neudesin	NENF	1	6	6	51.7	18.856	57033000	44273000	59179000	41329000	49152000	39468000	0.809727	0.138847
Q96A33	Coiled-coil domain-containing protein 47	CCDC47	1	5	5	14.3	55.873	12979000	16478000	14614000	9327000	10153000	14342000	0.767443	0.138923
P27487	Dipeptidyl peptidase 4;Dipeptidyl peptidase	DPP4	1	13	13	20	88.278	60841000	59077000	55119000	87527000	75235000	58967000	1.266755	0.139025
O60264;P28370	SWI/SNF-related matrix-associated actin-c	SMARCA5;SMARC	2	4	4	5	121.9	24152000	20128000	19422000	18246000	15771000	NaN	0.801003	0.13945
P54727	UV excision repair protein RAD23 homolog	RAD23B	1	8	6	22	43.171	79110000	94370000	65809000	98732000	88762000	1.03E+08	1.212902	0.139906
O00471	Exocyst complex component 5	EXOC5	1	5	5	7.6	81.852	11992000	17851000	14010000	16643000	18392000	19122000	1.234967	0.140004
P42025	Beta-centractin	ACTR1B	1	5	4	30.3	42.293	69953000	66412000	71361000	71939000	78886000	94444000	1.180733	0.140165
P25705	ATP synthase subunit alpha, mitochondrial	ATP5A1	1	18	18	40.3	59.75	5.76E+08	5.04E+08	5.44E+08	6.22E+08	6.41E+08	5.51E+08	1.117272	0.140389
Q9UQ80	Proliferation-associated protein 2G4	PA2G4	1	13	13	44.9	43.786	87037000	86820000	92622000	86495000	79373000	84821000	0.940746	0.14043
P62753	40S ribosomal protein S6	RPS6	1	7	7	24.9	28.68	3.25E+08	3.22E+08	3.93E+08	2.9E+08	3.25E+08	2.66E+08	0.847238	0.140562
O60551	Glycylpeptide N-tetradecanoyltransferase 2	NMT2	1	4	2	12	56.98	26331000	29392000	37882000	18295000	25771000	25989000	0.748411	0.140682
P42785	Lysosomal Pro-X carboxypeptidase	PRCP	1	4	4	12.5	55.799	20299000	21845000	32430000	50737000	32946000	31118000	1.539424	0.141219
Q9BXP5	Serrate RNA effector molecule homolog	SRRT	1	3	3	5.7	100.67	NaN	10889000	11669000	10720000	10636000	10734000	0.94837	0.141363
O95865	N(G),N(G)-dimethylarginine dimethylaminc	DDAH2	1	7	7	38.6	29.644	38513000	37987000	43847000	35693000	34139000	38286000	0.898386	0.141382
P62995	Transformer-2 protein homolog beta	TRA2B	1	4	4	17.4	33.665	44275000	52318000	41698000	35092000	36423000	43815000	0.833966	0.142068
P16401	Histone H1.5	HIST1H1B	1	4	4	17.7	22.58	46036000	45207000	28576000	80827000	54410000	47848000	1.528013	0.142498
P07737;CON__P02584	Profilin-1	PFN1	2	10	10	87.9	15.054	1.94E+09	1.93E+09	1.7E+09	1.7E+09	1.64E+09	1.75E+09	0.915922	0.143371
P62316	Small nuclear ribonucleoprotein Sm D2	SNRPD2	1	6	6	44.9	13.527	1.29E+08	1.25E+08	1.61E+08	1.09E+08	1.2E+08	1.21E+08	0.843418	0.144256
Q96P70	Importin-9	IPO9	1	10	10	13.9	115.96	38162000	44412000	43361000	44243000	45680000	49239000	1.10503	0.144664

P50402	Emerin	EMD	1	9	9	51.6	28.994	85584000	85274000	73685000	64517000	74452000	76425000	0.880802	0.14504
P37837	Transaldolase	TALDO1	1	13	13	36.5	37.54	3.96E+08	2.87E+08	3.83E+08	3.14E+08	2.92E+08	2.27E+08	0.781002	0.14569
Q9Y5Z4	Heme-binding protein 2	HEBP2	1	4	4	30.7	22.875	46728000	34704000	43704000	39720000	29740000	27459000	0.774509	0.145732
Q92499	ATP-dependent RNA helicase DDX1	DDX1	1	25	25	44.2	82.431	98286000	99348000	94565000	92127000	90098000	96760000	0.954777	0.146282
Q14738	Serine/threonine-protein phosphatase 2A !	PPP2R5D	1	5	3	12	69.991	13731000	15099000	13119000	7747100	13266000	11597000	0.777375	0.147362
P09493	Tropomyosin alpha-1 chain	TPM1	1	20	7	50.4	32.708	2.07E+08	1.1E+08	1.78E+08	1.19E+08	1.26E+08	63035000	0.620869	0.147628
Q04206	Transcription factor p65	RELA	1	11	11	26.1	60.218	33588000	41528000	48722000	33156000	33718000	33460000	0.810204	0.147717
P08243	Asparagine synthetase [glutamine-hydroly:	ASNS	1	6	6	16.2	64.369	12887000	NaN	14362000	12229000	9376900	11857000	0.818694	0.149129
O95674;Q92903	Phosphatidate cytidylyltransferase 2	CDS2	2	3	3	11.9	51.417	24541000	20326000	21371000	15956000	21168000	18610000	0.84142	0.149712
P61086	Ubiquitin-conjugating enzyme E2 K	UBE2K	1	6	6	33	22.406	83667000	75040000	94964000	70824000	79308000	56283000	0.813711	0.15001
Q7Z2W4	Zinc finger CCCH-type antiviral protein 1	ZC3HAV1	1	11	11	19.2	101.43	17619000	20323000	20587000	15899000	15998000	19092000	0.871175	0.150037
Q8N0X7	Spartin	SPG20	1	8	8	17.7	72.832	31188000	30276000	31658000	22548000	27312000	30628000	0.864329	0.151505
Q13637	Ras-related protein Rab-32	RAB32	1	5	4	26.2	24.997	15516000	16212000	NaN	26741000	24224000	17390000	1.436271	0.151521
P46459	Vesicle-fusing ATPase	NSF	1	18	18	30.2	82.593	70818000	64202000	68663000	64005000	56643000	65342000	0.913135	0.151544
P29966	Myristoylated alanine-rich C-kinase substra	MARCKS	1	5	5	29.8	31.554	35955000	42433000	45642000	46211000	50479000	71538000	1.356349	0.151714
O43765	Small glutamine-rich tetratricopeptide repe	SGTA	1	5	5	18.2	34.063	46876000	46318000	37543000	51605000	48429000	47845000	1.131118	0.152599
Q86V81	THO complex subunit 4	ALYREF	1	7	7	36.2	26.888	69858000	85975000	76035000	51381000	56772000	76444000	0.79613	0.153016
Q14157	Ubiquitin-associated protein 2-like	UBAP2L	1	13	13	19.2	114.53	46028000	38071000	47988000	34207000	39304000	39923000	0.858782	0.15314
P47897	Glutamine--tRNA ligase	QARS	1	17	17	28.6	87.798	42560000	47455000	44734000	41469000	40676000	43721000	0.934077	0.153575
P05023;P13637;P50993;Q1373	Sodium/potassium-transporting ATPase su	ATP1A1	6	31	31	37.7	112.89	87913000	78281000	80105000	70337000	75005000	79770000	0.913979	0.153604
Q9GZS3	WD repeat-containing protein 61;WD repe	WDR61	1	5	5	18.4	33.58	42882000	37972000	NaN	32679000	35519000	NaN	0.843471	0.155369
Q9P0V9	Septin-10	10-Sep	1	5	4	15.9	52.592	9974400	10487000	10535000	10461000	12306000	14680000	1.208108	0.156443
P62314	Small nuclear ribonucleoprotein Sm D1	SNRPD1	1	4	4	37	13.281	64543000	57305000	79150000	53766000	58358000	54489000	0.828929	0.156532
P35998	26S protease regulatory subunit 7	PSMC2	1	20	20	53.3	48.633	82814000	81573000	94068000	73740000	82148000	78233000	0.905848	0.156578
Q9Y5K8	V-type proton ATPase subunit D	ATP6V1D	1	4	4	32.4	28.262	14831000	18094000	21989000	22491000	20305000	26607000	1.263849	0.156622
P26447	Protein S100-A4	S100A4	1	5	5	36.6	11.728	9.21E+08	8.33E+08	8.53E+08	8.69E+08	7.3E+08	7.27E+08	0.892015	0.156818
P00387	NADH-cytochrome b5 reductase 3;NADH-	CYB5R3	1	19	19	66.8	34.234	6.52E+08	7.35E+08	7.01E+08	5.75E+08	6.1E+08	6.88E+08	0.897305	0.157662
Q9BYD1	39S ribosomal protein L13, mitochondrial	MRPL13	1	3	3	22.5	20.692	9167100	13140000	11708000	12549000	13546000	16225000	1.244153	0.158281
Q13428	Treacle protein	TCOF1	1	10	10	8.9	152.1	17986000	17523000	20319000	16391000	6728200	NaN	0.621172	0.158289
P08238;Q58FF7	Heat shock protein HSP 90-beta	HSP90AB1	2	38	23	56.2	83.263	9.03E+08	7.5E+08	9.53E+08	7.11E+08	8.09E+08	7.35E+08	0.865182	0.158368
Q9P0L0	Vesicle-associated membrane protein-assc	VAPA	1	6	5	32.1	27.893	63745000	84487000	70421000	50528000	60052000	67438000	0.814158	0.158747
Q8IWE2	Protein NOXP20	FAM114A1	1	11	11	30.7	60.741	2.98E+08	5.26E+08	4.56E+08	2.9E+08	3.28E+08	3.09E+08	0.723889	0.159216
A0A8I5KQE6;P08865	40S ribosomal protein SA	RPSA	2	11	11	44.7	32.909	2.98E+08	3.15E+08	3.2E+08	2.56E+08	2.59E+08	3.14E+08	0.888516	0.159372
Q15404	Ras suppressor protein 1	RSU1	1	8	8	31	31.54	1.17E+08	1.67E+08	1.13E+08	88616000	84665000	1.19E+08	0.734742	0.160123
P26038	Moesin	MSN	1	39	28	53.7	67.819	5.1E+08	5.91E+08	5.3E+08	4.92E+08	5.03E+08	5.08E+08	0.921432	0.16038
P60900	Proteasome subunit alpha type-6	PSMA6	1	11	11	49.2	27.399	1.17E+08	1.42E+08	1.46E+08	1.2E+08	1.16E+08	1.22E+08	0.88297	0.161048
Q02978	Mitochondrial 2-oxoglutarate/malate carrie	SLC25A11	1	7	7	23.6	34.061	90149000	1.06E+08	99061000	1.11E+08	1.01E+08	1.13E+08	1.102025	0.161123
P37235;P84074;P61601	Hippocalcin-like protein 1	HPCAL1	3	7	7	38.9	22.313	1.01E+08	87280000	1.08E+08	84326000	80965000	93916000	0.876143	0.16161
P40939	Trifunctional enzyme subunit alpha, mitoch	HADHA	1	25	25	44.4	82.999	2.94E+08	2.8E+08	3.03E+08	2.94E+08	3.19E+08	3.24E+08	1.068246	0.161736
P47985;P0C7P4	Cytochrome b-c1 complex subunit Rieske,	UQCRFS1;UQCRFS	2	6	6	28.1	29.668	85418000	85248000	72995000	73950000	69831000	76502000	0.904055	0.16186
Q9NUU7;Q9UMR2	ATP-dependent RNA helicase DDX19A;ATI	DDX19A;DDX19B	2	9	9	27.6	53.974	23835000	24605000	23253000	25243000	24557000	28579000	1.093259	0.162229
P80723	Brain acid soluble protein 1	BASP1	1	9	9	57.3	22.693	2.62E+08	2.37E+08	2.35E+08	1.99E+08	2.16E+08	2.41E+08	0.894283	0.162523
Q16822	Phosphoenolpyruvate carboxykinase [GTP],	PCK2	1	7	7	13.9	70.698	19143000	22572000	18240000	24325000	20442000	26625000	1.19076	0.163294
P42766	60S ribosomal protein L35	RPL35	1	4	4	26	14.551	1.02E+08	1.12E+08	68796000	1.77E+08	1.13E+08	1.17E+08	1.444319	0.163617
O75431	Metaxin-2	MTX2	1	3	3	22.8	29.763	24072000	25854000	28504000	22938000	24167000	24366000	0.911271	0.163944
Q9Y4P3	Transducin beta-like protein 2	TBL2	1	5	5	13	49.797	27292000	26566000	29939000	24144000	26121000	14527000	0.773202	0.164203
P08559;P29803	Pyruvate dehydrogenase E1 component su	PDHA1	2	8	8	29.2	43.295	61583000	54442000	61845000	63032000	62242000	67990000	1.086546	0.16447
O95747	Serine/threonine-protein kinase OSR1	OXSR1	1	8	6	21.8	58.022	31175000	31924000	29117000	24695000	28976000	29605000	0.903054	0.164934
Q9Y4E1;Q641Q2	WASH complex subunit FAM21C;WASH co	FAM21C;FAM21A	2	11	11	12.3	147.15	15349000	17611000	17345000	15225000	14054000	16206000	0.904184	0.164948
O15511	Actin-related protein 2/3 complex subunit	ARPC5	1	5	5	57	16.32	1.34E+08	1.51E+08	1.18E+08	1.53E+08	1.48E+08	1.51E+08	1.122183	0.165746
P61586	Transforming protein RhoA	RHOA	1	7	3	38.3	21.768	2.26E+08	2.94E+08	1.69E+08	3.17E+08	2.72E+08	2.95E+08	1.283085	0.165883
Q13907;Q9BXS1	Isopentenyl-diphosphate Delta-isomerase	IDI1	2	4	4	17.2	26.319	33839000	35765000	32505000	24267000	33788000	29310000	0.855605	0.16632
O14980	Exportin-1	XPO1	1	17	17	22.8	123.38	93063000	1.1E+08	1.1E+08	94143000	88176000	98394000	0.897325	0.166775
Q9Y5L4	Mitochondrial import inner membrane tran	TIMM13	1	4	4	53.7	10.5	42408000	41945000	45158000	47403000	44775000	44344000	1.054134	0.166912
Q16134	Electron transfer flavoprotein-ubiquinone c	ETFDH	1	5	5	9.7	68.495	15742000	13221000	12510000	20222000	17312000	14130000	1.245726	0.166941
O75643	U5 small nuclear ribonucleoprotein 200 kD	SNRNP200	1	23	23	15	244.5	75907000	72460000	60474000	55964000	56365000	66828000	0.857863	0.167456
P09417	Dihydropteridine reductase	QDPR	1	4	4	25.4	25.789	24892000	26238000	29380000	36594000	26067000	38619000	1.25798	0.167497
P12110	Collagen alpha-2(VI) chain	COL6A2	1	5	5	5.6	108.58	21138000	28981000	19213000	18498000	18679000	16770000	0.778097	0.167826
Q9Y277	Voltage-dependent anion-selective channe	VDAC3	1	7	7	37.1	30.658	82626000	70712000	92112000	98222000	1.27E+08	87527000	1.275694	0.168077

P22314	Ubiquitin-like modifier-activating enzyme 1	UBA1	1	36	36	45.9	117.85	3.19E+08	3.01E+08	3.27E+08	2.88E+08	3.12E+08	2.95E+08	0.944367	0.16862
O00629	Importin subunit alpha-3	KPNA4	1	10	7	31.7	57.886	42948000	46627000	50965000	41123000	41602000	44792000	0.907336	0.168686
O75116	Rho-associated protein kinase 2	ROCK2	1	17	14	15.1	160.9	29527000	37378000	30377000	28338000	28354000	28083000	0.871436	0.169253
Q96S66	Chloride channel CLIC-like protein 1	CLCC1	1	8	8	19.4	62.022	21756000	21179000	21440000	24791000	21751000	30126000	1.190959	0.170202
P22059	Oxysterol-binding protein 1	OSBP	1	12	12	18.5	89.42	34812000	35855000	36142000	43078000	41041000	35004000	1.11529	0.170213
P54920;Q9H115	Alpha-soluble NSF attachment protein	NAPA	2	12	12	60	33.232	47420000	43397000	52814000	40691000	43835000	44305000	0.896958	0.170291
P49588	Alanine--tRNA ligase, cytoplasmic	AARS	1	19	19	25.8	106.81	35946000	36018000	35711000	39774000	35446000	39368000	1.064202	0.171029
Q9UL46	Proteasome activator complex subunit 2	PSME2	1	9	9	43.5	27.401	2.29E+08	2.05E+08	1.99E+08	1.86E+08	1.97E+08	2E+08	0.920592	0.171429
Q9UBB4	Ataxin-10	ATXN10	1	12	12	29.3	53.488	27251000	23495000	29575000	38203000	33541000	27017000	1.229579	0.171642
Q5EBL4	RILP-like protein 1	RILPL1	1	6	6	19.6	47.107	18915000	14291000	14290000	12376000	14372000	12374000	0.82369	0.171765
P40616	ADP-ribosylation factor-like protein 1	ARL1	1	4	4	34.8	20.417	96689000	1.17E+08	1.07E+08	1.57E+08	1.08E+08	1.34E+08	1.240429	0.171954
P19784	Casein kinase II subunit alpha	CSNK2A2	1	11	11	42	41.213	34836000	38541000	38012000	27354000	30588000	37509000	0.856916	0.173353
Q8TED1	Probable glutathione peroxidase 8	GPX8	1	5	5	35.9	23.881	50787000	44868000	63491000	41401000	48040000	39648000	0.811136	0.17356
P35914;Q8TB92	Hydroxymethylglutaryl-CoA lyase, mitocho	HMGCL	2	6	6	22.2	34.36	43051000	44514000	41495000	46748000	50708000	43197000	1.089826	0.17369
Q9NR45	Sialic acid synthase	NANS	1	11	11	40.1	40.307	1.57E+08	1.77E+08	1.72E+08	1.43E+08	1.47E+08	1.67E+08	0.903606	0.174
Q9P253	Vacuolar protein sorting-associated protein	VPS18	1	5	5	8.5	110.18	9053600	11842000	10116000	12044000	10940000	13889000	1.189007	0.174045
P49720	Proteasome subunit beta type-3	PSMB3	1	5	5	24.4	22.949	1.05E+08	1.3E+08	1.37E+08	75069000	80257000	1.25E+08	0.752209	0.174388
P62140	Serine/threonine-protein phosphatase PP1	PPP1CB	1	11	3	38.8	37.186	98766000	63265000	33677000	1.18E+08	1.47E+08	72636000	1.725407	0.174847
Q5JTZ9	Alanine--tRNA ligase, mitochondrial	AARS2	1	5	5	7.3	107.34	11348000	10877000	15933000	13826000	16875000	16785000	1.244457	0.176782
O14974	Protein phosphatase 1 regulatory subunit 1	PPP1R12A	1	20	20	26.8	115.28	94255000	1.03E+08	89617000	91683000	89251000	81207000	0.9136	0.177502
P61353	60S ribosomal protein L27	RPL27	1	4	4	30.9	15.798	1.36E+08	1.47E+08	2.29E+08	2.73E+08	2.32E+08	1.91E+08	1.360671	0.177697
Q07960	Rho GTPase-activating protein 1	ARHGAP1	1	13	13	38	50.435	1.48E+08	1.54E+08	1.51E+08	1.3E+08	1.44E+08	1.5E+08	0.934835	0.177753
P21283	V-type proton ATPase subunit C 1	ATP6V1C1	1	8	8	26.4	43.941	47748000	31276000	47959000	52004000	58924000	47553000	1.248049	0.178404
P10599	Thioredoxin	TXN	1	7	7	80	11.737	8.75E+08	7.87E+08	9.64E+08	9.59E+08	9.49E+08	9.7E+08	1.095919	0.178596
P56192	Methionine--tRNA ligase, cytoplasmic	MARS	1	16	16	22.8	101.11	42586000	42427000	38660000	37776000	39391000	39624000	0.944353	0.178697
P60983	Glia maturation factor beta	GMFB	1	3	3	31	16.713	90338000	1.31E+08	81468000	57138000	78514000	83143000	0.723347	0.178747
P35813	Protein phosphatase 1A	PPM1A	1	4	3	13.4	42.447	NaN	67808000	75100000	89024000	1.04E+08	76731000	1.259108	0.17877
P61011	Signal recognition particle 54 kDa protein	SRP54	1	8	8	21.8	55.704	35233000	36560000	33719000	28309000	32166000	34903000	0.903954	0.179906
P07858	Cathepsin B;Cathepsin B light chain;Cathep	CTSB	1	11	11	41	37.821	5.48E+08	5.23E+08	4.93E+08	6.2E+08	6.13E+08	5.14E+08	1.117385	0.179993
O14744	Protein arginine N-methyltransferase 5;Pro	PRMT5	1	7	7	14.1	72.683	NaN	53847000	51374000	42209000	48800000	NaN	0.864932	0.180963
P35637	RNA-binding protein FUS	FUS	1	7	5	15.8	53.425	2.04E+08	1.48E+08	1.66E+08	1.22E+08	1.5E+08	1.53E+08	0.820512	0.181033
Q9Y5X3	Sorting nexin-5	SNX5	1	4	4	16.6	46.816	NaN	26863000	27768000	51568000	31816000	37871000	1.479685	0.181066
P13489	Ribonuclease inhibitor	RNH1	1	17	17	52.3	49.973	2.28E+08	2.42E+08	2.17E+08	1.94E+08	2.13E+08	2.25E+08	0.919594	0.181714
Q7L523;Q5VZM2	Ras-related GTP-binding protein A;Ras-rel	RRAGA;RRAGB	2	4	4	16.9	36.566	22657000	21030000	19673000	19217000	19168000	NaN	0.908736	0.181946
P63241;Q6IS14;Q9GZV4	Eukaryotic translation initiation factor 5A-1	EIF5A;EIF5AL1	3	11	11	82.5	16.832	5.13E+08	4.36E+08	5.46E+08	3.73E+08	4.77E+08	4.29E+08	0.856365	0.182243
P17844	Probable ATP-dependent RNA helicase DD	DDX5	1	15	9	26.9	69.147	67995000	53613000	60314000	50090000	57010000	52478000	0.877178	0.182531
Q9NP72	Ras-related protein Rab-18	RAB18	1	8	8	49	22.977	27929000	29278000	30008000	28872000	26569000	27425000	0.950135	0.18519
O00233	26S proteasome non-ATPase regulatory su	PSMD9	1	9	9	41.3	24.682	56360000	62035000	46655000	61675000	63282000	61696000	1.130888	0.186486
Q16718	NADH dehydrogenase [ubiquinone] 1 alph	NDUFA5	1	5	5	65.5	13.459	39489000	42342000	36078000	33403000	36350000	37639000	0.910804	0.186639
Q7Z4Q2	HEAT repeat-containing protein 3	HEATR3	1	4	4	8.8	74.582	7616400	10429000	9172300	9469900	11523000	11112000	1.17956	0.18793
Q9NQW7	Xaa-Pro aminopeptidase 1	XPNPEP1	1	6	6	12.5	69.917	26948000	38418000	31169000	25749000	27148000	27552000	0.833366	0.18925
Q6DKI1	60S ribosomal protein L7-like 1	RPL7L1	1	3	3	14.1	29.669	7885700	6606500	8348300	NaN	6496700	6454400	0.850535	0.189285
Q9H299	SH3 domain-binding glutamic acid-rich-lik	SH3BGRL3	1	4	4	67.7	10.438	1.59E+09	2.05E+09	1.8E+09	1.33E+09	1.32E+09	1.81E+09	0.818708	0.189401
P09429;B2RPK0	High mobility group protein B1	HMGB1	2	7	6	26	24.893	3.68E+08	3.14E+08	3.87E+08	3.4E+08	3.16E+08	2.82E+08	0.877629	0.189799
P23219	Prostaglandin G/H synthase 1	PTGS1	1	5	5	12.7	68.686	58837000	43777000	73429000	46268000	47400000	40844000	0.764086	0.190633
Q12765	Secernin-1	SCRN1	1	15	15	38.6	46.382	95807000	1.05E+08	83462000	81423000	88158000	83762000	0.890394	0.191038
Q9Y5M8	Signal recognition particle receptor subunit	SRPRB	1	10	10	47.6	29.702	30002000	30406000	20618000	23160000	21729000	20720000	0.809728	0.191679
O60232	Sjoegren syndrome/scleroderma autoantig	SSSCA1	1	3	3	31.7	21.474	11774000	11398000	16061000	11395000	10861000	9403100	0.806951	0.191871
Q12907	Vesicular integral-membrane protein VIP36	LMAN2	1	10	10	35.4	40.228	1.33E+08	1.4E+08	1.65E+08	1.08E+08	1.24E+08	1.41E+08	0.852848	0.192243
Q5JRX3	Presequence protease, mitochondrial	PITRM1	1	18	18	26.8	117.41	73895000	82174000	74713000	72177000	70198000	74640000	0.940346	0.192433
P17931	Galectin-3	LGALS3	1	5	5	22.4	26.152	1.51E+08	1.43E+08	1.65E+08	2.44E+08	1.99E+08	1.49E+08	1.289014	0.193107
Q9UKK9	ADP-sugar pyrophosphatase	NUDT5	1	9	9	40.2	24.327	90347000	95123000	1.09E+08	84604000	88449000	92804000	0.901332	0.193926
Q9Y394	Dehydrogenase/reductase SDR family mem	DHRS7	1	7	7	22.7	38.298	70164000	78652000	76406000	82187000	82806000	75901000	1.069585	0.195342
Q9Y4W2	Ribosomal biogenesis protein LAS1L	LAS1L	1	2	2	4.2	83.064	NaN	9432500	9715900	NaN	8021800	9062400	0.8922	0.195734
O96005	Cleft lip and palate transmembrane protein	CLPTM1	1	4	4	12.6	76.096	24679000	28154000	25994000	19251000	21358000	26781000	0.85491	0.196287
O95433	Activator of 90 kDa heat shock protein ATP	AHSA1	1	8	8	41.7	38.274	45180000	48910000	51704000	33250000	43935000	47193000	0.853108	0.196856
Q92538	Golgi-specific brefeldin A-resistance guani	GBF1	1	18	18	15.8	206.44	30124000	34943000	35129000	31157000	30376000	30988000	0.9234	0.19711
Q96CV9	Optineurin	OPTN	1	9	9	18.4	65.921	33792000	34043000	33383000	39629000	36504000	33428000	1.082426	0.197314
P09012	U1 small nuclear ribonucleoprotein A	SNRPA	1	3	3	16.7	31.279	42559000	58960000	63578000	46756000	43858000	44679000	0.819476	0.197536

P61201	COP9 signalosome complex subunit 2	COPS2	1	8	8	25.7	51.596	56450000	56865000	46171000	57093000	57537000	64456000	1.122895	0.197571
Q6WCQ1	Myosin phosphatase Rho-interacting prote	MPRIP	1	26	26	35.7	116.53	45288000	44205000	47769000	51513000	46016000	48557000	1.064286	0.197795
Q9UHD2	Serine/threonine-protein kinase TBK1	TBK1	1	3	3	7.5	83.641	NaN	18992000	17148000	NaN	20988000	26909000	1.325318	0.198448
P62805	Histone H4	HIST1H4A	1	9	9	53.4	11.367	2.7E+09	2.89E+09	2.6E+09	3.5E+09	2.93E+09	2.81E+09	1.12933	0.19878
Q07866;Q9NSK0;Q6P597	Kinesin light chain 1	KLC1	3	11	10	23.2	65.309	47199000	42479000	53693000	39775000	45484000	41168000	0.881817	0.199333
P61619;Q9H9S3	Protein transport protein Sec61 subunit alp	SEC61A1;SEC61A2	2	3	3	5.9	52.264	1.87E+08	1.94E+08	1.26E+08	1.15E+08	1.62E+08	50783000	0.647408	0.199337
O00273	DNA fragmentation factor subunit alpha	DFFA	1	6	6	23.9	36.521	16929000	14061000	13250000	17690000	25409000	15708000	1.329272	0.199499
Q96FJ2	Dynein light chain 2, cytoplasmic	DYNLL2	1	3	1	49.4	10.35	17522000	21042000	21685000	32411000	18127000	32508000	1.37838	0.199519
P63220	40S ribosomal protein S21	RPS21	1	5	5	53	9.1113	1.42E+08	1.29E+08	1.61E+08	1.37E+08	1.29E+08	1.11E+08	0.872245	0.199593
P30622	CAP-Gly domain-containing linker protein	CLIP1	1	10	8	9.5	162.24	15357000	18577000	18361000	20032000	18486000	18995000	1.09978	0.199987
Q14320	Protein FAM50A	FAM50A	1	3	3	14.2	40.241	25577000	27795000	27241000	33287000	33908000	25794000	1.153524	0.200069
Q14974	Importin subunit beta-1	KPNB1	1	20	20	35	97.169	1.9E+08	2.24E+08	2.25E+08	1.8E+08	1.92E+08	2.05E+08	0.903141	0.200938
O43491	Band 4.1-like protein 2	EPB41L2	1	16	15	24.2	112.59	46929000	42409000	39073000	39094000	38439000	40184000	0.916721	0.201187
Q9BUF5	Tubulin beta-6 chain	TUBB6	1	21	8	66.6	49.857	3.33E+08	4.13E+08	3.28E+08	4.01E+08	3.8E+08	4.68E+08	1.163187	0.201622
O43670	BUB3-interacting and GLEBS motif-contain	ZNF207	1	3	3	7.9	50.75	41754000	21337000	34299000	18117000	25216000	24799000	0.699579	0.201822
Q5T1M5	FK506-binding protein 15	FKBP15	1	5	5	6.3	133.63	10202000	17185000	12715000	11754000	9022600	9136900	0.745935	0.202172
Q13409	Cytoplasmic dynein 1 intermediate chain 2	DYNC1I2	1	11	11	25.4	71.456	81444000	88117000	96352000	93746000	94188000	1.13E+08	1.130949	0.202212
P29692	Elongation factor 1-delta	EEF1D	1	13	13	63.7	31.121	4.14E+08	3.34E+08	3.95E+08	3.49E+08	3.58E+08	2.94E+08	0.875346	0.202248
Q12846	Syntaxin-4	STX4	1	3	3	14.8	34.18	24372000	20245000	26028000	20366000	21924000	20076000	0.882808	0.202561
P30711	Glutathione S-transferase theta-1	GSTT1	1	5	5	25.8	27.335	40871000	40983000	44183000	33823000	37590000	42354000	0.902648	0.203925
Q9BT78	COP9 signalosome complex subunit 4	COPS4	1	10	10	34.2	46.268	31810000	32103000	31238000	23858000	25929000	32889000	0.868893	0.204134
O75822	Eukaryotic translation initiation factor 3 sub	EIF3J	1	4	4	27.1	29.062	14972000	18843000	19322000	16851000	13890000	15018000	0.861151	0.204744
Q9Y224	UPF0568 protein C14orf166	C14orf166	1	9	9	43.4	28.068	1.02E+08	94106000	1.04E+08	99369000	90099000	75153000	0.884588	0.205209
Q96S52	GPI transamidase component PIG-S	PIGS	1	3	3	7.9	61.655	12626000	16158000	13761000	10923000	12929000	13037000	0.867058	0.20525
Q16643	Drebrin	DBN1	1	16	16	36.8	71.428	1.57E+08	1.65E+08	1.58E+08	1.37E+08	1.5E+08	1.6E+08	0.931222	0.20544
Q8NCC3	Group XV phospholipase A2	PLA2G15	1	3	3	10.2	46.657	8209300	12630000	10987000	13445000	11280000	14111000	1.220249	0.205571
P18084	Integrin beta-5	ITGB5	1	7	7	17.3	88.053	28387000	30395000	25322000	17622000	20545000	29053000	0.799249	0.206077
P48739	Phosphatidylinositol transfer protein beta is	PITPNB	1	11	11	42.1	31.54	53690000	48529000	47507000	44656000	48925000	45725000	0.930406	0.206093
P26599	Polypyrimidine tract-binding protein 1	PTBP1	1	10	10	32.3	59.632	2.46E+08	2.45E+08	2.44E+08	1.99E+08	1.95E+08	2.54E+08	0.882471	0.206237
Q16563	Synaptophysin-like protein 1	SYPL1	1	3	3	19.7	28.565	20803000	20102000	30906000	20223000	19317000	14706000	0.7554	0.206355
O43242	26S proteasome non-ATPase regulatory su	PSMD3	1	15	15	30	60.977	1.02E+08	1.12E+08	1.01E+08	1.34E+08	1.17E+08	1.05E+08	1.127052	0.206751
O94760	N(G),N(G)-dimethylarginine dimethylaminc	DDAH1	1	11	11	57.2	31.121	44357000	47055000	38757000	44181000	60024000	49634000	1.181841	0.207321
P30533	Alpha-2-macroglobulin receptor-associate	LRPAP1	1	11	11	33.6	41.465	1.46E+08	1.3E+08	1.7E+08	1.26E+08	1.25E+08	1.39E+08	0.874437	0.207716
Q9NQG5	Regulation of nuclear pre-mRNA domain-c	RPRD1B	1	2	2	9.5	36.899	21503000	23065000	15070000	12386000	19448000	12664000	0.746135	0.207968
O95197	Reticulon-3	RTN3	1	2	2	4.8	112.61	57138000	NaN	58380000	66671000	94929000	68335000	1.326979	0.208597
P08579	U2 small nuclear ribonucleoprotein B	SNRPB2	1	4	4	24.9	25.486	66835000	56625000	1.1E+08	48561000	49546000	59975000	0.676229	0.208877
O95793	Double-stranded RNA-binding protein Sta	STAU1	1	9	9	23.1	63.182	27988000	24052000	25527000	25127000	22000000	23888000	0.915531	0.210133
Q01105;P0DME0	Protein SET;Protein SETSIP	SET;SETSIP	2	7	7	24.1	33.488	2.15E+08	1.8E+08	2.48E+08	1.91E+08	1.81E+08	1.82E+08	0.860767	0.210208
Q7KZI7;Q96L34;P27448;Q9P0L	Serine/threonine-protein kinase MARK2;M/	MARK2;MARK4;M/	4	2	2	3.7	87.91	NaN	11816000	12399000	27000000	16152000	15810000	1.623291	0.210223
O14618	Copper chaperone for superoxide dismuta:	CCS	1	3	3	12.8	29.04	1.43E+08	1.51E+08	1.45E+08	1.19E+08	1.43E+08	NaN	0.897853	0.211333
P00403	Cytochrome c oxidase subunit 2	MT-CO2	1	3	3	14.5	25.565	76744000	82959000	63753000	76712000	88336000	89437000	1.13886	0.212
Q14697	Neutral alpha-glucosidase AB	GANAB	1	27	27	42.5	106.87	2.53E+08	2.38E+08	2.64E+08	2.15E+08	2.2E+08	2.55E+08	0.914022	0.212883
P82909	28S ribosomal protein S36, mitochondrial	MRPS36	1	3	3	40.8	11.466	16220000	22396000	21301000	14264000	15626000	19279000	0.820619	0.213028
Q7L1Q6	Basic leucine zipper and W2 domain-conta	BZW1	1	8	8	19.8	48.043	61000000	53966000	67558000	78519000	76886000	59387000	1.176788	0.213254
Q9NVI7;Q5T2N8	ATPase family AAA domain-containing pro	ATAD3A	2	9	4	17.4	71.368	33264000	28846000	33898000	42640000	32776000	35428000	1.154529	0.213728
O15269	Serine palmitoyltransferase 1	SPTLC1	1	5	5	15.4	52.743	31648000	40408000	32457000	32554000	27021000	30622000	0.863022	0.213761
O75821	Eukaryotic translation initiation factor 3 sub	EIF3G	1	7	7	34.4	35.611	45940000	44338000	55908000	31627000	39817000	48196000	0.818409	0.21418
O15431	High affinity copper uptake protein 1	SLC31A1	1	2	2	15.8	21.09	19373000	24636000	NaN	20075000	18246000	16013000	0.823074	0.21436
Q96GA7	Serine dehydratase-like	SDSL	1	3	3	13.7	34.674	15501000	17028000	16259000	NaN	16807000	17949000	1.068582	0.214547
Q9BWF3;Q9BQ04	RNA-binding protein 4;RNA-binding prote	RBM4;RBM4B	2	4	4	18.4	40.313	22681000	19363000	20664000	19011000	18892000	NaN	0.906655	0.215588
P51688	N-sulphoglucosamine sulphohydrolase	SGSH	1	8	8	24.9	56.695	46138000	60933000	43706000	43424000	41871000	41658000	0.841992	0.216159
P53618	Coatomer subunit beta	COPB1	1	33	33	46	107.14	1.71E+08	1.87E+08	1.72E+08	1.41E+08	1.6E+08	1.78E+08	0.903418	0.216878
Q99805	Transmembrane 9 superfamily member 2	TM9SF2	1	9	9	17.8	75.775	42938000	50958000	41040000	36345000	34172000	44862000	0.855065	0.217526
P62877	E3 ubiquitin-protein ligase RBX1;E3 ubiquit	RBX1	1	2	2	24.1	12.274	NaN	28989000	25599000	51636000	36166000	31360000	1.455289	0.218057
P43243	Matrin-3	MATR3	1	18	18	25.1	94.622	1.56E+08	1.59E+08	1.9E+08	1.48E+08	1.47E+08	1.59E+08	0.900427	0.21828
O95168	NADH dehydrogenase [ubiquinone] 1 beta	NDUFB4	1	2	2	21.7	15.208	25068000	NaN	27240000	25330000	22857000	24510000	0.926525	0.218883
O60313	Dynamin-like 120 kDa protein, mitochondr	OPA1	1	6	6	8.3	111.63	11641000	9044400	11881000	17063000	10538000	14106000	1.280676	0.219277
P08240	Signal recognition particle receptor subunit	SRPR	1	7	7	14.3	69.81	26104000	25000000	29662000	24033000	26018000	23882000	0.915398	0.219486
Q2M2I8	AP2-associated protein kinase 1	AAK1	1	9	9	13.9	103.88	20664000	17580000	22156000	25667000	21314000	21813000	1.138974	0.219825

Q9UHB9	Signal recognition particle subunit SRP68	SRP68	1	16	16	38.1	70.729	51972000	59417000	58706000	47544000	52621000	55474000	0.915012	0.219989
P99999;CON__P62894	Cytochrome c	CYCS	2	4	4	48.6	11.749	98747000	76008000	51938000	1.08E+08	1.75E+08	80113000	1.599577	0.220396
P16152	Carbonyl reductase [NADPH] 1	CBR1	1	12	10	59.2	30.375	92090000	99135000	1.01E+08	94383000	93216000	92557000	0.960112	0.220472
O60832	H/ACA ribonucleoprotein complex subunit	DKC1	1	7	7	18.1	57.673	26937000	31230000	35096000	24767000	27543000	29245000	0.874463	0.220962
Q96KR1	Zinc finger RNA-binding protein	ZFR	1	7	7	9.5	117.01	NaN	11859000	12412000	11724000	11144000	NaN	0.942194	0.222094
Q9H9J2	39S ribosomal protein L44, mitochondrial	MRPL44	1	4	4	17.2	37.535	14774000	13579000	15146000	14588000	16282000	20470000	1.180257	0.22242
Q96HY6	DDRGK domain-containing protein 1	DDRGK1	1	3	3	20.4	35.61	NaN	29307000	23177000	14039000	21367000	23423000	0.747263	0.222988
P20073	Annexin A7	ANXA7	1	15	15	32.2	52.739	72378000	59439000	71014000	77419000	76955000	69419000	1.103347	0.223492
Q15050	Ribosome biogenesis regulatory protein hc	RRS1	1	3	3	11	41.193	12263000	14465000	12646000	12971000	16154000	22066000	1.300122	0.225286
O00754	Lysosomal alpha-mannosidase;Lysosomal a	MAN2B1	1	11	11	13.6	113.74	32868000	27802000	31951000	29210000	29257000	23047000	0.880081	0.225327
Q9Y6C9	Mitochondrial carrier homolog 2	MTCH2	1	8	8	38.9	33.331	82320000	88726000	67601000	87540000	89309000	88757000	1.112966	0.225415
O14735	CDP-diacylglycerol--inositol 3-phosphatid	CDIPT	1	3	3	15	23.539	15314000	13813000	NaN	18300000	15831000	NaN	1.171799	0.225446
Q6XQN6	Nicotinate phosphoribosyltransferase	NAPRT	1	4	4	8.6	57.578	14559000	16332000	9882900	8784500	12210000	10502000	0.772467	0.225991
P38646	Stress-70 protein, mitochondrial	HSPA9	1	28	27	53.5	73.68	2.81E+08	3.42E+08	2.95E+08	3.39E+08	3.15E+08	3.6E+08	1.104445	0.226082
O60493	Sorting nexin-3	SNX3	1	7	6	43.8	18.762	49977000	69982000	55289000	69343000	62461000	71726000	1.161383	0.226156
Q15813	Tubulin-specific chaperone E	TBCE	1	6	6	15.4	59.345	27620000	24966000	23783000	25162000	30822000	41626000	1.278136	0.226685
Q9UIQ6	Leucyl-cystinyl aminopeptidase;Leucyl-cyst	LNPEP	1	4	4	5	117.35	NaN	10149000	10872000	NaN	9023000	5055700	0.669745	0.227296
O15145	Actin-related protein 2/3 complex subunit	ARPC3	1	5	5	33.1	20.546	74973000	99158000	76237000	1.02E+08	86017000	1.04E+08	1.164753	0.227709
Q13155	Aminoacyl tRNA synthase complex-interac	AIMP2	1	3	3	17.2	35.348	26965000	25407000	30096000	21775000	25353000	26733000	0.895632	0.228702
P49915	GMP synthase [glutamine-hydrolyzing]	GMPS	1	11	11	20.3	76.715	26654000	27799000	26437000	26803000	24759000	26186000	0.961157	0.228948
O43252	Bifunctional 3-phosphoadenosine 5-phosp	PAPSS1	1	5	5	9.8	70.832	66027000	61615000	58643000	66740000	62517000	74374000	1.093115	0.229327
Q92616	Translational activator GCN1	GCN1L1	1	45	45	20.9	292.71	94314000	88506000	99947000	89813000	89490000	89437000	0.950394	0.230055
P46734;P52564	Dual specificity mitogen-activated protein I	MAP2K3	2	9	9	36	39.318	62413000	74187000	58104000	68516000	71049000	86205000	1.159555	0.230156
P12081	Histidine--tRNA ligase, cytoplasmic	HARS	1	12	8	29.1	57.41	65110000	73720000	71591000	74723000	72196000	75149000	1.055351	0.230629
Q92888	Rho guanine nucleotide exchange factor 1	ARHGEF1	1	8	8	12.3	102.43	16605000	20521000	21118000	19029000	14628000	16515000	0.861411	0.230646
P55263	Adenosine kinase	ADK	1	5	5	16	40.545	17722000	13214000	19013000	24872000	19404000	17573000	1.238243	0.230925
Q9H3H3	UPF0696 protein C11orf68	C11orf68	1	3	3	12.7	31.43	31813000	31752000	48793000	50498000	52154000	39231000	1.262776	0.231012
Q01995	Transgelin	TAGLN	1	18	17	76.6	22.611	1.33E+09	1.46E+09	1.26E+09	1.09E+09	1.17E+09	1.36E+09	0.895197	0.231131
Q5SSJ5	Heterochromatin protein 1-binding proteir	HP1BP3	1	12	12	23.1	61.206	50321000	53842000	50767000	49900000	50473000	41745000	0.917305	0.23127
Q9BSJ8	Extended synaptotagmin-1	ESYT1	1	23	23	28.1	122.85	67817000	64874000	63279000	64767000	69890000	74953000	1.069602	0.231626
Q8NEV1;P68400	Casein kinase II subunit alpha 3;Casein kina	CSNK2A3;CSNK2A	2	12	12	41.4	45.219	44279000	52224000	57109000	40252000	48356000	46191000	0.877529	0.231988
Q9NNW7	Thioredoxin reductase 2, mitochondrial	TXNRD2	1	5	5	14.1	56.506	12837000	13222000	NaN	22077000	21258000	12824000	1.436714	0.233154
Q15628	Tumor necrosis factor receptor type 1-assc	TRADD	1	6	6	24.7	34.247	14651000	10528000	11847000	13423000	14024000	15080000	1.148571	0.233754
Q92504	Zinc transporter SLC39A7	SLC39A7	1	1	1	3	50.117	1.27E+08	1.08E+08	1.11E+08	52974000	73153000	1.26E+08	0.726659	0.234071
Q8IVD9	NudC domain-containing protein 3	NUDCD3	1	4	4	17.5	40.822	10429000	9747400	9263400	9399300	12544000	11678000	1.142036	0.234613
P11177	Pyruvate dehydrogenase E1 component su	PDHB	1	5	5	17	39.233	32683000	38140000	41632000	46672000	51393000	36165000	1.193633	0.235222
Q9Y6H1;Q5T1J5	Coiled-coil-helix-coiled-coil-helix domain-	CHCHD2;CHCHD2	2	3	3	31.1	15.512	24724000	18298000	26384000	28588000	27644000	24634000	1.165115	0.235551
Q9HB40	Retinoid-inducible serine carboxypeptidase	SCPEP1	1	4	4	11.5	50.83	24351000	21438000	25729000	26989000	24202000	35601000	1.213569	0.236218
Q13435	Splicing factor 3B subunit 2	SF3B2	1	7	7	14.1	100.23	38898000	30856000	37972000	30069000	35309000	28943000	0.875564	0.236268
Q9Y281	Cofilin-2	CFL2	1	9	7	64.5	18.736	1.61E+08	1.25E+08	2.06E+08	1.87E+08	2.11E+08	1.96E+08	1.207616	0.236949
P49721	Proteasome subunit beta type-2	PSMB2	1	5	5	32.3	22.836	NaN	20055000	21099000	35115000	23994000	23887000	1.344478	0.238108
P50452	Serpin B8	SERPINB8	1	8	7	25.4	42.766	46876000	49239000	46645000	47835000	42790000	45169000	0.951205	0.238255
P20591	Interferon-induced GTP-binding protein M	MX1	1	29	26	52.3	75.519	1.59E+08	1.49E+08	1.63E+08	1.29E+08	1.34E+08	1.62E+08	0.901655	0.238436
Q96AC1	Fermitin family homolog 2	FERMT2	1	14	14	27.8	77.86	44165000	43070000	60935000	44585000	35921000	41512000	0.8235	0.239455
P21796	Voltage-dependent anion-selective channe	VDAC1	1	14	14	67.5	30.772	3.11E+08	3.63E+08	3.25E+08	3.08E+08	3.12E+08	3.15E+08	0.935204	0.239517
Q08722	Leukocyte surface antigen CD47	CD47	1	3	3	8.7	35.213	1.02E+08	80704000	1.36E+08	89139000	88780000	69860000	0.777556	0.240823
Q7Z739	YTH domain-containing family protein 3	YTHDF3	1	8	5	17.8	63.86	35941000	36643000	37424000	30946000	32818000	37745000	0.922742	0.243411
P62081	40S ribosomal protein S7	RPS7	1	9	9	53.6	22.127	2.66E+08	3.32E+08	2.64E+08	2.26E+08	2.57E+08	2.72E+08	0.876483	0.245271
Q9NWT6	Hypoxia-inducible factor 1-alpha inhibitor	HIF1AN	1	3	3	10.3	40.285	NaN	45073000	42461000	47951000	NaN	60706000	1.241312	0.246187
P46783;Q9NQ39	40S ribosomal protein S10;Putative 40S rib	RPS10;RPS10P5	2	6	6	34.5	18.898	3.82E+08	4.46E+08	3.8E+08	4.68E+08	4.7E+08	4E+08	1.106123	0.246273
P60033	CD81 antigen	CD81	1	3	3	25	25.809	1.1E+08	1.17E+08	99549000	80046000	78999000	1.15E+08	0.840026	0.246294
P62820	Ras-related protein Rab-1A	RAB1A	1	11	5	64.9	22.677	2.77E+08	3.31E+08	3.2E+08	2.48E+08	2.36E+08	3.19E+08	0.865972	0.246626
Q9Y570	Protein phosphatase methylesterase 1	PPME1	1	6	6	18.1	42.315	34632000	32300000	22258000	23765000	21316000	27214000	0.810573	0.247613
Q14764	Major vault protein	MVP	1	41	41	54.4	99.326	2.84E+08	2.97E+08	2.88E+08	2.88E+08	3E+08	3.06E+08	1.030174	0.248873
P21926	CD9 antigen	CD9	1	2	2	9.6	25.416	67004000	NaN	77798000	65582000	NaN	58012000	0.853538	0.248967
Q15287	RNA-binding protein with serine-rich doma	RNPS1	1	3	3	11.1	34.208	16870000	13774000	18545000	15647000	14491000	8824100	0.79209	0.249005
P63173	60S ribosomal protein L38	RPL38	1	5	5	50	8.2178	71238000	85073000	66252000	62568000	69012000	67013000	0.8923	0.249997
Q15631	Translin	TSN	1	7	7	39.5	26.183	65823000	63695000	64816000	64497000	67798000	66656000	1.023758	0.250613
P28062	Proteasome subunit beta type-8	PSMB8	1	7	7	27.2	30.354	43353000	35424000	38821000	42707000	42793000	41477000	1.079755	0.251749

Q9NW15	Anoctamin-10	ANO10	1	9	9	16.2	76.328	27551000	27557000	27573000	26918000	30793000	29556000	1.055466	0.251981
O00442	RNA 3-terminal phosphate cyclase	RTCA	1	4	4	17.2	39.336	26241000	33015000	32371000	26538000	25060000	29751000	0.887828	0.252532
P62854;Q5JNZ5	40S ribosomal protein S26;Putative 40S ribo	RPS26;RPS26P11	2	2	2	20.9	13.015	2.03E+08	2.46E+08	2.08E+08	1.96E+08	1.97E+08	2.09E+08	0.914406	0.252599
P24593	Insulin-like growth factor-binding protein 5	IGFBP5	1	1	1	4.4	30.57	NaN	15417000	23523000	12689000	NaN	13329000	0.668156	0.252966
Q8TDQ7	Glucosamine-6-phosphate isomerase 2	GNPDA2	1	7	4	38.8	31.084	15680000	16407000	16859000	22909000	14304000	24346000	1.257692	0.253402
P50213	Isocitrate dehydrogenase [NAD] subunit alp	IDH3A	1	10	10	28.1	39.591	67456000	67027000	84097000	79371000	76382000	95078000	1.147548	0.25389
Q9UGP8	Translocation protein SEC63 homolog	SEC63	1	10	10	16.4	87.996	30975000	27015000	28413000	27609000	26853000	27229000	0.945465	0.253917
Q8NFW8	N-acylneuraminate cytidylyltransferase	CMAS	1	10	10	35.3	48.379	27907000	19969000	31451000	21870000	23670000	19347000	0.817969	0.254242
Q53GS9	U4/U6.U5 tri-snRNP-associated protein 2	USP39	1	4	4	10.4	65.38	17688000	22632000	20226000	13442000	15862000	20855000	0.828444	0.254986
P54578	Ubiquitin carboxyl-terminal hydrolase 14	USP14	1	9	9	26.3	56.068	53586000	50388000	54724000	48180000	44881000	53919000	0.926162	0.255029
Q08AD1	Calmodulin-regulated spectrin-associated	CAMSAP2	1	6	6	5.7	168.09	25482000	23950000	16048000	18232000	18768000	16537000	0.817608	0.255099
Q9NSE4	Isoleucine--tRNA ligase, mitochondrial	IARS2	1	20	20	25	113.79	1.07E+08	91022000	1.01E+08	1.19E+08	1.14E+08	98170000	1.104302	0.25554
P55769	NHP2-like protein 1;NHP2-like protein 1, N	NHP2L1	1	5	5	55.5	14.173	42632000	27215000	37204000	51771000	51419000	33659000	1.278353	0.255594
Q99816	Tumor susceptibility gene 101 protein	TSG101	1	6	6	23.3	43.944	2.22E+08	2.65E+08	3.11E+08	2.2E+08	2.37E+08	2.37E+08	0.869433	0.255708
Q15006	ER membrane protein complex subunit 2	EMC2	1	5	5	20.5	34.833	23830000	23931000	29003000	20206000	20123000	26076000	0.865054	0.256041
Q9Y4E8	Ubiquitin carboxyl-terminal hydrolase 15	USP15	1	6	6	7.1	112.42	33608000	29668000	38787000	42967000	33794000	40385000	1.147781	0.256248
O76003	Glutaredoxin-3	GLRX3	1	10	10	38.5	37.432	1.59E+08	1.54E+08	1.45E+08	1.6E+08	1.66E+08	1.53E+08	1.047017	0.256435
P09622	Dihydrolipoyl dehydrogenase, mitochondri	DLD	1	11	11	27.9	54.177	1.41E+08	1.4E+08	1.38E+08	1.37E+08	1.5E+08	1.49E+08	1.039638	0.25696
P49773	Histidine triad nucleotide-binding protein 1	HINT1	1	5	5	50	13.802	86442000	97155000	84885000	87087000	74944000	85122000	0.920557	0.257486
Q9BTW9	Tubulin-specific chaperone D	TBCD	1	10	10	14.5	132.6	25474000	24426000	15044000	27587000	27458000	23873000	1.21517	0.257687
P23193;Q15560	Transcription elongation factor A protein 1	TCEA1	2	3	3	11.6	33.969	18201000	18729000	17638000	17100000	17534000	18178000	0.96782	0.257974
O95479	GDH/6PGL endoplasmic bifunctional protei	H6PD	1	11	11	18.3	88.892	30892000	35587000	26830000	33368000	34305000	36141000	1.112583	0.258091
Q8WXF7	Atlastin-1	ATL1	1	3	3	7.7	63.543	8369200	13583000	8280300	11795000	18358000	11378000	1.37372	0.258329
P26196	Probable ATP-dependent RNA helicase DD	DDX6	1	11	11	28	54.416	57991000	62486000	65906000	57554000	54862000	61858000	0.935032	0.258446
P25445	Tumor necrosis factor receptor superfamily	FAS	1	3	3	16.4	37.732	18590000	29009000	19700000	14980000	14178000	21902000	0.758704	0.258609
Q9P287	BRCA2 and CDKN1A-interacting protein	BCCIP	1	2	2	12.4	35.979	12926000	16310000	13786000	17619000	NaN	15378000	1.15047	0.259201
P62714	Serine/threonine-protein phosphatase 2A c	PPP2CB	1	8	2	36.2	35.575	63494000	75131000	73282000	56760000	54631000	73282000	0.871481	0.25934
O00410	Importin-5	IPO5	1	23	22	29.7	123.63	4.46E+08	4.39E+08	3.86E+08	4.24E+08	4.9E+08	4.64E+08	1.083371	0.26004
P05161	Ubiquitin-like protein ISG15	ISG15	1	5	5	35.8	17.887	1.41E+08	1.41E+08	1.57E+08	1.38E+08	1.14E+08	1.45E+08	0.903848	0.260355
Q9Y512	Sorting and assembly machinery componen	SAMM50	1	3	3	12.8	51.976	24584000	24233000	20012000	24494000	29505000	23891000	1.131645	0.260677
P55786;A6NEC2	Puromycin-sensitive aminopeptidase	NPEPPS	2	28	28	40.7	103.28	88957000	83761000	77941000	97480000	99980000	80399000	1.108514	0.260692
Q8TDZ2	Protein-methionine sulfoxide oxidase MICA	MICAL1	1	4	4	6.3	117.87	NaN	11753000	11517000	9038900	11833000	8597900	0.844286	0.260796
Q9BZE4	Nucleolar GTP-binding protein 1	GTPBP4	1	6	6	12.3	73.964	20830000	24903000	19110000	21731000	27638000	24977000	1.146554	0.261068
Q8TEX9	Importin-4	IPO4	1	4	4	6.1	118.71	9836700	10378000	NaN	10598000	15848000	12217000	1.275079	0.261313
P46379	Large proline-rich protein BAG6	BAG6	1	9	9	12.1	119.41	12481000	19275000	18039000	20795000	16537000	25551000	1.262838	0.261314
P13987	CD59 glycoprotein	CD59	1	3	3	18.8	14.177	3.02E+08	3.35E+08	2.75E+08	2.91E+08	2.62E+08	2.83E+08	0.917019	0.261662
P55010	Eukaryotic translation initiation factor 5	EIF5	1	6	6	19.3	49.222	NaN	45982000	51707000	50634000	62254000	54051000	1.139255	0.262011
Q14669	E3 ubiquitin-protein ligase TRIP12	TRIP12	1	4	4	2.7	220.43	10636000	12576000	14925000	10530000	10484000	NaN	0.82652	0.262126
Q9H6S3	Epidermal growth factor receptor kinase su	EPS8L2	1	6	6	13.4	80.62	42618000	38457000	52331000	53536000	60245000	44044000	1.183043	0.262321
Q8TDB6	E3 ubiquitin-protein ligase DTX3L	DTX3L	1	11	11	20.7	83.553	29001000	27476000	41439000	22518000	26156000	30020000	0.803689	0.263072
P02792	Ferritin light chain	FTL	1	6	6	49.7	20.019	61690000	75308000	68172000	77713000	68430000	1.07E+08	1.235722	0.263097
Q9P035	Very-long-chain (3R)-3-hydroxyacyl-CoA d	HACD3	1	7	7	26.8	43.159	50887000	46007000	54283000	52186000	53025000	56955000	1.07269	0.263201
P46937	Transcriptional coactivator YAP1	YAP1	1	6	6	20.6	54.461	21918000	19486000	15653000	23698000	24218000	18873000	1.170566	0.263257
Q9NWB1;O43251;A6NFN3	RNA binding protein fox-1 homolog 1;RNA	RBFOX1;RBFOX2;R	3	4	4	11.3	42.784	24481000	35539000	27848000	18469000	25888000	27009000	0.812196	0.263282
Q9NRX4	14 kDa phosphohistidine phosphatase	PHPT1	1	6	6	63.2	13.832	1.23E+08	65389000	96402000	1.11E+08	2.13E+08	1.1E+08	1.520855	0.264621
P31930	Cytochrome b-c1 complex subunit 1, mitoc	UQCRC1	1	14	14	36.7	52.645	92322000	1.13E+08	1.07E+08	1.13E+08	1.07E+08	1.24E+08	1.099683	0.264816
P09110	3-ketoacyl-CoA thiolase, peroxisomal	ACAA1	1	7	7	31.4	44.292	31135000	28894000	32304000	30046000	29859000	26282000	0.933437	0.264859
P30626	Sorcin	SRI	1	6	6	38.4	21.676	60586000	86566000	58865000	86974000	78450000	77332000	1.17833	0.265317
Q06323	Proteasome activator complex subunit 1	PSME1	1	14	14	55	28.723	2.09E+08	2.23E+08	2.1E+08	1.88E+08	2.02E+08	2.16E+08	0.94322	0.265952
O95782	AP-2 complex subunit alpha-1	AP2A1	1	27	21	33.6	107.54	1.53E+08	1.56E+08	1.53E+08	1.66E+08	1.63E+08	1.51E+08	1.038167	0.266967
Q14558	Phosphoribosyl pyrophosphate synthase-a	PRPSAP1	1	5	4	24.2	39.393	26908000	25766000	NaN	12145000	21804000	24835000	0.743998	0.267036
O00186	Syntaxin-binding protein 3	STXBP3	1	9	9	17.7	67.764	17041000	17526000	22221000	16995000	13250000	18044000	0.850338	0.267522
Q16836	Hydroxyacyl-coenzyme A dehydrogenase,	HADH	1	6	6	30.9	34.293	61431000	79661000	68629000	74669000	75335000	1.14E+08	1.261028	0.267626
Q8WW12	PEST proteolytic signal-containing nuclear	PCNP	1	3	3	24.7	18.925	59568000	58089000	49547000	47357000	49441000	55279000	0.90953	0.267858
O43776	Asparagine--tRNA ligase, cytoplasmic	NARS	1	16	16	39.6	62.942	1.14E+08	1.3E+08	1.27E+08	1.07E+08	1.21E+08	1.19E+08	0.932301	0.268153
Q9HCU5	Prolactin regulatory element-binding prote	PREB	1	4	4	14.4	45.468	9270900	14057000	17228000	9942600	9105500	11999000	0.765538	0.268272
O43896;Q12756;O60333	Kinesin-like protein KIF1C;Kinesin-like prot	KIF1C;KIF1A;KIF1B	3	3	3	3.3	122.95	26109000	28943000	22671000	30998000	37452000	24886000	1.20088	0.268737
P68104;Q5VTE0;Q05639	Elongation factor 1-alpha 1;Putative elonga	EEF1A1;EEF1A1P5	3	19	19	58.4	50.14	1.96E+09	2.16E+09	2.1E+09	2.2E+09	2.07E+09	2.25E+09	1.048757	0.269073
Q9Y5P6	Mannose-1-phosphate guanyltransferase b	GMPPB	1	5	5	19.4	39.834	32844000	37360000	26899000	26495000	24432000	31796000	0.85191	0.2692

P51397	Death-associated protein 1	DAP	1	1	1	24.5	11.164	19971000	21035000	NaN	14892000	20883000	15908000	0.840251	0.269237
P15531	Nucleoside diphosphate kinase A	NME1	1	10	5	57.9	17.149	2.34E+08	2.87E+08	3.31E+08	2.4E+08	2.35E+08	2.63E+08	0.866642	0.269898
P63279	SUMO-conjugating enzyme UBC9	UBE2I	1	7	7	39.2	18.007	51479000	54443000	57432000	49727000	55391000	44336000	0.914909	0.270309
P51116;Q06787	Fragile X mental retardation syndrome-rela	FXR2	2	11	9	21.8	74.222	21527000	23415000	20022000	18240000	18191000	22209000	0.902654	0.271492
Q15435	Protein phosphatase 1 regulatory subunit 7	PPP1R7	1	12	12	50.8	41.564	43152000	41968000	42434000	40361000	49733000	49035000	1.090746	0.272392
Q8TAQ2;Q92922	SWI/SNF complex subunit SMARCC2	SMARCC2	2	6	6	6	132.88	27595000	26007000	31869000	28301000	33147000	33070000	1.105849	0.27272
Q9Y3B3	Transmembrane emp24 domain-containing	TMED7	1	3	3	14.7	25.171	23525000	26578000	45402000	59020000	49337000	29386000	1.44226	0.272937
Q02218;Q9ULD0	2-oxoglutarate dehydrogenase, mitochond	OGDH	2	22	22	28.8	115.93	1.05E+08	1.02E+08	99325000	1.03E+08	1.05E+08	1.21E+08	1.072058	0.275121
Q13045	Protein flightless-1 homolog	FLII	1	17	17	16.9	144.75	50483000	51479000	35877000	55992000	50544000	51443000	1.146112	0.275345
P20020;Q01814	Plasma membrane calcium-transporting AT	ATP2B1	2	15	6	17.6	134.68	13718000	14484000	14587000	14798000	14221000	15702000	1.045152	0.275899
Q99497	Protein deglycase DJ-1	PARK7	1	12	12	64	19.891	3.54E+08	3.48E+08	4E+08	3.52E+08	3.29E+08	3.53E+08	0.938163	0.275951
Q15084	Protein disulfide-isomerase A6	PDIA6	1	16	16	43.6	48.121	4.96E+08	5.45E+08	4.95E+08	4.12E+08	4.9E+08	5.07E+08	0.917807	0.276522
Q9NZM1	Myoferlin	MYOF	1	56	56	35.9	234.71	86941000	89554000	89397000	77133000	80402000	91559000	0.936824	0.276565
P28289	Tropomodulin-1	TMOD1	1	5	5	23.7	40.569	9732700	15261000	8806500	6649600	NaN	8692500	0.680858	0.276579
C9JLW8	Protein FAM195B	FAM195B	1	2	2	29.9	10.92	26084000	22362000	NaN	24622000	34085000	29042000	1.207516	0.276877
P60903	Protein S100-A10	S100A10	1	3	3	35.1	11.203	3.96E+08	5.07E+08	4.62E+08	4.86E+08	4.77E+08	5.53E+08	1.111009	0.277321
O75348;O95670	V-type proton ATPase subunit G 1	ATP6V1G1	2	4	4	46.6	13.757	1.04E+08	1.25E+08	1.07E+08	79387000	89304000	1.18E+08	0.85111	0.277548
Q9Y617	Phosphoserine aminotransferase	PSAT1	1	10	10	36.5	40.422	35187000	24222000	32402000	39208000	38154000	30538000	1.17524	0.278101
P09496	Clathrin light chain A	CLTA	1	3	3	10.5	27.076	1.94E+08	1.88E+08	1.93E+08	1.8E+08	1.94E+08	1.4E+08	0.893681	0.278734
Q96N67;Q8NF50	Dedicator of cytokinesis protein 7	DOCK7	2	7	7	5	242.56	15674000	16878000	18416000	15682000	16812000	14739000	0.926719	0.278757
O15228	Dihydroxyacetone phosphate acyltransfera	GNPAT	1	4	4	9.1	77.187	16065000	11965000	14779000	9976300	12981000	13649000	0.855108	0.279855
Q08380	Galectin-3-binding protein	LGALS3BP	1	7	7	17.3	65.33	50481000	58856000	51686000	57250000	57300000	56336000	1.061252	0.279855
P84095	Rho-related GTP-binding protein RhoG	RHOG	1	5	4	41.4	21.308	28879000	30035000	37078000	25811000	32386000	24627000	0.862822	0.280411
O95571	Persulfide dioxygenase ETHE1, mitochondr	ETHE1	1	6	6	37	27.873	80764000	79412000	1.02E+08	1.04E+08	85900000	1.1E+08	1.146219	0.280975
P04406;O14556	Glyceraldehyde-3-phosphate dehydrogena	GAPDH	2	19	19	78.8	36.053	3.52E+09	3.78E+09	3.35E+09	3.31E+09	3.12E+09	3.55E+09	0.937749	0.281318
P62424	60S ribosomal protein L7a	RPL7A	1	11	11	39.5	29.995	2.46E+08	2.58E+08	2.31E+08	3.63E+08	3.08E+08	2.21E+08	1.21386	0.281758
P82650	28S ribosomal protein S22, mitochondrial	MRPS22	1	6	6	20.8	41.28	31287000	26210000	32746000	27593000	40533000	73052000	1.564421	0.281982
Q9UI12	V-type proton ATPase subunit H	ATP6V1H	1	10	10	33.3	55.882	51853000	60212000	59612000	62659000	56534000	74086000	1.125829	0.282618
P17096	High mobility group protein HMG-I/HMG-	HMGA1	1	5	5	23.4	11.676	6.63E+08	7.27E+08	8.4E+08	6.59E+08	6.55E+08	7.11E+08	0.908197	0.28272
Q9GZZ9	Ubiquitin-like modifier-activating enzyme 5	UBA5	1	4	4	19.3	44.863	32273000	35903000	27001000	29017000	26703000	29372000	0.89404	0.283567
O95777	U6 snRNA-associated Sm-like protein LSm	LSM8	1	3	3	37.5	10.403	20320000	25598000	21567000	20194000	NaN	19380000	0.879618	0.283597
Q07955	Serine/arginine-rich splicing factor 1	SRSF1	1	10	10	43.5	27.744	1.57E+08	1.37E+08	1.56E+08	1.38E+08	1.51E+08	1.16E+08	0.900332	0.283632
O95336	6-phosphogluconolactonase	PGLS	1	7	7	38.4	27.547	1.04E+08	99363000	1.41E+08	92612000	1.01E+08	1.02E+08	0.854396	0.284161
P32119	Peroxiredoxin-2	PRDX2	1	7	6	45.5	21.892	3.04E+08	3.33E+08	3.2E+08	2.75E+08	2.63E+08	3.33E+08	0.91042	0.284409
Q9NR50	Translation initiation factor eIF-2B subunit g	EIF2B3	1	4	4	14.8	50.24	21371000	27751000	27514000	19292000	22422000	25044000	0.871105	0.284908
Q13098	COP9 signalosome complex subunit 1	GPS1	1	10	10	22.6	55.536	33024000	31147000	32053000	32812000	36395000	32233000	1.054207	0.285031
O75083	WD repeat-containing protein 1	WDR1	1	26	26	61.7	66.193	2.33E+08	2.36E+08	2.61E+08	2.21E+08	2.44E+08	2.23E+08	0.941665	0.285171
Q8NBQ5	Estradiol 17-beta-dehydrogenase 11	HSD17B11	1	4	4	20	32.963	29373000	20550000	37443000	28356000	22225000	9328200	0.685727	0.285618
O60488	Long-chain-fatty-acid--CoA ligase 4	ACSL4	1	9	8	21.4	79.187	17939000	17232000	22978000	14205000	19630000	15248000	0.84409	0.28604
Q9BV20	Methylthioribose-1-phosphate isomerase	MRI1	1	2	2	6.2	39.149	1.36E+08	1.39E+08	1.94E+08	NaN	1.29E+08	1.21E+08	0.799079	0.287283
Q96AE4	Far upstream element-binding protein 1	FUBP1	1	13	12	25.6	67.56	1.7E+08	1.54E+08	1.93E+08	1.71E+08	1.43E+08	1.5E+08	0.900002	0.287551
O95810	Serum deprivation-response protein	SDPR	1	6	6	17.6	47.173	40223000	33751000	42826000	37405000	46234000	50259000	1.146387	0.288186
P61758	Prefoldin subunit 3	VBP1	1	6	6	35.5	22.626	72284000	79425000	1.02E+08	54699000	69276000	84337000	0.819903	0.288435
Q6P2E9	Enhancer of mRNA-decapping protein 4	EDC4	1	11	11	13.5	151.66	56965000	66099000	67615000	44803000	52853000	66653000	0.861705	0.2889
P06756	Integrin alpha-V;Integrin alpha-V heavy ch	ITGAV	1	11	11	14.1	116.04	22941000	27034000	24811000	23835000	23491000	23060000	0.941165	0.290123
O75494;Q8WXF0	Serine/arginine-rich splicing factor 10;Serin	SRSF10;SRSF12	2	4	4	13	31.3	20185000	20204000	24522000	14016000	17540000	22720000	0.83616	0.290136
Q14203	Dynactin subunit 1	DCTN1	1	31	31	35.2	141.69	52933000	47209000	50351000	50610000	53059000	53837000	1.0466	0.290201
Q9Y606	tRNA pseudouridine synthase A, mitochond	PUS1	1	2	2	5.2	47.47	NaN	7819100	7195000	8838700	NaN	7844700	1.111182	0.290887
Q9Y230	RuvB-like 2	RUVBL2	1	16	16	44.5	51.156	66903000	67910000	62853000	56047000	62663000	66489000	0.936929	0.291043
P61313	60S ribosomal protein L15	RPL15	1	10	10	43.1	24.146	1.4E+08	1.71E+08	2.19E+08	3.52E+08	2.23E+08	1.69E+08	1.405251	0.291219
P09104	Gamma-enolase	ENO2	1	7	5	27.9	47.268	23371000	15620000	23741000	37216000	28284000	18840000	1.344449	0.291259
Q8N8S7;Q9UI08	Protein enabled homolog	ENAH	2	10	10	23.9	66.509	52708000	47405000	42048000	53992000	47145000	55623000	1.102693	0.293415
P62191	26S protease regulatory subunit 4	PSMC1	1	16	16	43.6	49.184	62651000	70696000	75982000	66625000	62975000	65170000	0.930449	0.293674
O00151	PDZ and LIM domain protein 1	PDLIM1	1	21	21	84.8	36.071	4.08E+08	2.75E+08	3.61E+08	3.25E+08	3E+08	2.63E+08	0.850695	0.293988
Q12792	Twinfilin-1	TWF1	1	12	12	40.3	40.282	56538000	53112000	58929000	55009000	53763000	53466000	0.962386	0.294358
P53621	Coatomer subunit alpha;Xenin;Proxenin	COPA	1	39	39	40.6	138.34	1.29E+08	1.4E+08	1.27E+08	1.3E+08	1.25E+08	1.27E+08	0.962417	0.29445
Q9H2M9	Rab3 GTPase-activating protein non-cataly	RAB3GAP2	1	17	17	17.6	155.98	32798000	33785000	28135000	27794000	29688000	30366000	0.927469	0.295558
Q93009	Ubiquitin carboxyl-terminal hydrolase 7	USP7	1	7	7	9.6	128.3	65528000	1.13E+08	NaN	63455000	72624000	62856000	0.743579	0.295586
Q99470	Stromal cell-derived factor 2	SDF2	1	2	2	18	23.026	21402000	14520000	15476000	13966000	13247000	NaN	0.794185	0.295794

Q9P2K8	Eukaryotic translation initiation factor 2-alp	EIF2AK4	1	10	10	8.1	186.91	13977000	14007000	12564000	14780000	13254000	19502000	1.172339	0.296108
O43175	D-3-phosphoglycerate dehydrogenase	PHGDH	1	13	13	32.8	56.65	66393000	56533000	74504000	69698000	76334000	71404000	1.101332	0.296849
Q8N335	Glycerol-3-phosphate dehydrogenase 1-lil	GPD1L	1	2	2	6.8	38.418	17326000	NaN	15353000	18422000	NaN	17373000	1.095352	0.29788
Q9Y6M1	Insulin-like growth factor 2 mRNA-binding	IGF2BP2	1	8	7	19.5	66.121	40349000	41263000	39083000	47938000	37189000	49883000	1.118605	0.298571
P50990	T-complex protein 1 subunit theta	CCT8	1	27	27	55.5	59.62	1.97E+08	1.99E+08	2.14E+08	1.78E+08	2.01E+08	1.98E+08	0.946722	0.299018
O75369	Filamin-B	FLNB	1	87	75	48	278.16	1.13E+08	1E+08	1.1E+08	1.1E+08	1.11E+08	1.19E+08	1.052936	0.299802
Q7Z6B7	SLIT-ROBO Rho GTPase-activating protein	SRGAP1	1	4	3	4.6	124.26	16628000	NaN	18098000	16291000	15285000	17170000	0.935821	0.299845
Q16537	Serine/threonine-protein phosphatase 2A !	PPP2R5E	1	4	3	12.8	54.699	14539000	15799000	NaN	11364000	15391000	12790000	0.868987	0.300641
O00429	Dynamin-1-like protein	DNM1L	1	11	11	21.1	81.876	27968000	31628000	25178000	32641000	30457000	29219000	1.088978	0.300984
Q01085	Nucleolysin TIAR	TIAL1	1	4	4	19.2	41.59	11435000	14881000	13137000	15274000	13942000	14080000	1.097407	0.301586
P51532	Transcription activator BRG1	SMARCA4	1	3	3	2.6	184.64	8717500	9966200	9254400	8204500	6457100	9704700	0.872153	0.302008
Q32MZ4	Leucine-rich repeat flightless-interacting pı	LRRFIP1	1	14	13	24	89.252	42438000	45046000	41268000	43741000	38676000	39500000	0.946913	0.302318
P49593	Protein phosphatase 1F	PPM1F	1	8	8	23.8	49.83	29299000	28195000	37697000	33913000	36054000	36138000	1.114654	0.303972
P51991	Heterogeneous nuclear ribonucleoprotein ,	HNRNPA3	1	14	14	34.9	39.594	3.54E+08	3.89E+08	3.46E+08	3.28E+08	3.53E+08	3.53E+08	0.949885	0.305389
Q8TDX7;Q9HC98	Serine/threonine-protein kinase Nek7	NEK7	2	6	6	20.9	34.551	21762000	21920000	23713000	17194000	22787000	21171000	0.907367	0.306262
P17174	Aspartate aminotransferase, cytoplasmic	GOT1	1	6	6	20.6	46.247	45258000	49593000	43804000	39112000	43817000	46200000	0.931297	0.306788
P78356	Phosphatidylinositol 5-phosphate 4-kinase	PIP4K2B	1	7	5	21.6	47.377	19117000	17920000	21497000	17603000	18855000	18179000	0.933423	0.307527
Q16531	DNA damage-binding protein 1	DDB1	1	22	22	24.4	126.97	46438000	52328000	45790000	51266000	50960000	49787000	1.051586	0.307657
Q9HC38	Glyoxalase domain-containing protein 4	GLOD4	1	7	7	25.9	34.793	1.38E+08	1.37E+08	1.29E+08	1.31E+08	1.69E+08	1.44E+08	1.100149	0.307689
P05386	60S acidic ribosomal protein P1	RPLP1	1	3	3	65.8	11.514	1.28E+08	1.39E+08	1.27E+08	1.15E+08	1.17E+08	1.35E+08	0.933644	0.308744
P62847	40S ribosomal protein S24	RPS24	1	3	3	26.3	15.423	67049000	83797000	NaN	1.48E+08	87563000	NaN	1.560021	0.308973
Q5VT25	Serine/threonine-protein kinase MRCK alpł	CDC42BPA	1	5	5	3.9	197.3	40505000	41800000	35076000	41704000	40728000	42321000	1.062804	0.309151
P49748	Very long-chain specific acyl-CoA dehydro	ACADVL	1	21	21	42	70.389	1.89E+08	2.08E+08	2.04E+08	2.09E+08	2E+08	2.25E+08	1.054645	0.309403
Q5H9L2;Q969E4;Q6IPX3	Transcription elongation factor A protein-li	TCEAL5;TCEAL3;TC	3	2	2	8.7	23.307	47672000	55186000	49372000	47932000	47463000	48802000	0.947231	0.310594
O95757	Heat shock 70 kDa protein 4L	HSPA4L	1	20	17	35.2	94.511	66755000	57018000	51345000	60305000	69872000	63399000	1.105403	0.311015
Q9Y6Y8	SEC23-interacting protein	SEC23IP	1	10	10	15.5	111.08	23123000	23357000	28172000	23489000	22313000	23012000	0.921797	0.311289
Q9H9B4	Sideroflexin-1	SFXN1	1	8	8	36.6	35.619	56072000	52104000	55938000	52198000	51196000	54839000	0.964165	0.311823
P62942	Peptidyl-prolyl cis-trans isomerase FKBP1A	FKBP1A	1	5	5	29.6	11.951	9.32E+08	6.81E+08	8E+08	7.11E+08	7.27E+08	7.23E+08	0.895601	0.311859
P61769	Beta-2-microglobulin;Beta-2-microglobuli	B2M	1	4	4	37.8	13.714	2.2E+08	2.55E+08	1.78E+08	1.73E+08	2.29E+08	94240000	0.760627	0.311978
Q8TF72	Protein Shroom3	SHROOM3	1	10	10	8.2	216.85	1.04E+09	6.66E+08	8.14E+08	NaN	6.49E+08	6.89E+08	0.796152	0.31317
O94826	Mitochondrial import receptor subunit TON	TOMM70A	1	10	10	31.7	67.454	55596000	47471000	48847000	41796000	52456000	43219000	0.904926	0.313536
P49368	T-complex protein 1 subunit gamma	CCT3	1	25	25	52.1	60.533	3.03E+08	3.28E+08	2.98E+08	2.74E+08	2.85E+08	3.16E+08	0.941932	0.313586
Q15434	RNA-binding motif, single-stranded-intera	RBMS2	1	5	4	15.7	43.958	53856000	34793000	31720000	33231000	36948000	21206000	0.759207	0.313996
P55084	Trifunctional enzyme subunit beta, mitochc	HADHB	1	16	16	39.2	51.294	1.29E+08	1.36E+08	1.31E+08	1.31E+08	1.37E+08	1.49E+08	1.049703	0.31431
P47756	F-actin-capping protein subunit beta	CAPZB	1	17	17	56.6	30.628	2.34E+08	1.83E+08	2.33E+08	1.93E+08	2.05E+08	1.93E+08	0.907752	0.314354
Q15233	Non-POU domain-containing octamer-bir	NONO	1	17	16	45.6	54.231	2.07E+08	2.71E+08	2.27E+08	1.93E+08	1.89E+08	2.38E+08	0.880026	0.314872
Q9UII2	ATPase inhibitor, mitochondrial	ATPIF1	1	3	3	11.3	12.249	3.07E+08	1.28E+08	1.5E+08	94689000	1.18E+08	NaN	0.54613	0.314876
O15372	Eukaryotic translation initiation factor 3 sub	EIF3H	1	10	10	32.4	39.93	57476000	67849000	68524000	53899000	57527000	65441000	0.912396	0.315669
Q9H3P7	Golgi resident protein GCP60	ACBD3	1	11	11	27.5	60.593	78749000	85905000	78240000	87961000	79570000	88385000	1.053612	0.31592
Q15121	Astrocytic phosphoprotein PEA-15	PEA15	1	7	7	60	15.04	3.71E+08	3.55E+08	3.35E+08	2.69E+08	2.91E+08	3.8E+08	0.885282	0.316463
O43395	U4/U6 small nuclear ribonucleoprotein Prp	PRPF3	1	3	3	4.4	77.528	11277000	9840500	13041000	9257500	11183000	10006000	0.89133	0.31658
Q9BRR6	ADP-dependent glucokinase	ADPGK	1	5	5	14.9	54.088	36056000	34032000	42568000	35388000	35908000	31158000	0.909441	0.317806
Q92973	Transportin-1	TNPO1	1	13	12	20.2	102.35	91852000	84833000	81279000	73721000	82425000	85656000	0.937348	0.318094
Q9NPQ8;Q9NVN3	Synembryn-A	RIC8A	2	9	9	26.6	59.709	25592000	26048000	26967000	25335000	26404000	20344000	0.917005	0.318619
Q9H1B7	Interferon regulatory factor 2-binding prot	IRF2BPL	1	3	2	5.3	82.658	8566800	NaN	12819000	8407100	7143500	NaN	0.727146	0.31893
O43837	Isocitrate dehydrogenase [NAD] subunit be	IDH3B	1	8	8	34.5	42.183	42879000	46085000	43108000	45120000	44445000	46702000	1.031763	0.319188
Q13057	Bifunctional coenzyme A synthase;Phosphc	COASY	1	4	4	9.4	62.328	37755000	32430000	35772000	26329000	35149000	33761000	0.898846	0.31985
O43752	Syntaxin-6	STX6	1	2	2	9.4	29.176	15389000	13488000	13554000	11831000	14896000	11401000	0.898588	0.32002
Q96BJ3	Axin interactor, dorsalization-associated pr	AIDA	1	6	6	27.8	35.023	31204000	29067000	22707000	24306000	26260000	23215000	0.889163	0.320058
P19525	Interferon-induced, double-stranded RNA-	EIF2AK2	1	13	13	31.4	62.094	1.97E+08	1.87E+08	1.8E+08	1.87E+08	1.95E+08	2.29E+08	1.082413	0.320523
Q9Y265	RuvB-like 1	RUVBL1	1	17	17	50.2	50.227	84279000	87408000	85820000	78466000	79002000	88533000	0.955318	0.321442
Q8IY17	Neuropathy target esterase	PNPLA6	1	4	4	5.7	150.95	19571000	NaN	20307000	15039000	18062000	20013000	0.887942	0.321876
Q658P3	Metalloreductase STEAP3	STEAP3	1	4	4	11.1	54.6	20046000	18270000	NaN	13102000	18331000	17974000	0.859641	0.322517
Q16891	MICOS complex subunit MIC60	IMMT	1	18	18	30.6	83.677	83585000	93327000	80857000	82712000	94974000	1.07E+08	1.103841	0.323468
O75663	TIP41-like protein	TIPRL	1	7	7	36.4	31.444	59436000	60520000	28845000	57357000	64382000	62806000	1.240213	0.323825
Q8WZ82	Ovarian cancer-associated gene 2 protein	OVCA2	1	3	3	22	24.418	29754000	32243000	32045000	27264000	25736000	33059000	0.915112	0.324199
O75915	PRA1 family protein 3	ARL6IP5	1	6	6	30.9	21.614	1.56E+08	1.6E+08	1.58E+08	1.41E+08	1.57E+08	1.56E+08	0.961056	0.327205
P11234	Ras-related protein Ral-B	RALB	1	4	3	20.9	23.408	21292000	14111000	NaN	28150000	20616000	NaN	1.377454	0.327826
P41214	Eukaryotic translation initiation factor 2D	EIF2D	1	5	5	14.7	64.706	NaN	11592000	12662000	11972000	13487000	14212000	1.090432	0.327927

Q9HAU4;Q9HCE7	E3 ubiquitin-protein ligase SMURF2	SMURF2	2	6	6	10.6	86.195	22528000	25103000	26189000	24824000	22559000	21625000	0.934814	0.32845
P52630	Signal transducer and activator of transcrip	STAT2	1	10	10	14.7	97.915	14348000	15129000	15535000	15055000	15299000	17491000	1.062939	0.328483
Q6L8Q7	2,5-phosphodiesterase 12	PDE12	1	5	5	17.2	67.351	32958000	31771000	27415000	30576000	33403000	35563000	1.080287	0.32874
P30043	Flavin reductase (NADPH)	BLVRB	1	8	8	51.9	22.119	65023000	83230000	94580000	91178000	87455000	93445000	1.120433	0.329307
P07108	Acyl-CoA-binding protein	DBI	1	4	4	59.8	10.044	93425000	88560000	88188000	1.21E+08	94080000	88780000	1.126167	0.329645
Q92520	Protein FAM3C	FAM3C	1	5	5	27.8	24.68	47572000	59098000	44554000	39555000	47980000	46595000	0.886962	0.329958
P15144	Aminopeptidase N	ANPEP	1	17	17	21.9	109.54	76385000	86465000	82646000	81485000	86672000	90250000	1.052591	0.33028
O76024	Wolframin	WFS1	1	9	9	13.8	100.29	34155000	36273000	29466000	26896000	31609000	32561000	0.911626	0.331762
P05455	Lupus La protein	SSB	1	19	19	39.5	46.836	1.27E+08	1.32E+08	1.3E+08	1.12E+08	1.11E+08	1.37E+08	0.925493	0.33236
Q9GZN8	UPF0687 protein C20orf27	C20orf27	1	2	2	19	19.291	17597000	19965000	17904000	18926000	19048000	31554000	1.253525	0.332423
P41236;Q6NXS1	Protein phosphatase inhibitor 2	PPP1R2	2	3	3	22.4	23.015	34294000	28498000	NaN	30926000	26614000	26953000	0.897068	0.332537
P04216	Thy-1 membrane glycoprotein	THY1	1	2	2	18	17.935	1.22E+08	1.17E+08	1.11E+08	2.03E+08	1.34E+08	1.08E+08	1.269641	0.332605
O75312	Zinc finger protein ZPR1	ZPR1	1	4	4	11.5	50.925	10652000	12201000	12915000	10822000	11737000	10772000	0.931866	0.332754
P62979;P0CG47;P0CG48	Ubiquitin-40S ribosomal protein S27a;Ubiq	RPS27A;UBB;UBC	3	8	2	44.2	17.965	1.8E+09	1.77E+09	1.8E+09	1.47E+09	1.81E+09	1.74E+09	0.935562	0.332957
Q92890	Ubiquitin fusion degradation protein 1 hom	UFD1L	1	5	5	28.7	34.5	29478000	25762000	17417000	32764000	29072000	24812000	1.192562	0.333332
P30519	Heme oxygenase 2	HMOX2	1	5	5	25	36.032	22162000	21527000	24079000	22110000	20116000	22172000	0.950272	0.33338
Q9Y3A3	MOB-like protein phocein	MOB4	1	4	4	31.1	26.032	18833000	15286000	16686000	15662000	15814000	15920000	0.9329	0.33356
P11586	C-1-tetrahydrofolate synthase, cytoplasmic	MTHFD1	1	18	18	27.8	101.53	29540000	29108000	36965000	32576000	36847000	35543000	1.097821	0.334848
Q9Y3B8	Oligoribonuclease, mitochondrial	REXO2	1	6	6	30	26.832	43487000	56136000	69762000	65072000	63768000	65532000	1.147516	0.334951
P51970	NADH dehydrogenase [ubiquinone] 1 alph	NDUFA8	1	3	3	30.2	20.105	46243000	33315000	57045000	35256000	44166000	31494000	0.811959	0.335113
Q9NZL9	Methionine adenosyltransferase 2 subunit b	MAT2B	1	9	9	34.4	37.551	49549000	48316000	38565000	53279000	51549000	45428000	1.101341	0.335354
P30740	Leukocyte elastase inhibitor	SERPINB1	1	9	9	26.9	42.741	1.81E+08	2.08E+08	2.15E+08	2.28E+08	2.14E+08	2.03E+08	1.06849	0.336016
P08397	Porphobilinogen deaminase	HMBS	1	2	2	7.8	39.33	NaN	11920000	15695000	15946000	16309000	14539000	1.129676	0.336897
Q92804	TATA-binding protein-associated factor 2N	TAF15	1	5	3	10.8	61.829	17732000	23732000	22817000	NaN	16877000	19908000	0.85838	0.336935
Q9NWM8	Peptidyl-prolyl cis-trans isomerase FKBP14	FKBP14	1	4	4	27	24.171	25602000	24264000	38525000	19267000	21349000	29665000	0.795115	0.337628
Q15257	Serine/threonine-protein phosphatase 2A a	PPP2R4	1	5	5	15.9	40.667	32185000	30109000	29068000	30419000	29196000	28003000	0.95902	0.339394
Q96G23	Ceramide synthase 2	CERS2	1	4	4	14.2	44.876	77917000	63833000	1.17E+08	72580000	70790000	62780000	0.795209	0.339759
O95297	Myelin protein zero-like protein 1	MPZL1	1	5	5	27.9	29.082	22312000	21091000	25723000	20599000	22608000	21011000	0.928999	0.341086
Q8WUM0	Nuclear pore complex protein Nup133	NUP133	1	4	4	5.7	128.98	19805000	15976000	15019000	14106000	15326000	16232000	0.898898	0.341297
P00558;P07205	Phosphoglycerate kinase 1	PGK1	2	23	23	59	44.614	8.58E+08	8.86E+08	7.55E+08	9.09E+08	8.97E+08	8.4E+08	1.0583	0.342862
P31949	Protein S100-A11;Protein S100-A11, N-ter	S100A11	1	7	7	81.9	11.74	1.85E+09	2.1E+09	1.86E+09	1.85E+09	2.15E+09	2.33E+09	1.090568	0.343122
Q9BTV4	Transmembrane protein 43	TMEM43	1	10	10	38.5	44.875	36033000	32719000	39022000	41612000	36086000	37933000	1.072903	0.343474
Q14914	Prostaglandin reductase 1	PTGR1	1	13	13	45.9	35.869	2.19E+08	2.02E+08	2.33E+08	1.93E+08	1.94E+08	2.23E+08	0.934553	0.344677
O95232	Luc7-like protein 3	LUC7L3	1	2	2	5.8	51.466	12045000	10094000	11981000	9437900	10830000	11184000	0.921802	0.34571
P18077	60S ribosomal protein L35a	RPL35A	1	3	3	15.5	12.538	3.81E+08	3.37E+08	3.83E+08	3.56E+08	3.36E+08	3.56E+08	0.951982	0.34677
Q99643	Succinate dehydrogenase cytochrome b560	SDHC	1	2	2	12.4	18.61	15344000	16882000	NaN	46861000	25661000	15573000	1.822442	0.347214
Q9ULH0	Kinase D-interacting substrate of 220 kDa	KIDINS220	1	8	8	7.3	196.54	13809000	16802000	13699000	11716000	15590000	11803000	0.882622	0.348044
Q6PI48	Aspartate--tRNA ligase, mitochondrial	DARS2	1	5	5	10.2	73.562	23359000	21211000	22618000	24756000	24309000	21729000	1.05367	0.349055
P61247	40S ribosomal protein S3a	RPS3A	1	12	12	44.3	29.945	3.08E+08	3.53E+08	3.23E+08	2.83E+08	2.92E+08	3.39E+08	0.929452	0.349141
Q01518	Adenylyl cyclase-associated protein 1	CAP1	1	25	24	53.9	51.901	6.51E+08	6.88E+08	6.75E+08	6.85E+08	6.37E+08	6.22E+08	0.965537	0.349358
P30085	UMP-CMP kinase	CMPK1	1	8	8	47.4	22.222	78984000	90258000	77005000	67384000	75165000	83789000	0.91915	0.350471
Q9UNM6	26S proteasome non-ATPase regulatory su	PSMD13	1	11	11	37	42.945	60504000	66450000	52395000	52486000	55815000	57380000	0.923791	0.351219
Q13685	Angio-associated migratory cell protein	AAMP	1	2	2	6.2	46.75	14054000	14449000	NaN	13508000	14119000	NaN	0.969266	0.351745
Q96A49	Synapse-associated protein 1	SYAP1	1	7	7	25.3	39.933	42386000	46103000	53206000	36302000	48744000	41552000	0.893454	0.353987
Q7Z5L9	Interferon regulatory factor 2-binding prot	IRF2BP2	1	3	2	8.9	61.024	19039000	16794000	18096000	17479000	19180000	21259000	1.073968	0.354911
P07954	Fumarate hydratase, mitochondrial	FH	1	13	13	40	54.636	2.64E+08	3.07E+08	2.67E+08	2.78E+08	2.98E+08	3.2E+08	1.068682	0.355821
P31689	DnaJ homolog subfamily A member 1	DNAJA1	1	9	9	36	44.868	58575000	53819000	56942000	48215000	49342000	59624000	0.92822	0.356283
P14550	Alcohol dehydrogenase [NADP(+)]	AKR1A1	1	12	12	54.2	36.573	85527000	88616000	96033000	90619000	83551000	83900000	0.955192	0.356401
Q16401	26S proteasome non-ATPase regulatory su	PSMD5	1	5	5	10.3	56.195	52143000	49840000	44511000	42543000	50932000	41141000	0.918918	0.356468
Q03519	Antigen peptide transporter 2	TAP2	1	3	3	4.5	75.663	24103000	31259000	NaN	27415000	20889000	22846000	0.856785	0.357222
Q3MHD2	Protein LSM12 homolog	LSM12	1	4	4	27.2	21.701	40637000	37743000	32898000	35384000	36715000	28815000	0.906864	0.357562
P25325	3-mercaptopyruvate sulfurtransferase	MPST	1	2	2	12.1	33.178	10436000	12208000	NaN	10751000	8491100	NaN	0.849766	0.357871
P62906	60S ribosomal protein L10a	RPL10A	1	8	8	33.6	24.831	2.01E+08	1.78E+08	2.07E+08	1.8E+08	1.99E+08	1.65E+08	0.929279	0.358478
Q9H3N1	Thioredoxin-related transmembrane protei	TMX1	1	5	5	14.3	31.791	53147000	53227000	52787000	55083000	63030000	51579000	1.066166	0.358962
P27708	CAD protein;Glutamine-dependent carbam	CAD	1	19	19	13.4	242.98	22348000	27152000	25069000	24356000	29125000	27176000	1.081643	0.359498
Q14139	Ubiquitin conjugation factor E4 A	UBE4A	1	5	5	6.8	122.56	10011000	8725200	12162000	10926000	10649000	13560000	1.137121	0.359561
P52272	Heterogeneous nuclear ribonucleoprotein I	HNRNPM	1	18	18	31.5	77.515	2.33E+08	2.67E+08	2.51E+08	2.53E+08	2.15E+08	2.37E+08	0.938631	0.359696
Q9BR76	Coronin-1B	CORO1B	1	7	7	17.2	54.234	70026000	78396000	88307000	64841000	61286000	83650000	0.886148	0.361154
Q9UBQ7	Glyoxylate reductase/hydroxypyruvate redu	GRHPR	1	8	8	39.3	35.668	1.15E+08	1.39E+08	1.08E+08	1.21E+08	1.32E+08	1.43E+08	1.095532	0.361371

P43897	Elongation factor Ts, mitochondrial	TSFM	1	5	5	23.1	35.39	26058000	21385000	30501000	30760000	25554000	31650000	1.128554	0.361735
P14868	Aspartate--tRNA ligase, cytoplasmic	DARS	1	15	15	36.7	57.136	75861000	62333000	93154000	1.05E+08	85566000	77757000	1.161769	0.362412
Q16666;Q6K0P9	Gamma-interferon-inducible protein 16	IFI16	2	19	19	28.4	88.255	75657000	74565000	86515000	88058000	76544000	89262000	1.072346	0.363017
Q99426	Tubulin-folding cofactor B	TBCB	1	5	5	24.2	27.325	68399000	80590000	86806000	59489000	73766000	78393000	0.897593	0.363053
Q92747	Actin-related protein 2/3 complex subunit	ARPC1A	1	7	6	33.2	41.569	70810000	53591000	75117000	63939000	61669000	49413000	0.877219	0.363753
O75380	NADH dehydrogenase [ubiquinone] iron-s	NDUFS6	1	3	3	42.7	13.711	NaN	29319000	33699000	32333000	25251000	26539000	0.889936	0.36434
Q6IBS0	Twinfilin-2	TWF2	1	9	9	40.7	39.548	81190000	95372000	93830000	74005000	83455000	91916000	0.922276	0.364369
O00425	Insulin-like growth factor 2 mRNA-binding	IGF2BP3	1	10	8	23.7	63.704	39376000	48958000	41579000	46504000	44110000	49294000	1.076936	0.364802
P20338	Ras-related protein Rab-4A	RAB4A	1	3	3	19.3	24.389	13259000	16182000	15993000	15132000	15794000	19906000	1.11881	0.366083
Q8IZ07	Ankyrin repeat domain-containing protein	ANKRD13A	1	3	3	7.3	67.618	17297000	15872000	NaN	17027000	18604000	NaN	1.074226	0.36635
Q15070	Mitochondrial inner membrane protein OX	OXA1L	1	3	3	6.9	48.547	39422000	NaN	46587000	28876000	40917000	NaN	0.811462	0.366681
Q9Y2W1	Thyroid hormone receptor-associated prot	THRAP3	1	9	8	12.3	108.66	29117000	33511000	34841000	28779000	26961000	33676000	0.917379	0.368056
P30086	Phosphatidylethanolamine-binding protein	PEBP1	1	11	11	73.8	21.057	3.25E+08	3E+08	2.42E+08	3.32E+08	3.06E+08	3.09E+08	1.091501	0.368508
Q16576	Histone-binding protein RBBP7	RBBP7	1	5	2	16.9	47.82	42188000	54052000	38833000	44200000	38242000	37106000	0.885062	0.368805
P31153;Q00266	S-adenosylmethionine synthase isoform ty	MAT2A	2	8	8	29.9	43.66	93438000	63127000	91526000	67042000	81650000	66147000	0.865969	0.371172
Q15836	Vesicle-associated membrane protein 3	VAMP3	1	4	2	52	11.309	1.1E+08	1.27E+08	1.04E+08	96049000	1.12E+08	1.08E+08	0.92637	0.371577
O60841	Eukaryotic translation initiation factor 5B	EIF5B	1	19	19	22	138.83	53624000	51799000	51697000	52479000	47686000	51998000	0.968451	0.372623
O43681	ATPase ASNA1	ASNA1	1	7	7	28.4	38.792	60551000	61698000	50852000	63312000	64118000	57670000	1.069318	0.37325
P30050	60S ribosomal protein L12	RPL12	1	7	7	59.4	17.818	2.36E+08	2.09E+08	2.63E+08	2.29E+08	2.21E+08	2.07E+08	0.928855	0.373624
P84243	Histone H3.3	H3F3A	1	4	1	40.4	15.328	9.77E+08	9.52E+08	7.62E+08	1.16E+09	9.85E+08	8.71E+08	1.119197	0.373983
Q14161	ARF GTPase-activating protein GIT2	GIT2	1	2	2	2.9	84.542	9087400	10464000	NaN	9108300	9351700	9241000	0.944553	0.37465
Q9NVS9	Pyridoxine-5-phosphate oxidase	PNPO	1	5	5	27.2	29.988	17387000	12381000	20390000	21643000	27507000	14351000	1.266019	0.375577
Q15459	Splicing factor 3A subunit 1	SF3A1	1	9	9	14.2	88.885	26399000	24909000	28249000	23414000	22027000	28003000	0.923162	0.376061
Q10567	AP-1 complex subunit beta-1	AP1B1	1	26	12	34.1	104.61	47259000	55254000	50548000	47376000	48163000	50170000	0.951967	0.376337
Q96HC4	PDZ and LIM domain protein 5	PDLIM5	1	16	16	34.2	63.944	57634000	56623000	45015000	51601000	47426000	47514000	0.920068	0.377182
Q96BZ9	TBC1 domain family member 20	TBC1D20	1	2	2	7.9	45.854	NaN	13151000	12350000	NaN	16643000	13027000	1.163484	0.377238
P11717	Cation-independent mannose-6-phosphat	IGF2R	1	31	31	18.9	274.37	30770000	33771000	29749000	29387000	30963000	30120000	0.959487	0.379504
Q92688	Acidic leucine-rich nuclear phosphoprotein	ANP32B	1	4	2	19.1	28.787	59652000	64102000	58849000	60115000	56462000	NaN	0.957627	0.379815
P04156	Major prion protein	PRNP	1	2	2	9.5	27.661	42529000	28797000	46878000	33956000	36304000	31266000	0.858905	0.380071
Q86X76	Nitrilase homolog 1	NIT1	1	7	7	27.5	35.896	46725000	27123000	44907000	48639000	48984000	41113000	1.168254	0.38027
P09543	2,3-cyclic-nucleotide 3-phosphodiesterase	CNP	1	11	11	23	47.578	39856000	43791000	39334000	42756000	41386000	43331000	1.036526	0.38048
P42574	Caspase-3;Caspase-3 subunit p17;Caspase	CASP3	1	2	2	10.8	31.608	10347000	9448400	11186000	13124000	10465000	10420000	1.097723	0.380608
P30520	Adenylosuccinate synthetase isozyme 2	ADSS	1	12	12	37.5	50.097	53231000	43345000	50486000	48019000	43472000	46041000	0.935197	0.380656
Q969Q0	60S ribosomal protein L36a-like	RPL36AL	1	5	2	26.4	12.469	2.36E+08	2.72E+08	2.15E+08	2.18E+08	2.23E+08	2.32E+08	0.930178	0.381595
Q7Z406	Myosin-14	MYH14	1	10	2	4.6	227.87	6.56E+08	6.39E+08	NaN	1.1E+09	7.27E+08	6.59E+08	1.277876	0.381609
P45880	Voltage-dependent anion-selective channe	VDAC2	1	12	12	59.9	31.566	4.69E+08	4.21E+08	3.46E+08	4.98E+08	4.72E+08	4.01E+08	1.109872	0.381617
P52888	Thimet oligopeptidase	THOP1	1	4	4	7.7	78.839	21212000	29359000	NaN	29404000	30385000	26470000	1.137134	0.382228
Q9H4L5	Oxysterol-binding protein-related protein	OSBPL3	1	10	10	15.4	101.22	22869000	26751000	24712000	23455000	24168000	23338000	0.954649	0.384115
Q6P1J9	Parafibromin	CDC73	1	2	2	4.5	60.576	12820000	12544000	12886000	12498000	8093100	13054000	0.87961	0.384323
P07741	Adenine phosphoribosyltransferase	APRT	1	6	6	46.1	19.608	70090000	57259000	83139000	78061000	79512000	75106000	1.105426	0.384541
P40121	Macrophage-capping protein	CAPG	1	4	4	15.8	38.498	15717000	22231000	36241000	32671000	26005000	34851000	1.260659	0.384939
O00567	Nucleolar protein 56	NOP56	1	9	9	22.7	66.049	52834000	49139000	50365000	47734000	53135000	39649000	0.922409	0.38747
P37198	Nuclear pore glycoprotein p62	NUP62	1	3	3	10.5	53.254	17410000	20710000	21667000	17363000	18006000	20015000	0.926355	0.388025
P62879;Q9HAV0	Guanine nucleotide-binding protein G(I)/G	GNB2;GNB4	2	8	5	26.8	37.331	1.81E+08	2.1E+08	1.67E+08	1.84E+08	1.97E+08	2.33E+08	1.100023	0.389435
O00764	Pyridoxal kinase	PDXK	1	7	7	26.6	35.102	59957000	61176000	57148000	57283000	61723000	37526000	0.878007	0.390295
Q9Y6E0;Q9P289;O00506	Serine/threonine-protein kinase 24;Serine/	STK24	3	6	6	18.3	49.307	28809000	30024000	26366000	28928000	25518000	26487000	0.949929	0.390642
Q9UKK3	Poly [ADP-ribose] polymerase 4	PARP4	1	18	18	16.2	192.59	38346000	37953000	43691000	32106000	40564000	38306000	0.924877	0.3919
P61026	Ras-related protein Rab-10	RAB10	1	5	4	27.5	22.541	30015000	40427000	33589000	43215000	50598000	29805000	1.18828	0.391947
P00167	Cytochrome b5	CYB5A	1	4	4	42.5	15.33	86179000	54644000	87496000	95011000	92923000	75859000	1.15537	0.391958
Q9UJZ1	Stomatin-like protein 2, mitochondrial	STOML2	1	10	10	37.9	38.534	87847000	84402000	77278000	80357000	91352000	91762000	1.055882	0.393192
Q00765	Receptor expression-enhancing protein 5	REEP5	1	4	4	21.2	21.493	2.99E+08	3.31E+08	3.28E+08	3.66E+08	2.98E+08	3.66E+08	1.074554	0.393583
Q14192	Four and a half LIM domains protein 2	FHL2	1	19	19	76.3	32.193	2.71E+08	2.57E+08	2.74E+08	2.55E+08	2.7E+08	2.56E+08	0.97424	0.394047
O43615	Mitochondrial import inner membrane tran	TIMM44	1	4	4	10.4	51.355	20326000	18531000	18534000	16494000	15829000	20534000	0.920998	0.394861
P02794	Ferritin heavy chain;Ferritin heavy chain, N-	FTH1	1	6	6	50.3	21.225	37745000	52463000	51687000	42815000	43274000	42157000	0.903809	0.396244
P36776	Lon protease homolog, mitochondrial	LONP1	1	16	16	24.3	106.49	63934000	66560000	56735000	52979000	60264000	62383000	0.938028	0.397979
P22392;O60361	Nucleoside diphosphate kinase B;Putative r	NME2;NME2P1	2	10	5	62.5	17.298	6.35E+08	6.5E+08	5.59E+08	7.72E+08	7.96E+08	5.3E+08	1.137221	0.398227
O94925	Glutaminase kidney isoform, mitochondrial	GLS	1	11	11	23.9	73.46	73089000	74813000	68039000	72132000	72102000	86236000	1.067282	0.398303
Q8NC51	Plasminogen activator inhibitor 1 RNA-bind	SERBP1	1	18	18	47.5	44.965	2.48E+08	2.47E+08	2.82E+08	2.14E+08	2.61E+08	2.5E+08	0.933546	0.398905
P31749;P31751;Q9Y243	RAC-alpha serine/threonine-protein kinase	AKT1	3	9	9	25.4	55.686	21842000	19172000	14653000	15162000	15901000	18148000	0.884025	0.399078

O00217	NADH dehydrogenase [ubiquinone] iron-s	NDUFS8	1	2	2	14.3	23.705	11855000	13464000	15460000	NaN	11595000	12811000	0.897741	0.399924
Q9Y3F4	Serine-threonine kinase receptor-associate	STRAP	1	14	14	50.9	38.438	1.12E+08	92530000	1.15E+08	1.16E+08	1.13E+08	1.1E+08	1.063184	0.400295
P43121	Cell surface glycoprotein MUC18	MCAM	1	3	3	6.8	71.607	25849000	NaN	21011000	32376000	36825000	20380000	1.274449	0.40124
P12429	Annexin A3	ANXA3	1	3	3	14.6	36.375	13605000	14285000	NaN	14541000	17383000	13958000	1.096737	0.403126
Q9UJW0	Dynactin subunit 4	DCTN4	1	7	7	25.4	52.337	15440000	14531000	16801000	17252000	14394000	20067000	1.10564	0.403696
P14324	Farnesyl pyrophosphate synthase	FDPS	1	6	6	18.9	48.275	2.09E+08	2.16E+08	2.12E+08	2E+08	2.24E+08	1.66E+08	0.925741	0.404624
O60684;O15131	Importin subunit alpha-7;Importin subunit	KPNA6;KPNA5	2	8	6	22.6	60.029	22814000	31088000	23279000	29113000	29279000	26648000	1.101826	0.404803
P49591	Serine--tRNA ligase, cytoplasmic	SARS	1	12	12	31.7	58.777	68745000	84755000	82224000	73680000	70056000	77035000	0.936566	0.405209
Q9H1E3	Nuclear ubiquitous casein and cyclin-depe	NUCKS1	1	3	3	11.9	27.296	25825000	24145000	NaN	23285000	20907000	25495000	0.929718	0.406079
P51808	Dynein light chain Tctex-type 3	DYNLT3	1	3	3	64.7	13.062	1.86E+08	NaN	1.3E+08	1.22E+08	1.34E+08	NaN	0.812439	0.406836
P51665	26S proteasome non-ATPase regulatory su	PSMD7	1	6	6	27.5	37.025	73085000	62609000	88566000	64050000	67402000	71148000	0.903416	0.407744
P09211	Glutathione S-transferase P	GSTP1	1	13	13	66.2	23.356	2.57E+08	3.23E+08	2.76E+08	2.54E+08	2.32E+08	2.96E+08	0.912786	0.41157
Q96G03	Phosphoglucomutase-2	PGM2	1	15	15	31	68.283	52908000	48487000	44956000	54074000	48382000	51665000	1.053092	0.41197
Q15526	Surfeit locus protein 1	SURF1	1	3	3	12.3	33.331	NaN	16856000	12382000	14270000	18444000	17915000	1.154411	0.412319
P68366	Tubulin alpha-4A chain	TUBA4A	1	19	4	52.7	49.924	48888000	44576000	50822000	48105000	53037000	49598000	1.044731	0.412435
P06730	Eukaryotic translation initiation factor 4E	EIF4E	1	4	4	17.5	25.097	47516000	61477000	44646000	45436000	48670000	44941000	0.905024	0.41296
O00391	Sulfhydryl oxidase 1	QSOX1	1	7	7	11.2	82.577	29698000	41637000	34087000	27616000	31551000	35076000	0.89396	0.414681
Q9Y696	Chloride intracellular channel protein 4	CLIC4	1	16	16	74.7	28.772	3.81E+08	4.01E+08	3.4E+08	3.46E+08	3.41E+08	3.78E+08	0.94843	0.415841
P30049	ATP synthase subunit delta, mitochondrial	ATP5D	1	3	3	22.6	17.49	1.33E+08	1.34E+08	1.26E+08	1.42E+08	1.47E+08	1.24E+08	1.052285	0.417049
O14979	Heterogeneous nuclear ribonucleoprotein I	HNRNPDL	1	6	6	12.4	46.437	44782000	59823000	53272000	51441000	49411000	43624000	0.915117	0.417327
Q9BU23	Lipase maturation factor 2	LMF2	1	3	3	5	79.697	24060000	28391000	NaN	33521000	23774000	34873000	1.171481	0.41736
Q13363	C-terminal-binding protein 1	CTBP1	1	5	3	15.7	47.535	31068000	36831000	31779000	34921000	31918000	43256000	1.104507	0.417687
Q86YR5;P81274	G-protein-signaling modulator 1	GPSM1	2	6	6	10.4	74.509	15269000	17854000	14892000	15979000	16392000	19683000	1.08412	0.419208
Q8WVY7	Ubiquitin-like domain-containing CTD pho	UBLCP1	1	5	5	14.2	36.804	30002000	22523000	24364000	25172000	25344000	45934000	1.254406	0.419316
Q8IX12	Cell division cycle and apoptosis regulator	CCAR1	1	5	5	6.4	132.82	10550000	8212500	12467000	8814000	9036600	NaN	0.857391	0.419428
Q13509	Tubulin beta-3 chain	TUBB3	1	19	5	49.8	50.432	57788000	61938000	NaN	58547000	63769000	68803000	1.064202	0.420055
Q5VYK3	Proteasome-associated protein ECM29 hor	ECM29	1	9	9	7.3	204.29	25072000	30511000	23082000	21237000	25036000	25421000	0.911384	0.420476
Q86W92;Q8ND30	Liprin-beta-1	PPFIBP1	2	10	10	15.7	114.02	13785000	14036000	20080000	19573000	17818000	16527000	1.125613	0.421023
P50281	Matrix metalloproteinase-14	MMP14	1	3	3	6.4	65.893	15166000	17040000	NaN	16469000	18544000	NaN	1.087158	0.421136
Q08257	Quinone oxidoreductase	CRYZ	1	8	8	36.8	35.206	59552000	70701000	65484000	59730000	64335000	62321000	0.952227	0.421725
O60749	Sorting nexin-2	SNX2	1	13	11	30.4	58.47	74913000	66475000	70054000	87796000	96619000	58551000	1.149091	0.422118
O43504	Ragulator complex protein LAMTOR5	LAMTOR5	1	4	4	85.7	9.6138	53406000	48223000	39527000	45279000	49613000	67374000	1.149551	0.422307
Q16527	Cysteine and glycine-rich protein 2	CSRP2	1	8	8	45.6	20.954	1.04E+08	90735000	79687000	96723000	79539000	70406000	0.897425	0.422356
Q9Y608	Leucine-rich repeat flightless-interacting p	LRRFIP2	1	7	6	12.1	82.17	NaN	29344000	25420000	21716000	29101000	21488000	0.880201	0.422919
Q9NX63	MICOS complex subunit MIC19	CHCHD3	1	5	5	21.1	26.152	64047000	48374000	52529000	66000000	59155000	54988000	1.092107	0.423111
Q15393	Splicing factor 3B subunit 3	SF3B3	1	14	14	16.2	135.58	37417000	39366000	39719000	35052000	36079000	40506000	0.958241	0.423262
O60613	15 kDa selenoprotein	15-Sep	1	2	2	17.6	18.092	16935000	20896000	21027000	18564000	22291000	23222000	1.088671	0.424163
P61513	60S ribosomal protein L37a	RPL37A	1	4	4	42.4	10.275	50389000	82801000	1.41E+08	2.9E+08	62023000	NaN	1.923004	0.42439
Q69YQ0	Cytospin-A	SPECC1L	1	4	4	6.2	124.54	70644000	65463000	64309000	64696000	69237000	78468000	1.059801	0.424415
P23743	Diacylglycerol kinase alpha	DGKA	1	16	16	29.7	82.629	48268000	48096000	57276000	55699000	53220000	53108000	1.054589	0.424604
O75746	Calcium-binding mitochondrial carrier prot	SLC25A12	1	10	6	18.1	74.761	21147000	18092000	19450000	21246000	20754000	19442000	1.046908	0.425287
Q15029	116 kDa U5 small nuclear ribonucleoprotei	EFTUD2	1	15	14	21.6	109.43	26949000	27517000	28294000	22983000	24334000	29802000	0.931839	0.425371
P62136	Serine/threonine-protein phosphatase PP1	PPP1CA	1	13	4	45.8	37.512	1.87E+08	2.12E+08	2.29E+08	1.99E+08	1.86E+08	2.06E+08	0.942325	0.425707
P37108	Signal recognition particle 14 kDa protein	SRP14	1	3	3	19.9	14.57	2E+08	1.75E+08	1.44E+08	1.58E+08	1.69E+08	1.43E+08	0.90694	0.426276
Q8N1G4	Leucine-rich repeat-containing protein 47	LRRC47	1	13	13	26.6	63.472	34933000	33112000	36483000	38196000	35126000	34998000	1.036277	0.426338
O00139	Kinesin-like protein KIF2A	KIF2A	1	5	5	13.5	79.954	14913000	13767000	NaN	11635000	12300000	15116000	0.907741	0.426573
O43432	Eukaryotic translation initiation factor 4 gar	EIF4G3	1	10	7	8.1	176.65	16196000	20276000	20043000	21400000	17757000	22549000	1.091852	0.426907
O00499	Myc box-dependent-interacting protein 1	BIN1	1	2	2	5.4	64.699	10906000	12207000	NaN	12850000	11087000	14107000	1.097333	0.427991
P19338	Nucleolin	NCL	1	21	21	30.3	76.613	2.41E+08	2.64E+08	2.64E+08	2.72E+08	2.59E+08	2.62E+08	1.03033	0.428108
Q9ULA0	Aspartyl aminopeptidase	DNPEP	1	7	7	17.5	53.41	22062000	20820000	27435000	21484000	22943000	20109000	0.917787	0.42833
Q92783	Signal transducing adapter molecule 1	STAM	1	6	4	14.1	59.179	22402000	19326000	NaN	22293000	22462000	NaN	1.072541	0.42941
P59535	Taste receptor type 2 member 40	TAS2R40	1	2	2	9.6	36.811	33847000	29771000	NaN	22538000	31461000	NaN	0.848801	0.430212
Q9NY33	Dipeptidyl peptidase 3	DPP3	1	10	10	25.2	82.588	52898000	80559000	55584000	65419000	67251000	84419000	1.14837	0.430986
P26583	High mobility group protein B2	HMGB2	1	5	5	19.1	24.033	41229000	38682000	36523000	34557000	45136000	48077000	1.09736	0.431576
Q12996	Cleavage stimulation factor subunit 3	CSTF3	1	3	3	5.3	82.921	10526000	11968000	9719500	11943000	9922400	13667000	1.103028	0.431619
P35237	Serpin B6	SERPINB6	1	13	12	43.4	42.621	1.2E+08	1.16E+08	1.19E+08	1.13E+08	1.15E+08	1.21E+08	0.981041	0.43427
Q8N2K0	Monoacylglycerol lipase ABHD12	ABHD12	1	4	4	11.6	45.096	17459000	22104000	18742000	17258000	15131000	20492000	0.906972	0.43478
P09497	Clathrin light chain B	CLTB	1	7	7	19.7	25.19	1.5E+08	2.26E+08	1.73E+08	1.27E+08	1.64E+08	1.86E+08	0.866658	0.434825
P35232	Prohibitin	PHB	1	9	9	39.3	29.804	1.64E+08	1.52E+08	1.59E+08	1.73E+08	1.87E+08	1.47E+08	1.065696	0.43584

P52926	High mobility group protein HMGI-C	HMGA2	1	6	6	41.3	11.832	3.74E+08	4.73E+08	4.61E+08	3.61E+08	3.72E+08	4.61E+08	0.91204	0.435922
Q63HN8	E3 ubiquitin-protein ligase RNF213	RNF213	1	23	23	5.9	591.4	12943000	11437000	16502000	10653000	14404000	10808000	0.877281	0.436878
Q9BVA1	Tubulin beta-2B chain	TUBB2B	1	20	1	65.6	49.953	5.26E+08	5.46E+08	4.46E+08	4.55E+08	4.63E+08	5.1E+08	0.940381	0.437381
Q6UB35	Monofunctional C1-tetrahydrofolate syntha	MTHFD1L	1	8	8	9.7	105.79	27680000	21058000	20571000	21527000	19453000	22060000	0.90955	0.43775
Q07157	Tight junction protein ZO-1	TJP1	1	24	24	20.7	195.46	43893000	42372000	36696000	38500000	47323000	46266000	1.074235	0.43855
P41227;Q9BSU3	N-alpha-acetyltransferase 10	NAA10	2	6	6	30.2	26.458	54199000	52033000	45983000	48286000	64323000	53335000	1.090195	0.439318
Q9UHV9	Prefoldin subunit 2	PFDN2	1	7	7	51.9	16.648	90993000	65703000	90241000	1.64E+08	97357000	64105000	1.318118	0.439429
Q13439	Golgin subfamily A member 4	GOLGA4	1	13	13	7.7	261.14	14053000	9759600	14352000	12768000	9616700	11302000	0.882669	0.439483
Q9BXJ9;Q6N069	N-alpha-acetyltransferase 15, NatA auxiliar	NAA15	2	13	13	19.2	101.27	37452000	40654000	45854000	35651000	37007000	42832000	0.931672	0.439606
Q66K14	TBC1 domain family member 9B	TBC1D9B	1	3	3	3	140.52	17577000	26373000	NaN	12070000	20354000	NaN	0.737747	0.4408
P06748	Nucleophosmin	NPM1	1	7	7	28.9	32.575	6.12E+08	7.03E+08	6.37E+08	5.91E+08	6.13E+08	6.61E+08	0.955507	0.441208
P43487	Ran-specific GTPase-activating protein	RANBP1	1	6	6	54.2	23.31	1.17E+08	1.18E+08	1.07E+08	1.07E+08	1.24E+08	1.33E+08	1.061473	0.442802
Q9BQE3	Tubulin alpha-1C chain	TUBA1C	1	21	2	62.6	49.895	2.69E+08	3.34E+08	2.52E+08	2.12E+08	2.36E+08	3.1E+08	0.885239	0.443115
P60842	Eukaryotic initiation factor 4A-I	EIF4A1	1	21	11	61.6	46.153	4.82E+08	4.76E+08	4.58E+08	4.89E+08	4.85E+08	4.67E+08	1.017538	0.443745
Q13144	Translation initiation factor eIF-2B subunit (	EIF2B5	1	3	3	7.1	80.379	NaN	16933000	22753000	16896000	16686000	19466000	0.891129	0.443961
Q15181	Inorganic pyrophosphatase	PPA1	1	16	16	70.2	32.66	1.12E+08	94088000	1.17E+08	86673000	1.05E+08	1.07E+08	0.925244	0.444525
P00338;Q6ZMR3;P07864	L-lactate dehydrogenase A chain	LDHA	3	17	16	44	36.688	1.19E+09	1.27E+09	1.21E+09	1.14E+09	1.11E+09	1.27E+09	0.961676	0.445377
Q9NX40	OCIA domain-containing protein 1	OCIAD1	1	5	5	18.8	27.626	66580000	68636000	73424000	67068000	71133000	62024000	0.959667	0.446311
Q9H3Z4	DnaJ homolog subfamily C member 5	DNAJC5	1	5	5	33.3	22.149	29543000	32652000	29825000	29906000	31647000	35024000	1.049522	0.446411
P98082	Disabled homolog 2	DAB2	1	3	3	4.3	82.447	16728000	NaN	17839000	18700000	19309000	16708000	1.055284	0.447197
P25685	DnaJ homolog subfamily B member 1	DNAJB1	1	7	6	23.5	38.044	43559000	52153000	52771000	44806000	56992000	61174000	1.09758	0.447462
P08648	Integrin alpha-5;Integrin alpha-5 heavy cha	ITGA5	1	5	5	8.5	114.54	33814000	25687000	34417000	33722000	34578000	32833000	1.076822	0.447673
O43633	Charged multivesicular body protein 2a	CHMP2A	1	2	2	8.6	25.104	19271000	16006000	NaN	NaN	17011000	14276000	0.886895	0.447753
O60925	Prefoldin subunit 1	PFDN1	1	4	4	45.1	14.21	29716000	34791000	27423000	33076000	30514000	34601000	1.068106	0.447773
Q99880;Q99879;Q99877;Q93(	Histone H2B type 1-L;Histone H2B type 1-I	HIST1H2BL;HIST1H	11	6	2	41.3	13.952	1.31E+09	1.72E+09	9.67E+08	1.63E+09	1.44E+09	1.48E+09	1.141528	0.449322
P43490	Nicotinamide phosphoribosyltransferase	NAMPT	1	10	10	29.5	55.52	84989000	81057000	68315000	72440000	79482000	66840000	0.93344	0.450273
Q99541	Perilipin-2	PLIN2	1	7	7	24.3	48.075	16657000	15306000	14940000	17921000	19180000	13956000	1.088566	0.450711
P25786	Proteasome subunit alpha type-1	PSMA1	1	8	8	31.6	29.555	1.32E+08	96514000	1.17E+08	98426000	1.09E+08	1.11E+08	0.920887	0.451026
O00399	Dynactin subunit 6	DCTN6	1	2	2	12.6	20.747	NaN	15829000	16925000	NaN	16160000	15313000	0.96089	0.452695
Q9UNH7	Sorting nexin-6;Sorting nexin-6, N-termina	SNX6	1	12	12	27.6	46.648	48327000	46682000	46368000	46467000	47319000	52624000	1.0356	0.452859
P62495	Eukaryotic peptide chain release factor sub	ETF1	1	13	13	41.6	49.03	64608000	48225000	67850000	62808000	68407000	65103000	1.086533	0.45376
P63162;P14678	Small nuclear ribonucleoprotein-associatec	SNRPN;SNRPB	2	4	4	15.4	24.614	56018000	42140000	56790000	71128000	50951000	52761000	1.128379	0.454435
Q6P996	Pyridoxal-dependent decarboxylase domai	PDXDC1	1	3	3	6.2	86.706	11685000	NaN	12575000	11986000	16533000	NaN	1.175556	0.45502
P00533	Epidermal growth factor receptor	EGFR	1	13	13	16.5	134.28	32741000	23999000	28354000	31042000	33589000	27919000	1.087621	0.455304
Q9H444	Charged multivesicular body protein 4b	CHMP4B	1	4	4	22.8	24.95	70341000	78030000	94085000	69510000	75754000	78625000	0.923421	0.455313
P36957	Dihydrolipoyllysine-residue succinyltransfe	DLST	1	8	8	21.2	48.755	96261000	83996000	93980000	95214000	92366000	96432000	1.035644	0.456225
P09936	Ubiquitin carboxyl-terminal hydrolase isozy	UCHL1	1	14	14	79.4	24.824	1.19E+09	9.36E+08	1.17E+09	1.21E+09	1.15E+09	1.14E+09	1.063663	0.456501
Q12874	Splicing factor 3A subunit 3	SF3A3	1	6	6	14	58.848	20483000	25225000	22467000	20263000	22264000	21936000	0.945552	0.45804
Q9BR61	Acyl-CoA-binding domain-containing prot	ACBD6	1	3	3	12.8	31.15	9542400	7993900	NaN	8953500	10705000	NaN	1.121018	0.459826
P60660	Myosin light polypeptide 6	MYL6	1	9	7	73.5	16.93	9.8E+08	8.99E+08	8.52E+08	1.01E+09	9.89E+08	8.71E+08	1.051093	0.460107
Q9NRW7	Vacuolar protein sorting-associated protein	VPS45	1	8	8	17.4	65.076	50911000	51214000	52112000	45832000	44517000	55424000	0.945123	0.460168
Q9Y4P1	Cysteine protease ATG4B	ATG4B	1	4	4	15	44.294	8369100	NaN	9812300	7434600	8105900	9402300	0.91459	0.460372
Q14353	Guanidinoacetate N-methyltransferase	GAMT	1	3	3	21.2	26.318	66413000	48415000	61960000	58772000	63929000	69191000	1.085436	0.461768
Q99832	T-complex protein 1 subunit eta	CCT7	1	22	22	57.8	59.366	1.42E+08	1.29E+08	1.54E+08	1.49E+08	1.59E+08	1.4E+08	1.051429	0.46332
P52907	F-actin-capping protein subunit alpha-1	CAPZA1	1	12	10	65.7	32.922	3.09E+08	3.13E+08	3.18E+08	3E+08	3E+08	3.22E+08	0.980394	0.463881
Q12904	Aminoacyl tRNA synthase complex-interac	AIMP1	1	9	9	49.7	34.352	56130000	61900000	62210000	55170000	52881000	63204000	0.95015	0.463891
Q14376	UDP-glucose 4-epimerase	GALE	1	5	5	17	38.281	24370000	28170000	25232000	27406000	24583000	31193000	1.069562	0.464765
Q9Y371	Endophilin-B1	SH3GLB1	1	6	5	17.8	40.796	78523000	69063000	60847000	63264000	45535000	75330000	0.883397	0.465267
Q9BXF6	Rab11 family-interacting protein 5	RAB11FIP5	1	8	8	20.4	70.414	44401000	37052000	37180000	36519000	42537000	51794000	1.102981	0.466285
Q9BZL4	Protein phosphatase 1 regulatory subunit 1	PPP1R12C	1	4	4	9.5	84.88	NaN	15561000	13244000	14221000	13958000	11501000	0.918359	0.466316
P20645	Cation-dependent mannose-6-phosphate	M6PR	1	3	3	19.1	30.993	16682000	15069000	22593000	16133000	16236000	16467000	0.898646	0.467479
P38117	Electron transfer flavoprotein subunit beta	ETFB	1	12	12	51.8	27.843	91045000	77339000	93383000	89954000	1.01E+08	86671000	1.060772	0.468673
Q12768	WASH complex subunit strumpellin	KIAA0196	1	7	7	8.9	134.28	20810000	28553000	17359000	23121000	26597000	25299000	1.124322	0.46916
Q9Y5X1	Sorting nexin-9	SNX9	1	4	4	8.1	66.591	22814000	21088000	19931000	21334000	21490000	16887000	0.935425	0.470546
Q8WVM8	Sec1 family domain-containing protein 1	SCFD1	1	9	9	18.2	72.379	31879000	26505000	36104000	23146000	29877000	32197000	0.901913	0.470869
P55884	Eukaryotic translation initiation factor 3 sub	EIF3B	1	19	19	29.9	92.48	55875000	52141000	60630000	55356000	50109000	55860000	0.95659	0.470963
P46777	60S ribosomal protein L5	RPL5	1	11	11	33	34.362	2.13E+08	2.37E+08	2.56E+08	2.34E+08	2.1E+08	2.28E+08	0.952023	0.472246
Q7Z478	ATP-dependent RNA helicase DHX29	DHX29	1	8	8	8.4	155.23	15249000	16944000	14730000	15564000	15408000	19514000	1.075933	0.472951
P11766	Alcohol dehydrogenase class-3	ADH5	1	12	12	43	39.724	2.74E+08	2.81E+08	3.58E+08	2.69E+08	2.85E+08	2.93E+08	0.927413	0.473365

P41226	Ubiquitin-like modifier-activating enzyme 7	UBA7	1	13	13	19.4	111.69	22240000	19300000	22410000	19427000	18377000	22415000	0.941658	0.474054
O75976	Carboxypeptidase D	CPD	1	8	8	8.5	152.93	29975000	30530000	NaN	30534000	34939000	30155000	1.053665	0.474947
Q9NVP1	ATP-dependent RNA helicase DDX18	DDX18	1	4	4	6.9	75.406	10870000	11398000	12412000	10327000	13132000	14076000	1.082324	0.476683
Q15008	26S proteasome non-ATPase regulatory su	PSMD6	1	16	16	42.4	45.531	72776000	49591000	62510000	74533000	74835000	56667000	1.114444	0.477227
P51114	Fragile X mental retardation syndrome-rela	FXR1	1	14	12	35.9	69.72	38874000	34762000	44073000	48895000	41457000	37431000	1.085584	0.478951
Q969V3	Nicalin	NCLN	1	8	8	19.7	62.974	35033000	24519000	28830000	32650000	24525000	20005000	0.873255	0.479682
P49458	Signal recognition particle 9 kDa protein	SRP9	1	3	3	36	10.112	57120000	60482000	79943000	98156000	64549000	65492000	1.155165	0.480265
O95202	LETM1 and EF-hand domain-containing pr	LETM1	1	15	15	29.6	83.353	31624000	28350000	30763000	29338000	29566000	41316000	1.104511	0.48121
P08574	Cytochrome c1, heme protein, mitochondr	CYC1	1	5	5	18.5	35.422	95689000	86183000	1.02E+08	91327000	94597000	1.27E+08	1.10065	0.481618
P63244	Guanine nucleotide-binding protein subun	GNB2L1	1	16	16	61.5	35.076	2.62E+08	2.67E+08	3.04E+08	2.59E+08	2.63E+08	2.78E+08	0.96007	0.481713
Q9UHD1	Cysteine and histidine-rich domain-contair	CHORDC1	1	9	9	45.8	37.489	1.64E+08	1.38E+08	1.68E+08	1.19E+08	1.5E+08	1.64E+08	0.920026	0.483977
Q14165	Malectin	MLEC	1	5	5	24	32.233	40387000	34854000	46451000	39737000	39427000	33466000	0.925533	0.484121
P55145	Mesencephalic astrocyte-derived neurotro	MANF	1	7	7	30.8	20.7	48446000	51298000	60703000	56884000	47830000	43122000	0.921401	0.485726
O75390	Citrate synthase, mitochondrial	CS	1	7	7	20.8	51.712	1.39E+08	1.33E+08	1.5E+08	1.39E+08	1.52E+08	1.46E+08	1.034333	0.485735
Q7L576	Cytoplasmic FMR1-interacting protein 1	CYFIP1	1	10	5	10.5	145.18	44627000	50735000	38579000	37934000	42141000	44650000	0.931194	0.487257
Q14258	E3 ubiquitin/ISG15 ligase TRIM25	TRIM25	1	17	17	32.2	70.973	89250000	84218000	85393000	87931000	81572000	83857000	0.978749	0.487733
P34932	Heat shock 70 kDa protein 4	HSPA4	1	32	30	45.5	94.33	1.5E+08	1.56E+08	1.52E+08	1.55E+08	1.48E+08	1.68E+08	1.030944	0.488929
Q9BY43	Charged multivesicular body protein 4a	CHMP4A	1	4	4	22.1	25.098	1.33E+08	89328000	1.58E+08	1.38E+08	1.41E+08	1.49E+08	1.122012	0.48965
Q9BQE5	Apolipoprotein L2	APOL2	1	5	5	17.2	37.078	2.2E+08	2.28E+08	1.9E+08	1.66E+08	1.56E+08	2.46E+08	0.89031	0.489788
P62829	60S ribosomal protein L23	RPL23	1	5	5	50.7	14.865	2.11E+08	2.31E+08	1.78E+08	2.01E+08	2.26E+08	2.36E+08	1.067922	0.490836
P30419	Glycylpeptide N-tetradecanoyltransferase 1	NMT1	1	8	6	21.6	56.806	53448000	52701000	63546000	55972000	54914000	76864000	1.106397	0.491517
P01034	Cystatin-C	CST3	1	2	2	19.2	15.799	NaN	19738000	27968000	28913000	28406000	23259000	1.126036	0.492122
Q9UJS0	Calcium-binding mitochondrial carrier prot	SLC25A13	1	8	4	13.9	74.175	7946000	13338000	10176000	8981400	8842600	NaN	0.849841	0.492532
Q9Y6K9	NF-kappa-B essential modulator	IKBKG	1	4	4	13.4	48.197	NaN	18093000	13547000	15423000	13905000	13885000	0.910514	0.492793
Q9HB90;Q9NQL2	Ras-related GTP-binding protein C;Ras-rel	RRAGC;RRAGD	2	2	2	6.8	44.223	8960800	14178000	NaN	10722000	12765000	42150000	1.891109	0.493261
Q13425	Beta-2-syntrophin	SNTB2	1	5	5	12.2	57.949	15466000	16331000	15054000	16897000	13553000	13847000	0.945487	0.494324
Q9Y2H0	Disks large-associated protein 4	DLGAP4	1	5	5	6.6	108.01	21940000	NaN	26733000	21970000	24426000	21713000	0.932879	0.494563
Q9GZQ8;A6NCE7	Microtubule-associated proteins 1A/1B ligh	MAP1LC3B;MAP1L	2	2	1	16.8	14.688	46364000	41749000	42469000	39925000	48784000	49534000	1.058668	0.494655
P36578	60S ribosomal protein L4	RPL4	1	17	17	37.9	47.697	1.74E+08	1.91E+08	1.96E+08	2.15E+08	1.98E+08	1.77E+08	1.051692	0.494921
Q5T0D9	Tumor protein p63-regulated gene 1-like p	TPRG1L	1	2	2	17.3	30.211	17165000	NaN	12849000	13520000	NaN	12890000	0.879923	0.495529
Q13228	Selenium-binding protein 1	SELENBP1	1	11	11	30.1	52.39	41434000	41719000	42888000	36210000	39009000	44956000	0.95346	0.496457
Q16630	Cleavage and polyadenylation specificity fa	CPSF6	1	6	6	17.6	59.209	21801000	48492000	32927000	25059000	28257000	NaN	0.774792	0.497397
Q99757	Thioredoxin, mitochondrial	TXN2	1	2	2	9.6	18.383	18769000	20042000	22058000	NaN	23193000	20010000	1.064655	0.499579
Q99873;Q9NR22	Protein arginine N-methyltransferase 1	PRMT1	2	11	11	30.7	42.461	52598000	45724000	62611000	59893000	63384000	51176000	1.08401	0.501067
O94973	AP-2 complex subunit alpha-2	AP2A2	1	17	11	22.4	103.96	26617000	22746000	33225000	31565000	28030000	30132000	1.086441	0.501766
Q3KQU3	MAP7 domain-containing protein 1	MAP7D1	1	6	6	10.1	92.819	18049000	19937000	17406000	21682000	16548000	21113000	1.071328	0.503497
Q16795	NADH dehydrogenase [ubiquinone] 1 alph	NDUFA9	1	3	3	10.6	42.509	14534000	NaN	17392000	15005000	12715000	16271000	0.918603	0.503621
Q96CX2	BTB/POZ domain-containing protein KCTD	KCTD12	1	4	4	17.8	35.7	12709000	23640000	17236000	17838000	14817000	13384000	0.859177	0.504246
P51571	Translocon-associated protein subunit delt	SSR4	1	4	4	30.6	18.998	1.03E+08	99189000	85601000	87745000	1.02E+08	80554000	0.937425	0.505727
P18085	ADP-ribosylation factor 4	ARF4	1	7	5	47.8	20.511	4.6E+08	5.5E+08	4.23E+08	4.24E+08	3.96E+08	5.04E+08	0.924392	0.506377
P61956;Q6EEV6	Small ubiquitin-related modifier 2;Small ub	SUMO2;SUMO4	2	2	1	23.2	10.871	1.53E+08	1.88E+08	45800000	1.24E+08	1.84E+08	1.81E+08	1.265302	0.507482
O14879	Interferon-induced protein with tetratricop	IFIT3	1	6	6	20.2	55.984	53998000	43582000	60203000	46287000	47623000	52542000	0.928186	0.508522
O43464;P83110	Serine protease HTRA2, mitochondrial	HTRA2	2	4	4	13.8	48.84	19722000	24319000	NaN	22793000	22089000	27204000	1.091195	0.508686
Q6PIU2	Neutral cholesterol ester hydrolase 1	NCEH1	1	7	7	27.7	45.807	80545000	92595000	73873000	88843000	82994000	87696000	1.050686	0.509071
Q9UL15	BAG family molecular chaperone regulator	BAG5	1	1	1	2.5	51.199	49886000	12791000	27151000	22210000	18760000	25130000	0.735851	0.510248
O43399	Tumor protein D54	TPD52L2	1	13	13	58.7	22.237	2.9E+08	2.85E+08	3.01E+08	3.11E+08	2.79E+08	3.12E+08	1.029669	0.511171
O60256	Phosphoribosyl pyrophosphate synthase-a	PRPSAP2	1	5	4	22.8	40.925	33832000	35864000	34856000	32744000	37838000	37854000	1.037149	0.511539
P52434	DNA-directed RNA polymerases I, II, and II	POLR2H	1	2	2	14	17.143	18876000	NaN	17997000	17755000	19018000	24740000	1.11216	0.512843
O75955	Flotillin-1	FLOT1	1	10	10	32.3	47.355	78932000	85667000	67950000	80682000	82733000	80270000	1.047887	0.516215
P25787	Proteasome subunit alpha type-2	PSMA2	1	7	7	44.4	25.898	63628000	62837000	74667000	89888000	59067000	72802000	1.102545	0.517516
Q9UHY1	Nuclear receptor-binding protein	NRBP1	1	7	7	19.8	59.844	45717000	34032000	61339000	36201000	44573000	42652000	0.874816	0.517545
Q9UBT2	SUMO-activating enzyme subunit 2	UBA2	1	15	15	32	71.223	1.07E+08	85018000	91626000	90482000	98100000	75595000	0.929762	0.517689
Q9NR31	GTP-binding protein SAR1a	SAR1A	1	6	5	51.5	22.367	39458000	38881000	59102000	42989000	38572000	41531000	0.895599	0.518979
O14737	Programmed cell death protein 5	PDCD5	1	5	5	46.4	14.285	61647000	35161000	54472000	64097000	49951000	56080000	1.12459	0.51947
O15498	Synaptobrevin homolog YKT6	YKT6	1	2	2	17.7	22.417	11032000	18500000	17237000	20331000	15186000	17071000	1.12442	0.520082
P62280	40S ribosomal protein S11	RPS11	1	12	12	56.3	18.431	2.53E+08	2.88E+08	2.5E+08	2.97E+08	2.55E+08	2.76E+08	1.045567	0.520104
Q9Y3U8	60S ribosomal protein L36	RPL36	1	5	5	30.5	12.254	1.36E+08	1.14E+08	1.83E+08	1.25E+08	1.48E+08	1.12E+08	0.88785	0.520974
O96019;O94805	Actin-like protein 6A	ACTL6A	2	5	5	19.3	47.46	21070000	30488000	23670000	32719000	23959000	26603000	1.107048	0.521293
Q92769	Histone deacetylase 2	HDAC2	1	4	3	14.8	55.364	29649000	23157000	30389000	25762000	29243000	21406000	0.918457	0.522142

O95373;O15397	Importin-7	IPO7	2	15	15	20.1	119.52	75184000	94550000	83119000	71111000	78137000	87962000	0.938134	0.522448
Q9UGR2;Q8IWR0	Zinc finger CCCH domain-containing prote	ZC3H7B	2	4	4	5.9	109.86	14380000	16037000	14808000	18028000	15310000	14399000	1.055544	0.523039
Q9BTU6	Phosphatidylinositol 4-kinase type 2-alpha	PI4K2A	1	7	7	19.4	54.022	16981000	16701000	17906000	14588000	19722000	21872000	1.089052	0.52314
P51148	Ras-related protein Rab-5C	RAB5C	1	10	7	71.8	23.482	1.29E+08	1.89E+08	1.51E+08	1.75E+08	1.59E+08	1.73E+08	1.080778	0.524444
Q05209	Tyrosine-protein phosphatase non-recepto	PTPN12	1	6	6	10.3	88.105	16815000	NaN	19631000	18368000	14301000	NaN	0.896367	0.52492
Q96C36	Pyrroline-5-carboxylate reductase 2	PYCR2	1	4	4	19.7	33.637	NaN	16239000	15339000	14215000	13108000	16874000	0.933076	0.527086
P61457	Pterin-4-alpha-carbinolamine dehydratase	PCBD1	1	4	4	45.2	11.999	14292000	13625000	10199000	8120800	14509000	10836000	0.877999	0.52764
P05141	ADP/ATP translocase 2;ADP/ATP translocas	SLC25A5	1	6	3	18.5	32.852	94246000	1.15E+08	1.05E+08	1.07E+08	1E+08	1.31E+08	1.072681	0.528158
O15173	Membrane-associated progesterone recep	PGRMC2	1	7	6	26.5	23.818	60603000	52179000	60880000	54781000	59264000	52384000	0.95835	0.528278
P46782	40S ribosomal protein S5;40S ribosomal pr	RPS5	1	9	9	42.6	22.876	2.64E+08	4.09E+08	3.41E+08	3E+08	2.86E+08	3.37E+08	0.909163	0.529237
Q9BQ70	Transcription factor 25	TCF25	1	2	2	4.9	76.666	12066000	NaN	11838000	11552000	NaN	14807000	1.102702	0.530319
Q96EK5	KIF1-binding protein	KIAA1279	1	4	4	9.5	71.813	21672000	20777000	24182000	13611000	24703000	NaN	0.862526	0.532241
P48506	Glutamate--cysteine ligase catalytic subuni	GCLC	1	7	7	16.2	72.765	NaN	1.19E+09	1.48E+09	NaN	8.37E+08	1.38E+09	0.829026	0.534773
Q99471	Prefoldin subunit 5	PFDN5	1	4	4	32.5	17.328	52097000	47870000	51985000	46539000	55546000	57143000	1.047884	0.535471
P16278	Beta-galactosidase	GLB1	1	7	7	15.1	76.074	66304000	58480000	60727000	56785000	63563000	58957000	0.966546	0.536887
Q86Y82	Syntaxin-12	STX12	1	6	6	30.1	31.642	60996000	68005000	56227000	59768000	61164000	77991000	1.073936	0.537732
P22061	Protein-L-isoaspartate(D-aspartate) O-me	PCMT1	1	8	8	46.7	24.636	4.46E+08	5.22E+08	4.77E+08	4.66E+08	5.18E+08	5.17E+08	1.038784	0.538271
Q15102	Platelet-activating factor acetylhydrolase IB	PAFAH1B3	1	2	2	18.2	25.734	16515000	20811000	NaN	21108000	19732000	18897000	1.066942	0.538474
O75436	Vacuolar protein sorting-associated protei	VPS26A	1	12	12	52.9	38.169	58916000	58041000	55579000	59444000	63851000	54843000	1.032469	0.539384
Q9BZZ5	Apoptosis inhibitor 5	API5	1	5	5	10.3	59.004	23737000	20610000	24734000	24707000	24166000	22922000	1.039287	0.539486
Q9BZF1	Oxysterol-binding protein-related protein	OSBPL8	1	5	5	8.7	101.19	18393000	15319000	20754000	18959000	16308000	15368000	0.929663	0.539494
P24385	G1/S-specific cyclin-D1	CCND1	1	3	3	13.6	33.729	26661000	24350000	23887000	20335000	24904000	25847000	0.949104	0.541488
P22102	Trifunctional purine biosynthetic protein ad	GART	1	13	13	15.9	107.77	28507000	26590000	25077000	29413000	29322000	24964000	1.043967	0.543544
P18583	Protein SON	SON	1	6	6	3.3	263.83	16168000	23170000	NaN	14414000	18899000	NaN	0.84684	0.544015
Q16775	Hydroxyacylglutathione hydrolase, mitocho	HAGH	1	6	6	27.3	33.805	53435000	73092000	62261000	63733000	64547000	73335000	1.067944	0.544375
P62258	14-3-3 protein epsilon	YWHAE	1	24	21	77.6	29.174	7.75E+08	7.4E+08	7.21E+08	7.35E+08	7.68E+08	7.71E+08	1.017346	0.54508
Q92900	Regulator of nonsense transcripts 1	UPF1	1	15	15	19.5	124.34	26108000	27088000	38720000	27353000	30767000	25152000	0.905958	0.545485
Q13049	E3 ubiquitin-protein ligase TRIM32	TRIM32	1	5	5	12.9	71.988	40886000	41208000	41672000	42214000	46140000	39338000	1.031721	0.545645
Q13616	Cullin-1	CUL1	1	6	6	12.5	89.677	19709000	35223000	25802000	11852000	18033000	34706000	0.800047	0.546684
Q08431	Lactadherin;Lactadherin short form;Medin	MFGE8	1	5	5	16.8	43.104	35447000	32757000	31329000	32895000	31464000	42189000	1.070479	0.548455
O95786	Probable ATP-dependent RNA helicase DD	DDX58	1	7	7	8.5	106.6	51396000	NaN	64680000	37700000	29646000	71794000	0.799132	0.550739
Q9Y3Z3	Deoxynucleoside triphosphate triphosphoh	SAMHD1	1	14	14	28	72.2	45986000	36405000	38154000	37373000	40326000	36701000	0.949023	0.550877
Q92905	COP9 signalosome complex subunit 5	COPS5	1	6	6	23.7	37.578	46507000	36982000	48858000	50639000	44593000	45125000	1.060523	0.551644
P62304	Small nuclear ribonucleoprotein E	SNRPE	1	2	2	40.2	10.803	NaN	86983000	65803000	65322000	NaN	71843000	0.897759	0.553913
P17987	T-complex protein 1 subunit alpha	TCP1	1	27	27	62.9	60.343	1.56E+08	1.66E+08	1.58E+08	1.6E+08	1.58E+08	1.77E+08	1.02753	0.554967
P63104	14-3-3 protein zeta/delta	YWHAZ	1	20	15	73.1	27.745	1.07E+09	1.22E+09	1.12E+09	9.36E+08	1.09E+09	1.21E+09	0.948106	0.555281
Q9NTK5	Obg-like ATPase 1	OLA1	1	8	8	26.8	44.743	36452000	33794000	49765000	49520000	43742000	38169000	1.095158	0.555765
P62244	40S ribosomal protein S15a	RPS15A	1	4	4	39.2	14.839	2.87E+08	2.61E+08	4.05E+08	2.86E+08	3.2E+08	2.54E+08	0.90242	0.555987
P50570	Dynamin-2	DNM2	1	17	15	28.7	98.063	41187000	44291000	48579000	40740000	42335000	45918000	0.962225	0.556452
Q05519	Serine/arginine-rich splicing factor 11	SRSF11	1	4	4	13.6	53.542	16488000	18610000	NaN	15921000	17377000	NaN	0.948715	0.556678
P05387	60S acidic ribosomal protein P2	RPLP2	1	7	7	60.9	11.665	7.21E+08	4.63E+08	6.5E+08	6.28E+08	5.6E+08	4.74E+08	0.90673	0.557029
P27348	14-3-3 protein theta	YWHAQ	1	18	13	62.9	27.764	2.72E+08	2.89E+08	2.48E+08	2.64E+08	2.82E+08	2.9E+08	1.033345	0.557165
Q9UBS8	E3 ubiquitin-protein ligase RNF14	RNF14	1	4	4	9.5	53.837	7424300	10565000	NaN	7604200	8161200	NaN	0.876377	0.557814
P61019;Q8WUD1	Ras-related protein Rab-2A;Ras-related pr	RAB2A;RAB2B	2	10	10	55.2	23.545	1.07E+08	1.06E+08	1.08E+08	1.06E+08	1.17E+08	1.06E+08	1.022624	0.557898
Q13347	Eukaryotic translation initiation factor 3 sub	EIF3I	1	10	10	48.6	36.501	1.28E+08	1.36E+08	1.34E+08	93425000	1.21E+08	1.51E+08	0.919218	0.559122
Q14699	Raftlin	RFTN1	1	4	4	12.6	63.145	19457000	22257000	21477000	23743000	17664000	17579000	0.933456	0.55947
Q9NZ01	Very-long-chain enoyl-CoA reductase	TECR	1	4	4	14.9	36.034	1.17E+08	1.24E+08	1.31E+08	1.19E+08	1.3E+08	1.09E+08	0.962402	0.56089
P13804	Electron transfer flavoprotein subunit alpha	ETFA	1	13	13	53.2	35.079	1.5E+08	1.53E+08	1.6E+08	1.5E+08	1.62E+08	1.59E+08	1.018998	0.562987
P27816	Microtubule-associated protein 4	MAP4	1	45	45	48	121	2.86E+08	3.06E+08	3.2E+08	2.65E+08	3.03E+08	3.11E+08	0.9643	0.5632
P49821	NADH dehydrogenase [ubiquinone] flavop	NDUFV1	1	8	8	21.3	50.817	32138000	38673000	30578000	30594000	34397000	31233000	0.949058	0.56444
Q9H0U4;Q92928	Ras-related protein Rab-1B	RAB1B	2	11	5	66.7	22.171	2.1E+08	2.28E+08	1.99E+08	2.15E+08	2.19E+08	2.2E+08	1.025232	0.565347
Q9UEW8	STE20/SPS1-related proline-alanine-rich p	STK39	1	6	4	14.3	59.473	27670000	27881000	23112000	21959000	23368000	28569000	0.9394	0.565764
Q08J23	tRNA (cytosine(34)-C(5))-methyltransferase	NSUN2	1	7	7	14.6	86.47	17743000	13434000	18577000	16282000	15438000	14972000	0.938457	0.567222
P13861;P31323	cAMP-dependent protein kinase type II-alp	PRKAR2A	2	7	7	24.3	45.518	25789000	27755000	NaN	28683000	37863000	23973000	1.127036	0.567818
P46087	Probable 28S rRNA (cytosine(4447)-C(5))-r	NOP2	1	9	9	14.2	89.301	13925000	15401000	16463000	13750000	16589000	12997000	0.946428	0.568656
Q5JRA6	Melanoma inhibitory activity protein 3	MIA3	1	15	15	10.6	213.7	24533000	16570000	24818000	23375000	23537000	24042000	1.076349	0.569463
P54136	Arginine--tRNA ligase, cytoplasmic	RARS	1	25	25	45.5	75.378	1.08E+08	1.29E+08	1.06E+08	1.28E+08	1.15E+08	1.15E+08	1.045299	0.569513
O94874	E3 UFM1-protein ligase 1	UFL1	1	6	6	9.6	89.594	7236100	18787000	10793000	20224000	11117000	13559000	1.219575	0.570612
P29323;P54762	Ephrin type-B receptor 2	EPHB2	2	8	7	11.2	117.49	19654000	18759000	16782000	16141000	17199000	19462000	0.956645	0.571495

P40763	Signal transducer and activator of transcrip	STAT3	1	13	13	25.2	88.067	41658000	41871000	39487000	34071000	34697000	46612000	0.937927	0.57286
Q7Z3J2	UPF0505 protein C16orf62	C16orf62	1	3	3	5.7	109.56	15210000	17222000	20158000	21758000	17245000	17383000	1.072181	0.572977
Q14008	Cytoskeleton-associated protein 5	CKAP5	1	21	21	14.6	225.49	35114000	40259000	31755000	31633000	31133000	38249000	0.942937	0.578308
Q9UHQ9	NADH-cytochrome b5 reductase 1	CYB5R1	1	9	9	36.4	34.094	2.66E+08	2.17E+08	3.1E+08	2.46E+08	2.21E+08	2.71E+08	0.930626	0.578335
P22234	Multifunctional protein ADE2;Phosphoribos	PAICS	1	12	12	32.9	47.079	75812000	70872000	96333000	88832000	86348000	82370000	1.059802	0.57837
P28066	Proteasome subunit alpha type-5	PSMA5	1	7	7	46.5	26.411	1.26E+08	1.28E+08	1.32E+08	1.22E+08	1.25E+08	1.64E+08	1.064447	0.578797
Q02543	60S ribosomal protein L18a	RPL18A	1	5	5	30.1	20.762	1.98E+08	1.36E+08	1.48E+08	2.02E+08	2.13E+08	1.27E+08	1.123938	0.579056
O43865;Q96HN2	Putative adenosylhomocysteinase 2;Putativ	AHCYL1;AHCYL2	2	8	8	22.1	58.951	49672000	50888000	39487000	47749000	50227000	48734000	1.047577	0.579283
Q9P2J5	Leucine--tRNA ligase, cytoplasmic	LARS	1	24	24	24.1	134.46	36776000	41602000	37221000	41820000	37523000	39817000	1.030805	0.580768
Q02750	Dual specificity mitogen-activated protein I	MAP2K1	1	7	4	21.6	43.439	37417000	26988000	33962000	37421000	36933000	30740000	1.068387	0.581677
O60869	Endothelial differentiation-related factor 1	EDF1	1	3	3	26.4	16.368	38064000	44776000	33295000	37067000	30604000	40548000	0.931838	0.583039
P43304	Glycerol-3-phosphate dehydrogenase, mit	GPD2	1	12	12	20.4	80.852	31549000	35074000	31757000	35642000	36966000	30055000	1.043535	0.584857
O95721	Synaptosomal-associated protein 29	SNAP29	1	5	5	26.7	28.97	16237000	27932000	30778000	31170000	24975000	27342000	1.113947	0.585149
Q15370	Transcription elongation factor B polypepti	TCEB2	1	5	5	44.9	13.133	1.22E+08	1.43E+08	1.08E+08	1.37E+08	1.49E+08	1.13E+08	1.068221	0.585828
P34897;P34896	Serine hydroxymethyltransferase, mitochon	SHMT2	2	16	16	41.9	55.992	1.82E+08	1.91E+08	1.63E+08	1.68E+08	1.72E+08	1.79E+08	0.970981	0.585968
P20618	Proteasome subunit beta type-1	PSMB1	1	6	6	34.9	26.489	85391000	89536000	85559000	92551000	81854000	78039000	0.969127	0.587402
Q92572	AP-3 complex subunit sigma-1	AP3S1	1	4	4	26.4	21.732	36185000	30101000	36551000	16283000	34197000	39432000	0.874316	0.587493
A1L0T0	Acetolactate synthase-like protein	ILVBL	1	2	2	3.3	67.867	58222000	58710000	35298000	38463000	41312000	55844000	0.890882	0.58803
P00367;P49448	Glutamate dehydrogenase 1, mitochondria	GLUD1;GLUD2	2	13	13	32.6	61.397	1.48E+08	1.68E+08	1.62E+08	1.46E+08	1.51E+08	1.66E+08	0.969165	0.588204
Q6PKG0	La-related protein 1	LARP1	1	6	6	7.8	123.51	23173000	NaN	23299000	24894000	21464000	27339000	1.057224	0.588265
O43488;Q8NHP1	Aflatoxin B1 aldehyde reductase member 2	AKR7A2	2	6	6	26.5	39.589	56390000	64645000	82315000	71693000	72319000	72831000	1.066354	0.588343
Q6P9B6	TLD domain-containing protein 1	TLDC1	1	5	5	16.2	50.993	38358000	45114000	29329000	45917000	40676000	35775000	1.084813	0.588542
P28065	Proteasome subunit beta type-9	PSMB9	1	2	2	9.6	23.264	43675000	NaN	41501000	33185000	44584000	NaN	0.913039	0.588621
P50579	Methionine aminopeptidase 2	METAP2	1	7	7	20.5	52.891	30093000	35537000	36306000	35182000	37410000	33352000	1.039319	0.589442
Q9UM00	Transmembrane and coiled-coil domain-co	TMCO1	1	3	3	10	27.079	23683000	26389000	37998000	34578000	26947000	35604000	1.102861	0.590567
Q9Y547	Intraflagellar transport protein 25 homolog	HSPB11	1	2	2	25	16.297	21615000	16865000	20563000	20195000	19310000	22587000	1.05164	0.590806
O60884	DnaJ homolog subfamily A member 2	DNAJA2	1	8	8	25.2	45.745	69524000	84489000	63687000	54910000	66328000	79844000	0.923666	0.591236
Q9Y5K5	Ubiquitin carboxyl-terminal hydrolase isozy	UCHL5	1	6	6	27.7	37.606	34159000	34734000	39238000	38894000	31671000	44723000	1.066188	0.592069
P62195;Q8NB90	26S protease regulatory subunit 8	PSMC5	2	18	18	53.4	45.626	88593000	81028000	73447000	84588000	78364000	69225000	0.955194	0.592306
P52788	Spermine synthase	SMS	1	8	8	29.2	41.268	34625000	30914000	36586000	34168000	32638000	40333000	1.049097	0.592878
P52565	Rho GDP-dissociation inhibitor 1	ARHGDIA	1	9	9	71.1	23.207	7.41E+08	5.15E+08	6.85E+08	6.06E+08	6.59E+08	5.45E+08	0.93245	0.59289
P62263	40S ribosomal protein S14	RPS14	1	7	7	38.4	16.273	5.02E+08	4.6E+08	5.08E+08	4.46E+08	4.93E+08	4.92E+08	0.974258	0.593798
P14854	Cytochrome c oxidase subunit 6B1	COX6B1	1	4	4	57	10.192	67628000	55791000	75446000	63697000	68583000	54280000	0.938124	0.5939
P21964	Catechol O-methyltransferase	COMT	1	7	7	42.4	30.037	1.25E+08	1.63E+08	1.22E+08	1.2E+08	1.18E+08	1.45E+08	0.933018	0.594662
P10606	Cytochrome c oxidase subunit 5B, mitocho	COX5B	1	5	5	25.6	13.696	84435000	67199000	89485000	78509000	93055000	83354000	1.057229	0.595561
Q96EY1	DnaJ homolog subfamily A member 3, mito	DNAJA3	1	5	5	11.9	52.488	16065000	19485000	18218000	15332000	19764000	22780000	1.076402	0.596199
P40429;Q6NVV1	60S ribosomal protein L13a	RPL13A	2	7	7	31	23.577	3.04E+08	2.31E+08	3.11E+08	3.09E+08	3.19E+08	2.69E+08	1.060466	0.598481
O75396	Vesicle-trafficking protein SEC22b	SEC22B	1	11	11	53	24.74	2.94E+08	3.05E+08	3.14E+08	2.91E+08	3.08E+08	3.4E+08	1.028704	0.599298
Q7L5N1	COP9 signalosome complex subunit 6	COPS6	1	10	10	55	36.163	39218000	40203000	45637000	40612000	42238000	46889000	1.037431	0.59981
P20337	Ras-related protein Rab-3B	RAB3B	1	6	5	31.5	24.758	84590000	96245000	83649000	76405000	88641000	89457000	0.962262	0.599991
Q9H993	Protein-glutamate O-methyltransferase	ARMT1	1	5	5	13.8	51.172	35673000	34377000	50168000	41323000	39057000	50181000	1.086035	0.601798
Q13724	Mannosyl-oligosaccharide glucosidase	MOGS	1	7	7	12.9	91.916	33181000	39888000	31250000	37350000	32352000	40653000	1.057861	0.602151
P14735	Insulin-degrading enzyme	IDE	1	7	7	8.3	117.97	39386000	17947000	28594000	25776000	30237000	17780000	0.858787	0.603393
P51812;Q15349;Q15418	Ribosomal protein S6 kinase alpha-3	RPS6KA3	3	4	4	7.8	83.735	28668000	30414000	24737000	25465000	30871000	31859000	1.052208	0.604911
P23497;Q9H930	Nuclear autoantigen Sp-100	SP100	2	6	5	9.9	100.42	25896000	27346000	24045000	22972000	26763000	32408000	1.062831	0.606867
P28482	Mitogen-activated protein kinase 1	MAPK1	1	15	12	53.1	41.389	1.4E+08	1.36E+08	1.39E+08	1.32E+08	1.48E+08	1.45E+08	1.020766	0.606997
P26368	Splicing factor U2AF 65 kDa subunit	U2AF2	1	6	6	23.8	53.5	53799000	56795000	84657000	54741000	55190000	67510000	0.908784	0.607875
O15212	Prefoldin subunit 6	PFDN6	1	3	3	27.9	14.582	35519000	41166000	37214000	NaN	40883000	37836000	1.036695	0.608008
Q53H82	Beta-lactamase-like protein 2	LACTB2	1	5	5	20.8	32.805	16819000	19132000	21766000	18264000	15880000	20364000	0.944401	0.608719
Q6PGP7	Tetratricopeptide repeat protein 37	TTC37	1	7	7	7.2	175.48	12446000	16339000	14692000	13406000	12885000	15029000	0.950388	0.609636
Q16706	Alpha-mannosidase 2	MAN2A1	1	7	7	7.4	131.14	16563000	21571000	20963000	17859000	17874000	20412000	0.950048	0.612045
O14818;Q8TAA3	Proteasome subunit alpha type-7	PSMA7	2	10	10	55.6	27.887	1.69E+08	1.58E+08	1.81E+08	1.68E+08	1.79E+08	1.74E+08	1.02429	0.612059
Q15819	Ubiquitin-conjugating enzyme E2 variant 2	UBE2V2	1	6	3	50.3	16.363	1.02E+08	1.34E+08	1.62E+08	1.28E+08	1.2E+08	1.22E+08	0.9269	0.612893
Q9UQE7	Structural maintenance of chromosomes pı	SMC3	1	3	3	3.5	141.54	11653000	NaN	9927400	NaN	10769000	12106000	1.05999	0.613277
P26440	Isovaleryl-CoA dehydrogenase, mitochond	IVD	1	5	5	13.8	46.65	30133000	32272000	27620000	25615000	26292000	33451000	0.948159	0.613527
O60610	Protein diaphanous homolog 1	DIAPH1	1	10	10	11.6	141.35	21213000	21594000	NaN	16135000	19500000	23766000	0.925098	0.613539
O00291	Huntingtin-interacting protein 1	HIP1	1	7	7	8.4	116.22	22427000	20897000	23503000	18527000	21073000	24246000	0.955392	0.613889
O96008	Mitochondrial import receptor subunit TON	TOMM40	1	6	6	27.4	37.893	30013000	26724000	33643000	36696000	31717000	27435000	1.0605	0.614237
Q99614	Tetratricopeptide repeat protein 1	TTC1	1	5	5	18.5	33.526	21447000	16659000	15810000	24356000	26079000	11511000	1.148935	0.615279

P0DMV9;P0DMV8	Heat shock 70 kDa protein 1B;Heat shock 7	HSPA1B;HSPA1A	2	25	12	54.6	70.051	1.55E+08	1.69E+08	1.53E+08	1.6E+08	1.54E+08	1.78E+08	1.029396	0.616313
Q7Z6Z7	E3 ubiquitin-protein ligase HUWE1	HUWE1	1	9	9	2.8	481.89	16459000	12987000	16671000	15039000	16930000	16284000	1.046317	0.617433
O00505	Importin subunit alpha-4	KPNA3	1	9	6	25.7	57.81	42264000	46335000	39098000	33677000	41259000	46071000	0.94761	0.621271
Q6ZUT6	Uncharacterized protein C15orf52	C15orf52	1	4	4	12.4	57.324	18815000	28577000	22144000	24459000	21502000	18142000	0.921868	0.622185
Q13488	V-type proton ATPase 116 kDa subunit a is	TCIRG1	1	8	8	12.5	92.967	18581000	25025000	24397000	27101000	23529000	21552000	1.061453	0.622621
P29992;P50148	Guanine nucleotide-binding protein subun	GNA11	2	11	9	35.9	42.123	42159000	49585000	49988000	42645000	44673000	49282000	0.963791	0.622804
Q9UBB6	Neurochondrin	NCDN	1	5	5	9.6	78.863	NaN	44530000	42708000	43660000	44818000	NaN	1.014214	0.623705
P02462	Collagen alpha-1(IV) chain;Arresten	COL4A1	1	3	1	2.3	160.61	10787000	15344000	NaN	9650500	13188000	NaN	0.874	0.625758
O76071	Probable cytosolic iron-sulfur protein asser	CIAO1	1	5	5	20.9	37.84	18453000	14886000	14040000	16202000	17317000	16087000	1.047004	0.625919
Q15286	Ras-related protein Rab-35	RAB35	1	5	5	33.3	23.025	35326000	24865000	34048000	29019000	34870000	36676000	1.067127	0.627987
O43172	U4/U6 small nuclear ribonucleoprotein Prp	PRPF4	1	4	4	11.7	58.449	16551000	11395000	15296000	10360000	13835000	15609000	0.920494	0.628081
Q14155;Q15052	Rho guanine nucleotide exchange factor 7;	ARHGEF7;ARHGEF	2	3	3	5.9	90.011	19765000	22214000	23453000	15846000	21196000	24198000	0.935933	0.628652
P62826	GTP-binding nuclear protein Ran	RAN	1	8	8	33.3	24.423	5.41E+08	4.75E+08	5.43E+08	5.7E+08	5.47E+08	4.92E+08	1.032364	0.628827
Q99700	Ataxin-2	ATXN2	1	5	5	5.5	140.28	9425200	11323000	NaN	NaN	10847000	10976000	1.051802	0.628977
O95817	BAG family molecular chaperone regulator	BAG3	1	21	21	47.3	61.594	76736000	1.03E+08	87269000	90261000	90409000	98637000	1.047172	0.629353
Q93050	V-type proton ATPase 116 kDa subunit a is	ATP6V0A1	1	8	8	12.9	96.412	52312000	71977000	65590000	58540000	63899000	81552000	1.074321	0.630597
P45974;Q92995	Ubiquitin carboxyl-terminal hydrolase 5	USP5	2	18	18	28	95.785	74217000	79499000	69030000	73003000	72082000	72956000	0.978877	0.632804
P49902	Cytosolic purine 5-nucleotidase	NT5C2	1	6	6	14.4	64.969	74804000	72863000	76230000	67917000	87091000	77591000	1.038866	0.63301
O75165	DnaJ homolog subfamily C member 13	DNAJC13	1	18	18	11.5	254.41	27937000	26569000	31970000	22853000	29641000	29676000	0.950206	0.633847
Q04446	1,4-alpha-glucan-branching enzyme	GBE1	1	11	11	22.8	80.473	96662000	1.3E+08	86505000	93544000	99634000	99617000	0.934352	0.634725
Q15599	Na(+)/H(+) exchange regulatory cofactor N	SLC9A3R2	1	6	6	19.9	37.413	19357000	14946000	17682000	18210000	17717000	12771000	0.93677	0.638774
O75340	Programmed cell death protein 6	PDCD6	1	4	4	20.9	21.868	34309000	34006000	26526000	29560000	30154000	31189000	0.958478	0.638874
P63096	Guanine nucleotide-binding protein G(i) su	GNAI1	1	6	3	20.1	40.361	7378000	8440700	7398800	8380300	7455600	8048400	1.02872	0.642191
Q15046	Lysine--tRNA ligase	KARS	1	15	15	29	68.047	55084000	56303000	58161000	54619000	59369000	52129000	0.979764	0.645722
Q9NP97;Q8TF09	Dynein light chain roadblock-type 1;Dyneir	DYNLRB1;DYNLRB	2	3	3	38.5	10.921	1.08E+08	1.03E+08	1.11E+08	1.06E+08	1.14E+08	1.08E+08	1.015541	0.64588
Q53FA7	Quinone oxidoreductase PIG3	TP53I3	1	5	5	28	35.536	39166000	36322000	38451000	38108000	40182000	31639000	0.964806	0.647784
P61923	Coatomer subunit zeta-1	COPZ1	1	3	3	22.6	20.198	45999000	53604000	49104000	47583000	46789000	50652000	0.975233	0.649384
Q8NBJ5	Procollagen galactosyltransferase 1	COLGALT1	1	7	7	12.9	71.635	81135000	85116000	96059000	76758000	90170000	86563000	0.966379	0.649672
Q14789	Golgin subfamily B member 1	GOLGB1	1	11	11	5.1	376.01	32597000	34272000	27409000	27688000	30242000	32661000	0.960892	0.650759
Q14160;Q9BTT6	Protein scribble homolog	SCRIB	2	12	12	12.1	174.91	58685000	67524000	86952000	65638000	69895000	96193000	1.087094	0.651276
Q9Y3D0	Mitotic spindle-associated MMXD complex	FAM96B	1	4	4	50.3	17.663	26075000	33986000	20891000	21323000	24753000	28531000	0.92162	0.651477
Q8IUD2;O15083	ELKS/Rab6-interacting/CAST family membe	ERC1	2	13	13	13.4	128.08	12607000	18410000	17195000	15027000	15543000	15047000	0.946175	0.651536
P30566	Adenylosuccinate lyase	ADSL	1	3	3	8.7	54.889	21440000	19903000	21241000	19931000	23139000	NaN	1.032293	0.651638
Q9BPW8	Protein NipSnap homolog 1	NIPSNAP1	1	3	3	13.7	33.31	90741000	1.42E+08	72553000	99845000	1.04E+08	1.36E+08	1.113592	0.652931
Q9H2G2	STE20-like serine/threonine-protein kinase	SLK	1	12	11	13	142.69	57638000	59826000	45650000	43587000	64688000	67575000	1.07808	0.652962
P61326;Q96A72	Protein mago nashi homolog;Protein mago	MAGOH;MAGOHE	2	6	6	56.2	17.163	49582000	51395000	76343000	42350000	43845000	72231000	0.893447	0.653427
A0AVT1	Ubiquitin-like modifier-activating enzyme (	UBA6	1	14	14	19.6	117.97	39209000	42643000	43001000	45493000	41370000	40770000	1.022266	0.65365
Q9HD26	Golgi-associated PDZ and coiled-coil moti	GOPC	1	5	5	14.7	50.519	15988000	21953000	21584000	20160000	17380000	18955000	0.949097	0.654312
O75306	NADH dehydrogenase [ubiquinone] iron-s	NDUFS2	1	4	4	10.6	52.545	NaN	12849000	31062000	NaN	27794000	25648000	1.217053	0.655063
Q9Y6R0	Numb-like protein	NUMBL	1	2	2	2	64.891	33058000	22527000	NaN	23722000	26241000	NaN	0.898858	0.655359
O60784	Target of Myb protein 1	TOM1	1	7	7	20.7	53.818	18131000	21619000	20258000	20791000	16662000	20190000	0.960589	0.655801
Q53GQ0	Very-long-chain 3-oxoacyl-CoA reductase	HSD17B12	1	9	9	23.1	34.324	53649000	62215000	59573000	55013000	55955000	60167000	0.975478	0.656285
O75935	Dynactin subunit 3	DCTN3	1	4	4	24.7	21.119	31982000	24028000	33229000	29834000	37856000	27647000	1.068333	0.656318
P24390;P33947	ER lumen protein-retaining receptor 1;ER lu	KDELR1;KDELR2	2	2	2	14.2	24.542	25207000	29747000	NaN	33090000	24981000	29463000	1.061906	0.657455
Q15262;P28827	Receptor-type tyrosine-protein phosphata	PTPRK	2	7	7	8.7	162.1	11786000	12063000	13697000	12447000	11725000	15060000	1.044905	0.657523
Q96PK6	RNA-binding protein 14	RBM14	1	8	8	13.6	69.491	49472000	66319000	64856000	57692000	56188000	58966000	0.956816	0.658051
Q9BRP8	Partner of Y14 and mago	WIBG	1	2	2	17.2	22.655	13421000	10322000	12767000	8543700	11523000	13860000	0.929244	0.658121
Q13596	Sorting nexin-1	SNX1	1	11	9	28.2	59.069	26989000	24523000	32239000	27756000	21375000	29765000	0.942031	0.659103
O95816	BAG family molecular chaperone regulator	BAG2	1	11	11	61.6	23.772	88644000	97080000	1.02E+08	91862000	1.03E+08	1E+08	1.02594	0.659362
P22087	rRNA 2-O-methyltransferase fibrillarin	FBL	1	10	10	41.7	33.784	97860000	63582000	86938000	90646000	89241000	83265000	1.059473	0.659372
O75962;O60229	Triple functional domain protein	TRIO	2	12	12	5.2	346.9	89140000	85633000	55649000	82223000	78351000	85248000	1.066834	0.659741
Q15185	Prostaglandin E synthase 3	PTGES3	1	5	5	30	18.697	1.95E+08	3.4E+08	3.69E+08	2.38E+08	2.65E+08	3.17E+08	0.907688	0.6599
Q15036	Sorting nexin-17	SNX17	1	4	4	10.9	52.901	11032000	12730000	18848000	14698000	14830000	16565000	1.081741	0.66011
Q9BYX2	TBC1 domain family member 2A	TBC1D2	1	12	12	18.9	105.41	27707000	27130000	27390000	28247000	28783000	22177000	0.963272	0.660377
Q96CS3	FAS-associated factor 2	FAF2	1	7	7	26.1	52.623	37003000	36277000	38422000	28657000	33885000	43097000	0.945722	0.660509
Q9Y6I3;Q9H201	Epsin-1	EPN1	2	5	5	13.7	60.293	20798000	27609000	30792000	25838000	24056000	25058000	0.946376	0.66082
Q9UH65	Switch-associated protein 70	SWAP70	1	12	12	30.9	68.997	35699000	24670000	28874000	26819000	29146000	40948000	1.085945	0.662136
Q9Y2X3	Nucleolar protein 58	NOP58	1	8	8	20.4	59.578	41884000	44907000	40957000	40822000	39456000	44690000	0.978238	0.662505
P24666	Low molecular weight phosphotyrosine prc	ACP1	1	4	4	41.1	18.042	32758000	30733000	39098000	46547000	33789000	30090000	1.076392	0.664341

P53396	ATP-citrate synthase	ACLY	1	40	40	46.5	120.84	2.15E+08	2.34E+08	1.97E+08	2.08E+08	2.11E+08	2.11E+08	0.976307	0.665327
P22033	Methylmalonyl-CoA mutase, mitochondrial	MUT	1	8	8	14.3	83.134	10188000	7402000	12754000	8194600	9992200	9852400	0.924044	0.665663
Q9H269	Vacuolar protein sorting-associated protein	VPS16	1	4	4	8.7	94.693	12937000	NaN	23143000	15235000	16925000	16436000	0.897931	0.66581
O15067	Phosphoribosylformylglycinamidine syntha	PFAS	1	7	7	8.8	144.73	13324000	15081000	NaN	14446000	14864000	NaN	1.031861	0.666017
P46977	Dolichyl-diphosphooligosaccharide--prote	STT3A	1	8	8	14.3	80.529	80511000	44780000	42771000	58444000	46574000	44948000	0.892325	0.666208
Q9UBF2	Coatomer subunit gamma-2	COPG2	1	9	7	17.1	97.621	13364000	NaN	15706000	18426000	14543000	13620000	1.068433	0.666897
Q09028	Histone-binding protein RBBP4	RBBP4	1	8	5	35.8	47.655	39374000	48860000	61789000	45986000	47745000	70458000	1.094426	0.667854
Q9UP95	Solute carrier family 12 member 4	SLC12A4	1	7	7	8.4	120.65	28080000	13846000	29234000	21291000	24282000	18321000	0.897892	0.667887
Q9H3S7	Tyrosine-protein phosphatase non-recepto	PTPN23	1	11	11	10.1	178.97	15487000	19082000	17215000	15985000	19214000	18571000	1.038352	0.667949
Q92696	Geranylgeranyl transferase type-2 subunit	RABGGTA	1	6	6	13.1	65.071	11215000	13316000	13719000	12908000	15563000	11691000	1.049987	0.668598
Q9NR28	Diablo homolog, mitochondrial	DIABLO	1	3	3	14.2	27.131	37630000	40726000	48638000	46126000	42314000	43309000	1.037443	0.671596
P25788	Proteasome subunit alpha type-3	PSMA3	1	7	7	30.2	28.433	70465000	78199000	55999000	59472000	69673000	65731000	0.95218	0.671951
P40855	Peroxisomal biogenesis factor 19	PEX19	1	6	6	32.4	32.806	26688000	22671000	26369000	23318000	24450000	25921000	0.973075	0.672597
Q13131;P54646	5-AMP-activated protein kinase catalytic su	PRKAA1	2	9	9	23.6	64.009	37009000	36725000	49716000	36830000	40376000	40209000	0.951114	0.673766
Q5TAQ9	DDB1- and CUL4-associated factor 8	DCAF8	1	5	5	12.4	66.851	24368000	21129000	23402000	23424000	24889000	22250000	1.024151	0.674552
Q9Y508	E3 ubiquitin-protein ligase RNF114	RNF114	1	3	3	19.7	25.694	54469000	54129000	56587000	44977000	56223000	76475000	1.075612	0.675889
Q92544	Transmembrane 9 superfamily member 4	TM9SF4	1	5	5	10.7	74.518	24777000	25246000	26783000	24240000	23501000	33454000	1.057144	0.676691
Q13011	Delta(3,5)-Delta(2,4)-dienoyl-CoA isomera	ECH1	1	7	7	29	35.816	86369000	81898000	92152000	89955000	83263000	82010000	0.980067	0.677088
P09661	U2 small nuclear ribonucleoprotein A	SNRPA1	1	2	2	12.2	28.415	17167000	17858000	16712000	14574000	19138000	20459000	1.047046	0.677775
Q96TC7	Regulator of microtubule dynamics protein	RMDN3	1	6	6	20	52.118	42378000	40065000	40783000	40818000	42462000	41085000	1.009243	0.67908
Q8NFQ8	Torsin-1A-interacting protein 2	TOR1AIP2	1	9	8	28.5	51.263	38917000	67637000	39132000	43950000	39983000	48598000	0.909703	0.679439
Q9NPR9	Protein GPR108	GPR108	1	2	2	3.7	60.632	12454000	14253000	12494000	11194000	14626000	11761000	0.958675	0.680088
Q9H0D6	5-3 exoribonuclease 2	XRN2	1	6	6	8.4	108.58	11853000	14424000	15992000	12360000	10866000	16342000	0.9361	0.680523
Q8NEU8	DCC-interacting protein 13-beta	APPL2	1	7	7	16.4	74.493	23953000	33386000	24401000	23210000	22481000	30681000	0.934328	0.680588
P39656	Dolichyl-diphosphooligosaccharide--prote	DDOST	1	9	9	26.1	50.8	1.8E+08	2.55E+08	1.94E+08	2.05E+08	1.96E+08	1.98E+08	0.951254	0.681735
O95140	Mitofusin-2	MFN2	1	3	3	6.5	86.401	9895300	9093200	11456000	9849700	9056700	10475000	0.965081	0.682892
Q9ULC3	Ras-related protein Rab-23	RAB23	1	4	4	26.2	26.659	25897000	32088000	31184000	30259000	29886000	31669000	1.029663	0.682933
Q9BRX2	Protein pelota homolog	PELO	1	3	3	9.9	43.359	15519000	11939000	NaN	12404000	16158000	15298000	1.064899	0.683552
Q6DKJ4	Nucleoredoxin	NXN	1	9	9	31.7	48.392	31451000	29609000	31026000	31931000	32168000	29370000	1.015019	0.684622
O14880	Microsomal glutathione S-transferase 3	MGST3	1	2	2	23.7	16.516	2.58E+08	2.52E+08	2.42E+08	1.77E+08	2.26E+08	3.02E+08	0.936714	0.686836
P62917	60S ribosomal protein L8	RPL8	1	7	7	36.2	28.024	3.15E+08	2.92E+08	2.64E+08	2.72E+08	2.93E+08	3.39E+08	1.037021	0.686887
P53007	Tricarboxylate transport protein, mitochon	SLC25A1	1	4	4	13.5	34.012	89829000	NaN	65844000	49433000	1.14E+08	1.08E+08	1.159109	0.687144
Q9NUQ9	Protein FAM49B	FAM49B	1	3	3	13	36.748	19110000	NaN	21803000	19142000	21496000	23125000	1.039001	0.687384
P60174	Triosephosphate isomerase	TPI1	1	14	14	75.9	26.669	5.87E+08	6.68E+08	6.62E+08	6.38E+08	6.09E+08	6.35E+08	0.981474	0.688687
Q9Y5A9	YTH domain-containing family protein 2	YTHDF2	1	5	4	10.2	62.333	73279000	53992000	72579000	50551000	70103000	67857000	0.943262	0.690793
Q9UN86	Ras GTPase-activating protein-binding pro	G3BP2	1	7	6	23.9	54.12	81100000	78349000	71497000	78360000	90912000	70195000	1.036896	0.692024
P25398	40S ribosomal protein S12	RPS12	1	5	5	54.5	14.515	1.1E+08	1.2E+08	1.43E+08	1.21E+08	1.32E+08	1.33E+08	1.035872	0.693303
Q8N1F7	Nuclear pore complex protein Nup93	NUP93	1	9	9	14.2	93.487	18832000	15215000	21968000	15675000	19220000	NaN	0.934437	0.695953
Q13557	Calcium/calmodulin-dependent protein kin	CAMK2D	1	9	6	34.1	56.369	1.53E+08	1.61E+08	1.57E+08	1.41E+08	1.76E+08	1.69E+08	1.029392	0.696265
Q9NZ45	CDGSH iron-sulfur domain-containing pro	CISD1	1	3	3	35.2	12.199	28977000	17027000	30068000	25943000	27669000	27756000	1.069618	0.697097
P04632	Calpain small subunit 1	CAPNS1	1	5	5	31.7	28.315	2.57E+08	3.13E+08	2.65E+08	2.32E+08	2.51E+08	3.14E+08	0.953977	0.697229
P40222	Alpha-taxilin	TXLNA	1	12	12	29.1	61.89	43736000	42725000	36468000	38744000	40872000	47757000	1.036151	0.697474
Q96F85	CB1 cannabinoid receptor-interacting prot	CNRIP1	1	2	2	23.2	18.648	9681900	NaN	12316000	9523900	12324000	13461000	1.070069	0.697613
Q8IYI6	Exocyst complex component 8	EXOC8	1	2	2	3.7	81.798	20416000	16673000	21859000	NaN	19757000	21307000	1.044921	0.699497
P40227;Q92526	T-complex protein 1 subunit zeta	CCT6A	2	20	20	45.8	58.024	2.94E+08	2.53E+08	2.75E+08	2.78E+08	3.05E+08	2.6E+08	1.026278	0.701165
P27695	DNA-(apurinic or apyrimidinic site) lyase;DI	APEX1	1	11	11	42.1	35.554	90991000	86291000	75176000	80269000	84918000	81212000	0.976	0.701279
P00441	Superoxide dismutase [Cu-Zn]	SOD1	1	6	6	60.4	15.936	6.45E+08	6.46E+08	4.64E+08	5.48E+08	5.33E+08	5.95E+08	0.955343	0.701682
P08758	Annexin A5	ANXA5	1	23	23	72.2	35.936	2.6E+09	3.19E+09	2.59E+09	2.59E+09	2.76E+09	2.78E+09	0.969513	0.702673
Q15075	Early endosome antigen 1	EEA1	1	53	53	44.4	162.46	77379000	74360000	80993000	80094000	78579000	76704000	1.011365	0.703211
Q7Z4V5;O75475	Hepatoma-derived growth factor-related p	HDGFRP2	2	4	3	7	74.316	27967000	34618000	26995000	27861000	24800000	32835000	0.954409	0.705245
P10515	Dihydrolipoyllysine-residue acetyltransfera	DLAT	1	5	5	10.5	68.996	53829000	68597000	54879000	46978000	53944000	67187000	0.948135	0.707333
Q7L014	Probable ATP-dependent RNA helicase DD	DDX46	1	7	7	8.7	117.36	14585000	16440000	17591000	16182000	23375000	12929000	1.079603	0.708156
P52209	6-phosphogluconate dehydrogenase, deca	PGD	1	16	16	43.5	53.139	1.61E+08	1.35E+08	1.8E+08	1.6E+08	1.55E+08	1.44E+08	0.964919	0.711967
Q15056	Eukaryotic translation initiation factor 4H	EIF4H	1	5	5	28.6	27.385	1.33E+08	1.28E+08	1.22E+08	1.37E+08	1.22E+08	1.16E+08	0.978235	0.713379
Q92896	Golgi apparatus protein 1	GLG1	1	21	21	22.1	134.55	55696000	50663000	57095000	52263000	56962000	51096000	0.980833	0.713736
O95861	3(2),5-bisphosphate nucleotidase 1	BPNT1	1	9	9	36.4	33.392	58442000	62050000	52974000	61974000	64645000	52267000	1.031245	0.714175
O60271;Q9UPT6	C-Jun-amino-terminal kinase-interacting p	SPAG9	2	9	9	9.4	146.2	12177000	17749000	22333000	15377000	18028000	23261000	1.08433	0.714513
P49137	MAP kinase-activated protein kinase 2	MAPKAPK2	1	4	4	9.2	45.567	15039000	10202000	14999000	12219000	12927000	NaN	0.937351	0.714522
P28070	Proteasome subunit beta type-4	PSMB4	1	4	4	25	29.204	1.8E+08	1.82E+08	1.52E+08	1.48E+08	1.7E+08	1.81E+08	0.96865	0.714889

Q9UIJ7	GTP:AMP phosphotransferase AK3, mitocho	AK3	1	9	9	44.1	25.565	1.6E+08	1.54E+08	1.88E+08	1.68E+08	1.69E+08	1.51E+08	0.9722	0.715886
P22830	Ferrochelatase, mitochondrial	FECH	1	7	7	21.3	47.862	15579000	17088000	19981000	15906000	16298000	18640000	0.965735	0.717479
P17568	NADH dehydrogenase [ubiquinone] 1 beta	NDUFB7	1	2	2	13.1	16.402	24331000	28629000	23219000	NaN	23141000	30360000	1.05346	0.718829
Q9NQC3	Reticulon-4	RTN4	1	13	13	20.6	129.93	5.07E+08	5.22E+08	5.29E+08	4.9E+08	5.2E+08	5.8E+08	1.020339	0.719282
O14908;Q8TF64	PDZ domain-containing protein GIPC1	GIPC1	2	7	7	22.5	36.049	47075000	52400000	49081000	42901000	46728000	54574000	0.970698	0.719929
O15121	Sphingolipid delta(4)-desaturase DES1	DEGS1	1	2	2	5.6	37.866	15676000	NaN	14750000	16496000	15063000	12040000	0.955301	0.720583
P54725	UV excision repair protein RAD23 homolog	RAD23A	1	5	3	11.6	39.609	48689000	45290000	50757000	42129000	51341000	47683000	0.975245	0.721031
P60866	40S ribosomal protein S20	RPS20	1	3	3	22.7	13.373	2.52E+08	2.44E+08	2.69E+08	2.55E+08	2.81E+08	2.44E+08	1.019934	0.721131
P16435	NADPH--cytochrome P450 reductase	POR	1	7	7	15.2	76.689	22442000	20369000	25081000	23951000	25979000	20395000	1.035836	0.722328
Q9Y676	28S ribosomal protein S18b, mitochondrial	MRPS18B	1	4	4	25.6	29.395	29499000	NaN	33076000	23941000	37730000	25403000	0.927676	0.722521
P27361;P31152;Q16659	Mitogen-activated protein kinase 3	MAPK3	3	8	5	31.4	43.135	58219000	46234000	59201000	53367000	57327000	47117000	0.964297	0.72295
Q9HAT2	Sialate O-acetylesterase	SIAE	1	3	3	7.1	58.314	26616000	32064000	NaN	30597000	34359000	27033000	1.045092	0.72337
Q8WWM7	Ataxin-2-like protein	ATXN2L	1	7	7	12.2	113.37	17566000	18536000	22865000	15988000	21409000	18999000	0.956399	0.723928
P54709	Sodium/potassium-transporting ATPase su	ATP1B3	1	5	5	25.4	31.512	40105000	59652000	47696000	47752000	42716000	50062000	0.953049	0.723945
Q5BJH7	Protein YIF1B	YIF1B	1	3	3	16.6	34.435	15581000	11598000	15815000	16178000	13741000	14823000	1.040657	0.724266
Q07954	Prolow-density lipoprotein receptor-relate	LRP1	1	46	46	14	504.6	42334000	45586000	42110000	42254000	45018000	44341000	1.012174	0.724952
P39023;Q92901	60S ribosomal protein L3	RPL3	2	12	12	39.7	46.108	1.47E+08	1.54E+08	1.28E+08	1.36E+08	1.36E+08	1.47E+08	0.977753	0.72573
Q9P2R7	Succinyl-CoA ligase [ADP-forming] subunit	SUCLA2	1	6	6	20.1	50.317	43202000	48114000	32700000	39204000	42767000	36585000	0.955973	0.728443
P53367	Arfaptin-1	ARFIP1	1	8	8	30.3	41.738	84657000	58945000	68612000	67592000	68934000	67301000	0.960479	0.728754
Q9P0K7	Ankycorbin	RAI14	1	24	24	31	110.04	1.39E+08	1.54E+08	1.3E+08	1.38E+08	1.48E+08	1.45E+08	1.019997	0.729118
Q9NVG8	TBC1 domain family member 13	TBC1D13	1	7	7	23.2	46.553	11877000	14808000	12998000	11972000	13859000	15282000	1.036036	0.729243
Q13148	TAR DNA-binding protein 43	TARDBP	1	3	3	5.1	44.739	80803000	56922000	28465000	47450000	48059000	53694000	0.897786	0.729377
Q13620	Cullin-4B	CUL4B	1	7	2	8.1	103.98	31588000	23742000	32434000	27170000	28447000	29009000	0.964245	0.729515
Q9UHX1	Poly(U)-binding-splicing factor PUF60	PUF60	1	6	6	16.6	59.875	38832000	48577000	34545000	32861000	38868000	44354000	0.951859	0.731385
Q9Y5B9	FACT complex subunit SPT16	SUPT16H	1	9	9	11.4	119.91	34033000	25746000	25262000	24479000	29262000	27868000	0.959643	0.737308
Q00653	Nuclear factor NF-kappa-B p100 subunit;N	NFKB2	1	4	4	5.8	96.748	16330000	16444000	19381000	18176000	15373000	NaN	0.964884	0.737344
Q9BZV1	UBX domain-containing protein 6	UBXN6	1	2	2	8.4	49.753	13354000	NaN	11536000	13406000	NaN	9996600	0.940241	0.737352
Q9UBI6	Guanine nucleotide-binding protein G(I)/G	GNG12	1	4	4	59.7	8.0061	1.32E+08	1.68E+08	1.05E+08	1.31E+08	1.24E+08	1.3E+08	0.95094	0.737777
O43390	Heterogeneous nuclear ribonucleoprotein I	HNRNPR	1	15	12	30	70.942	70922000	65805000	65236000	68800000	72684000	63845000	1.016666	0.738295
P30084	Enoyl-CoA hydratase, mitochondrial	ECHS1	1	6	6	30.3	31.387	1.12E+08	95395000	1.32E+08	1.29E+08	1.15E+08	73993000	0.937924	0.739437
P35611;P35612	Alpha-adducin	ADD1	2	8	8	12.9	80.954	51131000	51029000	34970000	56711000	45169000	42622000	1.053759	0.739773
P40925	Malate dehydrogenase, cytoplasmic	MDH1	1	11	11	42.5	36.426	2.95E+08	2.47E+08	3.5E+08	2.91E+08	3.47E+08	2.91E+08	1.041627	0.740243
P54886	Delta-1-pyrroline-5-carboxylate synthase;(	ALDH18A1	1	18	18	27.5	87.301	43590000	59278000	31881000	55658000	48077000	40591000	1.071073	0.742091
E9PAV3;Q13765;Q9BZK3	Nascent polypeptide-associated complex s	NACA	3	5	5	3.4	205.42	1.97E+08	1.66E+08	1.95E+08	1.88E+08	2.08E+08	1.76E+08	1.025793	0.742533
Q9Y3A5	Ribosome maturation protein SBDS	SBDS	1	8	8	41.2	28.763	57310000	54850000	58405000	46879000	62909000	55796000	0.970797	0.744557
Q53H12	Acylglycerol kinase, mitochondrial	AGK	1	2	2	6.9	47.137	NaN	15585000	18183000	16725000	18149000	NaN	1.032753	0.744771
P05121	Plasminogen activator inhibitor 1	SERPINE1	1	7	7	25.4	45.059	37343000	23120000	20764000	27125000	29631000	17918000	0.919325	0.745685
O95573	Long-chain-fatty-acid--CoA ligase 3	ACSL3	1	13	12	26.1	80.419	44084000	36478000	40028000	41936000	38595000	42694000	1.021851	0.7462
Q14011	Cold-inducible RNA-binding protein	CIRBP	1	3	3	20.9	18.648	52223000	35525000	41394000	45402000	35537000	NaN	0.940116	0.747552
O00534	von Willebrand factor A domain-containing	VWA5A	1	9	9	17	86.488	31749000	22041000	35417000	24784000	30685000	29220000	0.949354	0.747567
P13798	Acylamino-acid-releasing enzyme	APEH	1	8	8	18.3	81.224	58889000	64555000	63321000	57399000	68567000	56398000	0.976436	0.748069
O14907	Tax1-binding protein 3	TAX1BP3	1	3	3	39.5	13.735	66747000	90723000	78549000	57229000	75289000	91143000	0.94764	0.748581
P52948	Nuclear pore complex protein Nup98-Nup	NUP98	1	5	5	4.2	197.58	12146000	15349000	8733800	13468000	8527400	NaN	0.910687	0.749141
Q9UBM7	7-dehydrocholesterol reductase	DHCR7	1	3	3	9.1	54.489	15382000	25040000	NaN	20980000	16285000	19165000	0.930681	0.749812
P42704	Leucine-rich PPR motif-containing protein,	LRPPRC	1	27	27	24.4	157.9	4.69E+08	4.98E+08	4.63E+08	4.32E+08	4.93E+08	4.83E+08	0.984596	0.752246
Q01628;P13164;Q01629;C9JQ	Interferon-induced transmembrane protein	IFITM3	4	3	3	48.9	14.632	75845000	36795000	58938000	68794000	41830000	46763000	0.917291	0.752769
P28288	ATP-binding cassette sub-family D membe	ABCD3	1	6	6	11.7	75.475	30557000	33352000	35721000	30231000	27982000	38030000	0.966004	0.755874
O60831	PRA1 family protein 2	PRAF2	1	3	3	22.5	19.258	66651000	89659000	29493000	38970000	45232000	79975000	0.883608	0.756168
Q6YN16	Hydroxysteroid dehydrogenase-like protein	HSDL2	1	8	8	26.1	45.394	68776000	79777000	88181000	74783000	83720000	84651000	1.027119	0.756316
P63000;P60763	Ras-related C3 botulinum toxin substrate 1	RAC1;RAC3	2	8	3	49.5	21.45	2.75E+08	3.26E+08	2.77E+08	2.97E+08	2.85E+08	2.78E+08	0.979833	0.756838
Q13555;Q13554;Q9UQM7	Calcium/calmodulin-dependent protein kin	CAMK2G;CAMK2B	3	5	2	15.6	62.606	11528000	16565000	NaN	14222000	13348000	16935000	1.056135	0.758167
Q9UKV3	Apoptotic chromatin condensation inducer	ACIN1	1	6	6	8.7	151.86	15761000	10952000	13676000	14914000	13625000	13310000	1.036148	0.758194
Q03252	Lamin-B2	LMNB2	1	18	16	29.2	69.948	92303000	65914000	87263000	89339000	85484000	58136000	0.948994	0.759342
P52306	Rap1 GTPase-GDP dissociation stimulator 1	RAP1GDS1	1	11	11	23.9	66.316	56573000	45598000	49013000	48920000	56337000	49852000	1.025962	0.759687
Q9NZZ3	Charged multivesicular body protein 5	CHMP5	1	3	3	25.1	24.57	34143000	32464000	26661000	24404000	30059000	35055000	0.959793	0.759991
Q9H0A8	COMM domain-containing protein 4	COMMD4	1	3	3	25.6	21.764	22082000	NaN	24142000	NaN	29053000	20304000	1.067779	0.760688
Q5JTJ3	Cytochrome c oxidase assembly factor 6 hc	COA6	1	3	3	22.4	14.116	16100000	17171000	18555000	18582000	21088000	14230000	1.040019	0.76132
P67812	Signal peptidase complex catalytic subunit	SEC11A	1	4	4	17.9	20.625	35764000	40426000	41357000	29746000	42125000	52236000	1.055807	0.761514
Q96FW1	Ubiquitin thioesterase OTUB1	OTUB1	1	8	8	50.9	31.284	81922000	63579000	76439000	67179000	79942000	81723000	1.031108	0.762346

O00231	26S proteasome non-ATPase regulatory su	PSMD11	1	18	18	52.6	47.463	98406000	88436000	99145000	93315000	99459000	88732000	0.984331	0.763909
O43707	Alpha-actinin-4	ACTN4	1	59	41	72.4	104.85	1.68E+09	1.77E+09	1.61E+09	1.68E+09	1.64E+09	1.69E+09	0.990839	0.765243
P08708	40S ribosomal protein S17	RPS17	1	6	6	33.3	15.55	1.38E+08	1.01E+08	1.29E+08	1.26E+08	1.35E+08	1.2E+08	1.031482	0.766146
Q9NP79	Vacuolar protein sorting-associated protei	VTA1	1	5	5	25.1	33.879	24746000	29674000	26537000	22356000	28196000	28105000	0.97159	0.76626
Q13162	Peroxiredoxin-4	PRDX4	1	11	9	46.5	30.54	1.27E+08	80750000	1.3E+08	1.16E+08	1.15E+08	1.22E+08	1.045456	0.76679
O00161	Synaptosomal-associated protein 23	SNAP23	1	7	7	40.3	23.354	25686000	30270000	26068000	24661000	24987000	30184000	0.973276	0.768034
P23919	Thymidylate kinase	DTYMK	1	6	6	33.5	23.819	25653000	36361000	35630000	32148000	31344000	37970000	1.039101	0.768268
O00743	Serine/threonine-protein phosphatase 6 ca	PPP6C	1	5	5	22.6	35.144	22872000	18087000	NaN	22642000	20136000	NaN	1.04441	0.768353
Q9BXK5	Bcl-2-like protein 13	BCL2L13	1	6	6	22.7	52.723	35663000	32284000	33568000	29339000	34217000	41370000	1.033601	0.769753
Q9P032	NADH dehydrogenase [ubiquinone] 1 alph	NDUFAF4	1	3	3	17.1	20.266	9347300	NaN	10894000	13489000	9265800	9408300	1.059323	0.770188
P10301	Ras-related protein R-Ras	RRAS	1	8	6	45.9	23.48	47697000	37955000	52769000	48526000	41548000	43853000	0.967534	0.770889
P49585	Choline-phosphate cytidylyltransferase A	PCYT1A	1	4	4	14.2	41.731	63940000	81030000	63208000	54744000	74032000	71561000	0.962335	0.771322
P62750	60S ribosomal protein L23a	RPL23A	1	9	9	43.6	17.695	3.12E+08	2.13E+08	3.45E+08	3.63E+08	2.58E+08	2.95E+08	1.053436	0.773608
Q99714	3-hydroxyacyl-CoA dehydrogenase type-2	HSD17B10	1	8	8	42.9	26.923	46890000	40554000	33715000	35308000	38261000	43454000	0.965863	0.774023
Q13951	Core-binding factor subunit beta	CBFB	1	4	4	26.4	21.508	14207000	11215000	18319000	18366000	14631000	13120000	1.05432	0.774419
Q9Y266	Nuclear migration protein nudC	NUDC	1	12	12	42	38.242	1.13E+08	98192000	1.01E+08	1.03E+08	1.1E+08	94747000	0.981933	0.774958
Q15417	Calponin-3	CNN3	1	11	10	43.2	36.413	2.81E+08	2.82E+08	2.64E+08	3.09E+08	2.39E+08	2.6E+08	0.976527	0.776748
Q14061	Cytochrome c oxidase copper chaperone	COX17	1	2	2	46	6.9151	25665000	21769000	NaN	19711000	26585000	28257000	1.047814	0.777826
Q07666	KH domain-containing, RNA-binding, signa	KHDRBS1	1	6	6	22.1	48.227	61627000	54485000	60338000	62967000	59097000	50513000	0.97805	0.778272
Q02880;P11388	DNA topoisomerase 2-beta	TOP2B	2	5	5	3.7	183.26	13162000	NaN	19069000	16782000	17684000	15975000	1.043323	0.780552
O94905	Erlin-2	ERLIN2	1	11	9	36.6	37.839	1.03E+08	87595000	82157000	76892000	82958000	1.04E+08	0.967394	0.785283
P26641	Elongation factor 1-gamma	EEF1G	1	15	15	37.1	50.118	2.26E+08	2.41E+08	2.74E+08	2.41E+08	2.54E+08	2.34E+08	0.981946	0.78533
Q14318	Peptidyl-prolyl cis-trans isomerase FKBP8	FKBP8	1	7	7	23.8	44.561	20793000	27941000	22790000	23009000	24409000	26123000	1.0282	0.785651
O76021	Ribosomal L1 domain-containing protein 1	RSL1D1	1	9	9	20.8	54.972	47125000	61477000	53883000	49627000	56525000	60945000	1.028384	0.785962
O75352	Mannose-P-dolichol utilization defect 1 pro	MPDU1	1	2	2	8.1	26.638	42215000	45361000	NaN	48959000	41793000	43311000	1.020546	0.787184
O43324	Eukaryotic translation elongation factor 1 e	EEF1E1	1	4	4	23	19.81	33534000	31034000	35967000	36076000	33502000	28750000	0.978047	0.789321
Q9BTE1	Dynactin subunit 5	DCTN5	1	3	3	15.4	20.126	25245000	16760000	NaN	18757000	NaN	20603000	0.937031	0.789443
P15880	40S ribosomal protein S2	RPS2	1	8	8	34.1	31.324	2.42E+08	1.97E+08	2.12E+08	2.34E+08	2.39E+08	1.94E+08	1.025642	0.790162
Q15369	Transcription elongation factor B polypepti	TCEB1	1	6	6	84.8	12.473	1.87E+08	1.62E+08	1.53E+08	1.6E+08	1.92E+08	1.61E+08	1.024637	0.790836
O43684	Mitotic checkpoint protein BUB3	BUB3	1	7	7	32.9	37.154	36132000	21518000	34769000	30830000	31419000	25946000	0.954295	0.79113
P62633	Cellular nucleic acid-binding protein	CNBP	1	5	5	35	19.463	67567000	61316000	50676000	57195000	54319000	63293000	0.973535	0.791159
P48643	T-complex protein 1 subunit epsilon	CCT5	1	23	23	59.5	59.67	2.42E+08	2.43E+08	2.32E+08	2.36E+08	2.34E+08	2.51E+08	1.007766	0.791322
Q9UKD2	mRNA turnover protein 4 homolog	MRTO4	1	4	4	21.8	27.56	11323000	13306000	NaN	10823000	11427000	16974000	1.061729	0.791597
Q9BWD1	Acetyl-CoA acetyltransferase, cytosolic	ACAT2	1	9	9	35.3	41.35	67008000	59302000	68780000	82861000	70581000	49986000	1.042739	0.795282
P53041	Serine/threonine-protein phosphatase 5	PPP5C	1	3	3	8.6	56.878	24143000	23798000	26692000	23731000	26467000	25431000	1.013345	0.797703
Q9UNS2	COP9 signalosome complex subunit 3	COPS3	1	8	8	27.4	47.873	68278000	86723000	66624000	69993000	71939000	86442000	1.030452	0.799108
Q13177;Q13153;O75914	Serine/threonine-protein kinase PAK 2;PAK	PAK2	3	19	19	56.7	58.042	50189000	39336000	46391000	44764000	47396000	46412000	1.019541	0.800047
A6NDG6	Phosphoglycolate phosphatase	PGP	1	4	4	18.1	34.006	18796000	18862000	14273000	15475000	18842000	19175000	1.030059	0.800238
P35580	Myosin-10	MYH10	1	41	25	25.5	229	38716000	44806000	41585000	37828000	44537000	45112000	1.018944	0.800514
P49023	Paxillin	PXN	1	14	14	39.6	64.505	44214000	50688000	48773000	41670000	48685000	50624000	0.981235	0.800567
Q15907;P62491	Ras-related protein Rab-11B;Ras-related p	RAB11B;RAB11A	2	10	10	49.5	24.488	2.19E+08	2.28E+08	2.19E+08	2.41E+08	2.16E+08	2.16E+08	1.010837	0.801671
Q92621	Nuclear pore complex protein Nup205	NUP205	1	4	4	2.4	227.92	8164200	NaN	8941700	NaN	7222700	10981000	1.064177	0.801751
P06703	Protein S100-A6	S100A6	1	7	7	64.4	10.18	7.23E+09	7.76E+09	8.16E+09	5.67E+09	7.02E+09	9.53E+09	0.959782	0.80271
Q14694	Ubiquitin carboxyl-terminal hydrolase 10	USP10	1	4	4	7.4	87.133	15333000	15634000	15330000	14904000	12841000	20326000	1.038318	0.804353
Q8WZA0	Protein LZIC	LZIC	1	4	4	32.1	21.494	35500000	48107000	23126000	26338000	25466000	67269000	1.115616	0.804606
Q9NYL9	Tropomodulin-3	TMOD3	1	8	7	38.6	39.594	92633000	92847000	88356000	94156000	88001000	93622000	1.007095	0.804769
Q13442	28 kDa heat- and acid-stable phosphoprot	PDAP1	1	5	5	22.7	20.63	62250000	50323000	69800000	66846000	61985000	58423000	1.026764	0.80513
Q15637	Splicing factor 1	SF1	1	6	6	14.9	68.329	19885000	19992000	19941000	21937000	NaN	18609000	1.016734	0.805448
Q9ULT8	E3 ubiquitin-protein ligase HECTD1	HECTD1	1	7	7	4	289.37	17095000	13088000	15198000	14548000	14902000	NaN	0.973425	0.80605
Q9BT09	Protein canopy homolog 3	CNPY3	1	3	3	14.4	30.748	69178000	57726000	85567000	NaN	71773000	64100000	0.959234	0.806797
P62328	Thymosin beta-4;Hematopoietic system reg	TMSB4X	1	6	6	77.3	5.0526	6.44E+08	5.46E+08	6.86E+08	6.34E+08	7.95E+08	5.17E+08	1.037624	0.807929
O00170	AH receptor-interacting protein	AIP	1	10	10	42.4	37.664	21873000	27299000	32651000	28189000	25727000	25410000	0.969483	0.809531
Q9H307	Pinin	PNN	1	3	3	6.1	81.627	NaN	11578000	13900000	20447000	8392700	NaN	1.131945	0.809893
Q4L180	Filamin A-interacting protein 1-like	FILIP1L	1	10	10	12.3	130.38	22048000	16098000	NaN	16901000	20975000	17149000	0.961656	0.810556
Q86XP3	ATP-dependent RNA helicase DDX42	DDX42	1	6	6	10.6	102.97	26258000	27960000	21722000	23534000	28503000	25690000	1.023532	0.812609
Q92575	UBX domain-containing protein 4	UBXN4	1	2	2	10.8	56.777	10168000	17447000	13665000	13095000	11736000	18692000	1.054336	0.814948
Q9Y285	Phenylalanine--tRNA ligase alpha subunit	FARSA	1	12	12	34.8	57.563	75790000	60296000	53809000	63111000	65541000	66175000	1.025972	0.815204
Q8N6T3	ADP-ribosylation factor GTPase-activating	ARFGAP1	1	8	8	33.3	44.667	32636000	26323000	25392000	24468000	27545000	30229000	0.974997	0.81536
Q9Y6M9	NADH dehydrogenase [ubiquinone] 1 beta	NDUFB9	1	5	5	36.3	21.831	26635000	30011000	33752000	25998000	28718000	33450000	0.975309	0.815994

P62937;P0DN26;A0A0B4J2A2;A	Peptidyl-prolyl cis-trans isomerase A;Peptic	PPIA	8	12	12	55.2	18.012	1.08E+09	1.03E+09	1.08E+09	1.17E+09	1.16E+09	9.21E+08	1.019672	0.816153
P00491	Purine nucleoside phosphorylase	PNP	1	4	4	21.1	32.118	29333000	35270000	25569000	28326000	31181000	32996000	1.025851	0.816391
Q9UK41	Vacuolar protein sorting-associated protein	VPS28	1	5	5	33.9	25.425	23664000	18788000	26668000	24497000	24536000	21907000	1.026331	0.817008
Q9Y2S7	Polymerase delta-interacting protein 2	POLDIP2	1	3	3	8.7	42.033	NaN	17146000	13149000	20213000	16603000	11373000	1.060439	0.81729
O60888	Protein CutA	CUTA	1	4	4	23.5	19.116	1.22E+08	1.33E+08	1.08E+08	75554000	1.51E+08	1.57E+08	1.05501	0.818179
Q96K76	Ubiquitin carboxyl-terminal hydrolase 47	USP47	1	3	3	3.5	157.31	8617400	8918200	10308000	9839200	8625300	8915600	0.983353	0.820134
Q5SW79;Q96L14	Centrosomal protein of 170 kDa	CEP170	2	25	25	23.3	175.29	51232000	49380000	55136000	49972000	51064000	53288000	0.990857	0.820284
O95831	Apoptosis-inducing factor 1, mitochondrial	AIFM1	1	9	9	20.1	66.9	80538000	80819000	83477000	71153000	72699000	95318000	0.976866	0.822114
O75439	Mitochondrial-processing peptidase subun	PMPCB	1	5	5	12.1	54.366	30218000	31593000	31516000	34687000	35465000	25500000	1.024912	0.822217
O15400	Syntaxin-7	STX7	1	4	4	22.2	29.815	35351000	NaN	31170000	26224000	40682000	37111000	1.042448	0.824134
P47813;O14602	Eukaryotic translation initiation factor 1A, X	EIF1AX;EIF1AY	2	6	6	34	16.46	52702000	36737000	62679000	56106000	50545000	50988000	1.036294	0.82425
P11142	Heat shock cognate 71 kDa protein	HSPA8	1	38	27	76.3	70.897	1.27E+09	1.43E+09	1.37E+09	1.34E+09	1.32E+09	1.39E+09	0.991195	0.824912
O14964	Hepatocyte growth factor-regulated tyrosi	HGS	1	10	10	12.6	86.191	39721000	36937000	33951000	32477000	36855000	39434000	0.983338	0.826551
O75489	NADH dehydrogenase [ubiquinone] iron-s	NDUFS3	1	3	3	14	30.241	31850000	44148000	33845000	40799000	33584000	38493000	1.027612	0.828175
Q15750	TGF-beta-activated kinase 1 and MAP3K7-	TAB1	1	5	5	14.3	54.643	6E+08	NaN	1.27E+09	7.98E+08	8.96E+08	9.26E+08	0.935815	0.828363
P04083	Annexin A1	ANXA1	1	22	22	67.9	38.714	1.21E+09	1.12E+09	1.14E+09	1.17E+09	1.23E+09	1.1E+09	1.008754	0.828735
Q9UMX0	Ubiquilin-1	UBQLN1	1	6	2	14.6	62.518	37903000	30898000	36573000	38130000	38140000	31263000	1.020489	0.82997
Q52LJ0	Protein FAM98B	FAM98B	1	6	4	19.4	45.547	24712000	7687800	21381000	15186000	17501000	NaN	0.911673	0.830368
O14828	Secretory carrier-associated membrane pro	SCAMP3	1	8	8	38.3	38.287	69487000	76371000	59529000	67531000	69693000	71560000	1.01654	0.832775
Q9Y2T2	AP-3 complex subunit mu-1	AP3M1	1	4	3	14.8	46.939	21623000	29827000	21799000	23517000	26060000	25569000	1.025898	0.833314
O75534	Cold shock domain-containing protein E1	CSDE1	1	13	13	20.9	88.884	52531000	70441000	63335000	56825000	58363000	76651000	1.029693	0.833603
Q9BWM7	Sideroflexin-3	SFXN3	1	10	10	37.1	35.503	49418000	44608000	57835000	54403000	51002000	43018000	0.977361	0.834203
O00244	Copper transport protein ATOX1	ATOX1	1	3	3	39.7	7.4016	NaN	77577000	69153000	59204000	71024000	1.01E+08	1.050674	0.83495
Q16698	2,4-dienoyl-CoA reductase, mitochondrial	DECR1	1	7	7	31.6	36.067	58038000	66563000	64477000	58480000	62664000	65742000	0.988407	0.836504
Q96DV4	39S ribosomal protein L38, mitochondrial	MRPL38	1	5	5	16.8	44.596	8844000	NaN	12409000	8755800	10898000	11120000	0.965316	0.837289
Q9P2R3	Rabankyrin-5	ANKFY1	1	10	10	12	128.4	1.24E+08	1.26E+08	1.33E+08	95161000	1.49E+08	1.51E+08	1.031568	0.837555
Q96I24	Far upstream element-binding protein 3	FUBP3	1	12	11	30.6	61.64	26109000	25523000	26873000	23235000	31649000	21560000	0.973747	0.837822
Q15365;P57723	Poly(rC)-binding protein 1	PCBP1	2	13	10	63.8	37.497	2.78E+08	3.21E+08	2.81E+08	2.94E+08	2.89E+08	3.07E+08	1.010939	0.837826
P62873	Guanine nucleotide-binding protein G(I)/G	GNB1	1	7	4	30.3	37.377	57047000	64143000	71932000	62019000	66405000	67712000	1.015607	0.83885
Q07021	Complement component 1 Q subcompone	C1QBP	1	7	7	40.8	31.362	1.5E+08	1.61E+08	1.64E+08	1.5E+08	1.52E+08	1.68E+08	0.9903	0.838941
Q9Y6K5	2-5-oligoadenylate synthase 3	OAS3	1	13	13	15.5	121.17	44382000	37816000	46197000	43992000	37777000	44466000	0.983177	0.83965
O75828	Carbonyl reductase [NADPH] 3	CBR3	1	6	4	29.2	30.85	15554000	13395000	13257000	14231000	16727000	12217000	1.022959	0.840202
P20700	Lamin-B1	LMNB1	1	12	10	27.3	66.408	54596000	54231000	49328000	52313000	48447000	59780000	1.01508	0.841838
P61289	Proteasome activator complex subunit 3	PSME3	1	4	4	19.7	29.506	43065000	37706000	40697000	42081000	38827000	39397000	0.990425	0.843991
P30153;P30154	Serine/threonine-protein phosphatase 2A	PPP2R1A	2	19	19	43.3	65.308	3.39E+08	3.75E+08	3.29E+08	3.26E+08	3.49E+08	3.57E+08	0.989927	0.844267
Q15424	Scaffold attachment factor B1	SAFB	1	8	4	16	102.64	22497000	14205000	27027000	17055000	23315000	20739000	0.958888	0.844363
P84090	Enhancer of rudimentary homolog	ERH	1	4	4	45.2	12.259	1.63E+08	1.36E+08	1.57E+08	1.36E+08	1.5E+08	1.62E+08	0.984699	0.84477
P31947	14-3-3 protein sigma	SFN	1	4	1	13.3	27.774	1.41E+08	8.97E+08	3.54E+08	2.64E+08	4.72E+08	5.08E+08	0.893191	0.844873
Q2TAY7	WD40 repeat-containing protein SMU1;WD	SMU1	1	5	5	15.2	57.543	14665000	17264000	16873000	15574000	16962000	16841000	1.011782	0.845615
Q13561	Dynactin subunit 2	DCTN2	1	13	13	44.1	44.23	1.47E+08	1.7E+08	1.51E+08	1.43E+08	1.54E+08	1.65E+08	0.987785	0.847129
Q9UDW1	Cytochrome b-c1 complex subunit 9	UQCR10	1	1	1	27	7.3084	61305000	NaN	43018000	53960000	59827000	35311000	0.952797	0.847132
P53814	Smoothelin	SMTN	1	8	8	11.6	99.058	12097000	19037000	13028000	13169000	14692000	14926000	0.968865	0.848098
O15260	Surfeit locus protein 4	SURF4	1	4	4	18.6	30.394	94861000	1.07E+08	NaN	1.19E+08	92801000	97783000	1.023415	0.848797
P47224	Guanine nucleotide exchange factor MSS4	RABIF	1	3	3	38.2	13.839	16437000	15364000	11940000	12851000	13070000	16699000	0.974372	0.849206
Q6NYC8	Phostensin	PPP1R18	1	8	8	23	67.942	30428000	28936000	33451000	29609000	30239000	32047000	0.990088	0.849548
Q9UHY7	Enolase-phosphatase E1	ENOPH1	1	4	4	27.2	28.932	30142000	30922000	31843000	26979000	29952000	34602000	0.985211	0.850083
Q9NYF8	Bcl-2-associated transcription factor 1	BCLAF1	1	6	5	9.3	106.12	37541000	54772000	46599000	52167000	44421000	45649000	1.023936	0.850834
Q86TI2	Dipeptidyl peptidase 9	DPP9	1	9	9	16.5	98.262	27418000	35830000	34947000	31737000	31287000	37074000	1.01938	0.85487
Q15843	NEDD8	NEDD8	1	3	3	28.4	9.0714	1.36E+08	1.12E+08	1.7E+08	1.63E+08	1.41E+08	1E+08	0.965128	0.854972
Q9Y2Y0	ADP-ribosylation factor-like protein 2-binc	ARL2BP	1	3	3	27.6	18.822	31798000	22556000	25299000	27272000	24359000	NaN	0.972299	0.855166
P60228	Eukaryotic translation initiation factor 3 sub	EIF3E	1	10	10	30.6	52.22	49017000	43075000	50754000	49645000	53060000	37004000	0.978039	0.856116
P05091;P00352;P47895;O9478	Aldehyde dehydrogenase, mitochondrial	ALDH2	4	10	9	24	56.381	1.33E+08	1.59E+08	1.53E+08	1.39E+08	1.46E+08	1.67E+08	1.014618	0.858303
P15927	Replication protein A 32 kDa subunit	RPA2	1	2	2	12.6	29.247	12188000	10780000	11866000	11906000	13310000	8828600	0.977338	0.859096
P53990	IST1 homolog	IST1	1	4	4	12.6	39.75	54438000	79721000	NaN	84281000	60970000	63740000	1.038524	0.85913
Q15555	Microtubule-associated protein RP/EB fami	MAPRE2	1	4	3	20.2	37.031	40011000	43649000	34710000	38990000	40678000	37161000	0.986981	0.862735
Q9NRH3;P23258	Tubulin gamma-2 chain;Tubulin gamma-1	TUBG2;TUBG1	2	5	5	19.7	51.091	13178000	15224000	13186000	15240000	12779000	14110000	1.013009	0.863566
P14927	Cytochrome b-c1 complex subunit 7	UQCRB	1	3	3	34.2	13.53	56029000	47280000	59082000	47871000	56851000	55211000	0.984864	0.863985
Q9H3U1	Protein unc-45 homolog A	UNC45A	1	12	12	17.7	103.08	22710000	22034000	32057000	24006000	21735000	28965000	0.972722	0.865752
P11172	Uridine 5-monophosphate synthase;Orotat	UMPS	1	4	4	13.3	52.221	13402000	15350000	NaN	15227000	18129000	11245000	1.034154	0.866138

Q9BXS5;Q9Y6Q5	AP-1 complex subunit mu-1	AP1M1	2	9	9	33.3	48.586	32040000	33301000	32868000	32905000	29365000	35036000	0.990805	0.867659
P35241	Radixin	RDX	1	21	9	34.8	68.563	89808000	97702000	84904000	85567000	87672000	1.03E+08	1.012756	0.868098
Q9Y6M5	Zinc transporter 1	SLC30A1	1	5	5	16.8	55.299	38873000	41185000	40965000	46494000	38369000	34208000	0.983871	0.868323
Q02818	Nucleobindin-1	NUCB1	1	10	10	30.6	53.879	35544000	33101000	40799000	29457000	38828000	39113000	0.981306	0.869721
Q9H173	Nucleotide exchange factor SIL1	SIL1	1	3	3	13.2	52.084	12262000	13587000	6170700	10898000	9776600	10144000	0.962489	0.870666
Q9UM54	Unconventional myosin-VI	MYO6	1	18	18	20.5	149.69	31778000	30950000	34201000	27201000	33680000	34728000	0.986382	0.871283
P53680	AP-2 complex subunit sigma	AP2S1	1	5	5	29.6	17.018	90088000	70576000	90105000	93798000	99524000	64046000	1.026315	0.871677
Q9BRK5	45 kDa calcium-binding protein	SDF4	1	4	4	19.6	41.806	43923000	27148000	39797000	36375000	46015000	24336000	0.96264	0.872139
P17050	Alpha-N-acetylgalactosaminidase	NAGA	1	4	4	13.1	46.564	18373000	19882000	NaN	19218000	18797000	19709000	1.005951	0.873528
Q15126	Phosphomevalonate kinase	PMVK	1	5	5	30.2	21.995	24164000	23017000	28995000	24027000	23430000	30150000	1.018785	0.873999
P62256	Ubiquitin-conjugating enzyme E2 H	UBE2H	1	2	2	16.4	20.655	NaN	13887000	9208600	10129000	10589000	12885000	0.969968	0.877123
Q4G0F5	Vacuolar protein sorting-associated protei	VPS26B	1	4	4	14.9	39.154	17724000	24008000	35099000	23231000	25780000	NaN	0.95686	0.878175
P63208	S-phase kinase-associated protein 1	SKP1	1	5	5	44.8	18.658	1.02E+08	1.34E+08	99267000	1.18E+08	1.1E+08	1.13E+08	1.016748	0.878857
Q5JTH9	RRP12-like protein	RRP12	1	5	5	5.7	143.7	36276000	29031000	38382000	34348000	NaN	36004000	1.017736	0.879201
P61160	Actin-related protein 2	ACTR2	1	10	10	29.7	44.76	2.31E+08	1.95E+08	1.94E+08	2.32E+08	2.12E+08	1.65E+08	0.982163	0.881098
Q15691	Microtubule-associated protein RP/EB fami	MAPRE1	1	9	8	45.9	29.999	83894000	88675000	1.08E+08	87842000	92521000	96864000	0.986612	0.882243
P53999	Activated RNA polymerase II transcriptiona	SUB1	1	4	4	37	14.395	87246000	51578000	98201000	79769000	88000000	61729000	0.968244	0.883505
O15357	Phosphatidylinositol 3,4,5-trisphosphate 5-	INPPL1	1	5	5	5.5	138.6	11246000	NaN	11948000	10638000	NaN	12951000	1.01703	0.885213
P49755	Transmembrane emp24 domain-containing	TMED10	1	5	5	30.1	24.976	2.03E+08	2.46E+08	2.16E+08	1.74E+08	2.18E+08	2.59E+08	0.980976	0.885631
Q9UMY4	Sorting nexin-12	SNX12	1	3	2	19.1	18.884	25001000	27726000	26490000	25465000	24358000	28697000	0.991201	0.886027
Q96M27	Protein PRRC1	PRRC1	1	3	3	10.6	46.701	20543000	23740000	33343000	25129000	25041000	25694000	0.977301	0.886184
P52597	Heterogeneous nuclear ribonucleoprotein I	HNRNPF	1	9	7	30.6	45.671	42663000	45407000	37878000	36285000	43918000	47566000	1.014458	0.886327
P29353;P98077;Q6S5L8	SHC-transforming protein 1	SHC1	3	12	12	32.6	62.821	44182000	54173000	58494000	46831000	44715000	62137000	0.979815	0.88641
Q66K74	Microtubule-associated protein 1S;MAP1S	MAP1S	1	12	12	19.3	112.21	29512000	29773000	25547000	29052000	26537000	30016000	1.009112	0.887912
P51570	Galactokinase	GALK1	1	7	7	20.7	42.272	50499000	51951000	48267000	47349000	49425000	53053000	0.994095	0.888229
P62841	40S ribosomal protein S15	RPS15	1	4	4	33.1	17.04	1.14E+08	1.45E+08	97809000	1.03E+08	1.16E+08	1.31E+08	0.979742	0.888314
Q92530	Proteasome inhibitor PI31 subunit	PSMF1	1	4	4	20.7	29.816	38058000	65121000	50820000	49254000	46672000	62178000	1.026656	0.888614
Q8IYM9	E3 ubiquitin-protein ligase TRIM22	TRIM22	1	2	2	4.6	56.947	12633000	13073000	10786000	11721000	11912000	13237000	1.010358	0.888994
O43592	Exportin-T	XPOT	1	6	6	8.3	109.96	12636000	16323000	13544000	13122000	13082000	17050000	1.017669	0.891389
P83111	Serine beta-lactamase-like protein LACTB,	LACTB	1	5	5	15.2	60.693	21911000	26313000	27055000	23257000	19581000	30853000	0.978905	0.892777
Q13242	Serine/arginine-rich splicing factor 9	SRSF9	1	6	6	27.6	25.542	30903000	26077000	30283000	26924000	34431000	27148000	1.01421	0.893355
Q9H4M9	EH domain-containing protein 1	EHD1	1	23	16	55.8	60.626	1.45E+08	1.1E+08	1.48E+08	1.28E+08	1.34E+08	1.36E+08	0.98682	0.894276
Q13126	S-methyl-5-thioadenosine phosphorylase	MTAP	1	6	6	37.1	31.236	22261000	23907000	23440000	20908000	20735000	27059000	0.986984	0.894432
Q9UBW8	COP9 signalosome complex subunit 7a	COPS7A	1	5	5	23.6	30.276	26356000	17309000	18505000	15650000	24383000	NaN	0.965892	0.894448
Q15363	Transmembrane emp24 domain-containing	TMED2	1	4	4	30.8	22.761	1.47E+08	2.03E+08	1.52E+08	1.44E+08	1.81E+08	1.68E+08	0.982566	0.895689
Q9NZT2	Opioid growth factor receptor	OGFR	1	5	5	18.2	73.324	13999000	16040000	15773000	14895000	17241000	13110000	0.987645	0.896133
Q96T76	MMS19 nucleotide excision repair protein I	MMS19	1	6	6	10.2	113.29	15324000	13202000	12546000	12432000	NaN	15387000	1.015984	0.896279
P36969	Phospholipid hydroperoxide glutathione pe	GPX4	1	4	4	29.4	22.174	19647000	19618000	24385000	18653000	24494000	19506000	0.984336	0.897157
Q14677	Clathrin interactor 1	CLINT1	1	10	10	21.4	68.259	42107000	37352000	35884000	38095000	34358000	41719000	0.989848	0.89718
Q15717	ELAV-like protein 1	ELAVL1	1	6	6	27.6	36.091	29839000	35126000	31872000	29311000	29298000	37002000	0.98734	0.897969
P23786	Carnitine O-palmitoyltransferase 2, mitoch	CPT2	1	7	7	13.7	73.776	15728000	14419000	17468000	13460000	15666000	19262000	1.016234	0.899076
O00116	Alkyldihydroxyacetonephosphate synthase,	AGPS	1	11	11	28.9	72.911	41635000	48427000	49015000	44514000	44610000	48836000	0.991968	0.89934
O15127	Secretory carrier-associated membrane pr	SCAMP2	1	2	2	9.7	36.648	45682000	36697000	45211000	38239000	49619000	37785000	0.98474	0.899987
O95302;Q75LS8	Peptidyl-prolyl cis-trans isomerase FKBP9	FKBP9	2	6	6	11.4	63.083	96485000	1.01E+08	76603000	1.08E+08	82316000	87567000	1.015838	0.900243
P48637	Glutathione synthetase	GSS	1	12	12	32.9	52.384	80111000	97284000	98428000	92465000	92651000	93060000	1.008531	0.901108
Q09161	Nuclear cap-binding protein subunit 1	NCBP1	1	8	8	14.3	91.838	15204000	10634000	13667000	12752000	16644000	9099400	0.974444	0.901715
Q14166	Tubulin--tyrosine ligase-like protein 12	TTLL12	1	6	6	13.8	74.403	17226000	24582000	23516000	20913000	20535000	24915000	1.015905	0.903736
Q9UDY2	Tight junction protein ZO-2	TJP2	1	10	10	10.9	133.96	20775000	15514000	16374000	17803000	17509000	16734000	0.988284	0.907389
O95218	Zinc finger Ran-binding domain-containing	ZRANB2	1	3	3	7	37.404	19141000	NaN	17640000	17539000	18196000	19824000	1.007024	0.909018
P51553	Isocitrate dehydrogenase [NAD] subunit ga	IDH3G	1	6	6	25.4	42.794	22558000	NaN	29130000	27360000	22797000	26254000	0.985541	0.909481
O75886	Signal transducing adapter molecule 2	STAM2	1	6	4	13.9	58.164	11479000	17343000	21205000	18473000	15759000	16859000	1.021269	0.909651
O15305	Phosphomannomutase 2	PMM2	1	3	3	15	28.082	16982000	20306000	18895000	17005000	20040000	NaN	0.989045	0.910573
Q8TC12	Retinol dehydrogenase 11	RDH11	1	6	6	25.8	35.386	NaN	22252000	32057000	26106000	23828000	29954000	0.98066	0.910967
Q9Y295	Developmentally-regulated GTP-binding p	DRG1	1	4	4	14.4	40.542	16990000	20421000	19317000	19048000	20070000	18022000	1.007263	0.912318
P68036;A0A1B0GUS4	Ubiquitin-conjugating enzyme E2 L3	UBE2L3	2	7	7	48.7	17.861	1.24E+08	1.64E+08	1.93E+08	1.59E+08	1.44E+08	1.85E+08	1.016613	0.91473
Q12849	G-rich sequence factor 1	GRSF1	1	5	5	14.4	53.126	21350000	20325000	22314000	22362000	21373000	20521000	1.004173	0.914956
Q93008	Probable ubiquitin carboxyl-terminal hydro	USP9X	1	20	11	10.6	290.46	23769000	25206000	28431000	22892000	24895000	28861000	0.990207	0.915276
Q9Y6E2	Basic leucine zipper and W2 domain-conta	BZW2	1	4	4	9.3	48.162	23041000	28357000	23810000	23565000	23436000	29045000	1.011142	0.91587
Q9NX46	Poly(ADP-ribose) glycohydrolase ARH3	ADPRHL2	1	6	6	25.1	38.946	13045000	14801000	15157000	13140000	15526000	14655000	1.007395	0.91697

O00203;Q13367	AP-3 complex subunit beta-1	AP3B1	2	14	14	14.7	121.32	39612000	47849000	40316000	44551000	37681000	44410000	0.991117	0.918588
Q8NBU5	ATPase family AAA domain-containing pro	ATAD1	1	4	4	19.9	40.744	18750000	22915000	14928000	17830000	16709000	21184000	0.984627	0.918726
O94901	SUN domain-containing protein 1	SUN1	1	4	4	7.4	87.109	20418000	17030000	12728000	17387000	16703000	NaN	1.019113	0.918739
P36873	Serine/threonine-protein phosphatase PP1	PPP1CC	1	11	2	39.6	36.983	42077000	52894000	NaN	46241000	47480000	NaN	0.986838	0.919085
Q8WU90	Zinc finger CCCH domain-containing prote	ZC3H15	1	4	4	14.6	48.602	24172000	22158000	21386000	20713000	20519000	27249000	1.011297	0.919243
Q71DI3	Histone H3.2	HIST2H3A	1	4	1	40.4	15.388	82817000	NaN	1.04E+08	1.26E+08	NaN	52059000	0.95286	0.919278
P46109	Crk-like protein	CRKL	1	5	5	26.4	33.777	27551000	24005000	27345000	24167000	23903000	30085000	0.990545	0.919886
Q9BQS8	FYVE and coiled-coil domain-containing pı	FYCO1	1	18	18	19.2	166.98	35445000	31273000	33801000	28743000	35543000	35432000	0.992031	0.921799
Q96C19	EF-hand domain-containing protein D2	EFHD2	1	7	6	35.4	26.697	40048000	42944000	43942000	42074000	45560000	38574000	0.99428	0.92229
P29728	2-5-oligoadenylate synthase 2	OAS2	1	7	7	14.5	82.43	1.07E+08	66378000	97133000	98783000	88486000	87241000	1.014671	0.922496
O94804	Serine/threonine-protein kinase 10	STK10	1	4	3	5.5	112.13	17532000	21713000	22704000	18070000	23856000	NaN	1.015174	0.922704
Q3ZCM7	Tubulin beta-8 chain	TUBB8	1	9	2	21.4	49.775	2.88E+08	3.52E+08	2.84E+08	2.76E+08	2.45E+08	3.88E+08	0.984107	0.924873
P19634	Sodium/hydrogen exchanger 1	SLC9A1	1	4	4	6.7	90.762	22840000	19887000	13457000	20007000	18191000	NaN	1.01981	0.925237
Q14980	Nuclear mitotic apparatus protein 1	NUMA1	1	20	20	13	238.26	13258000	20852000	13296000	15169000	15100000	17933000	1.016791	0.926224
O15427	Monocarboxylate transporter 4	SLC16A3	1	4	4	11	49.469	1.08E+08	1.15E+08	1.05E+08	1.02E+08	1.16E+08	1.12E+08	1.004477	0.927565
P43307	Translocon-associated protein subunit alph	SSR1	1	3	3	12.6	32.235	89735000	67692000	1.02E+08	84593000	1.01E+08	76866000	1.013485	0.928574
Q86V48	Leucine zipper protein 1	LUZP1	1	10	10	13.4	120.27	18588000	18922000	16744000	18326000	20371000	15082000	0.991245	0.929537
Q9UPT5	Exocyst complex component 7	EXOC7	1	8	8	15.9	83.381	20904000	30149000	45267000	33535000	37796000	27167000	1.022612	0.92981
P78347	General transcription factor II-I	GTF2I	1	3	3	4.5	112.42	14969000	10441000	NaN	13033000	12824000	NaN	1.017591	0.930438
Q15125	3-beta-hydroxysteroid-Delta(8),Delta(7)-is	EBP	1	1	1	5.2	26.352	97075000	1E+08	1E+08	75907000	1.15E+08	1.1E+08	1.010719	0.935523
O95070	Protein YIF1A	YIF1A	1	2	2	9.2	32.011	22480000	25544000	19685000	27549000	21454000	17856000	0.987446	0.935632
Q9NWV4	UPF0587 protein C1orf123	C1orf123	1	4	4	40	18.048	31773000	42630000	37568000	30863000	38399000	43968000	1.011244	0.93619
Q9Y5L0	Transportin-3	TNPO3	1	5	5	8	104.2	7906500	8424600	NaN	9509700	NaN	6552200	0.983516	0.93673
Q6UWP7	Lysocardiolipin acyltransferase 1	LCLAT1	1	2	2	8.2	48.92	9528600	11625000	NaN	10666000	9785500	11042000	0.992534	0.936823
Q9H9Q2	COP9 signalosome complex subunit 7b	COPS7B	1	3	3	16.7	29.622	58252000	37613000	NaN	44423000	53433000	NaN	1.020769	0.937606
O00461	Golgi integral membrane protein 4	GOLIM4	1	10	10	18.1	81.879	35459000	32827000	42040000	30119000	34799000	44185000	0.988915	0.938453
Q12905	Interleukin enhancer-binding factor 2	ILF2	1	9	9	25.9	43.062	1.17E+08	1.08E+08	1E+08	1.07E+08	1.15E+08	1.05E+08	1.00446	0.938567
P22307	Non-specific lipid-transfer protein	SCP2	1	9	9	11.5	58.993	72710000	99873000	74086000	65242000	77609000	1E+08	0.986468	0.938662
Q13642	Four and a half LIM domains protein 1	FHL1	1	4	4	16.4	36.263	33297000	38046000	28370000	26262000	33502000	38848000	0.988958	0.940162
Q7Z4H3	HD domain-containing protein 2	HDDC2	1	3	3	21.6	23.39	11239000	12712000	NaN	9941600	10349000	15100000	0.985083	0.940585
P54577	Tyrosine--tRNA ligase, cytoplasmic;Tyrosin	YARS	1	17	17	37.9	59.143	1.1E+08	97085000	1.2E+08	1.05E+08	1.2E+08	1.04E+08	1.00558	0.94585
P20340;Q9NRW1;Q14964	Ras-related protein Rab-6A	RAB6A	3	6	6	29.3	23.593	6.06E+08	4.66E+08	5.3E+08	5.18E+08	5.95E+08	4.99E+08	1.006722	0.946167
Q9UBQ0	Vacuolar protein sorting-associated proteiı	VPS29	1	4	4	25.3	20.505	58351000	64976000	60309000	63470000	62271000	58422000	1.00287	0.947057
P07437;A6NNZ2	Tubulin beta chain	TUBB	2	20	4	59.7	49.67	1.85E+09	2.08E+09	1.76E+09	1.78E+09	1.89E+09	2E+09	0.995996	0.949566
Q9UG63	ATP-binding cassette sub-family F membe	ABCF2	1	9	9	20.5	71.289	22319000	20168000	25424000	17700000	25286000	25525000	1.008835	0.949849
Q99653	Calcineurin B homologous protein 1	CHP1	1	5	5	29.7	22.456	39140000	66342000	37484000	61102000	30109000	54396000	1.018473	0.9503
P11310	Medium-chain specific acyl-CoA dehydrog	ACADM	1	4	4	11.2	46.588	33618000	30033000	33347000	35275000	34004000	28205000	1.00501	0.950701
P10155	60 kDa SS-A/Ro ribonucleoprotein	TROVE2	1	12	12	24.3	60.67	1.33E+08	1.41E+08	1.48E+08	1.23E+08	1.59E+08	1.38E+08	0.994887	0.951882
P06733;P13929	Alpha-enolase	ENO1	2	25	23	68.2	47.168	1.16E+09	1.23E+09	1.04E+09	1.1E+09	1.14E+09	1.19E+09	0.996682	0.95325
O60502	Protein O-GlcNAcase	MGEA5	1	3	3	5.3	102.91	14526000	15252000	17451000	16712000	18396000	12473000	1.007453	0.955347
O43768	Alpha-endosulfine	ENSA	1	4	3	40.5	13.389	52800000	35472000	58044000	51227000	47628000	46253000	0.991744	0.95676
P08621	U1 small nuclear ribonucleoprotein 70 kDa	SNRNP70	1	6	6	18.1	51.556	57171000	35405000	48198000	44249000	54313000	41014000	0.99149	0.959973
P06132	Uroporphyrinogen decarboxylase	UROD	1	3	3	12.3	40.786	39223000	32786000	32854000	36321000	39709000	29410000	1.005502	0.961106
P59998	Actin-related protein 2/3 complex subunit	ARPC4	1	9	9	71.4	19.667	2.32E+08	2.48E+08	2.2E+08	2.29E+08	2.33E+08	2.38E+08	0.998173	0.962914
O94906	Pre-mRNA-processing factor 6	PRPF6	1	7	7	9.5	106.92	11901000	14049000	11474000	10739000	13365000	13164000	0.995832	0.966409
P12004	Proliferating cell nuclear antigen	PCNA	1	9	9	52.5	28.768	1.35E+08	1.27E+08	1.31E+08	1.28E+08	1.21E+08	1.44E+08	0.997536	0.96729
P04899;P11488;P19087;P0947	Guanine nucleotide-binding protein G(i) su	GNAI2	5	11	8	37.7	40.45	64765000	77107000	65011000	70958000	61321000	73883000	0.996515	0.967637
P62993	Growth factor receptor-bound protein 2	GRB2	1	6	6	30.9	25.206	33795000	22283000	32740000	28718000	32687000	27918000	1.005686	0.968123
P40261	Nicotinamide N-methyltransferase	NNMT	1	4	4	16.7	29.574	45132000	43675000	50459000	47844000	46940000	44185000	0.997867	0.968242
A0FGR8	Extended synaptotagmin-2	ESYT2	1	7	7	11.2	102.36	18834000	21391000	16720000	18554000	20068000	18139000	0.996769	0.968765
Q6P587	Acylpyruvase FAHD1, mitochondrial	FAHD1	1	3	3	19.6	24.843	63898000	50840000	60990000	53701000	62890000	59689000	1.003141	0.971183
Q9Y6G9	Cytoplasmic dynein 1 light intermediate ch	DYNC1LI1	1	12	11	32.9	56.578	24530000	27096000	29627000	27863000	28833000	24777000	1.002708	0.971261
P21399	Cytoplasmic aconitate hydratase	ACO1	1	16	16	24.3	98.398	99872000	1.2E+08	1.11E+08	1.09E+08	1.01E+08	1.2E+08	0.997212	0.97145
Q13283	Ras GTPase-activating protein-binding pro	G3BP1	1	11	10	33.3	52.164	63927000	77761000	58116000	62944000	57978000	77937000	0.99527	0.971759
P36405	ADP-ribosylation factor-like protein 3	ARL3	1	5	5	42.9	20.455	59800000	70975000	64991000	69058000	63730000	62575000	0.997941	0.973466
Q9BQA1	Methylosome protein 50	WDR77	1	5	5	23.4	36.724	26001000	33914000	31804000	28929000	31429000	31625000	1.002878	0.973815
Q9BVL2	Nucleoporin p58/p45	NUPL1	1	3	3	8.3	60.896	21075000	18743000	NaN	22898000	16719000	19864000	0.995881	0.975517
Q9ULH1	Arf-GAP with SH3 domain, ANK repeat anc	ASAP1	1	5	5	6.2	125.5	15492000	NaN	13426000	9610500	18356000	15750000	1.007827	0.975782
Q96JJ3	Engulfment and cell motility protein 2	ELMO2	1	7	7	16.1	82.614	14640000	15104000	12000000	15122000	14393000	12350000	1.002899	0.976258

Q9ULC4;A0A3B3IRV3	Malignant T-cell-amplified sequence 1	MCTS1	2	4	4	31.5	20.555	29770000	38372000	NaN	46015000	33564000	23484000	1.008316	0.976869
Q13243	Serine/arginine-rich splicing factor 5	SRSF5	1	4	3	22.4	31.263	23805000	21151000	31521000	23535000	22302000	31020000	1.004969	0.977026
P46108	Adapter molecule crk	CRK	1	8	8	38.8	33.83	40537000	34749000	35276000	32264000	39629000	38918000	1.002252	0.979155
P18621	60S ribosomal protein L17	RPL17	1	6	6	37.5	21.397	2.07E+08	2.33E+08	2.25E+08	2.25E+08	2.01E+08	2.39E+08	0.998331	0.979689
Q9BQG0	Myb-binding protein 1A	MYBBP1A	1	12	12	10.6	148.85	12906000	11884000	14529000	11036000	12861000	15327000	0.997584	0.983762
O95834	Echinoderm microtubule-associated protei	EML2	1	10	10	20.2	70.678	40146000	39011000	39178000	40014000	42323000	35878000	0.998986	0.984362
Q16543	Hsp90 co-chaperone Cdc37;Hsp90 co-cha	CDC37	1	10	10	33.3	44.468	1.46E+08	1.64E+08	1.6E+08	1.42E+08	1.48E+08	1.79E+08	0.998573	0.986926
Q6XZF7	Dynamin-binding protein	DNMBP	1	9	9	8.6	177.35	54354000	60147000	65270000	48663000	62220000	68573000	0.998248	0.988185
Q99598	Translin-associated protein X	TSNAX	1	5	5	26.6	33.112	14232000	17959000	19521000	15041000	16681000	20090000	1.001934	0.988435
Q5T4S7	E3 ubiquitin-protein ligase UBR4	UBR4	1	25	25	6.6	573.83	43531000	42676000	37669000	38358000	41049000	44574000	1.000848	0.989768
P46939	Utrophin	UTRN	1	8	8	3.1	394.46	13948000	8900000	11928000	9988200	14556000	10309000	1.00222	0.990713
P29466	Caspase-1;Caspase-1 subunit p20;Caspase	CASP1	1	8	8	28.7	45.158	1.14E+08	1.3E+08	97338000	1.09E+08	1.08E+08	1.25E+08	0.998837	0.991041
P61106	Ras-related protein Rab-14	RAB14	1	9	9	48.8	23.897	1.2E+08	1.3E+08	1.18E+08	1.24E+08	1.14E+08	1.3E+08	0.999511	0.992707
Q86VS8	Protein Hook homolog 3	HOOK3	1	7	7	13.2	83.125	32465000	30057000	33998000	25733000	34421000	36457000	1.000943	0.993468
Q8IXB1	DnaJ homolog subfamily C member 10	DNAJC10	1	6	6	8.8	91.079	20563000	22831000	19376000	18206000	22022000	22575000	1.000526	0.995167
O75832	26S proteasome non-ATPase regulatory su	PSMD10	1	7	7	37.2	24.428	67519000	45329000	64787000	57980000	65785000	54017000	1.000828	0.995285
Q96TA1	Niban-like protein 1	FAM129B	1	17	17	24.8	84.137	1.74E+08	1.65E+08	1.84E+08	1.8E+08	1.79E+08	1.64E+08	0.99977	0.996002
O43747	AP-1 complex subunit gamma-1	AP1G1	1	8	8	13.4	91.35	96136000	73524000	70336000	74853000	81834000	83358000	1.000204	0.998564
P42167;P42166	Lamina-associated polypeptide 2, isoforms	TMPO	2	7	7	19.4	50.67	24960000	17434000	20219000	22825000	21689000	18087000	0.999808	0.998855

Table S5. A full list of human proteins within Co-IP pellets (immunoprecipitates) as identified by MS assays

Protein IDs	Majority protein IDs	Protein names	Gene names	Number of proteins	Peptides	Unique peptides	Sequence coverage [%]	Mol. weight [kDa]	LFQ intensity_BLEO-IgG	LFQ intensity_BLEO-PRDX6
Q71UI9;P0C0S5	Q71UI9;P0C0S5	Histone H2A.V;Histone H2A.Z	H2AFV;H2AFZ	2	4	2	31.2	13.509	1185600000	544120000
P42766	P42766	60S ribosomal protein L35	RPL35	1	2	2	18.7	14.551	70202000	124700000
P05114	P05114	Non-histone chromosomal protein HMG-14	HMGN1	1	2	2	14	10.659	77778000	51605000
P17096	P17096	High mobility group protein HMG-I/HMG-Y	HMGA1	1	2	2	23.4	11.676	42362000	42933000
Q15366;P57721	Q15366;P57721	Poly(rC)-binding protein 2;Poly(rC)-binding pr	PCBP2;PCBP3	2	4	2	16.7	38.58	34190000	41058000
Q9Y3Y2	Q9Y3Y2	Chromatin target of PRMT1 protein	CHTOP	1	2	2	10.5	26.396	69893000	31154000
P15559	P15559	NAD(P)H dehydrogenase [quinone] 1	NQO1	1	2	2	7.7	30.867	53409000	25286000
Q9NUL5	Q9NUL5	UPF0515 protein C19orf66	C19orf66	1	2	2	8.9	33.11	29996000	20848000
Q08170;Q13247;Q13243	Q08170;Q13247;Q13243	Serine/arginine-rich splicing factor 4;Serine/arg	SRSF4;SRSF6;SRSF5	3	2	2	4.3	56.678	44793000	14436000
Q99439	Q99439	Calponin-2	CNN2	1	2	2	7.8	33.697	14649000	13076000
P12235	P12235	ADP/ATP translocase 1	SLC25A4	1	8	2	24.5	33.064	7299900	12498000
P53621	P53621	Coatomer subunit alpha;Xenin;Proxenin	COPA	1	2	2	1.6	138.34	9631500	12406000
P10515	P10515	Dihydrolipoyllysine-residue acetyltransferase c	DLAT	1	2	2	4.5	68.996	10844000	8406800
Q5SNT2	Q5SNT2	Transmembrane protein 201	TMEM201	1	2	2	3.5	72.235	10228000	8297000
P05976;P08590	P05976;P08590	Myosin light chain 1/3, skeletal muscle isoform	MYL1;MYL3	2	2	2	8.2	21.145	3240600000	1443400000
A0A0J9YX35	A0A0J9YX35			1	2	2	18.8	12.822	89742000	NaN
P51636	P51636	Caveolin-2	CAV2	1	2	2	17.3	18.291	123450000	NaN
P78347	P78347	General transcription factor II-I	GTF2I	1	2	2	3.3	112.42	26218000	NaN
P29558;Q6XE24	P29558;Q6XE24	RNA-binding motif, single-stranded-interactin	RBMS1;RBMS3	2	2	2	7.4	44.505	32036000	NaN
Q9NVI7;Q5T2N8	Q9NVI7	ATPase family AAA domain-containing proteir	ATAD3A	2	6	2	12.3	71.368	18358000	39716000
Q01081;Q8WU68	Q01081;Q8WU68	Splicing factor U2AF 35 kDa subunit;Splicing fa	U2AF1;U2AF1L4	2	2	2	11.2	27.872	26943000	8511200
P02745	P02745	Complement C1q subcomponent subunit A	C1QA	1	2	2	4.5	26.016	NaN	67616000
P16403;P22492;Q02539	P16403	Histone H1.2	HIST1H1C	3	7	2	25.4	21.364	540570000	261400000
P07437;A6NNZ2;CON__ENSEM	P07437	Tubulin beta chain	TUBB	4	15	2	35.1	49.67	116680000	243860000
P61254	P61254	60S ribosomal protein L26	RPL26	1	8	2	44.1	17.258	268850000	318930000
Q13885;Q9BVA1	Q13885;Q9BVA1	Tubulin beta-2A chain;Tubulin beta-2B chain	TUBB2A;TUBB2B	2	16	2	38.9	49.906	NaN	208220000
Q13509	Q13509	Tubulin beta-3 chain	TUBB3	1	15	2	34.7	50.432	38293000	229140000
Q71DI3;Q16695;P84243;P6843	Q71DI3;Q16695;P84243;P68	Histone H3.2;Histone H3.1t;Histone H3.3;Histor	HIST2H3A;HIST3H3;H3F3.	5	4	2	19.9	15.388	3234500000	2910900000
Q99879;Q99877;Q93079;Q5QN	Q99879;Q99877;Q93079;Q5	Histone H2B type 1-M;Histone H2B type 1-N;H	HIST1H2BM;HIST1H2BN;H	11	7	2	46.8	13.989	8629400000	3912400000
P01344	P01344	Insulin-like growth factor II;Insulin-like growth	IGF2	1	2	2	13.9	20.14	22920000	40873000
P46779	P46779	60S ribosomal protein L28	RPL28	1	2	2	16.1	15.747	57641000	96260000
Q71UM5	Q71UM5	40S ribosomal protein S27-like	RPS27L	1	4	2	40.5	9.4771	131880000	232960000
P42677	P42677	40S ribosomal protein S27	RPS27	1	4	2	40.5	9.461	27017000	38356000
P98179	P98179	RNA-binding protein 3	RBM3	1	2	2	14	17.17	74452000	27403000
Q3ZCQ8	Q3ZCQ8	Mitochondrial import inner membrane translo	TIMM50	1	2	2	7.6	39.646	NaN	27065000
O60783	O60783	28S ribosomal protein S14, mitochondrial	MRPS14	1	2	2	21.9	15.139	NaN	NaN
P09234	P09234	U1 small nuclear ribonucleoprotein C	SNRPC	1	2	2	18.9	17.394	NaN	43358000
Q9NZT1	Q9NZT1	Calmodulin-like protein 5	CALML5	1	2	2	15.1	15.892	NaN	NaN
P35232	P35232	Prohibitin	PHB	1	2	2	8.1	29.804	26473000	30233000
O94905;O75477	O94905;O75477	Erlin-2;Erlin-1	ERLIN2;ERLIN1	2	2	2	7.1	37.839	20948000	NaN
Q9NVJ2;Q96BM9	Q9NVJ2;Q96BM9	ADP-ribosylation factor-like protein 8B;ADP-ri	ARL8B;ARL8A	2	2	2	10.2	21.539	NaN	17795000
Q9UNZ5	Q9UNZ5	Leydig cell tumor 10 kDa protein homolog	C19orf53	1	2	2	16.2	10.576	45865000	NaN
P52943	P52943	Cysteine-rich protein 2	CRIP2	1	3	3	32.2	22.492	43460000	144580000
P07305	P07305	Histone H1.0;Histone H1.0, N-terminally proce	H1F0	1	3	3	18.6	20.863	64820000	78013000
P16070	P16070	CD44 antigen	CD44	1	3	3	5.3	81.537	54375000	45704000
Q86U42	Q86U42	Polyadenylate-binding protein 2	PABPN1	1	3	3	12.7	32.749	44881000	41778000
Q07955	Q07955	Serine/arginine-rich splicing factor 1	SRSF1	1	3	3	12.5	27.744	80555000	39912000
P09669	P09669	Cytochrome c oxidase subunit 6C	COX6C	1	3	3	37.3	8.7813	49437000	32441000
P52926	P52926	High mobility group protein HMGI-C	HMGA2	1	3	3	33	11.832	46771000	30117000
Q15287	Q15287	RNA-binding protein with serine-rich domain	RNPS1	1	3	3	11.5	34.208	73954000	29126000
Q12797	Q12797	Aspartyl/asparaginyl beta-hydroxylase	ASPH	1	3	3	7.3	85.862	13104000	24934000
Q00577	Q00577	Transcriptional activator protein Pur-alpha	PURA	1	3	3	18	34.91	26062000	24130000
Q8TED0	Q8TED0	U3 small nucleolar RNA-associated protein 15	UTP15	1	3	3	6.9	58.414	41952000	22926000

Q9H0A0	Q9H0A0	N-acetyltransferase 10	NAT10	1	3	3	4.2	115.73	25675000	21639000
Q9UJS0;O75746	Q9UJS0	Calcium-binding mitochondrial carrier protein	SLC25A13	2	3	3	6.1	74.175	8696300	21456000
Q9NX24	Q9NX24	H/ACA ribonucleoprotein complex subunit 2	NHP2	1	3	3	24.8	17.201	59010000	21365000
Q99848	Q99848	Probable rRNA-processing protein EBP2	EBNA1BP2	1	3	3	14.4	34.852	38647000	20437000
P48729;Q8N752	P48729;Q8N752	Casein kinase I isoform alpha;Casein kinase I is	CSNK1A1;CSNK1A1L	2	3	3	12.8	38.914	10023000	20108000
P31946	P31946	14-3-3 protein beta/alpha;14-3-3 protein bet	YWHAB	1	5	3	25.6	28.082	17248000	15769000
O94925	O94925	Glutaminase kidney isoform, mitochondrial	GLS	1	3	3	7.3	73.46	20824000	15441000
Q9BZE4	Q9BZE4	Nucleolar GTP-binding protein 1	GTPBP4	1	3	3	5.2	73.964	30637000	14750000
O15231	O15231	Zinc finger protein 185	ZNF185	1	3	3	9.4	73.525	23470000	14405000
O94907	O94907	Dickkopf-related protein 1	DKK1	1	3	3	11.7	28.671	43811000	13627000
P42224	P42224	Signal transducer and activator of transcriptior	STAT1	1	3	3	4.8	87.334	17196000	11022000
P04075	P04075	Fructose-bisphosphate aldolase A	ALDOA	1	3	3	14	39.42	25456000	9139100
P37802	P37802	Transgelin-2	TAGLN2	1	3	3	15.6	22.391	15582000	8817100
Q9Y4W2	Q9Y4W2	Ribosomal biogenesis protein LAS1L	LAS1L	1	3	3	5.4	83.064	14029000	5378100
Q71RC2	Q71RC2	La-related protein 4	LARP4	1	3	3	7	80.595	NaN	63360000
P24844	P24844	Myosin regulatory light polypeptide 9	MYL9	1	10	3	50.6	19.827	452350000	742120000
Q9UMS4	Q9UMS4	Pre-mRNA-processing factor 19	PRPF19	1	3	3	10.9	55.18	91962000	29137000
P17931	P17931	Galectin-3	LGALS3	1	3	3	14.4	26.152	38476000	24680000
O43809	O43809	Cleavage and polyadenylation specificity facto	NUDT21	1	3	3	16.7	26.227	21396000	NaN
P0DOX7;P01834	P0DOX7;P01834	Ig kappa chain C region	IGKC	2	3	3	25.7	23.379	23431000	NaN
P07951	P07951	Tropomyosin beta chain	TPM2	1	30	3	68.3	32.85	288720000	407550000
Q8N684	Q8N684	Cleavage and polyadenylation specificity facto	CPSF7	1	3	3	8.9	52.049	7393400	NaN
Q86UE4	Q86UE4	Protein LYRIC	MTDH	1	3	3	6.9	63.836	30341000	NaN
P62273	P62273	40S ribosomal protein S29	RPS29	1	3	3	33.9	6.6767	4512100000	2518700000
P46776	P46776	60S ribosomal protein L27a	RPL27A	1	3	3	22.3	16.561	258790000	414490000
P62979	P62979	Ubiquitin-40S ribosomal protein S27a;Ubiquiti	RPS27A	1	7	3	51.3	17.965	384130000	382820000
P35268	P35268	60S ribosomal protein L22	RPL22	1	3	3	39.1	14.787	228400000	235190000
P46778	P46778	60S ribosomal protein L21	RPL21	1	3	3	27.5	18.565	53460000	132300000
Q92567	Q92567	Protein FAM168A	FAM168A	1	3	3	22.5	26.184	NaN	35981000
P81605	P81605	Dermcidin;Survival-promoting peptide;DCD-1	DCD	1	3	3	22.7	11.284	65217000	89200000
Q8IY67	Q8IY67	Ribonucleoprotein PTB-binding 1	RAVER1	1	3	3	7.1	63.876	NaN	NaN
P49207	P49207	60S ribosomal protein L34	RPL34	1	3	3	22.2	13.293	26371000	107910000
P16401	P16401	Histone H1.5	HIST1H1B	1	3	3	17.3	22.58	51352000	27223000
O60884	O60884	DnaJ homolog subfamily A member 2	DNAJA2	1	3	3	10.7	45.745	NaN	20838000
Q9GZT3	Q9GZT3	SRA stem-loop-interacting RNA-binding prote	SLIRP	1	3	3	33.9	12.349	27033000	NaN
P62820;Q9H0U4;Q92928;P511	P62820;Q9H0U4;Q92928	Ras-related protein Rab-1A;Ras-related protei	RAB1A;RAB1B;RAB1C	7	4	3	25.4	22.677	43525000	53137000
Q8NEY8	Q8NEY8	Periphilin-1	PPHLN1	1	3	3	7.9	52.736	31853000	23553000
Q9BUF5	Q9BUF5	Tubulin beta-6 chain	TUBB6	1	12	3	31.8	49.857	NaN	51416000
Q02413	Q02413	Desmoglein-1	DSG1	1	3	3	4.9	113.75	NaN	18844000
P62258	P62258	14-3-3 protein epsilon	YWHAE	1	4	3	18	29.174	NaN	23855000
P62937;P0DN26;A0A0B4J2A2;A	P62937	Peptidyl-prolyl cis-trans isomerase A;Peptidyl-	PPIA	7	3	3	17.6	18.012	25628000	35117000
Q9NUL3	Q9NUL3	Double-stranded RNA-binding protein Staufe	STAU2	1	4	3	8.1	62.608	NaN	10149000
O75531	O75531	Barrier-to-autointegration factor;Barrier-to-au	BANF1	1	4	4	50.6	10.058	175070000	1327000000
Q96S97	Q96S97	Myeloid-associated differentiation marker	MYADM	1	4	4	18.6	35.273	173440000	264480000
P09038	P09038	Fibroblast growth factor 2	FGF2	1	4	4	12.5	30.77	153780000	152580000
O95817	O95817	BAG family molecular chaperone regulator 3	BAG3	1	4	4	9.6	61.594	355320000	73281000
P28799	P28799	Granulins;Acrogranin;Paragranulin;Granulin-1;(	GRN	1	4	4	8.6	63.544	246140000	62402000
P05198	P05198	Eukaryotic translation initiation factor 2 subuni	EIF2S1	1	4	4	16.2	36.112	13828000	51685000
Q9H2D6	Q9H2D6	TRIO and F-actin-binding protein	TRIOBP	1	5	4	2.4	261.37	45222000	48586000
Q96EY1	Q96EY1	DnaJ homolog subfamily A member 3, mitoch	DNAJA3	1	4	4	12.1	52.488	17527000	38148000
O15145	O15145	Actin-related protein 2/3 complex subunit 3	ARPC3	1	4	4	22.5	20.546	24960000	31244000
P00387	P00387	NADH-cytochrome b5 reductase 3;NADH-cyt	CYB5R3	1	4	4	19.3	34.234	66011000	29485000
Q15050	Q15050	Ribosome biogenesis regulatory protein homo	RRS1	1	4	4	15.6	41.193	72935000	27650000
P21589	P21589	5-nucleotidase	NT5E	1	4	4	9.8	63.367	24755000	25818000
P52294;O60684	P52294	Importin subunit alpha-5;Importin subunit alp	KPNA1	2	4	4	11.5	60.221	18360000	24061000
P49748	P49748	Very long-chain specific acyl-CoA dehydroger	ACADVL	1	4	4	8.9	70.389	17348000	23675000
O95470	O95470	Sphingosine-1-phosphate lyase 1	SGPL1	1	4	4	10.9	63.523	120390000	21861000
P23284	P23284	Peptidyl-prolyl cis-trans isomerase B	PPIB	1	4	4	21.3	23.742	17313000	19530000
Q9BYG3	Q9BYG3	MKI67 FHA domain-interacting nucleolar phos	NIFK	1	4	4	19.5	34.222	63274000	19201000

P08582	P08582	Melanotransferrin	MFI2	1	4	4	8.9	80.214	24972000	17043000
Q15746	Q15746	Myosin light chain kinase, smooth muscle;Myo	MYLK	1	4	4	3	210.71	28309000	13801000
O00541	O00541	Pescadillo homolog	PES1	1	4	4	9.4	68.002	20809000	13167000
Q9Y3T9	Q9Y3T9	Nucleolar complex protein 2 homolog	NOC2L	1	4	4	5.5	84.918	57309000	12518000
Q03135;P56539	Q03135	Caveolin-1	CAV1	2	4	4	23	20.471	175040000	134530000
P02452	P02452	Collagen alpha-1(I) chain	COL1A1	1	4	4	3.3	138.91	81355000	NaN
Q96CW1	Q96CW1	AP-2 complex subunit mu	AP2M1	1	4	4	12.6	49.654	87198000	30586000
O75569	O75569	Interferon-inducible double-stranded RNA-de	PRKRA	1	4	4	15.7	34.404	42999000	42259000
P36957	P36957	Dihydrolipoyllysine-residue succinyltransferase	DLST	1	4	4	12.8	48.755	26506000	31325000
Q9NX05	Q9NX05	Constitutive coactivator of PPAR-gamma-like	FAM120C	1	4	4	5.1	120.57	15772000	14421000
P50238	P50238	Cysteine-rich protein 1	CRIP1	1	4	4	71.4	8.5328	1178100000	1649300000
Q6P1L8	Q6P1L8	39S ribosomal protein L14, mitochondrial	MRPL14	1	4	4	35.2	15.947	420990000	482540000
P62854;Q5JNZ5	P62854;Q5JNZ5	40S ribosomal protein S26;Putative 40S riboso	RPS26;RPS26P11	2	4	4	37.4	13.015	187790000	402890000
P60866	P60866	40S ribosomal protein S20	RPS20	1	4	4	28.6	13.373	296500000	448520000
Q14011	Q14011	Cold-inducible RNA-binding protein	CIRBP	1	4	4	33.1	18.648	548740000	256880000
P62829	P62829	60S ribosomal protein L23	RPL23	1	4	4	37.9	14.865	376490000	213530000
P23528;Q9Y281	P23528	Cofilin-1	CFL1	2	4	4	28.9	18.502	299710000	206720000
P00338;Q6ZMR3;P07864	P00338	L-lactate dehydrogenase A chain	LDHA	3	5	4	19	36.688	NaN	122160000
Q99729	Q99729	Heterogeneous nuclear ribonucleoprotein A/B	HNRNPAB	1	4	4	12.3	36.224	68249000	54076000
P84098	P84098	60S ribosomal protein L19	RPL19	1	4	4	21.9	23.466	140810000	238390000
P30050	P30050	60S ribosomal protein L12	RPL12	1	4	4	40	17.818	114180000	368940000
Q15365	Q15365	Poly(rC)-binding protein 1	PCBP1	1	6	4	23.3	37.497	103690000	67465000
P62847	P62847	40S ribosomal protein S24	RPS24	1	4	4	26.3	15.423	100410000	179620000
P50914	P50914	60S ribosomal protein L14	RPL14	1	4	4	20.9	23.432	134830000	294040000
P62841	P62841	40S ribosomal protein S15	RPS15	1	4	4	29	17.04	148330000	164000000
P25398	P25398	40S ribosomal protein S12	RPS12	1	4	4	38.6	14.515	30362000	64007000
P62910	P62910	60S ribosomal protein L32	RPL32	1	4	4	33.3	15.86	47392000	305210000
O95793	O95793	Double-stranded RNA-binding protein Staufer	STAU1	1	5	4	11.4	63.182	42863000	33611000
P18085;P84085	P18085	ADP-ribosylation factor 4	ARF4	2	5	4	29.4	20.511	49742000	84541000
Q99623	Q99623	Prohibitin-2	PHB2	1	4	4	16.7	33.296	72594000	67992000
P62633	P62633	Cellular nucleic acid-binding protein	CNBP	1	4	4	30.5	19.463	17076000	NaN
P68366	P68366	Tubulin alpha-4A chain	TUBA4A	1	19	4	52.2	49.924	39055000	158220000
P29692	P29692	Elongation factor 1-delta	EEF1D	1	4	4	21	31.121	16348000	19692000
P62140	P62140	Serine/threonine-protein phosphatase PP1-be	PPP1CB	1	10	4	38.2	37.186	290140000	238120000
P62995	P62995	Transformer-2 protein homolog beta	TRA2B	1	6	4	20.8	33.665	171890000	32081000
Q16527	Q16527	Cysteine and glycine-rich protein 2	CSRP2	1	4	4	24.4	20.954	NaN	35446000
A0A8I5KQE6;P08865	A0A8I5KQE6;P08865	40S ribosomal protein SA	RPSA	2	4	4	19.3	32.909	25136000	20553000
O43920	O43920	NADH dehydrogenase [ubiquinone] iron-sulfu	NDUFS5	1	4	4	44.3	12.517	NaN	42550000
Q9BVC5	Q9BVC5	Ashwin	C2orf49	1	4	4	26.3	25.858	NaN	62438000
P29401	P29401	Transketolase	TKT	1	4	4	11.4	67.877	26133000	27960000
Q92522	Q92522	Histone H1x	H1FX	1	4	4	25.8	22.487	24330000	59176000
P42704	P42704	Leucine-rich PPR motif-containing protein, mi	LRPPRC	1	5	5	4.4	157.9	3140800000	2424900000
Q13573	Q13573	SNW domain-containing protein 1	SNW1	1	5	5	14.2	61.494	873120000	610080000
P48307	P48307	Tissue factor pathway inhibitor 2	TFPI2	1	5	5	27.2	26.934	163470000	161550000
P63000;P60763;P15153	P63000;P60763;P15153	Ras-related C3 botulinum toxin substrate 1;Ra	RAC1;RAC3;RAC2	3	6	5	36.5	21.45	102610000	121810000
Q15293	Q15293	Reticulocalbin-1	RCN1	1	5	5	20.5	38.89	19794000	87458000
P04004	P04004	Vitronectin;Vitronectin V65 subunit;Vitronectin	VTN	1	8	5	22.2	54.305	39515000	78315000
Q7Z7K6	Q7Z7K6	Centromere protein V	CENPV	1	5	5	26.5	29.946	100240000	70986000
P63173	P63173	60S ribosomal protein L38	RPL38	1	5	5	50	8.2178	60790000	70461000
Q12792	Q12792	Twinfilin-1	TWF1	1	5	5	20.3	40.282	43228000	56475000
O75955	O75955	Flotillin-1	FLOT1	1	5	5	15.5	47.355	66150000	42094000
P39060	P39060	Collagen alpha-1(XVIII) chain;Endostatin	COL18A1	1	5	5	4.3	178.19	19258000	41303000
Q6ZUT6	Q6ZUT6	Uncharacterized protein C15orf52	C15orf52	1	5	5	14.8	57.324	41772000	35777000
Q8WXF1	Q8WXF1	Paraspeckle component 1	PSPC1	1	6	5	15.1	58.743	26314000	35056000
O43293	O43293	Death-associated protein kinase 3	DAPK3	1	5	5	13.2	52.535	31089000	29797000
Q9NW13	Q9NW13	RNA-binding protein 28	RBM28	1	5	5	8.3	85.737	51782000	22398000
Q14137	Q14137	Ribosome biogenesis protein BOP1	BOP1	1	5	5	11.3	83.629	37319000	16448000
Q14974	Q14974	Importin subunit beta-1	KPNB1	1	5	5	7.6	97.169	47365000	16380000
P05091	P05091	Aldehyde dehydrogenase, mitochondrial	ALDH2	1	7	5	19.7	56.381	10786000	14991000

Q9Y230	Q9Y230	RuvB-like 2	RUVBL2	1	5	5	14.5	51.156	14357000	10676000
P62316	P62316	Small nuclear ribonucleoprotein Sm D2	SNRPD2	1	5	5	41.5	13.527	109420000	44071000
Q9GZR7	Q9GZR7	ATP-dependent RNA helicase DDX24	DDX24	1	5	5	8.3	96.331	47282000	NaN
P84090	P84090	Enhancer of rudimentary homolog	ERH	1	5	5	45.2	12.259	3087600000	3141400000
P68032;P62736;P63267;P6813	P68032;P62736;P63267;P681	Actin, alpha cardiac muscle 1;Actin, aortic smo	ACTC1;ACTA2;ACTG2;AC	4	18	5	43.5	42.019	1307800000	2112400000
Q3MHD2	Q3MHD2	Protein LSM12 homolog	LSM12	1	5	5	28.2	21.701	346310000	212110000
P62899	P62899	60S ribosomal protein L31	RPL31	1	5	5	40	14.463	510500000	651410000
P62244	P62244	40S ribosomal protein S15a	RPS15A	1	5	5	36.9	14.839	240780000	166260000
P62851	P62851	40S ribosomal protein S25	RPS25	1	5	5	29.6	13.742	512620000	383260000
P63244	P63244	Guanine nucleotide-binding protein subunit b	GNB2L1	1	5	5	26.2	35.076	49445000	70352000
Q02543	Q02543	60S ribosomal protein L18a	RPL18A	1	5	5	30.1	20.762	119630000	205890000
P19105;O14950	P19105;O14950	Myosin regulatory light chain 12A;Myosin regu	MYL12A;MYL12B	2	12	5	63.2	19.794	4962400000	8620200000
Q9BSD7	Q9BSD7	Cancer-related nucleoside-triphosphatase	NTPCR	1	5	5	35.3	20.713	25717000	69280000
P22061	P22061	Protein-L-isoaspartate(D-aspartate) O-methyl	PCMT1	1	5	5	28.2	24.636	48228000	36725000
P60981	P60981	Destrin	DSTN	1	5	5	29.1	18.506	39605000	75664000
Q00325	Q00325	Phosphate carrier protein, mitochondrial	SLC25A3	1	5	5	16.9	40.094	99768000	220660000
P55081	P55081	Microfibrillar-associated protein 1	MFAP1	1	5	5	17.1	51.958	46816000	NaN
P06733;P13929;P09104	P06733	Alpha-enolase	ENO1	3	5	5	16.8	47.168	40218000	NaN
O60218	O60218	Aldo-keto reductase family 1 member B10	AKR1B10	1	7	5	31.3	36.019	77630000	70966000
P09382	P09382	Galectin-1	LGALS1	1	5	5	45.2	14.716	31604000	68781000
P63104	P63104	14-3-3 protein zeta/delta	YWHAZ	1	7	5	34.3	27.745	47103000	75120000
Q9NUQ6	Q9NUQ6	SPATS2-like protein	SPATS2L	1	5	5	10.4	61.728	NaN	31670000
P07900;Q14568	P07900	Heat shock protein HSP 90-alpha	HSP90AA1	2	7	5	10	84.659	25777000	NaN
Q04837	Q04837	Single-stranded DNA-binding protein, mitoch	SSBP1	1	5	5	41.9	17.259	88195000	529350000
Q86XZ4	Q86XZ4	Spermatogenesis-associated serine-rich prote	SPATS2	1	5	5	16.5	59.544	43686000	11661000
O14949	O14949	Cytochrome b-c1 complex subunit 8	UQCRQ	1	5	5	46.3	9.9062	34069000	61440000
O95816	O95816	BAG family molecular chaperone regulator 2	BAG2	1	5	5	30.8	23.772	37400000	31116000
P61981;Q04917	P61981	14-3-3 protein gamma;14-3-3 protein gamma	YWHAG	2	6	5	25.5	28.302	45770000	29627000
Q9Y265	Q9Y265	RuvB-like 1	RUVBL1	1	5	5	17.8	50.227	27329000	16567000
P59998	P59998	Actin-related protein 2/3 complex subunit 4	ARPC4	1	5	5	30.4	19.667	69838000	163550000
Q9HCE1	Q9HCE1	Putative helicase MOV-10	MOV10	1	5	5	7.4	113.67	29049000	NaN
Q16629	Q16629	Serine/arginine-rich splicing factor 7	SRSF7	1	6	5	25.2	27.366	71805000	19673000
P00390	P00390	Glutathione reductase, mitochondrial	GSR	1	5	5	16.3	56.256	21896000	39399000
P23588	P23588	Eukaryotic translation initiation factor 4B	EIF4B	1	5	5	9.2	69.15	11956000	9056500
P55769	P55769	NHP2-like protein 1;NHP2-like protein 1, N-te	NHP2L1	1	6	6	42.2	14.173	385210000	175610000
O15143	O15143	Actin-related protein 2/3 complex subunit 1B	ARPC1B	1	6	6	27.4	40.949	84063000	126780000
Q00059	Q00059	Transcription factor A, mitochondrial	TFAM	1	6	6	25.6	29.096	25569000	125120000
P40429;Q6NVV1	P40429	60S ribosomal protein L13a	RPL13A	2	6	6	31	23.577	37174000	60731000
P62714;P67775;P60510	P62714;P67775	Serine/threonine-protein phosphatase 2A cata	PPP2CB;PPP2CA	3	6	6	29.4	35.575	16895000	35992000
Q0ZGT2	Q0ZGT2	Nexilin	NEXN	1	6	6	12.7	80.657	74912000	34869000
P80723	P80723	Brain acid soluble protein 1	BASP1	1	6	6	50.7	22.693	15066000	31975000
O14639	O14639	Actin-binding LIM protein 1	ABLIM1	1	6	6	11.1	87.687	23632000	24518000
Q5BKZ1	Q5BKZ1	DBIRD complex subunit ZNF326	ZNF326	1	6	6	14.8	65.653	58014000	18173000
Q15459	Q15459	Splicing factor 3A subunit 1	SF3A1	1	6	6	10.1	88.885	59748000	14892000
P42167	P42167	Lamina-associated polypeptide 2, isoforms be	TMPO	1	15	6	49.6	50.67	NaN	209260000
Q9Y6M1	Q9Y6M1	Insulin-like growth factor 2 mRNA-binding pro	IGF2BP2	1	7	6	17.2	66.121	29092000	41100000
P53007	P53007	Tricarboxylate transport protein, mitochondrial	SLC25A1	1	6	6	19	34.012	57663000	46060000
O95758	O95758	Polypyrimidine tract-binding protein 3	PTBP3	1	9	6	19	59.689	82288000	39270000
P27816	P27816	Microtubule-associated protein 4	MAP4	1	6	6	8.2	121	19380000	63805000
P61513;A6NKH3	P61513	60S ribosomal protein L37a	RPL37A	2	6	6	58.7	10.275	846070000	563060000
Q9BTL3;A0A3B3IU46	Q9BTL3;A0A3B3IU46	RNMT-activating mini protein	FAM103A1	2	6	6	53.4	14.381	261380000	125480000
P05387	P05387	60S acidic ribosomal protein P2	RPLP2	1	6	6	64.3	11.665	196030000	454820000
P62277	P62277	40S ribosomal protein S13	RPS13	1	6	6	43	17.222	196560000	316510000
P62888	P62888	60S ribosomal protein L30	RPL30	1	6	6	59.1	12.784	171310000	511970000
Q99417	Q99417	C-Myc-binding protein	MYCBP	1	6	6	67	11.967	293300000	213740000
P05141;Q9H0C2	P05141	ADP/ATP translocase 2;ADP/ATP translocase 2	SLC25A5	2	13	6	32.2	32.852	219400000	285670000
P62913	P62913	60S ribosomal protein L11	RPL11	1	6	6	32.6	20.252	259980000	334410000
P08238;Q58FF8;Q58FF7	P08238	Heat shock protein HSP 90-beta	HSP90AB1	3	8	6	14.6	83.263	54025000	38686000
P18077	P18077	60S ribosomal protein L35a	RPL35A	1	6	6	39.1	12.538	112860000	127390000

P61353	P61353	60S ribosomal protein L27	RPL27	1	6	6	44.9	15.798	123550000	250400000
P31689	P31689	DnaJ homolog subfamily A member 1	DNAJA1	1	6	6	18.9	44.868	NaN	27146000
Q07020	Q07020	60S ribosomal protein L18	RPL18	1	6	6	36.7	21.634	147360000	154010000
P55795	P55795	Heterogeneous nuclear ribonucleoprotein H2	HNRNPH2	1	11	6	35.4	49.263	97902000	61119000
Q9Y6N5	Q9Y6N5	Sulfide:quinone oxidoreductase, mitochondrial	SQRDL	1	6	6	19.6	49.96	29392000	48152000
Q6NZI2	Q6NZI2	Polymerase I and transcript release factor	PTRF	1	6	6	23.6	43.476	100080000	54630000
P26373	P26373	60S ribosomal protein L13	RPL13	1	6	6	29.4	24.261	79161000	141740000
P60842;Q14240	P60842;Q14240	Eukaryotic initiation factor 4A-I;Eukaryotic initia	EIF4A1;EIF4A2	2	7	6	20.4	46.153	NaN	23565000
Q5SSJ5	Q5SSJ5	Heterochromatin protein 1-binding protein 3	HP1BP3	1	6	6	11.2	61.206	130150000	31742000
P49368	P49368	T-complex protein 1 subunit gamma	CCT3	1	6	6	13.6	60.533	NaN	19188000
P38919	P38919	Eukaryotic initiation factor 4A-III;Eukaryotic init	EIF4A3	1	7	6	18.7	46.871	48062000	21125000
P53396	P53396	ATP-citrate synthase	ACLY	1	6	6	7.3	120.84	11965000	15287000
P51648	P51648	Fatty aldehyde dehydrogenase	ALDH3A2	1	7	6	18.8	54.847	18137000	18602000
O95084	O95084	Serine protease 23	PRSS23	1	7	7	29	43.001	124710000	252620000
Q9P0M6	Q9P0M6	Core histone macro-H2A.2	H2AFY2	1	8	7	26.9	40.058	164080000	71373000
Q92743	Q92743	Serine protease HTRA1	HTRA1	1	7	7	16.2	51.286	54321000	47053000
P11387	P11387	DNA topoisomerase 1	TOP1	1	7	7	11.9	90.725	46697000	44346000
Q01813;P17858	Q01813	ATP-dependent 6-phosphofructokinase, plate	PFKP	2	7	7	11.7	85.595	52100000	33560000
Q9BQG0	Q9BQG0	Myb-binding protein 1A	MYBBP1A	1	7	7	6.9	148.85	51089000	32755000
Q8IVT2	Q8IVT2	Mitotic interactor and substrate of PLK1	MISP	1	7	7	16.2	75.356	53011000	32663000
Q14254	Q14254	Flotillin-2	FLOT2	1	7	7	20.1	47.064	54300000	28192000
Q9UKM9	Q9UKM9	RNA-binding protein Raly	RALY	1	7	7	25.8	32.463	78075000	23520000
Q16555;Q14194;Q14195	Q16555	Dihydropyrimidinase-related protein 2	DPYSL2	3	7	7	18.5	62.293	17144000	22342000
Q13501	Q13501	Sequestosome-1	SQSTM1	1	7	7	26.1	47.687	58899000	20227000
Q12874	Q12874	Splicing factor 3A subunit 3	SF3A3	1	7	7	18.8	58.848	47716000	9456400
P27105	P27105	Erythrocyte band 7 integral membrane protein	STOM	1	7	7	29.2	31.73	96837000	57163000
Q07666;O75525;Q5VWX1	Q07666	KH domain-containing, RNA-binding, signal tr	KHDRBS1	3	7	7	20.5	48.227	418800000	504380000
P04156	P04156	Major prion protein	PRNP	1	7	7	24.9	27.661	156840000	226060000
Q86V81	Q86V81	THO complex subunit 4	ALYREF	1	7	7	36.2	26.888	1060400000	1047500000
P62263	P62263	40S ribosomal protein S14	RPS14	1	7	7	39.7	16.273	600150000	814330000
P62266	P62266	40S ribosomal protein S23	RPS23	1	7	7	49	15.807	349480000	261590000
P60660	P60660	Myosin light polypeptide 6	MYL6	1	9	7	73.5	16.93	8869700000	13101000000
P83731	P83731	60S ribosomal protein L24	RPL24	1	7	7	36.3	17.779	82925000	170720000
P27635;Q96L21	P27635	60S ribosomal protein L10	RPL10	2	7	7	33.2	24.577	89687000	267880000
P62917	P62917	60S ribosomal protein L8	RPL8	1	7	7	27.2	28.024	79085000	262180000
P18621	P18621	60S ribosomal protein L17	RPL17	1	7	7	38.6	21.397	154560000	240090000
Q13151	Q13151	Heterogeneous nuclear ribonucleoprotein A0	HNRNPA0	1	7	7	27.5	30.84	186170000	148100000
P62826	P62826	GTP-binding nuclear protein Ran	RAN	1	7	7	30.1	24.423	190010000	74671000
P0DMV9;P0DMV8	P0DMV9;P0DMV8	Heat shock 70 kDa protein 1B;Heat shock 70 k	HSPA1B;HSPA1A	2	14	7	27.8	70.051	76105000	109610000
P62906	P62906	60S ribosomal protein L10a	RPL10A	1	7	7	30	24.831	125830000	331430000
P32969	P32969	60S ribosomal protein L9	RPL9	1	7	7	50.5	21.863	165420000	223610000
P62081	P62081	40S ribosomal protein S7	RPS7	1	7	7	33	22.127	114740000	128120000
Q9NX20	Q9NX20	39S ribosomal protein L16, mitochondrial	MRPL16	1	7	7	31.1	28.449	54467000	41739000
Q14694	Q14694	Ubiquitin carboxyl-terminal hydrolase 10	USP10	1	7	7	14.8	87.133	NaN	29093000
P16989	P16989	Y-box-binding protein 3	YBX3	1	9	7	45.7	40.089	54705000	125830000
P17812;Q9NRF8	P17812	CTP synthase 1	CTPS1	2	7	7	14.6	66.69	16319000	38631000
P13639	P13639	Elongation factor 2	EEF2	1	7	7	10	95.337	28786000	23267000
P11586	P11586	C-1-tetrahydrofolate synthase, cytoplasmic;Me	MTHFD1	1	7	7	8.8	101.53	14874000	15742000
Q14204	Q14204	Cytoplasmic dynein 1 heavy chain 1	DYNC1H1	1	7	7	1.9	532.4	36430000	31769000
P46777	P46777	60S ribosomal protein L5	RPL5	1	7	7	29.6	34.362	74284000	53832000
Q9NZN4;Q9NZN3	Q9NZN4	EH domain-containing protein 2	EHD2	2	8	7	16.4	61.161	24331000	49615000
Q04637;O43432	Q04637	Eukaryotic translation initiation factor 4 gamma	EIF4G1	2	7	7	5.3	175.49	NaN	NaN
P55084	P55084	Trifunctional enzyme subunit beta, mitochondr	HADHB	1	7	7	20.5	51.294	25570000	44705000
P61160	P61160	Actin-related protein 2	ACTR2	1	8	8	26.4	44.76	54150000	122620000
Q16643	Q16643	Drebrin	DBN1	1	8	8	21.4	71.428	137250000	65293000
O00622	O00622	Protein CYR61	CYR61	1	8	8	22.3	42.026	52859000	57540000
Q9H307	Q9H307	Pinin	PNN	1	8	8	12.3	81.627	66421000	19348000
Q9Y490	Q9Y490	Talin-1	TLN1	1	8	8	5.9	269.76	16194000	15378000
Q03701	Q03701	CCAAT/enhancer-binding protein zeta	CEBPZ	1	8	8	9.1	120.97	52316000	12422000

P06753	P06753	Tropomyosin alpha-3 chain	TPM3	1	24	8	49.5	32.95	900250000	1690700000
Q86SQ0	Q86SQ0	Pleckstrin homology-like domain family B mer	PHLDB2	1	8	8	8.7	142.16	29298000	30748000
O75533	O75533	Splicing factor 3B subunit 1	SF3B1	1	8	8	9.1	145.83	76105000	26019000
Q13242	Q13242	Serine/arginine-rich splicing factor 9	SRSF9	1	8	8	32.6	25.542	128760000	45828000
Q08945	Q08945	FACT complex subunit SSRP1	SSRP1	1	8	8	12.8	81.074	85322000	8899800
P02511	P02511	Alpha-crystallin B chain	CRYAB	1	8	8	59.4	20.159	12284000	243920000
Q01844	Q01844	RNA-binding protein EWS	EWSR1	1	8	8	16	68.477	2419600000	2408500000
P68104;Q5VTE0;Q05639	P68104;Q5VTE0;Q05639	Elongation factor 1-alpha 1;Putative elongatio	EEF1A1;EEF1A1P5;EEF1A2	3	8	8	24.5	50.14	271730000	463650000
Q52LJ0	Q52LJ0	Protein FAM98B	FAM98B	1	11	8	34.9	45.547	191550000	267100000
P05388;Q8NHW5	P05388;Q8NHW5	60S acidic ribosomal protein P0;60S acidic ribo	RPLP0;RPLP0P6	2	8	8	36	34.273	242390000	695310000
P67809;Q9Y2T7	P67809	Nuclease-sensitive element-binding protein 1	YBX1	2	10	8	46	35.924	215280000	216810000
O14979	O14979	Heterogeneous nuclear ribonucleoprotein D-li	HNRNPDL	1	8	8	17.1	46.437	276700000	230590000
Q15637	Q15637	Splicing factor 1	SF1	1	8	8	13.5	68.329	70825000	23264000
P48047	P48047	ATP synthase subunit O, mitochondrial	ATP5O	1	8	8	46	23.277	113110000	271940000
P08708	P08708	40S ribosomal protein S17	RPS17	1	8	8	46.7	15.55	115040000	248390000
P15121	P15121	Aldose reductase	AKR1B1	1	9	8	38	35.853	190730000	181080000
P04792	P04792	Heat shock protein beta-1	HSPB1	1	8	8	52.7	22.782	178350000	394450000
P27348	P27348	14-3-3 protein theta	YWHAQ	1	10	8	42.4	27.764	94762000	108400000
P52597	P52597	Heterogeneous nuclear ribonucleoprotein F;He	HNRNPF	1	10	8	30.8	45.671	89662000	127390000
Q14847	Q14847	LIM and SH3 domain protein 1	LASP1	1	8	8	33.7	29.717	36259000	47666000
P43490	P43490	Nicotinamide phosphoribosyltransferase	NAMPT	1	8	8	22	55.52	54779000	73324000
P0DP25;P0DP24;P0DP23	P0DP25;P0DP24;P0DP23			3	8	8	55.7	16.837	1026300000	479080000
P41091;Q2VIR3	P41091;Q2VIR3	Eukaryotic translation initiation factor 2 subuni	EIF2S3;EIF2S3L	2	8	8	25.2	51.109	101200000	83301000
Q06210;O94808	Q06210	Glutamine--fructose-6-phosphate aminotrans	GFPT1	2	8	8	15.6	78.806	21611000	26300000
Q8NBQ5	Q8NBQ5	Estradiol 17-beta-dehydrogenase 11	HSD17B11	1	8	8	31.3	32.963	108710000	200180000
P30837	P30837	Aldehyde dehydrogenase X, mitochondrial	ALDH1B1	1	9	8	23.2	57.249	NaN	41594000
Q14192	Q14192	Four and a half LIM domains protein 2	FHL2	1	8	8	36.2	32.193	22973000	111110000
P05121	P05121	Plasminogen activator inhibitor 1	SERPINE1	1	9	9	27.9	45.059	63025000	189420000
O75367	O75367	Core histone macro-H2A.1	H2AFY	1	10	9	36.6	39.183	382000000	151560000
Q14257	Q14257	Reticulocalbin-2	RCN2	1	9	9	37.2	36.876	40825000	133100000
Q14690	Q14690	Protein RRP5 homolog	PDCD11	1	9	9	6.4	208.7	34471000	24748000
O75643	O75643	U5 small nuclear ribonucleoprotein 200 kDa he	SNRNP200	1	9	9	5.3	244.5	27790000	24539000
Q92614	Q92614	Unconventional myosin-XVIIIa	MYO18A	1	9	9	5.6	233.11	35801000	23419000
O60292;Q9P2F8;O43166	O60292	Signal-induced proliferation-associated 1-like	SIPA1L3	3	9	9	6.9	194.61	36977000	19423000
P14649	P14649	Myosin light chain 6B	MYL6B	1	11	9	53.8	22.764	8153900000	19969000000
P63010;Q10567	P63010	AP-2 complex subunit beta	AP2B1	2	9	9	11.5	104.55	25414000	56499000
P31943	P31943	Heterogeneous nuclear ribonucleoprotein H;H	HNRNPH1	1	15	9	47.4	49.229	341880000	207810000
P62249	P62249	40S ribosomal protein S16	RPS16	1	9	9	48.6	16.445	452170000	597790000
P21291	P21291	Cysteine and glycine-rich protein 1	CSRP1	1	9	9	64.2	20.567	299960000	829170000
P18124	P18124	60S ribosomal protein L7	RPL7	1	9	9	32.3	29.225	162960000	272480000
Q14157	Q14157	Ubiquitin-associated protein 2-like	UBAP2L	1	9	9	14.5	114.53	44487000	27347000
P36542	P36542	ATP synthase subunit gamma, mitochondrial	ATP5C1	1	9	9	32.2	32.996	49046000	191990000
P61313	P61313	60S ribosomal protein L15	RPL15	1	9	9	40.7	24.146	181100000	210410000
P04083	P04083	Annexin A1	ANXA1	1	9	9	33.8	38.714	69190000	57041000
P19525	P19525	Interferon-induced, double-stranded RNA-act	EIF2AK2	1	9	9	17.4	62.094	56975000	54027000
P17987	P17987	T-complex protein 1 subunit alpha	TCP1	1	9	9	21.9	60.343	24598000	33899000
Q8NFW8	Q8NFW8	N-acylneuraminate cytidylyltransferase	CMAS	1	10	10	33.4	48.379	69874000	159440000
P61158;Q9P1U1	P61158	Actin-related protein 3	ACTR3	2	10	10	37.6	47.371	75748000	142140000
P47755	P47755	F-actin-capping protein subunit alpha-2	CAPZA2	1	11	10	56.3	32.949	86584000	129440000
P46783;Q9NQ39	P46783;Q9NQ39	40S ribosomal protein S10;Putative 40S riboso	RPS10;RPS10P5	2	10	10	44.8	18.898	568030000	598610000
P62750	P62750	60S ribosomal protein L23a	RPL23A	1	10	10	42.3	17.695	421120000	503680000
P62241	P62241	40S ribosomal protein S8	RPS8	1	10	10	51	24.205	272920000	403320000
P62753	P62753	40S ribosomal protein S6	RPS6	1	10	10	39.4	28.68	210490000	378570000
P04406	P04406	Glyceraldehyde-3-phosphate dehydrogenase	GAPDH	1	10	10	35.5	36.053	339380000	263350000
Q14244	Q14244	Ensconsin	MAP7	1	10	10	16.2	84.051	202580000	88039000
P06748	P06748	Nucleophosmin	NPM1	1	10	10	43.9	32.575	1056200000	687550000
P46781	P46781	40S ribosomal protein S9	RPS9	1	10	10	43.3	22.591	176220000	229820000
P26196	P26196	Probable ATP-dependent RNA helicase DDX6	DDX6	1	10	10	24.4	54.416	78479000	62545000
P26599	P26599	Polypyrimidine tract-binding protein 1	PTBP1	1	13	10	29.6	59.632	284280000	219780000

Q15717;Q12926;P26378	Q15717	ELAV-like protein 1	ELAVL1	3	10	10	41.1	36.091	187490000	129360000
Q9NYF8	Q9NYF8	Bcl-2-associated transcription factor 1	BCLAF1	1	11	10	14	106.12	84978000	66422000
Q12905	Q12905	Interleukin enhancer-binding factor 2	ILF2	1	10	10	31.3	43.062	328830000	130630000
Q5T749	Q5T749	Keratinocyte proline-rich protein	KPRP	1	10	10	30.4	64.135	18173000	58187000
Q96HS1	Q96HS1	Serine/threonine-protein phosphatase PGAM5	PGAM5	1	10	10	35.6	32.004	41814000	28914000
P04843	P04843	Dolichyl-diphosphooligosaccharide--protein g	RPN1	1	10	10	20.4	68.569	22239000	87070000
P20700	P20700	Lamin-B1	LMNB1	1	15	11	27.1	66.408	60458000	47305000
Q13428	Q13428	Treacle protein	TCOF1	1	11	11	10.8	152.1	84183000	47245000
O43143;O60231;Q14562	O43143	Pre-mRNA-splicing factor ATP-dependent RN	DHX15	3	11	11	16.4	90.932	77179000	34633000
Q03252	Q03252	Lamin-B2	LMNB2	1	15	11	24.4	69.948	60726000	19782000
Q7Z2W4	Q7Z2W4	Zinc finger CCCH-type antiviral protein 1	ZC3HAV1	1	11	11	18.7	101.43	23752000	32879000
P62269	P62269	40S ribosomal protein S18	RPS18	1	11	11	51.3	17.718	1670500000	2123600000
Q16891	Q16891	MICOS complex subunit MIC60	IMMT	1	11	11	17.5	83.677	965920000	658930000
P15880	P15880	40S ribosomal protein S2	RPS2	1	11	11	39.6	31.324	109080000	284380000
P22695	P22695	Cytochrome b-c1 complex subunit 2, mitocho	UQCRC2	1	11	11	37.7	48.442	60386000	219390000
P30838;P48448;P43353	P30838	Aldehyde dehydrogenase, dimeric NADP-pref	ALDH3A1	3	12	11	32.5	50.394	104010000	50991000
Q14247	Q14247	Src substrate cortactin	CTTN	1	11	11	30.4	61.585	56679000	101980000
P19338	P19338	Nucleolin	NCL	1	11	11	20.4	76.613	60600000	139290000
P14923;P35222	P14923	Junction plakoglobin	JUP	2	11	11	18.3	81.744	79677000	44138000
O15144	O15144	Actin-related protein 2/3 complex subunit 2	ARPC2	1	11	11	32.3	34.333	34579000	72707000
Q96IZ0	Q96IZ0	PRKC apoptosis WT1 regulator protein	PAWR	1	12	12	55.9	36.567	804380000	985440000
P52907	P52907	F-actin-capping protein subunit alpha-1	CAPZA1	1	13	12	65.4	32.922	310920000	426070000
O60832	O60832	H/ACA ribonucleoprotein complex subunit 4	DKC1	1	12	12	25.7	57.673	192130000	58255000
O76021	O76021	Ribosomal L1 domain-containing protein 1	RSL1D1	1	12	12	25.1	54.972	85733000	30356000
Q6P2Q9	Q6P2Q9	Pre-mRNA-processing-splicing factor 8	PRPF8	1	12	12	5.8	273.6	37267000	6266900
P19474	P19474	E3 ubiquitin-protein ligase TRIM21	TRIM21	1	12	12	27.2	54.169	10946000	NaN
Q92804	Q92804	TATA-binding protein-associated factor 2N	TAF15	1	14	12	26.4	61.829	5370000000	3806500000
P31942	P31942	Heterogeneous nuclear ribonucleoprotein H3	HNRNPH3	1	13	12	54.6	36.926	546490000	464040000
Q14103	Q14103	Heterogeneous nuclear ribonucleoprotein D0	HNRNPD	1	12	12	38.3	38.434	1088500000	1075800000
P39023;Q92901	P39023	60S ribosomal protein L3	RPL3	2	12	12	32.8	46.108	464360000	1031100000
P62280	P62280	40S ribosomal protein S11	RPS11	1	12	12	57.6	18.431	926740000	819630000
P46782	P46782	40S ribosomal protein S5;40S ribosomal protei	RPS5	1	12	12	54.9	22.876	281360000	276950000
O60701	O60701	UDP-glucose 6-dehydrogenase	UGDH	1	12	12	28.5	55.023	50743000	125980000
Q06830;P32119;Q13162	Q06830	Peroxiredoxin-1	PRDX1	3	12	12	57.3	22.11	217580000	209610000
Q15424	Q15424	Scaffold attachment factor B1	SAFB	1	22	12	26.4	102.64	292600000	109320000
Q06787	Q06787	Fragile X mental retardation protein 1	FMR1	1	14	12	28.5	71.174	130200000	108640000
Q99700	Q99700	Ataxin-2	ATXN2	1	12	12	11.7	140.28	36419000	31307000
O14647;O14646	O14647	Chromodomain-helicase-DNA-binding protei	CHD2	2	12	12	8.7	211.34	51856000	15954000
Q9ULV4;Q6QEF8	Q9ULV4	Coronin-1C	CORO1C	2	13	13	34.6	53.248	180380000	87893000
Q8N3V7	Q8N3V7	Synaptopodin	SYNPO	1	13	13	18.5	99.462	172980000	68779000
P49916	P49916	DNA ligase 3	LIG3	1	13	13	14.2	112.91	2841900	67607000
P55265	P55265	Double-stranded RNA-specific adenosine dea	ADAR	1	13	13	14.3	136.06	58456000	29681000
P46087	P46087	Probable 28S rRNA (cytosine(4447)-C(5))-met	NOP2	1	13	13	19.5	89.301	79180000	23153000
P35637	P35637	RNA-binding protein FUS	FUS	1	15	13	25.9	53.425	3171000000	2210200000
Q9UN86	Q9UN86	Ras GTPase-activating protein-binding proteir	G3BP2	1	15	13	35.3	54.12	1433200000	2269400000
P61247	P61247	40S ribosomal protein S3a	RPS3A	1	13	13	48.1	29.945	508260000	641900000
P39019	P39019	40S ribosomal protein S19	RPS19	1	13	13	60	16.06	531140000	469270000
Q9Y285	Q9Y285	Phenylalanine--tRNA ligase alpha subunit	FARSA	1	13	13	28.7	57.563	36008000	132250000
P46940	P46940	Ras GTPase-activating-like protein IQGAP1	IQGAP1	1	13	13	11.4	189.25	95012000	105150000
P62805	P62805	Histone H4	HIST1H4A	1	13	13	58.3	11.367	16023000000	4803700000
Q9H0D6	Q9H0D6	5-3 exoribonuclease 2	XRN2	1	13	13	15.7	108.58	138610000	97670000
Q13310;P0CB38	Q13310	Polyadenylate-binding protein 4	PABPC4	2	22	13	32.1	70.782	129260000	57472000
Q02878	Q02878	60S ribosomal protein L6	RPL6	1	13	13	30.9	32.728	170730000	92104000
Q9H089	Q9H089	Large subunit GTPase 1 homolog	LSG1	1	13	13	23.9	75.225	53099000	45840000
P14866	P14866	Heterogeneous nuclear ribonucleoprotein L	HNRNPL	1	13	13	37.7	64.132	228360000	100270000
P06576	P06576	ATP synthase subunit beta, mitochondrial	ATP5B	1	13	13	33.6	56.559	35999000	146240000
Q7L2E3	Q7L2E3	Putative ATP-dependent RNA helicase DHX30	DHX30	1	13	13	13.1	133.94	22211000	35639000
Q15582	Q15582	Transforming growth factor-beta-induced pro	TGFBI	1	13	13	21.8	74.68	124590000	274110000
P54652	P54652	Heat shock-related 70 kDa protein 2	HSPA2	1	22	13	42.7	70.02	36343000	204250000

Q9Y2X3	Q9Y2X3	Nucleolar protein 58	NOP58	1	14	14	36.7	59.578	421770000	132300000
Q9BZL4	Q9BZL4	Protein phosphatase 1 regulatory subunit 12C	PPP1R12C	1	14	14	29.9	84.88	27871000	38321000
P47756	P47756	F-actin-capping protein subunit beta	CAPZB	1	14	14	54	30.628	226250000	251920000
Q8NCA5	Q8NCA5	Protein FAM98A	FAM98A	1	17	14	32.8	55.272	2847100000	1779900000
P23396	P23396	40S ribosomal protein S3	RPS3	1	14	14	58.4	26.688	248480000	257110000
Q9BZZ5	Q9BZZ5	Apoptosis inhibitor 5	API5	1	14	14	29.6	59.004	335620000	269230000
P22087	P22087	rRNA 2-O-methyltransferase fibrillarin	FBL	1	15	14	43.9	33.784	916200000	280720000
P25705	P25705	ATP synthase subunit alpha, mitochondrial	ATP5A1	1	14	14	32	59.75	65566000	166000000
P62424	P62424	60S ribosomal protein L7a	RPL7A	1	14	14	39.8	29.995	153740000	244370000
Q9NYL2	Q9NYL2	Mitogen-activated protein kinase kinase kinase	ZAK	1	14	14	20.9	91.154	35754000	73525000
P50402	P50402	Emerin	EMD	1	14	14	65.4	28.994	98311000	989120000
P36578	P36578	60S ribosomal protein L4	RPL4	1	14	14	31.6	47.697	123670000	129740000
Q9NR12	Q9NR12	PDZ and LIM domain protein 7	PDLIM7	1	14	14	43.8	49.844	42796000	172600000
P09493	P09493	Tropomyosin alpha-1 chain	TPM1	1	30	14	56.7	32.708	1460300000	2261600000
O43390	O43390	Heterogeneous nuclear ribonucleoprotein R	HNRNPR	1	18	14	32.4	70.942	365500000	60333000
Q9NR30;Q9BQ39	Q9NR30	Nucleolar RNA helicase 2	DDX21	2	14	14	22.7	87.343	185260000	22952000
Q13045	Q13045	Protein flightless-1 homolog	FLII	1	15	15	15.1	144.75	31824000	96992000
Q8NC56	Q8NC56	LEM domain-containing protein 2	LEMD2	1	15	15	33.4	56.974	2689900	311410000
Q13283	Q13283	Ras GTPase-activating protein-binding protein	G3BP1	1	17	15	47.4	52.164	266680000	535750000
P09651;Q32P51;A0A2R8Y4L2	P09651;Q32P51;A0A2R8Y4L2	Heterogeneous nuclear ribonucleoprotein A1;I	HNRNPA1;HNRNPA1L2	3	16	15	46	38.746	1372500000	572710000
P61978	P61978	Heterogeneous nuclear ribonucleoprotein K	HNRNPK	1	15	15	35.9	50.976	378940000	314890000
Q9NYL9	Q9NYL9	Tropomodulin-3	TMOD3	1	17	16	56.8	39.594	153790000	238990000
P46821;P78559	P46821	Microtubule-associated protein 1B;MAP1B hea	MAP1B	2	16	16	10.3	270.63	26855000	28550000
Q9Y5B9	Q9Y5B9	FACT complex subunit SPT16	SUPT16H	1	16	16	17	119.91	163790000	16503000
P67936;Q2TAC2	P67936	Tropomyosin alpha-4 chain	TPM4	2	30	16	73	28.521	923320000	991690000
P07910;P0DMR1;O60812;B7ZW	P07910;P0DMR1;O60812;B7	Heterogeneous nuclear ribonucleoproteins C1	HNRNPC;HNRNPCL4;HNI	6	16	16	36.9	33.67	1625000000	557430000
Q8ND56	Q8ND56	Protein LSM14 homolog A	LSM14A	1	16	16	33	50.529	849870000	541510000
P62701;Q8TD47;P22090	P62701	40S ribosomal protein S4, X isoform	RPS4X	3	16	16	57.8	29.597	1085400000	743930000
Q92841	Q92841	Probable ATP-dependent RNA helicase DDX17	DDX17	1	23	16	37.2	80.272	155940000	171490000
Q9NZI8	Q9NZI8	Insulin-like growth factor 2 mRNA-binding pro	IGF2BP1	1	18	16	37.8	63.48	485850000	342400000
P21980	P21980	Protein-glutamine gamma-glutamyltransferase	TGM2	1	16	16	27.8	77.328	160360000	151270000
Q6ZRV2	Q6ZRV2	Protein FAM83H	FAM83H	1	16	16	20.6	127.12	27165000	72049000
Q69YQ0	Q69YQ0	Cytospin-A	SPECC1L	1	17	17	18.7	124.54	83948000	88158000
Q5M775	Q5M775	Cytospin-B	SPECC1	1	17	17	20.1	118.58	1305900000	617370000
P51991	P51991	Heterogeneous nuclear ribonucleoprotein A3	HNRNPA3	1	17	17	37.6	39.594	1876700000	890340000
P49411	P49411	Elongation factor Tu, mitochondrial	TUFM	1	17	17	46.2	49.874	139720000	365420000
O60506	O60506	Heterogeneous nuclear ribonucleoprotein Q	SYNCRIP	1	21	17	44.3	69.602	318610000	160860000
P38646	P38646	Stress-70 protein, mitochondrial	HSPA9	1	17	17	30.6	73.68	105610000	197500000
Q96EK7	Q96EK7	Constitutive coactivator of peroxisome prolifer	FAM120B	1	17	17	24.5	103.78	28239000	36667000
P40939	P40939	Trifunctional enzyme subunit alpha, mitochonc	HADHA	1	17	17	30.3	82.999	45467000	123210000
Q9Y224	Q9Y224	UPF0568 protein C14orf166	C14orf166	1	18	18	67.2	28.068	1887900000	1542200000
P11142	P11142	Heat shock cognate 71 kDa protein	HSPA8	1	27	18	49.4	70.897	256850000	602610000
O43148	O43148	mRNA cap guanine-N7 methyltransferase	RNMT	1	18	18	38.9	54.843	127100000	111230000
P11413	P11413	Glucose-6-phosphate 1-dehydrogenase	G6PD	1	18	18	42.1	59.256	116540000	139490000
P00352;P47895;O94788	P00352	Retinal dehydrogenase 1	ALDH1A1	3	20	18	48.1	54.861	78105000	102200000
Q9NZB2;Q5T035	Q9NZB2	Constitutive coactivator of PPAR-gamma-like	FAM120A	2	18	18	23.8	121.89	96139000	88825000
Q07065	Q07065	Cytoskeleton-associated protein 4	CKAP4	1	18	18	41	66.022	64778000	86109000
P17844	P17844	Probable ATP-dependent RNA helicase DDX5	DDX5	1	26	19	37.9	69.147	416050000	252320000
P14618;P30613	P14618	Pyruvate kinase PKM	PKM	2	19	19	44.6	57.936	123880000	114480000
P07355;A6NMY6	P07355;A6NMY6	Annexin A2;Putative annexin A2-like protein	ANXA2;ANXA2P2	2	19	19	51.6	38.604	465280000	790040000
P11021	P11021	78 kDa glucose-regulated protein	HSPA5	1	20	19	34.9	72.332	370480000	577210000
Q1KMD3	Q1KMD3	Heterogeneous nuclear ribonucleoprotein U-li	HNRNPUL2	1	19	19	30.9	85.104	326310000	88051000
Q9Y520	Q9Y520	Protein PRRC2C	PRRC2C	1	19	19	10.1	316.91	80703000	72408000
P43243	P43243	Matrin-3	MATR3	1	19	19	27.4	94.622	236750000	108490000
Q6ZMP0	Q6ZMP0	Thrombospondin type-1 domain-containing p	THSD4	1	19	19	30.5	112.45	44434000	160710000
O00159	O00159	Unconventional myosin-Ic	MYO1C	1	19	19	22.8	121.68	315720000	39840000
P38159;Q96E39;O75526;Q8N7	P38159;Q96E39	RNA-binding motif protein, X chromosome;RN	RBMX;RBMXL1	4	20	20	46.8	42.331	1011400000	361100000
Q7Z417	Q7Z417	Nuclear fragile X mental retardation-interactin	NUFIP2	1	20	20	37.7	76.12	283560000	144660000
P11940;Q9H361;Q4VXU2;Q96D	P11940	Polyadenylate-binding protein 1	PABPC1	4	29	20	46.9	70.67	436720000	282820000

Q9BUJ2	Q9BUJ2	Heterogeneous nuclear ribonucleoprotein U-li	HNRNPUL1	1	20	20	29.8	95.737	127590000	148270000
Q9Y2W1	Q9Y2W1	Thyroid hormone receptor-associated protein	THRAP3	1	21	20	22.7	108.66	140470000	176780000
P51116	P51116	Fragile X mental retardation syndrome-relatec	FXR2	1	23	20	40.3	74.222	205240000	267650000
P12814	P12814	Alpha-actinin-1	ACTN1	1	30	20	43.6	103.06	512030000	297470000
P49792;A6NKT7;Q7Z3J3;Q996	P49792	E3 SUMO-protein ligase RanBP2	RANBP2	7	21	21	9.8	358.2	66601000	4649700
Q14444	Q14444	Caprin-1	CAPRIN1	1	21	21	34.6	78.365	964450000	1028700000
Q14764	Q14764	Major vault protein	MVP	1	21	21	30.2	99.326	76739000	23153000
Q16352;P07197	Q16352	Alpha-internexin	INA	2	24	21	46.5	55.39	297510000	86252000
Q9UHB6	Q9UHB6	LIM domain and actin-binding protein 1	LIMA1	1	22	22	36.9	85.225	366330000	133020000
P83111	P83111	Serine beta-lactamase-like protein LACTB, mit	LACTB	1	22	22	41	60.693	53540000	249010000
O00425	O00425	Insulin-like growth factor 2 mRNA-binding pro	IGF2BP3	1	24	22	47.5	63.704	412950000	249660000
O00567	O00567	Nucleolar protein 56	NOP56	1	23	23	45.1	66.049	411450000	122260000
Q8NC51	Q8NC51	Plasminogen activator inhibitor 1 RNA-binding	SERBP1	1	23	23	51.2	44.965	761540000	803030000
P51114	P51114	Fragile X mental retardation syndrome-relatec	FXR1	1	25	23	43.5	69.72	244870000	280470000
Q12906	Q12906	Interleukin enhancer-binding factor 3	ILF3	1	26	23	40	95.337	460780000	206260000
Q96PK6	Q96PK6	RNA-binding protein 14	RBM14	1	24	24	38.9	69.491	835890000	775350000
Q92900	Q92900	Regulator of nonsense transcripts 1	UPF1	1	24	24	30.1	124.34	61013000	66870000
O43707;P35609;Q08043	O43707	Alpha-actinin-4	ACTN4	3	35	24	49.4	104.85	721120000	327120000
P12956	P12956	X-ray repair cross-complementing protein 6	XRCC6	1	25	25	41.1	69.842	133720000	678160000
P22626	P22626	Heterogeneous nuclear ribonucleoproteins A2	HNRNPA2B1	1	26	25	71.1	37.429	4615500000	1847600000
P27708	P27708	CAD protein;Glutamine-dependent carbamoyl	CAD	1	25	25	15.1	242.98	8154300	9922400
Q86V48	Q86V48	Leucine zipper protein 1	LUZP1	1	26	26	31.1	120.27	100020000	117940000
P30041	P30041	Peroxiredoxin-6	PRDX6	1	26	26	85.7	25.035	15818000	12399000000
Q9P2E9;Q8N4C6	Q9P2E9	Ribosome-binding protein 1	RRBP1	2	26	26	25	152.45	54485000	82633000
O43795;Q9UBC5	O43795	Unconventional myosin-Ib	MYO1B	2	27	27	26.1	131.98	208730000	110920000
Q05682	Q05682	Caldesmon	CALD1	1	27	27	30.4	93.23	95204000	607050000
Q7Z406	Q7Z406	Myosin-14	MYH14	1	41	27	23.6	227.87	5468500000	5194000000
P15924	P15924	Desmoplakin	DSP	1	27	27	11.7	331.77	46222000	28122000
Q8WWI1	Q8WWI1	LIM domain only protein 7	LMO7	1	28	28	24.3	192.69	98933000	64503000
Q9BZF9	Q9BZF9	Uveal autoantigen with coiled-coil domains an	UACA	1	28	28	23.7	162.5	57713000	56720000
O14974	O14974	Protein phosphatase 1 regulatory subunit 12A	PPP1R12A	1	29	28	35.1	115.28	229790000	261200000
P13010	P13010	X-ray repair cross-complementing protein 5	XRCC5	1	28	28	35.4	82.704	286600000	1391700000
P78527	P78527	DNA-dependent protein kinase catalytic subur	PRKDC	1	28	28	9.9	469.08	36011000	177460000
Q00610;P53675	Q00610	Clathrin heavy chain 1	CLTC	2	28	28	23	191.61	217560000	116230000
Q15233	Q15233	Non-POU domain-containing octamer-bindin	NONO	1	31	29	57.3	54.231	1867900000	2069900000
Q8WWM7	Q8WWM7	Ataxin-2-like protein	ATXN2L	1	29	29	33.2	113.37	698760000	313310000
P02751	P02751	Fibronectin;Anastellin;Ugl-Y1;Ugl-Y2;Ugl-Y3	FN1	1	29	29	17.1	272.32	62112000	225970000
Q9Y3I0	Q9Y3I0	tRNA-splicing ligase RtcB homolog	RTCB	1	30	30	62.2	55.21	3146000000	1982700000
Q00839	Q00839	Heterogeneous nuclear ribonucleoprotein U	HNRNPU	1	30	30	37.3	90.583	2595000000	1598300000
O95425	O95425	Supervillin	SVIL	1	31	31	20.5	247.74	126160000	89894000
Q02880;P11388	Q02880	DNA topoisomerase 2-beta	TOP2B	2	31	31	20.5	183.26	171530000	48826000
P05783;CON__H-INV:HIT00001	P05783	Keratin, type I cytoskeletal 18	KRT18	5	44	32	81.4	48.057	13850000000	28623000000
Q9UPQ0	Q9UPQ0	LIM and calponin homology domains-containi	LIMCH1	1	33	33	39.5	121.87	51666000	279270000
Q08211	Q08211	ATP-dependent RNA helicase A	DHX9	1	33	33	33.8	140.96	462440000	215440000
O00571;O15523;Q9NQI0	O00571;O15523	ATP-dependent RNA helicase DDX3X;ATP-dep	DDX3X;DDX3Y	3	35	34	62.4	73.243	1160600000	782450000
Q01804	Q01804	OTU domain-containing protein 4	OTUD4	1	35	35	38.3	124.04	393840000	360060000
Q01082;P11277;O15020	Q01082	Spectrin beta chain, non-erythrocytic 1	SPTBN1	3	36	36	19.9	274.61	80096000	72993000
P52272	P52272	Heterogeneous nuclear ribonucleoprotein M	HNRNPM	1	40	40	58.1	77.515	818960000	689910000
P23246	P23246	Splicing factor, proline- and glutamine-rich	SFPQ	1	42	41	55.3	76.149	3746600000	3535900000
Q92499	Q92499	ATP-dependent RNA helicase DDX1	DDX1	1	41	41	53.1	82.431	2469700000	2127500000
O75369	O75369	Filamin-B	FLNB	1	46	41	25.5	278.16	100830000	51811000
Q13813	Q13813	Spectrin alpha chain, non-erythrocytic 1	SPTAN1	1	42	42	21.4	284.54	53017000	85559000
P02545	P02545	Prelamin-A/C;Lamin-A/C	LMNA	1	44	42	56.6	74.139	403120000	1854400000
P09874	P09874	Poly [ADP-ribose] polymerase 1	PARP1	1	48	48	48.7	113.08	120600000	841730000
Q9P0K7	Q9P0K7	Ankycorbin	RAI14	1	49	49	53.3	110.04	405980000	239650000
P08670;P17661;P41219;P1413	P08670	Vimentin	VIM	4	55	50	82.6	53.651	11088000000	3768900000
P35555	P35555	Fibrillin-1	FBN1	1	58	56	30.5	312.3	16773000	236850000
Q6WCQ1	Q6WCQ1	Myosin phosphatase Rho-interacting protein	MPRIP	1	57	56	63.5	116.53	854410000	493900000
P35580	P35580	Myosin-10	MYH10	1	114	83	55.1	229	657680000	252360000

P21333;Q14315	P21333	Filamin-A	FLNA	2	91	86	47.4	280.74	508640000	518160000
P35579;P12883;P13535;Q9UKX	P35579	Myosin-9	MYH9	10	219	188	75	226.53	7846500000	14672000000
Q15149;Q9UPN3	Q15149	Plectin	PLEC	2	261	256	56.3	531.78	1752500000	1227200000
